# A comprehensive study of the delay vector variance method for quantification of nonlinearity in dynamical systems

**DOI:** 10.1098/rsos.150493

**Published:** 2016-01-06

**Authors:** V. Jaksic, D. P. Mandic, K. Ryan, B. Basu, V. Pakrashi

**Affiliations:** 1 Dynamical Systems and Risk Laboratory, Civil and Environmental Engineering, School of Engineering, University College Cork, Cork, Republic of Ireland; 2 Department of Electrical and Electronic Engineering, Imperial College London, London, UK; 3 Department of Civil, Structural and Environmental Engineering, Trinity College Dublin, Dublin, Republic of Ireland

**Keywords:** delay vector variance, signal nonlinearity, structural dynamics, benchmarking, wind turbine blade

## Abstract

Although vibration monitoring is a popular method to monitor and assess dynamic structures, quantification of linearity or nonlinearity of the dynamic responses remains a challenging problem. We investigate the delay vector variance (DVV) method in this regard in a comprehensive manner to establish the degree to which a change in signal nonlinearity can be related to system nonlinearity and how a change in system parameters affects the nonlinearity in the dynamic response of the system. A wide range of theoretical situations are considered in this regard using a single degree of freedom (SDOF) system to obtain numerical benchmarks. A number of experiments are then carried out using a physical SDOF model in the laboratory. Finally, a composite wind turbine blade is tested for different excitations and the dynamic responses are measured at a number of points to extend the investigation to continuum structures. The dynamic responses were measured using accelerometers, strain gauges and a Laser Doppler vibrometer. This comprehensive study creates a numerical and experimental benchmark for structurally dynamical systems where output-only information is typically available, especially in the context of DVV. The study also allows for comparative analysis between different systems driven by the similar input.

## 1. Introduction

Changes in mass, stiffness, natural frequency, etc., are often indicators of structural damage [1]. However, the complex, new and ageing structures of different sizes are often too hard to model [2,3] in their operational conditions and the excitations that they experience are immeasurable [4]. Therefore, various devices are employed to record structural response signals in order to monitor structural health. Statistical analyses and benchmarking of time or frequency domain dynamic responses of structures are popular in structural health monitoring (SHM) [4,5,6,7,8]. Therefore, in signal analysis of dynamical systems, there is often a need to assess and quantify the extent of nonlinearity in the response signal and possibly in the system [9]. In this regard, a theoretically well-established technique for detecting the nature or nonlinearity of time series is the surrogate data method [10], which was originally motivated by statistical hypothesis testing and presents an indirect way of detecting nonlinearity in a signal [11]. In recent literature, there is extensive research on signal linearity analysis based on surrogate data [12,13,14]. Failure to detect nonlinearity may result from an inappropriate choice of test statistic and there are also problems with artefacts occurring in the process of generating surrogate datasets [15]. Many non-parametric analysis techniques have been developed for the detection of nonlinearity in the signal [16].

Gautama *et al*. [17] discuss several established nonlinearity analysis methods for comparing and testing the degree of nonlinearity between populations of signals. They tested deterministic versus stochastic (DVS) plots proposed by Casdagli [18], traditional nonlinearity metrics [19], i.e. the third-order autocovariance (C3) and a measure of the deviation due to time reversibility, and the *δ*–*ε* method proposed by Kaplan [20]. Gautama *et al*. [17] also presented a novel test statistic for detecting the determinism and nonlinearity through a time series delay vector variance (DVV) method, which characterizes a time series based upon its predictability and compares the results to those obtained for linearized versions of the signal, i.e. surrogate data. The method is most similar to Kaplan’s *δ*–*ε* method and the false nearest neighbours [21]. The comparison of the above-listed nonlinearity methods (DVS method is excluded because it does not allow for a quantitative analysis) with DVV showed that only the DVV method consistently detected nonlinear behaviour for higher slopes and for all noise levels.

The advance technique for nonlinear data analysis is also a recurrence plot (RP) based on Takens’ embedding theorem [22]. It can visualize the dynamics of phase-space trajectories [23]. However, the RP is testing only for deterministic–stochastic behaviour, while DVV is testing deterministic–stochastic and linear–nonlinear behaviour of a signal. The aim of the DVV method is to verify whether or not a time series observed is generated by a linear stochastic system [24]. A DVV-based characterization does not require prior knowledge about the signal and is considered to be robust to the presence of noise and straightforward to interpret and visualize nonlinearity, while retaining superior performance over many other available methods [17]. The DVV method has been successfully applied for characterization of biomedical signals, such as functional magnetic resonance imaging [25,26] and heart rate variability [27,28].

Although DVV assesses signal nonlinearity rather than system nonlinearity, it is sometimes possible to attribute signal nonlinearity to system nonlinearity [25]. For example, when DVV has been applied in diagnostic medicine the aim was to assess the presence or the absence of nonlinear behaviour within the signal observed, as the linear or nonlinear nature of the signal conveys information concerning the health condition of the subject [24,29]. The DVV method has also been used as an efficient tool for acquiring the information on determinism and nonlinearity of response of mechanical systems [30]. A study on a diesel engine vibration in different conditions found that the vibration signals have strong nonlinearity and that nonlinearity gets stronger as a fault of a diesel engine becomes worse. The root mean square deviation of the DVV scatter diagram from the bisector line was used for a quantitative analysis of the fault state, which could be used to detect fault. Recently, DVV has been applied and tested in the field of SHM of offshore floating foundations [31,32,33] and bridges [34]. These are big dynamical systems which, due to their geometry and material complexity, are hard to model in a dynamically accurate manner since many parameters influence their behaviour. Furthermore, the nature of excitation force, e.g. deterministic or random, to these systems is in reality unknown and monitoring of the dynamic responses is only remotely possible, because of the structure’s difficult accessibility, interruption to its operation, etc. On the other hand, an understanding of the behaviour of floating platforms in normal or extreme weather situations and bridges under the operational loads is crucial for their safe operation.

Existing applications of DVV indicate the significant possibility of employing this method for assessing dynamical systems in relation to structural mechanics. However, no benchmark is present in terms of how, for different dynamical systems, the signal nonlinearities are assessed and to what extent. Our aim is to investigate, in detail, to what extent estimates of signal nonlinearity can distinguish between different systems, the excitation to the systems and the different parameters that guide the dynamics of the systems. In this regard, we have chosen a number of theoretical dynamical systems and have carried out a range of experimental analyses. A single degree of freedom (SDOF) system and a wind turbine blade (WTB) have been considered representative of a lumped mass system and of a continuum, respectively. The influences of measuring devices have also been considered, i.e. accelerometers, strain gauges and a Laser Doppler vibrometer (LDV) have been used to obtain the dynamic responses of the different experimental systems. This work attempts to create a comprehensive benchmark for dynamical systems with different parameters, input ranges and experimental validations in relation to estimates of nonlinearities computed from the dynamical system outputs using DVV.

## 2. Signal and system nonlinearity

The signal and system nonlinearity is defined as per Gautama *et al.* [26,28], i.e. a linear signal, *x*, is generally defined as the output of a linear shift-invariant system that is driven by Gaussian white noise [35]. However, in most cases, this definition has become flexible by allowing the probability distribution of the signal values, i.e. the signal distribution, to deviate from the Gaussian. This can be interpreted as a linear signal (following a strict definition) measured by a static (possibly nonlinear) observation function [28]. Any signal that cannot be generated in such a way is generally referred to as a nonlinear signal [28]. The principle of temporal summation for analysing the nonlinearity of a system implies that input and output time series can be measured simultaneously, while in typical real-world settings this is often not possible [25,28]. The analysis of the nonlinearity of a signal can often provide information on the nature of the underlying system. However, the assessment of nonlinearity within a signal does not necessarily imply that the underlying signal generation system is nonlinear.

The input signal and system nonlinearities may be confounded [17] and care should be taken in the interpretation of results. For example, if the input to the system is nonlinear and the system itself is linear, the measured signal at the output would be nonlinear. Therefore, a straightforward conclusion cannot be drawn from nonlinearity analysis of one signal for an underlying system, but this method allows for comparative analysis between different systems, driven by the same input. It is important to know what assumptions of nonlinearity analysis are so as not to confuse cause and effect [10].

## 3. Theoretical background

### 3.1 Delay vector variance

The DVV theory was initially introduced in Mandic & Chambers [36] and was further elaborated and tested by Gautama *et al.* [17,28] and Mandic *et al.* [26]. A background to DVV theory and discussion on embedded parameter choice methods is provided in the electronic supplementary material, appendix S1.

The DVV method is based on time delay embedding representation of a time series *x*(*n*), *n*=1,2,…,*N*. For a given embedding dimension *m*, the mean target variance, σ*^2^, is calculated over all sets of ‘neighbouring’ delay vectors *Ω*_*k*_. A set *Ω*_*k*_ is generated by grouping those DVs that are within a certain distance to **x**(*k*), which is varied in a manner standardized with respect to the distribution of pairwise distance between DVs. The DVV algorithm is given in this section for completeness and for establishing the context of the study and can be summarized as follows [17,24,28]:

(1) Reconstruct the phase-space and obtain the set of DVs in phase space
  3.1 $$\text{x}(k)={\left[x_{k-\tau m},\ldots ,x_{k-\tau }\right]}^{T},\;k=1,\ldots ,N-m+1,$$
  where *N* denotes the length of time series and *τ* denotes time delay.
(2) Compute the mean *μ*_d_ and standard deviation *σ*_d_ over all pairwise Euclidian distances between DVs [25]
  3.2 d(i,j)=∥x(i)−x(j)∥,(i≠j).
(3) The sets *Ω*_*k*_(*r*_d_) are generated so that
  3.3 $$\Omega_{k}(r_{d})=\{x(i)|∥x(k)-x(i)∥\leq r_{d}\},$$
  i.e. sets that consist of all DVs that lie closer to **x**(*k*) than the certain distance *r*_d_ calculated as:
  3.4 $$r_{d}(n)=\mu_{d}-n_{d}\sigma_{d}+(n-1)\frac{2n_{d}\sigma_{d}}{N_{\mathrm{tv}}-1};\;n=1,\ldots ,N_{\mathrm{tv}},$$
  where *n*_d_ is a parameter controlling the span over which to perform the DVV analysis, usually set to be 3 [37] and *N*_tv_, number of target variance, indicates how fine the standardized distance is uniformly spaced.
(4) For each set *Ω*_*k*_(*r*_d_), the variance of the corresponding targets $\sigma_{k}^{2}(r_{d})$ is computed. The average over all sets *Ω*_*k*_(*r*_d_) normalized by the variance of the time series, $\sigma_{x}^{2}$, yields the measure of unpredictability, ‘target variance’, σ*^2^(*r*_d_):
  3.5 $$\sigma^{*2}(r_{d})=\frac{\left(1/N)\sum_{k=1}^{N}\sigma_{k}^{2}(r_{d}\right)}{\sigma_{x}^{2}}.$$
  Jianjun *et al.* suggest that the set of *Ω*_*k*_(*r*_d_) should contain at least *N*_0_=30 DVs [38]. A sample of 30 data points for estimating mean or variance is the general rule-of-thumb [24,25,28].

The basis of the DVV method is that if two DVs of a predictable signal are close to one another in terms of their Euclidean distance, they should have similar targets, i.e. the smaller the Euclidian distance between them, the more similar targets they have. Hence, the presence of a strong deterministic component within a signal will result in the smaller target variances for small spans *r*_d_ [28,38]. The minimal target variance σ*^2^_min_=min_*r*_d__[σ*^2^(*r*_d_)] represents the amount of noise present within the time series (the prevalence of the stochastic component) and has an upper bound which is unity. The reason for this lies in the fact that all DVs belong to the same set of *Ω*_*k*_(*r*_d_) when *r*_d_ is sufficiently large. Therefore, the variance of the corresponding target of those DVs will be almost equal to that of the original time series. As a result of the standardization of the distance axes, the resulting DVV plots are straightforward to interpret. The resulting DVV plots are presented with the standardized distance *r*_d_ on the horizontal axis and normalized variance σ*^2^ on the vertical axis (see the electronic supplementary material, appendix S1). At the extreme right, DVV plots smoothly converge to unity, because for maximum spans all DVs belong to the same set, and the variance of the targets is equal to the variance of the time series. If this is not the case, the span parameter *n*_d_ should be increased [17,24].

Performing DVV analysis on the original and a number of surrogate time series, a scatter diagram can be produced. The DVV scatter diagram can characterize the linear or nonlinear nature of time series using the optimal embedding dimension of the original time series. If the surrogate time series yield DVV plots similar to the original time series, in which case the DVV scatter diagram coincides with the bisector line, then the original time series is likely to be linear [17,26]. Thus, the deviation from the bisector line is an indicator of nonlinearity of the original time series [17,28], i.e. the deviation from the bisector line grows as nonlinearity of a response increases (see the electronic supplementary material, appendices S1 and S3–S5).

The deviation from the bisector line can be quantified by the root mean squared error (RMSE) between the σ*^2^ values of the original time series and the σ*^2^ values averaged over the DVV plots of the surrogate time series (when computing this average, as well as computing RMSE, only the valid variance measurements, e.g. if the set *Ω*_*k*_(*r*_d_) contains at least 30 DVs measurements, are taken into account [24]). Thus, a single test statistic *t*^*DVV*^ is calculated [24] as

3.6 $$t^{\mathrm{DVV}}=\sqrt{{\left\langle {\left(\sigma^{*2}(r_{d})-\frac{\sum_{i=1}^{N_{s}}\sigma_{s,i}^{*2}(r_{d})}{N_{s}}\right)}^{2}\right\rangle }_{{\mathrm{valid}}_{r_{d}}}}$$

where $\sigma_{s,i}^{*2}(r_{d})$ is the target variance at the span *r*_d_ for the *i*th surrogate, and the average is taken over all spans *r*_d_ that are valid in all surrogate and original DVV plots.

DVV toolbox for Matlab and related documentation are available from Mandic [39] and are used for this work with little modification of DVV parameters.

### 3.2 Discussion on parameters

For a correct choice of embedding parameters, which might not be unique, the target variance, σ*^2^, gives information regarding one of the fundamental properties of a signal, i.e. its predictability. Two extreme cases correspond to a white noise (entirely unpredictable) and a deterministic signal (entirely predictable). It is important to determine the embedding dimension and time lag correctly, since in combination with the structured signal, similar delay vectors in terms of their Euclidian distance have similar targets [40]. The embedding dimension *m* determines how many previous time samples are used for examining the local predictability. We used and compared three different methods when adopting the embedding dimension and time lag:

*Method 1*. Differential Entropy Method, which determines the optimal embedding parameters of the signal using a differential entropy method proposed by Gautama *et al*. [40]. The optimal *m*, and time lag, *τ*, are simultaneously determined [25].

*Method 2*. Minimal Target Variance Method which determines optimal embedding dimension, *m* by running a number of DVV analyses for different values of *m* and choosing the one for which the minimal target variance, σ*^2^_min_, is the lowest while time lag is set to unity [28].

*Method 3*. Manual Setting of Parameters, where the *m* is set manually, for within the context of nonlinearity detection it is not considered critical [17]. Therefore, the embedding dimension was set to 3, after observation of DVV plots of available experiments and the time lag is set to unity for convenience.

The background theory on these three methods is provided in the electronic supplementary material, appendix S1.

The maximal span parameter, *n*_d_, determines the range of standardized distances to consider. It is the parameter controlling the span over which to perform the DVV analyses. Visual inspection of the convergence of DVV plot to unity should be used for setting this parameter, i.e. typically starting at value *n*_d_=2 and increasing it using unit steps until DVV plots converge to unity. We adopt *n*_d_=3 in all simulations in this paper when using this method. The number of standardized distances for which target variances are computed, *N*_tv_, has been set to 50. The number of reference DVs considered, *N*_*sub*_, is 200 for all simulations. Reducing the size of subset of DVs to which pairwise Euclidean distances are computed significantly speeds up DVV analysis. For each of the time series, we perform a set of DVV-based nonlinearity analyses for a range of parameter values using a set of *N*_*s*_=25 surrogates. Gautama *et al*. [28] have analysed the sensitivity of the proposed DVV method to parameter settings for four different time series, of which three were nonlinear. They found that the embedding dimension, *m*, and the maximal span, *n*_d_, were the only parameters with a notable effect with respect to nonlinearity detection. They also concluded that the effects were minor for reasonable parameter values, i.e. *m*∈[3,10] and *n*_d_≥1.

## 4. Simulated vibration problems for benchmarking delay vector variance

In order to establish benchmark values for the behaviour of mechanical systems using their dynamic responses to different excitation, an SDOF has been chosen as a reference system. The length of the time series analysed in every simulation is approximately 1000. The ‘RMSE’ indicates the RMSE of DVV plots (original versus surrogate data) and is quantified as the deviation from the bisector line of the DVV scatter plot. The differential equations describing the SDOF systems and its responses with different parameters and input forces considered are given in the electronic supplementary material, Appendix S2. DVV results of responses were compared considering two levels. The first level of DVV analysis represents the choice of embedding parameters for DVV analysis), while the second level refers to detection of the changes in observed system characteristics (electronic supplementary material, appendix S3).

A number of observations are made from the simulations considered for different theoretical models. Method 3, keeping *m*=3 and *τ*=1, shows the best consistency in interpretation of DVV method results when SDOF parameter changes. The degree of nonlinearity of different SDOF systems response was observed for variation of the following parameters: system mass, damping, driving frequency, natural frequency and input force magnitude.

**Figure 1.**
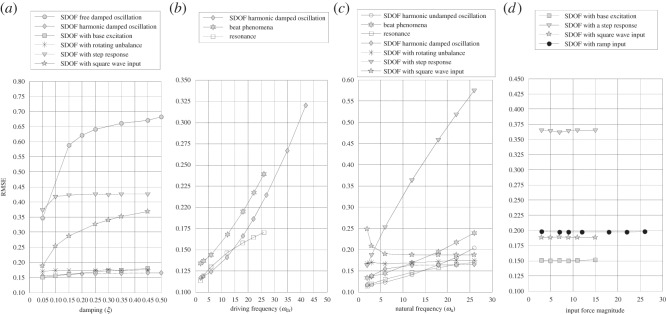
RMSE dependency on: (*a*) SDOF damping, (*b*) driving frequency, (*c*) natural frequency and (*d*) input force magnitude increase.

The results of analysis of undamped free oscillator and rotating imbalance shows that RMSE changes negligibly with changing system mass. The results of all underdamped systems (*ζ*<1) analysed are summarized in table 1 and figure 1*a*. The results show that when damping ratio increases RMSE also increases.

**Table 1.** RMSE obtained using third approach on damped SDOF system.

system	constants	damping ratio	RMSE third approach
SDOF damped oscillation $m\dot{x}+c\ddot{x}+kx=0$	natural frequency *ω*_n_=7	0.05	0.347
	initial displacement *x*_0_=3	0.20	0.621
	initial velocity *v*_0_=1	0.50	0.682
	time duration to test *t*_f_=30 s		
SDOF harmonic damped oscillation $m\ddot{x}+c\dot{x}+kx=F\cos\;(\omega t)$	driving frequency *ω*_dr_=3	0.05	0.155
	natural frequency *ω*_n_=3.5	0.20	0.162
	force magnitude per unit mass *f*_0_=6	0.50	0.166
	time duration to test *t*_f_=30 s		
SDOF base excitation $m\ddot{x}+c(\dot{x}-\dot{y})+k(x-y)=0$$y(t)=Y\sin(\omega_{b}t)$	base amplitude *y*_0_=3	0.05	0.151
	base excitation frequency *w*_b_=6	0.10	0.156
	natural frequency *ω*_n_=4	0.30	0.172
	time duration to test *t*_f_=10 s		
SDOF having a rotating unbalance for zero initial conditions	rotating mass *m*_o_=3	0.05	0.170
	SDOF mass *m*=7	0.10	0.174
	angular velocity of rot mass *ω*_r_=4	0.30	0.175
	natural frequency *ω*_n_=12		
	time duration to test *t*_f_=10 s		
	constant *e*=0.1		
SDOF having a step response	system mass *m*=1	0.05	0.373
	natural frequency *ω*_n_=12	0.10	0.418
	time duration to test *t*_f_=10 s	0.30	0.425
	force magnitude *F*_m_=5		
	initial time *t*_o_=2		
SDOF having a square wave input	system mass *m*=1	0.05	0.189
	natural frequency *ω*_n_=12	0.10	0.254
	time duration to test *t*_f_=10 s	0.30	0.341
	force magnitude *F*_m_=7		
	wave starts at *t*_o_=0		
	wave stops at *t*_o_=3		

The RMSE values show almost the same results for three SDOF systems when exposed to harmonic oscillation, to base excitation and for rotating imbalance, respectively. The curves asymptotically approach RMSE of 0.180 for higher values of damping. However, for three other cases: free damped oscillation, step response and square wave input, this is not the case and the values are much higher. These phenomena may arise from the fact that the systems reach equilibrium quite fast and as a consequence there are not sufficient data for analysis. For example, in the case of free vibrations, the response quickly becomes virtually zero. This occurs within 10 s, even for a damping coefficient as small as 0.05, and the RMSE obtained is the highest recorded for such condition. For overdamped (*ζ*>1) and critically damped (*ζ*=1) systems analysed, when damping ratio decreases RMSE increases. Here as well, the system reach equilibrium very fast; as a result DVV plots do not converge to unity as expected and deviation from bisector line of the DVV scatter plot is high.

Figure 1*b* shows that the RMSE increases with increasing driving frequency. For the case of resonance RMSE is lower, while for beat phenomena RMSE has higher values when compared with the case when natural frequency is not close or equal to driving frequency. Figure 1*c* shows the dependency of RMSE on increasing natural frequency. In all observed cases of SDOF RMSE increases with increased natural frequency, except in the case of a square pulse input, where it has steep decrease for low natural frequency (3–6 Hz) and remains almost constant for higher frequencies. The curve for the case of the step response gives visibly higher values of RMSE in comparison to the other cases observed. This is a result of system fast return to static equilibrium as was the case with some underdamped systems observed earlier. The increase in the base frequency for the SDOF system with base excitation results in almost constant value of RMSE. Figure 1*d* shows that the change in the input force magnitude does not produce the change in the RMSE. Note that the SDOF with the step response has the higher value of RMSE than the three other cases observed; this is, again, the consequence of a ‘flat’ (equilibrium) part of the system response.

Therefore, the linearity or nonlinearity of the system response (signal) could be linked with the type of the system in relative terms. Hence, if we have observed the system and have its response in its ‘original’ form, any kind of change to that system will produce the change in its response. By doing the DVV analysis of ‘original’ and ‘new’ signal and comparing their RMSE (deviation from bisector line), we could tell if some parameter influencing system response is changed. Following this conclusion, the series of experiments on the SDOF system is performed, the responses recorded and DVV analysed. The aim is to investigate to what extent response measuring devices can capture the change in system characteristics using the DVV technique on recorded measurements to quantify that change. Hence, the discussion and results on SDOF experiments are presented next.

## 5. Single degree of freedom car experiment

The vibration of a bilinear SDOF system exposed to known, dynamic input forces for a period of time was recorded in a laboratory environment. The dynamic response of the SDOF system was measured using a Polytec RSV-150 Remote Sensing Vibrometer and a MicroStrain G-Link Wireless Accelerometer (mounted on the SDOF system). The accelerometer is a traditional and reliable tool for monitoring structures adopted for laboratory and large-scale *in situ* measurements but the disadvantage is that they have to be attached to the structure at all times during monitoring, which is not always possible. The LDV is still a relatively new measurement tool but it allows for rapid, accurate, non-contact and long distance measurement of vibrating structures [18,19].

### 5.1 Details of experiment

**Figure 2.**
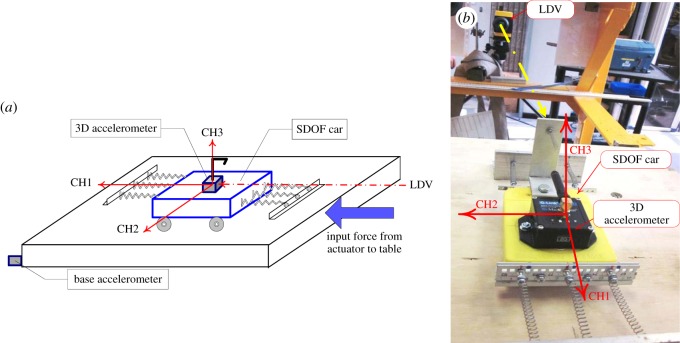
(*a*) Schematic diagram of SDOF car experiment and (*b*) experiment set-up.

The SDOF model was made of a mass cart connected to fixed supports on either side through calibrated springs (figure 2). The SDOF model was placed on a vibration bench and exposed to the external force.

The stiffness of the SDOF system is presented with the linear springs which are empirically calibrated prior to the experiment under tensile static loading. The equivalent stiffness of the combined all six springs for the first 10 experiments was *k*=0.378 N mm^−1^ (higher stiffness). For the second part of the experiment, two middle springs were removed and the system stiffness was reduced to *k*=0.249 N mm^−1^ (lower stiffness). In one of the experiments, the sudden change of stiffness was simulated by a sudden failure of the middle springs on either side during vibration measurements. For this experiment, only the middle springs were attached to rigid support with adhesive with a lower strength in order to simulate the spring failure/sudden stiffness change. The excitation force, the type of the surface beneath the wheels of the SDOF car and the stiffness of the SDOF system were varied. For all SDOF car experiments, the recorded responses are shown in the electronic supplementary material, appendix S4. The forced vibration response of the system was considered.

The DVV analysis results are compared at three different levels here. The first level considered is choice of DVV parameters using the three different methods for establishing embedding parameters; the second level is comparison of DVV results due to change in system characteristics (e.g. input force, surface roughness, stiffness change) and the third level is comparison of results obtained using two instruments (3D accelerometer and LDV).

**Figure 3.**
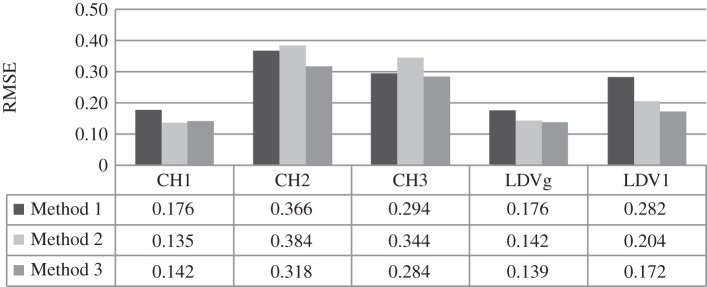
The example of RMSE obtained when using three methods for calculating embedding parameters.

Methods 1 and 2 can be difficult to implement and computationally intensive for a large number of data points, as was the case here. Segmentation of the responses can be effective in such circumstances. In this paper, a segmentation length of 9000 data was adequate. The analysis of system response to white noise shows that the parameters calculated using Methods 1 and 2 on segmented data applied on all data gave almost the same RMSE. This was not the case with responses on sine sweep and increasing frequency input. In general, Method 1 yields higher RMSE values than other approaches. This might be due to the segmentation of the signal which leads to different parameter choice for each section of the signal. Further, the parameter choice does not always fall within reasonable limits using this method [28]. When Methods 2 and 3 are applied on response data, RMSE values are close (or the same) in a majority of cases. Values of optimum *m*, obtained by using Method 2, were higher for most analysed examples. This is in agreement with the findings of Gautama *et al.* [28] that the effects with respect to nonlinearity detection are minor for reasonable parameters values, i.e. *m*∈[3,10] and *n*_d_≥1. However, when results of all three approaches are compared the trend is similar for RMSE values. Figure 3 shows the results of DVV analysis using all three approaches in determination of optimal parameters for an SDOF car connected with six springs, moving over a wooden surface excited by harmonic force. CH1, CH2 and CH3 represent 3D Accelerometer Cartesian direction measurements recorded by Channel 1 (direction of movement), Channel 2 (sidewise movement) and Channel 3 (vertical movement), while LDVg and LDV1 represent Laser acceleration input and Laser response, respectively. The movement along CH1 is prominent while small sidewise movement can still happen although there rails discourage the mass cart from doing so. The vertical movement is expected to a minimum. The complexity and duration of simulation indicates that Method 3 is easier to implement while obtaining accurate results. This approach was shown to be consistent in application on a large set of data. Therefore, only the results obtained by the third approach will be discussed.

### 5.2 Results

**Figure 4.**
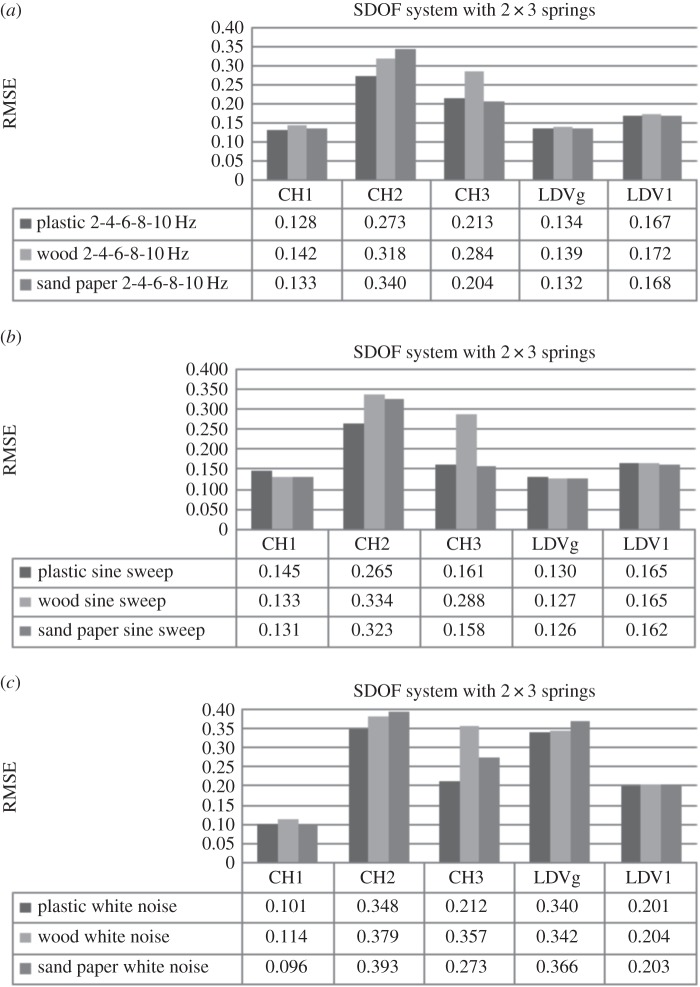
Effects of surface roughness on SDOF system (*k*=0.378 N mm^−1^) exposed to (*a*) the increasing frequency external force, (*b*) sine sweep and (*c*) white noise.

Three different surface types made of plastic, wood and sand paper were used for experiments. This section investigates the influence of these surface roughness types on the system response while excitation force and system stiffness remain constant. First the SDOF car moving over three different surfaces, exposed to one type of loading at the time, connected to the supports on each side by three springs with higher stiffness, is observed. Figure 4*a* shows RMSE obtained using DVV analysis on response of the system excited by the external force with increasing frequency for the three surfaces. The input frequency was discretely increased from 2 to 10 Hz with an increment of 2 Hz at a time.

RMSE values for the measurements in the principal direction of vibration, CH1 and LDV1, do not have a trend as surface roughness changes from good to poor. As observed earlier (figure 1), RMSE of modelled SDOF system exposed to free damped oscillations increases with the increase in damping. However, the results of the SDOF experiments are sometime insensitive to change in surface roughness which should result in variation in damping ratio of the system. Actually, analyses using both measurements give close values of RMSE for different surfaces, which could indicate that the friction between the wheels of the SDOF car and the surface was low enough to be neglected. RMSE of recorded responses by both instruments is consistent, i.e. being higher for sand paper and lower for wood surface. However, accelerometer measurements appear to be more sensitive to surface change since the difference in the degree of nonlinearity is higher than for LDV measurements. This is might be above all due to the sampling frequency of the accelerometer and the LDV, which were 128 and 830 Hz, respectively, and to a lesser extent due to possible amplification of errors during the differentiation process being applied on LDV velocity measurements when obtaining the acceleration. Further, the difference in surface roughness is better detected with the lateral car movement as friction between the car wheels and surfaces is more prominent. Hence, RMSE calculated for CH2 data (lateral direction to vibration) increases with the decrease in surface roughness and the values are about twice that for the main direction of vibration, i.e. the system response is less linear than in the main direction and therefore more sensitive to change in surface roughness. This is explained as there the two rails in the experiment providing a barrier against the mass cart from moving in the lateral direction.

A similar situation is observed when the excitation force is a sine sweep (figure 4*b*). The RMSE values obtained by DVV analysis of accelerometer data and LDV data in the direction of main vibration are very close for all surfaces tested. Here, the RMSE values for LDV are lower than in the case of harmonic loading and almost constant. The RMSE calculated on CH1 data decrease with road surface change from smooth to rough. RMSE values for the CH2 data are about twice higher than for CH1 data.

Figure 4*c* shows RMSE for experiments when the SDOF is exposed to white noise excitation. The RMSE values obtained for CH1 data are lower while for LDV are higher than for the two other types of loading. The results for the three types of surfaces are similar for both measuring devices in the main direction, while in lateral direction the increase with the decrease of the quality in surface and the values are almost four times greater than in the main direction.

**Figure 5.**
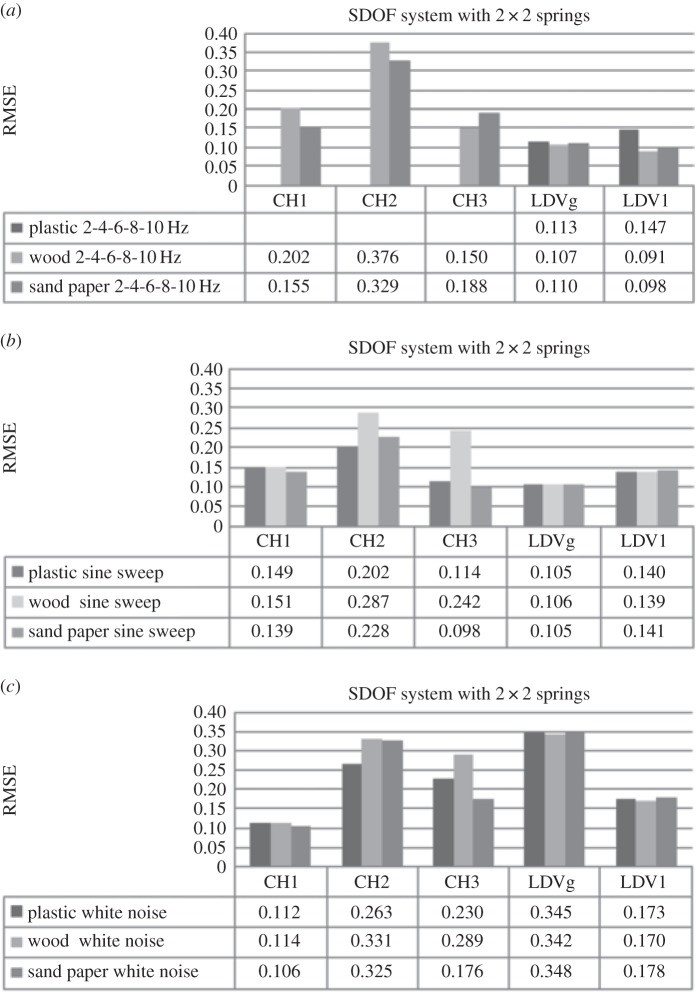
Effects of surface roughness on SDOF system (*k*=0.249 N mm^−1^) exposed to (*a*) increasing frequency, (*b*) sine sweep and (*c*) white noise.

Next, the set-up where two middle springs, one on each side of the car, are removed, i.e. reduced stiffness, is observed. Figure 5*a* shows the RMSE for the system excited by external force with increasing frequency. The results show that accelerometer measurements are more sensitive to stiffness change than LDV, i.e. RMSE is higher for CH1. The reason is use of the higher sampling frequency and differentiation of velocity measured with LDV in order to obtain acceleration, which tends to amplify any errors when adjacent data points are quite similar in size as was the case here. By closer inspection of the recorded data and their DVV plots (see the electronic supplementary material, appendix S4) for wood and sand paper surface, irregularities are observed. The response output changes the amplitude quite irregularly with high and low peaks, which is not the case in response with the plastic surface. The variation in amplitude leads to higher values of RMSE for CH1 data and lower for LDV data, than expected. This could be due to the change in stiffness combined with surface roughness or some external influence on the system. To address this issue, sine sweep and white noise excitation force responses were also investigated.

Figure 5*b* shows RMSE values for sine sweep loading. The RMSE values for principal direction of each instrument are close for all surfaces. In comparison with the system with higher stiffness, the RMSE values for CH1 data are slightly higher, while for CH2 and LDV data are notably lower, especially for wood surface. The RMSE values for the system exposed to white noise while moving on different surfaces are shown in figure 5*c*. The RMSE produced on recorded data by each instrument is very close in values for all surfaces, as previously observed. The results of these experiments, in comparison with the ones where the stiffness of the mechanical model was higher, give slightly higher RMSE values for CH1, while they are lower for LDV data. Again, this could be attributed to sampling frequency and to differentiation method being applied on the LDV velocity measurements.

Considering all of the RMSE values, it appears that the RMSE value for CH2 and LDV could be the indicator of the change in surface roughness (table 2). RMSE values for CH1 data are relatively insensitive to change in surface roughness. However, it must also be noted that in this experimental set-up, surface roughness differences were relatively hard to detect using both instruments. Consequently, for the dynamic responses along CH1, the surface roughness may be ignored and the results may be interpreted to track changes in the excitation response or changes in the system.

**Table 2.** Effects of surface roughness on RMSE calculated on CH2 (lateral movement direction) and LDV signals for SDOF system with higher and lower stiffness exposed to different loading.

excitation force			harmonic loading		sine sweep		white noise	
stiffness			higher	lower	higher	lower	higher	lower
surface type	plastic	CH2	0.273	—	0.265	0.202	0.348	0.263
		LDV	0.167	0.147	0.165	0.140	0.201	0.173
	wood	CH2	0.318	0.376	0.334	0.287	0.379	0.331
		LDV	0.172	0.091	0.165	0.139	0.204	0.170
	sand paper	CH2	0.340	0.329	0.323	0.228	0.393	0.325
		LDV	0.168	0.098	0.162	0.141	0.203	0.178

**Figure 6.**
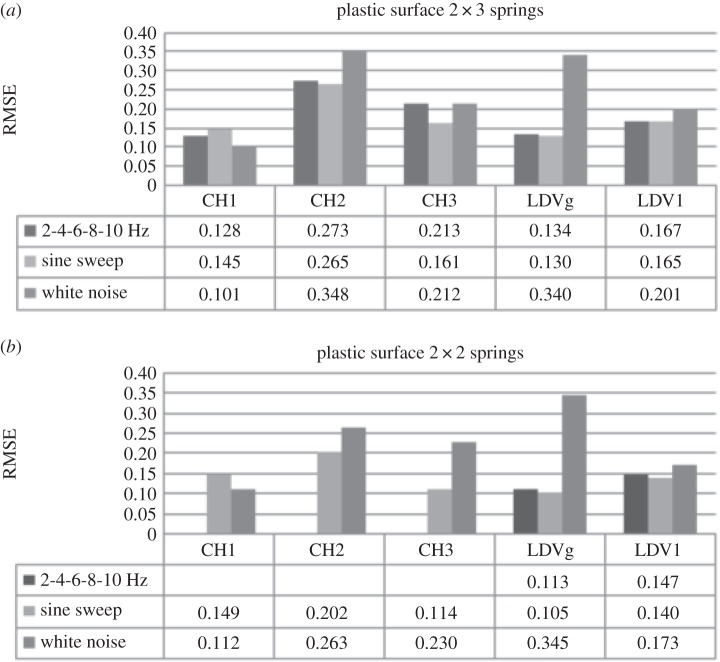
Effects of excitation force on the SDOF system on plastic surface with (*a*) *k*=0.378 N mm^−1^ and (*b*) *k*=0.249 N mm^−1^.

In order to investigate sensitivity of the DVV analysis on system response due to the change in excitation force, three different loads are used: (i) a harmonic load with discretely increasing frequency of 2, 4, 6, 8 and 10 Hz, (ii) a gradual sine sweep from 3 to 5 Hz, and (iii) the responses to white noise excitation. The results of DVV analysis on system response to these loads for a system moving over plastic surfaces for the model of the SDOF car with higher and lower stiffness are shown in figure 6.

The RMSE values for CH1 differ for each type of loading. The highest value is for sine sweep and the smallest for white noise. These values increase in the case of reduced stiffness. On the other hand, the RMSE values calculated on CH2 and LDV recorded data are the highest for white noise loading for the higher system stiffness. However, these values decrease for the lower system stiffness. RMSE for CH2 and LDV negligibly differs for the harmonic and sine sweep loading for the higher system stiffness. While calculated RMSE values on CH1 data increase, they decrease for CH2 and LDV. This decrease of RMSE for reduced stiffness in the case of CH2 and LDV data is greater than the increase for CH1 data.

**Figure 7.**
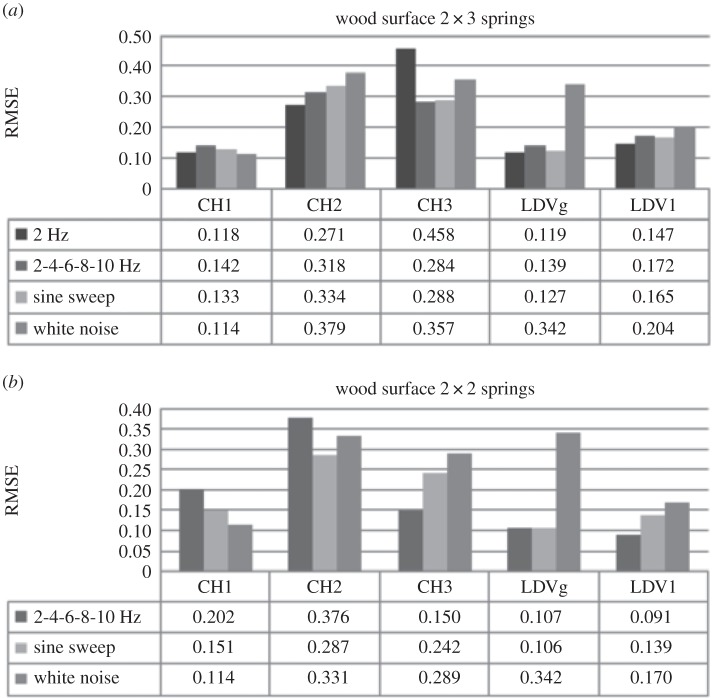
Effects of excitation force on the SDOF system on wood surface with (*a*) *k*=0.378 N mm^−1^ and (*b*) *k*=0.249 N mm^−1^.

Figure 7 shows the RMSE results for the SDOF system, with higher and lower stiffness, on wood surface exposed to three types of loads. The results for the system with higher stiffness (figure 7*a*) show that the effects of change in the input force nature are successfully recorded by LDV and CH2. Here, as for the plastic surface, the differences in RMSE for the harmonic and sine sweep loading are small. However, the difference in values increases with the change of surface from plastic to wood. The results for CH1 show that calculated RMSE for 2 Hz harmonic and white noise external force differ negligibly. Figure 7*b* demonstrates that the 3D accelerometer and LDV successfully record the change in external force when system stiffness is reduced. The values for CH1 decrease while for LDV they increase with frequency, sine sweep and white noise force, respectively.

**Figure 8.**
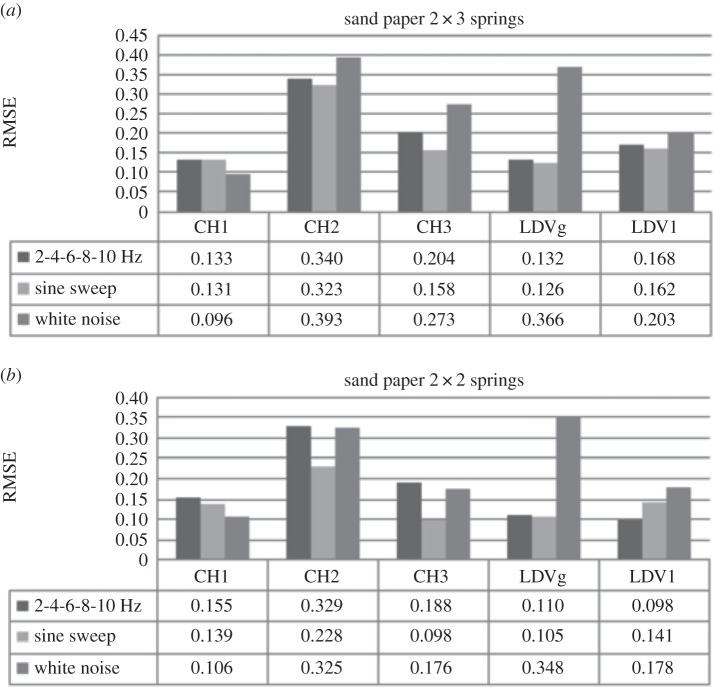
Effects of excitation force on the SDOF system on sand paper surface with (*a*) *k*=0.378 N mm^−1^ and (*b*) *k*=0.249 N mm^−1^.

Figure 8*a* shows the results of three experiments performed on the SDOF system with higher stiffness moving over a sand paper surface exposed to different forces. The RMSE calculated on CH1 data for harmonic and sine sweep force differ negligibly while for white noise loading the RMSE value is lower. The results for CH2 and LDV data also show that the RMSE for harmonic and sine sweep loading are close, but the values are greater than that calculated for CH1 data. On the other hand, the RMSE of the response to white noise is greater. Results of the experiment with same type of loading and surface as the one just discussed but with reduced SDOF stiffness are shown in figure 7*b*. The RMSE values for this system are higher for CH1 and lower for CH2 and LDV recorded data in comparison with the stiffer system. It is interesting to point out that the trend in RMSE values from CH1, CH2 and LDV in this case are the same for the model of the same stiffness moving on the wood surface. However, values of RMSE for CH1 are lower while for CH2 and LDV are higher than those for the wood surface.

Table 3 summarizes the degree of nonlinearity change due to excitation force of the signals recorded by CH1 and LDV for SDOF with higher and lower stiffness moving over different surface roughness.

**Table 3.** Effects of different excitation force on RMSE calculated on CH1 and LDV signals for SDOF system with higher and lower stiffness moving over different surface.

surface type			plastic		wood		sand paper	
stiffness			higher	lower	higher	lower	higher	lower
excitation force	harmonic loading	CH1	0.128	—	0.142	0.202	0.133	0.155
		LDV	0.167	0.147	0.172	0.091	0.168	0.098
	sine sweep	CH1	0.145	0.149	0.133	0.151	0.131	0.139
		LDV	0.165	0.140	0.165	0.139	0.162	0.141
	white noise	CH1	0.101	0.112	0.114	0.114	0.096	0.106
		LDV	0.201	0.173	0.204	0.170	0.203	0.178

Next, the possibility to detect sudden stiffness change was explored by introducing the failure of a spring at a certain instance in time during a given period of forced vibration. The forced vibration on the SDOF system was in the form of a white noise input. The stiffness of the system at the beginning of the experiment was 0.378 N mm^−1^. The first spring was detached after 13 s, which reduced system stiffness to *k*=0.303 N mm^−1^, and the second one after 38 s, reducing stiffness further to *k*=0.249 N mm^−1^.

**Figure 9.**
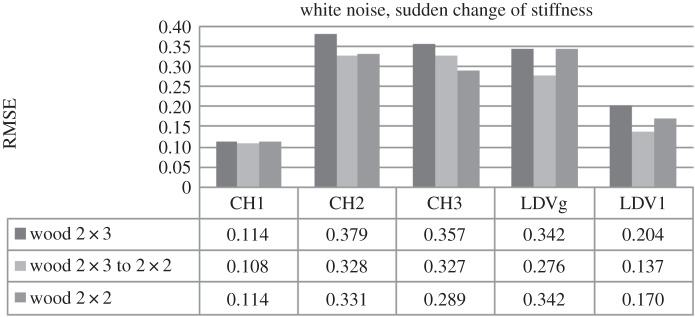
The comparison of SDOF systems with different stiffness.

Figure 9 compares RMSE values in relation to the SDOF car moving over a wooden surface, excited by white noise, for different stiffness values and including a sudden change in stiffness. The results for CH1 and LDV show the same pattern, i.e. the RMSE values for the system that suffered sudden change of stiffness are lower than for the systems with constant stiffness. The reason for this could be the fact that the system goes through change from a relatively linear system to a strongly bilinear system with some lateral effects for a certain period of time and then returns to a relatively linear system, averaged over time. The DVV method can be used to determine occurrence of sudden stiffness change but not the exact time when the change happens.

The experimental study on the mass cart SDOF system can be summarized through a number of observations. Only the forced vibration response of the system should be analysed. A close observation of the response data is required prior to a DVV analysis, as irregularities in the response signal can lead to the unexpectedly higher or lower values of RMSE. These irregularities occur due to sensitivity of measuring instruments to changes in experimental environment. Methods 1 and 2 have limitations when analysing large sets of data while determining embedding parameter and time lag. Method 3 shows the best efficiency and consistency in interpretation of DVV method results for the observed system. The RMSE values calculated using measurement data in the main direction of vibration obtained by 3D accelerometer and LDV are different in value and may have different trends. The recording parameters within 3D accelerometer and LDV were fixed and for this reason different results can be attributed to different nature of data collected (acceleration and displacement), but also the sensitivity of instruments to noise. The CH2 accelerometer data (lateral direction to vibration, horizontal plane) correspond to RMSE values multiple times higher than for the main direction of vibration, i.e. the system response is less linear than in the main direction. The DVV analysis of CH2 data shows that some changes could be detected observing vibrations in lateral direction. The difference in calculated RMSE on DVV analysed response data is hard to detect when surface roughness changes. The surrogate data deviation from bisector line in DVV analysis shows almost the same nonlinearity for different surfaces. Therefore, for this set up of the experiment, the friction between the wheels of the SDOF car and the surface is low enough to be ignored. However, for the harmonic and white noise load on the SDOF system with higher stiffness it seems that 3D accelerometer CH2 is able to pick up the change in surface roughness. Hence, the nonlinearity of data increases as surface roughness changes from good to poor. The switch in the stiffness of the observed system is recorded by both instruments. In the case of 3D accelerometer CH1, the difference in RMSE is small and increases, while for CH2 and LDV the difference in RMSE is greater and decreases with decrease in system stiffness. Therefore, the change in RMSE can be used to identify the change in system stiffness. The occurrence of sudden stiffness change in the SDOF system can be detected by the DVV method, but not the time of change. There is not enough information to assess if the DVV method can consistently identify the extent of the change of stiffness. The difference in the nature of the external force is recorded by both instruments. There are only few exceptions. For example, the difference in RMSE values is negligible for harmonic and sine sweep loading in the case of a plastic surface for response recorded by LDV regardless of the system stiffness. The same is observed for CH1 and LDV data in the case where the surface is sand paper, but only for higher stiffness of the studied model. In general, calculated RMSE values for CH1 are the highest for harmonic load with changing frequency and the lowest for white noise load, while for LDV the opposite is the case: the RMSE values are the highest for white noise load, and the lowest for harmonic load. The difference in loading is best represented in cases when the surface is wood or sand paper while the observed model has lower stiffness. Therefore, the combination of surface roughness and system stiffness could influence the success of the DVV model in recognition of the type of excitation force.

The detailed analysis of the DVV results on responses of the SDOF system showed that the change of some of the system characteristics are successfully identified by proposed signal processing technique. Therefore, the next challenge represents analysis of more complex system response using the DVV method. For that reason experiments are performed on a WTB (representing continuum) and responses using three different instruments are analysed using the DVV technique. Here, as before, the results of DVV analysis are compared on several levels, i.e. testing the sensitivity of the method on different excitation force, locations of measurements and instrumentation sensitivity.

## 6. Experiments on a wind turbine blade

### 6.1 Experimental set-up and methodology

**Figure 10.**
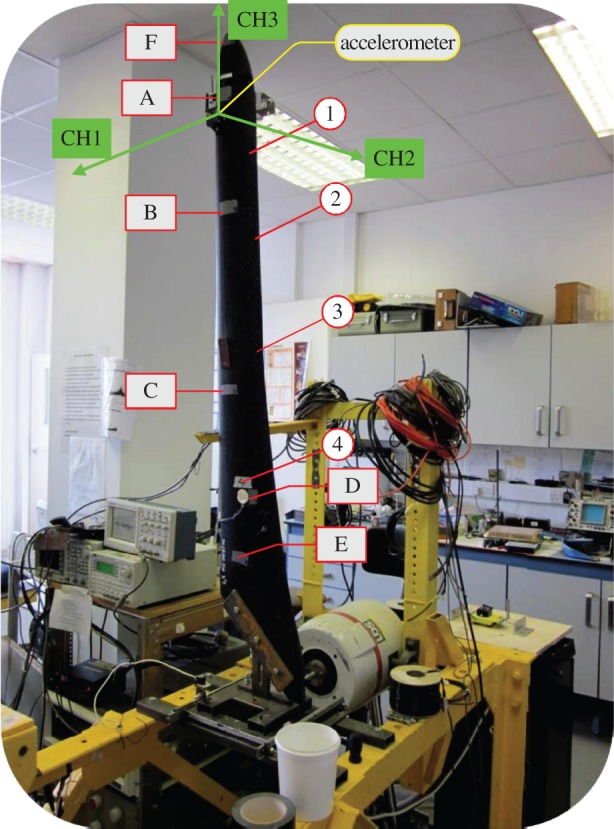
WTB experiment set-up. Letters A–F indicate positions used for focusing the vibrometer during set-up of LDV while numbers 1–4 mark locations of strain gauges.

A 1.4 m long WTB of mass 1.7 kg, made from a polypropylene/glass fibre composite, was used for the test. The instrumentation scheme is shown in figure 10. Base excitations were applied using a uniaxial LDS electrodynamic shaker to which the excitation signal was input via an amplifier. The dynamic response of a WTB was measured using a wireless MicroStrain G-Link Accelerometer and Polytec RSV-150 Remote Sensing Vibrometer. The G-Link, located at 235 mm from the blade tip, collected acceleration data in Cartesian coordinates. LDV focus points varied throughout experiments. For setting up LDV focusing points A–F are used. The dynamic strain was measured at four different locations for the WTB. The excitation forces included harmonic excitation, sine sweep and white noise. The harmonic excitations were discretely increased corresponding to individual frequencies 2.0, 2.5, 3.0, 3.5, 4.0, 4.2, 4.3, 4.4, 4.5, 4.6, 5.0, 5.5, 6.0, 6.5 and 7.0 Hz. The sine sweep tests were carried out by gradually varying the frequency from 3 to 5 Hz. The experiments were repeated four times, for each type of excitation force, while LDV target changed, focusing on accelerometer and points A, C and E. Point E is very close to the base of the turbine blade and thus this is not an appropriate measurement location for assessing the global dynamics of the blade.

A comprehensive set of results is reported in the electronic supplementary material, appendix S5, in relation to the measurements at this point as evidence of the inappropriateness of the location of measurement. The electronic supplementary material, appendix S5 also contains the measurements and DVV results of all other points measured. Previous experiments and modelling had established the natural frequency of the WTB to be 4.38 Hz. Only forced vibration responses are considered for DVV analysis. In this set-up, CH1 is the acceleration measured in the main direction of vibration, CH2 is the acceleration measured laterally to the main direction of vibration (horizontal plane), CH3 is the acceleration measured laterally to the main direction of vibration (vertical plane), LDVg is the Laser acceleration input, while LDV1 and LDV2 are the displacement and velocity recorded by LDV. Strain 1 is the strain gauge (SG) measurement at 415 mm (top SG); Strain 2 is the SG measurement at 835 mm; Strain 3 is the SG measurement at 1095 mm; and Strain 4 is the SG measurement at 1095 mm (bottom SG). The recorded data were in most cases too long to be analysed by Method 1. The attempt to find optimal embedding parameter using Method 1 was done by segmenting the response signal. The obtained embedding parameters give different values of RMSE of response signal for the segmented section as compared to the whole signal. The difference in RMSE values increases when embedding parameter is greater than 5 and time lag greater than 1. RMSE changes using Method 1 are representative of changes within the signal but cannot be used to compare two or more different responses of the system with consistent interpretation. Hence, Method 1 was observed to be time consuming and unreliable for the large sets of data. It is hard to observe the time-series structure in phase space as a whole, and therefore impossible to determine its clear minimum in order to find adequate parameters, *m* and *τ*, to represent the response signal, therefore this method is not adequate for the analysis [40]. A similar situation is created for Method 2, where the long set of response data takes too long to be processed and obtained values of embedding parameter are in most cases out of reasonable range *m*∈[3,10] [26]. When comparing the results obtained by analysis of one set of data by all three methods, the trend tends to be similar, i.e. the values of RMSE for the measured data in general retain their relative relationship. By closer inspection of the results obtained by the second and third methods, the calculated RMSE are similar or even identical when embedding parameter chosen is less than 10. It is adequate to use Method 3 for such a situation, as has been observed in the previous sections. The results of DVV analysis obtained when using Method 3 are discussed here while results of all three methods are included in the electronic supplementary material, appendix S5.

### 6.2 Results

**Figure 11.**
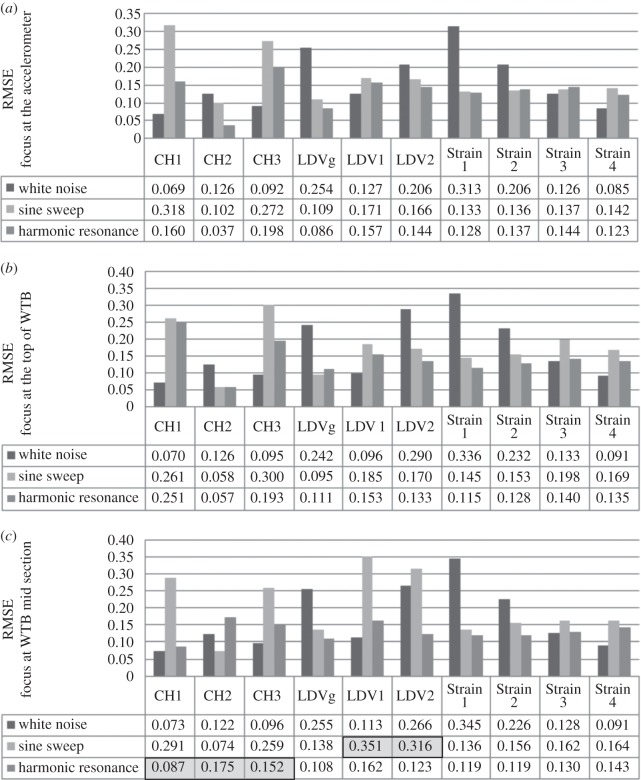
Comparison of WTB results for different forces when LDV focused on: (*a*) the accelerometer, (*b*) the top of the WTB and (*c*) the WTB mid-section.

The results of DVV analysis (in terms of calculated RMSE values) on response data for different loads are shown in figure 11*a*. In these experiments, the focus of the LDV is at the location of the accelerometer. The RMSE values for CH1, CH3 and LDV1 of the accelerometer show maximum values for sine sweep and minimum values for white noise. The values for LDV2 are the highest for white noise and the lowest for harmonic response.

Figure 11*b* shows the RMSE values on response data for different excitation forces when the target of the LDV is at the top of the WTB. The RMSE values in connection with CH1 and CH3 show the same trend as in previous experiments, with the maximum value observed for sine sweep and the minimum value observed for white noise. LDV1 and LDV2 results keep the same trend as in figure 11*a*. LDV1 shows more nonlinearity for sine sweep loading response than for the other two types of excitation. RMSE values for LDV2 show more nonlinearity of the response signal when the system is excited with white noise than with the other type of loading.

The RMSE values in connection with different excitation forces are presented in figure 11*c* where the LDV is focused at the midpoint of the WTB. Here, as in previous examples, the relative pattern of the response nonlinearity is retained. Still, there is visible discrepancy in RMSE of acceleration data when the system is exposed to harmonic force in comparison with previous calculations. This is due to the length of recorded data, i.e. total response is not recorded. The irregularity in LDV recorded data when the WTB is exposed to sine sweep is reflected in calculated RMSE values, resulting in much greater nonlinearity of the signal from what was observed in previous examples. The irregularities in recorded signal are referred to here as unexpected rear peaks within the signals, which are possible due to instrument sensitivity to the environmental vibration changes or disturbances. The results that are affected by this phenomenon are highlighted in the following figures and are not considered in the analysis.

**Figure 12.**
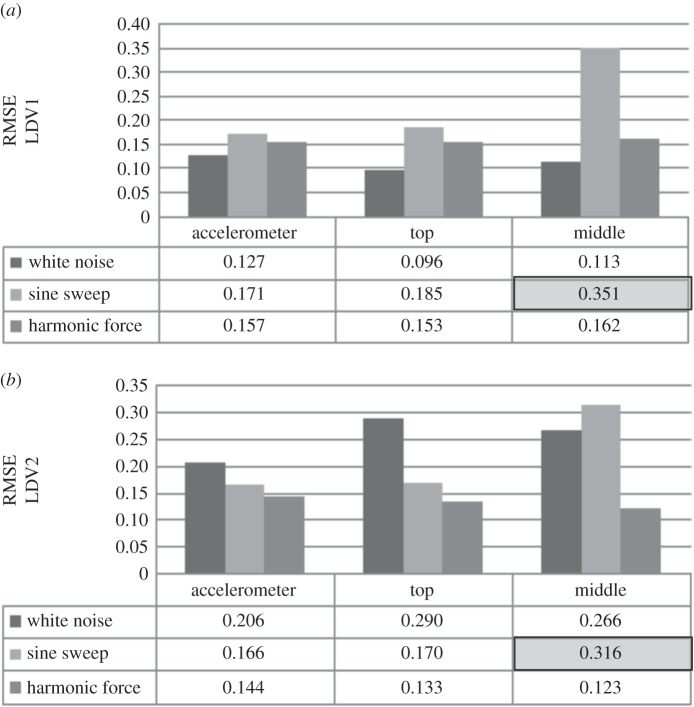
RMSE of DVV analysed (*a*) displacement and (*b*) velocity data at different LDV focus points.

In general, displacement data show high nonlinearity when the system is exposed to sine sweep and the smallest nonlinearity for white noise loading, regardless of the target focus of the LDV (figure 12*a*). The velocity shows the greatest nonlinearity when WTB is excited by white noise, and the smallest for harmonic loading (figure 12*b*). The acceleration data recorded are descriptors of responses to the given loading only at one location, i.e. the location of the accelerometer at the top of the WTB. Therefore, the range of RMSE for acceleration measured when the system is exposed to different forces as well as the range and trend of RMSE values of strain data along the WTB is investigated next.

**Figure 13.**
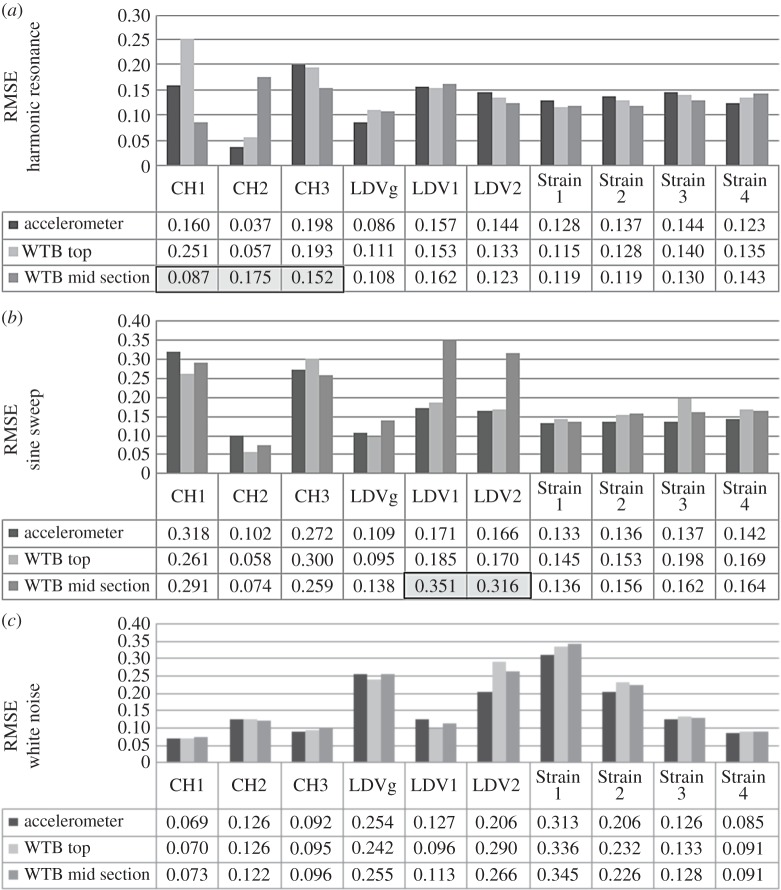
The effects of (*a*) harmonic force, (*b*) sine sweep force and (*c*) white noise force on linearity of response measurements.

Figure 13*a* shows RMSE values obtained as result of DVV analysis when the WTB was exposed to harmonic force. The range of RMSE for CH1 data is relatively wide, at accelerometer location and WTB top. On the other hand, the nonlinearity of response signal recorded by CH2 and CH3 are about the same for measurements at these two locations with CH3 representing a greater degree of nonlinearity. LDV measurements show increase in nonlinearity of displacement and decrease in nonlinearity of velocity signal from top to middle of WTB. The results for measured strain at four different locations show the tendency of the recorded signal to exhibit a greater nonlinearity closer to the base. RMSE values obtained from sine sweep response data are presented in figure 13*b*. The RMSE values are greater for this type of loading than for harmonic loading in the case of acceleration measurements. This would mean that the measured acceleration signal shows higher nonlinearity in the case of sine sweep loading. Further, LDV measurements show a greater nonlinearity for sine sweep loading than when the system is exposed to harmonic loading with an increasing trend. The RMSE values calculated on strain data are in general higher than for the previous loading observed. Figure 13*c* shows the results of DVV analysis of WTB recorded responses when the excitation force is a white noise. The results for acceleration data, CH1 and CH3, show less nonlinearity than for the other types of loadings observed. The DVV analysis of LDV measurements shows greater nonlinearity for velocity than for displacement data. The results show that measured displacement is more linear, while velocity is less linear when compared with the results obtained for harmonic and sine sweep loading measurements.

WTB responses to the external excitation were measured by different instruments using a 3D accelerometer, an LDV and strain gauges. DVV analysis results for measurements performed by each instrument are presented next.

**Figure 14.**
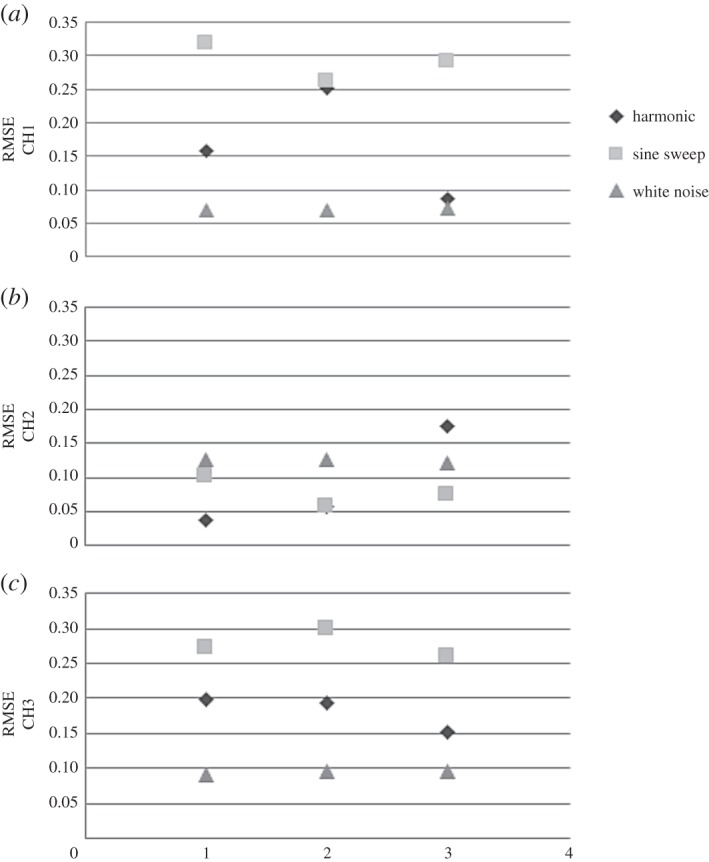
Comparison of DVV analysis results (RMSE) for 3D accelerometer measurements.

The 3D accelerometer recorded the acceleration in three different directions (CH1, CH2 and CH3) at the top (free end) of the WTB. Figure 14 shows the DVV results on data recorded by the three channels. The horizontal axis represents the different individual tests carried out. The results summarize all three types of loading applied. The data recorded by CH1 and CH3 show the highest nonlinearity of the signal when WTB is exposed to sine sweep and the lowest when exposed to white noise. Sine sweep loading corresponds to less nonlinearity than white noise for acceleration measured by CH2. The LDV recorded measurements of displacement, LDV1, and velocity, LDV2, at four different locations for WTB exposed to three different excitation forces.

**Figure 15.**
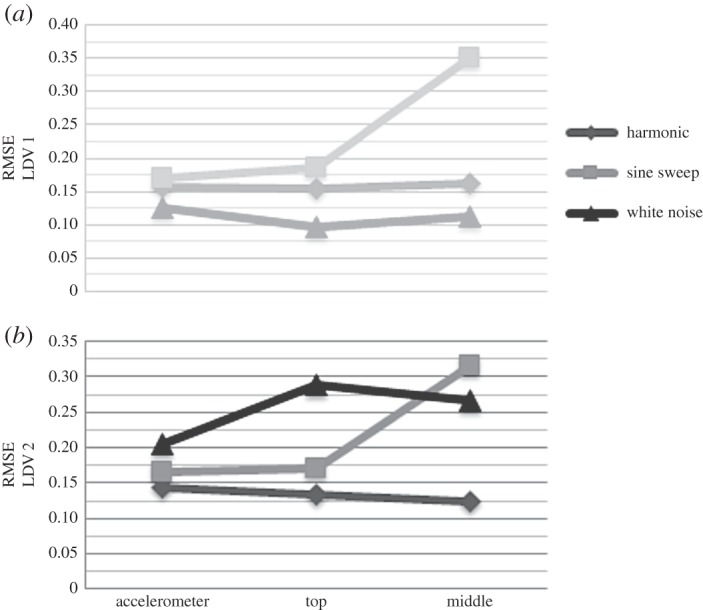
Comparison of DVV analysis results (RMSE) for LDV measurements.

Figure 15 shows the results of DVV analysis of the LDV data. The displacement measurements show the highest nonlinearity for sine sweep load, and the lowest for white noise. In the case of velocity, white noise corresponds to the highest nonlinearity and harmonic loading to the lowest nonlinearity. The nonlinearity of the displacement data slightly increases, while the nonlinearity of velocity data slightly decreases in the case when excitation is harmonic from free to fixed end measurements.

**Figure 16.**
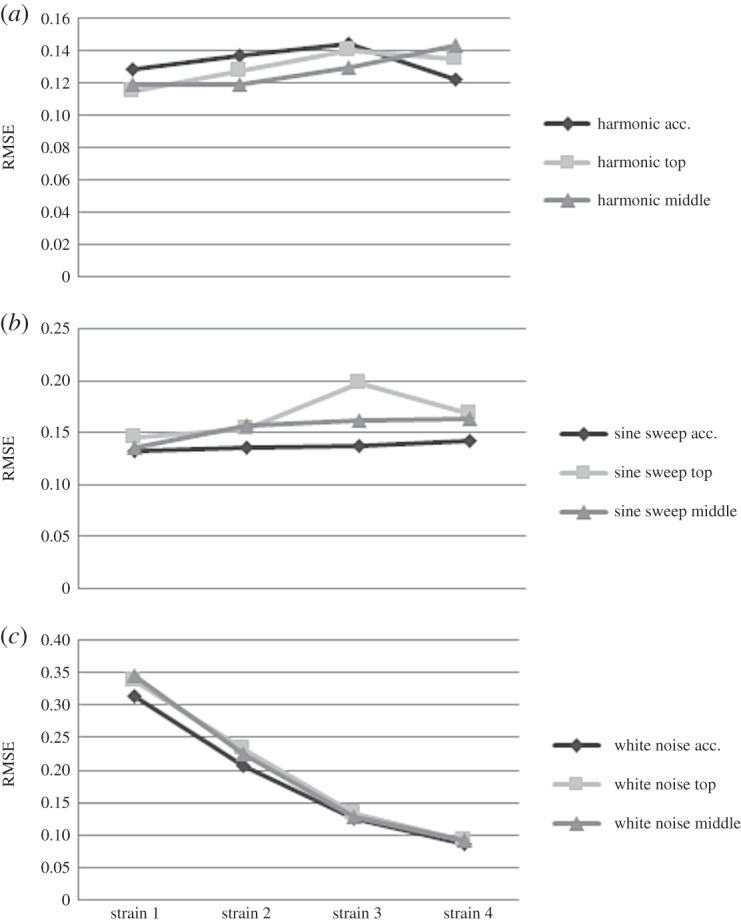
Comparison of DVV analysis results (RMSE) for strain gauges measurements.

Figure 16 shows RMSE results of the DVV analysis of SG recorded data at four different locations for different loadings. The experiments were repeated four times for each load. The nonlinearity of the strain data varies very little at observed points between experiments for all loads. The exception is measurement, Strain 3 for sine sweep, where the RMSE values are high for irregularities in obtaining some of the surrogates. In the case of white noise excitation, the nonlinearity of measurements decreases from WTB top to its base.

The experiments on WTB can be summarized through a number of observations. Methods 1 and 2 for determining embedding parameter and time lag have limitations when analysing large sets of the forced vibration data considered. Method 3 shows the best efficiency and consistency in the interpretation of DVV method results for the observed system. The method is sensitive to rapid/unexpected change in the recorded signal. The acceleration data recorded by CH1 and CH3 show the highest nonlinearity of the signal when WTB is exposed to sine sweep and the lowest nonlinearity when WTB is exposed to white noise. Sine sweep loading produces less nonlinearity than white noise for acceleration measured by CH2. The sine sweep loading causes the greatest nonlinearity in acceleration measurements in comparison with harmonic and white noise loading. The displacement measurements show the greatest nonlinearity for sine sweep and the lowest nonlinearity for white noise loading, regardless of the LDV focus point. The velocity measurements show the highest nonlinearity for white noise and the lowest for harmonic loading, regardless of the LDV focus point. The nonlinearity of the strain data varies very little at observed points between experiments in all load cases. This shows the stability of the instruments’ measurements. In the case of white noise, the nonlinearity of measurements decreases from WTB top to its base. From the results, range of RMSE, it is possible to detect difference in the external force.

## 7. Conclusion

This paper has investigated the estimation, assessment and interpretation of signal nonlinearities of mechanical dynamical systems from output-only conditions from a theoretical and experimental point of view and has employed the DVV method in this regard. A comprehensive set of theoretical dynamical systems and experiments on an SDOF system and a WTB have been carried out considering a wide variation of excitation forces, including investigation into the measurement variables and measurement devices. The practical implementation of the method for assessment of signal nonlinearities and potential relationships with system nonlinearities and the associated limitations has been discussed. The DVV method has been applied to analyse the response signals of one theoretical and two experimental models. The first goal was to establish the best method of choosing the embedding parameters for DVV analysis. The methods considered were differential entropy method, the minimal target variance method and manually setting embedding dimensions. It has been found that manually setting embedding parameter, for the observed set of data *m*=3 and time lag *τ*=1, gives reasonable and interpretable results in all observed cases. The DVV method is sensitive to length of available data, as well as to existence of any rapid (unexpected) change in recorded data. In the examples where the observed system reaches equilibrium fast there is not a sufficient number of data for DVV analysis. Outcome of DVV analysis on response signal is represented by a single number, RMSE value, which quantifies deviation of the DVV scatter plot from the bisector line. The DVV analysis of the SDOF theoretical model exposed to different types of oscillations has shown that the RMSE values are sensitive to some system parameters and insensitive to others. The former group includes damping ratio, driving frequency and natural frequency, while the latter group includes mass, base frequency and input force magnitude. The SDOF car experiments show that the DVV method can be used in detection of change in the system stiffness as well as change in the nature of excitation force. The change of the system stiffness is successfully recorded by 3D accelerometer and LDV. The occurrence of sudden stiffness change in SDOF system can be detected by the DVV method, but not the exact time or necessarily the extent of change. Further, three different instruments, 3D accelerometer, LDV and strain gauges have been used to monitor responses of the WTB system to vibration. All three instruments successfully record the changes in loading, but with different sensitivities. For example, 3D accelerometer data recorded by CH1 and CH3 show the highest nonlinearity of the signal when WTB is exposed to sine sweep and the lowest nonlinearity when exposed to white noise. On the other hand, the sine sweep loading corresponds to less nonlinearity than white noise for acceleration measured by CH2. The displacement shows the greatest nonlinearity for sine sweep and the lowest nonlinearity for white noise loading, while the velocity exhibits the highest nonlinearity for white noise and the lowest for harmonic loading, regardless of the LDV focus point. For the same load, the nonlinearity of the strain data varies very little at observed points; this indicates the stability of strain gauge measurements. The application of the DVV method on recorded responses of the observed structural system can be useful for Level 1 damage detection [41] in the context of continuous SHM.

## Supplementary Material

APPENDIX 1

## Supplementary Material

APPENDIX 2

## Supplementary Material

APPENDIX 3

## Supplementary Material

APPENDIX 4

## Supplementary Material

APPENDIX 5
