# Supplementary material for: A comprehensive study of the delay vector variance method for quantification of nonlinearity in dynamical systems
Source: R Soc Open Sci. 2016 Jan 6;3(1):150493. doi: 10.1098/rsos.150493 (PMC4736930; doi:10.1098/rsos.150493)
Supplement: APPENDIX 1 [file rsos150493supp1.pdf]

APPENDIX 1

Delay Vector Variance Method and Choice of Embedding Parameter

Table of Contents

APPENDIX 1 1

Delay Vector Variance and Choice of Embedded Parameter........ 1

List of Figures........ ii

A1.1 Delay Vector Variance 1

A1.2 Discussion on Parameters........ 7

A1.2.1 Method 1 – Differential Entropy Method........ 7

A1.2.2 Method 2 – Minimal Target Variance Method........ 9

A1.2.3 Method 3 – Manual Setting of Parameters 10

References 11

List of Figures

Figure A1. 1. DVV plots of SDOF system response a) linear and b) nonlinear / weakly linear signal. 5

Figure A1. 2. DVV scatter plots of SDOF system response a) linear and b) nonlinear / weakly nonlinear signal. 6

Figure A1. 3. Plot of the Entropy Ratio (ER) for harmonically excited SDOF system response signal. 8

Figure A1. 4. Finding the optimal embedding parameter, m: a) DVV plots obtained for m = 2 to 25 and b) Target variance σ^{*2} for response of a SDOF undamped system under harmonic excitation as a function of embedding dimension, m. 10

A1.1 Delay Vector Variance

The DVV method is a method for detecting the nonlinearity of the time series, which examines the predictability of time series in phase space at different scales, using the method of time delay embedding for representing a time series. The detailed description and testing of the method is presented in Gautama et al. [1-3] and Mandic et al. [4]. It has become fundamental tool for nonlinear time series analysis in many different research fields, such as geophysics and physiology, and can be used with any nonlinear statistic that characterises a time series with single number [2, 5-7].

The DVV method, as name suggests, is based on time delay embedding representation of a time series $x(n),\ n = 1, 2, \ldots, N$. For a given embedding dimension m, the Delay Vectors (DVs) are denoted as $\mathbf{x}(k) = [x_{k-\tau m}, \ldots, x_{k-\tau}]^T$, a vector containing m consecutive time samples and τ denotes time lag (delay). Every DV $\mathbf{x}(k)$ has a corresponding target, namely the following sample, x_k.

The surrogate data method can be used for assessing nonlinearity present in a time series [8]. A surrogate time series is generated as a realisation of null hypothesis of linearity where the 'test statistic' is computed for original time series and is compared to those computed for all generated surrogates, i.e. linearised versions of these data [8]. The null hypothesis in our case is that the original time series is linear. A time series is nonlinear if the test statistic for the original data is not drawn from the same distribution as the test statistics for the surrogates. When the test statistic computed for the original data set is significantly different from that computed for the surrogates, the null hypothesis is rejected, and the original time series is hypothesized to be nonlinear [1]. A key issue in surrogate data testing is the definition of an appropriate null hypothesis [5]. A simple null hypothesis verifies that the data is generated by a specific and known (linear) process, e.g. data are drawn from a Gaussian distribution with zero mean and unit variance. On the other hand, a composite null hypothesis, adopted here, asserts that the unknown underlying process is a member of a certain family of processes, e.g. data are drawn from a Gaussian distribution. Hence, surrogates are constrained to produce autocorrelation functions identical to those of the original time series, e.g. by phase randomising the frequency spectrum of original time series. Schreiber and Schmitz [9] have proposed a fixed point

iteration scheme, i.e. iterative Amplitude Adjusted Fourier Transform (iAAFT) method, which produces surrogates with identical signal distributions and approximately identical amplitude spectra as the original series, or vice versa. For every original time series, the surrogates are generated using the iAAFT method. By using iAAFT method instead of an Amplitude Adjusted Fourier Transform (AAFT) method the possibility of false rejections of null hypothesis is avoided and computational efficiency is achieved. Details of these methods are well explained by Mandic et al. [4]. Schreiber and Schmitz [5] show that algorithms converge after finite numbers of steps. In simulations performed by Gautama et al. [2] was typically 50 iterations for a time series of 1000 samples, while for the example surrogate for the Lorenz series the method was shown to converge after 25 iterations. We have used iAAFT method for generating surrogate time series, since it has been observed that it gives superior results in comparison with other methods [2, 5, 10].

Nonlinearity is often assessed as the absence of linearity and in statistical context, a null hypothesis is asserted that the time series is linear, and it is rejected if the time series does not conform to the properties associated with a linear signal. If the metric of the original time series is significantly different from that of surrogates, the null hypothesis is rejected and the original time series is hypothesized to be nonlinear [3]. For every original time series, we generate N_s = 25 surrogates for the nonlinearity tests [11, 12]. The test statistics for the original, t_0, and for the surrogates, $t_{s,i}$ $(i = 1, \dots, N_s)$ are computed and the series of $\{t_{0,}\ t_{s,i}\}$ is sorted in increasing order, after which the position index / rank r of t_0 is determined. Gautama et al. [2], for every original time series, used N_s = 99 surrogates to perform nonlinearity tests where a right-tailed test (DVV) is rejected if rank r of the original time series exceeds 90, left-tailed test is rejected if it is smaller or equal to 10 and a two-tailed test is rejected if rank r is greater than 95, or less or equal to 5. For every test statistic, it is important to verify the assumptions on which they are based or the properties they are examining, since these are important issues in the interpretation of analysis results [2].

The DVV method is based on time delay embedding representation of a time series $x(n),\ n = 1, 2, \dots, N$. For a given embedding dimension m, the Delay Vectors (DVs) are denoted as $x(k) = [x_{k-\tau m}, \dots, x_{k-\tau}]^T$, a vector containing m consecutive time samples. Every DV $x(k)$ has corresponding target, namely the following sample, $\mathrm{x_k}$. A set Ω_k is generated by grouping those DVs that are within a certain distance to

$x(k)$, which is varied in a manner standardised with respect to the distribution of pairwise distance between DVs. In this way, the threshold scales automatically with the embedding dimension m, as well as with dynamical range of the time series at hand, and thus the complete range of pairwise distances is examined. The proposed DVV method, for given embedding parameter m, can be summarised by an algorithm [2, 13] as

1) Reconstruct the phase-space and obtain the set of delay vectors (DVs) in phase space

$$x(k) = [x_{k-\tau m}, \dots, x_{k-\tau}]^T, \quad k = 1, \dots, N-m+1 \tag{A1.1}$$

where N denotes the length of time series and τ denotes time lag (delay).

2) Compute pairwise Euclidian distances between DVs

$$d(i,j) = \|x(i) - x(j)\|, \quad (i \neq j) \tag{A1.2}$$

3) Compute the mean μ_d and standard deviation σ_d over all pairwise Euclidian distances between DVs, a pragmatic approach to determine the scaling region

$$\mu_d = mean(d(i,j))_{ij} \tag{A1.3}$$

$$\sigma_d = std(d(i,j))_{ij} \tag{A1.4}$$

Since the surrogate time series have signal distribution identical to that of the original, the distributions of pairwise distances, and thus, the mean and standard deviation, will be similar. This distribution is approximately Gaussian for high embedding dimensions.

4) The sets $\Omega_k(r_d)$ are generated by grouping those DVs that are within a certain Euclidean distance to $x(k)$ so that

$$\Omega_k(r_d) = \{x(i) \mid \|x(k) - x(i)\| \leq r_d\} \tag{A1.5}$$

i.e. sets that consist of all DVs that lie closer to $x(k)$ than the certain distance r_d calculated as

$$r_d(n) = \mu_d - n_d\sigma_d + (n-1)\frac{2n_d\sigma_d}{N_{tv}-1}; \quad n = 1; \dots; N_{tv} \tag{A1.6}$$

taken from the interval $[max\{0, \mu_d - n_d\sigma_d\}; \mu_d + n_d\sigma_d]$, uniformly spaced, where n_d is a parameter controlling the span over which to perform the DVV analysis, usually set to be 3 and N_{tv} (number of target variance) indicates how finely the standardised distance is uniformly spaced.

5) For a given embedding dimension *m*, the main target variance, a measure of unpredictability, σ^{*2} is calculated over all sets $\Omega_k(r_d)$. Namely for every set $\Omega_k(r_d)$, the variance of the corresponding targets $\sigma_k^2(r_d)$ is computed. The average over all sets $\Omega_k(r_d)$ normalised by the variance of the time series, σ_x^2, yields the measure of unpredictability, the target variance $\sigma^{*2}(r_d)$ as

$$\sigma^{*2}(r_d) = \frac{(1/N)\sum_{k=1}^{N}\sigma_k^2(r_d)}{\sigma_x^2} \tag{A1.7}$$

Considering a variance measurement valid, too few points for computing a sample variance yields unreliable estimates of the true variance. Jianjun et al. [13] suggest that the set of $\Omega_k(r_d)$ should contain at least $N_0 = 30$ DVs. A sample of 30 data points for estimating mean or variance is a general rule-of-thumb and in this paper we only consider a variance measurement to be valid, if the set $\Omega_k(r_d)$ contains at least 30 DVs. If two DVs of a predictable signal are close to one another in terms of their Euclidean distance, they should have similar targets, i.e. the smaller the Euclidian distance between them, the more similar targets they have. Hence, the presence of strong deterministic component within a signal will result in the smaller target variances for small spans r_d. The minimal target variance $\sigma_{min}^{*2} = min_{r_d}[\sigma^{*2}(r_d)]$ represents the amount of noise present within the time series (the prevalence of the stochastic component) and has an upper bound of unity. The reason for this lies in the fact that all DVs belong to the same set of $\Omega_k(r_d)$ when r_d is sufficiently large.

Therefore the variance of the corresponding target of those DVs will be almost equal to that of the original time series [2]. As a result of the standardisation of the distance axes the resulting DVV plots are straightforward to interpret.

6) The resulting DVV plots are plotted with the standardised distance r_d on horizontal axis and normalised variance σ^{*2} on vertical axis. At the extreme right, DVV plots smoothly converge to unity, because for maximum spans, all DVs belong to the same set, and the variance of the targets is equal to the variance of the time series. If this is not the case, the span parameter n_d should be increased [3]. If the surrogate time series yield DVV plots similar to that of original time series, it indicates that time series is likely to be linear and vice versa. The example of a DVV plot is illustrated in Figure A1.1.

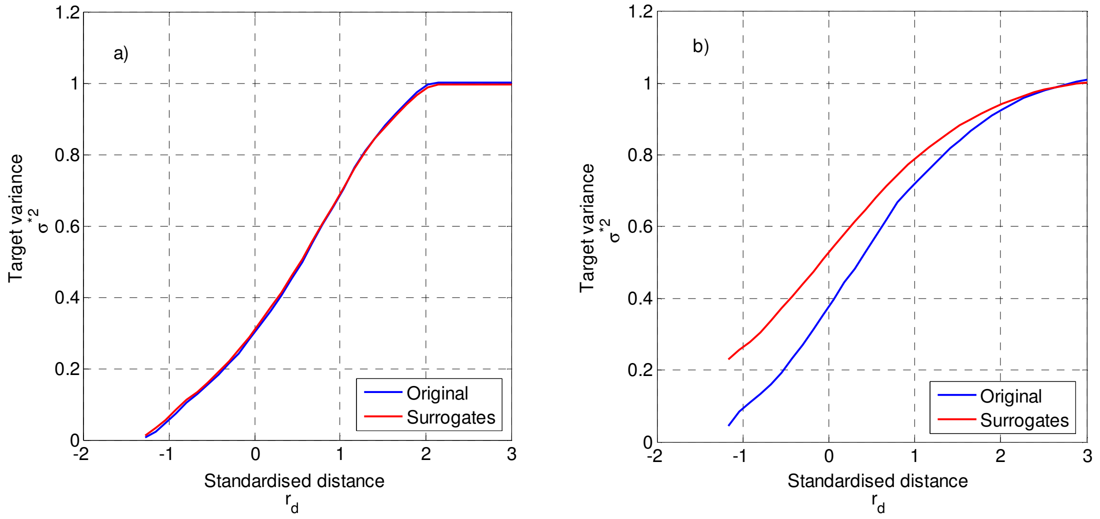

Figure A1. 1. DVV plots of SDOF system response a) linear and b) nonlinear / weakly linear signal.

7) Performing DVV analysis on the original and number of surrogate time series. DVV scatter diagram can characterise the linear or nonlinear nature of time series using the optimal embedding dimension of the original time series. If the surrogate time series yield DVV plots similar to the original time series (the DVV scatter diagram coincides with bisector line) then the original time series is likely to be

linear. The deviation from the bisector line is an indicator of non-linearity of the original time series [2]. The example of DVV scatter plots is given in Figure A1.2.

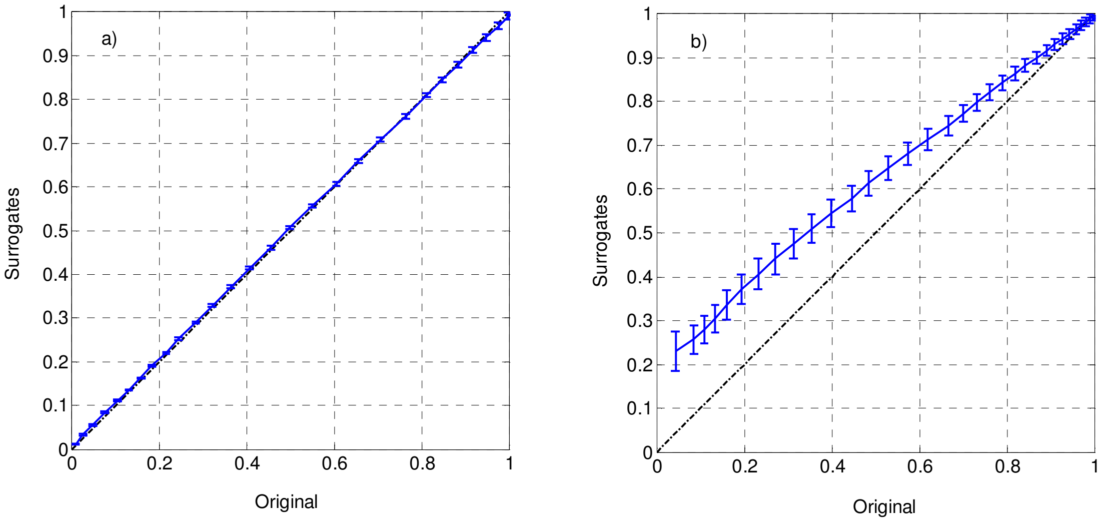

Figure A1. 2. DVV scatter plots of SDOF system response a) linear and b) nonlinear / weakly nonlinear signal.

The deviation from the bisector line can be quantified by the root mean squared error (RMSE) between the σ^{*2}'s of the original time series and the σ^{*2}'s averaged over the DVV plots of the surrogate time series [1]. A single test statistic t^{DVV} is calculated as

$$t^{DVV} = \sqrt{\langle \left(\sigma^{*2}(r_d) - \frac{\sum_{i=1}^{N_s} \sigma_{s,i}^{*2}(r_d)}{N_s} \right)^2 \rangle_{valid_{r_d}}} \qquad \text{(A1.8)}$$

where $\sigma_{s,i}^{*2}(r_d)$ is the target variance at the span r_d for the ith surrogate, and the average is taken over all spans r_d that are valid in all surrogate and original DVV plots.

A1.2 Discussion on Parameters

For a correct choice of embedding parameters, which might not be unique, the target variance, σ^{*2}, gives information regarding one of the fundamental properties of a signal, i.e. its predictability. Two extreme cases correspond to a white noise (entirely unpredictable) and a deterministic signal (entirely predictable). It is important to determine the embedding dimension and time lag correctly, since in combination with the structured signal, similar delay vectors in terms of their Euclidian distance have similar targets [14]. The embedding dimension m determines how many previous time samples are used for examining the local predictability. It is important to choose m sufficiently large, such that the m-dimensional phase space enables for a proper representation of the dynamic system. We used and compared three different methods when adopting the embedding dimension and time lag.

A1.2.1 Method 1 – Differential Entropy Method

Method 1 determines the optimal embedding parameters of the signal using a differential entropy method proposed by Gautama *et al.* [14]. The optimal m, and time lag, τ, are simultaneously determined based on estimates of the differential entropy ratio of the phase space representation of a sampled time signal and an ensemble of its surrogates. The entropy ratio method first uses the Kozachenko-Leonenko (K-L) estimate of the differential entropy [15] as

$$H(x) = \sum_{j=1}^{N} ln\left(Nd_j\right) + ln(2) + C_E \qquad \text{(A1.9)}$$

where N is the number of samples in the data set, d_j is Euclidean distance of jth delay vector to its nearest neighbour, and C_E (≈ 0.5772) is Euler constant. To determine the optimal embedding parameters the ratio between K-L estimates for the time delay embedded versions of the original time series, x, and its surrogates $x_{s,i}$, $i = 1,\ldots N_s$ needs to be minimised using

$$I(m,\tau) = \frac{H(x,m,\tau)}{\langle H\left(x_{s,i},m,\tau\right)\rangle_i} \qquad \text{(A1.10)}$$

where $\langle - \rangle_i$ denotes the average over i.

The Entropy Ratio (ER) is calculated using

$$R_{ent}(m,\tau) = I(m,\tau)\left(1 + \frac{m\, lnN_{sub}}{N_{sub}}\right) \tag{A1.11}$$

N_{sub} is the number of delay vectors, which is kept constant for all values of m and τ under consideration. If the temporal span of $(m \cdot \tau)$ is too small, the signal variation within the delay vector is mostly governed by noise and either m or τ should be increased. The set of optimal parameters, $\{m_{opt}, \tau_{opt}\}$, yields a phase space representation which best reflects the dynamics of the underlying signal production system and it is expected that this representation has a minimal differential entropy. The minimum of the plot of the entropy ratio yields the optimal set of embedding parameters. In order to determine the optimum embedding parameters in all simulations $N_s = 5$ surrogates were generated using iAAFT method and the entropy ratios were evaluated for m = 2, 3, …, 10 and τ = 1, 2, …, 10 [14]. Increasing the number of surrogates does not affect the results. The proposed method is illustrated in Figure A1.3 showing entropy ratio $R_{ent}(m,\tau)$. The minimum of the plot indicated with a large circle gives the optimum embedding parameters for the case shown $m_{opt} = 3$; and $\tau_{opt} = 1$.

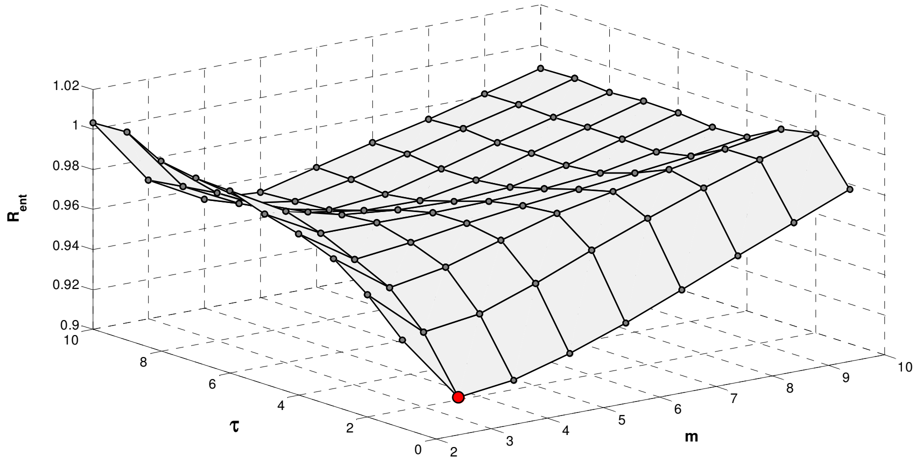

Figure A1. 3. Plot of the Entropy Ratio (ER) for harmonically excited SDOF system response signal.

The ER criterion requires a time series to display clear structure in phase space; i.e. for signals with no clear structure, the method will not generate clear minimum, and a different approach needs to be adopted [14]. In practice, it is common to have fixed time lag (sampling rate) and to adjust the embedding dimension (length of filter) accordingly [7, 16].

A1.2.2 Method 2 – Minimal Target Variance Method

Method 2 determines the optimal embedding dimension by running a number of DVV analyses for different values of m, and choosing the one for which the minimal target variance, σ^{*2}_{min}, is the lowest, i.e which yields the best predictability. In this work we performed this analysis for embedding dimensions ranging from 2 to 25 based on Gautama et al. [1]. The time lag, τ, for convenience, is set to unity in all simulations. This choice of τ is conservative in the context of nonlinearity detection. Assuming the embedding dimension is sufficiently high, a linear time series can be accurately represented using $\tau = 1$, while this is not the case for a nonlinear signal, for which time lag plays an important role in its characterisation. Hence, if the null hypothesis of linearity is rejected, one can assume that the time series is nonlinear. Since the linear part was accurately described for time lag equal to unity, the rejection can be attributed to the nonlinear part of the signal. On the other hand, if the null hypothesis is found to hold, the signal is genuinely linear or the phase space is poorly reconstructed using $\tau = 1$, i.e. the signal is actually nonlinear. The example of the method described is shown in Figure A1.4. The dashed line indicates the minimal target variance, σ^{*2}_{min}, and thus the optimal embedding dimension.

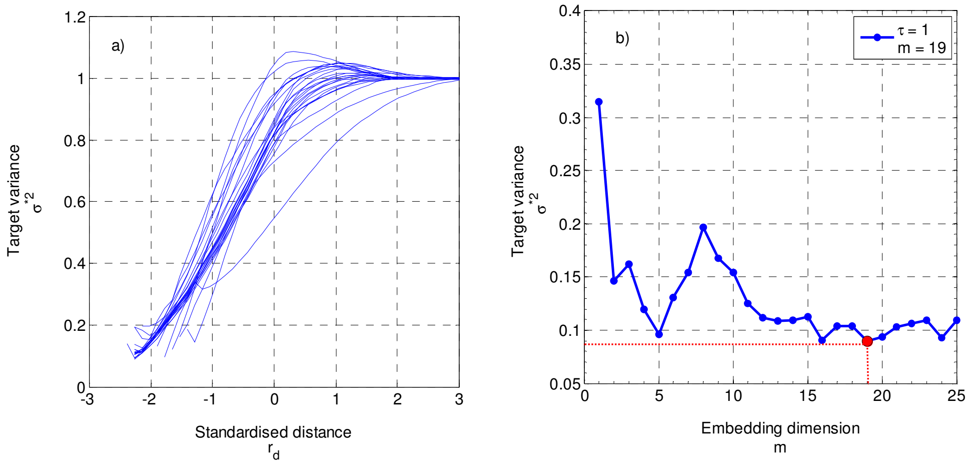

Figure A1. 4. Finding the optimal embedding parameter, m: a) DVV plots obtained for m = 2 to 25 and b) Target variance σ^{*2} for response of a SDOF undamped system under harmonic excitation as a function of embedding dimension, m.

A1.2.3 Method 3 – Manual Setting of Parameters

In Method 3, within the context of nonlinearity detection, *m* is not considered critical and the optimal embedding dimension of the original time series can be set manually. Gautama et al. [2] report this as a desirable property for a robust analysis method relative insensitivity of the DVV method to the parameter choice. The embedding dimension was set to 3, after observation of DVV plots of available experiments, and the time lag is set to unity for convenience. This convenience does not influence the generality of the results.

References

1. Gautama, T., D.P. Mandic, and M.M.V. Hulle, *Signal Nonlinearity in fMRI: A Comparison Between BOLD and MION.* IEEE Transactions on Medical Imagining, 2003. **22**(5): p. 636 - 644.
2. Gautama, T., D.P. Mandic, and M.M.V. Hulle, *The delay vector variance method for detecting determinism and nonlinearity in time series.* Physica D: Nonlinear Phenomena, 2004. **190**(3-4): p. 167–176.
3. Gautama, T., M.M.V. Hulle, and D.P. Mandic, *On the characterisation of the deterministic/stochastic and linear/nonlinear nature of time series*, in *DPM-04-05*. 2004, Imperial College London. p. 30.
4. Mandic, D.P., M. Chen, T. Gautama, M.M. Van Hulle, and A. Constantinides, *On the characterization of the deterministic/stochastic and linear/nonlinear nature of time series.* Proceedings of the Royal Society A: Mathematical, Physical and Engineering Science, 2008. **464**(2093): p. 1141-1160.
5. Schreiber, T. and A. Schmitz, *Surrogate time series.* Physica D: Nonlinear Phenomena, 2000. **142**(3–4): p. 346-382.
6. Xu, Z.-l., Y.-y. Wang, J.-l. Zhou, and P.-y. He, *Detecting the Nonlinear Determinism of a Room Acoustic System Using Surrogates.* Journal of Sichuan University (Engineering Science Edition) 2007. **39**(5): p. 155-158.
7. Gautama, T., D.P. Mandic, and M.M.V. Hulle, *Indications of nonlinear structures in brain electrical activity.* Physical Review E, 2003. **67**(4): p. 046204 (5).
8. Theiler, J., S. Eubank, A. Longtin, B. Galdrikian, and J. Doyne Farmer, *Testing for nonlinearity in time series: the method of surrogate data.* Physica D: Nonlinear Phenomena, 1992. **58**(1–4): p. 77-94.
9. Schreiber, T. and A. Schmitz, *Improved Surrogate Data for Nonlinearity Tests.* Physical Review Letters, 1996. **77**(4): p. 635-638.
10. Kugiumtzis, D., *Test your surrogate data before you test for nonlinearity.* Physical Review E, 1999. **60**(3): p. 2808-2816.
11. Mandic, D.P. *Delay Vector Variance MATLAB Toolbox*. 2010; Available from: http://www.commsp.ee.ic.ac.uk/~mandic/dvv.htm.
12. Kuntamalla, S. and R.G.L. Reddy, *The Effect of Aging on Nonlinearity and Stochastic Nature of Heart Rate Variability Signal Computed using Delay Vector Variance Method.* International Journal of Computer Applications 2011. **14**(5): p. 40-44.
13. Jianjun, Y., W. Haijun, X. Chunming, W. Yiqin, L. Fufeng, G. Rui, and M. Tiancai. *Nonlinear Analysis in TCM Acoustic Diagnosis Using Delay Vector Variance*. in *Bioinformatics and Biomedical Engineering, 2008. ICBBE 2008. The 2nd International Conference on*. 2008.

14. Gautama, T., D.P. Mandic, and M.M. Van Hulle. *A differential entropy based method for determining the optimal embedding parameters of a signal*. in *Acoustics, Speech, and Signal Processing, 2003. Proceedings. (ICASSP '03). 2003 IEEE International Conference on*. 2003.

15. Beirlant, J., E.J. Dudewicz, L. Gyorfi, and E.C.v.d. Meulen, *Nonparametric entropy estimation: An overview*. International Journal of Mathematical and Statistical Sciences, 2001. **6**: p. 17-39.

16. Hongying, H. and Y. Fuliang. *Diesel Engine Fault Information Acquisition Based on Delay Vector Variance Method*. in *Knowledge Acquisition and Modeling, 2009. KAM '09. Second International Symposium on*. 2009.
