# Supplementary material for: A comprehensive study of the delay vector variance method for quantification of nonlinearity in dynamical systems
Source: R Soc Open Sci. 2016 Jan 6;3(1):150493. doi: 10.1098/rsos.150493 (PMC4736930; doi:10.1098/rsos.150493)
Supplement: APPENDIX 2 [file rsos150493supp2.pdf]

APPENDIX 2

Reference Model and Simple Vibration Problems

Table of Contents

APPENDIX 2 ....

Reference Model and Simple Vibration Problems ....

List of Figures... ii

A2.1 SDOF Undamped Oscillation 1

A2.1.1 SDOF Undamped Oscillation – varying mass........................ 2

A2.2 A Damped SDOF System 3

A2.3 Overdamped SDOF Oscillation 4

A2.4 Harmonic Excitation of Undamped SDOF Systems 6

A2.5 Harmonic Excitation of Damped SDOF Systems 9

A2.6 Base Excitation of SDOF Systems 11

A2.7 SDOF Systems with a Rotating Unbalance 15

A2.8 Step Response of SDOF System 19

A2.9 Response of SDOF System to Square Pulse Inputs....................... 22

A2.10 Response of SDOF System to Ramp Input 25

A2.11 Modelling a van der Pol Oscillator... 27

A2.12 Response of SDOF System to Random Vibration....................... 29

A2.13 Randomly-Excited Duffing Oscillator.. 31

References 34

List of Figures

Figure A2.1 Typical SDOF free oscillator. 1

Figure A2.2 Responses of SDOF undamped system for different masses. 2

Figure A2.3 Typical damped SDOF oscillator. 3

Figure A2.4 Responses of SDOF damped system for different damping values. 4

Figure A2.5 Response of three overdamped system for decreasing damping. 5

Figure A2.6 SDOF system subject to external force. 6

Figure A2.7 SDOF undamped system response to harmonic load for increasing driving and set natural frequencies. 7

Figure A2.8 SDOF undamped system response to harmonic load for set driving and increased natural frequencies. 7

Figure A2.9 SDOF undamped system response to harmonic load – Beating phenomenon. 8

Figure A2.10 SDOF undamped system response to harmonic load – Resonance phenomenon. 9

Figure A2.11 Responses of damped SDOF system to harmonic loading for different damping values. 10

Figure A2.12 Responses of damped SDOF system to harmonic loading for different natural frequencies. 11

Figure A2.13 SDOF system subject to base excitation. 12

Figure A2.14 Responses of a base-excited SDOF system for different excitation frequencies. 14

Figure A2.15 Responses of a base-excited SDOF system for different base excitation magnitudes. 14

Figure A2.16 Responses of a base-excited SDOF system for different damping ratios. 15

Figure A2.17 SDOF System with Rotating Unbalance. 15

Figure A2.18 Responses of a SDOF system with different natural frequencies to a rotating unbalance. 17

Figure A2.19 Responses of a SDOF system with varying damping ratio to a rotating unbalance........ 18

Figure A2.20 Responses of a SDOF system with varying system mass to a rotating unbalance........ 19

Figure A2.21 Step response of SDOF system to different step magnitudes........ 21

Figure A2.22 Step response of SDOF system having different natural frequencies. 21

Figure A2.23 Step response of SDOF system to different levels of damping........ 22

Figure A2.24 Response of SDOF systems to square pulse inputs for different force magnitudes. 24

Figure A2.25 Response of SDOF systems to square pulse inputs for different natural frequencies. 24

Figure A2.26 Response of SDOF systems to square pulse inputs for different damping ratio. 25

Figure A2.27 Response of SDOF system to Ramp input for different rates of loading. 26

Figure A2.28 Response of SDOF system to Ramp input for different rates of loading – focusing on first few seconds of oscillation, showing that system oscillate during the transient period. 27

Figure A2.29 Displacement and velocity vs. time for the van der Pol oscillator. 28

Figure A2.30 Velocity vs. Displacement for van der Pol oscillator. 29

Figure A2.31 Response of SDOF system to Random Vibration 31

Figure A2.32 Response of the Duffing oscillator to amplitude A = 3.7999 and forcing frequency ω = 3.7960........ 32

Figure A2.33 Response of the Duffing oscillator to amplitude A = 4.4531 and forcing frequency ω = 1.7404........ 33

Figure A2.34 Response of the Duffing oscillator to amplitude A = 1.0062 and forcing frequency ω = 2.0115........ 33

A2.1 SDOF Undamped Oscillation

The simplest form of vibration that we can study is the single degree of freedom system without damping or external forcing. A sample of such a system is shown in Figure A2.1.

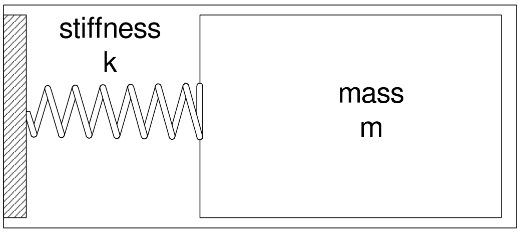

Figure A2.1 Typical SDOF free oscillator.

The mechanical system equation of motion is:

$$m\ddot{x} + kx = 0 \qquad (A2.1)$$

where m is mass, k is stiffness and x is displacement. In general, we would have the forcing function F (t) on the right-hand side but it is assumed zero for this analysis. Dividing through by m and introducing parameter $\omega_n = \sqrt{\frac{k}{m}}$ the solution is obtained:

$$x(t) = Asin(\omega_n t + \phi) \qquad (A2.2)$$

where A is amplitude, ω_n is natural frequency, t is period and ϕ is phase angle. In the terms of physical parameters of the system:

$$x(t) = \frac{\sqrt{\omega_n^2 x_0^2 + \dot{x}_0^2}}{\omega_n} cos(\omega_n t - tan^{-1}\frac{\dot{x}_0}{\omega_n x_0}) \qquad (A2.3)$$

From equation (A2.3), the complete response of an undamped, unforced, one degree of freedom oscillator depends on three physical parameters: ω_n, x_0 and $\dot{x}_0$ (e.g. the natural frequency, initial velocity, and initial displacement, respectively). It is also evident that the phase angle and maximum amplitude are also functions of the natural frequency.

From the definition of the natural frequency, we see that it is inversely proportional to $\sqrt{m}$, and is directly proportional to $\sqrt{k}$. Variation of mass or stiffness, then, will cause a variation in the frequency of vibration. Therefore we looked at case of varying mass. The initial conditions adopted are: velocity is $\dot{x}_0 = 1$, and the initial displacement $x_0 = 3$.

A2.1.1 SDOF Undamped Oscillation – varying mass

Figure A2.2 shows the variation of the vibrational characteristics for an increasing mass (m = 2, 4 and 12 kg) while stiffness remains constant (k = 8 N/m).

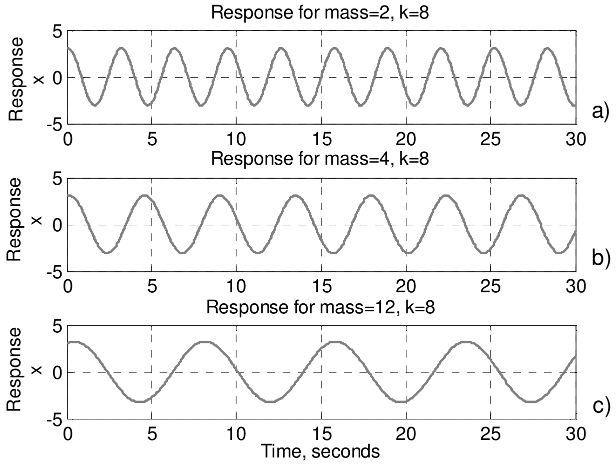

Figure A2.2 Responses of SDOF undamped system for different masses.

The frequency decreases with increasing mass; hence it would increases with increasing stiffness, as expected. Also, the maximum amplitude decreases with increasing mass, due to the corresponding reduction in natural frequency. As a result, the phase shift diminishes, with the peak of oscillation becoming nearer to t = 0. The maximum displacement would occur at t = 0 if the initial velocity were zero. For this case, the parameter A (see equation A2.2) reduces to x_0, and the phase angle becomes 0º.

A2.2 A Damped SDOF System

The equation of motion for a damped single degree of freedom oscillator shown in Figure A2.3 can be written:

$$m\ddot{x} + c\dot{x} + kx = 0 \qquad (A2.4)$$

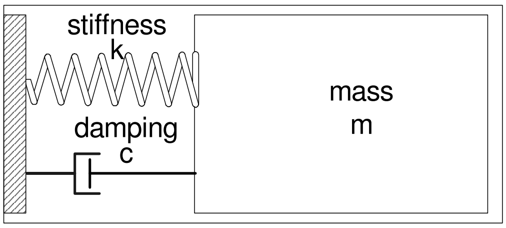

Figure A2.3 Typical damped SDOF oscillator.

If we divide through by m, we introduce the dimensionless parameters ω and ζ:

$$\ddot{x} + 2\zeta\omega_n\dot{x} + \omega_n^2 x = 0 \qquad (A2.5)$$

where ω_n represents the undamped natural frequency, and ζ is the viscous damping ratio. For the purposes of this example, it is assumed the underdamped case ($\zeta < 1$). The solution to this equation is:

$$x(t) = Ae^{-\zeta\omega_n t}sin(\omega_d t + \phi) \qquad (A2.6)$$

where $\omega_d = \omega_n\sqrt{1-\zeta^2}$ is damped natural frequency. The equation A2.6 can be written in the function of the parameters ω_n and ζ:

$$x(t) = \sqrt{\frac{(v_0 + \zeta\omega_n x_0)^2 + \left(x_0\omega_n\sqrt{1-\zeta^2}\right)^2}{\left(\omega_n\sqrt{1-\zeta^2}\right)^2}}\, e^{(-\zeta\omega_n t)} sin\left[\left(\omega_n\sqrt{1-\zeta^2}\right)t + tan^{-1}\left(\frac{x_0\omega_n\sqrt{1-\zeta^2}}{v_0 + \zeta\omega_n x_0}\right)\right] \qquad (A2.7)$$

The response of the system therefore only depends on four quantities: x_0, v_0, ω_n and ζ (e.g. the initial displacement, initial velocity, natural frequency, and, viscous

damping coefficient, respectively). The only difference to the undamped case is existence of viscous damping coefficient. The effects of the increasing viscous damping coefficient ($\zeta = 0.05$, 0.2 and 0.5) on the system response are shown in Figure A2.4. The initial conditions adopted are: velocity is $v_0 = 1$, and the initial displacement $x_0 = 3$ while natural frequency is set to $\omega_n = 7$.

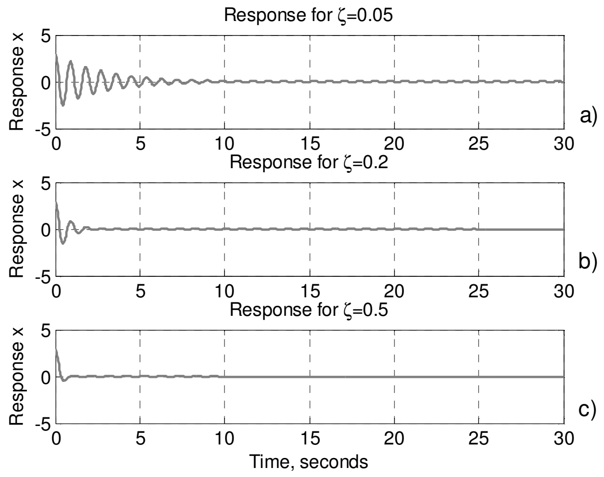

Figure A2.4 Responses of SDOF damped system for different damping values.

Note how quickly the response becomes virtually zero; this occurs within ten seconds, even for a damping coefficient as small as 0.05. The Matlab code used in this analysis only works for the underdamped case since the term $\omega_d = \omega_n\sqrt{1-\zeta^2}$ is in the denominator of the response equation which would lead to division by zero for $\zeta = 1$, and when $\zeta > 1$ will give an imaginary damped natural frequency.

A2.3 Overdamped SDOF Oscillation

The equation A2.5 represents equation of motion of a damped single degree of freedom oscillator. Assume a solution of the form $Ae^{\lambda t}$ substitute it into equation A2.5, and obtain the quadratic formula defining possible values for λ:

$$\lambda = -\zeta\omega_n \pm \omega_n\sqrt{\zeta^2 - 1} \qquad (A2.8)$$

Since it was assumed that $\zeta > 1$ the quantity inside the radical is always greater than zero. Therefore, the solution of the equation of motion is:

$$x(t) = e^{-\zeta\omega_n t}\left(a_1 e^{\omega_n t\sqrt{\zeta^2-1}} + a_2 e^{-\omega_n t\sqrt{\zeta^2-1}}\right) \qquad (A2.9)$$

If initial displacement is x_0 and initial velocity is v_0, the constants a_1 and a_2 become

$$a_1 = \frac{-v_0 + \left(-\zeta + \sqrt{\zeta^2 - 1}\right)\omega_n x_0}{2\omega_n\sqrt{\zeta^2 - 1}} \qquad (A2.10)$$

$$a_2 = \frac{v_0 + \left(\zeta + \sqrt{\zeta^2 - 1}\right)\omega_n x_0}{2\omega_n\sqrt{\zeta^2 - 1}} \qquad (A2.11)$$

Equation A2.9 is a decaying exponential and the system will simply return to its initial position instead of oscillating about the equilibrium. This is shown in Figure A2.5. Note that if $\zeta = 1$, a singularity exists in the constants; a second independent solution must be found; from ordinary differential equations, we can find response of such system

$$x(t) = (a_1 + a_2 t)e^{-\omega_n t} \qquad (A2.12)$$

Where $a_1 = x_0$ and $a_2 = v_0 + \omega_n x_0$.

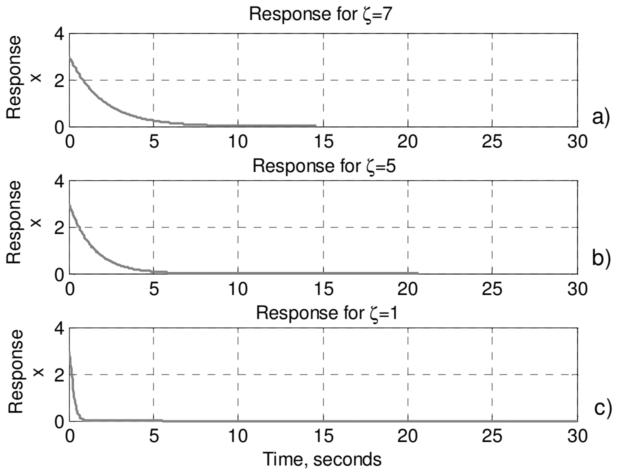

Figure A2.5 Response of three overdamped system for decreasing damping.

Figure A2.5 was generated for $\omega_n = 7$; $x_0 = 3$; and $v_0 = 1$. The critically damped response returns to equilibrium faster than the others. For the plots in the

figure, the motion with critical damping is stopped after about two seconds, while the others do not reach equilibrium until more than eight seconds. This is the distinguishing characteristic of the critically damped case. Also the motion of the masses is, as expected, purely exponential; there is no oscillation, only a decay of the response to equilibrium.

A2.4 Harmonic Excitation of Undamped SDOF Systems

The effects of an external force on the system are examined next. The simplest form of external force, harmonic load, is adopted and system under consideration is shown in Figure A2.6.

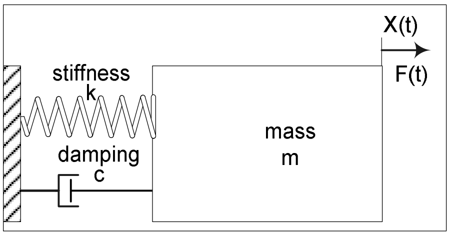

Figure A2.6 SDOF system subject to external force.

The assumed form of external force is:

$$F(t) = F_o cos\omega t \qquad (A2.13)$$

where ω is driving frequency. When there is no damping, Newton's Second Law gives us the equation of motion:

$$m\ddot{x} + kx = F_o cos\omega t \qquad (A2.14)$$

$$\ddot{x} + \omega_n^2 x = f_o cos\omega t \qquad (A2.15)$$

where $f_o = F_o/m$. The solution for response $x(t)$ is:

$$x(t) = A_1 sin\omega_n t + A_2 cos\omega_n t + \frac{f_o}{\omega_n^2 - \omega^2} cos\omega t \qquad (A2.16)$$

where constants are: $A_1 = \frac{v_0}{\omega_n}$ and $A_2 = \frac{f_o}{\omega_n^2 - \omega^2}$. The key parameters which define the response are the natural and driving frequencies, or more precisely, their ratio ω/ω_n. Figure A2.7 shows the effect of varying driving frequency ω (in the figure indicated as ω_{dr}) for a given natural frequency. Figure A2.8 the same for various natural frequencies ω_n. The figures are generated using initial displacement, amplitude and force magnitude per unit mass: $v_0 = 0$ $x_0 = 0$; and $f_0 = 6$, respectively. Making the initial conditions zero allows us to better see the effects of varying frequencies.

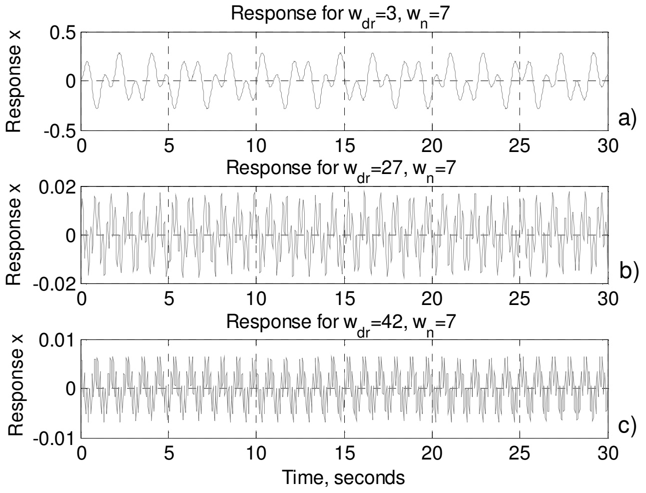

Figure A2.7 SDOF undamped system response to harmonic load for increasing driving and set natural frequencies.

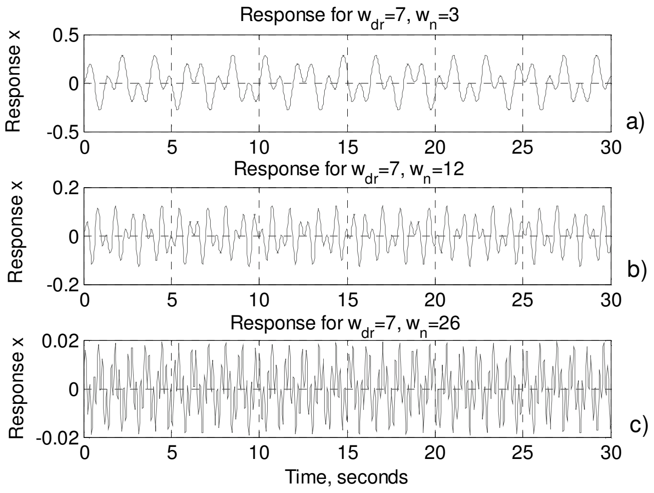

Figure A2.8 SDOF undamped system response to harmonic load for set driving and increased natural frequencies.

The fact that two of the three constants in the expression for x(t), equation A2.16, involve the difference between the frequencies gives rise to two interesting phenomena: beats and resonance. Beats occur when the natural frequency and the driving frequency are close but not equal. The result is then a rapid oscillation with slowly varying amplitude, as shown in Figure A2.9. The rapid oscillation and the slow change of the amplitude both vary along a sinusoid.

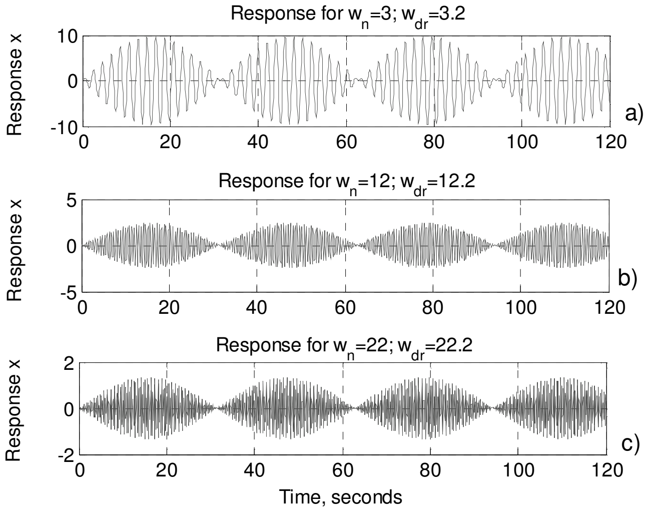

Figure A2.9 SDOF undamped system response to harmonic load – Beating phenomenon.

When the driving and natural frequencies are equal, $\omega_{dr} = \omega_n$ resonance is the result. The third term in Equation A2.16 is not valid as a particular solution of the governing equation of motion. Instead, the particular solution is:

$$x_p(t) = \frac{f_o}{2\omega_n} t sin\omega_n t \qquad (A2.17)$$

In this case the amplitude of oscillation will increase without limit. In a real system, the stiffness element has a certain yield point which will be met and exceeded by a resonant vibration. Figure A2.10 shows resonant vibration.

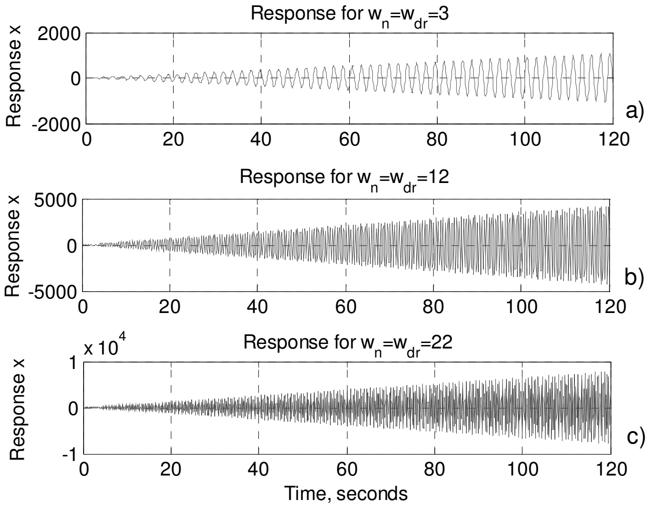

Figure A2.10 SDOF undamped system response to harmonic load – Resonance phenomenon.

A2.5 Harmonic Excitation of Damped SDOF Systems

The equation of motion of damped SDOF System excited with the harmonic forcing can be written:

$$m\ddot{x} + c\dot{x} + kx = Fcos\omega t \qquad (A2.18)$$

$$\zeta = c/2m\omega_n \qquad (A2.19)$$

$$\ddot{x} + 2\zeta\omega_n\dot{x} + \omega_n^2 x = fcos\omega t \qquad (A2.20)$$

where $f = F/m$. The homogeneous solution to equation A2.20 is of the form:

$$x_h(t) = Ae^{-\zeta\omega t}sin(\omega_d t + \theta) \qquad (A2.21)$$

$$\omega_d = \omega_n\sqrt{1-\zeta^2} \qquad (A2.22)$$

Constants A and θ depend on initial conditions. The particular solution to the external force is:

$$x_p(t) = A_0 cos(\omega t - \phi) \qquad (A2.23)$$

where the constants are:

$$A_0 = \frac{f}{\sqrt{(\omega_n^2 - \omega^2)^2 + (2\zeta\omega_n\omega)^2}} \tag{A2.24}$$

$$\phi = tan^{-1}\frac{2\zeta\omega_n\omega}{\omega_n^2 - \omega^2} \tag{A2.25}$$

The complete solution:

$$x(t) = x_h(t) + x_p(t) \tag{A2.26}$$

is used to evaluate constants, A and θ. These constants are found for zero initial conditions. The damping causes the response of the system to differ slightly, as shown in Figure A2.11 and Figure A2.12.

In Figure A2.11, the damping ratio ζ was varied, which shows that the transient period of vibration varies inversely with damping ratio. The length of the transient period varies from about 4.5 seconds for ζ = 0.05 to about 1.5 seconds for ζ = 0.5; showing that, in many cases, the transient response can be ignored due to its short time period. However, for some cases, the transient period may be much longer or may have very large amplitude, so it is always important to examine the transient effects of a system before neglecting them. It is also noticeable that the damping ratio affects the amplitude of the steady-state vibration, also in an inverse relationship. That is, the amplitude of the response for ζ = 0.05 is almost 2, while that for ζ = 0.5 is less than 1.

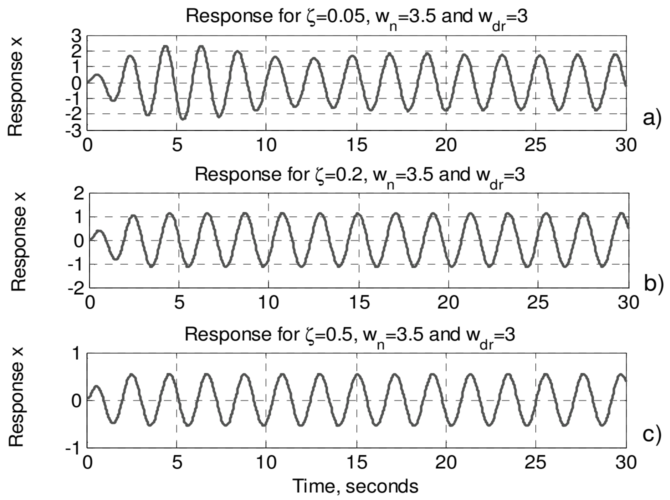

Figure A2.11 Responses of damped SDOF system to harmonic loading for different damping values.

Figure A2.12 shows the effects of changing the natural frequency. For the two frequencies that are near the driving frequency, the transient period is quite long, almost 10 seconds. However, for the large natural frequency, the transient period is less than 4 seconds, which shows that the length of the transient period also depends on the natural frequency. In the damped system, resonance also takes on a different meaning (for $\omega = \omega_n$ the amplitude does not become infinite) the introduction of damping introduces a term that keeps the denominator of the steady-state amplitude from becoming zero. However, at this point, the phase angle becomes 90º. For a damped system, this condition defines resonance; since it is also at this point that the denominator of the amplitude is a minimum (i.e. the amplitude will be maximized when the denominator is minimized and both terms are never negative, so the minimum will occur when the two frequencies are equal; making the first term of the denominator zero). Also, as the driving frequency increases greatly, the amplitude nears zero.

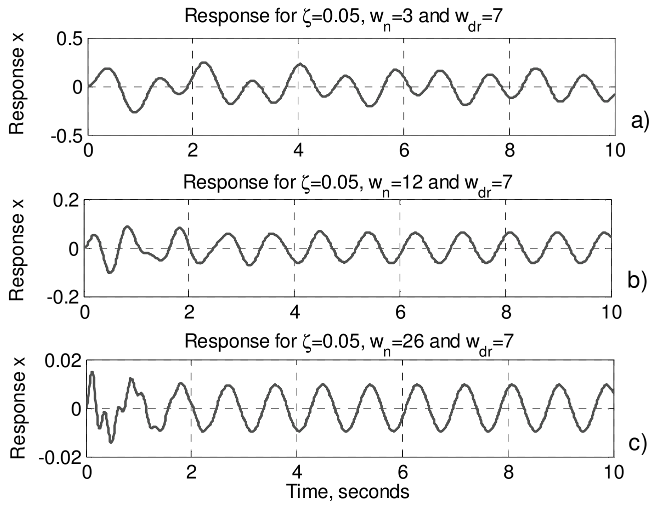

Figure A2.12 Responses of damped SDOF system to harmonic loading for different natural frequencies.

A2.6 Base Excitation of SDOF Systems

The equation of the motion for the system with base excitation shown in Figure A2.13 is:

$$m\ddot{x} + c(\dot{x} - \dot{y}) + k(x - y) = 0 \qquad (A2.27)$$

where the base motion is $y(t)$ and the response of the mass by $x(t)$. Using assumed form for the motion:

$$y(t) = Ysin(\omega_b t) = 0 \qquad (A2.28)$$

we can substitute for y and its derivative, resulting in:

$$m\ddot{x} + c\dot{x} + kx = cY\omega_b cos(\omega_b t) + kYsin(\omega_b t) \qquad (A2.29)$$

which when divided through by the mass, yields:

$$\ddot{x} + 2\zeta\omega\dot{x} + \omega^2 x = 2\zeta\omega\omega_b Ycos(\omega_b t) + \omega^2 Ysin(\omega_b t) \qquad (A2.30)$$

The homogeneous solution is of the form:

$$x_h(t) = Ae^{-\zeta\omega t}\, sin(\omega_d t + \theta) \qquad (A2.31)$$

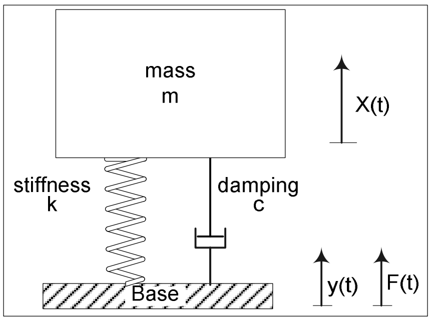

Figure A2.13 SDOF system subject to base excitation.

The expression for each part of the particular solution is similar to that for the general sinusoidal forcing function; the sine term produces a sine solution, and the cosine term produces a cosine solution. If we find these solutions and combine their sum into a single sinusoid, we obtain:

$$x_p(t) = A_0\, cos(\omega_b t - \phi_1 - \phi_2) \qquad (A2.32)$$

Constants are:

$$A_0 = \omega Y \sqrt{\frac{\omega^2 + (2\zeta\omega_b)^2}{(\omega^2 - \omega_b^2)^2 + (2\zeta\omega\omega_b)^2}} \tag{A2.33}$$

$$\phi_1 = tan^{-1}\frac{2\zeta\omega_b\omega}{\omega^2 - \omega_b^2} \tag{A2.34}$$

$$\phi_2 = tan^{-1}\frac{\omega}{2\zeta\omega_b} \tag{A2.35}$$

Thus, the complete solution is the sum of the homogeneous and particular solutions, or:

$$x(t) = Ae^{-\zeta\omega t}sin(\omega_d t + \theta) + A_0 cos(\omega_b t - \phi_1 - \phi_2) \tag{A2.36}$$

From equation A2.36 one can notice that the particular solution represents the steady-state response, while the homogeneous solution is the transient response, since the particular solution is independent of the initial displacement and velocity. After solving equation for initial velocity and displacement (which are not necessarily equal to zero), it was found that both are dependent upon the initial velocity and displacement. However, the expression for the constants A and θ is, in general, very difficult to solve therefore the initial velocity and displacement were both assumed to be zero.

Figure A2.14 shows the effects of changing the excitation (base) frequency while holding all other parameters constant. In the steady state, from about three seconds forward, the frequency of vibration increases with the base frequency. This is expected, since the base excitation portion dominates the steady state. Figure A2.14 (c) where ω_b =12, in the transient portion, the response has the shape of a sum of two sinusoids; these are, of course, the transient and steady-state functions. Since the base excitation is of such high frequency, this graph shows best what is happening between the transient and steady responses. Note that, if a line was drawn through the upper or lower peaks of the motion, the result would be a curve similar to that shown by a damped free response (section A2.2. A Damped SDOF System). The midpoint of the

oscillation caused by the steady response becomes exponentially closer to zero with increasing time, as the transient response diminishes.

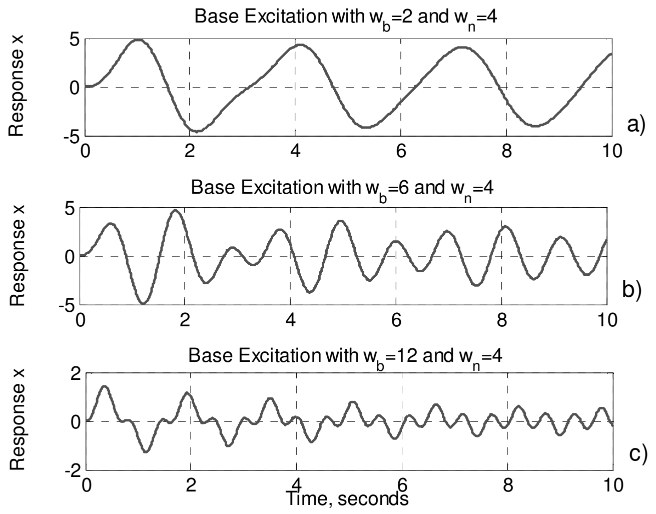

Figure A2.14 Responses of a base-excited SDOF system for different excitation frequencies.

Figure A2.15 shows plots for three different vibration amplitudes. The differences caused by changing the amplitude is what would be expected; the maximum amplitude of the overall vibration and of the steady-state response both increase with increasing input amplitude.

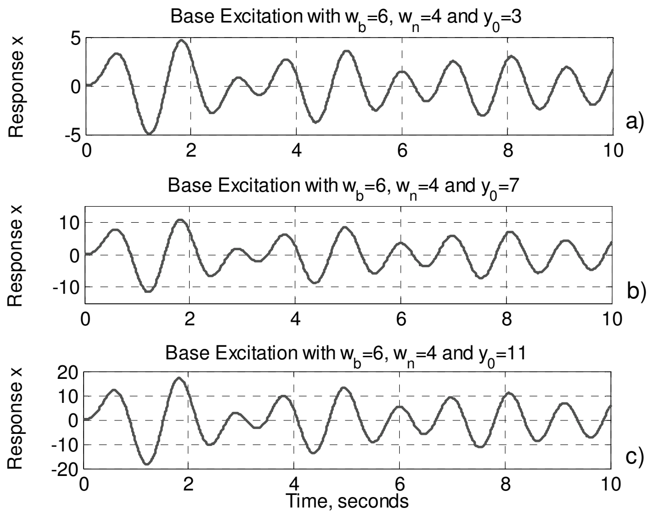

Figure A2.15 Responses of a base-excited SDOF system for different base excitation magnitudes.

The plots in figure A2.16 for various damping ratios show two effects of changing the damping ratio. First, the change in damping ratio causes the length of the

transient period to vary; an increase in ζ causes the transient period to decrease, as the plots show. Also, the change in damping ratio causes a change in the frequency of the transient vibration. Again, an increase in ζ causes a decrease in the damped natural frequency. Because the plots also include the base excitation (steady-state) terms, whose frequency has not changed, the decrease is not entirely evident from just looking at the plots. The initial displacements are zero for all plots, as are the initial velocities.

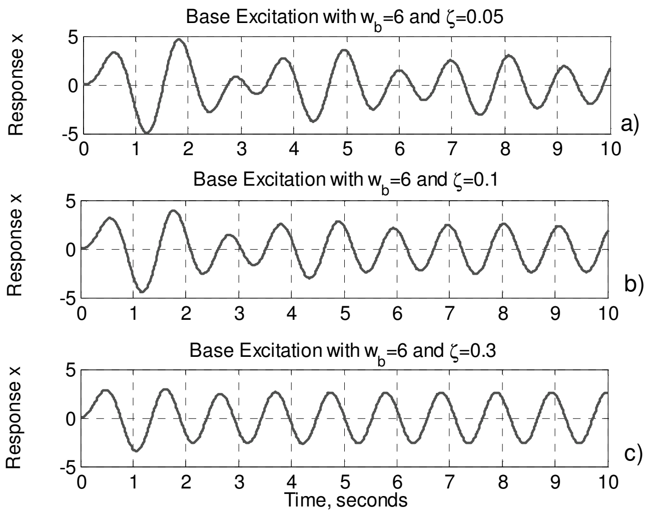

Figure A2.16 Responses of a base-excited SDOF system for different damping ratios.

A2.7 SDOF Systems with a Rotating Unbalance

A SDOF System with rotating unbalance and assumed coordinates is shown in Figure A2.17.

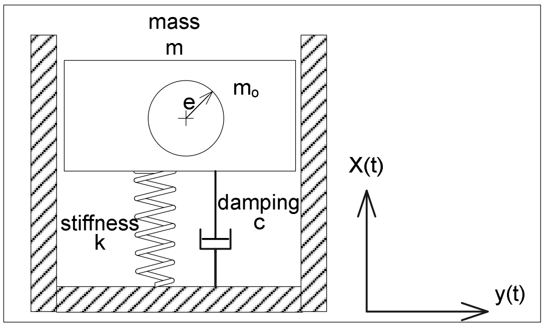

Figure A2.17 SDOF System with Rotating Unbalance.

It is assumed that the guides are frictionless. The radius e is measured from the center of the mass m. To write the equation of motion, we need an expression for the motion of the rotating unbalance in terms of displacement x. If the mass rotates with a constant angular velocity ω_r then parametrically the circle it defines can be described as:

$$x(t) = e\ sin\ \omega_r t \qquad (A2.37)$$

$$y(t) = e\ cos\ \omega_r t \qquad (A2.38)$$

With the coordinate x being vertical, the position coordinate of the rotating unbalance is defined as $x + sin\omega_r t$ and the acceleration is the second derivative of this expression with respect to time. The acceleration of the mass without the unbalance is $\ddot{x}$; adding in the effects of the stiffness and damper the equation of motion is:

$$(m - m_o)\ddot{x} + m_o \frac{d^2}{dt^2}(x + e\ sin\omega_r t) = -kx - c\dot{x} \qquad (A2.39)$$

$$(m - m_o)\ddot{x} + m_o(\ddot{x} + e\omega_r^2\ sin\omega_r t) = -kx - c\dot{x} \qquad (A2.40)$$

Collecting x and its derivatives, moving the sine term to the other side of the expression, and dividing by the system mass, the equation of motion can be written in form:

$$\ddot{x} + 2\zeta\omega_n\dot{x} + \omega_n^2 x = m_o e\omega_r^2 sin\omega_r t \qquad (A2.41)$$

This is identical to the harmonic forcing function case (section A2.5 Harmonic Excitation of Damped SDOF Systems) except that the force is in the form of a sine rather than a cosine. For that reason, the particular solution is of the form:

$$x_p(t) = A_1\ sin(\omega_r t - \phi) \qquad (A2.42)$$

If the ratio of rotating and natural frequency is $r = \omega_r/\omega_n$ the constants are:

$$A_1 = \frac{m_o e}{m}\frac{r^2}{\sqrt{(1 - r^2)^2 + (2\zeta r)^2}} \qquad (A2.43)$$

$$\phi = tan^{-1}\frac{2\zeta r}{1 - r^2} \qquad (A2.44)$$

The homogenous solution for this expression is:

$$x_h(t) = Ae^{-\zeta\omega_n t}\, sin(\omega_d t + \theta) \qquad (A2.45)$$

constants A and θ are determined from the initial conditions. The final solution is:

$$x(t) = x_p(t) + x_h(t) = A_1\, sin(\omega_r t - \phi) + Ae^{-\zeta\omega_n t}\, sin(\omega_d t + \theta) \qquad (A2.46)$$

For modelling purposes the initial conditions were assumed to be zero and, unless otherwise specified, m = 7; m_o = 3; and e = 0,1. The solution to this is not reproduced here, due to the complexity of the expression; the solution for A and θ depend on the solution to a quadratic equation. In following figures effects of different varying parameters on the system were explored.

For Figure A2.18, the natural frequency was varied while holding all other parameters constant. In the case when ω_n is not a multiple of ω_r the motion is the sum of two sinusoids (Figure A2.18 (a)). For the highest natural frequency tested, the oscillation occurs along a single sinusoid. This is because the natural frequency of the system is too high to be excited by the relatively slow rotation frequencies. The first two plots, (a) and (b), have natural frequencies small enough to be excited by the slow rotation of the eccentric mass.

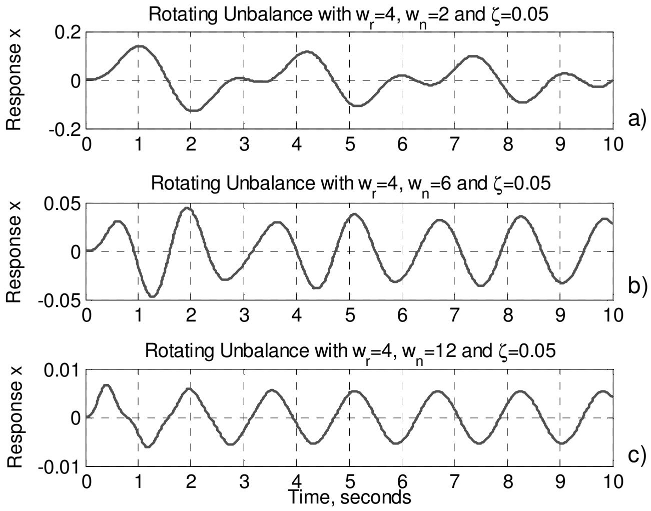

Figure A2.18 Responses of a SDOF system with different natural frequencies to a rotating unbalance.

In Figure A2.19, the system damping is varied. The result is that the transient portion (the portion with the curve that looks like the sum of sinusoids) becomes smaller, to the point where it disappears at $\zeta = 0.3$. A difference in the magnitude of oscillation, as would be predicted from the expression we have derived for the parameter A_1 is not present because the frequency ratio we are testing is in the range where oscillation magnitude shows little variation with damping ratio. This consideration is important in the design of machinery; if the machine can be designed to have a much higher natural frequency than the oscillating mass, then the level of damping can be made low without increasing the amplitude past acceptable levels.

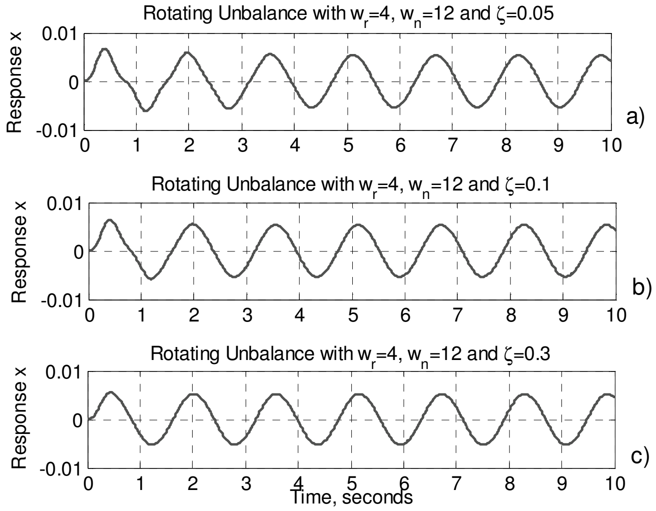

Figure A2.19 Responses of a SDOF system with varying damping ratio to a rotating unbalance.

Figure A2.20 shows the variation of vibration with increasing system mass. The amplitude of the vibration decreases with increasing mass which is this is due to the dependence of A_1 on the mass ratio, i.e. as m_o/m decreases, so does the amplitude of vibration.

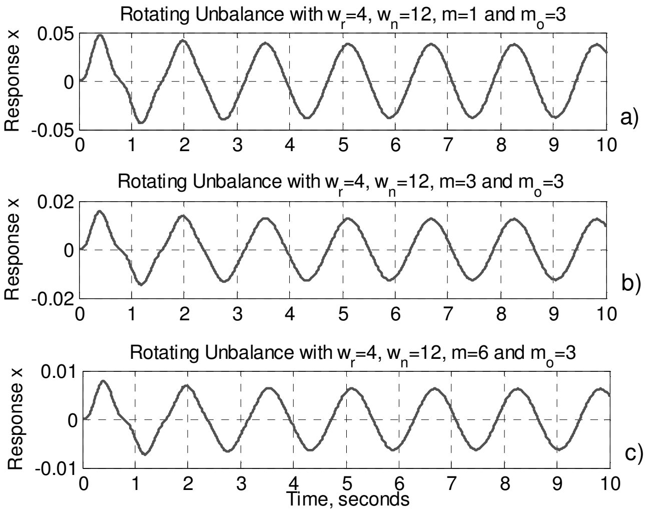

Figure A2.20 Responses of a SDOF system with varying system mass to a rotating unbalance.

A2.8 Step Response of SDOF System

The force is assumed to be applied instantaneously, but it is sustained out to infinity. If a force of this sort is plotted versus time, the force looks like a step up. The behaviour of the system under this type of load is considered the step response of the SDOF system. It is assumed that the system is underdamped ($\zeta < 1$) and will have zero initial conditions. The equation of motion of the system is:

$$\ddot{x} + 2\zeta\omega_n\dot{x} + \omega_n^2 x = F(t)/m \qquad (A2.47)$$

where

$$F(t) = \begin{cases} 0 & if \ \ 0 < t < t_o \\ F_o & if \qquad t \geq t_o \end{cases} \qquad (A2.48)$$

In order to solve the differential equation, the convolution integral was used:

$$x(t) = \int_0^t F(\tau)g(t-\tau)\,d\tau \qquad (A2.49)$$

The convolution integral is derived by treating the force as an infinite series of impulse forces; hence the infinite series can be treated as the integral given above. The impulse response can be expressed:

$$x(t) = \frac{F_o}{m\omega_d} e^{-\zeta\omega_n t} sin\omega_d t = F_o g(t) \qquad (A2.50)$$

Where

$$g(t) = \frac{1}{m\omega_d} e^{-\zeta\omega_n t} sin\omega_d t \qquad (A2.51)$$

Therefore,

$$x(t) = \frac{1}{m\omega_d} e^{-\zeta\omega_n t} \int_0^t F(\tau) e^{\zeta\omega_n \tau} sin\omega_d (t-\tau) d\tau \qquad (A2.52)$$

Substituting F(t) into (A2.8f):

$$x(t) = \frac{1}{m\omega_d} e^{-\zeta\omega_n t} \left\{ \int_0^{t_o} F(0) e^{\zeta\omega_n \tau} sin\omega_d (t-\tau) d\tau + \int_{t_o}^t F_o e^{\zeta\omega_n \tau} sin\omega_d (t-\tau) d\tau \right\} \qquad (A2.53)$$

The first term inside the brackets is zero, hence, for $t < t_o$, the response of the system is zero. To find the response for all other times, the second integral needs to be evaluated (by parts):

$$x(t) = \frac{F_o}{k} \left\{ 1 - \frac{1}{\sqrt{1-\zeta^2}} e^{-\zeta\omega_n (t-t_o)} cos[\omega_d (t-t_o) - \phi] \right\} \; for \; t \geq t_o \qquad (A2.54)$$

$$\phi = tan^{-1} \frac{\zeta}{\sqrt{1-\zeta^2}} \qquad (A2.55)$$

This equation (A2.54) is only valid for the time after the force is applied; the response is zero before application of the force.

Figure A2.21 shows the variation of the response with the force magnitude. The only difference that result from changing the magnitude of the external force is that the magnitude of the response changes. That is, the magnitude of the response is

directly proportional to the magnitude of the external force. The magnitude of the external force also causes a second difference, i.e. when the oscillatory motion begins, it is not centered around zero. Instead, the mass oscillates around a displacement greater than zero. The value of this center point is also dependent on the magnitude of the external force (see first term in equation A2.53).

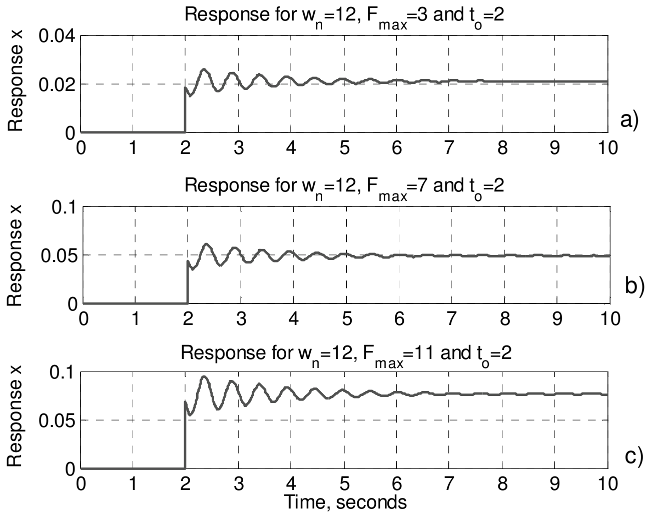

Figure A2.21 Step response of SDOF system to different step magnitudes.

Figure A2.22 shows the step responses of SDOF system when vary the natural frequency. This causes two changes in the response. First, the rate of exponential decrease in the response (the effect of damping) is increased; that is, the response stabilizes more quickly. Second, the oscillation frequency decreases, since the natural frequency also dictates the damped frequency.

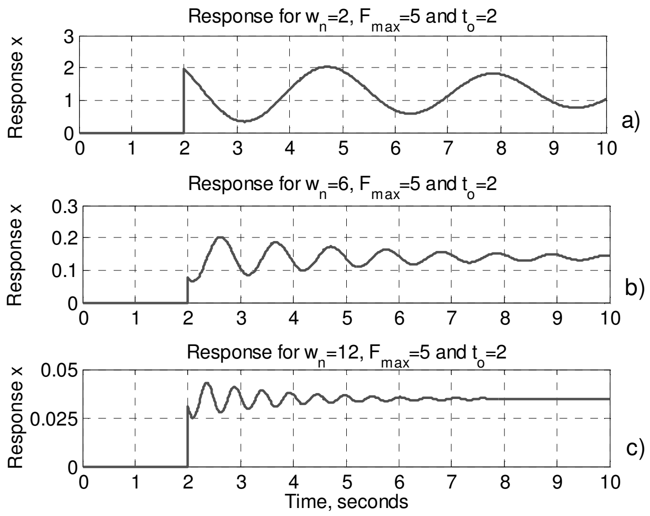

Figure A2.22 Step response of SDOF system having different natural frequencies.

Figure A2.23 shows the changes caused by changing the damping ratio. With increasing damping ratio, the amount of time to damp out all vibration decreases. For the third ratio tested, $\zeta = 0.3$, the damping is sufficient to allow no oscillation around the new center point (x = 1.5). A second result, which is not immediately evident from the figure but follows from the mathematics, is that the phase angle changes with the damping ratio (ϕ is a function of only ζ).

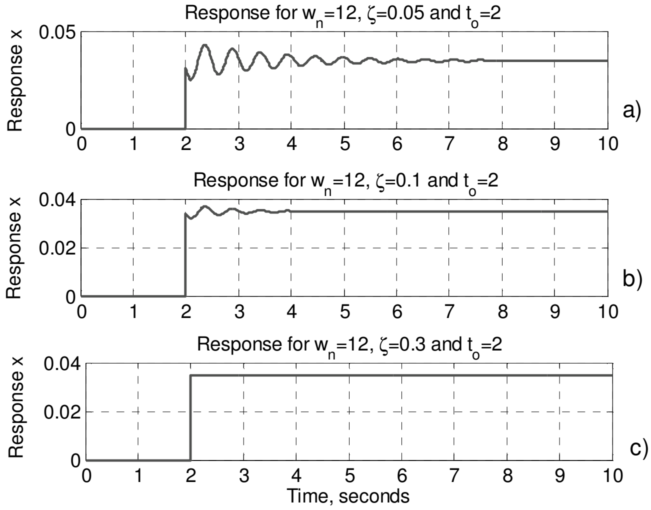

Figure A2.23 Step response of SDOF system to different levels of damping.

A2.9 Response of SDOF System to Square Pulse Inputs

A square pulse is a single pulse of constant magnitude and finite duration. To analyse the response of systems to a square wave input, the square wave is treated as the sum of two equal and opposite step inputs applied at different times. The time interval between applications of the step inputs is the duration of the square wave. If the magnitude of the square wave is F_o, and its duration is t_1 seconds, to simulate the wave using step inputs, we begin with a step input of magnitude F_o from time $t = 0$; and add to it at time t_1 a step input of magnitude $-F_o$. By superposition, the total response is the sum of the response of the system to each step input.

As per section A2.8 the response of a single degree of freedom system to a step input of magnitude F_m applied at time t_o is:

$$x(t) = \frac{F_m}{k}\left\{1 - \frac{1}{\sqrt{1-\zeta^2}} e^{-\zeta\omega_n(t-t_o)} cos[\omega_d(t-t_o) - \phi]\right\} \; for \; t \geq t_o \qquad (A2.56)$$

where

$$\phi = tan^{-1}\frac{\zeta}{\sqrt{1-\zeta^2}} \qquad (A2.57)$$

Considering the two step inputs separately and denoting the response of the system to the input at time $t = 0$ as $x_1(t)$ and the response to the input at time $t = t_1$ as $x_2(t)$:

$$x_1(t) = \frac{F_o}{k}\left\{1 - \frac{1}{\sqrt{1-\zeta^2}}e^{-\zeta\omega t}cos[\omega_d t - \phi]\right\} \; for\; t \geq 0 \qquad (A2.58)$$

$$x_2(t) = -\frac{F_o}{k}\left\{1 - \frac{1}{\sqrt{1-\zeta^2}}e^{-\zeta\omega(t-t_1)}cos[\omega_d (t-t_1) - \phi]\right\} \; for\; t \geq t_1 \qquad (A2.59)$$

The total response is:

$$x(t) = \frac{F_o}{k}\left\{1 - \frac{1}{\sqrt{1-\zeta^2}}e^{-\zeta\omega_n t}cos[\omega_d t - \phi]\right\} \; for\; 0 \leq t < t_1 \qquad (A2.60)$$

$$x(t) = \frac{F_o e^{-\zeta\omega_n t}}{k\sqrt{1-\zeta^2}}\left\{e^{\zeta\omega_n t_1}cos[\omega_d (t-t_1) - \phi] - cos(\omega_d t - \phi)\right\} for\; t \geq t_1 \qquad (A2.61)$$

For the time interval after period t_1, the response no longer includes a (1-) term; the addition of the two responses has removed this term entirely. This term caused the oscillation to be about a new equilibrium (i.e., $x = F_o/k$). Now, since the term has disappeared, the oscillation is centred around zero.

The movement of the center point of the oscillation is best shown in Figure A2.24. This figure shows response of the system for three different values of F_o. The oscillation begins about a center point at $x = F_o/k$; when the square wave ends, or, when the equal and opposite step is added, the center point returns to zero. If assumed that the magnitude of the second step (F_1) is not equal to that of the first, the center point of the oscillation after adding the second step input would be at $x = (F_o - F_1)/k$. From Figure A2.24 is also evident that the change in F_o causes the magnitude of the oscillations to increase, as expected from Equation A2.61.

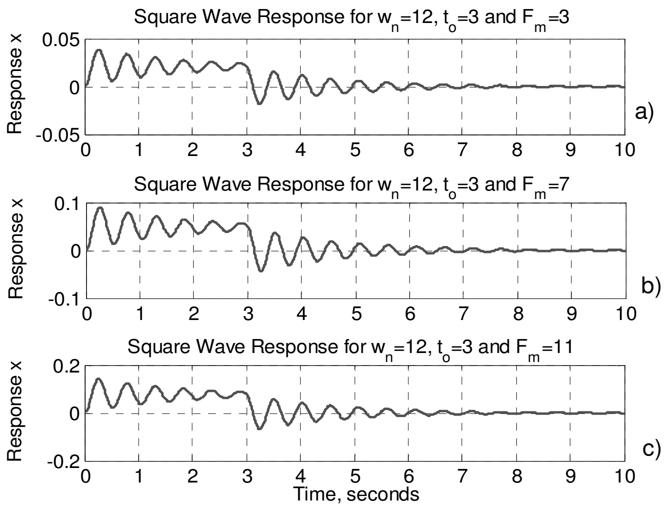

Figure A2.24 Response of SDOF systems to square pulse inputs for different force magnitudes.

Figure A2.25 demonstrates the effects of changing the natural frequency. A transition point occurs when the second step input is added. The sudden shift in vibration characteristics is expected, since we have a piecewise expression for $x(t)$. But while the transition becomes more abrupt as the natural frequency increases, it is never discontinuous. Since the motion of the mass remains continuous, we can infer that the approach is correct; if we had obtained a discontinuity in the motion, we would know the expression is incorrect. This is because a discontinuous expression would imply that the mass moved from one point to another nonadjacent point without passing through the points in between, which is a physically impossible situation.

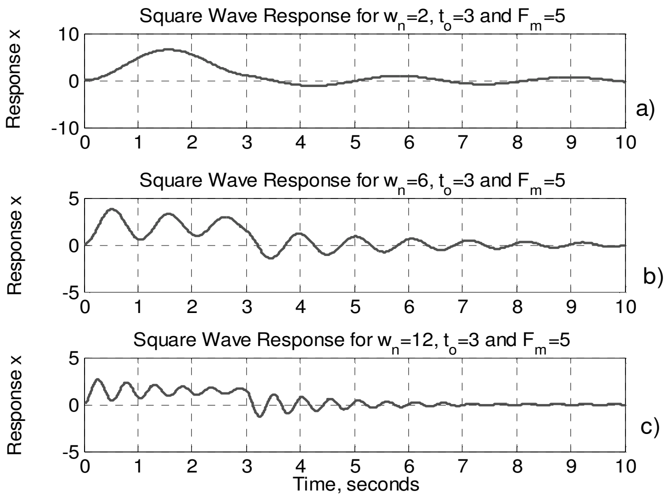

Figure A2.25 Response of SDOF systems to square pulse inputs for different natural frequencies.

Figure A2.26 shows the response behaviour for three different damping ratios. The high damping ratio ($\zeta = 0.3$) causes all of the vibration to be damped out quickly, so that the mass is practically at rest when the second step input is applied. Again, we see that the transient period decreases with increasing damping ratio.

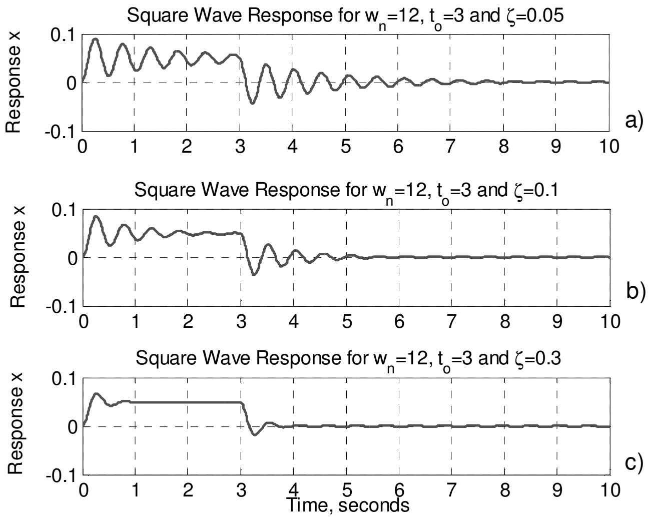

Figure A2.26 Response of SDOF systems to square pulse inputs for different damping ratio.

A2.10 Response of SDOF System to Ramp Input

To examine the response of a SDOF system to ramp input, we must again apply the convolution integral. Assuming that the load is increased uniformly at a rate of *fo* per second and reaches its maximum at time t_d, the expression for the external force is:

$$F(t) = \begin{cases} f_o t & for\ \ 0 \le t < t_d \\ f_o t_d & for\ \ \ \ \ \ t \ge t_d \end{cases} \qquad (A2.62)$$

Substituting the expression for $F(t)$ into the convolution integral yields:

$$x(t) =$$

$$\begin{cases} \dfrac{f_o}{m\omega_d} e^{-\zeta\omega_n t} \displaystyle\int_0^{t_o} \tau e^{\zeta\omega_n \tau} sin\omega_d(t-\tau)d\tau & for\ \ 0 \le t < t_d \\ \dfrac{f_o}{m\omega_d} e^{-\zeta\omega_n t} \left\{ \displaystyle\int_0^{t_d} \tau e^{\zeta\omega_n \tau} sin\omega_d(t-\tau)d\tau + t_d \int_{t_d}^{t} e^{\zeta\omega_n \tau} sin\omega_d(t-\tau)d\tau \right\} & for\ t \ge t_d \end{cases} \qquad (A2.63)$$

In its evaluated form:

$$x(t) = \frac{f_o}{m\omega_d} e^{-\zeta\omega_n t}\left(\frac{1}{(\zeta^2\omega_n^2 + \omega_d^2)^2}\right)\{\omega_d e^{\zeta\omega_n t}[t\zeta^2\omega_n^2 + t\omega_d^2 - 2\zeta\omega_n] + 2\zeta\omega_n\omega_d cos(\omega_d t) + (\zeta^2\omega_n^2 - \omega_d^2)sin(\omega_d t)\};\ for\ 0 \le t < t_d \quad (A2.64)$$

$$x(t) = \frac{f_o}{m\omega_d} e^{-\zeta\omega_n t}\left(\frac{1}{(\zeta^2\omega_n^2 + \omega_d^2)^2}\right)\{\omega_d e^{\zeta\omega_n t}[t\zeta^2\omega_n^2 + t\omega_d^2 - 2\zeta\omega_n] + 2\zeta\omega_n\omega_d cos(\omega_d t) + (\zeta^2\omega_n^2 - \omega_d^2)sin(\omega_d t)\} + \frac{f_o t_d}{k} - \frac{f_o t_d}{k\sqrt{1-\zeta^2}} e^{-\zeta\omega_n(t-t_d)}cos(\omega_d(t-t_d) - \phi);\ for\ t \ge t_d \quad (A2.65)$$

The solution of this expression shows that there is no equilibrium position (as for the step and square wave responses) until after the input has levelled off. This is because the constant that creates the new center point is the result of an integration that does not start at zero, and no such integration exists in this solution until after time t_d.

From Figure A2.27 can be concluded that the transition to the new equilibrium of vibration is discontinuous (as in the step and square wave responses). It seems that the response is nonexistent for the first few seconds, until the load is fully applied, and then begins oscillating, as in the step response. It appears that the ramp response and step response of a single degree of freedom system are similar.

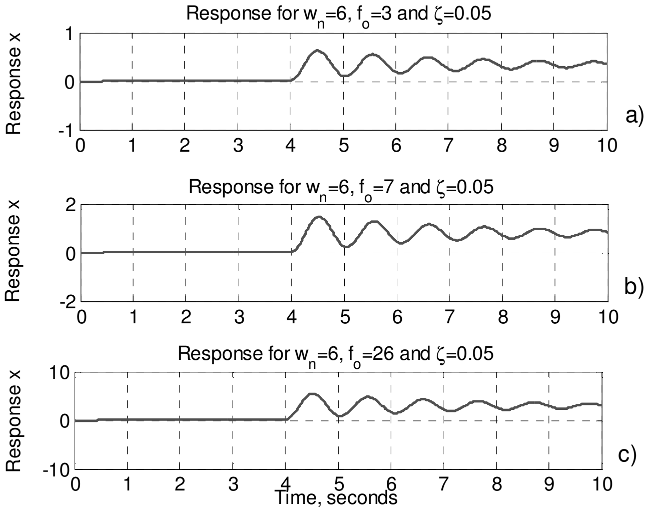

Figure A2.27 Response of SDOF system to Ramp input for different rates of loading.

However, if the response during the transient loading period (Figure A2.28) is observed it can be seen that this is not the case. Actually is quite evident that the system

is oscillating during this period, around a constantly increasing equilibrium. That is, if a line was drawn through the identical point on each period of the sinusoid, the result would be a line of positive slope. This shows that the ramp response is different than the step response; the ramp response has less deflection at the point in time that the full load is applied than the step response.

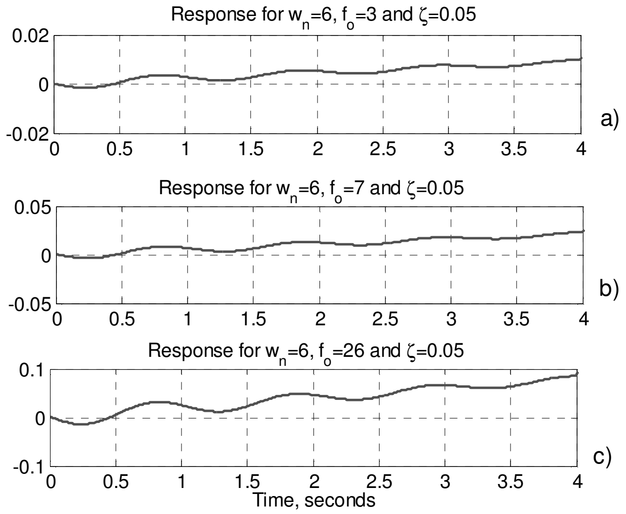

Figure A2.28 Response of SDOF system to Ramp input for different rates of loading – focusing on first few seconds of oscillation, showing that system oscillate during the transient period.

A2.11 Modelling a van der Pol Oscillator

The real-world vibratory systems such as the oscillatory motion of a structure surrounded by a fluid (e.g. structures include the support pylons of offshore oil platforms and antennae attached to the exterior surfaces of aircraft) are looked at next. These structures exhibit vibratory motion due to the creation of vortices in the fluid by viscous interaction between the structure and the particles comprising the fluid. The van der Pol equation can be applied as a model for the motion of such a structure:

$$\ddot{x} + e(x^2 - 1)\dot{x} + x = 0, e > 0 \qquad (A2.66)$$

This equation, given the positive parameter e exhibits the usual form of damping when $|x| < 1$. When $|x| > 1$ the term multiplying first derivative of x will become negative. If equation A2.66 is solved for $\ddot{x}$ then, the damping term would add energy to the system, instead of removing it. This negative damping approximates

some of the phenomena observed in such fluid-structure interactions, and so is an attractive (and necessary) feature of the model.

In order to solve the problem the equation A2.66 need to be integrated analytically over a particular time interval [1]. The only parameters to use here are the parameter *e*, the initial conditions and the time step.

Figure A2.29 and Figure A2.30 shows results for e =0.5, initial conditions x_0=[1; 0]; $x(0)=1$, $x'(0)=0$ and the time interval t_f=30sec. Figure A2.29 shows a simple comparison of displacement and velocity versus time representing oscillatory behaviour. Figure A2.30 represents phase diagram (these diagrams are often used in studies of nonlinear and chaotic systems, since they can clearly show the effects of initial conditions on the response). The key feature of Figure A2.30 is the closed loop.

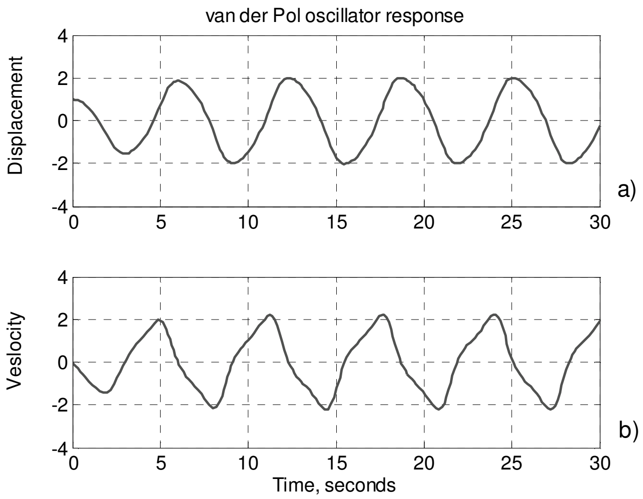

Figure A2.29 Displacement and velocity vs. time for the van der Pol oscillator.

The initial location is denoted by a circle and the final state by a triangle. The closed loop means that the van der Pol system eventually settled down into oscillatory behaviour. It can be proved that a closed loop in phase space corresponds to an oscillating response by considering the function $x = sin\ (t)$. If this (obviously oscillating) function is assumed displacement, then the velocity is described by $y = cos\ (t)$. Plotting this result in phase space, circle is obtained as these functions $x\ (t)$ and $y\ (t)$ are parametric equations for a circle. The van der Pol oscillator's loop is not precisely circular, so it is not periodic in the same regular way as the sine or cosine function. However, it will repeat the same sets of positions and velocities.

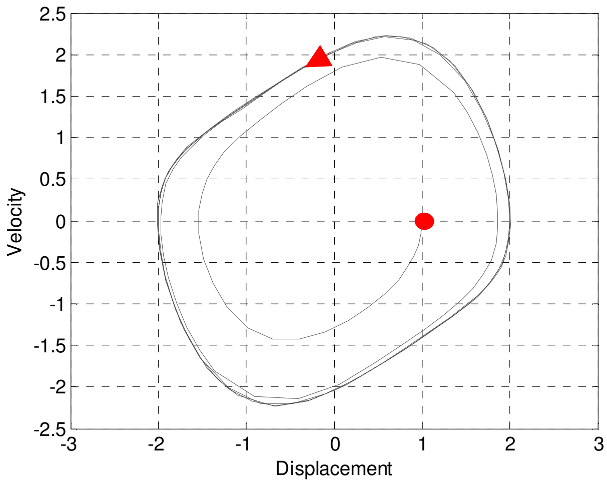

Figure A2.30 Velocity vs. Displacement for van der Pol oscillator.

If different initial conditions are considered the motion will settle into the same limit cycle. Thus, the limit cycle is determined by the parameter *e*; and not by the initial conditions. This limit cycle behaviour is similar to a phenomenon seen in the vibration of structures in a moving fluid; thus, several investigators have used the van der Pol equation to describe these systems [2-4].

A2.12 Response of SDOF System to Random Vibration

Assuming random forcing input as a sinusoidal forcing input of the form:

$$F(t) = A\cos(\omega t + \varphi) \qquad (A2.67)$$

The vast majority of the energy input to the system comes at one particular frequency. For this case, we could then (as a first approximation) treat the forcing frequency as deterministic, and use a random distribution for the amplitude. The uniform and Gaussian distributions are built into Matlab through the <u>rand</u> and <u>randn</u> commands, respectively. If this is applied to damped SDOF system defined by equation:

$$\ddot{x} + 2\zeta\omega_n\dot{x} + \omega_n^2 x = F(t) \qquad (A2.68)$$

where $F(t)$ defined above having random amplitude $A(t)$. It will be convenient to solve for the steady-state response only, neglecting the transient response, which also eliminates the need to specify initial conditions. The analytical solutions can be found the same way as in section A2.5, or the numerical integration routines can be used.

First differential equation at the time $t = t_0$ is solved, and than substitute into the relation the values of the forcing and the forced response at that time, to solve for the initial position and velocity required to match the forced response. As in section A2.5 the complete solution is:

$$x(t) = x_h(t) + x_p(t) \quad (A2.69)$$

$$x_h(t) = A_h e^{-\zeta\omega t} sin(\omega_d t + \theta) \quad (A2.70)$$

$$x_p(t) = A_0(t) cos(\omega t - \phi) \quad (A2.71)$$

where the constants are:

$$A_0 = \frac{A(t)}{\sqrt{(\omega_n^2 - \omega^2) + (2\zeta\omega_n\omega)^2}} \quad (A2.72)$$

$$\phi = tan^{-1}\frac{2\zeta\omega_n\omega}{\omega_n^2 - \omega^2} \quad (A2.73)$$

The next is to specify the initial conditions $x(t_0)$ and $\dot{x}(t_0)$ so that we can have $A_h = 0$, i.e. eliminating transient response. Using the initial value of the force amplitude, specifying $x(t_0) = x_p(t_0)$ and $\dot{x}(t_0) = \dot{x_p}(t_0)$, or

$$x(t_0) = A_0(t_0) cos(\omega t_0 - \phi) \quad (A2.74)$$

$$\dot{x}(t_0) = -\omega A_0(t_0) sin(\omega t_0 - \phi) \quad (A2.75)$$

the coding using Matlab is simplified [1].

Figure A2.31 represents displacement versus time of SDOF system exposed to random vibration. The oscillation remains periodic even with the random forcing amplitude, albeit with an irregular amplitude.

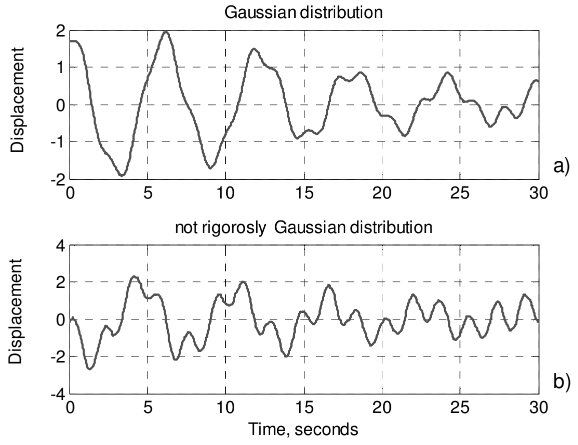

Figure A2.31 Response of SDOF system to Random Vibration

In the top figure it is assumed Gaussian distribution and A is random variable, while the bottom figure represents displacement of the system when the distribution is not rigorously Gaussian as the amplitude is defined between 0 and 5 ($0 < A \leq 5$). In this example natural frequency is set to 1, damping ratio is 0.05 and frequency of input force is 3.5.

A2.13 Randomly-Excited Duffing Oscillator

A nonlinear oscillator is excited with a random forcing. For SDOF system Duffing equation can be written in the form:

$$\ddot{x} + c\dot{x} + kx + \varepsilon g(x) = A\cos\omega t \qquad (A2.76)$$

where the parameters *c* and *k* are assumed to be positive, and $|\varepsilon| \ll 1$ (i.e. ε is not restricted to solely positive nor negative values). This equation allows us to choose the parameter ε and the function $g(x)$ to model nearly-linear springs, for example. Also, this equation retains some attractive quasi-linear qualities. For example if $A = 0$ and $c = 0$ a roughly oscillatory motion for small amplitudes x would be expected; if further introduce a small damping coefficient c; these small-amplitude oscillations would reduce to zero.

Further, function g (x) can be manipulated so that the sign of the parameter ε determines the character of the stiffness element being modelled. For the case where $\varepsilon < 0$ the restoring force will be smaller in extension, and arrive at a soft spring. Conversely, if $\varepsilon > 0$ the spring gains stiffness in extension over the purely linear case, and is called a hard spring.

Also it could be set parameter $\varepsilon > 0$ and select $g(x) = -x^2$, a net restoring force would then be $kx + \varepsilon\, g(x)$ that is negative for $x < 0$ and positive for $x > 0$. In other words, the spring will be soft in extension but hard in compression, meaning that the center of oscillation (equilibrium point) will be shifted slightly away from zero, where the magnitude of the shift depends on the relative values of k and ε.

For the modelling purposes Duffing oscillator response to a harmonic input was looked at. The differential equation solver suite inside Matlab [5] to calculate a numerical solution to a Duffing equation, given the input values of $c = 0.05$; $k = 1$; $\varepsilon = 0.01$; A; and ω. The function used is $g(x) = -x^2$. A random forcing frequency [0, 2] and amplitude [0, 5], which will remain constant for the duration of the oscillation is specified next.

The results for a few default runs are plotted below, along with the random frequency and amplitude used. In Figure A2.33 a random forcing frequency is very close to the system's natural frequency. Hence, we see a nearly-resonant condition in this undamped oscillator. The phase diagram in the same figure corresponds to this run. The response traces out arcs in the phase plane that are circular, but by no means closed.

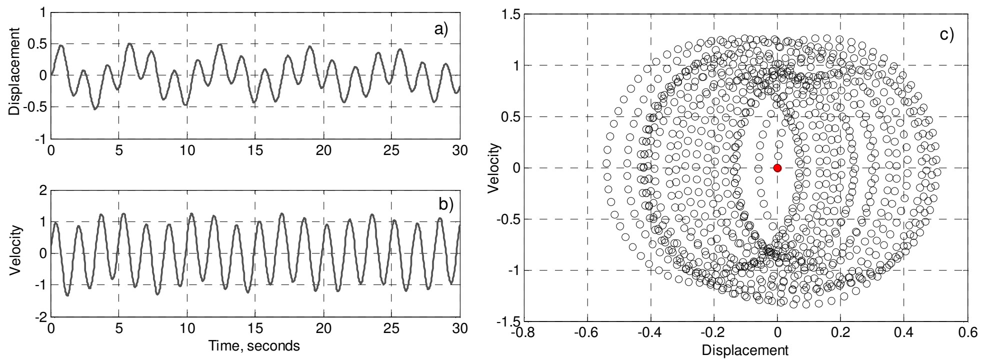

Figure A2.32 Response of the Duffing oscillator to amplitude A = 3.7999 and forcing frequency ω = 3.7960.

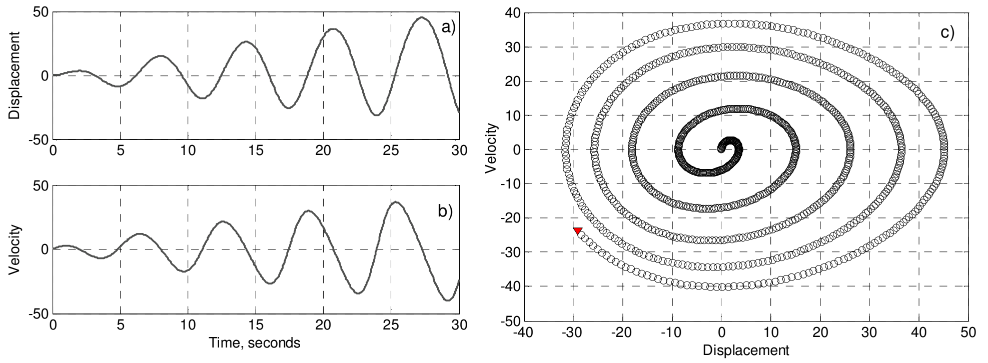

Figure A2.33 Response of the Duffing oscillator to amplitude A = 4.4531 and forcing frequency $\omega = 1.7404$.

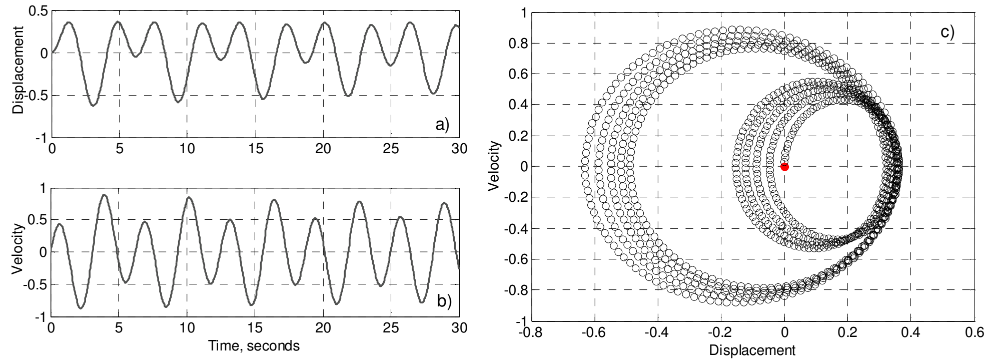

Figure A2.34 Response of the Duffing oscillator to amplitude A = 1.0062 and forcing frequency $\omega = 2.0115$.

References

1. Kuchnicki, S., *Simple Vibration Problems with MATLAB (and Some Help from MAPLE); This document is companion to the text: Mechanical Vibration: Analysis, Uncertainties and Control, by Haym Benaroya and Mark Nagurka, CRC Press 2010*. 2009. p. 165.
2. Akhtar, I., O.A. Marzouk, and A.H. Nayfeh, *A van der Pol--Duffing Oscillator Model of Hydrodynamic Forces on Canonical Structures.* Journal of Computational and Nonlinear Dynamics, 2009. 4(4): p. 041006.
3. Barrón-Meza, M.A., *Vibration Analysis Of a Self-Excited Elastic Beam.* Journal of applied research and technology, 2010. 8: p. 227-238.
4. Ikeda, T. and S. Murakami, *Autoparametric resonances in a structure/fluid interaction system carrying a cylindrical liquid tank.* Journal of Sound and Vibration, 2005. 285: p. 517–546.
5. *MATLAB*. 2004; Available from: http://www.mathworks.co.uk/products/matlab/.
