# Supplementary material for: A comprehensive study of the delay vector variance method for quantification of nonlinearity in dynamical systems
Source: R Soc Open Sci. 2016 Jan 6;3(1):150493. doi: 10.1098/rsos.150493 (PMC4736930; doi:10.1098/rsos.150493)

APPENDIX 3

Delay Vector Variance Method Results for Reference Model

& Discussion on DVV Parameters Choice

A	B	C	D	E	F	G	H	I	J	K	L	M	N	O	P	Q
	SYSTEM	DETAILS	VARIABLES		METHOD 1				METHOD 2				METHOD 3			
					best m	best τ	rmse	RMSE	calc m	τ	rmse	RMSE	calc m	τ	rmse	RMSE
1	SDOF UNDAMPED OSCLATION VARYING MASS	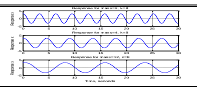	stiffness k=8;<br>initial displacement x0=3;<br>initial velocity v0=1;<br>time duration to test tf=30s;	m=2	5	10	0.0726	0.1672	5	1	0.0109	0.1637	3	1	0.0073	0.1714
				m=4	4	10	0.0452	0.2237	9	1	0.0755	0.1476	3	1	0.0279	0.1729
				m=12	2	7	0.0132	0.1510	11	1	0.0405	0.1580	3	1	0.0058	0.1764
2	DAMPED SDOF SYSTEM OSCELLATION FOR VARYING DAMPING VALUES (only for underdamped case xi<1)	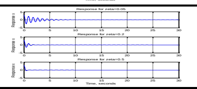	natural frequency wn=7;<br>initial displacement x0=3;<br>initial velocity v0=1;<br>time duration to test tf=30s;	ζ=0.05	10	10	0.6891	0.4716	11	1	0.1921	0.3570	3	1	0.0268	0.3465
				ζ=0.2	10	9	0.8662	0.5372	23	1	0.3338	0.6046	3	1	0.0967	0.6207
				ζ=0.5	2	9	1.0195	0.5799	21	1	0.2961	0.6309	3	1	0.1076	0.6818
3	OVERDAMPED SDOF SYSTEM OSCILATION FOR VARYING DAMPING VALUES<br>(only for overdamped case xi>1))!!!	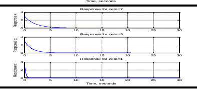	natural frequency wn=7;<br>initial displacement x0=3;<br>initial velocity v0=1;<br>time duration to test tf=30s;	ζ=7	5	10	0.6491	0.4364	18	1	0.2885	0.3296	3	1	0.0486	0.2918
				ζ=5	2	1	0.0313	0.4253	24	1	0.4093	0.4717	3	1	0.0742	0.4349
				ζ=1	3	10	0.8726	0.5464	22	1	0.2759	0.6457	3	1	0.0927	0.6995
4	HARMONIC EXCITATION OF UNDAMPED SDOF SYSTEMS<br>effect of varying driving frequency w for a given natural frequency wn	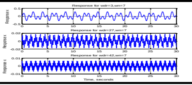	natural frequency wn=7;<br>initial displacement x0=0;<br>initial velocity v0=0;<br>force magnitude per unit mass f0=6;<br>time duration to test tf=30s;	wdr=3	4	10	0.2300	0.3334	5	1	0.0061	0.1073	3	1	0.0028	0.1185
				wdr=27	6	8	0.0365	0.2433	19	1	0.0238	0.2103	3	1	0.0108	0.2143
				wdr=42	5	10	0.0682	0.2271	10	1	0.0606	0.2832	3	1	0.0736	0.3205
5	HARMONIC EXCITATION OF UNDAMPED SDOF SYSTEMS<br>effect of varying natural frequency wn for a given<br>driving frequency w	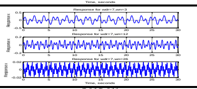	driving frequency wdr=7;<br>initial displacement x0=0;<br>initial velocity v0=0;<br>force magnitude per unit mass f0=6;<br>time duration to test tf=30s;	wn=3	4	10	0.2300	0.3334	4	1	0.0042	0.1071	3	1	0.0034	0.1176
				wn=12	5	7	0.0627	0.2938	3	1	0.1336	0.1416	3	1	0.1283	0.1406
				wn=26	6	9	0.0617	0.2581	17	1	0.0388	0.2256	3	1	0.0170	0.2037
6	HARMONIC EXCITATION OF UNDAMPED SDOF SYSTEMS<br>BEAT Phenomenon natural and driving frequencies are close but not equal	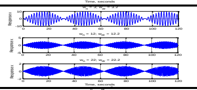	initial displacement x0=0;<br>initial velocity v0=0;<br>force magnitude per unit mass f0=6;<br>time duration to test tf=120s;	wn=3; wdr=3.2;	3	1	0.1314	0.1759	3	1	0.0046	0.1369	3	1	0.0037	0.1366
				wn=12; wdr=12.2;	6	10	0.1740	0.2360	2	1	0.0068	0.1337	3	1	0.0094	0.1682
				wn=22; wdr=22.2;	3	6	0.0150	0.1044	3	1	0.1248	0.2167	3	1	0.1260	0.2172
7	HARMONIC EXCITATION OF UNDAMPED SDOF SYSTEMS<br>RESONANCE natural and driving frequencies are equal	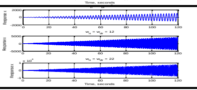	initial displacement x0=0;<br>initial velocity v0=0;<br>force magnitude per unit mass f0=6;<br>time duration to test tf=120s	wn=wdr=3;	4	9	0.1462	0.1205	7	1	0.0165	0.1439	3	1	0.0096	0.1190
				wn=wdr=12;	7	10	0.0613	0.1334	3	1	0.0027	0.1461	3	1	0.0035	0.1462
				wn=wdr=22;	4	6	0.0194	0.1176	6	1	0.0044	0.1199	3	1	0.0041	0.1646
8	HARMONIC EXCITATION OF DAMPED SDOF SYSTEMS (VARYING DAMPING VALUES)	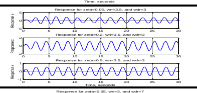	driving frequency wdr =3;<br>natural frequency wn=3.5;<br>force magnitude per unit mass f0=6;<br>time duration to test tf=30s;	ζ=0.05	5	10	0.1243	0.2020	4	1	0.0039	0.1476	3	1	0.0052	0.1550
				ζ=0.2	7	10	0.0550	0.1791	3	1	0.0030	0.1617	3	1	0.0048	0.1620
				ζ=0.5	6	10	0.2053	0.2243	4	1	0.0044	0.1615	3	1	0.0082	0.1659
9	HARMONIC EXCITATION OF DAMPED SDOF SYSTEMS VARYNG NATURAL FREQUENCY	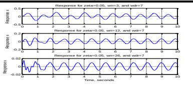	driving frequency wdr =7;<br>damping ratio ζ=0.05;<br>force magnitude per unit mass f0=6;<br>time duration to test tf=30s;	wn=3	4	1	0.0403	0.1326	1	1	0.0110	0.1507	3	1	0.0312	0.1383
				wn=12	3	1	0.0029	0.1613	6	1	0.0075	0.1496	3	1	0.0059	0.1625
				wn=26	4	10	0.2174	0.2077	4	1	0.0034	0.1604	3	1	0.0054	0.1648
10	BASE EXCITATION OF SDOF SYSTEMS VARYING EXCITATION FREQUENCY	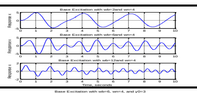	base excitation magnitude y0=3;<br>damping ratio ζ=0.05;<br>natural frequency wn=4<br>time duration to test tf=10s;	wb=2	4	1	0.0125	0.1600	19	1	0.0159	0.1489	3	1	0.0053	0.1651
				wb=6	4	1	0.0415	0.1359	1	1	0.0029	0.1548	3	1	0.0043	0.1509
				wb=12	3	1	0.0049	0.1604	1	1	0.0074	0.1765	3	1	0.0101	0.1661
11	BASE EXCITATION OF SDOF SYSTEMS VARYING BASE EXCITATION MAGNITUDE	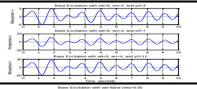	base excitation frequency wb=6;<br>damping ratio ζ=0.05;<br>natural frequency wn=4<br>time duration to test tf=10s;	y0=3	4	1	0.0415	0.1359	1	1	0.0050	0.1567	3	1	0.0018	0.1509
				y0=7	4	1	0.0415	0.1359	1	1	0.0051	0.1571	3	1	0.0018	0.1502
				y0=11	4	1	0.0405	0.1359	1	1	0.0072	0.1543	3	1	0.0028	0.1509
12	BASE EXCITATION OF SDOF SYSTEMS VARYING DAMPING RATIO	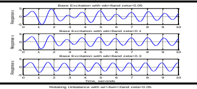	base amplitude y0=3;<br>base excitation frequency wb=6;<br>natural frequency wn=4<br>time duration to test tf=10s;	ζ=0.05	4	1	0.0415	0.1359	1	1	0.0050	0.1567	3	1	0.0021	0.1511
				ζ=0.1	4	1	0.0412	0.1399	10	1	0.0061	0.1266	3	1	0.0023	0.1561
				ζ=0.3	4	1	0.0371	0.1557	7	1	0.0050	0.1576	3	1	0.0062	0.1717
13	SDOF SYSTEM WITH A ROTATING UNBALANCE VARYING NATURAL FREQUENCY	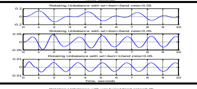	rotating mass mo=3;<br>sdof mass m=7;<br>angular velocity of rot mass wr=4; damping ratio ζ=0.05;<br>time duration to test tf=10s;<br>constant e=0.1	wn=2	3	1	0.0079	0.1642	1	1	0.0057	0.1695	3	1	0.0036	0.1702
				wn=6	4	1	0.0224	0.1575	12	1	0.0151	0.1450	3	1	0.0042	0.1661
				wn=12	4	1	0.0064	0.1654	6	1	0.0047	0.1624	3	1	0.0051	0.1681
14	SDOF SYSTEM WITH A ROTATING UNBALANCE VARYING DAMPING	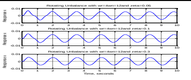	rotating mass mo=3;<br>sdof mass m=7;<br>angular velocity of rot mass wr=4; natural fequency wn=12;<br>time duration to test tf=10s;<br>constant e=0.1	ζ=0.05	4	1	0.0064	0.1654	9	1	0.0076	0.1568	3	1	0.0097	0.1695
				ζ=0.1	4	1	0.0207	0.1642	8	1	0.0104	0.1636	3	1	0.0049	0.1737
				ζ=0.3	4	2	0.0531	0.1506	8	1	0.0068	0.1665	3	1	0.0083	0.1746
15	SDOF SYSTEM WITH A ROTATING UNBALANCE VARYING MASS	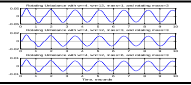	rotating mass mo=3;<br>damping ratio ζ=0.05;<br>angular velocity of rot mass wr=4; natural fequency wn=12;<br>time duration to test tf=10s;<br>constant e=0.1	m=1	4	1	0.0152	0.1595	7	1	0.0056	0.1582	3	1	0.0043	0.1673
				m=3	4	1	0.0202	0.1595	8	1	0.0089	0.1607	3	1	0.0026	0.1674
				m=6	4	1	0.0202	0.1595	7	1	0.0058	0.1599	3	1	0.0026	0.1678
16	SDOF-STEP RESPONSE VARYING FORCE MAGNITUDE	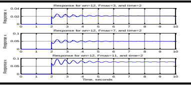	initial time to=2;<br>damping ratio ζ=0.05;<br>natural fequency wn=12;<br>time duration to test tf=10s;<br>system mass m=1;	Fm=3	3	1	0.1690	0.3004	1	1	0.0256	0.3678	3	1	0.0364	0.3647
				Fm=7	3	1	0.1690	0.3004	1	1	0.0585	0.3636	3	1	0.0296	0.3588
				Fm=11	3	1	0.0364	0.3533	1	1	0.0265	0.3645	3	1	0.0592	0.3647
17	SDOF-STEP RESPONSE VARYING NATURAL FREQUENCY	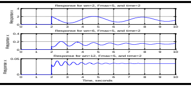	system mass m=1;<br>damping ratio ζ=0.05;<br>natural fequency wn=12;<br>time duration to test tf=10s;<br>force magnitude Fm=5;<br>initial time to=2;	wn=2	3	1	0.0488	0.1475	2	1	0.0110	0.1628	3	1	0.0105	0.1626
				wn=6	3	1	0.0865	0.2209	1	1	0.0193	0.2567	3	1	0.0168	0.2534
				wn=12	3	1	0.1690	0.3004	1	1	0.0317	0.3662	3	1	0.0489	0.3632
18	SDOF-STEP RESPONSE VARYING DAMPING RATIO	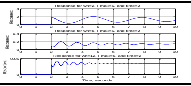	system mass m=1;<br>natural fequency wn=12;<br>time duration to test tf=10s;<br>force magnitude Fm=5;<br>initial time to=2;	ζ=0.05	3	1	0.1690	0.3004	1	1	0.0347	0.3757	3	1	0.0833	0.3732
				ζ=0.1	2	1	0.0353	0.4032	19	1	0.0881	0.3670	3	1	0.1318	0.4179
				ζ=0.3	2	1	0.0512	0.4191	6	1	0.1731	0.4233	3	1	0.0320	0.4253
19	SDOF SQUARE PULSE INPUTS VARYING NATURAL FREQUENCY	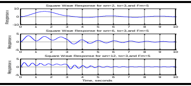	system mass m=1;<br>damping ratio ζ=0.05;<br>time duration to test tf=10s;<br>force magnitude Fm=5;<br>wave starts at to=0;<br>wave stops at to=3;	wn=2	4	1	0.0311	0.2394	3	1	0.0134	0.2502	3	1	0.01732	0.2490
				wn=6	6	10	0.2304	0.1718	1	1	0.0086	0.1917	3	1	0.00582	0.1903
				wn=12	9	10	0.4009	0.2187	4	1	0.0179	0.1875	3	1	0.01004	0.1876
20	SDOF SQUARE PULSE INPUTS VARYING DAMPING RATIO	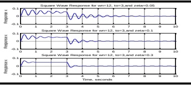	system mass m=1;<br>natural frequency wn=12;<br>time duration to test tf=10s;<br>force magnitude Fm=7;<br>wave starts at to=0;<br>wave stops at to=3;	ζ=0.05	9	10	0.4009	0.2187	19	1	0.0359	0.1428	3	1	0.0095	0.1886
				ζ=0.1	10	10	0.4237	0.2176	3	1	0.0247	0.2544	3	1	0.0236	0.2544
				ζ=0.3	5	5	0.1207	0.2798	10	1	0.0270	0.3248	3	1	0.0085	0.3409
21	SDOF SQUARE PULSE INPUTS VARYING FORCE MAGNITUDE	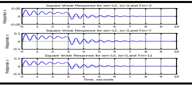	system mass m=1;<br>natural frequency wn=12;<br>time duration to test tf=10s;<br>damping ratio ζ=0.05;<br>wave starts at to=0;<br>wave stops at to=3;	Fm=3	9	10	0.4009	0.2187	5	1	0.0051	0.1798	3	1	0.0074	0.1882
				Fm=7	9	10	0.4009	0.2187	3	1	0.0053	0.1906	3	1	0.0052	0.1892
				Fm=11	9	10	0.4009	0.2187	3	1	0.0130	0.1908	3	1	0.0096	0.1880
22	SDOF RAMP INPUT VARYING RATE OF LOADING	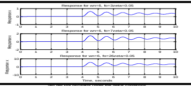	system mass m=1;<br>natural frequency wn=6;<br>k=wn^2;<br>wd=wn*sqrt(1-zeta^2);<br>damping ratio ζ=0.05;<br>force starts at to=0;<br>force levels at at te=4;	fo=3	5	1	0.0442	0.1861	11	1	0.0183	0.1864	3	1	0.0101	0.1982
				fo=7	5	1	0.0442	0.1861	4	1	0.0107	0.1978	3	1	0.0062	0.1977
				fo=26	5	1	0.0442	0.1861	6	1	0.0069	0.1932	3	1	0.0073	0.1980
23	VAN DER POL OSCILLATOR	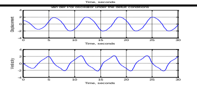	e =0.5;<br>initial conditions<br>x0=[1; 0]; x(0)=1, x'(0)=0<br>time interval tf=30;	disp	2	1	0.0063	0.1667	3	1	0.0078	0.1534	3	1	0.0113	0.1554
				vel	2	1	0.1088	0.1082	1	1	0.0804	0.1218	3	1	0.1344	0.1136

	A	B	C	D	E	F	G	H	I	J	K	L	M	N	O	P	Q
73		SYSTEM	DETAILS	VARIABLES		METHOD 1				METHOD 2				METHOD 3			
74						best m	best τ	rmse	RMSE	calc m	τ	rmse	RMSE	calc m	τ	rmse	RMSE
75	24	SDOF RANDOM VIBRATION	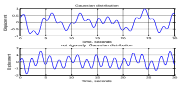	A is a random variable; distflag should be zero for uniform, and 1 for Gaussian distribution	case 1	4	1	0.0084	0.1541	18	1	0.0418	0.1220	3	1	0.0070	0.1556
76				A=[0 to 5] for the uniform distribution, and as a distribution with mean of 2.5 for the Gaussian (won't be rigorously Gaussian).	case 2	2	1	0.0015	0.1560	1	1	0.0224	0.1534	3	1	0.0366	0.1391
77	25	SDOF RANDOMLY EXCITED DUFFING OSCILLATOR	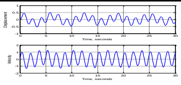	tspan=[0 30]; xinit=[0 0]'; e=0.01; c=0.05; k=1; A=3.7999; w=3.7960; chosen to prevent resonance	dis	3	1	0.0523	0.1252	1	1	0.0187	0.1435	3	1	0.0545	0.1252
78					vel	4	1	0.0910	0.1316	10	1	0.1201	0.1712	3	1	0.0703	0.1365
79	26	SDOF RANDOMLY EXCITED DUFFING OSCILLATOR	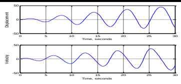	tspan=[0 30]; xinit=[0 0]'; e=0.01; c=0.05; k=1; A=4.4351; w=1.7404; chosen to prevent resonance	dis	6	1	0.0332	0.1452	16	1	0.0106	0.1342	3	1	0.0090	0.1573
80					vel	5	1	0.0266	0.1450	20	1	0.0965	0.1187	3	1	0.0269	0.1548
81	27	SDOF RANDOMLY EXCITED DUFFING OSCILLATOR	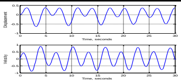	tspan=[0 30]; xinit=[0 0]'; e=0.01; c=0.05; k=1; A=1.0062; w=2.0115; chosen to prevent resonance	dis	4	1	0.0376	0.1507	1	1	0.0153	0.1656	3	1	0.0379	0.1579
82					vel	3	1	0.0245	0.1484	10	1	0.0079	0.1318	3	1	0.0035	0.1560

A3.1 SDOF Undamped Oscillation

A3.1.1 SDOF Undamped Oscillation – varying mass

An undamped SDOF system undergoing free vibration was considered first for three different masses (2, 4 and 12 kg). Using three different approaches to determine embedding parameters resulted in slightly different RMSE values in the case of low and high system masses. The highest deviation is observed for the mass of the system of 4kg, where RMSE is the highest using Method 1 and the lowest using Method 2. The calculated RMSE values using Method 2 and Method 3 approach give similar answers for all masses. In the case of Method 1 and Method 2 there is no visible trend in results while RMSE calculated using Method 3 shows slight increase when mass of the system increases. Overall, the RMSE of DVV scatter plot from the bisector line is insensitive to changing the mass of the system and has an average value of 0.181; 0.156 and 0.174 for Method 1, Method 2 and Method 3 respectively.

A3.2 A Damped SDOF System

The effects of the increasing viscous damping ratio (ζ = 0.05, 0.2 and 0.5) on an underdamped SDOF system was investigated next. As the damping ratio increases, the response of the system dies out quite quickly, while RMSE increases in all three approaches. With Method 1, the increase is almost linear, while with two other methods it is steeper when damping increases between 0.05 and 0.2 than between 0.2 and 0.5. In general Method 2 and Method 3 give similar results, especially for damping ratios lower than 0.2. RMSE for Method 2 varies between 0.357 and 0.631, while in the case of Method 3 it is between 0.347 and 0.682 for damping ratio ζ = 0.05 and 0.5 respectively.

A3.3 Overdamped SDOF Oscillation

For overdamped systems, decreasing viscous damping ratio (ζ = 7, 5 and 1) as expected, lead to critically damped quicker dying out of responses. The RMSE in the case of Method 1 does not have clear trend for higher values of damping, but as the system damping ratio decreases to ζ = 1, RMSE increases. The results obtained using Method 2 and Method 3 are similar, except that for an overdamped case the RMSE increases as damping decreases. RMSE for the Method 2 varies between 0.330 and 0.646, while in the case of the Method 3 it is between 0.292 and 0.699 for damping ratio ζ = 7 and 1, respectively. For the damped SDOF system in all three cases, underdamped (ζ < 1), critically damped (ζ = 1), or overdamped (ζ > 1), the initial conditions are assumed to be the same.

Appendix 2 provided as a supplementary material with this paper presents all the dynamic responses along with the details of parameters for each response. The system response dies out very quickly and consequently DVV plots do not converge to unity even with increased span parameter n_d. The result is that the response of the system shows nonlinearity. The nonlinearity increases with increasing damping for underdamped case and with decreasing damping for overdamped case, giving the highest value of RMSE for critically damped case. The results of this analysis also show that the measurements of system response obtained close to a nearly static condition could strongly influence the DVV analysis.

A3.4 Harmonic Excitation of Undamped SDOF Systems

A harmonic load is imposed on the undamped SDOF system next and the natural and driving frequencies are observed keeping the initial conditions the same. The natural frequency is set to 7 Hz, while the driving frequency assumes values 3, 27 and 42 Hz. While with increasing driving frequency RMSE decreases when the Method 1 is applied, RMSE increases for the Method 2 and Method 3. The RMSE values for the latter two are very close to each other, for Method 2 the values being 0.107, 0.210 and 0.283 and for Method 3, 0.118, 0.214 and 0.320 for driving

frequencies 3, 27 and 42 Hz, respectively. Similar results are obtained when the driving frequency is set to constant value of 7 Hz and natural frequency increased adopting the values 3, 12 and 26 Hz. The values are decreasing for Method 1 and increasing for Method 2 and Method 3. RMSE values for Method 2 are 0.107 and 0.226 here and for Method 3, 0.118 and 0.204 for natural frequencies 3 and 26 Hz respectively.

Since the response of the system analysed depends on the driving-natural frequency ratio, phenomena of beats and resonance were investigated next. For beats, the natural frequency and the driving frequency are close but not equal. Pairwise, these correspond to 3 and 3.2 Hz, 12 and 12.2 Hz and 22 and 22.2 Hz for simulations in this paper. The results of DVV analysis of the dynamic response shows that a visible trend in RMSE values is obtained only with Method 3, i.e. RMSE increases with increasing frequency from 0.137 to 2.217. Also, according to the DVV plots this type of response is less linear than the responses of the same system discussed above.

For resonance, the driving and natural frequencies are set equal (3, 12 and 22 Hz respectively). RMSE calculated using only Method 3 shows a consistent trend, i.e. with increasing frequency it increases from 0.119 to 0.165 for frequencies 3 and 22 Hz, respectively. Overall, for response of the undamped SDOF system excited with harmonic load with increasing natural frequency, or driving frequency, or both (regardless if it results in the beats or resonance) the results of the DVV analysis show that RMSE obtained using Method 3 increase. Furthermore, the DVV analysis of the beats shows greater nonlinearity than other cases.

A3.5 Harmonic Excitation of Damped SDOF Systems

The response of the SDOF System under harmonic forcing was analysed next when the damping ratio ζ was varied and the natural and the driving frequencies were kept constant. The transient period of vibration varies inversely with damping ratio and damping ratio affects the amplitude of the steady-state vibration. The value of RMSE calculated using Method 1 does not show a distinctive trend, while it increases in the case of Method 2 and Method 3. For example, the amplitude of the response for ζ = 0.05 is almost 2 and RMSE is 0.155, while that for ζ = 0.5 is less than 1 and

RMSE is 0.166, using Method 3. Studying the same system, keeping the damping constant while changing the natural frequency (3, 12 and 26 Hz) it is found that amplitude of response decreases while RMSE increases, taking values: 0.138, 0.163 and 0.165 with Method 3 and 0.133, 0.161 and 0.208 with Method 1. For higher frequencies, RMSE obtained differs greatly between these two methods.

A3.6 Base Excitation of SDOF Systems

The effects of changing the excitation (base) frequency (2, 6 and 12 Hz) on system response while keeping all other parameters constant was looked at first. The results obtained using Method 1 and Method 3 are very close in value but do not conform to a specific trend, while RMSE calculated using Method 2 increases with base frequency increase. The results obtained with Method 3 indicate that the linearity of the signal is almost unaffected by change of base frequency, with average value of RMSE = 0.161.

By increasing input amplitude (y_0 = 3, 7 and 11) and keeping the rest of parameters constant, the maximum amplitude of the overall vibration and of the steady-state response both increase. While results of DVV analysis differ slightly between the methods, they remain almost constant for increase in amplitude. This would mean that increase in amplitude does not affect the linearity of the system. The average values of RMSE are 0.1359, 0.1560 and 0.1507 for Method 1, Method 2 and Method 3 respectively.

The changes of damping (ζ = 0.05, 0.1 and 0.3) as in previous cases of increasing damping ratio lead to increase in RMSE value of the Method 1 and Method 3, hence nonlinearity estimates of the response signal increases. The values range from 0.136 to 0.156 and from 0.151 to 0.172 for Method 1 and 3, respectively. The results obtained with Method 3 are similar in value with other two, but do not show consistent trend.

A3.7 SDOF Systems with a Rotating Unbalance

The natural frequency of SDOF system with a rotating imbalance was varied (2, 6 and 12 Hz) while keeping all other parameters constant. The response of the system is analysed using DVV method. The results show that there is no trend in the RMSE obtained with any of approaches proposed, similar to the previous cases with increased natural frequency. But with a closer look at the results it could be concluded that that RMSE derived using the Method 1 and 3 are close in value and almost constant for all natural frequencies considered. Their average values are 0.162 and 0.168 for the Method 1 and 3, respectively.

For the increasing damping of the system (ζ = 0.05, 0.1 and 0.3) DVV analysis gives increasing values of RMSE when applying the Method 2 and 3. The results are 0.157, 0.164 and 0.167 for the 2nd and 0.170, 0.174 and 0.175 for the 3rd Method.

Finally, the variation of vibration with increasing system mass shows that the amplitude of the vibration decreases with increasing mass (1, 3, 6 kg), but there is no reflection on RMSE value calculated for system response. Moreover, the RMSE values are constant, i.e. the response of the system does not change its degree of linearity, when Method 1 (0.160) and Method 3 (0.167) are considered.

A3.8 Step Response of SDOF System

The step responses of SDOF system, when varying the natural frequency, result in two changes in the response. First, the rate of exponential decrease in the response (the effect of damping) is accentuated; that is, the response stabilizes more quickly and the oscillation frequency decreases, since the natural frequency also dictates the damped frequency. When applying the DVV analysis on system response signal it is evident that RMSE increases with natural frequency (2 to 12 Hz) for all three methods, from 0.148 to 0.300, 0.163 to 0.366 and from 0.163 to 0.363 for Method 1, Method 2 and Method 3.

With increasing damping ratio (ζ = 0.05, 0.1 and 0.3), the amount of time to damp out all vibration decreases, while RMSE increases taking the values 0.300, 0.403 and 0.419 when Method 1 and 0.373, 0.418 and 0.425 when Method 3 is applied.

A3.9 Response of SDOF System to Square Pulse Inputs

A single pulse of constant magnitude and finite duration is considered.

When natural frequency is increased (2, 6 and 12 Hz), the deviation from bisector line of DVV scatter plots decreases in the cases of the Method 2 and the Method 3 resulting in RMSE values of 0.250, 0.192 and 0.188, and 0.249, 0.190 and 0.188 for the three frequencies, respectively. The results obtained with the two approaches are almost the same and represent the only case, up to now, that RMSE decreases with increasing natural frequency.

On the other hand, when damping coefficient increases (ζ = 0.05, 0.1 and 0.3) the deviation from the bisector line increases, as it was the case with all underdamped cases before. The RMSE varies between 0.143 and 0.325 for the 2nd and between 0.189 and 0.341 for the 3rd Method respectively.

When force magnitude varies (F_0 = 3, 7 and 11) the deviation from the bisector line of DVV scatter plots remains unchanged resulting in constant value of RMSE in all three approaches (0.219; 0.191 and 0.188 for Method 1; Method 2 and Method 3).

A3.10 Response of SDOF System to Ramp Input

The response of an SDOF system to ramp input shows that there is no equilibrium position (as for the step and square wave responses) until after the input is absent. With increasing initial amplitude (f_0 = 3, 7 and 26) and keeping the natural frequency and damping constant the RMSE calculated remains constant, 0.186 and 0.198 for Method 1 and Method 3.

A3.11 Modelling a van der Pol Oscillator

Displacement and velocity responses of a Van der Pol oscillator for initial conditions $x(0) = 1$, $\dot{x}(0) = 0$, the constant of the equation, e = 0.5 and the time of response considered t_f = 30sec shows that the deviation from bisector line obtained from three approaches are very close in the value. For displacement responses, the RMSE values are 0.167, 0.153 and 0.155, while for velocity it is 0.108, 0.122 and 0.114 for Method 1, Method 2 and Method 3 respectively. Note that the velocity related values are lower than those for displacement in all three cases.

A3.12 Response of SDOF System to Random Vibration

The displacement of the SDOF system excited by random vibration was analysed next. Cases of a Gaussian distribution and a distribution not rigorously Gaussian are both compared as the amplitude is defined between 0 and 5 ($0 < A \leq 5$) where A is a random variable. For the first approximation the initial value of: natural frequency is set to 1 Hz, the damping ratio is 0.05 and frequency of input force is 3.5 Hz. The RMSE value for the first case is 0.154, 0.122 and 0.156, while for the second case it is 0.156, 0.153 and 0.139 for Method 1, Method 2 and Method 3, respectively. The results show that Method 1 is insensitive to change of amplitude, while it increases for Method 2 but decreases for Method 3.

A3.13 Randomly-Excited Duffing Oscillator

The response of a Duffing oscillator is considered for a harmonic input $g(x) = -x^2$ when the input values are $c = 0.05$; $k = 1$; $\varepsilon = 0.01$ where c is the viscous damping and ε corresponds to a constant coefficient scaling the nonlinear term in stiffness. The parameters A and ω were varied. For A=3.7999 and ω =3.7960 Hz the RMSE is found to give almost the same results for displacement and velocity signals when using Method 1 and Method 3. For displacement responses, the RMSE values are 0.125 for

both approaches and for velocity they are 0.132 and 0.136 for Method 1 and Method 3 respectively. By choosing A=4.4351 and ω =1.7404 Hz, when the system is in nearly-resonant condition, the RMSE is the same value 0.145 for displacement and velocity when applying Method 1. For the Method 3, the values are still very close with 0.157 for displacement response and 0.155 for velocity. For A=1.0062 and ω =2.0115 Hz, the RMSE is found to give almost the same results for displacement and velocity signals when using Method 1 and Method 3. For displacement, they are 0.151 and 0.158 and for velocity they are 0.148 and 0.156 for Method 1 and Method 3, respectively.

A3.2 DVV Parameter Choice

The analysis of the results obtained using three different methods to determine the optimal parameters for DVV analysis is discussed in previous chapters. It is concluded that Method 3, keeping m=3 and τ=1, shows the best consistency in interpretation of DVV method results when SDOF parameter changes.

	SYSTEM	DETAILS	VARIABLES		METHOD 1				METHOD 2				METHOD 3			
				mass:	best m	best τ	rmse	RMSE	calc m	τ	rmse	RMSE	set m	set τ	rmse	RMSE
1	SDOF UNDAMPED OSCILATION VARYING MASS	SDOF Undamped Oscillation mx"+kx=0; x(t) = Asin(ωnt + φ)	stiffness k=8; initial displacement x0=3; initial velocity v0=1; time duration to test tf=30s;	m=2	5	10	0.0726	0.1672	5	1	0.0109	0.1637	3	1	0.0073	0.1714
				m=4	4	10	0.0452	0.2237	9	1	0.0755	0.1476	3	1	0.0279	0.1729
				m=12	2	7	0.0132	0.1510	11	1	0.0405	0.1580	3	1	0.0058	0.1764

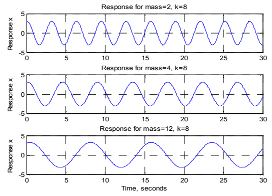

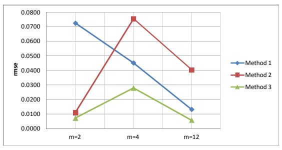

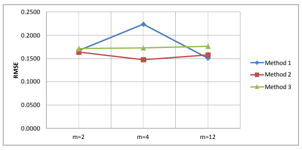

m=2 m=4 m=12

METHOD 1

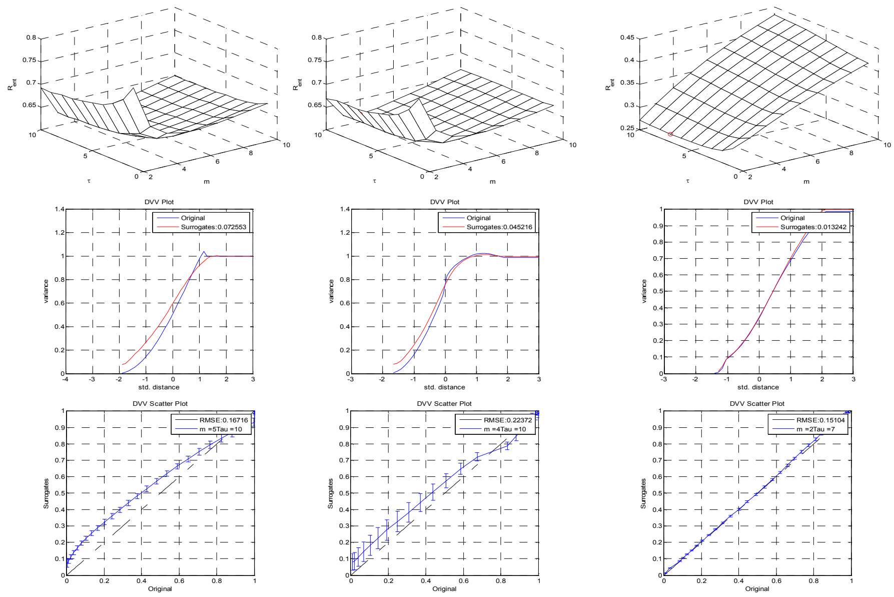

m=2 m=4 m=12

METHOD 2

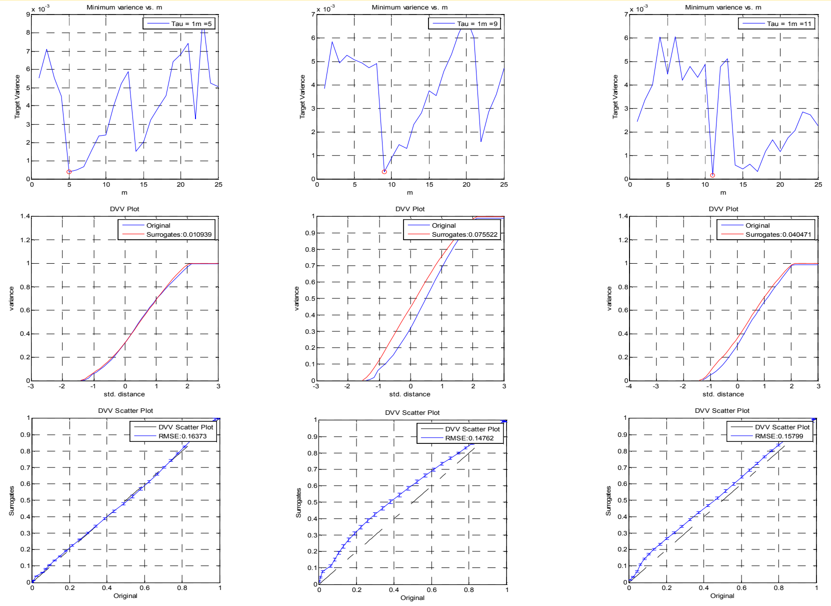

m=2 m=4 m=12

METHOD 3

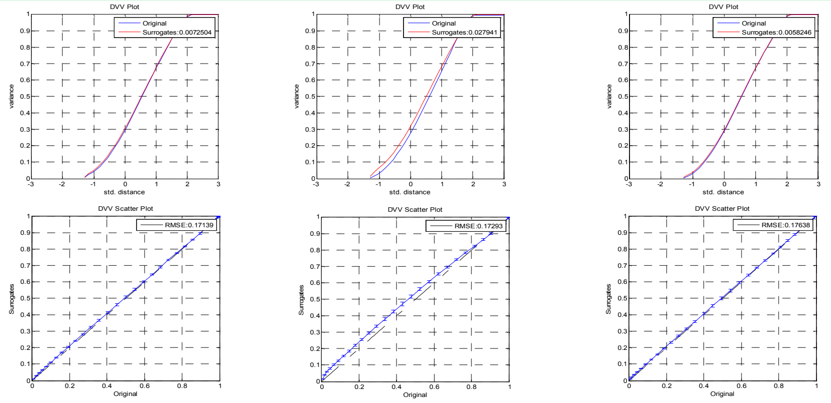

	SYSTEM	DETAILS	VARIABLES		METHOD 1				METHOD 2				METHOD 3			
				mass:	best m	best τ	rmse	RMSE	calc m	τ	rmse	RMSE	set m	set τ	rmse	RMSE
2	DAMPED SDOF SYSTEM OSCILATION FOR VARYING DAMPING VALUES (only for underdamped case xi<1)	SDOF Damped Oscillation mx''+cx'+kx=0	natural frequency wn=7; initial displacement x0=3; initial velocity v0=1; time duration to test tf=30s;	ζ=0.05	10	10	0.6891	0.4716	11	1	0.1921	0.3570	3	1	0.0268	0.3465
				ζ=0.2	10	9	0.8662	0.5372	23	1	0.3338	0.6046	3	1	0.0967	0.6207
				ζ=0.5	2	9	1.0195	0.5799	21	1	0.2961	0.6309	3	1	0.1076	0.6818

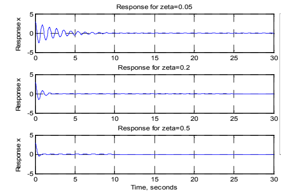

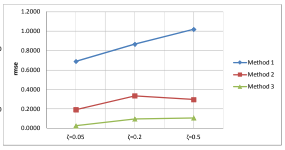

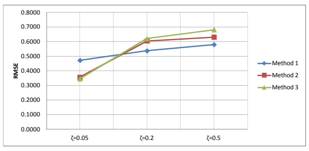

ζ=0.05 ζ=0.2 ζ=0.5

METHOD 1

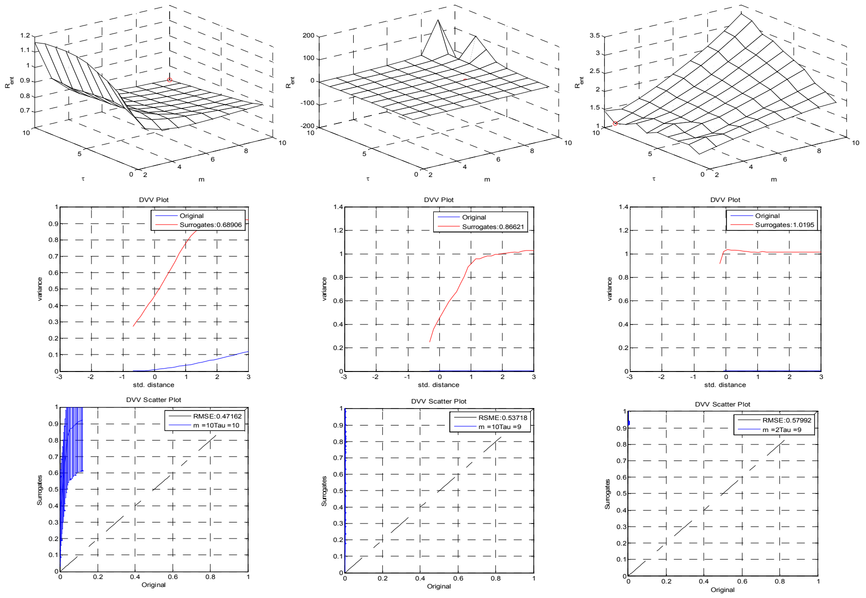

ζ=0.05 ζ=0.2 ζ=0.5

METHOD 2

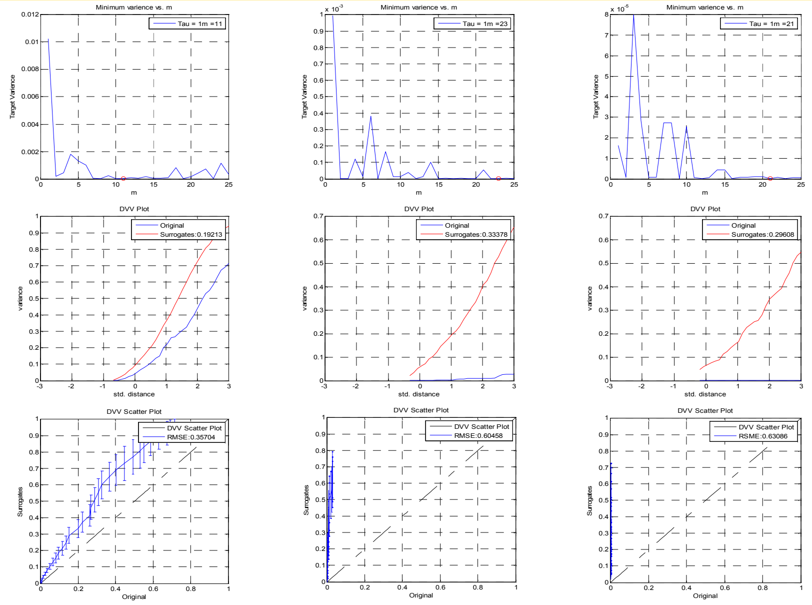

ζ=0.05 ζ=0.2 ζ=0.5

METHOD 3

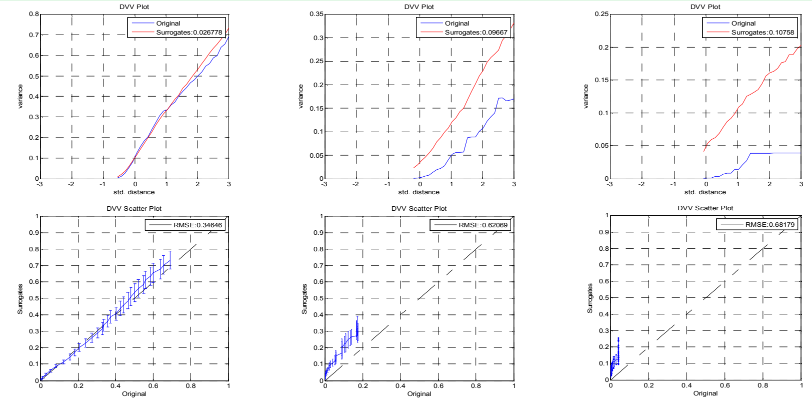

	SYSTEM	DETAILS	VARIABLES		METHOD 1				METHOD 2				METHOD 3			
				mass:	best m	best τ	rmse	RMSE	calc m	τ	rmse	RMSE	set m	set τ	rmse	RMSE
3	OVERDAMPED SDOF SYSTEM OSCILATION FOR VARYING DAMPING VALUES (only for overdamped case xi>1)!!!	SDOF Damped Oscillation x''+2ζwnx'+wn²x=0;	natural frequency wn=7; initial displacement x0=3; initial velocity v0=1; time duration to test tf=30s;	ζ=7	5	10	0.6491	0.4364	18	1	0.2885	0.3296	3	1	0.0486	0.2918
				ζ=5	2	1	0.0313	0.4253	24	1	0.4093	0.4717	3	1	0.0742	0.4349
				ζ=1	3	10	0.8726	0.5464	22	1	0.2759	0.6457	3	1	0.0927	0.6995

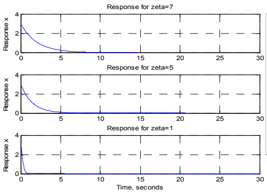

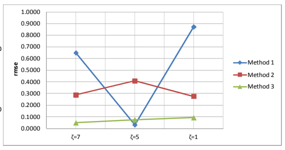

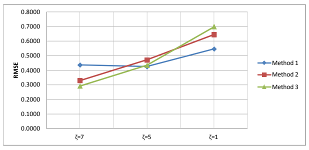

ζ=7 ζ=5 ζ=1

METHOD 1

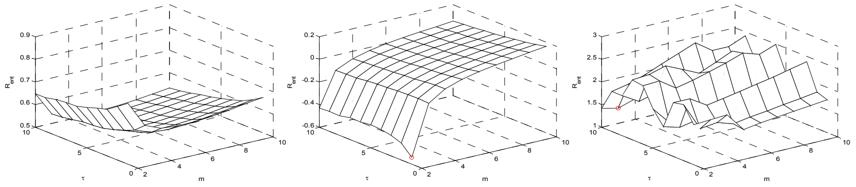

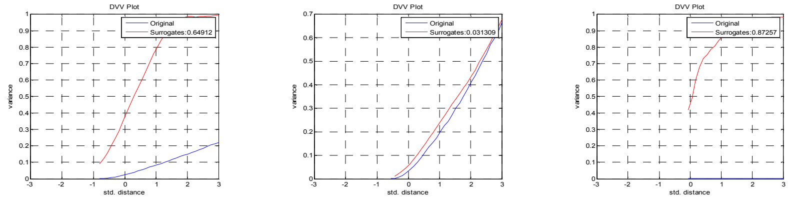

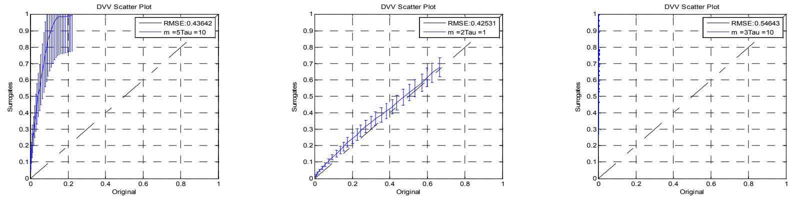

ζ=7 ζ=5 ζ=1

METHOD 2

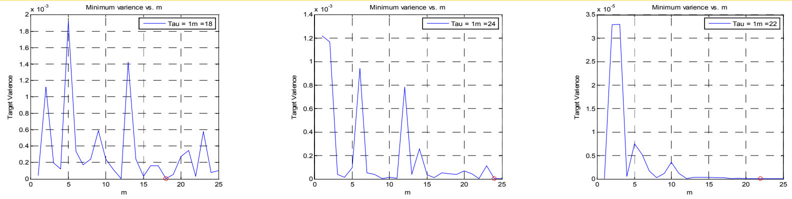

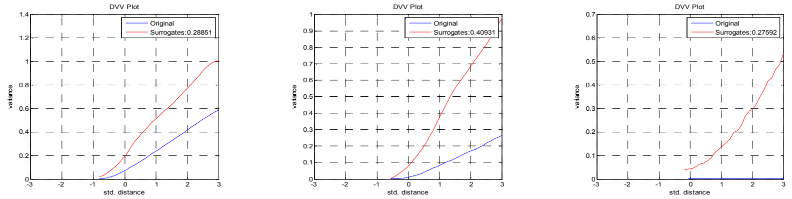

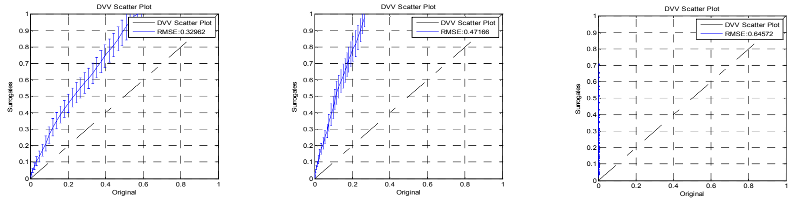

ζ=7 ζ=5 ζ=1

METHOD 3

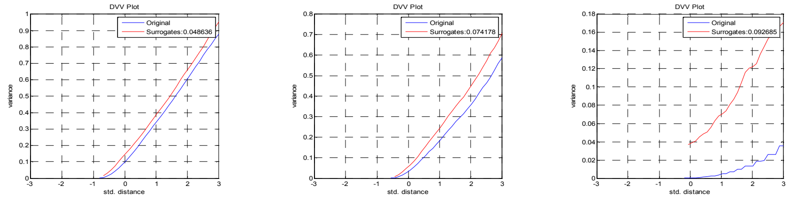

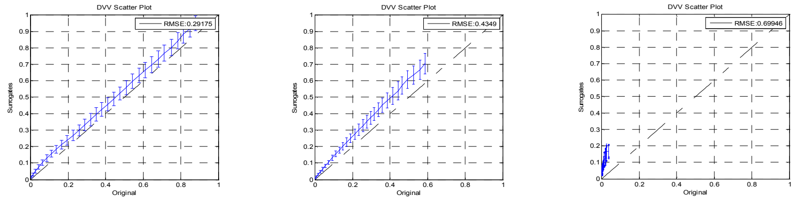

	SYSTEM	DETAILS	VARIABLES		METHOD 1				METHOD 2				METHOD 3			
				mass:	best **m**	best **τ**	rmse	RMSE	calc **m**	**τ**	rmse	RMSE	set **m**	set **τ**	rmse	RMSE
4	HARMONIC EXCITATION OF UNDAMPED SDOF SYSTEMS effect of varying driving frequency w for a given natural frequency wn	SDOF Harmonic Undamped Oscillation mx"+kx=Focoswt;	natural frequency wn=7; initial displacement x0=0; initial velocity v0=0; force magnitude per unit mass f0=6; time duration to test tf=30s;	wdr=3	4	10	0.2300	0.3334	5	1	0.0061	0.1073	3	1	0.0028	0.1185
				wdr=27	6	8	0.0365	0.2433	19	1	0.0238	0.2103	3	1	0.0108	0.2143
				wdr=42	5	10	0.0682	0.2271	10	1	0.0606	0.2832	3	1	0.0736	0.3205

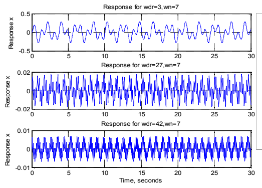

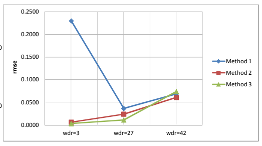

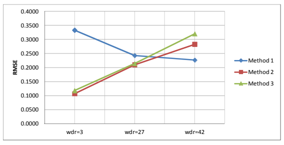

wdr=3 wdr=27 wdr=42

METHOD 1

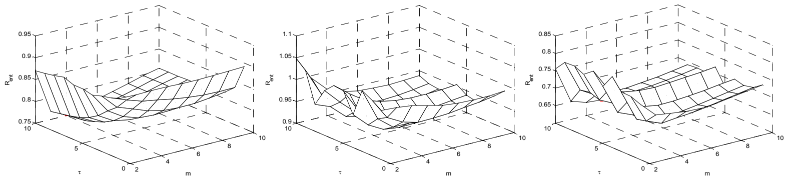

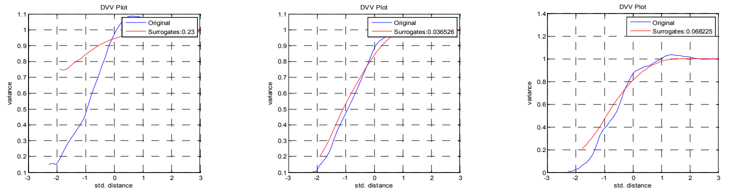

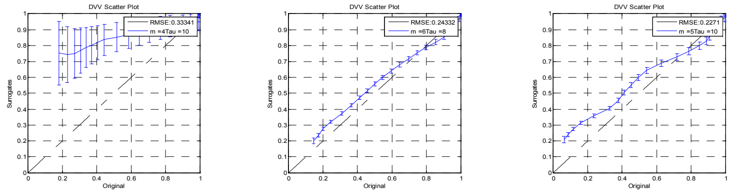

wdr=3 wdr=27 wdr=42

METHOD 2

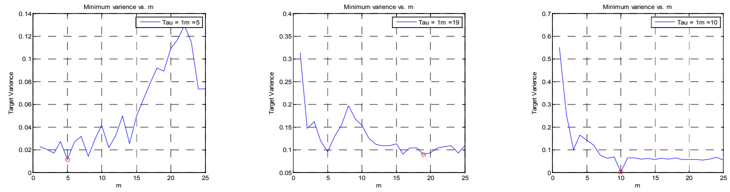

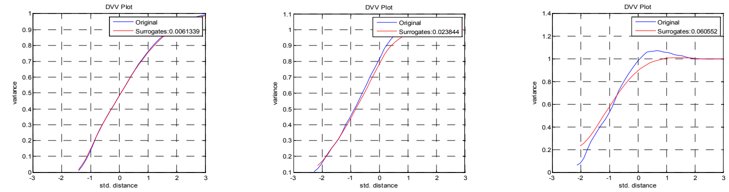

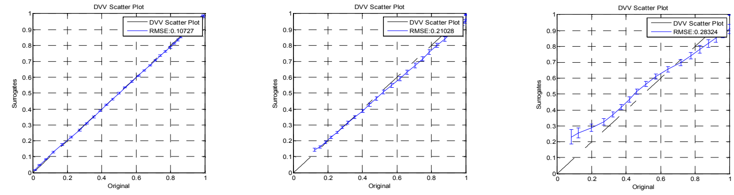

wdr=3 wdr=27 wdr=42

METHOD 3

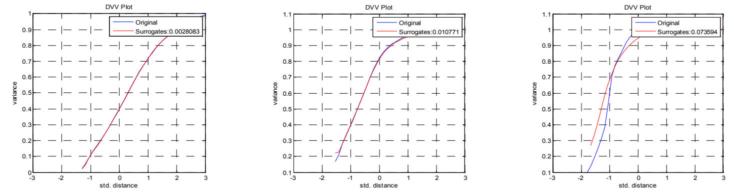

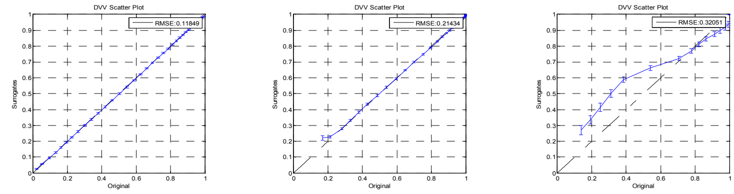

	SYSTEM	DETAILS	VARIABLES		METHOD 1				METHOD 2				METHOD 3			
				mass:	best m	best τ	rmse	RMSE	calc m	τ	rmse	RMSE	set m	set τ	rmse	RMSE
5	HARMONIC EXCITATION OF UNDAMPED SDOF SYSTEMS<br>effect of varying natural frequency wn for a given driving frequency w	SDOF Harmonic Undamped Oscillation<br>mx''+kx=Focoswt ;	driving frequency wdr=7;<br>initial displacement x0=0;<br>initial velocity v0=0;<br>force magnitude per unit mass f0=6;<br>time duration to test tf=30s;	wn=3	4	10	0.2300	0.3334	4	1	0.0042	0.1071	3	1	0.0034	0.1176
				wn=12	5	7	0.0627	0.2938	3	1	0.1336	0.1416	3	1	0.1283	0.1406
				wn=26	6	9	0.0617	0.2581	17	1	0.0388	0.2256	3	1	0.0170	0.2037

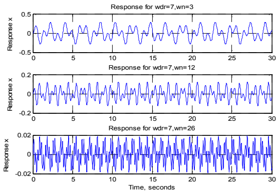

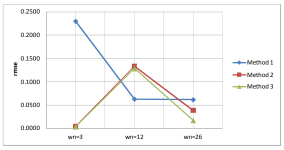

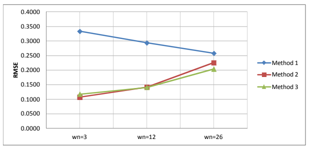

wn=3 wn=12 wn=26

METHOD 1

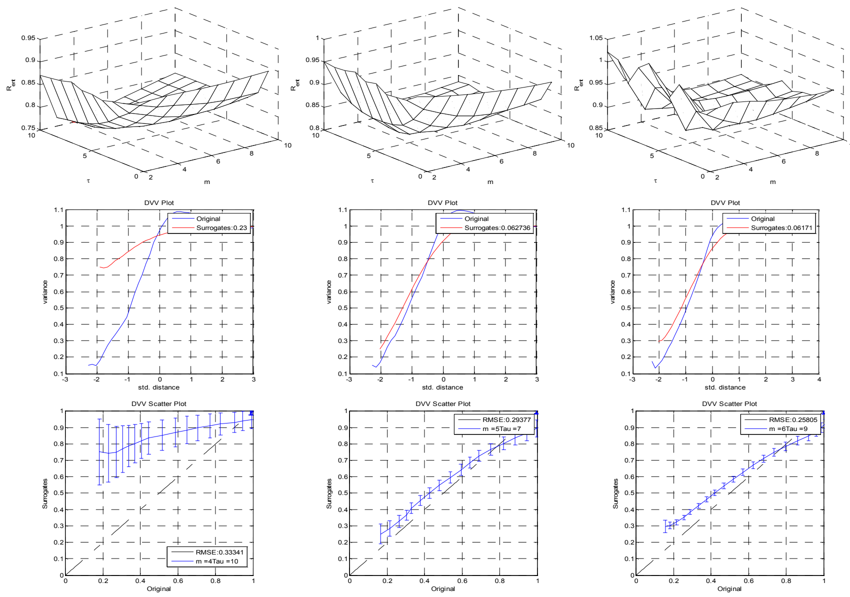

wn=3 wn=12 wn=26

METHOD 2

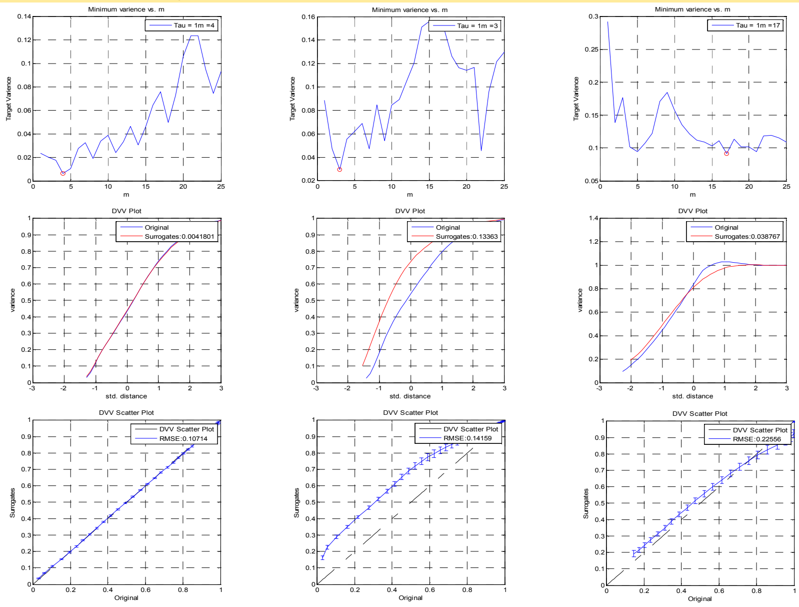

wn=3 wn=12 wn=26

METHOD 3

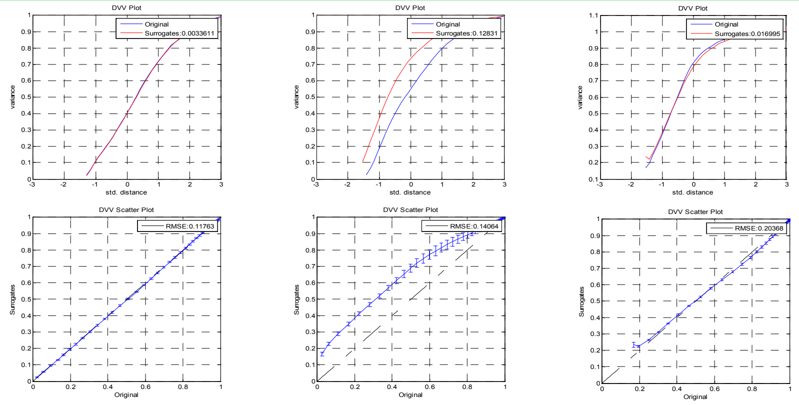

	SYSTEM	DETAILS	VARIABLES		METHOD 1				METHOD 2				METHOD 3			
				mass:	best m	best τ	rmse	RMSE	calc m	τ	rmse	RMSE	set m	set τ	rmse	RMSE
6	HARMONIC EXCITATION OF UNDAMPED SDOF SYSTEMS<br>BEAT Phenomenon natural and driving frequencies are close but not equal	SDOF Harmonic Undamped Oscillation mx''+kx=Focoswt;	initial displacement x0=0;<br>initial velocity v0=0;<br>force magnitude per unit mass f0=6;<br>time duration to test tf=120s;	wn=3; wdr=3.2;	3	1	0.1314	0.1759	3	1	0.0046	0.1369	3	1	0.0037	0.1366
				wn=12; wdr=12.	6	10	0.1740	0.2360	2	1	0.0068	0.1337	3	1	0.0094	0.1682
				wn=22; wdr=22.	3	6	0.0150	0.1044	3	1	0.1248	0.2167	3	1	0.1260	0.2172

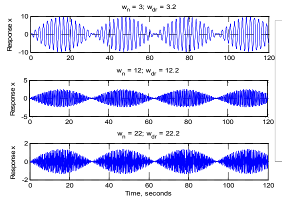

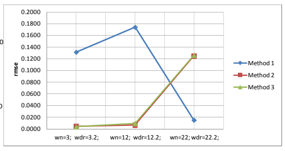

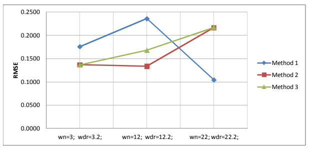

wn=3; wdr=3.2; wn=12; wdr=12.2; wn=22; wdr=22.2;

METHOD 1

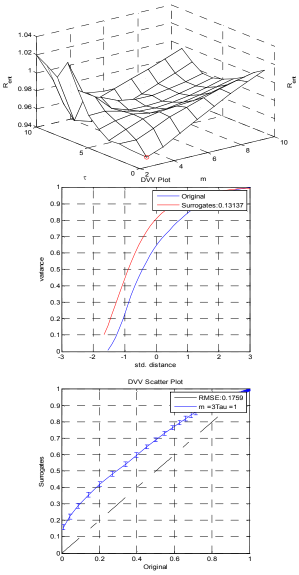

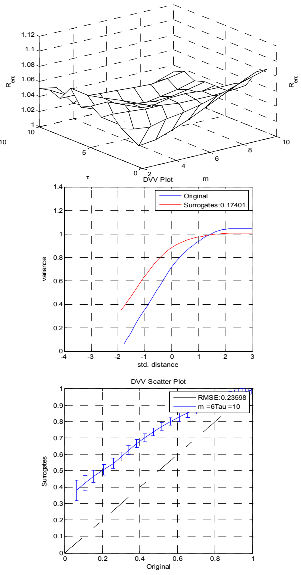

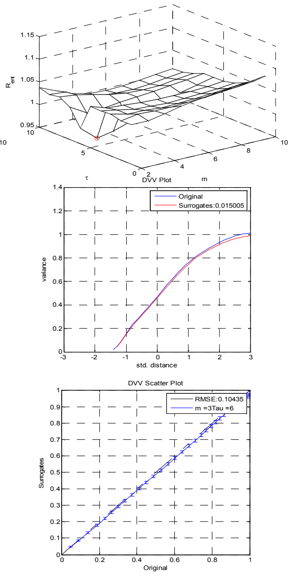

wn=3; wdr=3.2; wn=12; wdr=12.2; wn=22; wdr=22.2;

METHOD 2

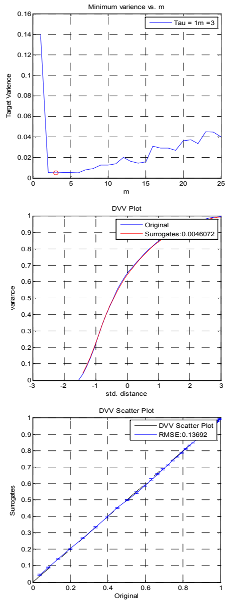

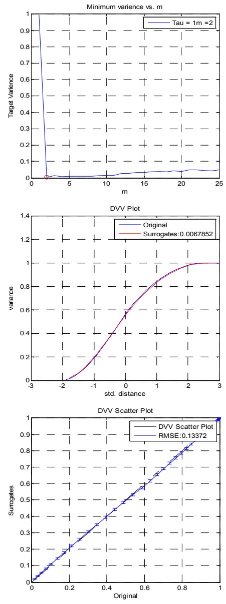

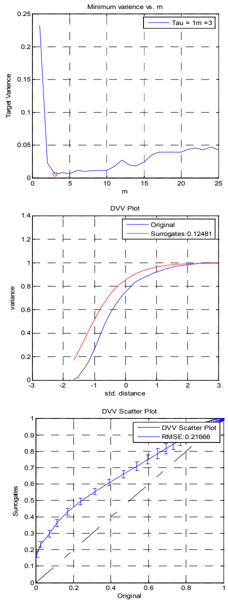

wn=3; wdr=3.2; wn=12; wdr=12.2; wn=22; wdr=22.2;

METHOD 3

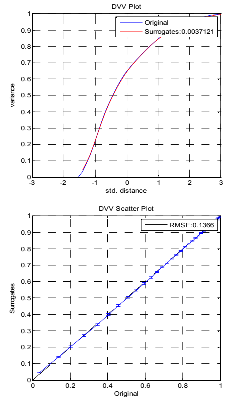

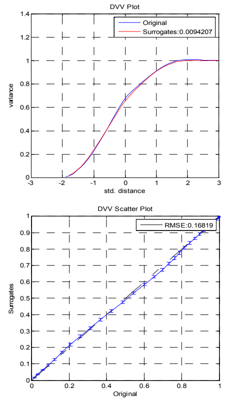

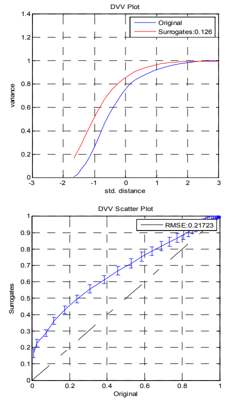

	SYSTEM	DETAILS	VARIABLES		METHOD 1				METHOD 2				METHOD 3			
				wn=wdr=	best m	best τ	rmse	RMSE	calc m	τ	rmse	RMSE	set m	set τ	rmse	RMSE
7	HARMONIC EXCITATION OF UNDAMPED SDOF SYSTEMS<br>RESONANCE natural and driving frequencies are equal	SDOF Harmonic Undamped Oscillation mx''+kx=Focoswt	initial displacement x0=0;<br>initial velocity v0=0;<br>force magnitude per unit mass f0=6;<br>time duration to test tf=120s	wn=wdr	4	9	0.1462	0.1205	7	1	0.0165	0.1439	3	1	0.0096	0.1190
				wn=wdr	7	10	0.0613	0.1334	3	1	0.0027	0.1461	3	1	0.0035	0.1462
				wn=wdr	4	6	0.0194	0.1176	6	1	0.0044	0.1199	3	1	0.0041	0.1646

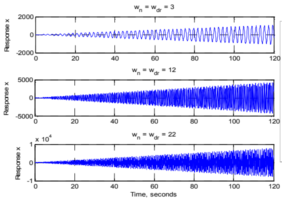

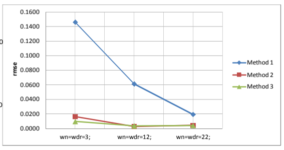

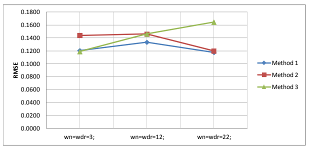

wn=wdr=3; wn=wdr=12; wn=wdr=22;

METHOD 1

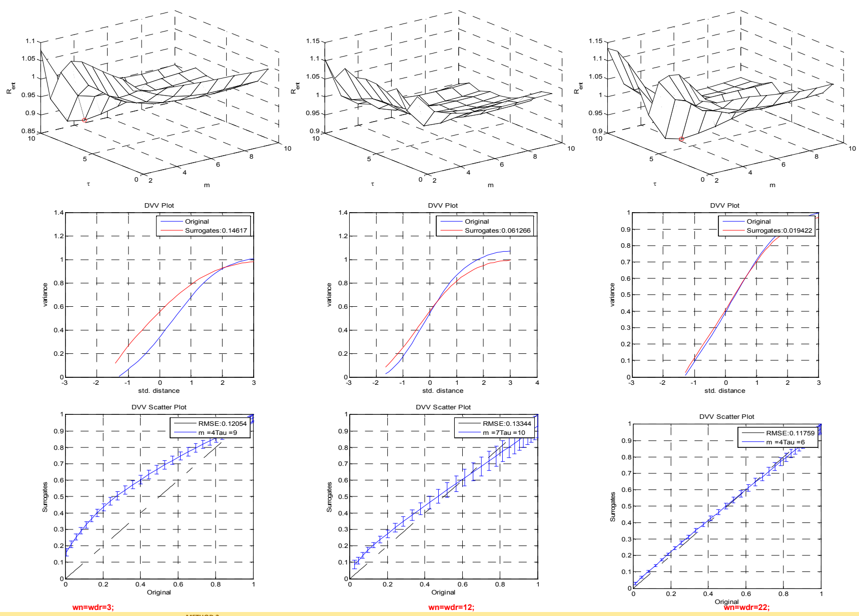

wn=wdr=3; wn=wdr=12; wn=wdr=22;

METHOD 2

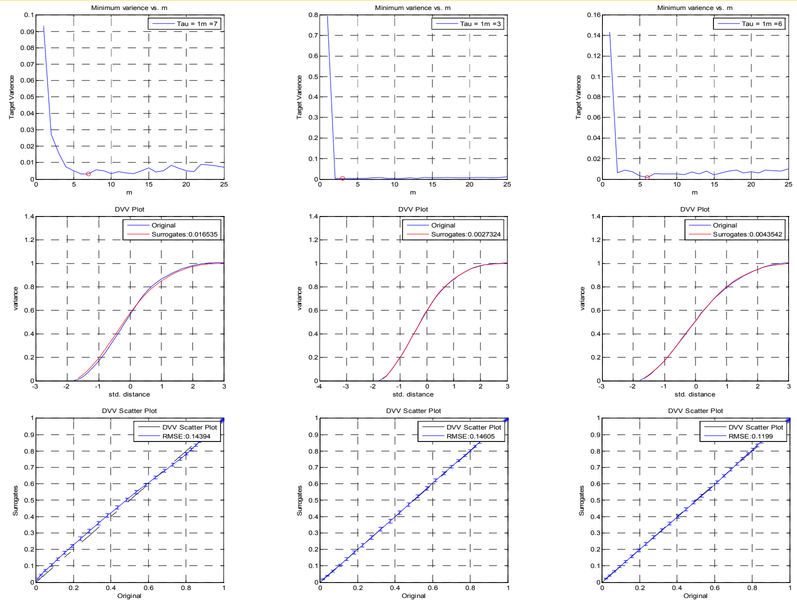

wn=wdr=3; wn=wdr=12; wn=wdr=22;

METHOD 3

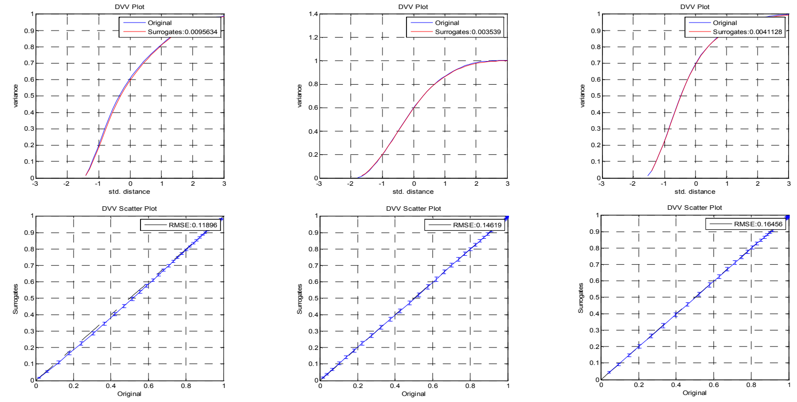

8	SYSTEM	DETAILS	VARIABLES		METHOD 1				METHOD 2				METHOD 3			
				wn=wdr=	best m	best τ	rmse	RMSE	calc m	τ	rmse	RMSE	set m	set τ	rmse	RMSE
	HARMONIC EXCITATION OF DAMPED SDOF SYSTEMS (VARYING DAMPING VALUES)	SDOF Harmonic Damped Oscillation mx''+cx'+kx=Fcos(wt)	driving frequency wdr =3; natural frequency wn=3.5; force magnitude per unit mass f0=6; time duration to test tf=30s;	ζ=0.05	5	10	0.1243	0.2020	4	1	0.0039	0.1476	3	1	0.0052	0.1550
				ζ=0.2	7	10	0.0550	0.1791	3	1	0.0030	0.1617	3	1	0.0048	0.1620
				ζ=0.5	6	10	0.2053	0.2243	4	1	0.0044	0.1615	3	1	0.0082	0.1659

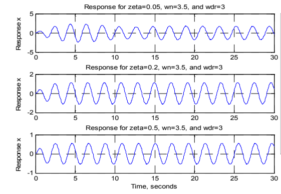

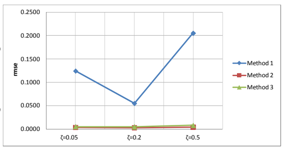

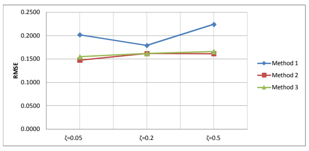

ζ=0.05 ζ=0.2 ζ=0.5

METHOD 1

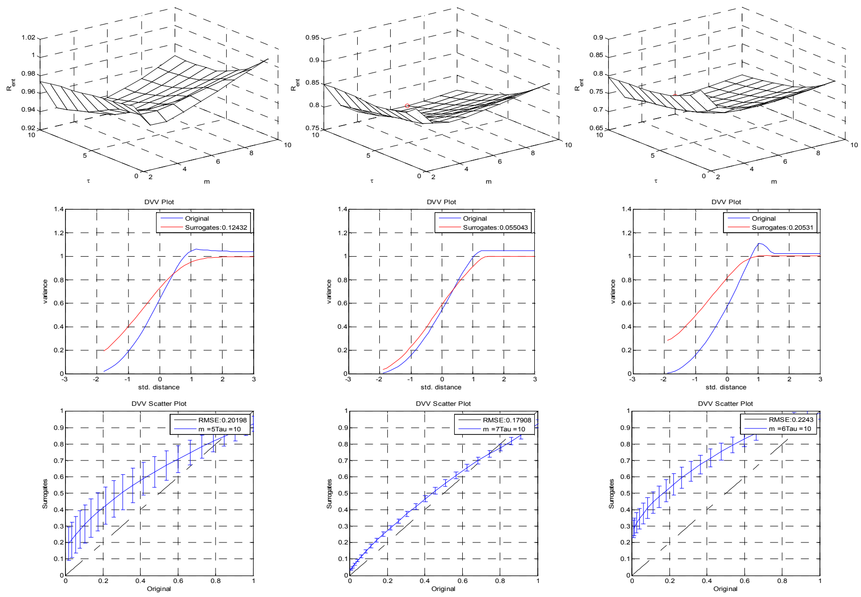

ζ=0.05 ζ=0.2 ζ=0.5

METHOD 2

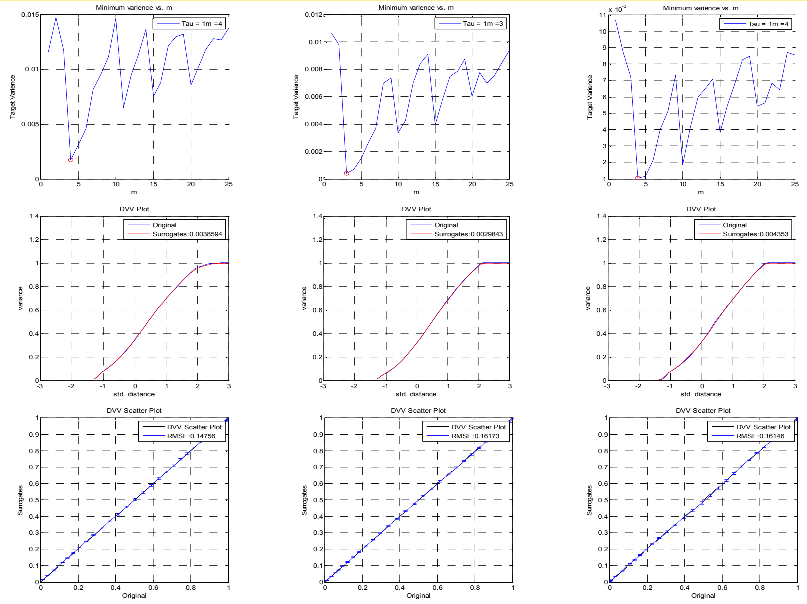

ζ=0.05 ζ=0.2 ζ=0.5

METHOD 3

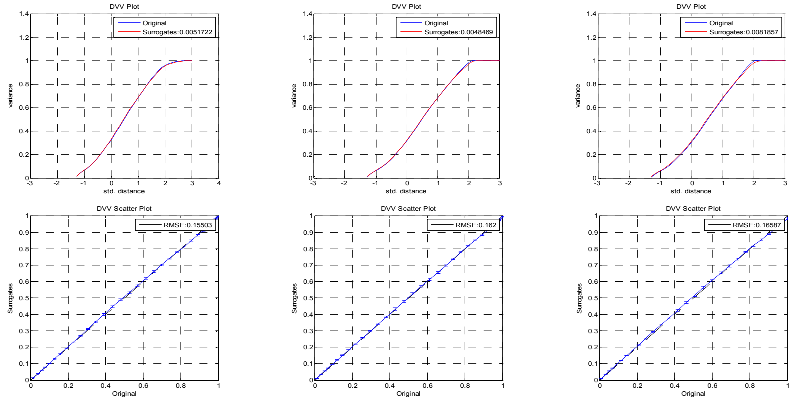

	SYSTEM	DETAILS	VARIABLES		METHOD 1				METHOD 2				METHOD 3			
					best m	best τ	rmse	RMSE	calc m	τ	rmse	RMSE	set m	set τ	rmse	RMSE
9	HARMONIC EXCITATION OF DAMPED SDOF SYSTEMS VARYNG NATURAL FREQUENCY	SDOF Harmonic Damped Oscillation mx''+cx'+kx=Fcos(wt)	driving frequency wdr =7; damping ratio ζ=0.05; force magnitude per unit mass f0=6; time duration to test tf=30s;	wn=3	4	1	0.0403	0.1326	1	1	0.0110	0.1507	3	1	0.0312	0.1383
				wn=12	3	1	0.0029	0.1613	6	1	0.0075	0.1496	3	1	0.0059	0.1625
				wn=26	4	10	0.2174	0.2077	4	1	0.0034	0.1604	3	1	0.0054	0.1648

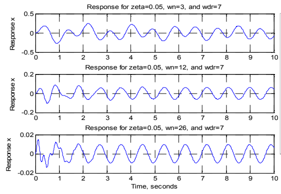

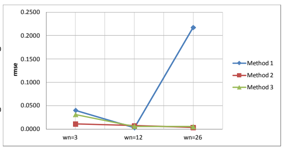

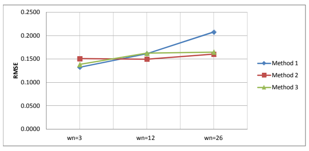

wn=3 wn=12 wn=26

METHOD 1

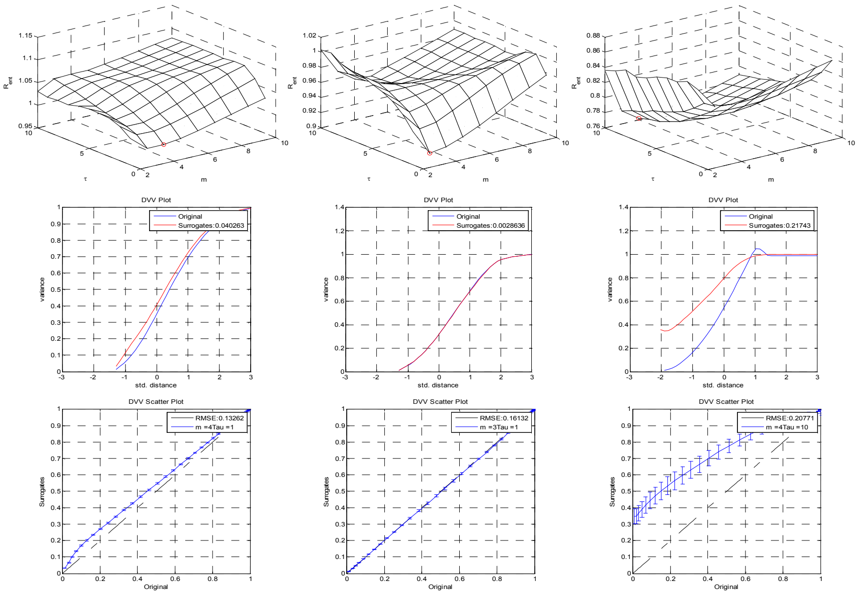

wn=3 wn=12 wn=26

METHOD 2

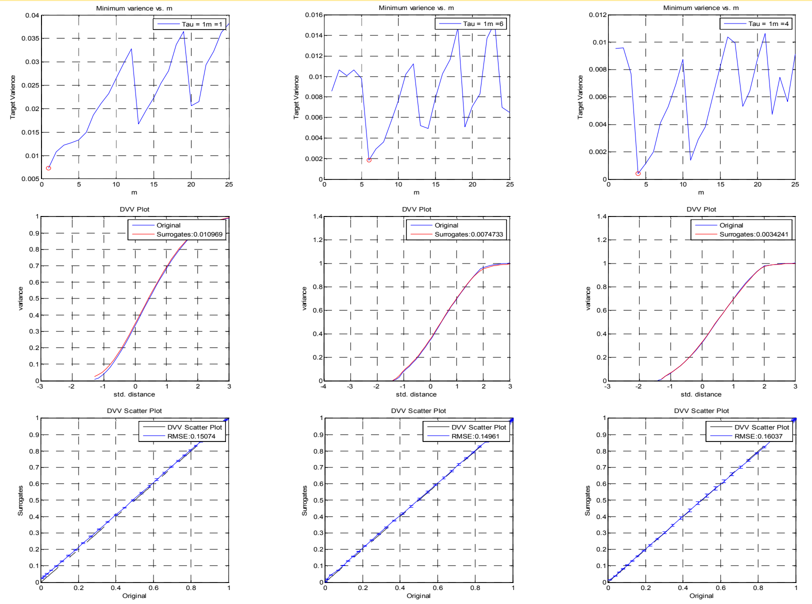

wn=3 wn=12 wn=26

METHOD 3

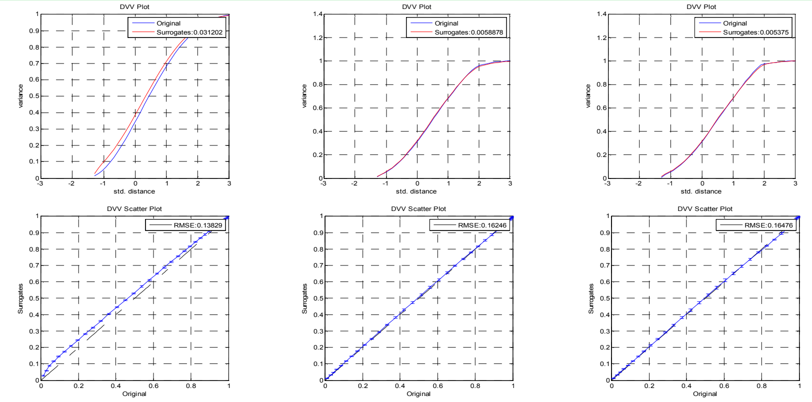

	SYSTEM	DETAILS	VARIABLES		METHOD 1				METHOD 2				METHOD 3			
				base freq	best m	best τ	rmse	RMSE	calc m	τ	rmse	RMSE	set m	set τ	rmse	RMSE
10	BASE EXCITATION OF SDOF SYSTEMS VARYING EXCITATION FREQUENCY	SDOF Base excitation mx''+c(x'-y')+k(x-y)=0; y(t)=Ysin(wbt)	base excitation magnitude yo=3; damping ratio ζ=0.05; natural frequency wn=4 time duration to test tf=10s;	wb=2	4	1	0.0125	0.1600	19	1	0.0159	0.1489	3	1	0.0053	0.1651
				wb=6	4	1	0.0415	0.1359	1	1	0.0029	0.1548	3	1	0.0043	0.1509
				wb=12	3	1	0.0049	0.1604	1	1	0.0074	0.1765	3	1	0.0101	0.1661

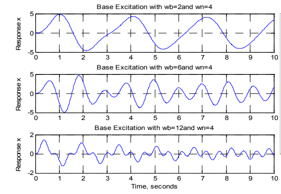

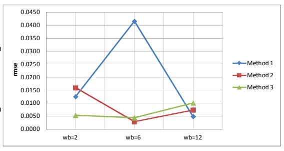

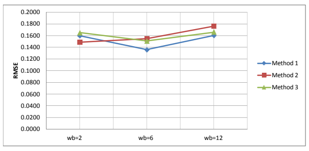

wb=2 wb=6 wb=12

METHOD 1

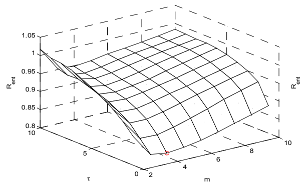

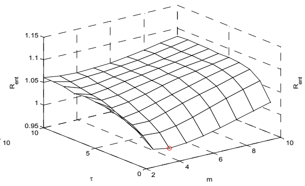

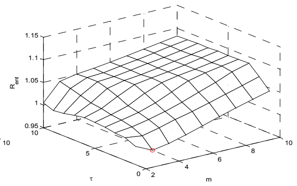

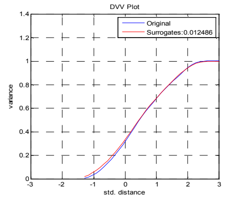

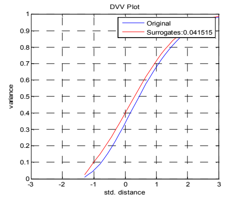

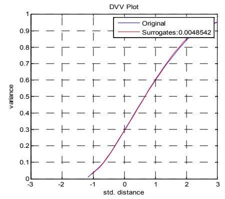

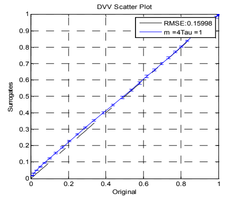

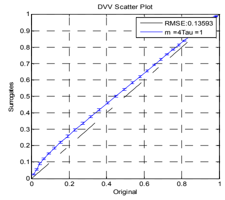

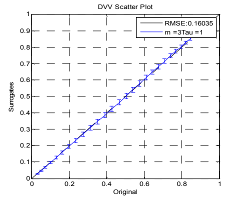

wb=2 wb=6 wb=12

METHOD 2

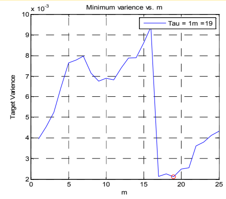

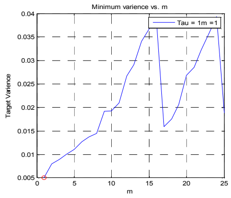

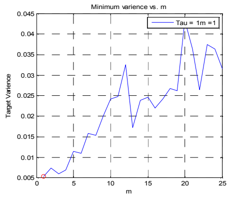

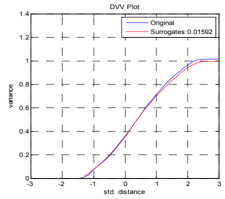

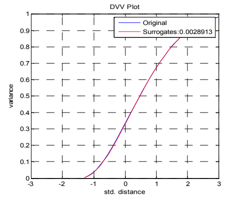

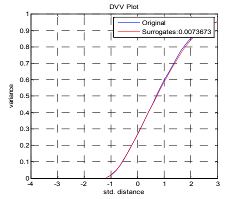

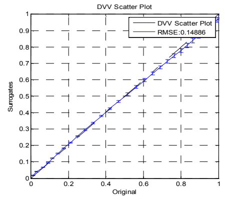

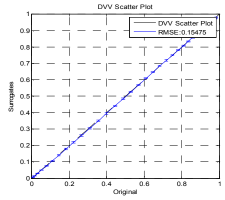

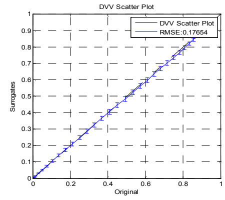

wb=2 wb=6 wb=12

METHOD 3

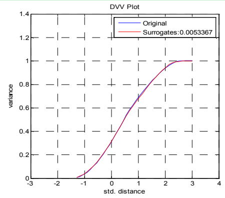

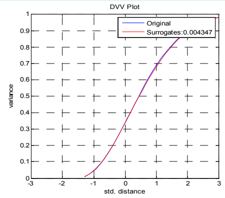

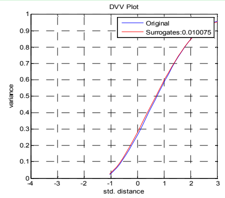

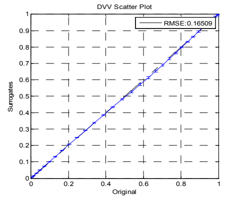

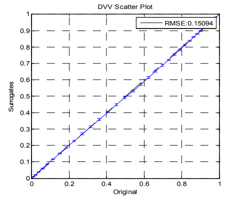

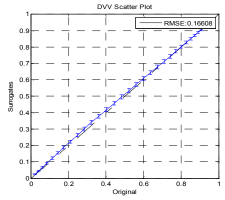

	SYSTEM	DETAILS	VARIABLES		METHOD 1				METHOD 2				METHOD 3			
				baseamplitu	best m	best τ	rmse	RMSE	calc m	τ	rmse	RMSE	set m	set τ	rmse	RMSE
11	BASE EXCITATION OF SDOF SYSTEMS VARYING BASE EXCITATION MAGNITUDE	SDOF Base excitation mx''+c(x'-y')+k(x-y)=0; y(t)=Ysin(wbt)	base excitation frequency wb=6; damping ratio ζ=0.05; natural frequency wn=4 time duration to test tf=10s;	y0=3	4	1	0.0415	0.1359	1	1	0.0050	0.1567	3	1	0.0018	0.1509
				y0=7	4	1	0.0415	0.1359	1	1	0.0051	0.1571	3	1	0.0018	0.1502
				y0=11	4	1	0.0405	0.1359	1	1	0.0072	0.1543	3	1	0.0028	0.1509

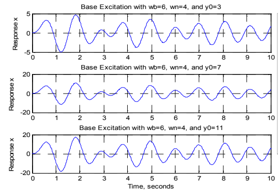

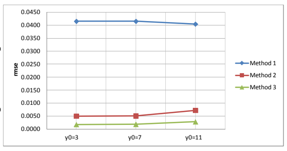

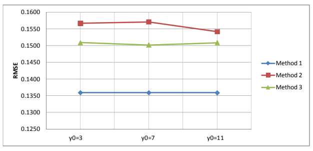

y0=3 y0=7 y0=11

METHOD 1

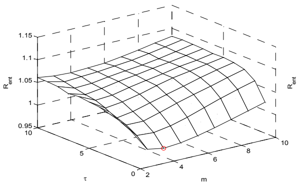
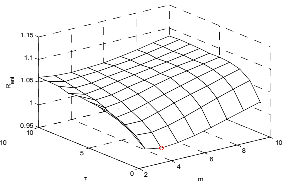
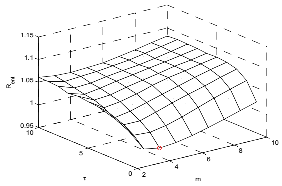

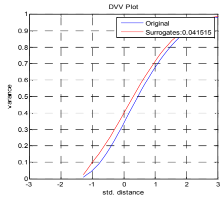

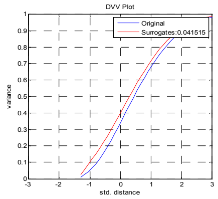

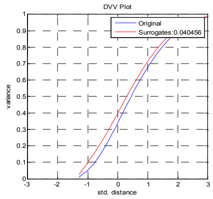

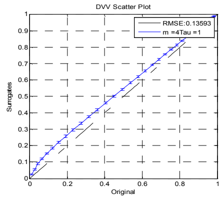

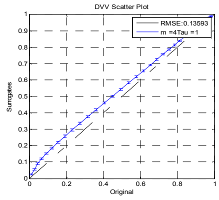

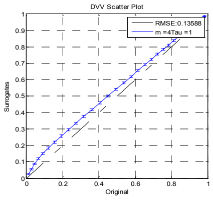

y0=3 y0=7 y0=11

METHOD 2

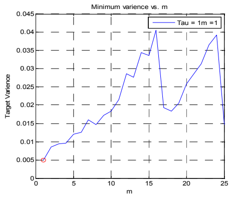

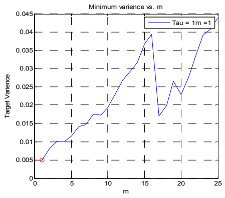

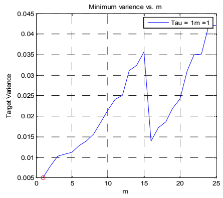

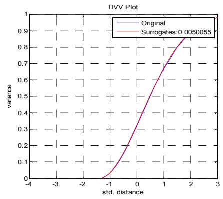

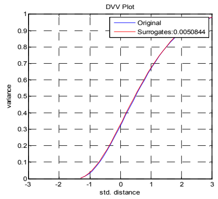

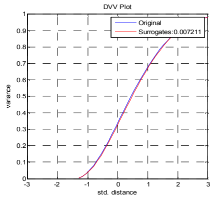

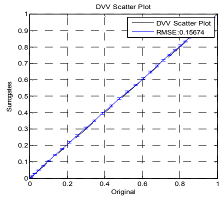

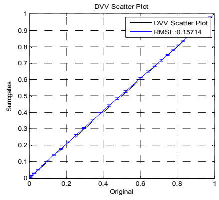

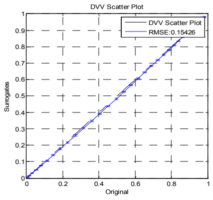

y0=3 y0=7 y0=11

METHOD 3

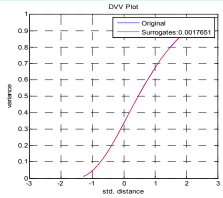

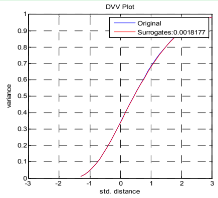

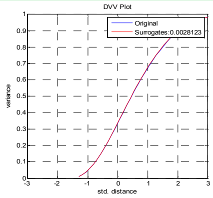

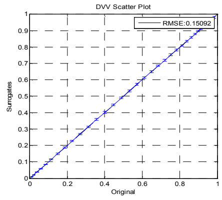

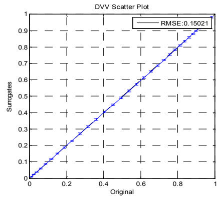

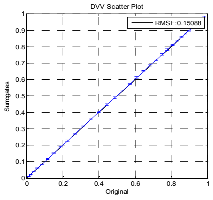

	SYSTEM	DETAILS	VARIABLES		METHOD 1				METHOD 2				METHOD 3			
				damping rat	best m	best τ	rmse	RMSE	calc m	τ	rmse	RMSE	set m	set τ	rmse	RMSE
12	BASE EXCITATION OF SDOF SYSTEMS VARYING DAMPING RATIO	SDOF Base excitation mx''+c(x'-y')+k(x-y)=0; y(t)=Ysin(wbt)	base amplitude y0=3; base excitation frequency wb=6; natural frequency wn=4 time duration to test tf=10s;	ζ=0.05	4	1	0.0415	0.1359	1	1	0.0050	0.1567	3	1	0.0021	0.1511
				ζ=0.1	4	1	0.0412	0.1399	10	1	0.0061	0.1266	3	1	0.0023	0.1561
				ζ=0.3	4	1	0.0371	0.1557	7	1	0.0050	0.1576	3	1	0.0062	0.1717

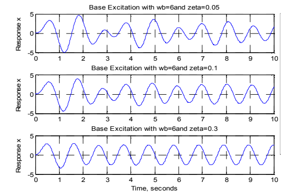

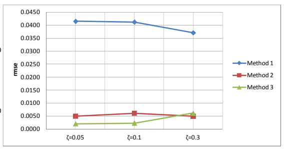

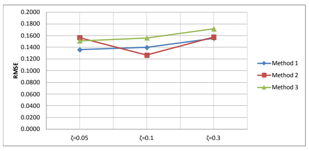

ζ=0.05 ζ=0.1 ζ=0.3

METHOD 1

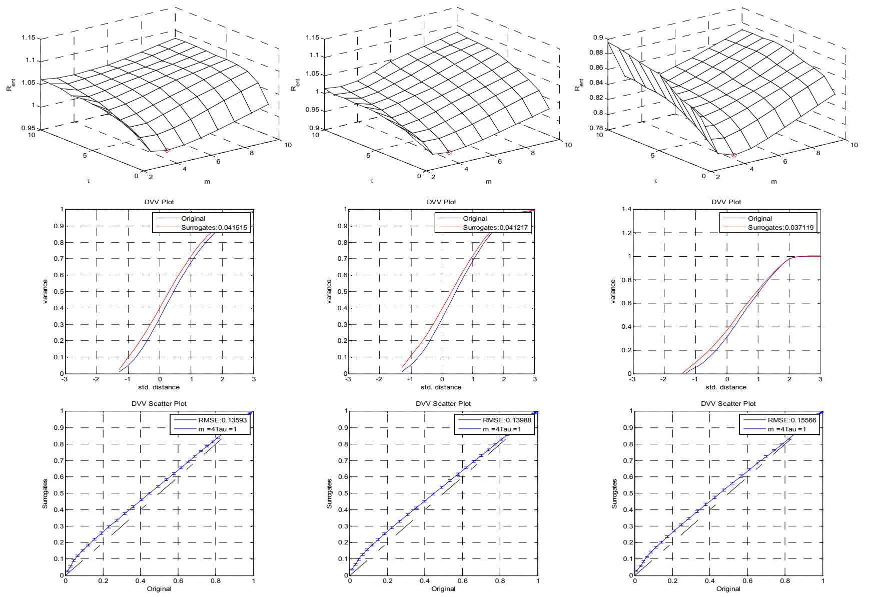

ζ=0.05 ζ=0.1 ζ=0.3

METHOD 2

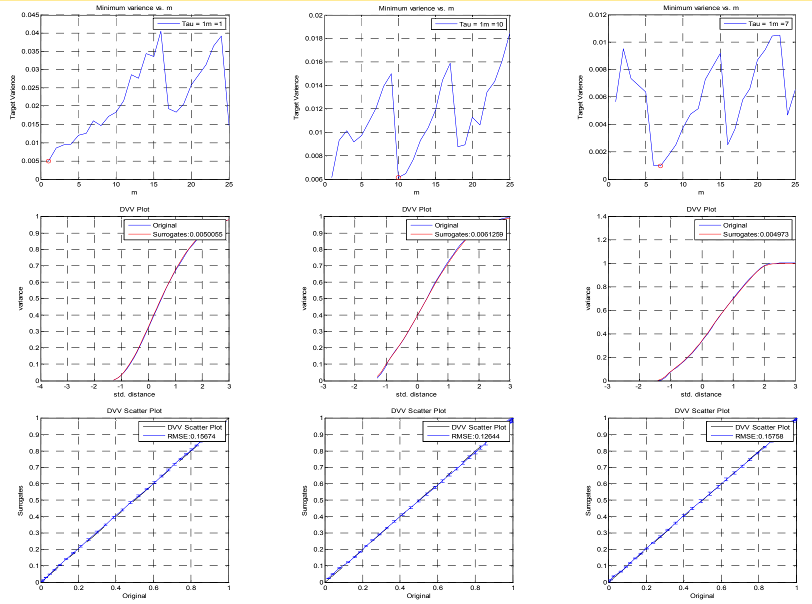

ζ=0.05 ζ=0.1 ζ=0.3

METHOD 3

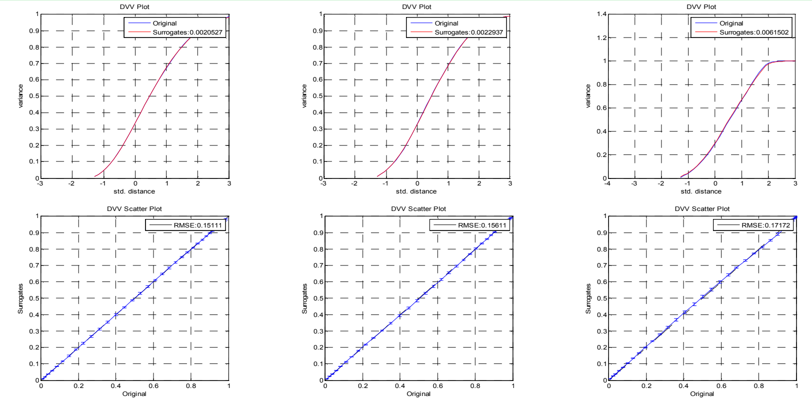

	SYSTEM	DETAILS	VARIABLES		METHOD 1				METHOD 2				METHOD 3			
				natural fre	best m	best τ	rmse	RMSE	calc m	τ	rmse	RMSE	set m	set τ	rmse	RMSE
13	SDOF SYSTEM WITH A ROTATING UNBALANCE VARYING NATURAL FREQUENCY	SDOF having a rotating unbalance for zero initial conditions	rotating mass mo=3; sdof mass m=7; angular velocity of rot mass wr=4; damping ratio ζ=0.05; time duration to test tf=10s; constant e=0.1	wn=2	3	1	0.0079	0.1642	1	1	0.0057	0.1695	3	1	0.0036	0.1702
				wn=6	4	1	0.0224	0.1575	12	1	0.0151	0.1450	3	1	0.0042	0.1661
				wn=12	4	1	0.0064	0.1654	6	1	0.0047	0.1624	3	1	0.0051	0.1681

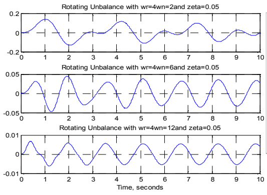

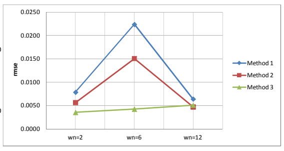

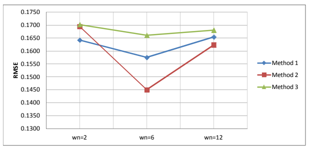

wn=2 wn=6 wn=12

METHOD 1

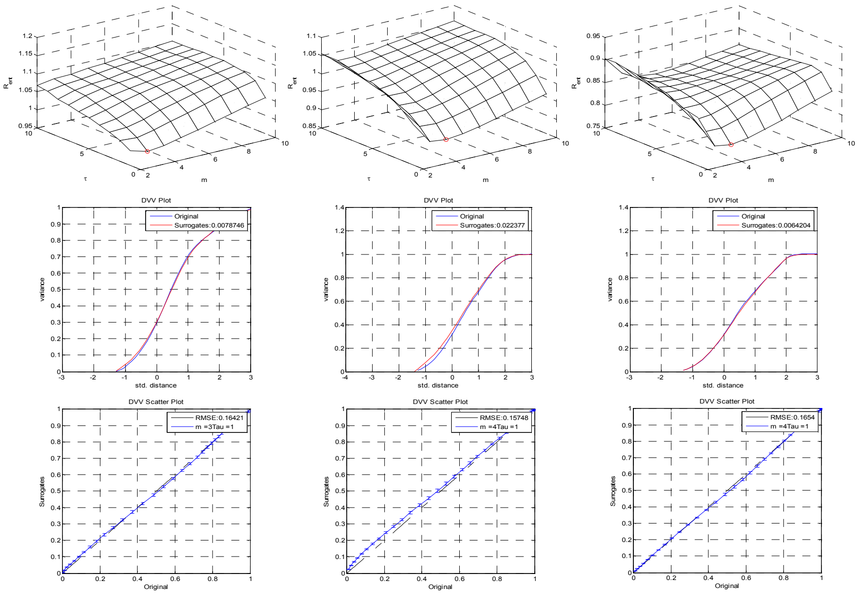

wn=2 wn=6 wn=12

METHOD 2

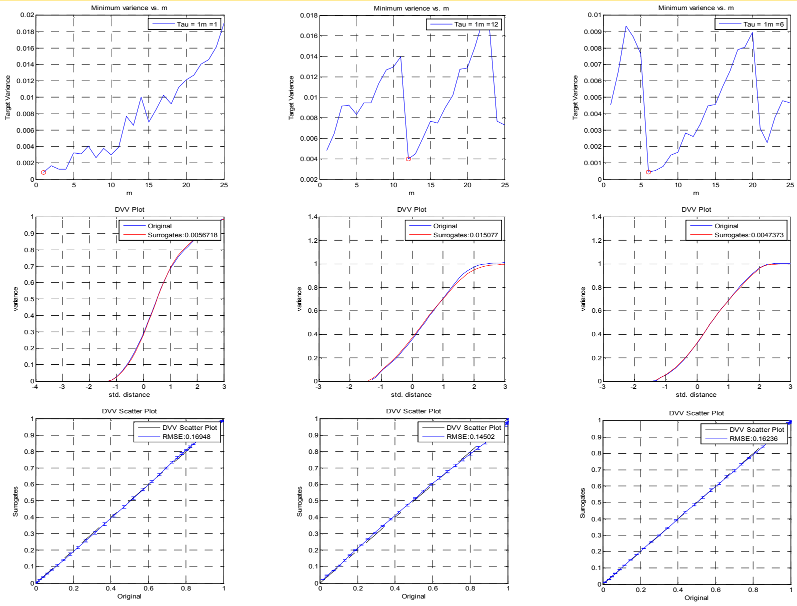

wn=2 wn=6 wn=12

METHOD 3

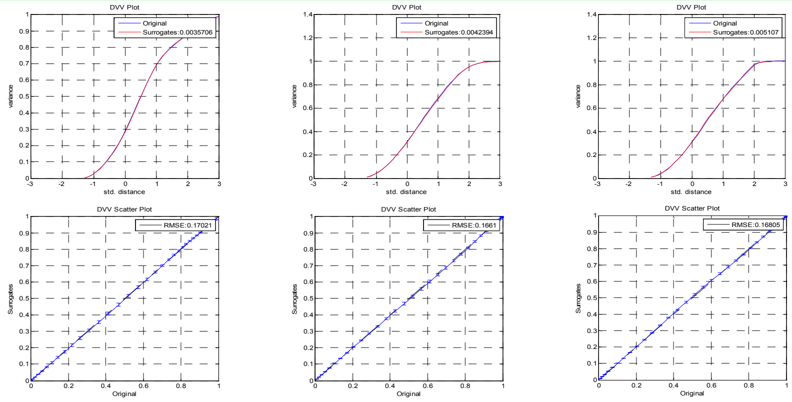

	SYSTEM	DETAILS	VARIABLES	damping rat	METHOD 1 best m	METHOD 1 best τ	METHOD 1 rmse	METHOD 1 RMSE	METHOD 2 calc m	METHOD 2 τ	METHOD 2 rmse	METHOD 2 RMSE	METHOD 3 set m	METHOD 3 set τ	METHOD 3 rmse	METHOD 3 RMSE
14	SDOF SYSTEM WITH A ROTATING UNBALANCE VARYING DAMPING	SDOF having a rotating unbalance for zero initial conditions	rotating mass mo=3; sdof mass m=7; angular velocity of rot mass wr=4; natural fequency wn=12; time duration to test tf=10s; constant e=0.1	ζ=0.05	4	1	0.0064	0.1654	9	1	0.0076	0.1568	3	1	0.0097	0.1695
				ζ=0.1	4	1	0.0207	0.1642	8	1	0.0104	0.1636	3	1	0.0049	0.1737
				ζ=0.3	4	2	0.0531	0.1506	8	1	0.0068	0.1665	3	1	0.0083	0.1746

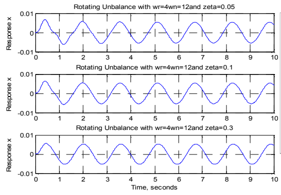

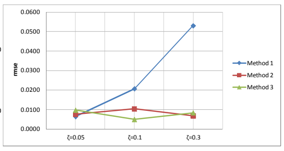

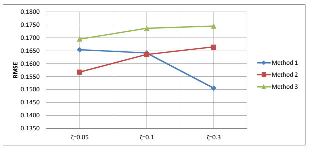

ζ=0.05 ζ=0.1 ζ=0.3

METHOD 1

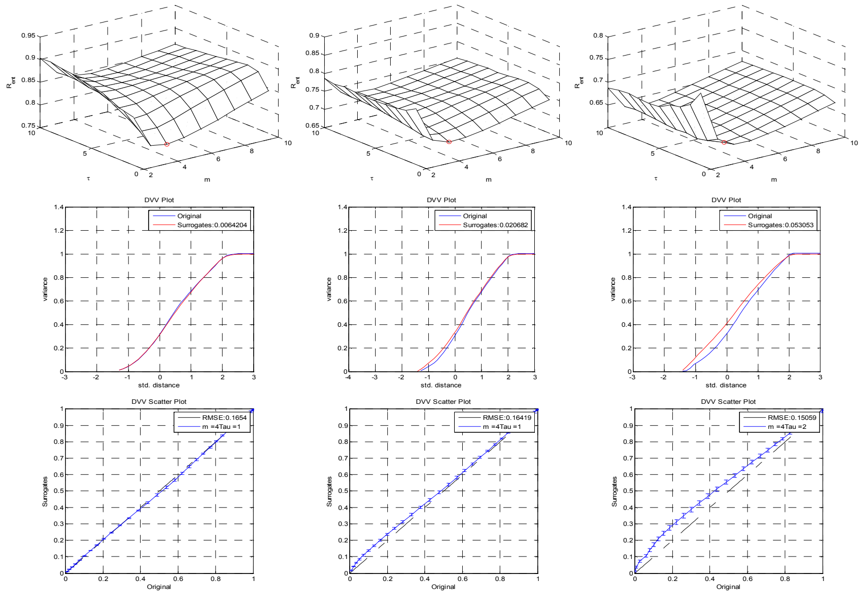

ζ=0.05 ζ=0.1 ζ=0.3

METHOD 2

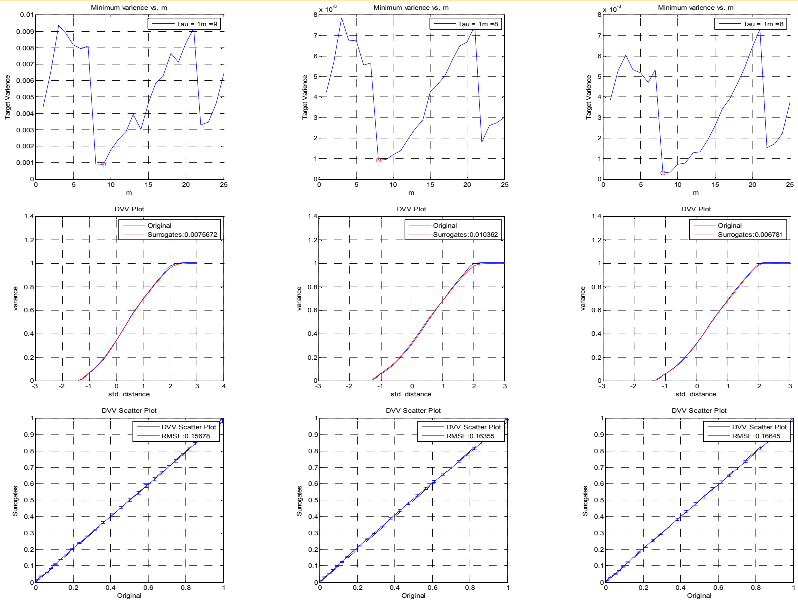

ζ=0.05 ζ=0.1 ζ=0.3

METHOD 3

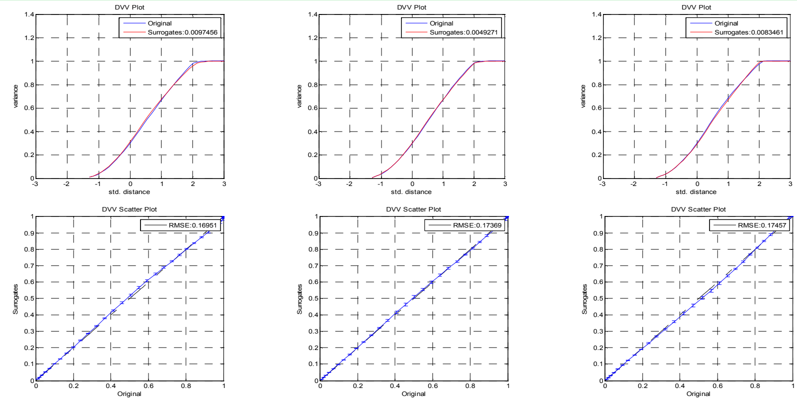

	SYSTEM	DETAILS	VARIABLES		METHOD 1				METHOD 2				METHOD 3			
				mass	best m	best τ	rmse	RMSE	calc m	τ	rmse	RMSE	set m	set τ	rmse	RMSE
15	SDOF SYSTEM WITH A ROTATING UNBALANCE VARYING MASS	SDOF having a rotating unbalance for zero initial conditions	rotating mass mo=3; damping ratio ζ=0.05; angular velocity of rot mass wr=4; natural fequency wn=12; time duration to test tf=10s; constant e=0.1	m=1	4	1	0.0152	0.1595	7	1	0.0056	0.1582	3	1	0.0043	0.1673
				m=3	4	1	0.0202	0.1595	8	1	0.0089	0.1607	3	1	0.0026	0.1674
				m=6	4	1	0.0202	0.1595	7	1	0.0058	0.1599	3	1	0.0026	0.1678

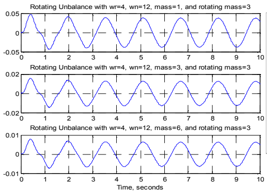

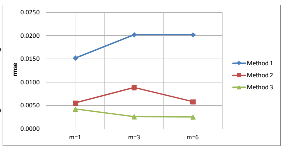

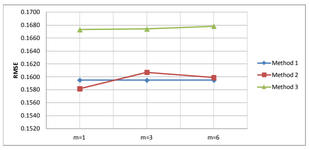

m=1 m=3 m=6

METHOD 1

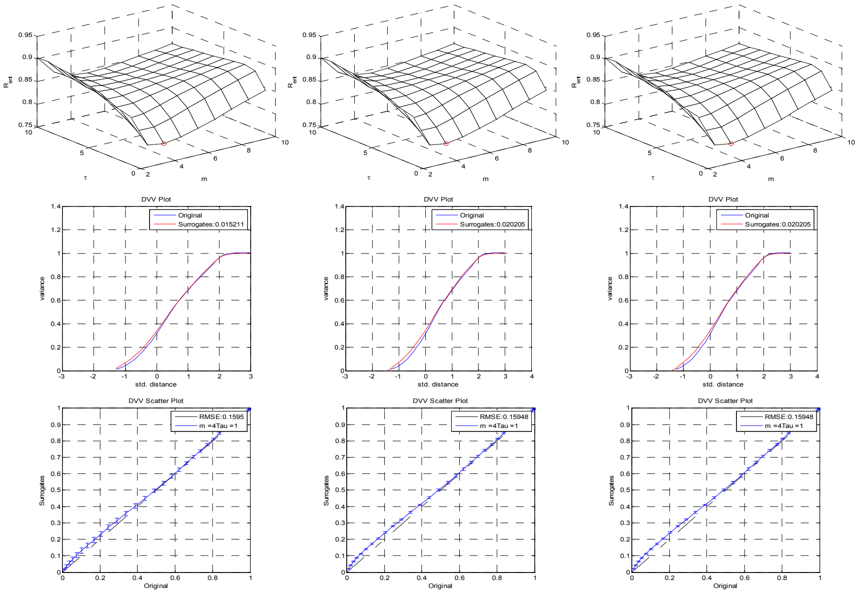

m=1 m=3 m=6

METHOD 2

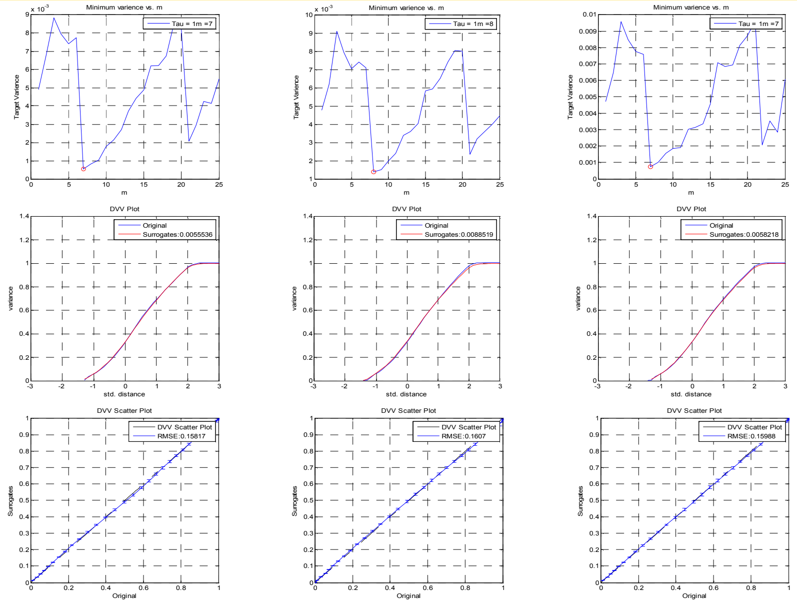

m=1 m=3 m=6

METHOD 3

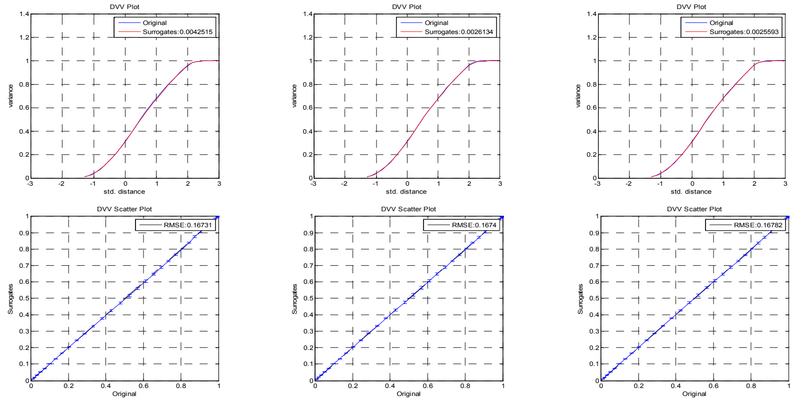

	SYSTEM	DETAILS	VARIABLES		METHOD 1				METHOD 2				METHOD 3			
				force mag	best m	best τ	rmse	RMSE	calc m	τ	rmse	RMSE	set m	set τ	rmse	RMSE
16	SDOF-STEP RESPONSE VARYING FORCE MAGNITUDE	SDOF with step response	initial time to=2; damping ratio ζ=0.05; natural fequency wn=12; time duration to test tf=10s; system mass m=1;	Fm=3	3	1	0.1690	0.3004	1	1	0.0256	0.3678	3	1	0.0364	0.3647
				Fm=7	3	1	0.1690	0.3004	1	1	0.0585	0.3636	3	1	0.0296	0.3588
				Fm=11	3	1	0.0364	0.3533	1	1	0.0265	0.3645	3	1	0.0592	0.3647

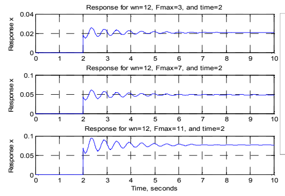

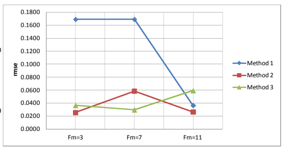

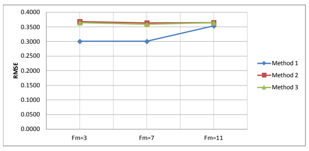

Fm=3 Fm=7 Fm=11

METHOD 1

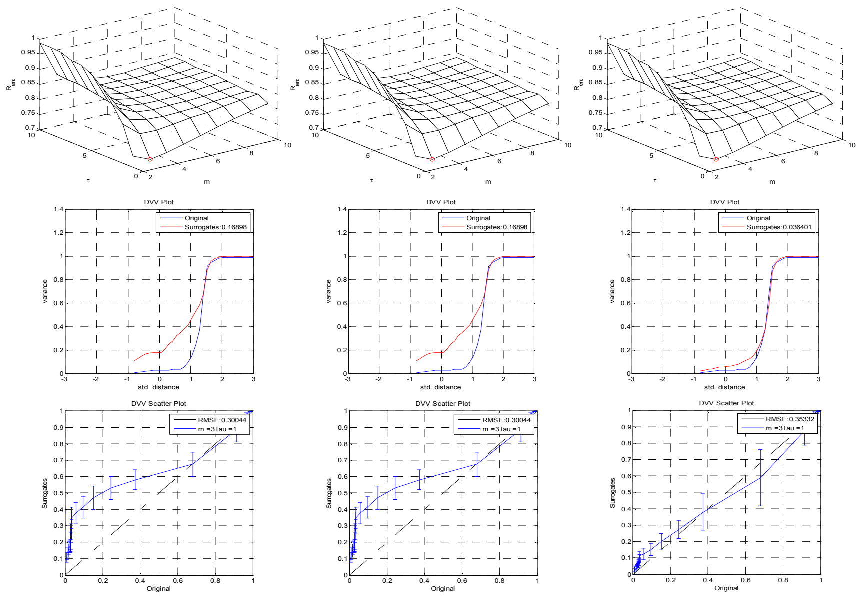

Fm=3 Fm=7 Fm=11

METHOD 2

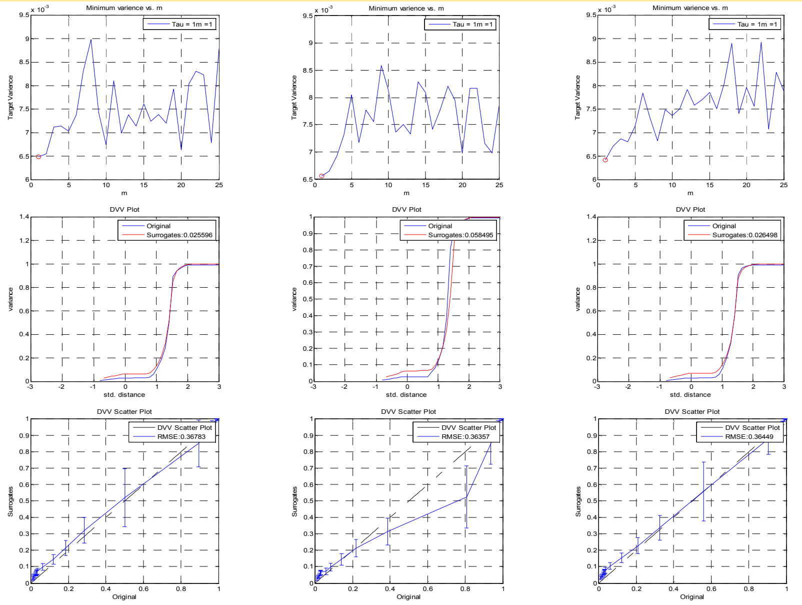

Fm=3 Fm=7 Fm=11

METHOD 3

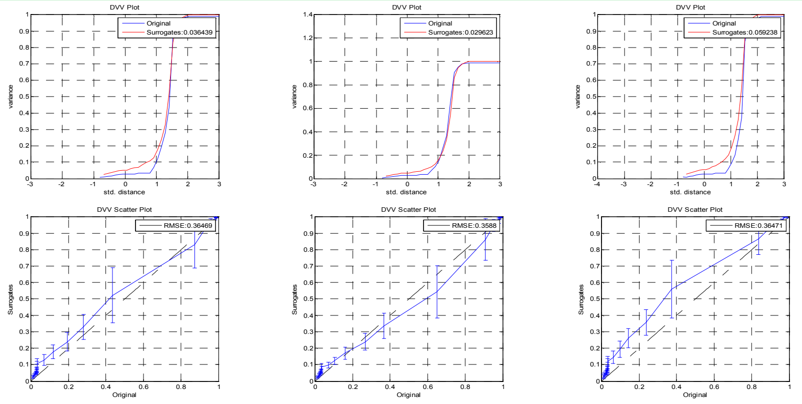

	SYSTEM	DETAILS	VARIABLES		METHOD 1				METHOD 2				METHOD 3			
				nat frequen	best m	best τ	rmse	RMSE	calc m	τ	rmse	RMSE	set m	set τ	rmse	RMSE
17	SDOF-STEP RESPONSE VARYING NATURAL FREQUENCY	SDOF having a step response	system mass m=1; damping ratio ζ=0.05; natural fequency wn=12; time duration to test tf=10s; force magnitude Fm=5; initial time to=2;	wn=2	3	1	0.0488	0.1475	2	1	0.0110	0.1628	3	1	0.0105	0.1626
				wn=6	3	1	0.0865	0.2209	1	1	0.0193	0.2567	3	1	0.0168	0.2534
				wn=12	3	1	0.1690	0.3004	1	1	0.0317	0.3662	3	1	0.0489	0.3632

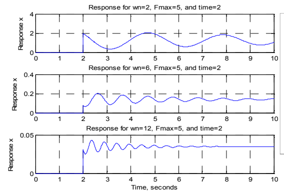

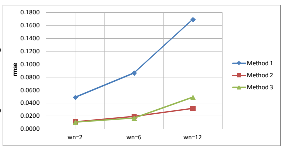

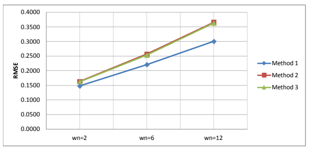

wn=2 wn=6 wn=12

METHOD 1

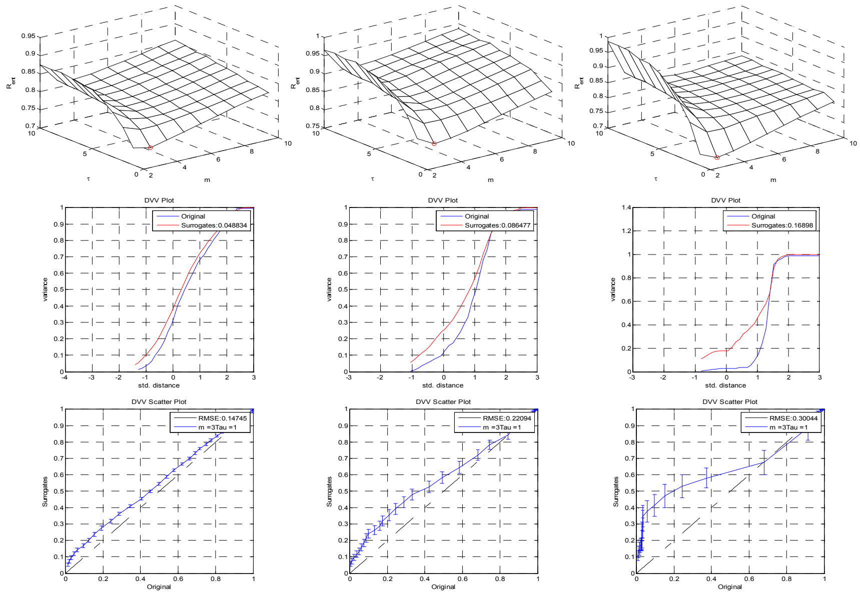

wn=2 wn=6 wn=12

METHOD 2

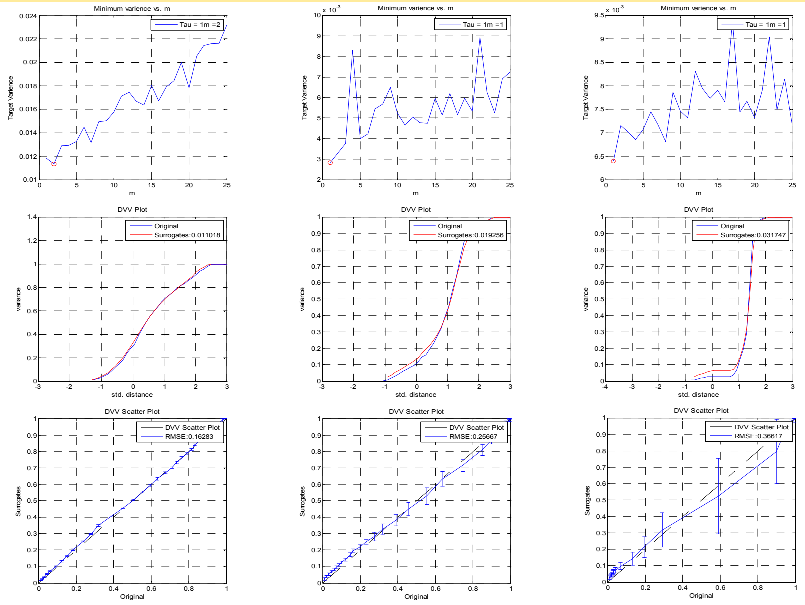

wn=2 wn=6 wn=12

METHOD 3

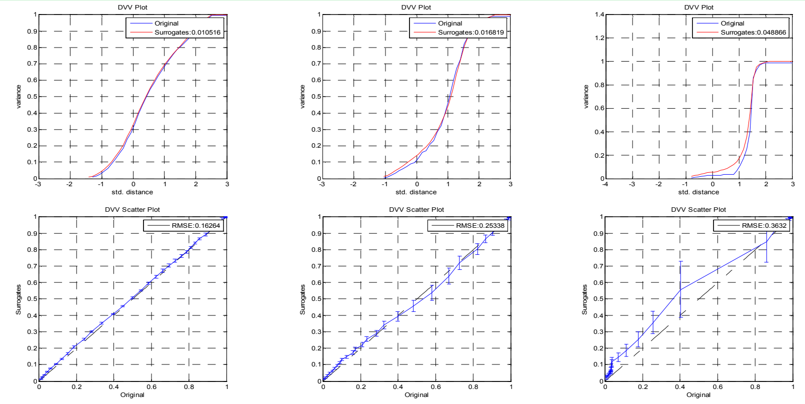

	SYSTEM	DETAILS	VARIABLES		METHOD 1				METHOD 2				METHOD 3			
				damping ra	best m	best τ	rmse	RMSE	calc m	τ	rmse	RMSE	set m	set τ	rmse	RMSE
18	SDOF-STEP RESPONSE VARYING DAMPING RATIO	SDOF having a step response	system mass m=1; natural fequency wn=12; time duration to test tf=10s; force magnitude Fm=5; initial time to=2;	ζ=0.05	3	1	0.1690	0.3004	1	1	0.0347	0.3757	3	1	0.0833	0.3732
				ζ=0.1	2	1	0.0353	0.4032	19	1	0.0881	0.3670	3	1	0.1318	0.4179
				ζ=0.3	2	1	0.0512	0.4191	6	1	0.1731	0.4233	3	1	0.0320	0.4253

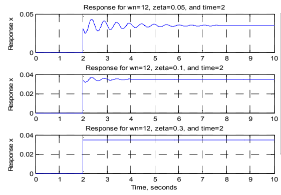

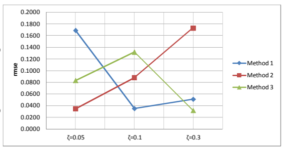

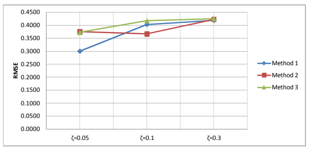

ζ=0.05 ζ=0.1 ζ=0.3

METHOD 1

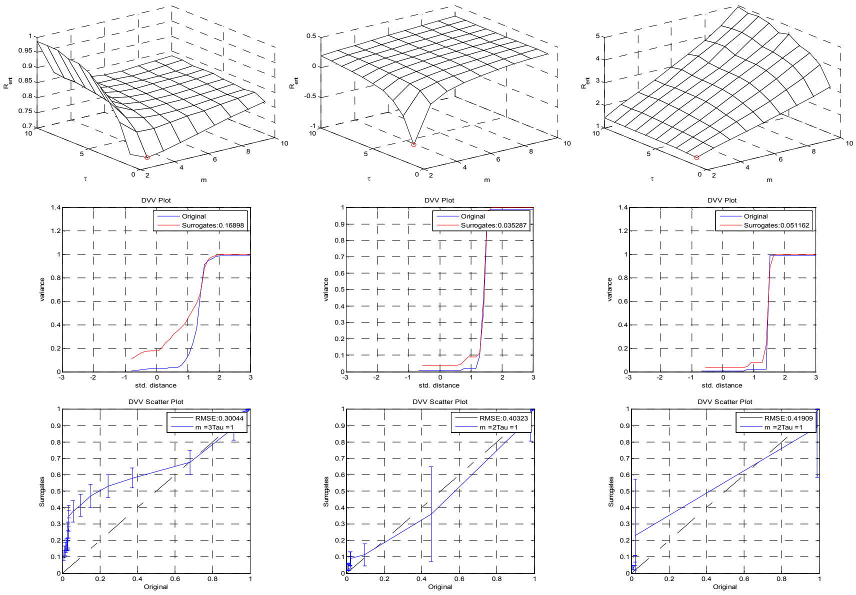

ζ=0.05 ζ=0.1 ζ=0.3

METHOD 2

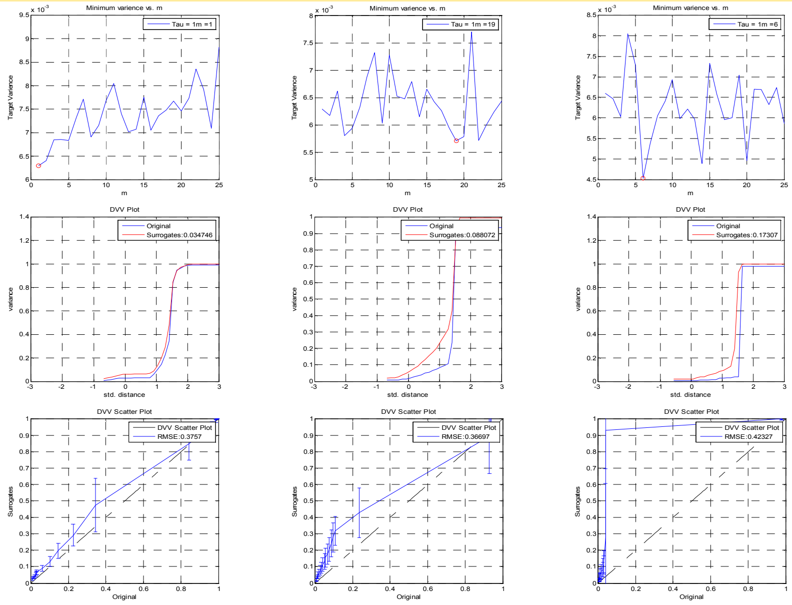

ζ=0.05 ζ=0.1 ζ=0.3

METHOD 3

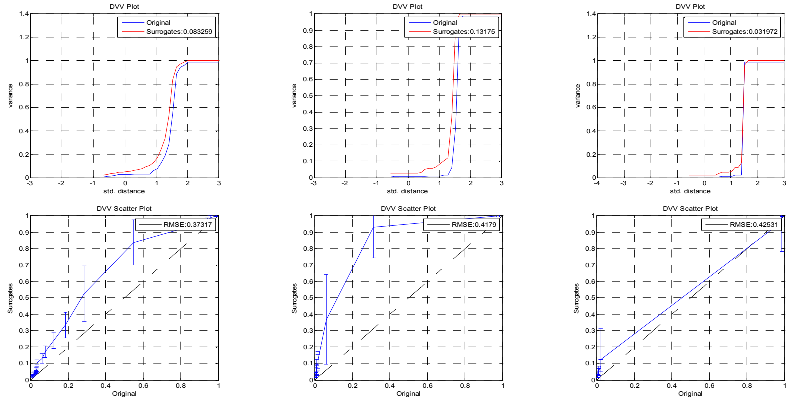

19	SYSTEM	DETAILS	VARIABLES		METHOD 1				METHOD 2				METHOD 3			
				nat.freq.	best m	best τ	rmse	RMSE	calc m	τ	rmse	RMSE	set m	set τ	rmse	RMSE
19	SDOF SQUARE PULSE INPUTS VARYING NATURAL FREQUENCY	SDOF having a square wave input	system mass m=1;<br>damping ratio ζ=0.05;<br>time duration to test tf=10s;<br>force magnitude Fm=5;<br>wave starts at to=0;<br>wave stops at to=3;	wn=2	4	1	0.0311	0.2394	3	1	0.0134	0.2502	3	1	0.0173	0.2490
				wn=6	6	10	0.2304	0.1718	1	1	0.0086	0.1917	3	1	0.0058	0.1903
				wn=12	9	10	0.4009	0.2187	4	1	0.0179	0.1875	3	1	0.0100	0.1876

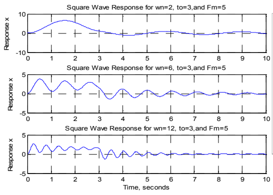

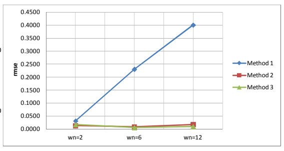

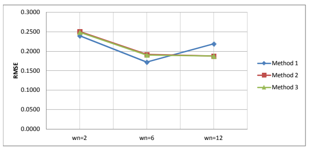

wn=2 wn=6 wn=12

METHOD 1

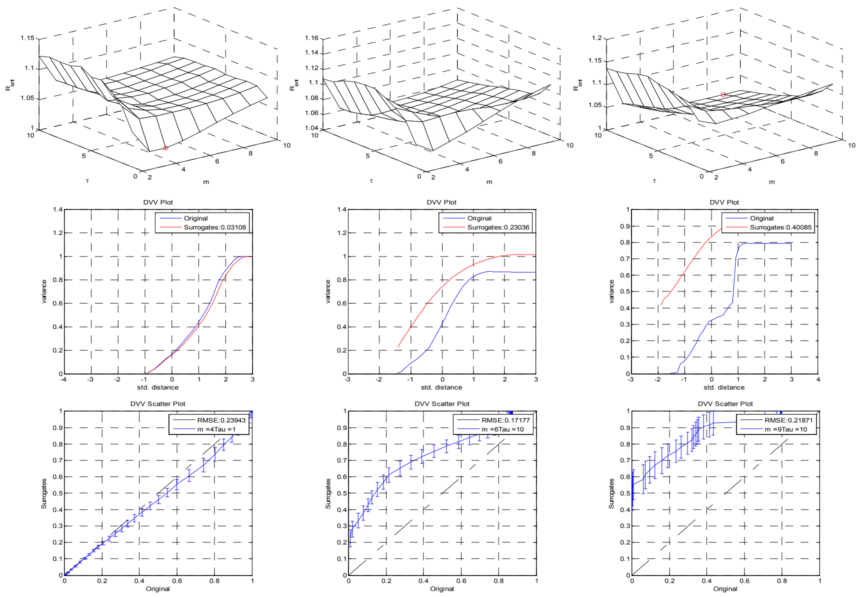

wn=2 wn=6 wn=12

METHOD 2

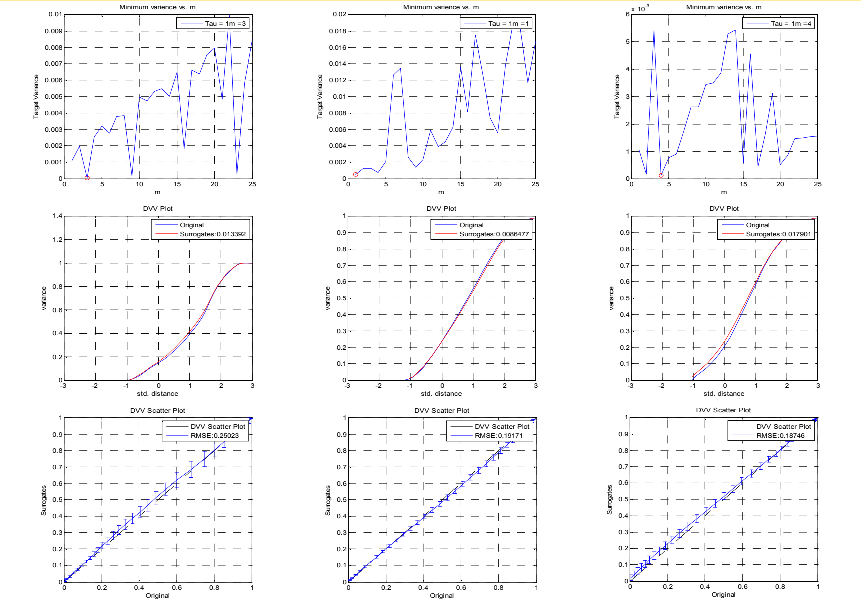

wn=2 wn=6 wn=12

METHOD 3

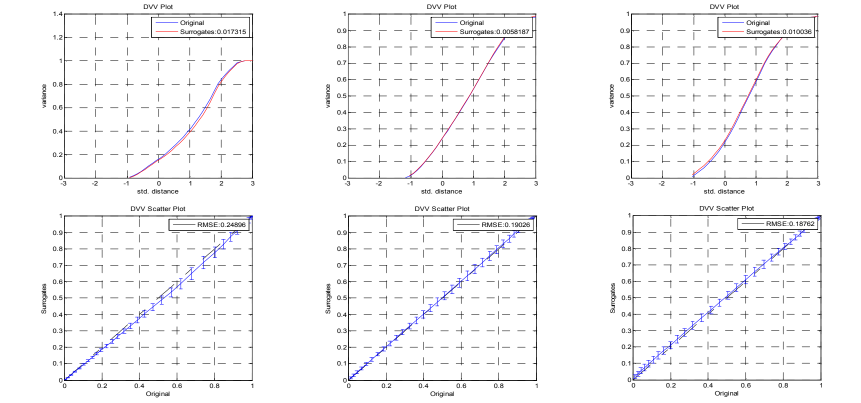

	SYSTEM	DETAILS	VARIABLES	damping ra	METHOD 1 best m	best τ	rmse	RMSE	METHOD 2 calc m	τ	rmse	RMSE	METHOD 3 set m	set τ	rmse	RMSE
20	SDOF SQUARE PULSE INPUTS VARYING DAMPING RATIO	SDOF having a square wave input	system mass m=1;<br>natural frequency wn=12;<br>time duration to test tf=10s;<br>force magnitude Fm=7;<br>wave starts at to=0;<br>wave stops at to=3;	ζ=0.05	9	10	0.4009	0.2187	19	1	0.0359	0.1428	3	1	0.0095	0.1886
				ζ=0.1	10	10	0.4237	0.2176	3	1	0.0247	0.2544	3	1	0.0236	0.2544
				ζ=0.3	5	5	0.1207	0.2798	10	1	0.0270	0.3248	3	1	0.0085	0.3409

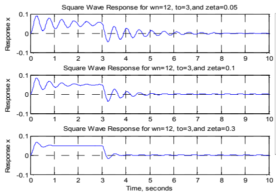

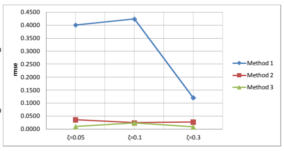

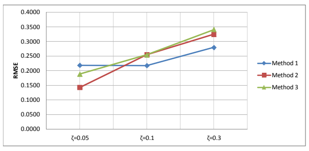

ζ=0.05 ζ=0.1 ζ=0.3

METHOD 1

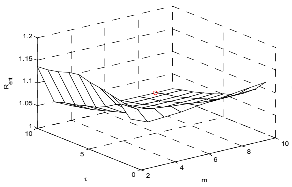
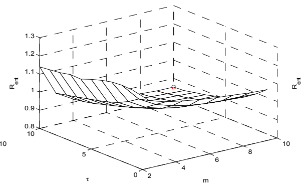
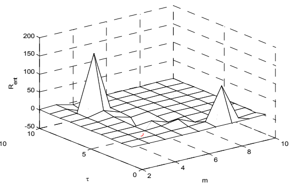

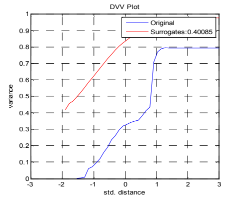

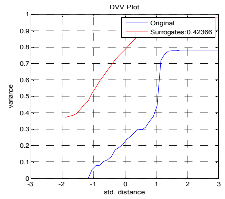

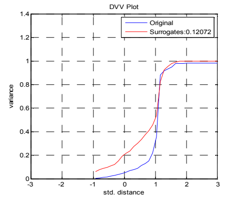

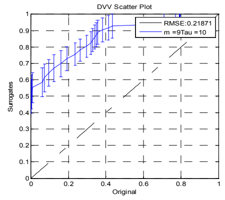

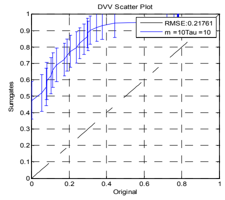

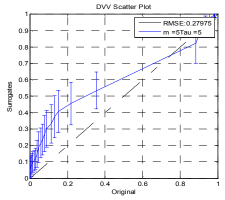

ζ=0.05 ζ=0.1 ζ=0.3

METHOD 2

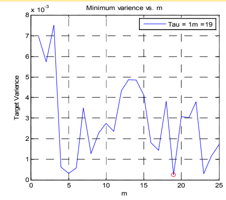

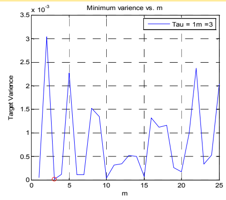

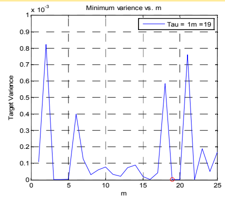

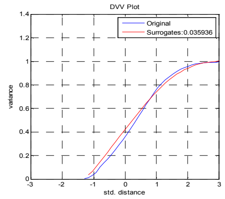

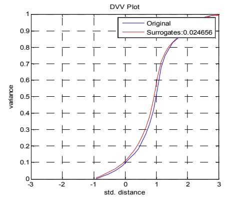

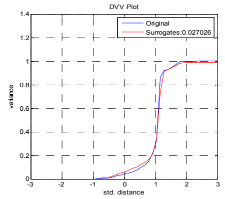

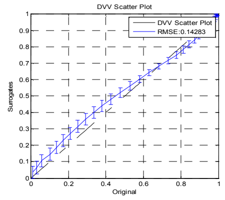

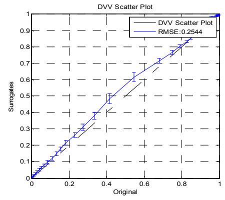

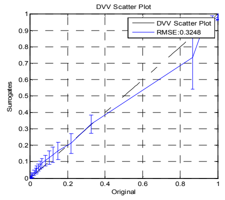

ζ=0.05 ζ=0.1 ζ=0.3

METHOD 3

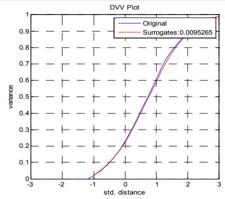

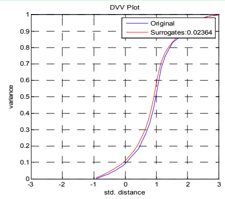

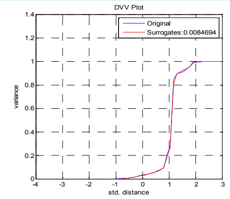

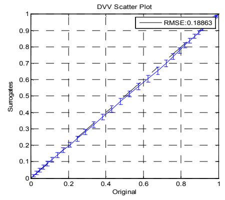

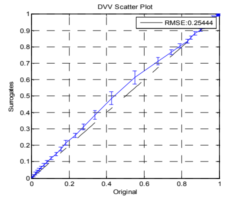

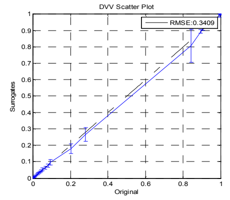

	SYSTEM	DETAILS	VARIABLES		METHOD 1				METHOD 2				METHOD 3			
				force magnit	best m	best τ	rmse	RMSE	calc m	τ	rmse	RMSE	set m	set τ	rmse	RMSE
21	SDOF SQUARE PULSE INPUTS VARYING FORCE MAGNITUDE	SDOF having a square wave input	system mass m=1;<br>natural frequency wn=12;<br>time duration to test tf=10s;<br>damping ratio ζ=0.05;<br>wave starts at to=0;<br>wave stops at to=3;	Fm=3	9	10	0.4009	0.2187	5	1	0.0051	0.1798	3	1	0.0074	0.1882
				Fm=7	9	10	0.4009	0.2187	3	1	0.0053	0.1906	3	1	0.0052	0.1892
				Fm=11	9	10	0.4009	0.2187	3	1	0.0130	0.1908	3	1	0.0096	0.1880

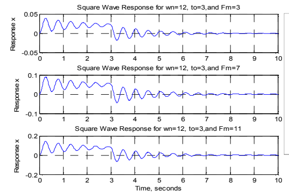

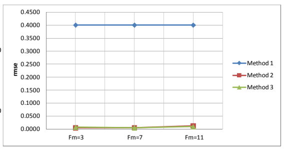

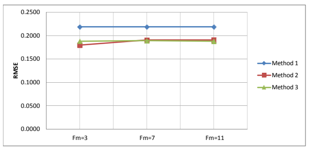

Fm=3 Fm=7 Fm=11

METHOD 1

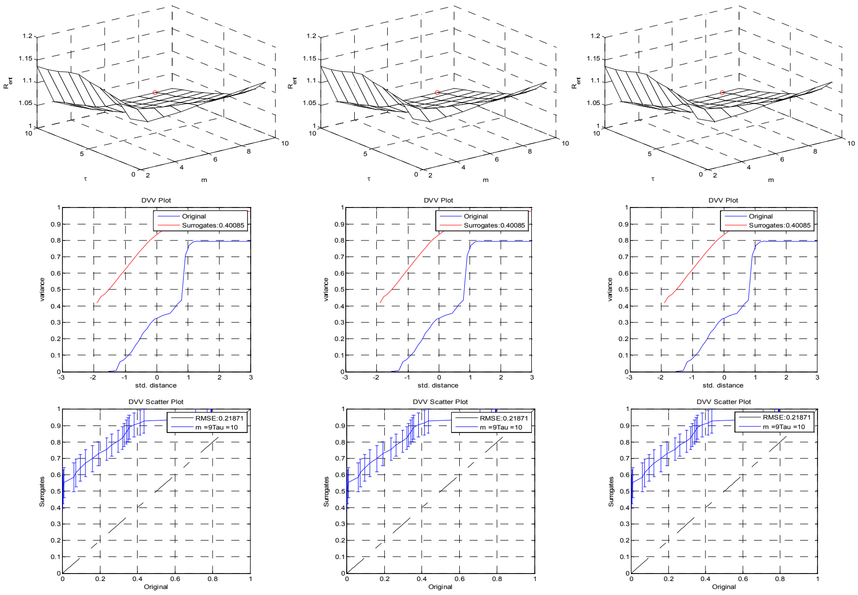

Fm=3 Fm=7 Fm=11

METHOD 2

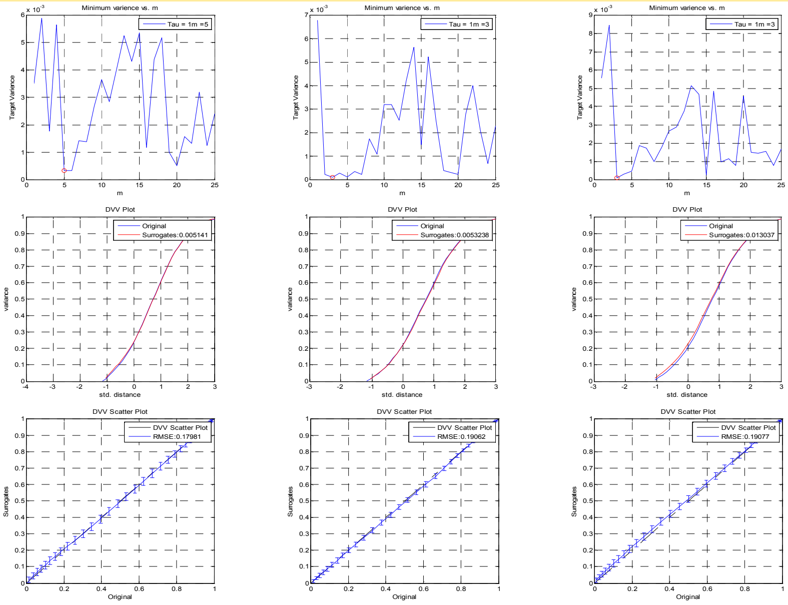

Fm=3 Fm=7 Fm=11

METHOD 3

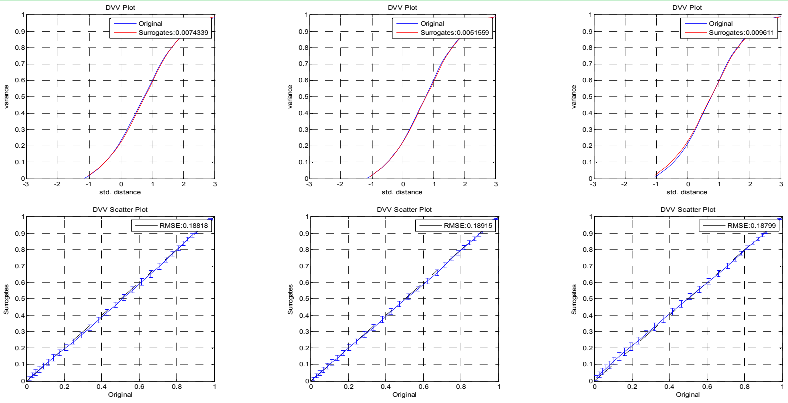

	SYSTEM	DETAILS	VARIABLES		METHOD 1				METHOD 2				METHOD 3			
				force magnit	best m	best τ	rmse	RMSE	calc m	τ	rmse	RMSE	set m	set τ	rmse	RMSE
22	SDOF RAMP INPUT VARYING RATE OF LOADING	SDOF having a ramp response external force F(t)=fo*t levels off at F=fo*te until infnity	system mass m=1; natural frequency wn=6; k=wn^2; wd=wn*sqrt(1-zeta^2); damping ratio ζ=0.05; force starts at to=0; force levels at at te=4;	fo=3	5	1	0.0442	0.1861	11	1	0.0183	0.1864	3	1	0.0101	0.1982
				fo=7	5	1	0.0442	0.1861	4	1	0.0107	0.1978	3	1	0.0062	0.1977
				fo=26	5	1	0.0442	0.1861	6	1	0.0069	0.1932	3	1	0.0073	0.1980

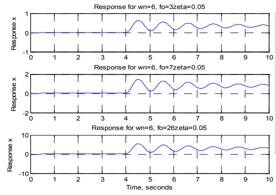

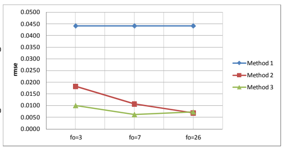

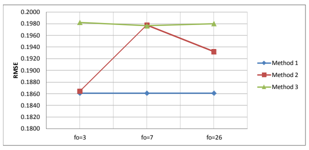

fo=3 fo=7 fo=26

METHOD 1

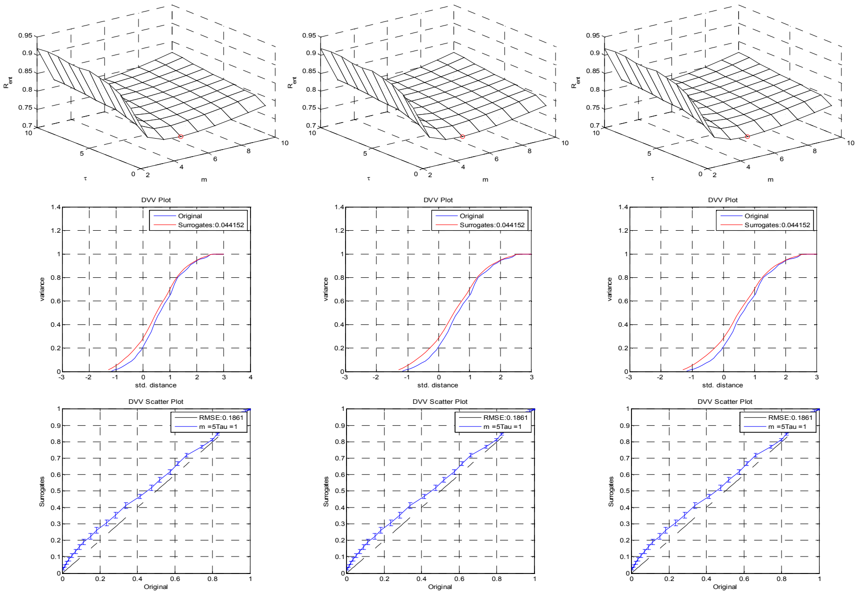

fo=3 fo=7 fo=26

METHOD 2

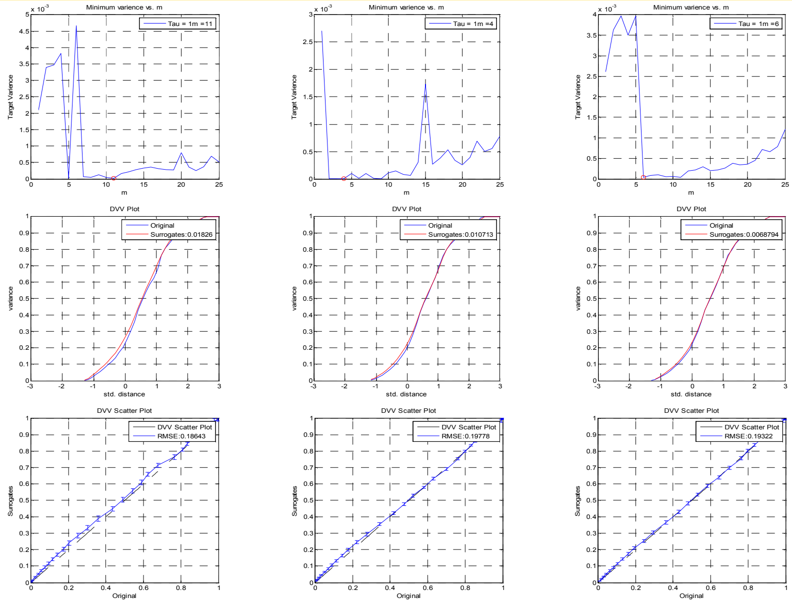

fo=3 fo=7 fo=26

METHOD 3

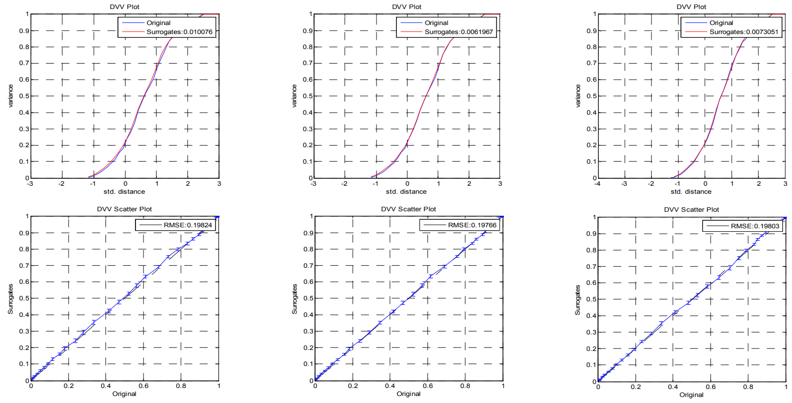

	SYSTEM	DETAILS	VARIABLES		METHOD 1				METHOD 2				METHOD 3			
				signal	best m	best τ	rmse	RMSE	calc m	τ	rmse	RMSE	set m	set τ	rmse	RMSE
23	VAN DER POL OSCILLATOR	van der Pol oscillator expression, x''+e(x^2-1)x'+x=0	e =0.5; initial conditions x0=[1; 0]; x(0)=1, x'(0)=0 time interval tf=30;	disp	2	1	0.0063	0.1667	3	1	0.0078	0.1534	3	1	0.0113	0.1554
				vel	2	1	0.1088	0.1082	1	1	0.0804	0.1218	3	1	0.1344	0.1136

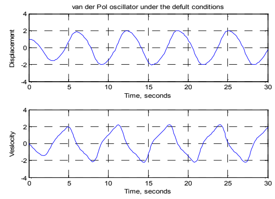

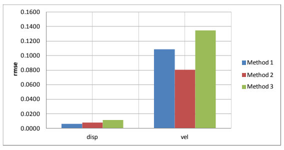

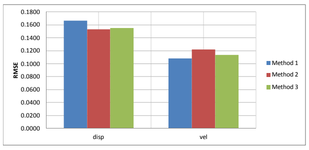

disp vel

METHOD 1

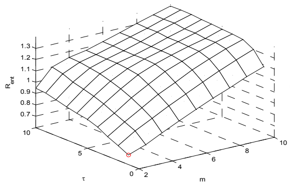

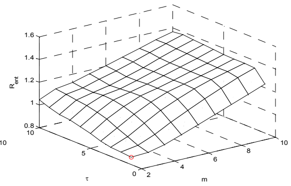

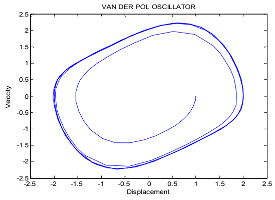

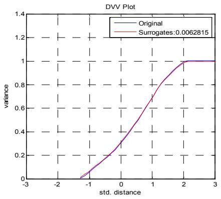

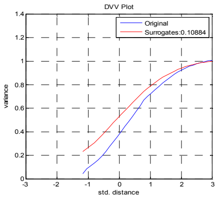

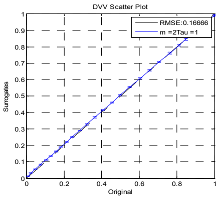

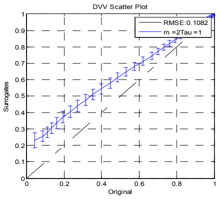

disp vel

METHOD 2

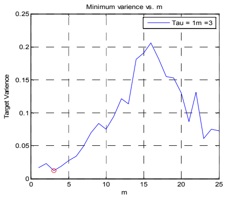

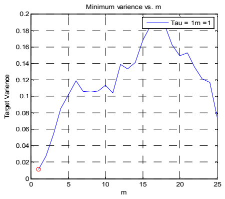

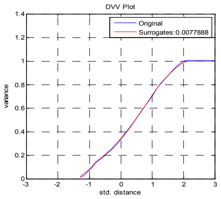

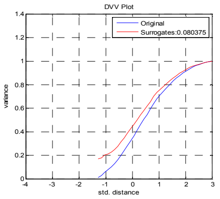

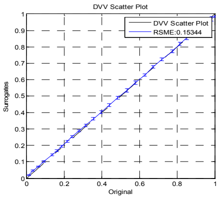

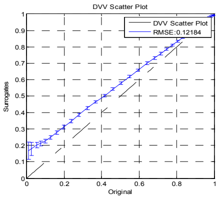

disp vel

METHOD 3

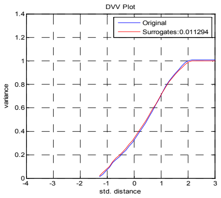

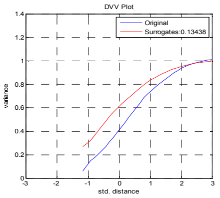

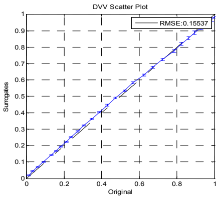

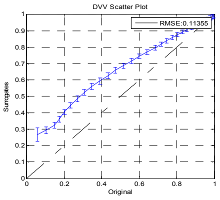

	SYSTEM	DETAILS	VARIABLES		METHOD 1				METHOD 2				METHOD 3			
				case	best **m**	best **τ**	rmse	RMSE	calc **m**	**τ**	rmse	RMSE	set **m**	set **τ**	rmse	RMSE
	SDOF RANDOM VIBRATION	input: F(t)=Acos(omega*t+phi),	A is a random variable; distflag should be zero for uniform, and 1 for Gaussian distribution	case 1	4	1	0.0084	0.1541	18	1	0.0418	0.1220	3	1	0.0070	0.1556
24			A=[0 to 5] for the uniform distribution, and as a distribution with mean of 2.5 for the Gaussian (won't be rigorously Gaussian).	case 2	2	1	0.0015	0.1560	1	1	0.0224	0.1534	3	1	0.0366	0.1391

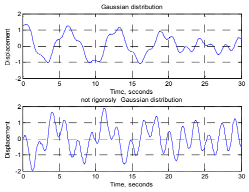

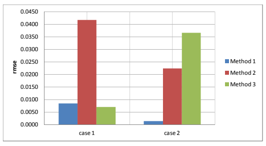

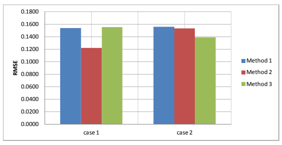

case 1 case 2

METHOD 1

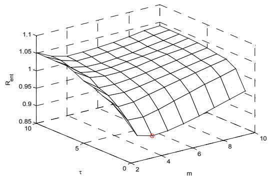

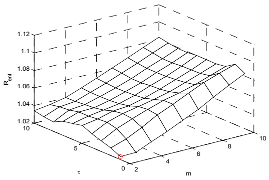

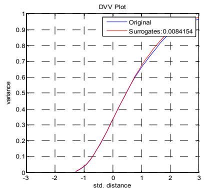

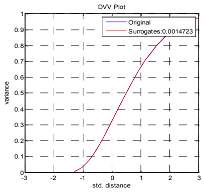

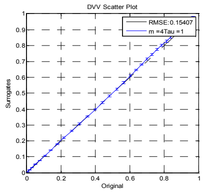

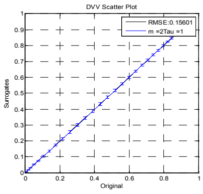

case 1 case 2

METHOD 2

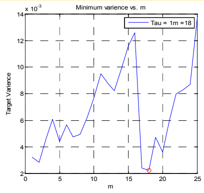

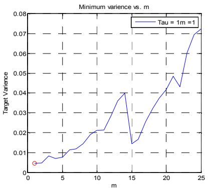

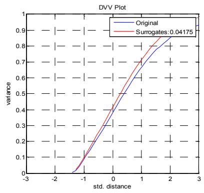

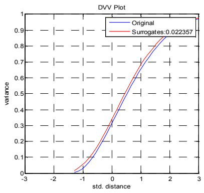

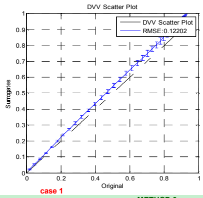

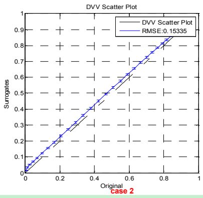

case 1 case 2

METHOD 3

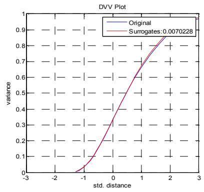

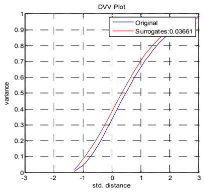

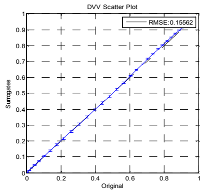

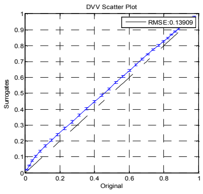

	SYSTEM	DETAILS	VARIABLES	case	METHOD 1 best m	METHOD 1 best τ	METHOD 1 rmse	METHOD 1 RMSE	METHOD 2 calc m	METHOD 2 τ	METHOD 2 rmse	METHOD 2 RMSE	METHOD 3 set m	METHOD 3 set τ	METHOD 3 rmse	METHOD 3 RMSE
25	SDOF RANDOMLY EXCITED DUFFING OSCILLATOR	Duffing oscillator	tspan=[0 30];<br>xinit=[0 0]'; e=0.01;<br>c=0.05;<br>k=1;<br>A=3.7999;<br>w=3.7960; chosen to prevent resonance	dis	3	1	0.0523	0.1252	1	1	0.0187	0.1435	3	1	0.0545	0.1252
				vel	4	1	0.0910	0.1316	10	1	0.1201	0.1712	3	1	0.0703	0.1365

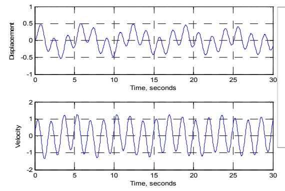

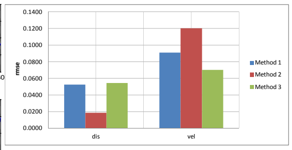

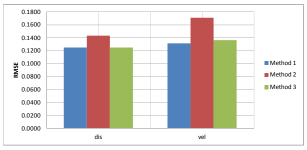

dis | vel

METHOD 1

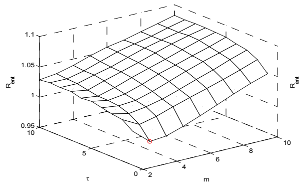

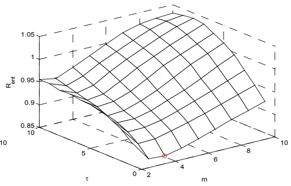

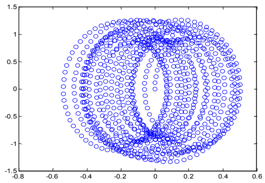

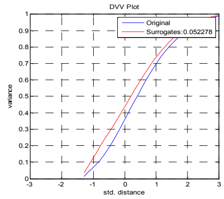

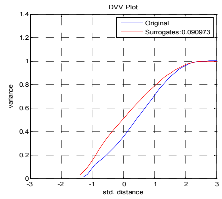

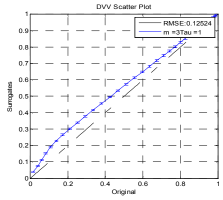

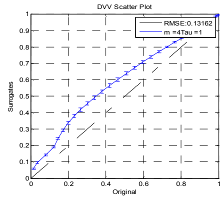

dis | vel

METHOD 2

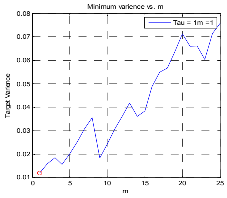

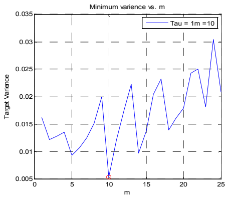

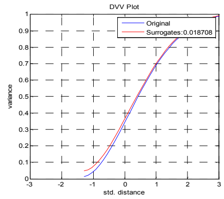

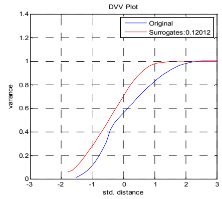

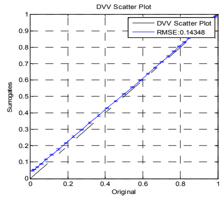

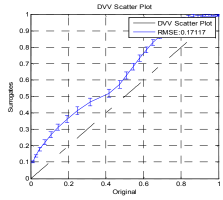

dis | vel

METHOD 3

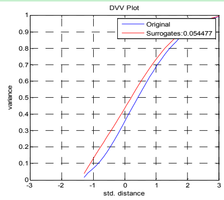

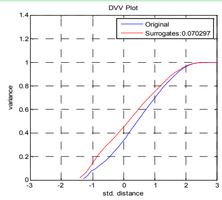

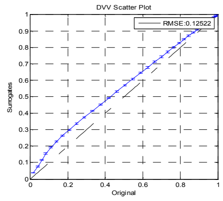

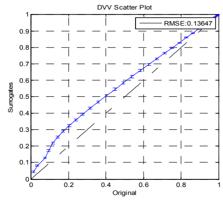

	SYSTEM	DETAILS	VARIABLES	case	METHOD 1 best m	METHOD 1 best τ	METHOD 1 rmse	METHOD 1 RMSE	METHOD 2 calc m	METHOD 2 τ	METHOD 2 rmse	METHOD 2 RMSE	METHOD 3 set m	METHOD 3 set τ	METHOD 3 rmse	METHOD 3 RMSE
26	SDOF RANDOMLY EXCITED DUFFING OSCILLATOR	Duffing oscillator	tspan=[0 30]; xinit=[0 0]'; e=0.01; c=0.05; k=1; A=4.4351; w=1.7404; chosen to prevent resonance	dis	6	1	0.0332	0.1452	16	1	0.0106	0.1342	3	1	0.0090	0.1573
				vel	5	1	0.0266	0.1450	20	1	0.0965	0.1187	3	1	0.0269	0.1548

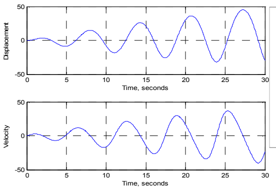

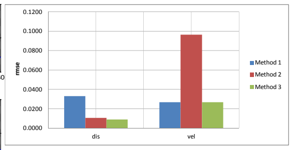

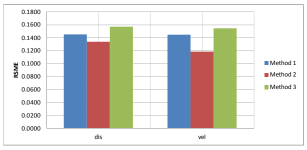

METHOD 1

dis | vel

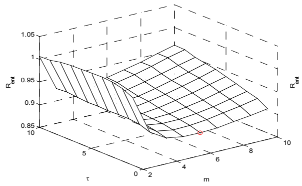

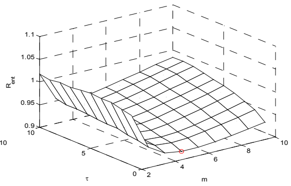

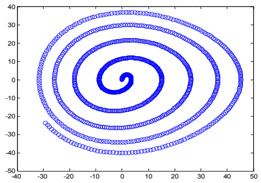

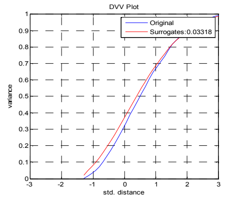

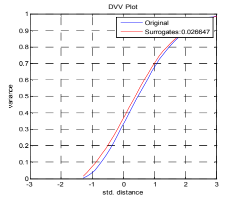

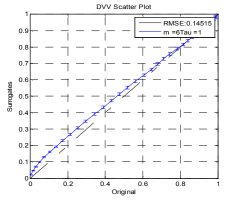

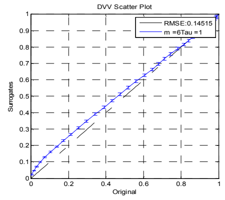

METHOD 2

dis | vel

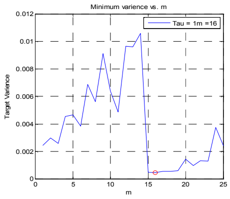

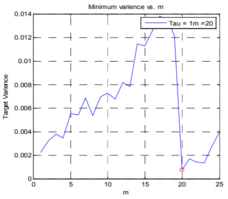

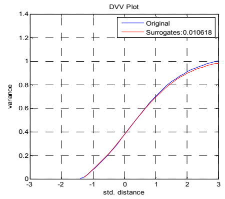

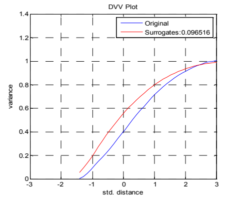

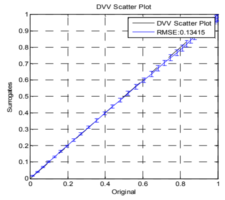

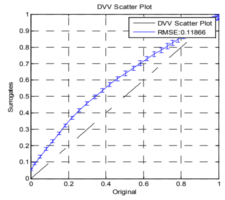

METHOD 3

dis | vel

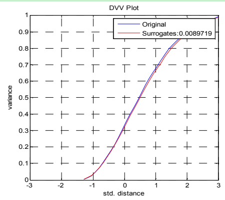

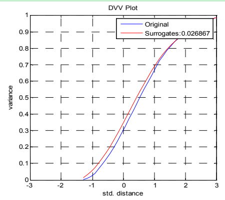

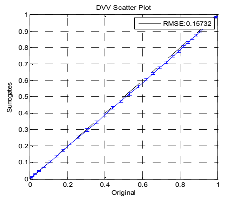

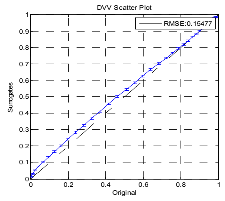

	SYSTEM	DETAILS	VARIABLES	case	METHOD 1 best m	METHOD 1 best τ	METHOD 1 rmse	METHOD 1 RMSE	METHOD 2 calc m	METHOD 2 τ	METHOD 2 rmse	METHOD 2 RMSE	METHOD 3 set m	METHOD 3 set τ	METHOD 3 rmse	METHOD 3 RMSE
27	SDOF RANDOMLY EXCITED DUFFING OSCILLATOR	Duffing oscillator	tspan=[0 30];<br>xinit=[0 0]'; e=0.01;<br>c=0.05;<br>k=1;<br>A=1.0062;<br>w=2.0115; chosen to prevent resonance	dis	4	1	0.0376	0.1507	1	1	0.0153	0.1656	3	1	0.0379	0.1579
				vel	3	1	0.0245	0.1484	10	1	0.0079	0.1318	3	1	0.0035	0.1560

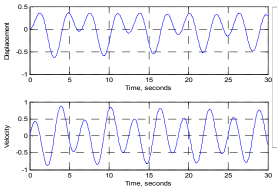

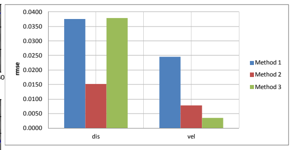

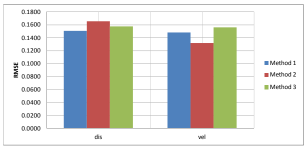

dis | vel

METHOD 1

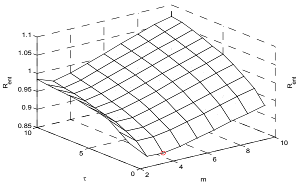

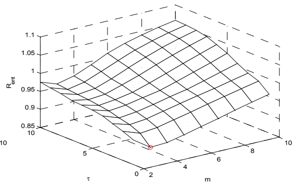

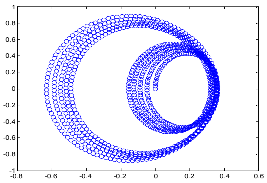

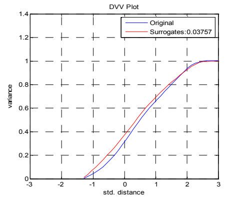

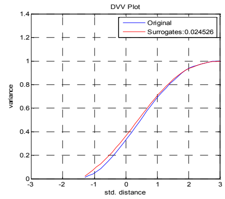

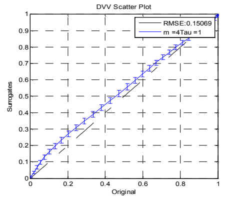

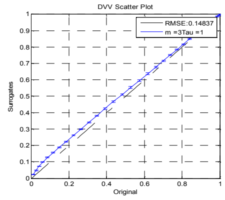

dis | vel

METHOD 2

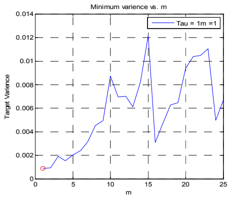

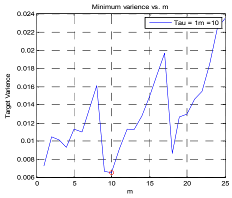

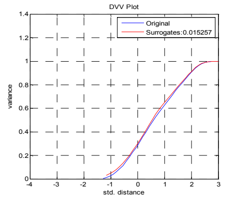

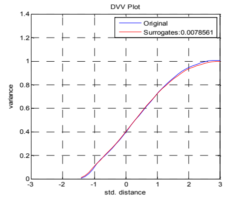

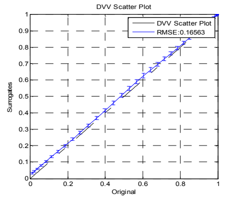

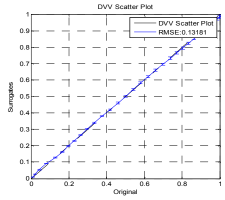

dis | vel

METHOD 3

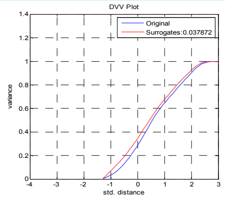

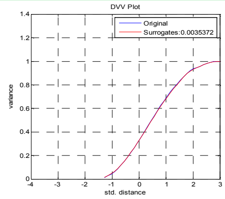

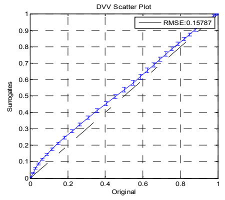

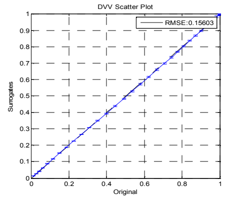

Supplement: APPENDIX 3 [file rsos150493supp3.pdf]
