# Supplementary material for: A comprehensive study of the delay vector variance method for quantification of nonlinearity in dynamical systems
Source: R Soc Open Sci. 2016 Jan 6;3(1):150493. doi: 10.1098/rsos.150493 (PMC4736930; doi:10.1098/rsos.150493)

APPENDIX 4

Delay Vector Variance Method SDOF Car Experiment Results

	SYSTEM	VARIABLES		METHOD 1				METHOD 2				METHOD 3			
				best m	best τ	rsme	RSME	calc m	τ	rsme	RSME	calc m	τ	rsme	RSME
1	SDOF CAR attached to fixed supports by 6 calibrated springs (3 on each side)	surface wood; number of springs 2 x 3; loading harmonic 2Hz	CH1	5	10	0.3538	0.3800	19	1	0.1669	0.1712	3	1	0.0760	0.1182
			CH2	2	3	0.2853	0.3461	9	1	0.2991	0.3017	3	1	0.2429	0.2713
			CH3	2	4	0.0071	0.4538	21	1	0.0375	0.5017	3	1	0.0088	0.4579
			LDVg	5	1	0.0861	0.1384	6	1	0.0097	0.1256	3	1	0.0763	0.1186
			LDV1	3	10	0.0562	0.1599	20	1	0.0519	0.1932	3	1	0.0073	0.1467
2	SDOF CAR attached to fixed supports by 6 calibrated springs (3 on each side)	surface wood; number of springs 2 x 3; loading harmonic 2-4-6-8-10 Hz	CH1	5	9	0.2918	0.1763	17	1	0.1821	0.1349	3	1	0.1537	0.1416
			CH2	10	1	0.4714	0.3659	15	1	0.5078	0.3839	3	1	0.3069	0.3176
			CH3	9	1	0.4371	0.2938	24	1	0.5226	0.3441	3	1	0.2395	0.2839
			LDVg	8	5	0.2085	0.1758	5	1	0.0277	0.1421	3	1	0.0303	0.1386
			LDV1	6	5	0.4141	0.2822	21	1	0.3169	0.2037	3	1	0.2106	0.1716
3	SDOF CAR attached to fixed supports by 6 calibrated springs (3 on each side)	surface plastic (smooth) number of springs 2 x 3; loading harmonic 2-4-6-8-10 Hz	CH1	4	10	0.2571	0.1755	11	1	0.1748	0.1235	3	1	0.2326	0.1279
			CH2	10	1	0.4215	0.2985	14	1	0.4728	0.3259	3	1	0.2326	0.2729
			CH3	10	1	0.3278	0.2016	19	1	0.4009	0.2342	3	1	0.1274	0.2131
			LDVg	9	5	0.2006	0.1526	15	1	0.0743	0.1285	3	1	0.0336	0.1340
			LDV1	6	5	0.4473	0.3014	5	1	0.2631	0.1786	3	1	0.1406	0.1675
4	SDOF CAR attached to fixed supports by 6 calibrated springs (3 on each side)	surface wood number of springs 2 x 3; loading Sine Sweep	CH1	4	10	0.3245	0.1879	25	1	0.2112	0.1329	3	1	0.1167	0.1327
			CH2	10	2	0.4291	0.3725	22	1	0.4903	0.3679	3	1	0.2855	0.3344
			CH3	9	1	0.4660	0.2979	13	1	0.4939	0.3062	3	1	0.2418	0.2878
			LDVg	10	3	0.3765	0.2964	17	1	0.0839	0.1140	3	1	0.0320	0.1272
			LDV1	9	4	0.3671	0.2911	11	1	0.2483	0.1759	3	1	0.1722	0.1654
5	SDOF CAR attached to fixed supports by 6 calibrated springs (3 on each side)	surface wood number of springs 2 x 3; loading White Noise	CH1	7	1	0.1448	0.1130	9	1	0.1392	0.1295	3	1	0.1344	0.1141
			CH2	6	1	0.2556	0.3944	14	1	0.3764	0.3937	3	1	0.1444	0.3790
			CH3	7	1	0.3480	0.3566	10	1	0.3926	0.3682	3	1	0.1853	0.3566
			LDVg	6	1	0.0703	0.2926	10	1	0.1983	0.4177	3	1	0.2496	0.3423
			LDV1	3	1	0.0052	0.1550	3	1	0.1789	0.2018	3	1	0.1805	0.2045
6	SDOF CAR attached to fixed supports by 6 calibrated springs (3 on each side)	surface plastic (smooth) number of springs 2 x 3; loading Sine Sweep	CH1	4	10	0.2964	0.1693	23	1	0.1757	0.1291	3	1	0.1165	0.1448
			CH2	10	2	0.3993	0.3150	16	1	0.3979	0.2878	3	1	0.2521	0.2645
			CH3	10	1	0.3018	0.1605	14	1	0.2986	0.1663	3	1	0.1607	0.1612
			LDVg	10	6	0.2407	0.2245	6	1	0.0417	0.1360	3	1	0.0289	0.1296
			LDV1	8	10	0.4237	0.3596	6	1	0.2396	0.1734	3	1	0.1724	0.1652
7	SDOF CAR attached to fixed supports by 6 calibrated springs (3 on each side)	surface plastic (smooth) number of springs 2 x 3; loading White Noise	CH1	6	1	0.1415	0.1096	13	1	0.1231	0.1480	3	1	0.1145	0.1007
			CH2	6	1	0.3002	0.3599	6	1	0.3477	0.3946	3	1	0.1855	0.3481
			CH3	7	1	0.2142	0.1943	14	1	0.2655	0.2077	3	1	0.0841	0.2121
			LDVg	6	1	0.2506	0.3666	4	1	0.2516	0.3528	3	1	0.2431	0.3397
			LDV1	2	3	0.2555	0.2630	3	1	0.1849	0.2014	3	1	0.1942	0.2013
8	SDOF CAR attached to fixed supports by 6 calibrated springs (3 on each side)	surface sand paper (rough) number of springs 2 x 3; loading White Noise	CH1	6	1	0.1395	0.1110	5	1	0.1363	0.1030	3	1	0.1236	0.0964
			CH2	3	8	0.0632	0.4353	12	1	0.3188	0.4012	3	1	0.1377	0.3934
			CH3	4	4	0.0432	0.3464	7	1	0.1167	0.2232	3	1	0.0657	0.2730
			LDVg	6	1	0.1901	0.4007	9	1	0.1931	0.4276	3	1	0.1886	0.3663
			LDV1	5	1	0.1922	0.1463	17	1	0.2249	0.2143	3	1	0.1933	0.2026
9	SDOF CAR attached to fixed supports by 6 calibrated springs (3 on each side)	surface sand paper (rough) number of springs 2 x 3; loading Sine Sweep	CH1	5	9	0.2844	0.1682	4	1	0.1280	0.1360	3	1	0.1015	0.1308
			CH2	10	1	0.4205	0.3643	7	1	0.3815	0.3498	3	1	0.2444	0.3234
			CH3	10	1	0.3147	0.1674	18	1	0.3119	0.1823	3	1	0.1763	0.1581
			LDVg	10	3	0.1551	0.1549	15	1	0.0677	0.1084	3	1	0.0391	0.1260
			LDV1	9	4	0.3675	0.2888	22	1	0.2944	0.2135	3	1	0.1745	0.1623
10	SDOF CAR attached to fixed supports by 6 calibrated springs (3 on each side)	surface sand paper (rough) number of springs 2 x 3; loading harmonic 2-4-6-8-10 Hz	CH1	3	9	0.2816	0.1670	14	1	0.1980	0.1349	3	1	0.1458	0.1328
			CH2	8	1	0.3732	0.3401	11	1	0.4204	0.3552	3	1	0.2067	0.3402
			CH3	8	1	0.3422	0.1905	12	1	0.3237	0.2037	3	1	0.1902	0.2038
			LDVg	8	5	0.2355	0.1912	11	1	0.0542	0.1181	3	1	0.0303	0.1316
			LDV1	6	5	0.2460	0.1848	12	1	0.2924	0.1828	3	1	0.2012	0.1681
11	SDOF CAR attached to fixed supports by 4 calibrated springs (2 on each side)	surface sand paper (rough) Middle spring taken out number of springs 2 x2; loading harmonic 2-4-6-8-10 Hz	CH1	4	10	0.3958	0.2010	10	1	0.2735	0.1514	3	1	0.2079	0.1548
			CH2	10	1	0.6419	0.3674	20	1	0.6193	0.3486	3	1	0.5733	0.3291
			CH3	10	1	0.2980	0.1626	19	1	0.2564	0.1484	3	1	0.3044	0.1883
			LDVg	8	6	0.2343	0.1855	14	1	0.0675	0.1175	3	1	0.0235	0.1102
			LDV1	7	5	0.2680	0.1687	14	1	0.1438	0.1134	3	1	0.0710	0.0978
12	SDOF CAR attached to fixed supports by 4 calibrated springs (2 on each side)	surface sand paper (rough) Middle spring taken out number of springs 2 x2; loading Sine Sweep	CH1	4	10	0.2691	0.1546	9	1	0.1486	0.1102	3	1	0.1325	0.1415
			CH2	10	3	0.5232	0.2775	15	1	0.5250	0.2838	3	1	0.4094	0.2149
			CH3	10	1	0.3269	0.1693	14	1	0.3390	0.1803	3	1	0.1057	0.1350
			LDVg	8	3	0.1022	0.1263	8	1	0.0302	0.1164	3	1	0.0276	0.1029
			LDV1	7	7	0.1367	0.1063	24	1	0.0805	0.1195	3	1	0.0141	0.0981
13	SDOF CAR attached to fixed supports by 4 calibrated springs (2 on each side) REPEATED EXPERIMENT NO.12	surface sand paper (rough) Middle spring taken out number of springs 2 x2; loading Sine Sweep	CH1	4	10	0.2932	0.1736	19	1	0.1795	0.1250	3	1	0.1026	0.1393
			CH2	10	3	0.4319	0.3034	13	1	0.4338	0.2673	3	1	0.2973	0.2284
			CH3	9	1	0.2023	0.1111	15	1	0.2177	0.1216	3	1	0.1123	0.0981
			LDVg	8	3	0.1056	0.1260	20	1	0.0875	0.1072	3	1	0.0357	0.1047
			LDV1	7	5	0.1701	0.1632	12	1	0.2371	0.1559	3	1	0.1701	0.1410
14	SDOF CAR attached to fixed supports by 4 calibrated springs (2 on each side)	surface sand paper (rough) Middle spring taken out number of springs 2 x2; loading White Noise	CH1	6	1	0.1597	0.1052	21	1	0.1710	0.1335	3	1	0.1340	0.1064
			CH2	5	1	0.3213	0.3289	14	1	0.4115	0.3509	3	1	0.2359	0.3252
			CH3	3	6	0.0762	0.2905	25	1	0.1015	0.1225	3	1	0.0583	0.1759
			LDVg	6	1	0.0144	0.2840	4	1	0.1934	0.3564	3	1	0.2385	0.3479
			LDV1	4	1	0.2109	0.1760	8	1	0.2282	0.1448	3	1	0.1994	0.1780

**15**	SDOF CAR attached to fixed supports by 4 calibrated springs (2 on each side)	surface plastic (smooth) Middle spring taken out number of springs 2 x2; loading White Noise	CH1	4	1	0.1428	0.1078	7	1	0.1520	0.1053	3	1	0.1312	0.1118
			CH2	4	5	0.1913	0.3615	19	1	0.3416	0.2899	3	1	0.1730	0.2628
			CH3	8	1	0.2986	0.3386	10	1	0.2631	0.2176	3	1	0.1307	0.2297
			LDVg	6	1	0.0212	0.2731	9	1	0.1961	0.4149	3	1	0.2533	0.3451
			LDV1	2	1	0.1941	0.1550	11	1	0.2138	0.1470	3	1	0.1862	0.1727
**16**	SDOF CAR attached to fixed supports by 4 calibrated springs (2 on each side)	surface plastic (smooth) Middle spring taken out number of springs 2 x2; loading Sine Sweep	CH1	5	9	0.2767	0.1552	17	1	0.1652	0.1195	3	1	0.0990	0.1493
			CH2	10	2	0.3390	0.2481	13	1	0.3118	0.2162	3	1	0.2198	0.2020
			CH3	10	1	0.3098	0.1654	18	1	0.3506	0.1856	3	1	0.1513	0.1137
			LDVg	8	3	0.0936	0.1236	19	1	0.0688	0.1058	3	1	0.0306	0.1046
			LDV1	8	5	0.1726	0.1714	21	1	0.2837	0.1924	3	1	0.1688	0.1401
**17**	SDOF CAR attached to fixed supports by 4 calibrated springs (2 on each side)	surface plastic (smooth) Middle spring taken out number of springs 2 x2; loading harmonic 2-4-6-8-10 Hz	CH1	~~0~~	~~0~~	~~0.0000~~	~~0.0000~~	~~0~~	~~1~~	~~0.0000~~	~~0.0000~~	~~3~~	~~1~~	~~0.0000~~	~~0.0000~~
			CH2	~~0~~	~~0~~	~~0.0000~~	~~0.0000~~	~~0~~	~~1~~	~~0.0000~~	~~0.0000~~	~~3~~	~~1~~	~~0.0000~~	~~0.0000~~
			CH3	~~0~~	~~0~~	~~0.0000~~	~~0.0000~~	~~0~~	~~1~~	~~0.0000~~	~~0.0000~~	~~3~~	~~1~~	~~0.0000~~	~~0.0000~~
			LDVg	8	7	0.4169	0.2947	9	1	0.0295	0.1135	3	1	0.0213	0.1132
			LDV1	6	6	0.3819	0.2660	11	1	0.2797	0.1535	3	1	0.2075	0.1473
**18**	SDOF CAR attached to fixed supports by 4 calibrated springs (2 on each side)	surface wood Middle spring taken out number of springs 2 x2; loading harmonic 2-4-6-8-10 Hz	CH1	4	10	0.3734	0.1949	2	1	0.2415	0.1973	3	1	0.2609	0.2021
			CH2	9	1	0.6737	0.3737	25	1	0.7082	0.3989	3	1	0.6603	0.3757
			CH3	5	2	0.3453	0.1859	8	1	0.2877	0.1577	3	1	0.2672	0.1497
			LDVg	9	7	0.4325	0.3138	17	1	0.0782	0.1186	3	1	0.0362	0.1067
			LDV1	7	5	0.2536	0.1535	3	1	0.0788	0.0906	3	1	0.0824	0.0909
**19**	SDOF CAR attached to fixed supports by 4 calibrated springs (2 on each side)	surface wood Middle spring taken out number of springs 2 x2; loading White Noise	CH1	6	2	0.1763	0.1301	10	1	0.1571	0.1116	3	1	0.1344	0.1141
			CH2	4	1	0.2821	0.3305	22	1	0.4097	0.3801	3	1	0.2458	0.3314
			CH3	4	1	0.2183	0.2895	24	1	0.4016	0.3327	3	1	0.1942	0.2893
			LDVg	6	1	0.2554	0.3742	3	1	0.2775	0.3400	3	1	0.2496	0.3423
			LDV1	2	4	0.2657	0.2551	9	1	0.2023	0.1411	3	1	0.1988	0.1703
**20**	SDOF CAR attached to fixed supports by 4 calibrated springs (2 on each side)	surface wood Middle spring taken out number of springs 2 x2; loading Sine Sweep	CH1	5	10	0.2834	0.1563	10	1	0.1620	0.1213	3	1	0.1279	0.1515
			CH2	10	1	0.4544	0.3182	12	1	0.4903	0.3278	3	1	0.3329	0.2866
			CH3	4	1	0.3403	0.2314	25	1	0.5086	0.2976	3	1	0.2175	0.2418
			LDVg	9	3	0.1069	0.1251	17	1	0.0593	0.1081	3	1	0.0348	0.1059
			LDV1	8	5	0.1804	0.1634	14	1	0.2493	0.1649	3	1	0.1649	0.1388
**21**	SDOF CAR attached to fixed supports by 6 / 4 calibrated springs; Two middle springs are glued, detached at 13 sec / 38sec	surface wood Two middle springs are glued, detached at 13 sec / 38sec number of springs 2x3 / 2 x2; loading White Noise	CH1	6	1	0.1562	0.1031	6	1	0.1554	0.1043	3	1	0.1335	0.1079
			CH2	4	4	0.2074	0.3631	19	1	0.3613	0.3404	3	1	0.1947	0.3281
			CH3	3	1	0.1947	0.3281	14	1	0.3901	0.3646	3	1	0.2148	0.3271
			LDVg	6	1	0.1677	0.3935	10	1	0.0412	0.3492	3	1	0.0193	0.2759
			LDV1	2	8	0.0519	0.2460	20	1	0.0448	0.1007	3	1	0.0050	0.1372
**HF**	SDOF CAR attached to fixed supports by 4 calibrated springs	surface plastic (smooth) number of springs 2 x 3; loading High Frequency	CH1	2	8	0.1395	0.3746	24	1	0.3550	0.3235	3	1	0.2382	0.3390
			CH2	2	4	0.0564	0.4120	22	1	0.4344	0.3732	3	1	0.1624	0.3904
			CH3	2	2	0.0888	0.3616	20	1	0.4163	0.3340	3	1	0.1799	0.3568
			LDVg	5	1	0.3344	0.3091	22	1	0.4547	0.3248	3	1	0.2707	0.2848
			LDV1	2	2	0.0344	0.1209	13	1	0.2846	0.2393	3	1	0.2800	0.2289

EXPERIMENT	SYSTEM CHARACTERISTCS	VARIABLES		METHOD 1				METHOD 2				METHOD 3			
				best **m**	best τ	rsme	RSME	calc **m**	τ	rsme	RSME	set **m**	set τ	rsme	RSME
1	SDOF CAR attached to fixed supports by 6 calibrated springs (3 on each side)	surface wood; number of springs 2 x 3; loading harmonic 2Hz	CH1	5	10	0.354	0.380	19	1	0.167	0.171	3	1	0.076	0.118
			CH2	2	3	0.285	0.346	9	1	0.299	0.302	3	1	0.243	0.271
			CH3	2	4	0.007	0.454	21	1	0.038	0.502	3	1	0.009	0.458
			LDVg	5	1	0.086	0.138	6	1	0.010	0.126	3	1	0.076	0.119
			LDV1	3	10	0.056	0.160	20	1	0.052	0.193	3	1	0.007	0.147

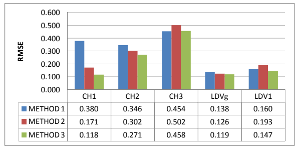

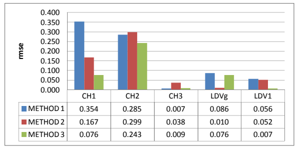

Data recorded 3D Accelerometer

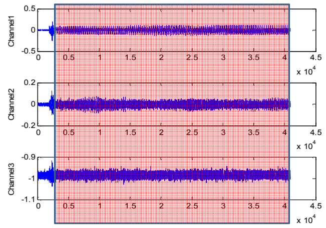

Data analysed 3D Accelerometer

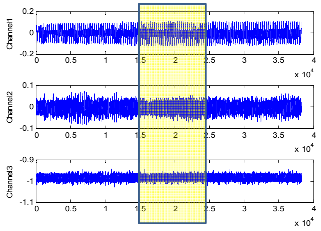

Data recorded LDV

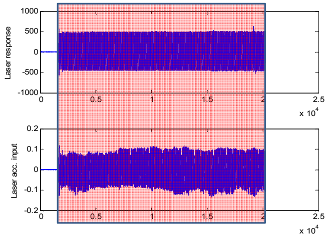

Data analysed LDV

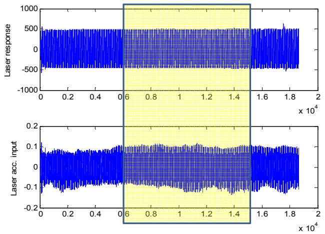

CH1 CH2 CH3 LDVg LDV1

METHOD 1

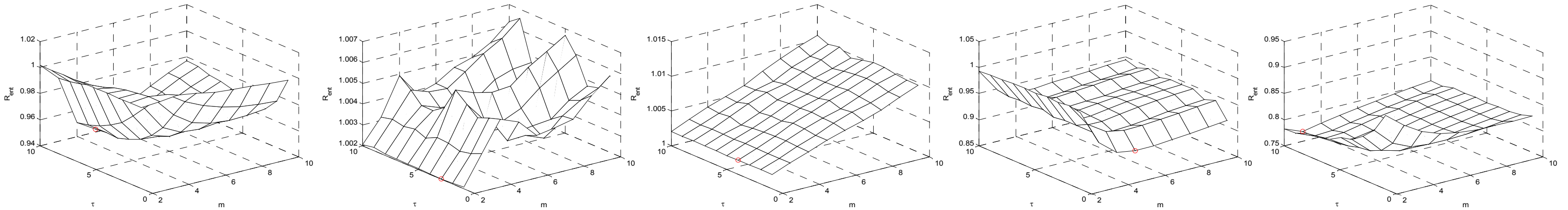

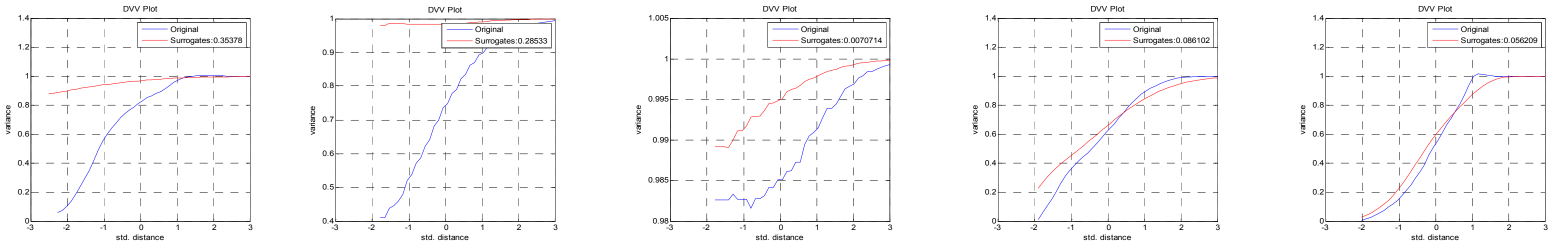

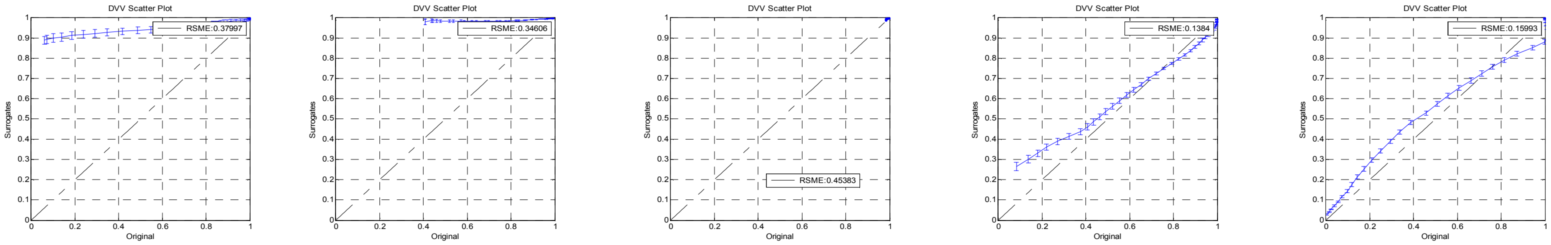

CH1 CH2 CH3 LDVg LDV1

EXP 1	METHOD 2
	CH1 / CH2 / CH3 / LDVg / LDV1

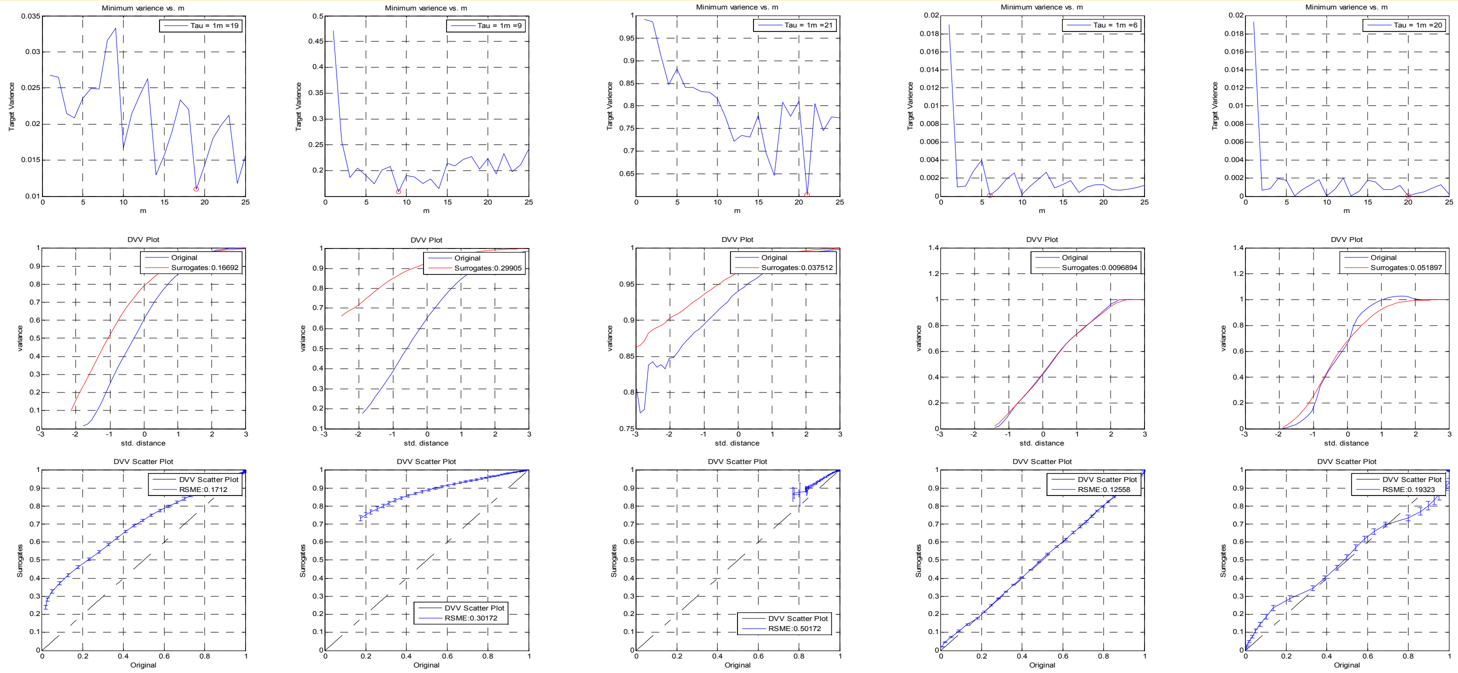

METHOD 3

CH1 / CH2 / CH3 / LDVg / LDV1

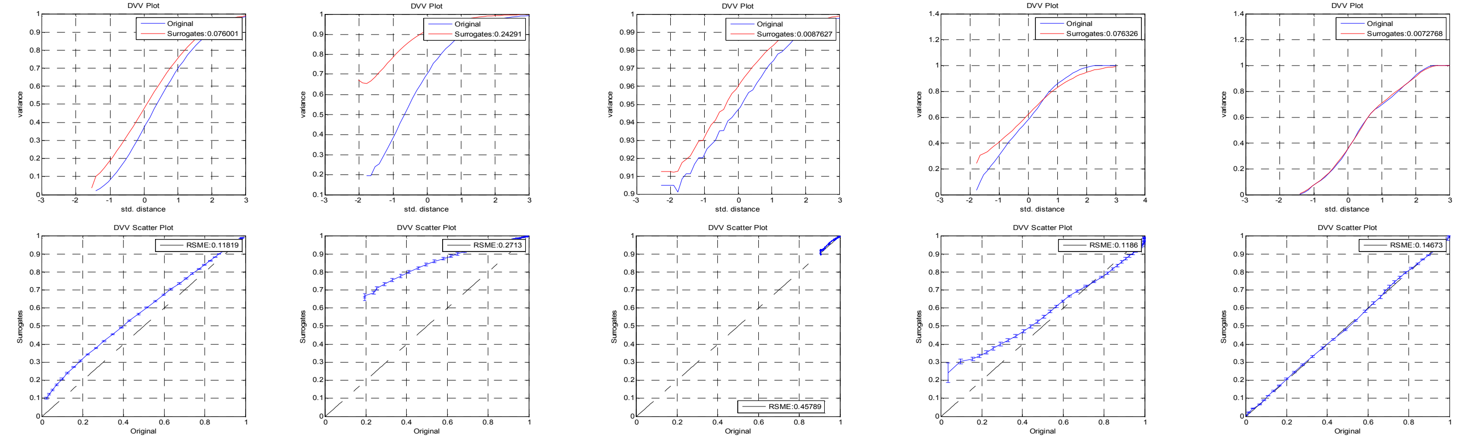

EXPERIMENT	SYSTEM CHARACTERISTCS	VARIABLES		METHOD 1				METHOD 2				METHOD 3			
				best **m**	best **τ**	rsme	RSME	calc **m**	**τ**	rsme	RSME	set **m**	set **τ**	rsme	RSME
2	SDOF CAR attached to fixed supports by 6 calibrated springs (3 on each side)	surface wood; number of springs 2 x 3; loading harmonic 2-4-6-8-10 Hz	CH1	5	9	0.292	0.176	17	1	0.182	0.135	3	1	0.154	0.142
			CH2	10	1	0.471	0.366	15	1	0.508	0.384	3	1	0.307	0.318
			CH3	9	1	0.437	0.294	24	1	0.523	0.344	3	1	0.239	0.284
			LDVg	8	5	0.208	0.176	5	1	0.028	0.142	3	1	0.030	0.139
			LDV1	6	5	0.414	0.282	21	1	0.317	0.204	3	1	0.211	0.172

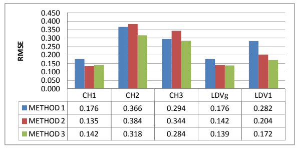

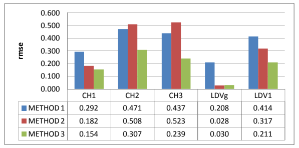

Data recorded 3D Accelerometer **Data analysed 3D Accelerometer** **Data recorded LDV** **Data analysed LDV**

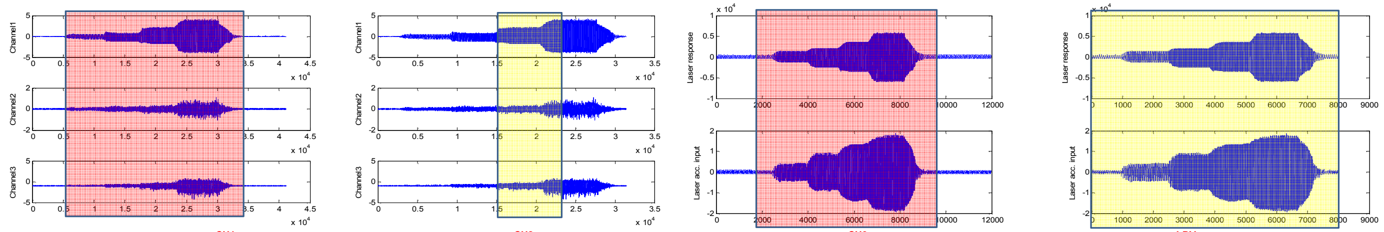

CH1 CH2 CH3 LDVg LDV1

METHOD 1

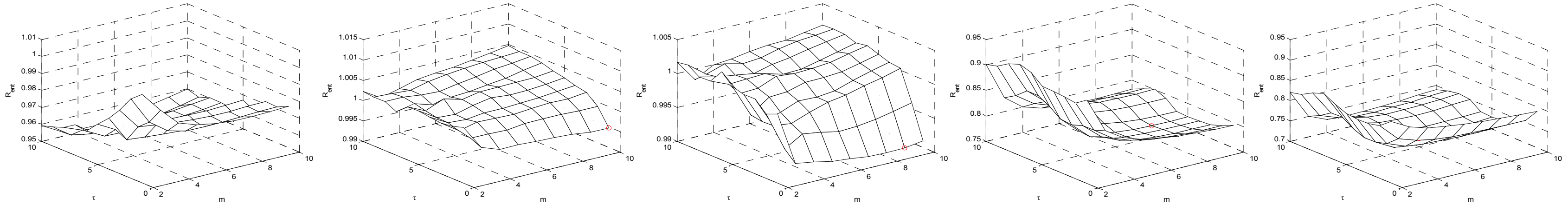

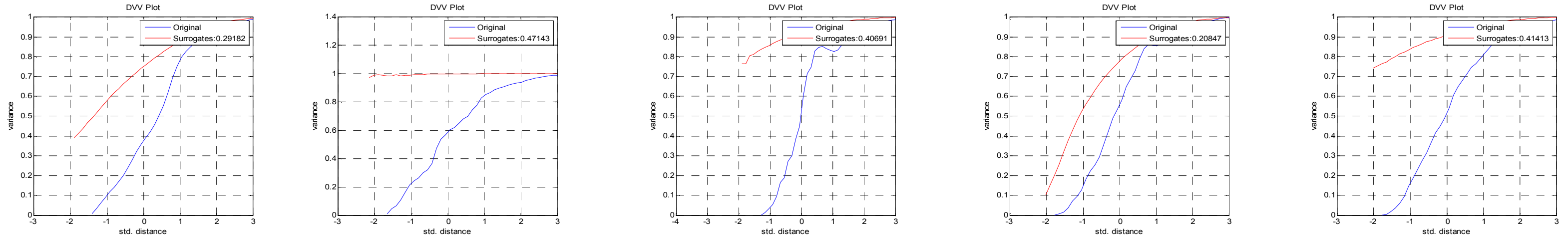

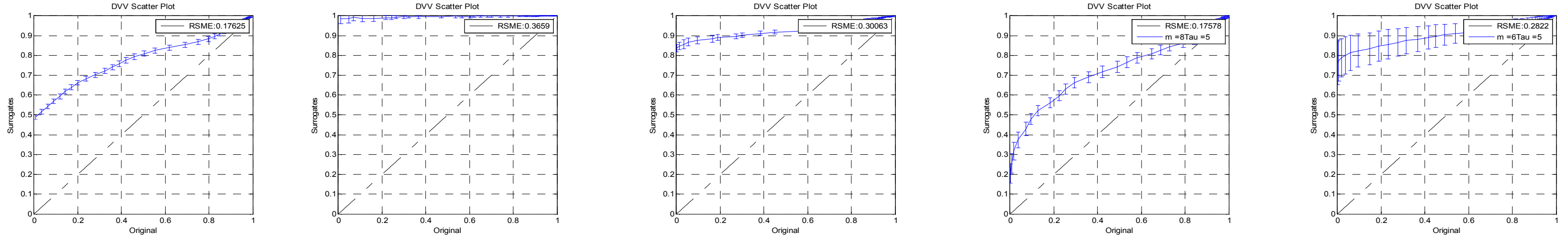

CH1 CH2 CH3 LDVg LDV1

CH1 CH2 CH3 LDVg LDV1

EXP 2 METHOD 2

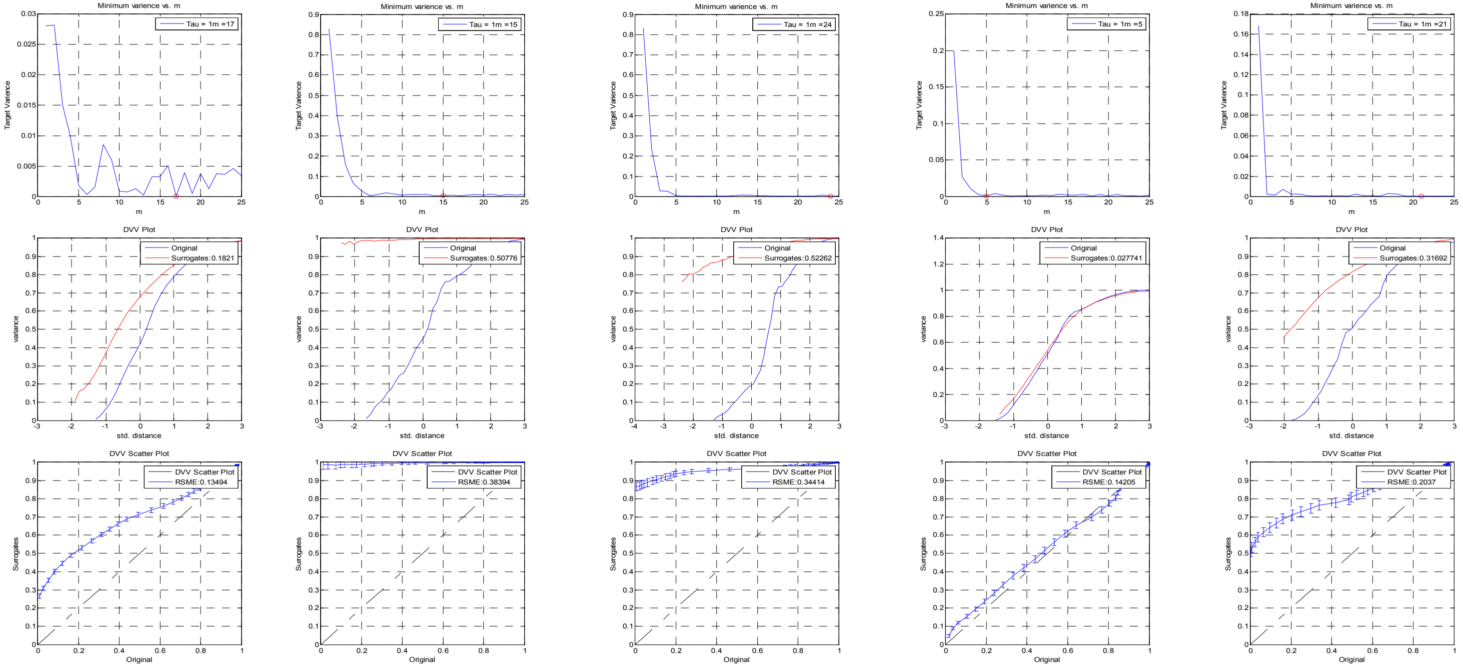

CH1 CH2 CH3 LDVg LDV1

METHOD3

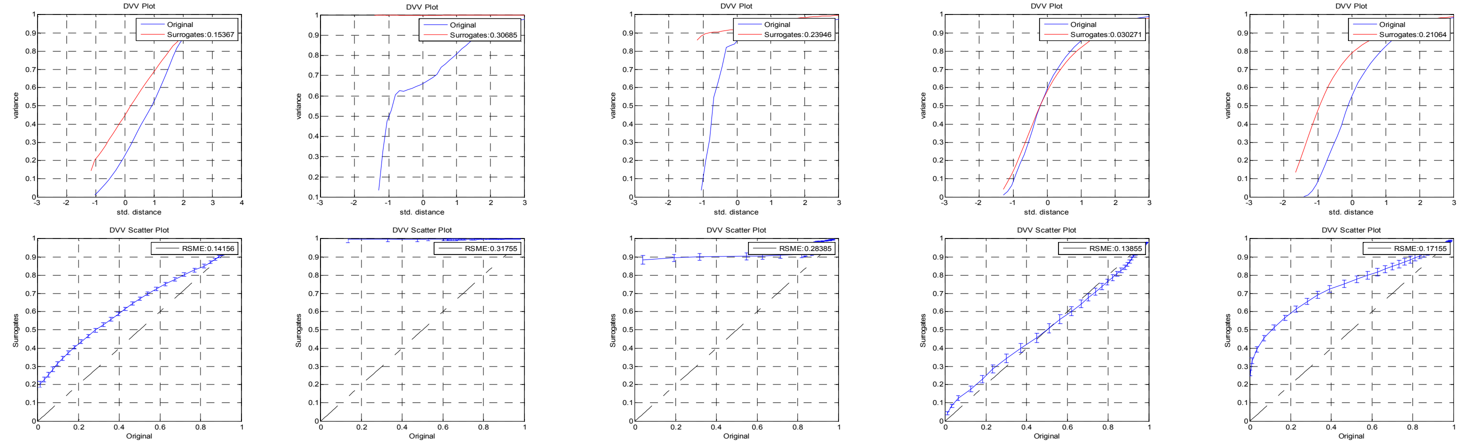

EXPERIMENT	SYSTEM CHARACTERISTCS	VARIABLES		METHOD 1				METHOD 2				METHOD 3			
				best **m**	best **τ**	rsme	RSME	calc **m**	**τ**	rsme	RSME	set **m**	set **τ**	rsme	RSME
3	SDOF CAR attached to fixed supports by 6 calibrated springs (3 on each side)	surface plastic (smooth) number of springs 2 x 3; loading harmonic 2-4-6-8-10 Hz	CH1	4	10	0.257	0.175	11	1	0.175	0.123	3	1	0.233	0.128
			CH2	10	1	0.421	0.299	14	1	0.473	0.326	3	1	0.233	0.273
			CH3	10	1	0.328	0.202	19	1	0.401	0.234	3	1	0.127	0.213
			LDVg	9	5	0.201	0.153	15	1	0.074	0.129	3	1	0.034	0.134
			LDV1	6	5	0.447	0.301	5	1	0.263	0.179	3	1	0.141	0.167

* results from EXP3_A-C

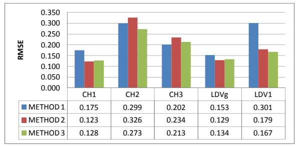

	CH1	CH2	CH3	LDVg	LDV1
METHOD 1	0.175	0.299	0.202	0.153	0.301
METHOD 2	0.123	0.326	0.234	0.129	0.179
METHOD 3	0.128	0.273	0.213	0.134	0.167

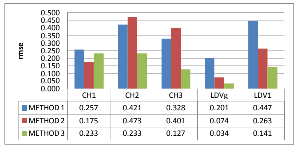

	CH1	CH2	CH3	LDVg	LDV1
METHOD 1	0.257	0.421	0.328	0.201	0.447
METHOD 2	0.175	0.473	0.401	0.074	0.263
METHOD 3	0.233	0.233	0.127	0.034	0.141

Data recorded 3D Accelerometer

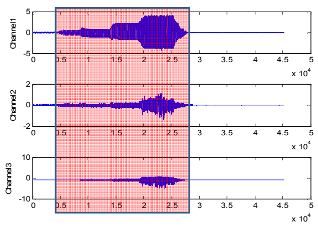

Data analysed 3D Accelerometer

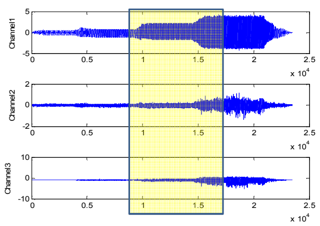

Data recorded LDV

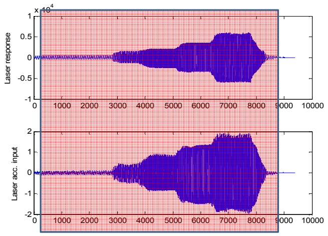

Data analysed LDV

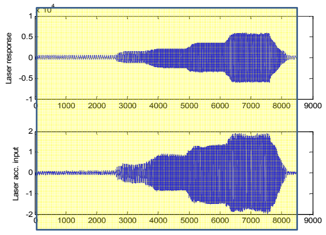

CH1 CH2 CH3 LDVg LDV1

METHOD 1

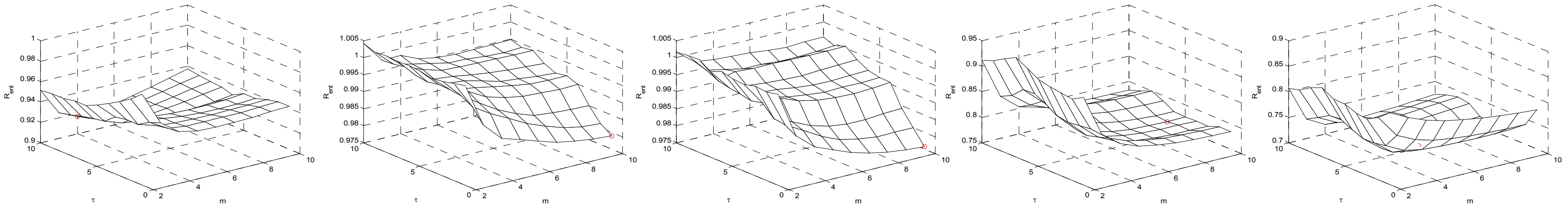

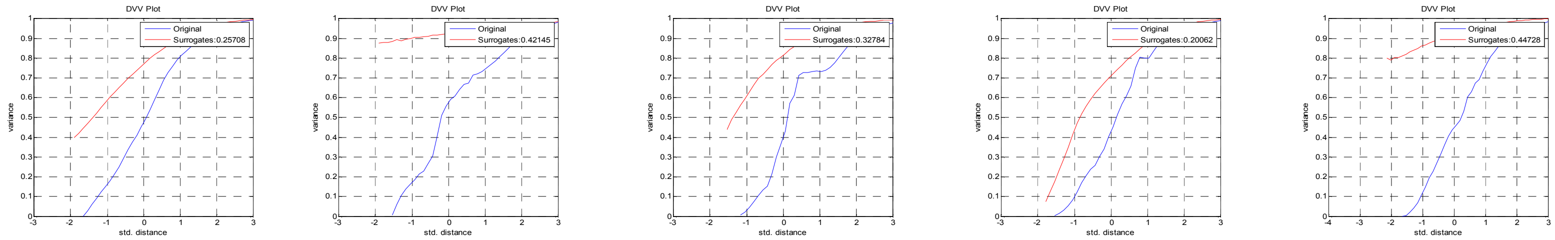

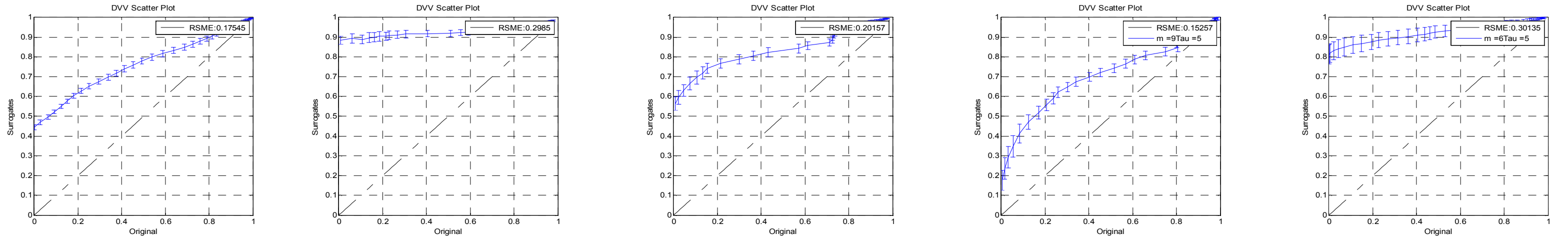

CH1 CH2 CH3 LDVg LDV1

CH1 CH2 CH3 LDVg LDV1

EXP 3 **METHOD 2**

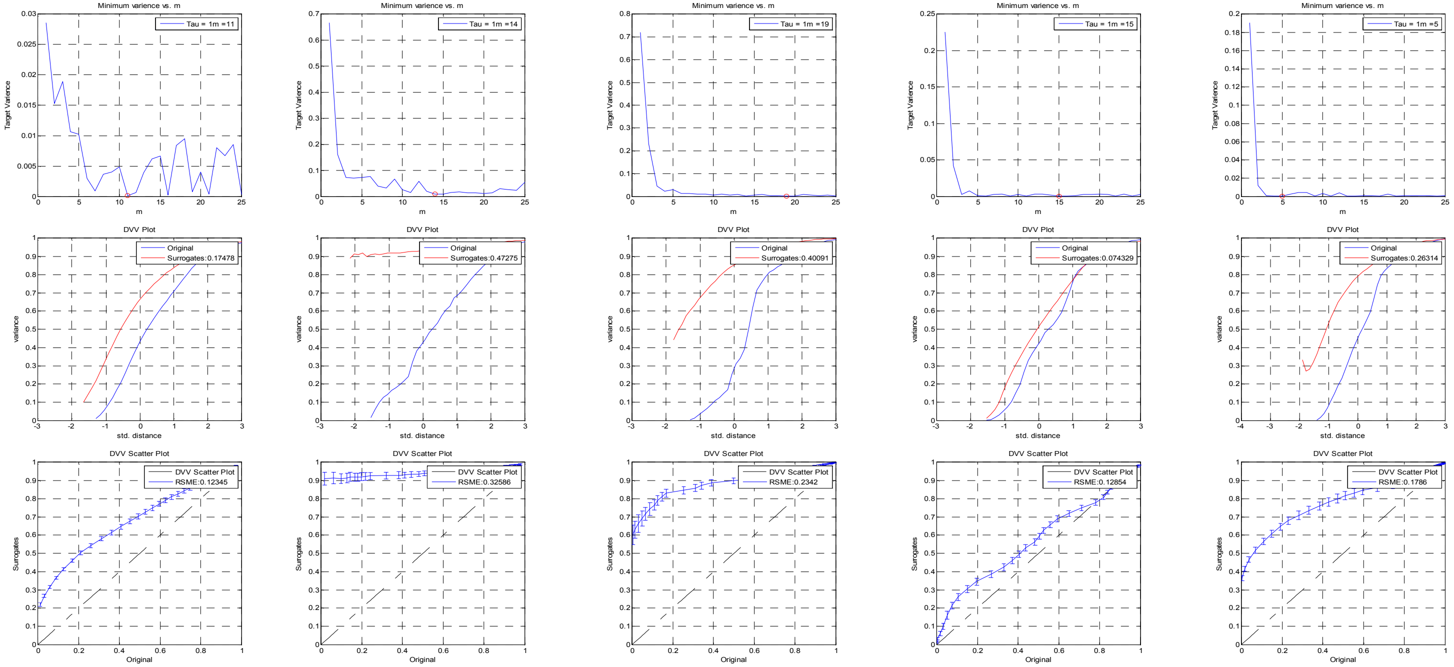

CH1 CH2 CH3 LDVg LDV1

METHOD 3

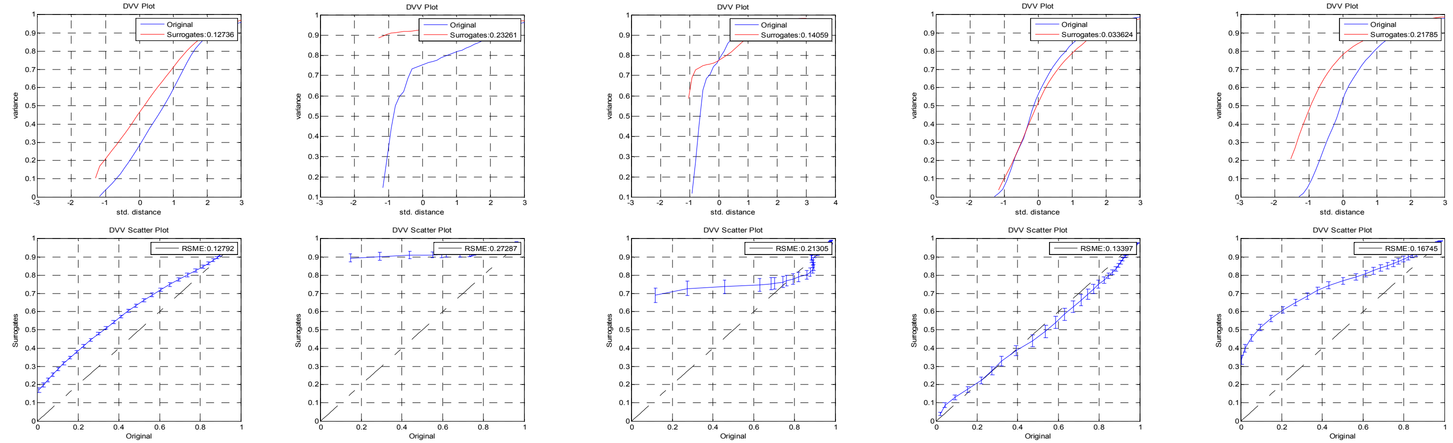

EXPERIMENT	SYSTEM CHARACTERISTCS	VARIABLES		METHOD 1				METHOD 2				METHOD 3			
				best **m**	best **τ**	rsme	RSME	calc **m**	**τ**	rsme	RSME	set **m**	set **τ**	rsme	RSME
4	SDOF CAR attached to fixed supports by 6 calibrated springs (3 on each side)	surface wood number of springs 2 x 3; loading Sine Sweep	CH1	4	10	0.325	0.188	25	1	0.211	0.133	3	1	0.117	0.133
			CH2	10	2	0.429	0.373	22	1	0.490	0.368	3	1	0.286	0.334
			CH3	9	1	0.466	0.298	13	1	0.494	0.306	3	1	0.242	0.288
			LDVg	10	3	0.376	0.296	17	1	0.084	0.114	3	1	0.032	0.127
			LDV1	9	4	0.367	0.291	11	1	0.248	0.176	3	1	0.172	0.165

* results from EXP4_A-E

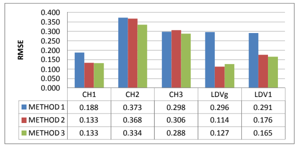

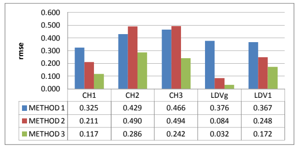

Data recorded 3D Accelerometer

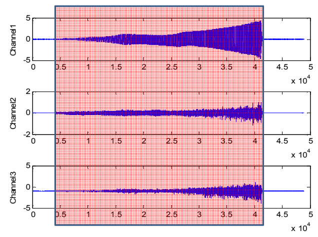

Data analysed 3D Accelerometer

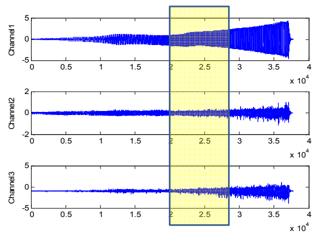

Data recorded LDV

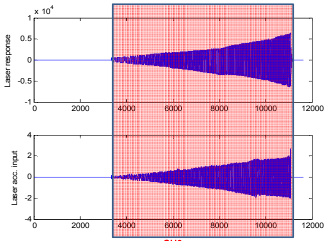

Data analysed LDV

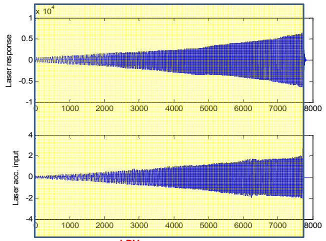

CH1 CH2 CH3 LDVg LDV1

METHOD 1

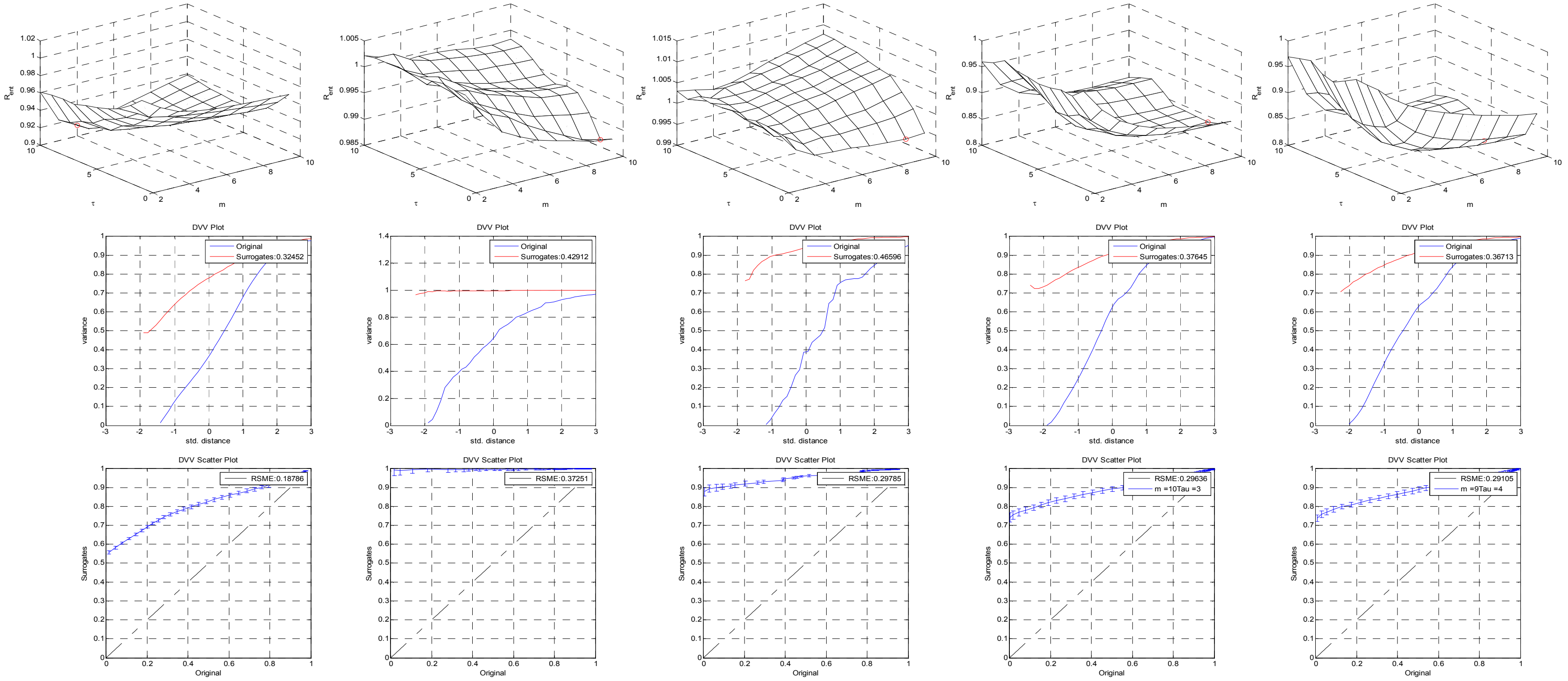

CH1 CH2 CH3 LDVg LDV1

CH1 CH2 CH3 LDVg LDV1

EXP 4 METHOD 2

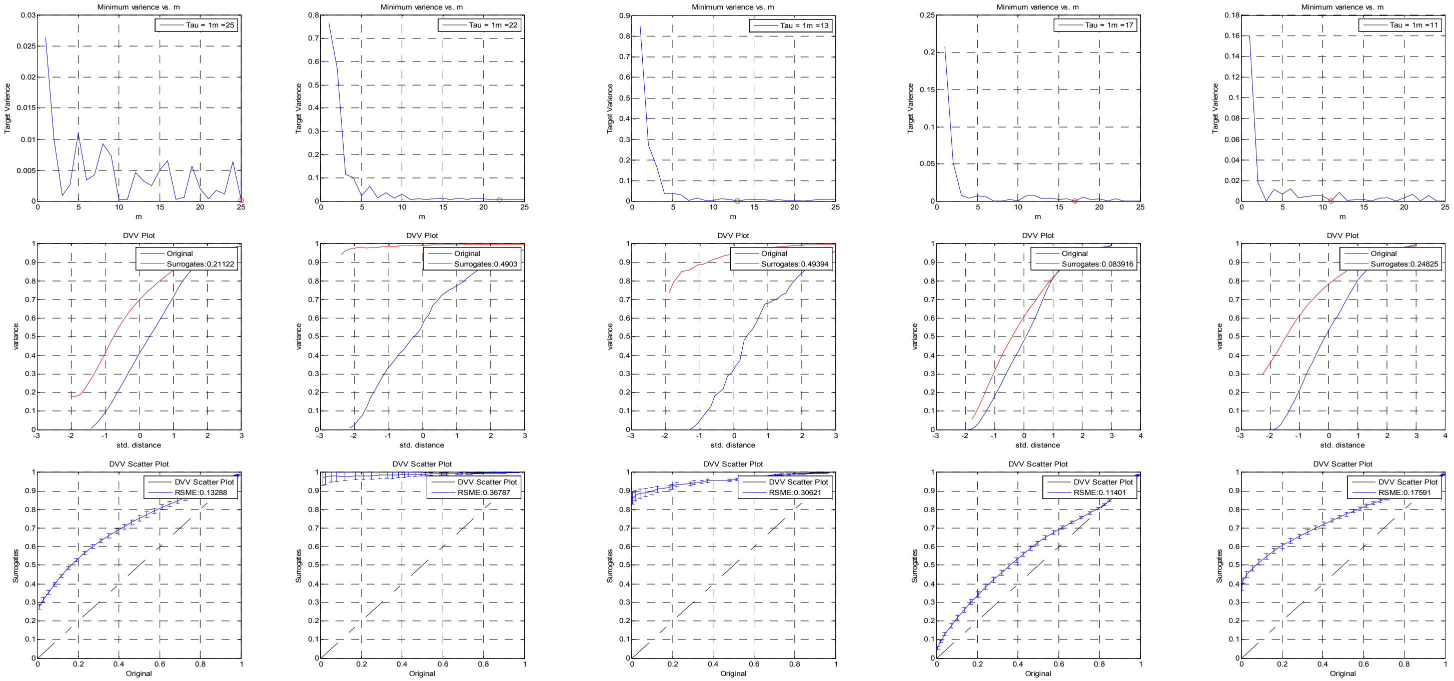

CH1 CH2 CH3 LDVg LDV1

METHOD 3

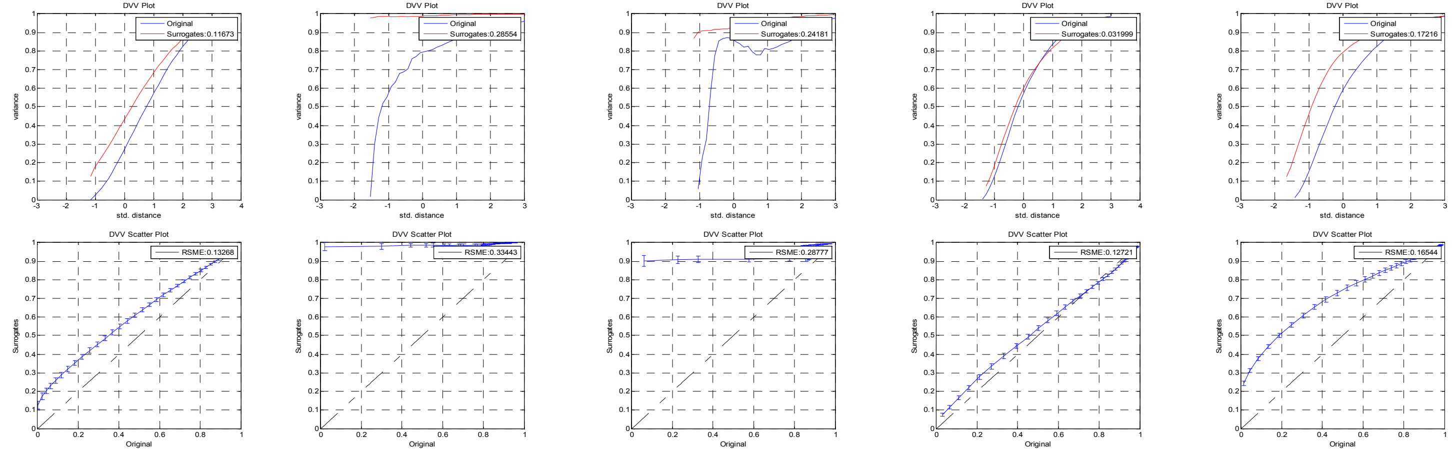

EXPERIMENT	SYSTEM CHARACTERISTCS	VARIABLES		METHOD 1				METHOD 2				METHOD 3			
				best **m**	best **τ**	rsme	RSME	calc **m**	**τ**	rsme	RSME	set **m**	set **τ**	rsme	RSME
5	SDOF CAR attached to fixed supports by 6 calibrated springs (3 on each side)	surface wood number of springs 2 x 3; loading White Noise	CH1	7	1	0.145	0.113	9	1	0.139	0.129	3	1	0.122	0.097
			CH2	6	1	0.256	0.394	14	1	0.376	0.394	3	1	0.144	0.379
			CH3	7	1	0.348	0.357	10	1	0.393	0.368	3	1	0.185	0.357
			LDVg	6	1	0.070	0.293	10	1	0.198	0.418	3	1	0.232	0.347
			LDV1	3	1	0.005	0.155	3	1	0.179	0.202	3	1	0.180	0.204

*** results from EXP5_A**

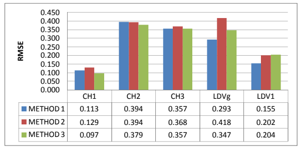

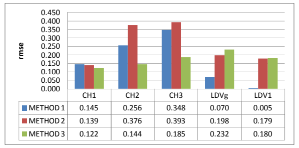

Data recorded 3D Accelerometer

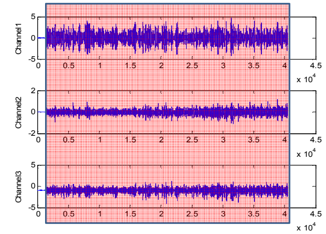

Data analysed 3D Accelerometer

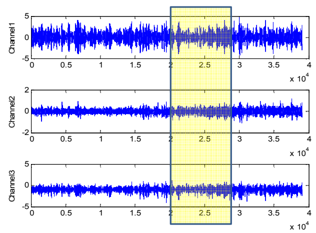

Data recorded LDV

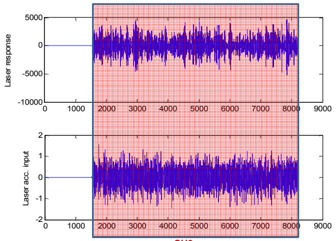

Data analysed LDV

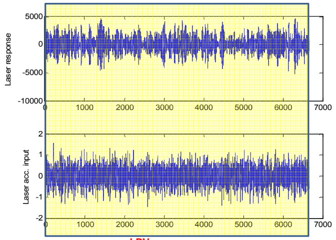

CH1 **CH2** **CH3** **LDVg** **LDV1**

METHOD 1

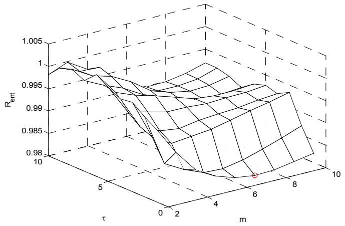
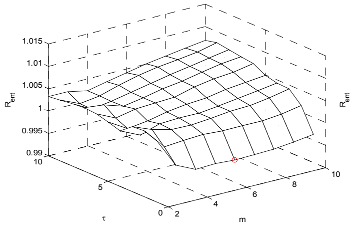
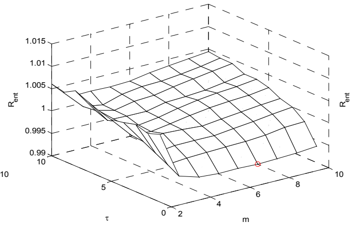
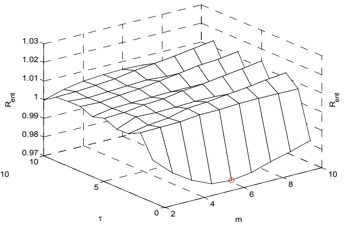
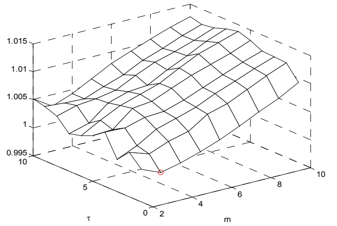

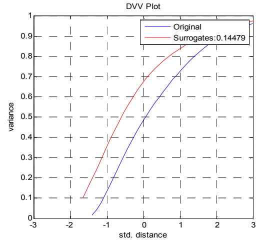

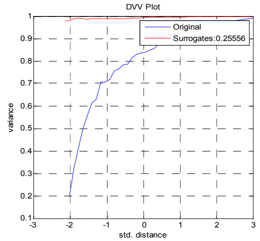

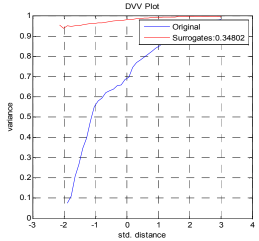

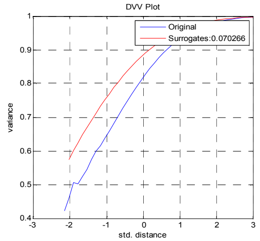

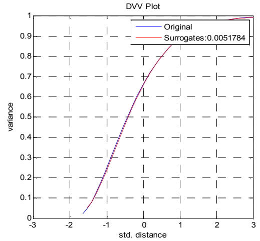

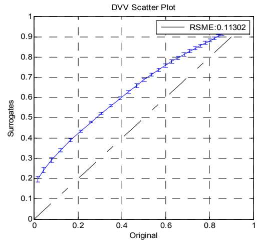

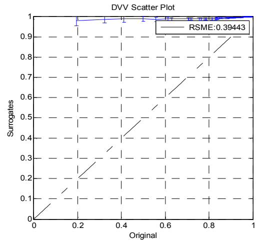

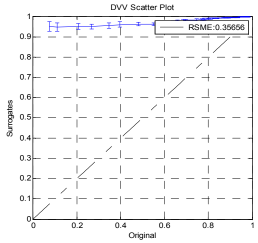

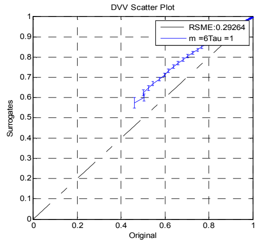

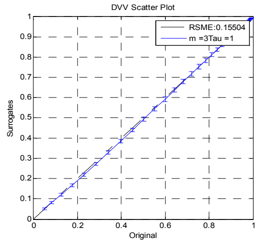

CH1 **CH2** **CH3** **LDVg** **LDV1**

	CH1		CH2	CH3	LDVg	LDV1
EXP 5		METHOD 2				

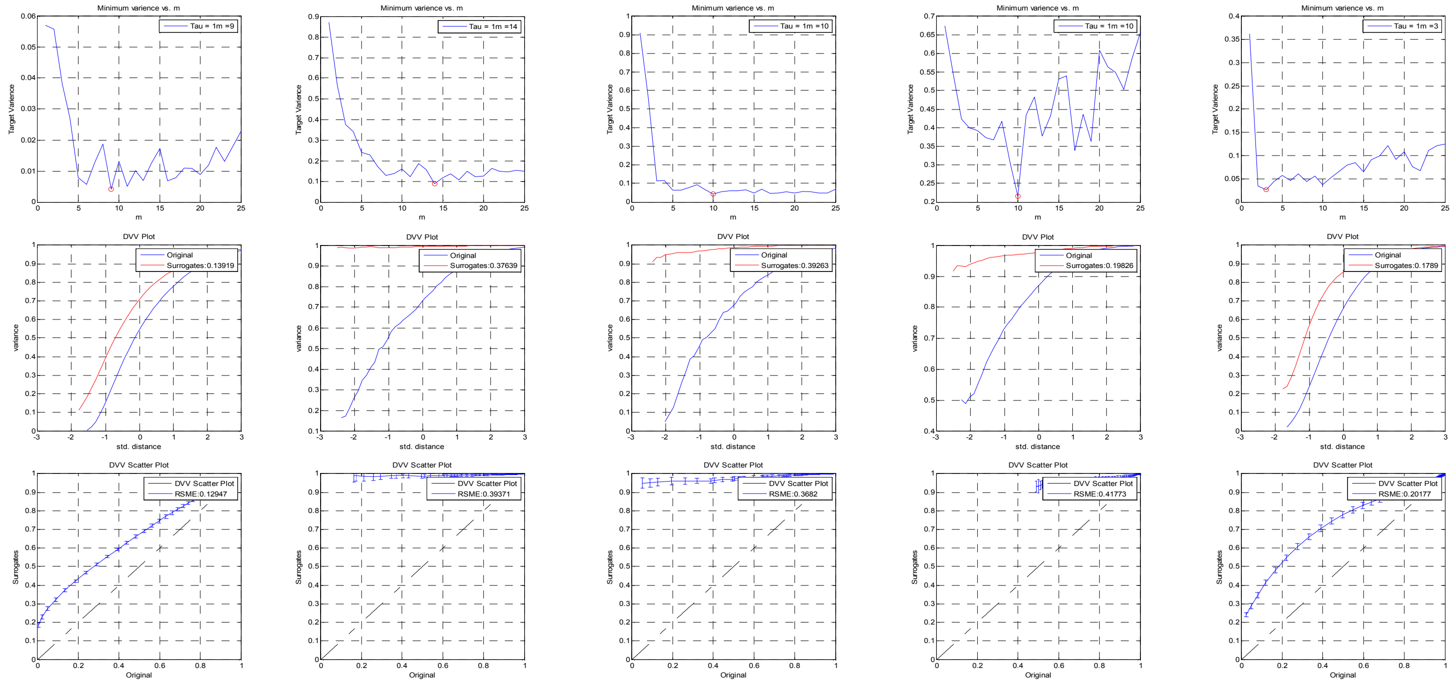

CH1	METHOD 3	CH2	CH3	LDVg	LDV1

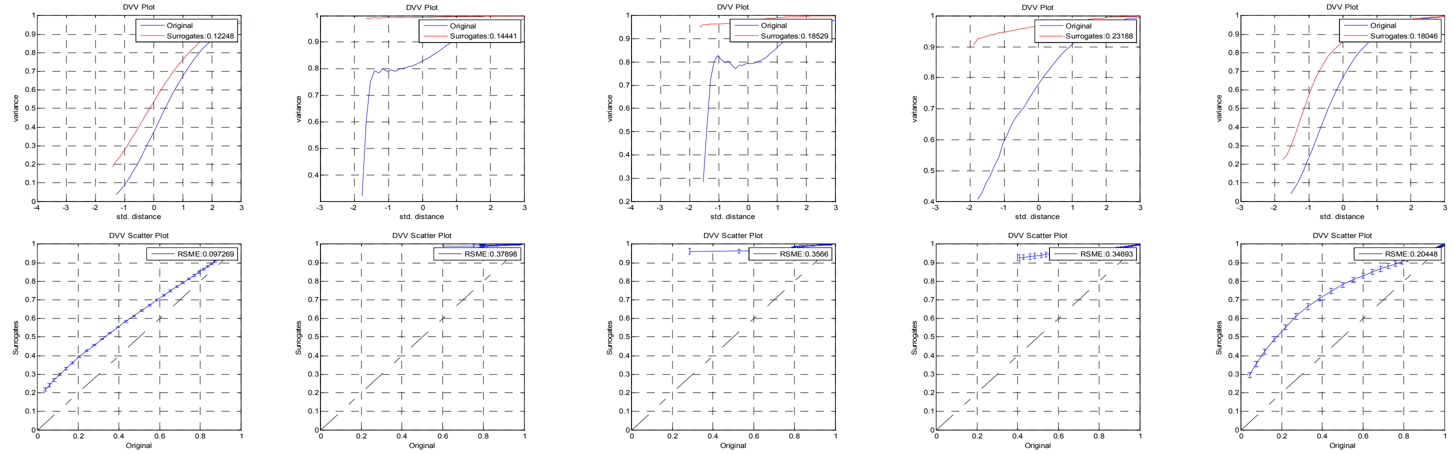

EXPERIMENT	SYSTEM CHARACTERISTCS	VARIABLES		METHOD 1				METHOD 2				METHOD 3			
				best **m**	best τ	rsme	RSME	calc **m**	τ	rsme	RSME	set **m**	set τ	rsme	RSME
6	SDOF CAR attached to fixed supports by 6 calibrated springs (3 on each side)	surface plastic (smooth) number of springs 2 x 3; loading Sine Sweep	CH1	4	10	0.296	0.169	23	1	0.176	0.129	3	1	0.117	0.145
			CH2	10	2	0.399	0.315	16	1	0.398	0.288	3	1	0.252	0.265
			CH3	10	1	0.302	0.160	14	1	0.299	0.166	3	1	0.161	0.161
			LDVg	10	6	0.241	0.224	6	1	0.042	0.136	3	1	0.029	0.130
			LDV1	8	10	0.424	0.360	6	1	0.240	0.173	3	1	0.172	0.165

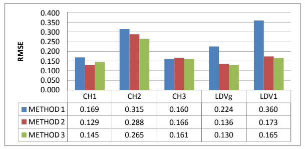

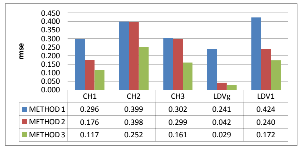

Data recorded 3D Accelerometer

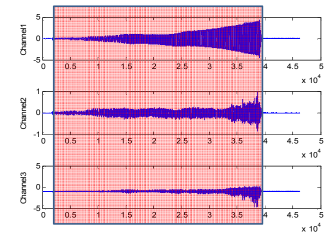

Data analysed 3D Accelerometer

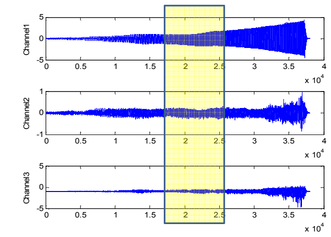

Data recorded LDV

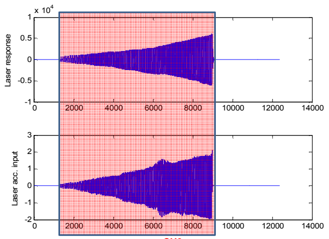

Data analysed LDV

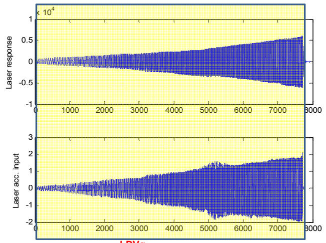

CH1 CH2 CH3 LDVg LDV1

METHOD 1

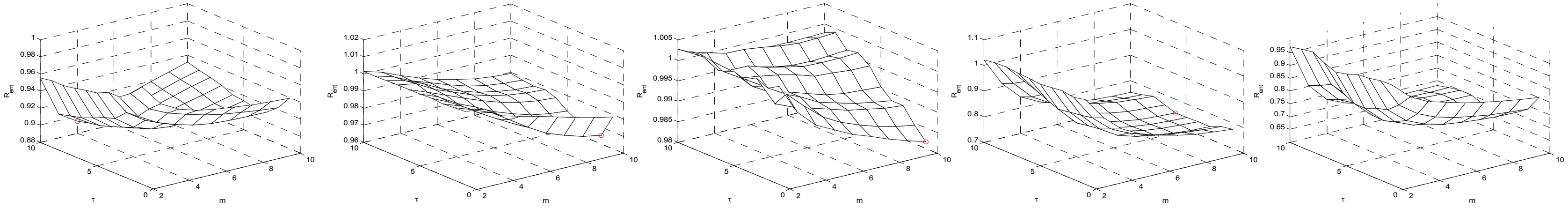

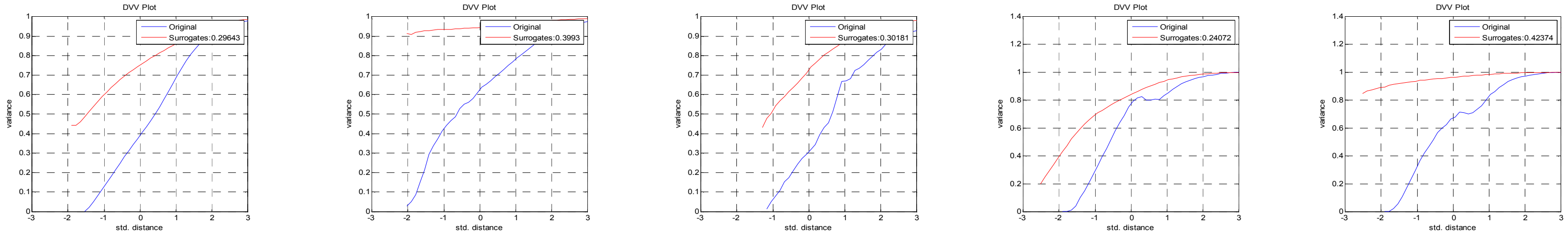

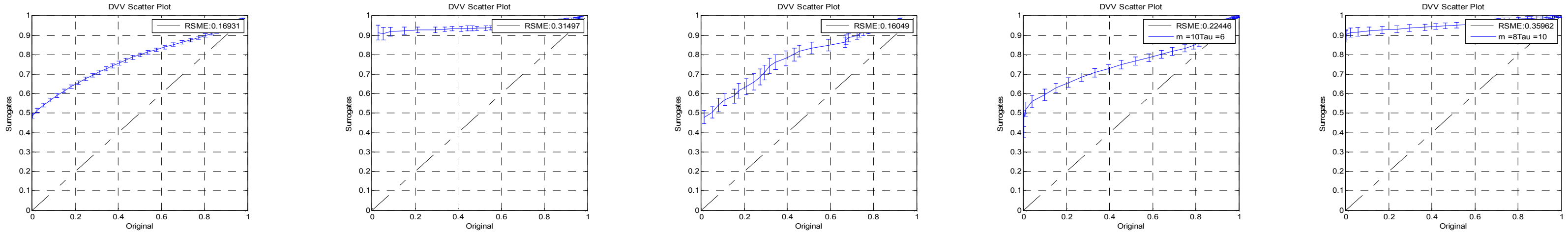

CH1 CH2 CH3 LDVg LDV1

CH1	CH2	CH3	LDVg	LDV1

EXP 6 **METHOD 2**

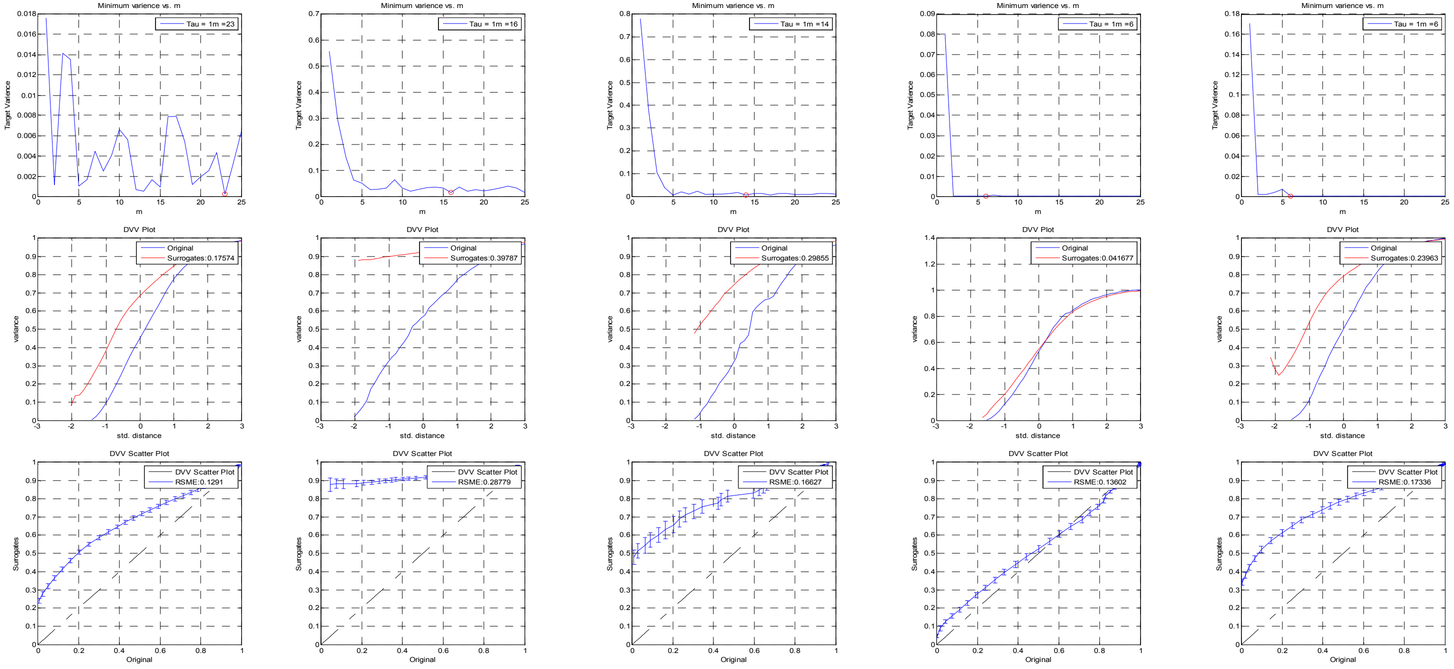

METHOD 3

CH1	CH2	CH3	LDVg	LDV1

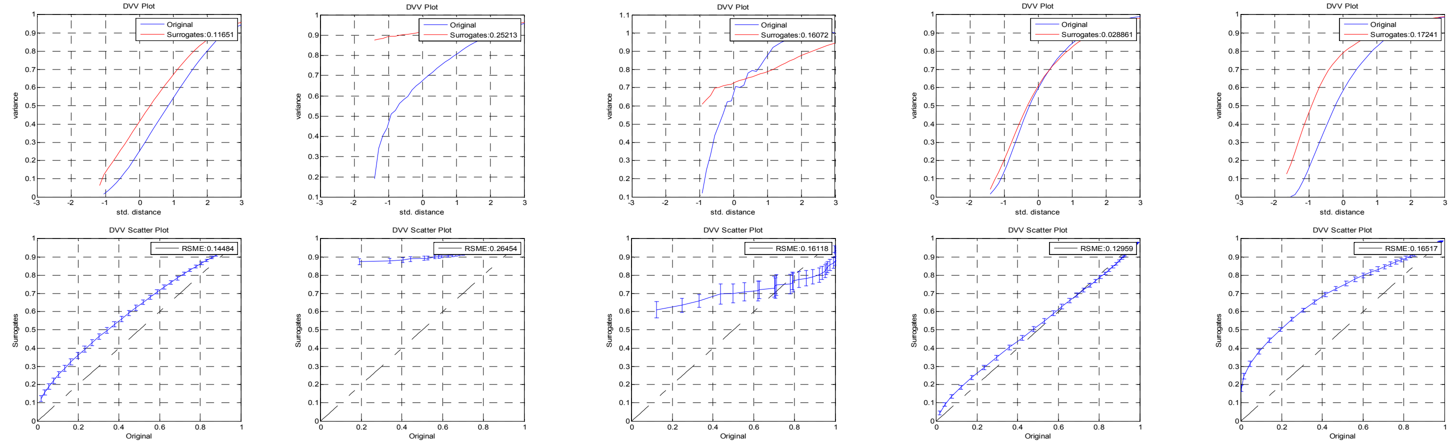

EXPERIMENT	SYSTEM CHARACTERISTCS	VARIABLES		METHOD 1				METHOD 2				METHOD 3			
				best **m**	best **τ**	rsme	RSME	calc **m**	**τ**	rsme	RSME	set **m**	set **τ**	rsme	RSME
7	SDOF CAR attached to fixed supports by 6 calibrated springs (3 on each side)	surface plastic (smooth) number of springs 2 x 3; loading White Noise	CH1	6	1	0.142	0.110	13	1	0.123	0.148	3	1	0.115	0.101
			CH2	6	1	0.300	0.360	14	1	0.348	0.395	3	1	0.185	0.348
			CH3	7	1	0.214	0.194	14	1	0.266	0.208	3	1	0.084	0.212
			LDVg	6	1	0.251	0.367	4	1	0.252	0.353	3	1	0.243	0.340
			LDV1	2	3	0.256	0.263	3	1	0.185	0.201	3	1	0.194	0.201

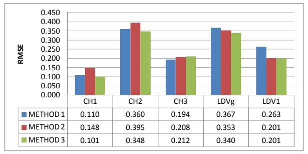

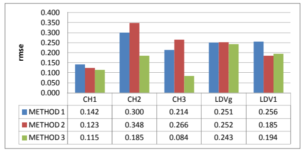

Data recorded 3D Accelerometer **Data analysed 3D Accelerometer** **Data recorded LDV** **Data analysed LDV**

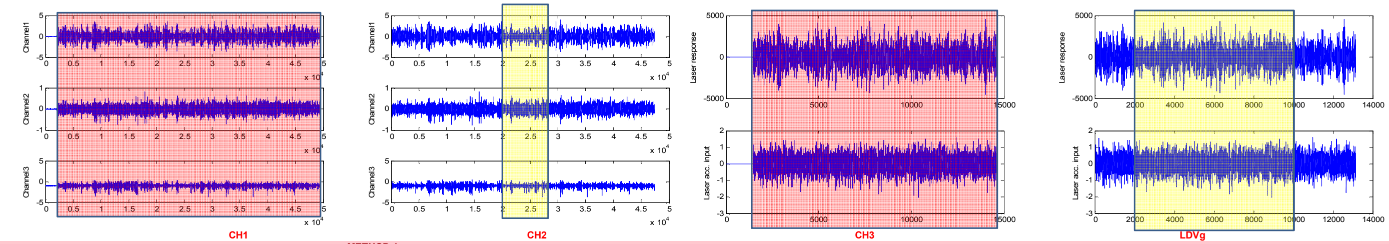

CH1 CH2 CH3 LDVg LDV1

METHOD 1

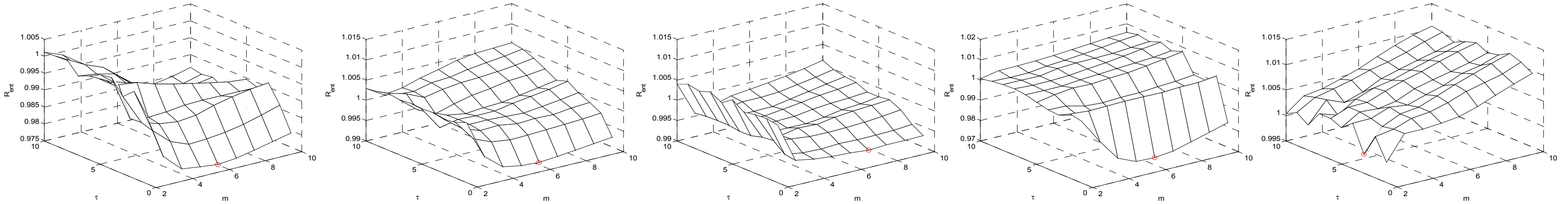

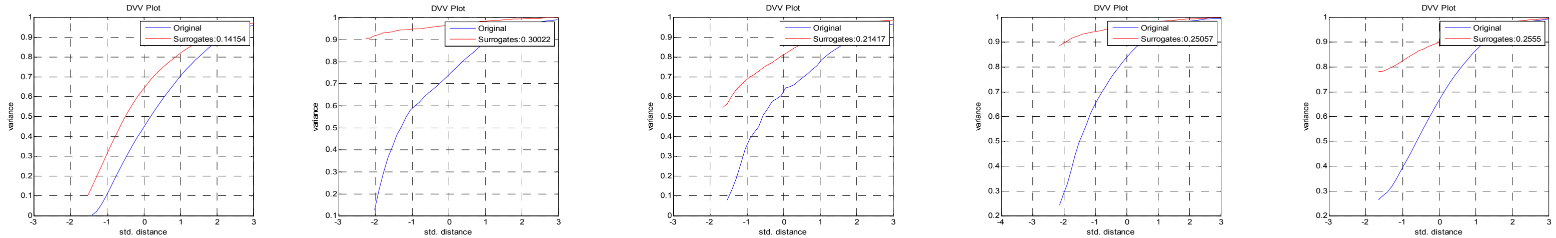

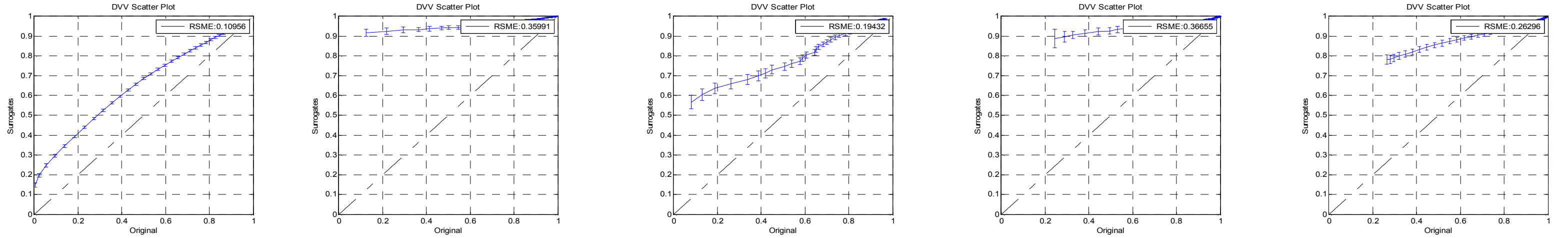

CH1 CH2 CH3 LDVg LDV1

CH1 CH2 CH3 LDVg LDV1

EXP 7 **METHOD 2**

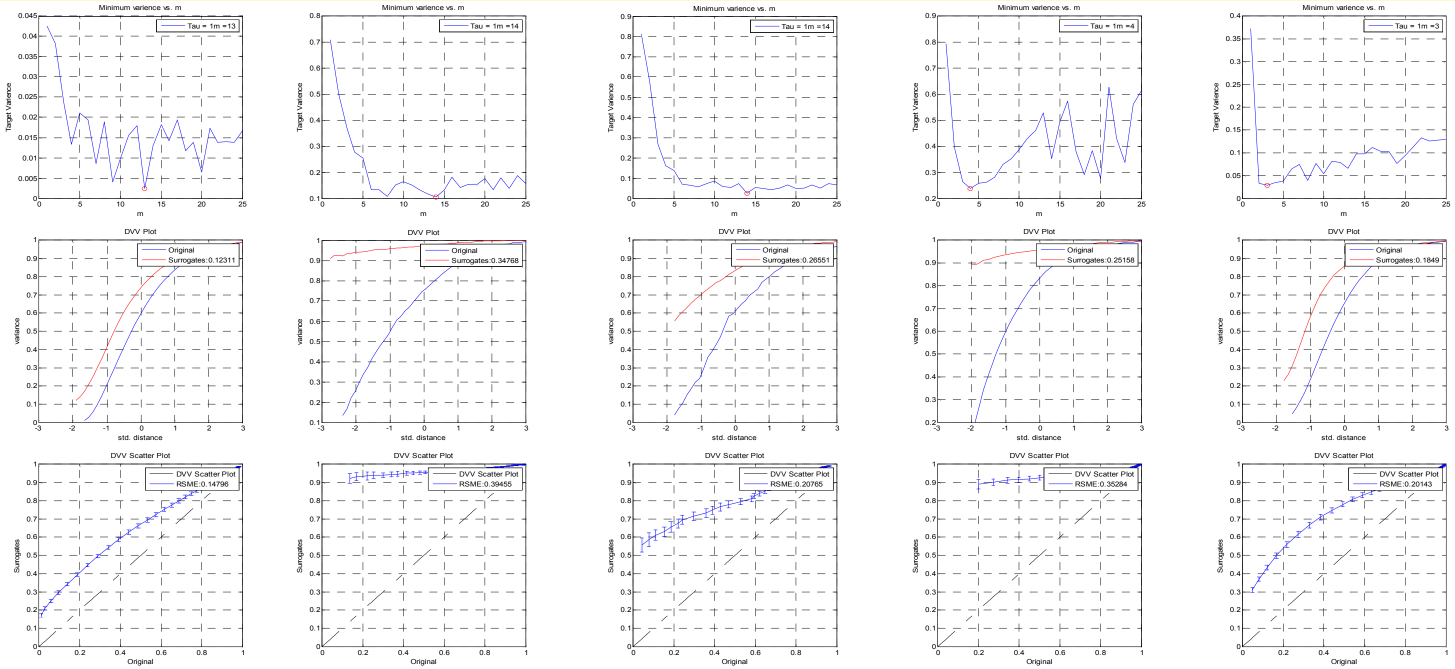

CH1 CH2 CH3 LDVg LDV1

METHOD 3

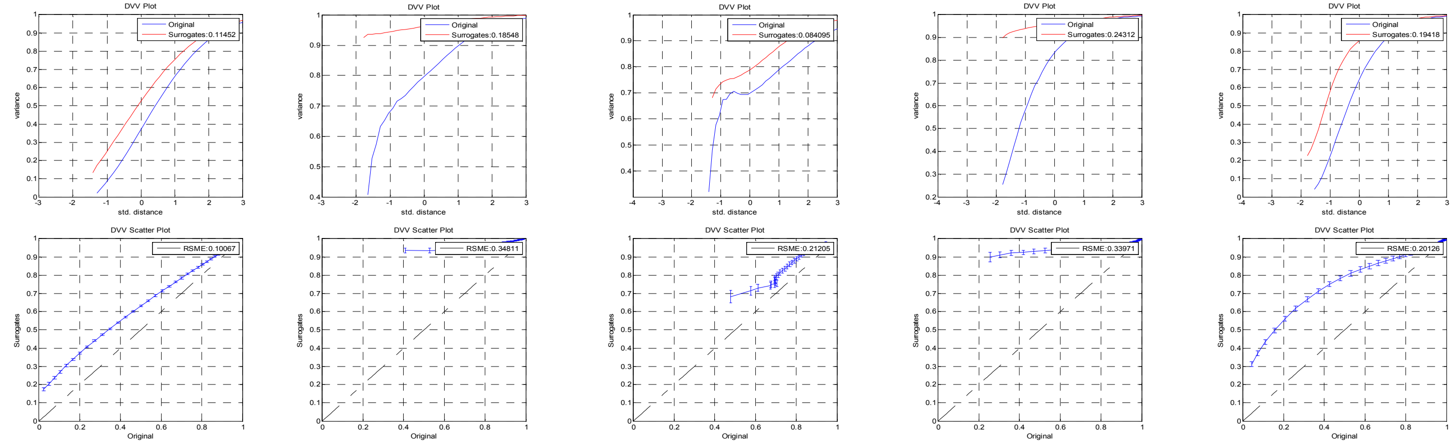

EXPERIMENT	SYSTEM CHARACTERISTCS	VARIABLES		METHOD 1				METHOD 2				METHOD 3			
				best **m**	best τ	rsme	RSME	calc **m**	τ	rsme	RSME	set **m**	set τ	rsme	RSME
8	SDOF CAR attached to fixed supports by 6 calibrated springs (3 on each side)	surface sand paper (rough) number of springs 2 x 3; loading White Noise	CH1	6	1	0.1395	0.1110	5	1	0.1363	0.1030	3	1	0.1236	0.0964
			CH2	3	8	0.0632	0.4353	12	1	0.3188	0.4012	3	1	0.1377	0.3934
			CH3	4	4	0.0432	0.3464	7	1	0.1167	0.2232	3	1	0.0657	0.2730
			LDVg	6	1	0.1901	0.4007	9	1	0.1931	0.4276	3	1	0.1886	0.3663
			LDV1	5	1	0.1922	0.1463	17	1	0.2249	0.2143	3	1	0.1933	0.2026

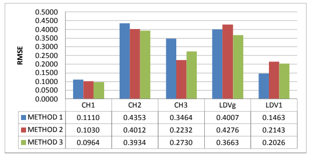

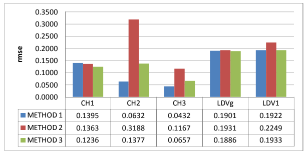

Data recorded 3D Accelerometer

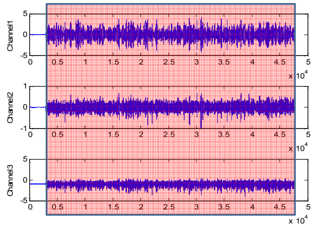

Data analysed 3D Accelerometer

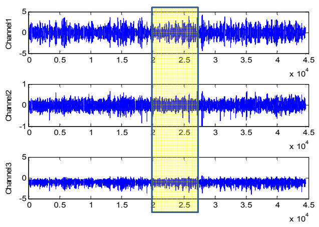

Data recorded LDV

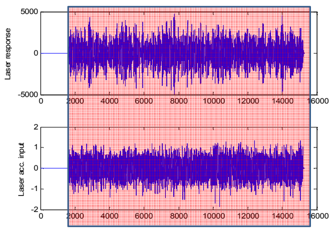

Data analysed LDV

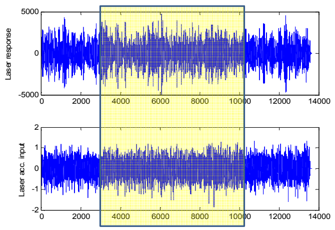

CH1 **CH2** **CH3** **LDVg** **LDV1**

METHOD 1

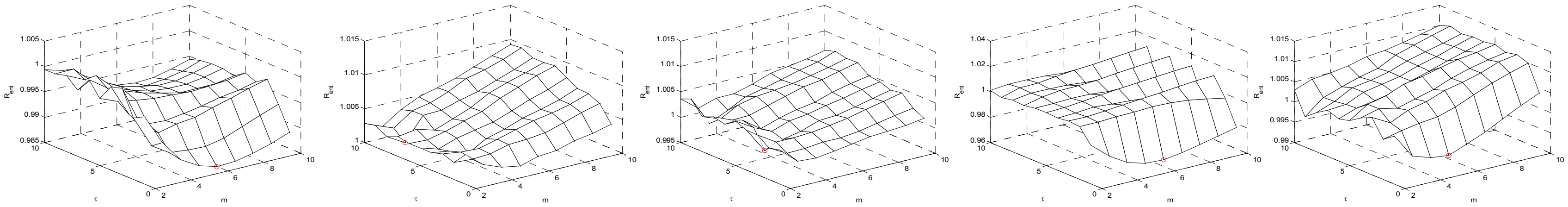

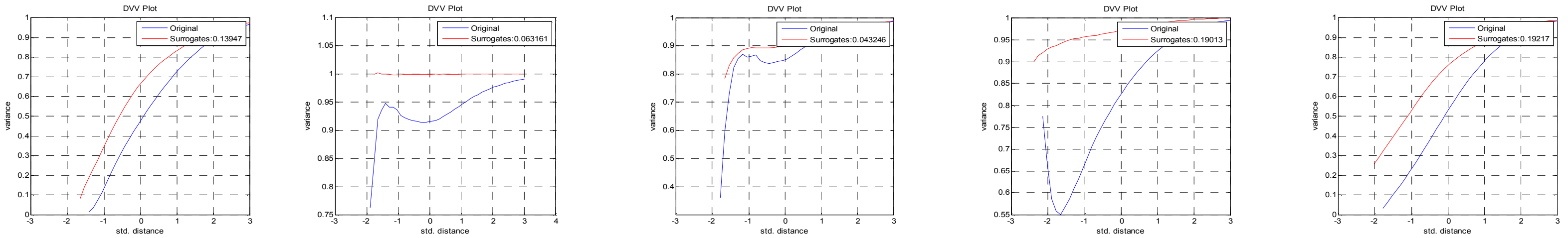

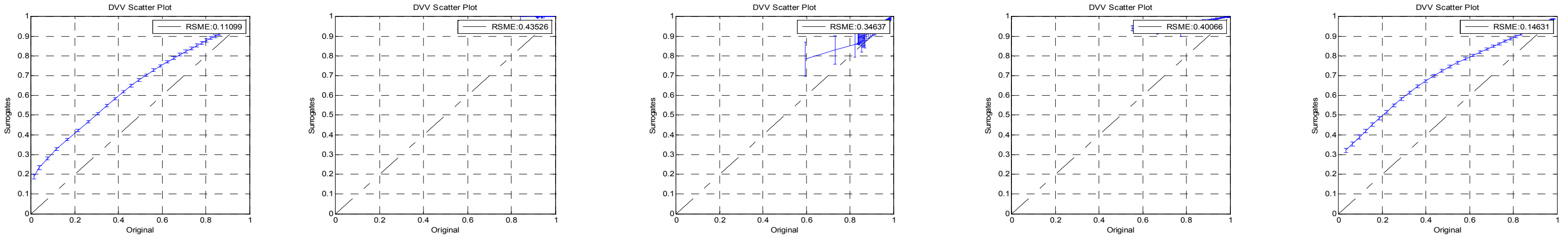

CH1 **CH2** **CH3** **LDVg** **LDV1**

CH1 CH2 CH3 LDVg LDV1

EXP 8 METHOD 2

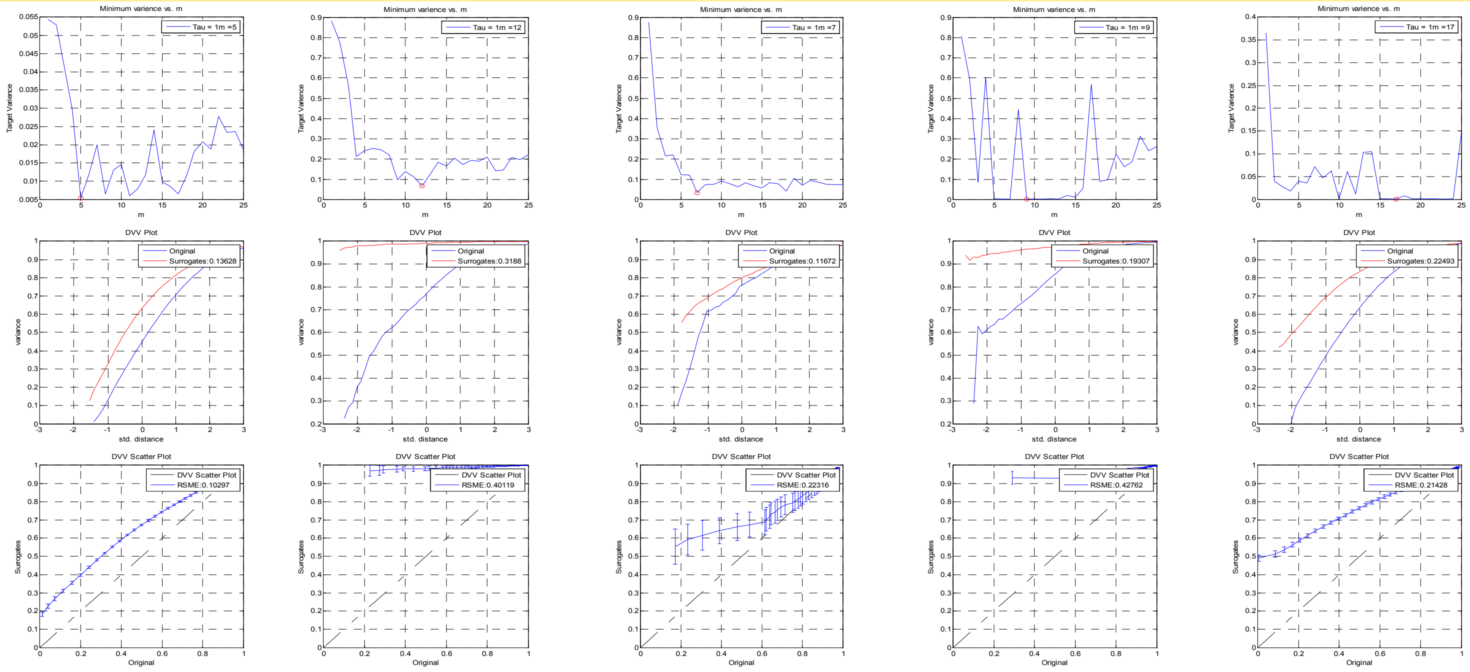

METHOD 3

CH1 CH2 CH3 LDVg LDV1

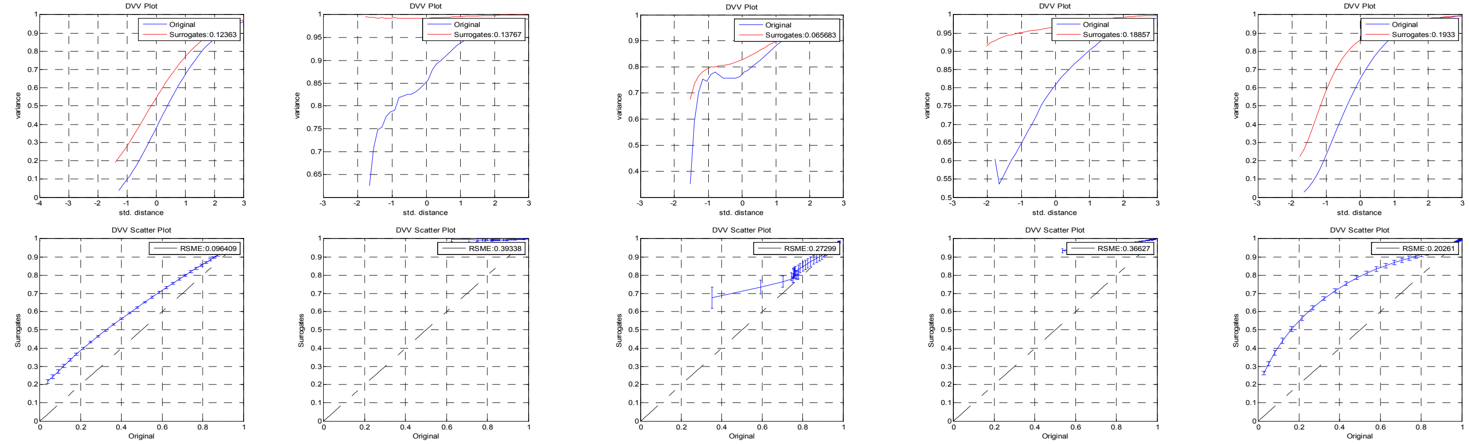

EXPERIMENT	SYSTEM CHARACTERISTCS	VARIABLES		METHOD 1				METHOD 2				METHOD 3			
				best **m**	best **τ**	rsme	RSME	calc **m**	**τ**	rsme	RSME	set **m**	set **τ**	rsme	RSME
9	SDOF CAR attached to fixed supports by 6 calibrated springs (3 on each side)	surface sand paper (rough) number of springs 2 x 3; loading Sine Sweep	CH1	5	9	0.2844	0.1682	4	1	0.1280	0.1360	3	1	0.1015	0.1308
			CH2	10	1	0.4205	0.3643	7	1	0.3815	0.3498	3	1	0.2444	0.3234
			CH3	10	1	0.3147	0.1674	18	1	0.3119	0.1823	3	1	0.1763	0.1581
			LDVg	10	3	0.1551	0.1549	15	1	0.0677	0.1084	3	1	0.0391	0.1260
			LDV1	9	4	0.3675	0.2888	22	1	0.2944	0.2135	3	1	0.1745	0.1623

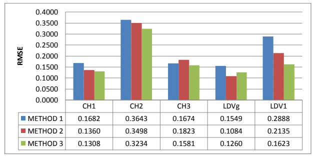

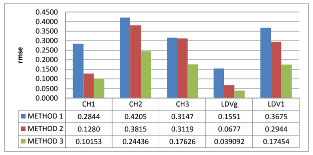

Data recorded 3D Accelerometer

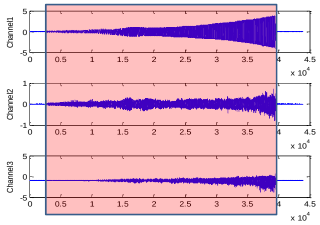

Data analysed 3D Accelerometer

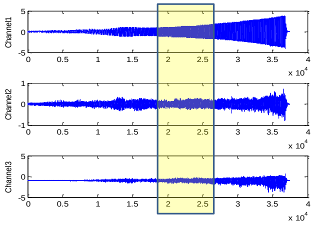

Data recorded LDV

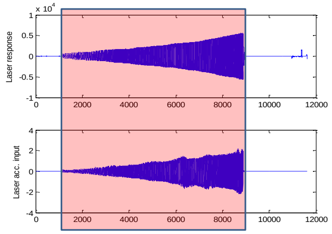

Data analysed LDV

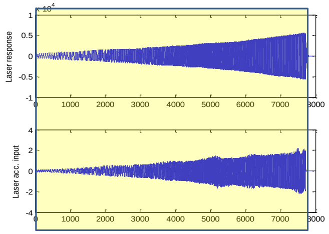

CH1 CH2 CH3 LDVg LDV1

METHOD 1

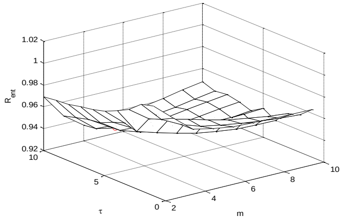
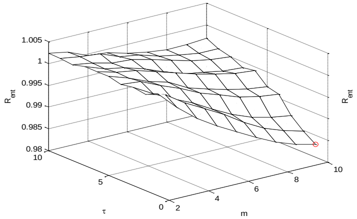
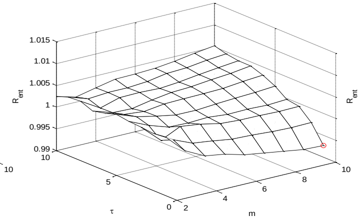
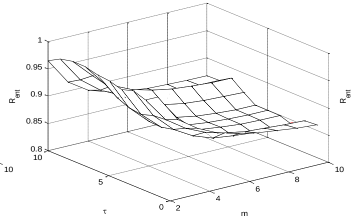
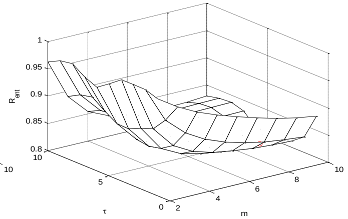

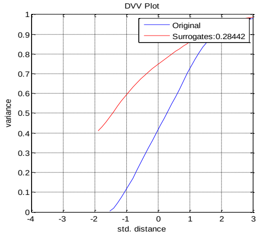

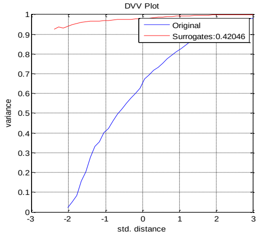

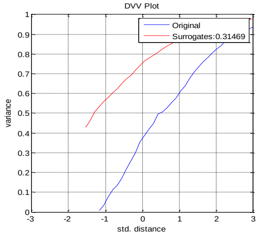

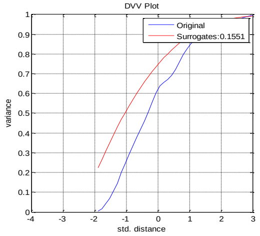

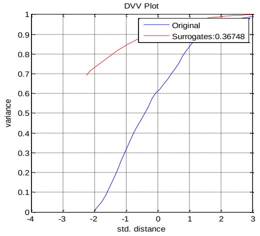

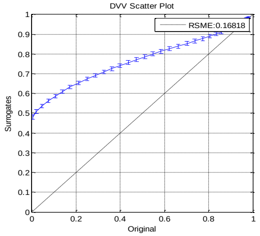

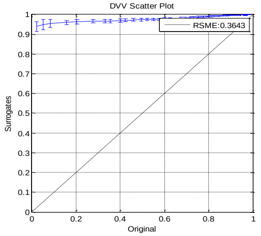

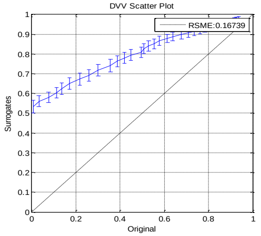

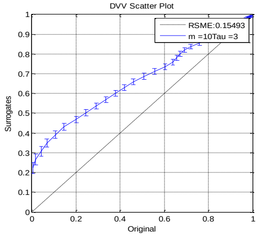

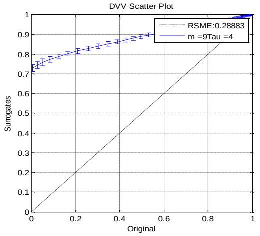

CH1 CH2 CH3 LDVg LDV1

EXP 9 METHOD 2

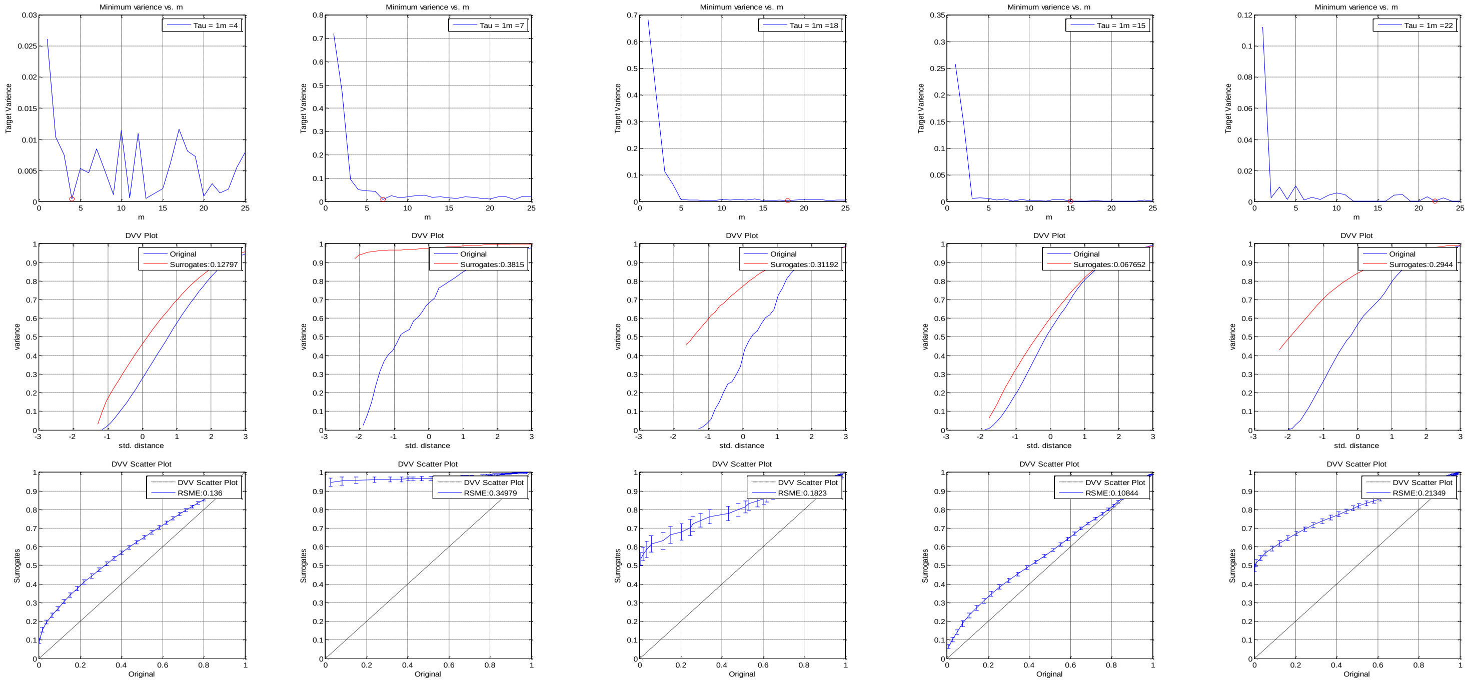

CH1 CH2 CH3 LDVg LDV1

METHOD 3

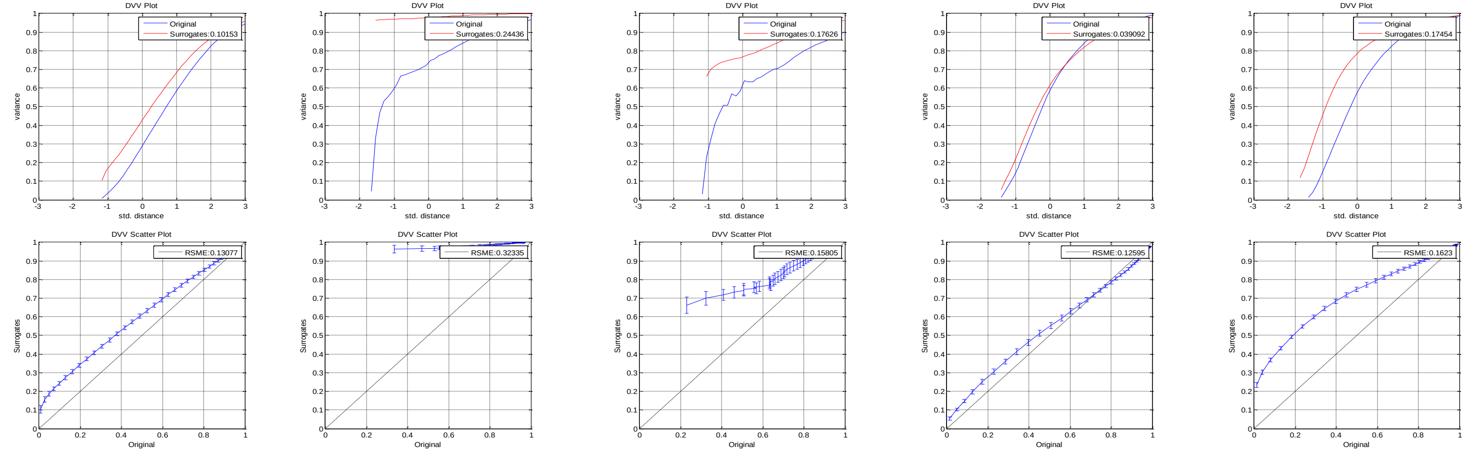

EXPERIMENT	SYSTEM CHARACTERISTCS	VARIABLES		METHOD 1				METHOD 2				METHOD 3			
				best m	best τ	rsme	RSME	calc m	τ	rsme	RSME	set m	set τ	rsme	RSME
10	SDOF CAR attached to fixed supports by 6 calibrated springs (3 on each side)	surface sand paper (rough) number of springs 2 x 3; loading harmonic 2-4-6-8-10 Hz	CH1	3	9	0.2816	0.1670	14	1	0.1980	0.1349	3	1	0.1458	0.1328
			CH2	8	1	0.3732	0.3401	11	1	0.4204	0.3552	3	1	0.2067	0.3402
			CH3	8	1	0.3422	0.1905	12	1	0.3237	0.2037	3	1	0.1902	0.2038
			LDVg	8	5	0.2355	0.1912	11	1	0.0542	0.1181	3	1	0.0303	0.1316
			LDV1	6	5	0.2460	0.1848	12	1	0.2924	0.1828	3	1	0.2012	0.1681

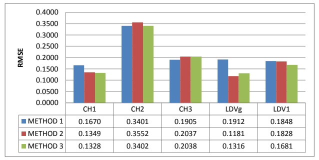

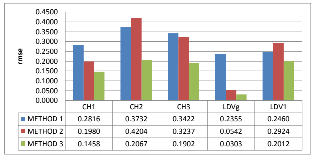

Data recorded 3D Accelerometer

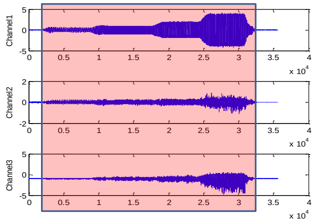

Data analysed 3D Accelerometer

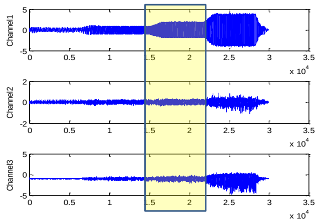

Data recorded LDV

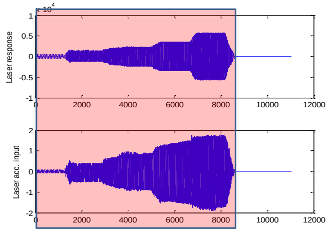

Data analysed LDV

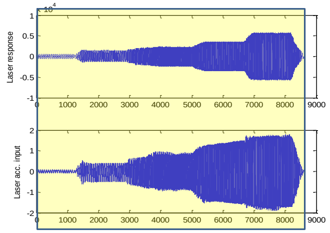

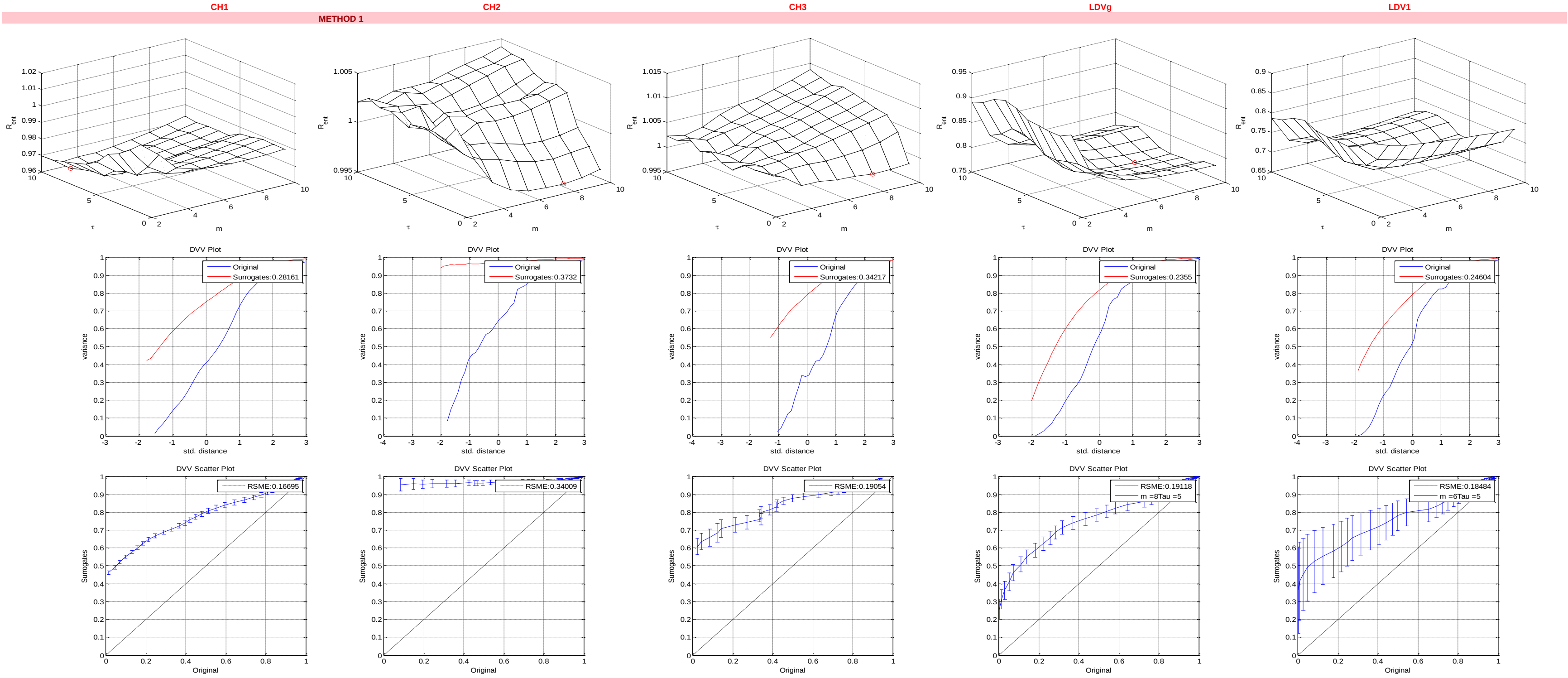

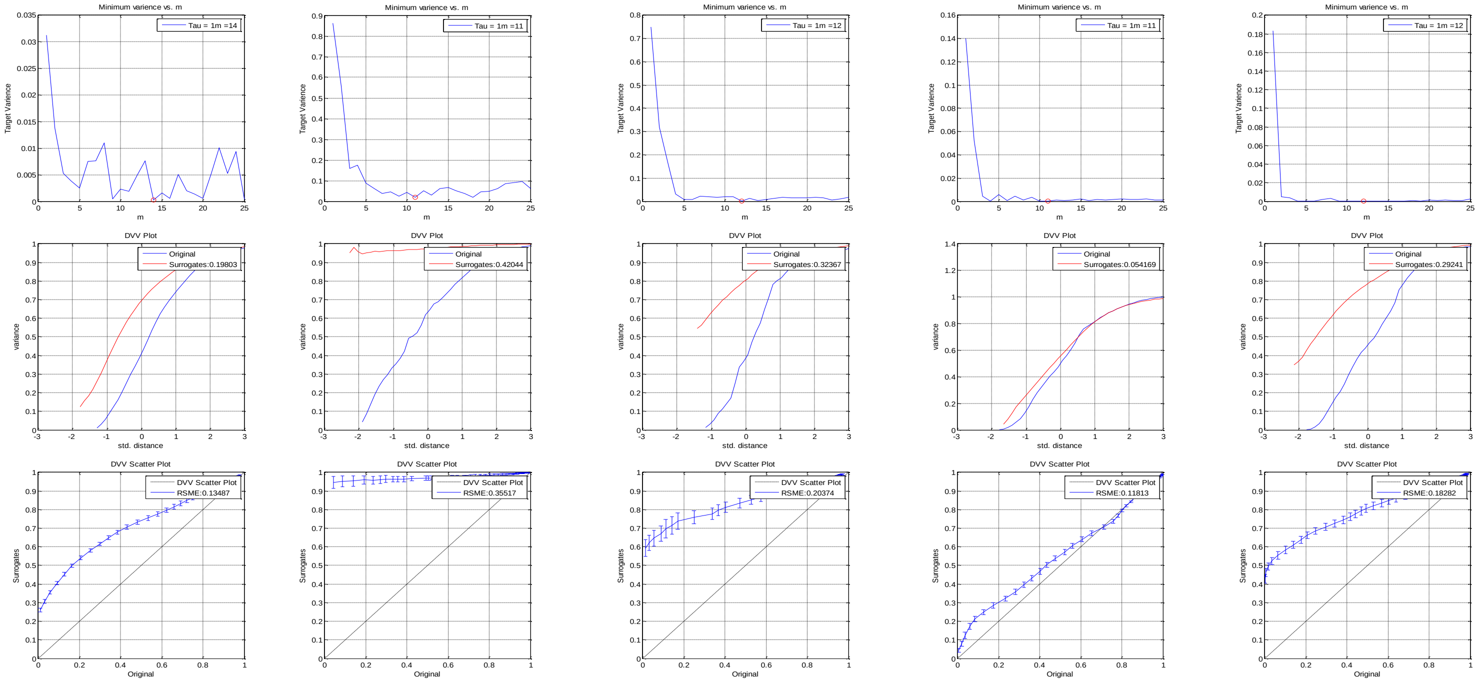
Minimum varience vs. m
Tau = 1m =14
Tau = 1m =11
Tau = 1m =12
Tau = 1m =11
Tau = 1m =12
Target Varience
m
DVV Plot
Original
Surrogates:0.19803
Surrogates:0.42044
Surrogates:0.32367
Surrogates:0.054169
Surrogates:0.29241
variance
std. distance
DVV Scatter Plot
DVV Scatter Plot
RSME:0.13487
RSME:0.35517
RSME:0.20374
RSME:0.11813
RSME:0.18282
Surrogates
Original

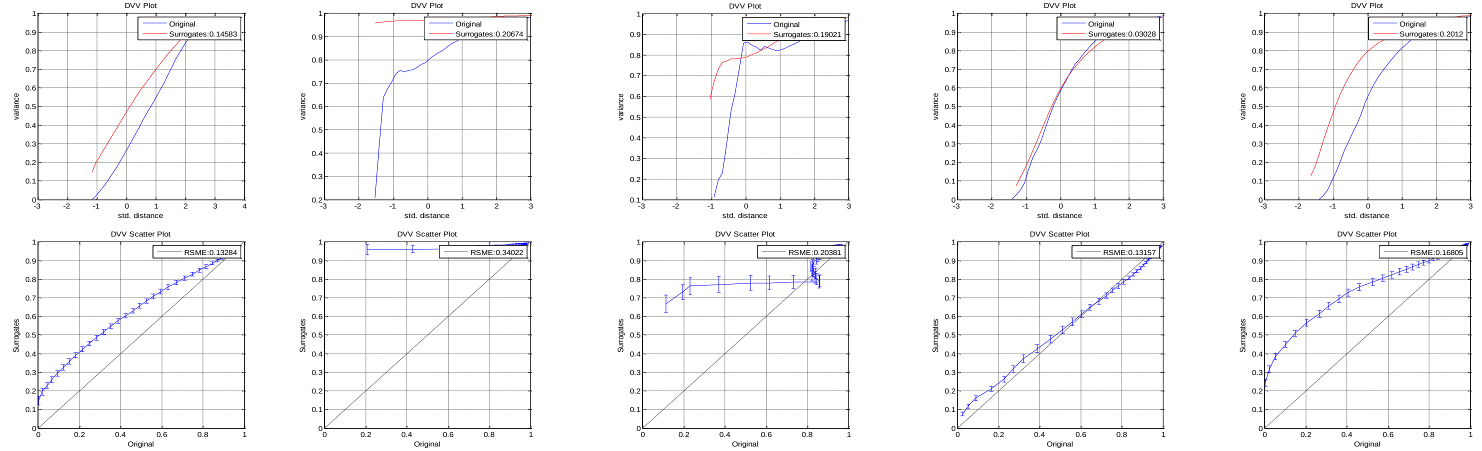
DVV Plot
Original
Surrogates:0.14583
Surrogates:0.20674
Surrogates:0.19021
Surrogates:0.03028
Surrogates:0.2012
variance
std. distance
DVV Scatter Plot
RSME:0.13284
RSME:0.34022
RSME:0.20381
RSME:0.13157
RSME:0.16805
Surrogates
Original

EXPERIMENT	SYSTEM CHARACTERISTCS	VARIABLES		METHOD 1				METHOD 2				METHOD 3			
				best **m**	best **τ**	rsme	RSME	calc **m**	**τ**	rsme	RSME	set **m**	set **τ**	rsme	RSME
11	SDOF CAR attached to fixed supports by 4 calibrated springs (2 on each side)	surface sand paper (rough) Middle spring taken out number of springs 2 x2; loading harmonic 2-4-6-8-10 Hz	CH1	4	10	0.3958	0.2010	10	1	0.2735	0.1514	3	1	0.2079	0.1548
			CH2	10	1	0.6419	0.3674	20	1	0.6193	0.3486	3	1	0.5733	0.3291
			CH3	10	1	0.2980	0.1626	19	1	0.2564	0.1484	3	1	0.3044	0.1883
			LDVg	8	6	0.2343	0.1855	14	1	0.0675	0.1175	3	1	0.0235	0.1102
			LDV1	7	5	0.2680	0.1687	14	1	0.1438	0.1134	3	1	0.0710	0.0978

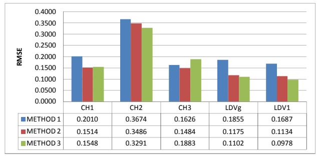

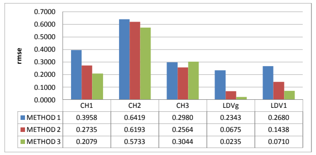

Data recorded 3D Accelerometer

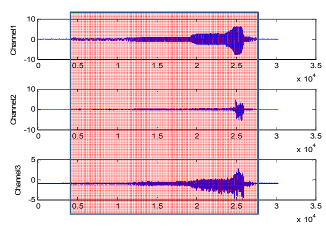

Data analysed 3D Accelerometer

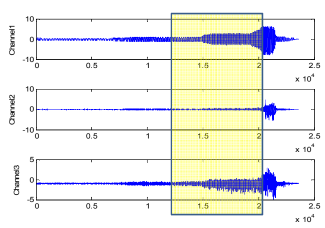

Data recorded LDV

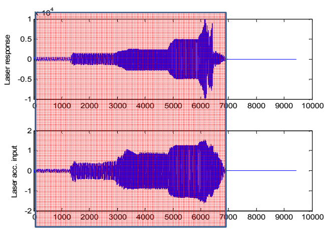

Data analysed LDV

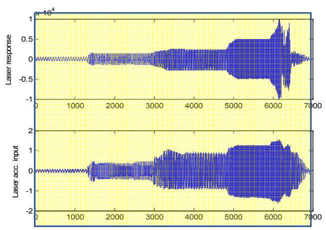

CH1 **CH2** **CH3** **LDVg** **LDV1**

METHOD 1

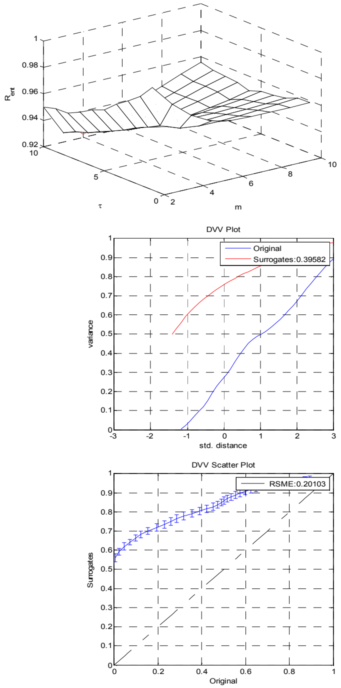

CH1

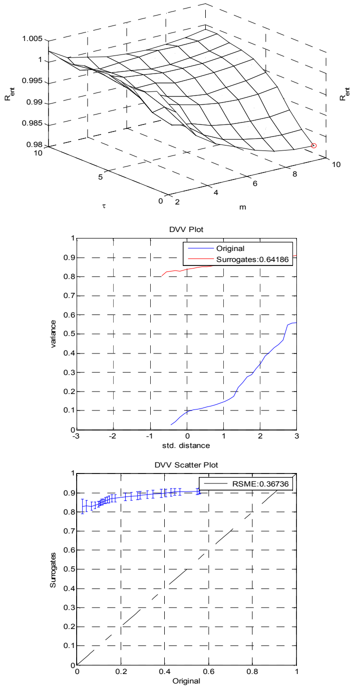

CH2

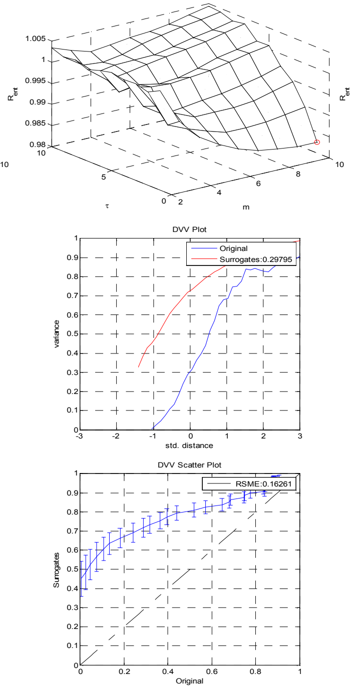

CH3

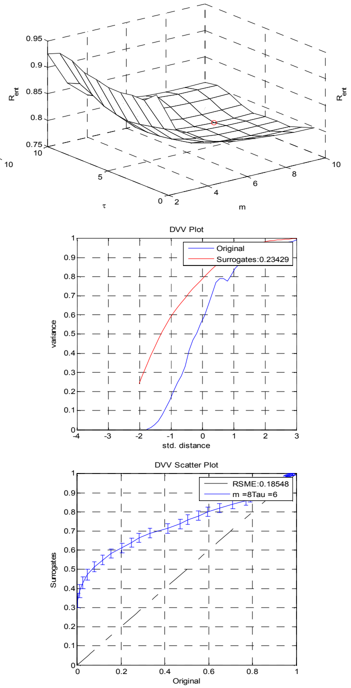

LDVg

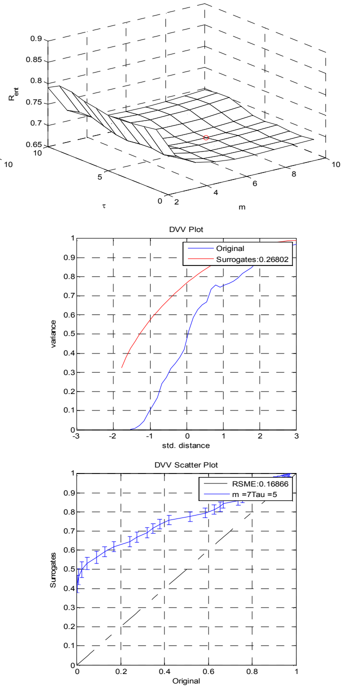

LDV1

CH1 CH2 CH3 LDVg LDV1

EXP 11 METHOD 2

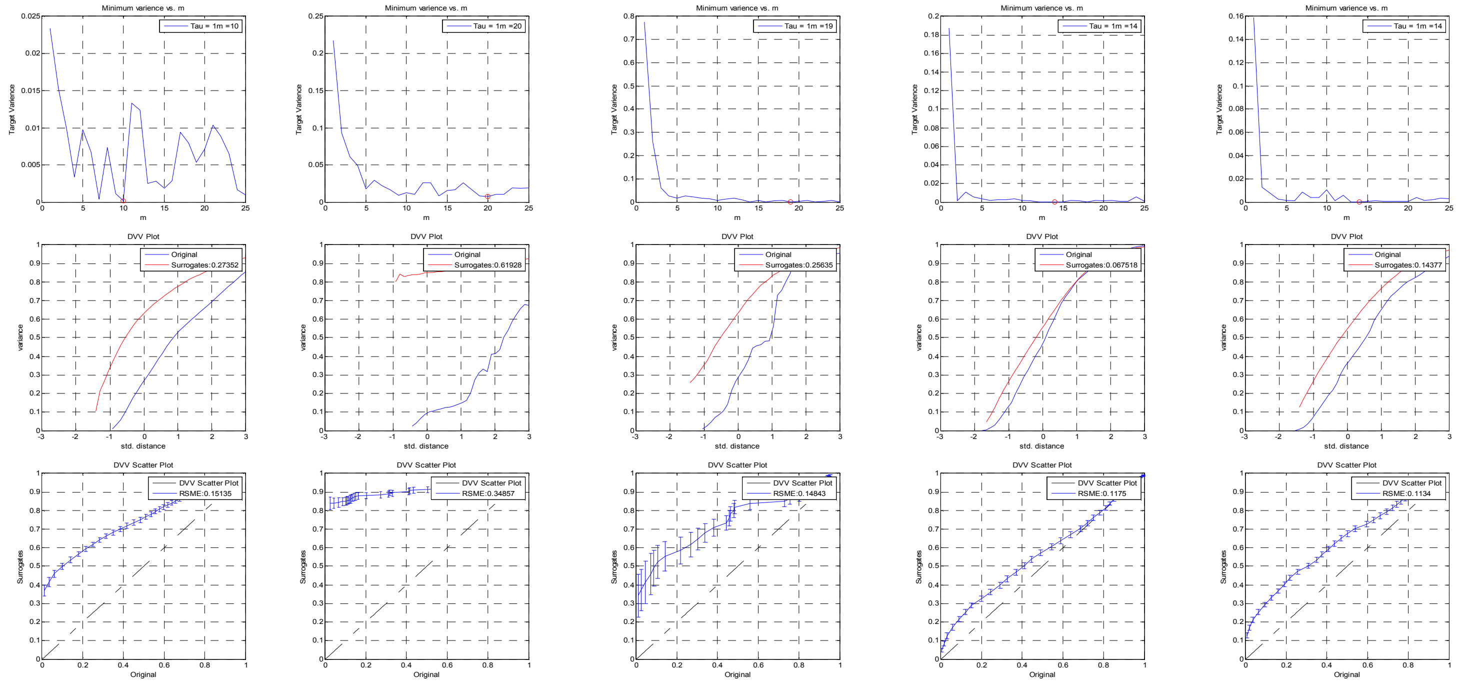

CH1 CH2 CH3 LDVg LDV1

METHOD 3

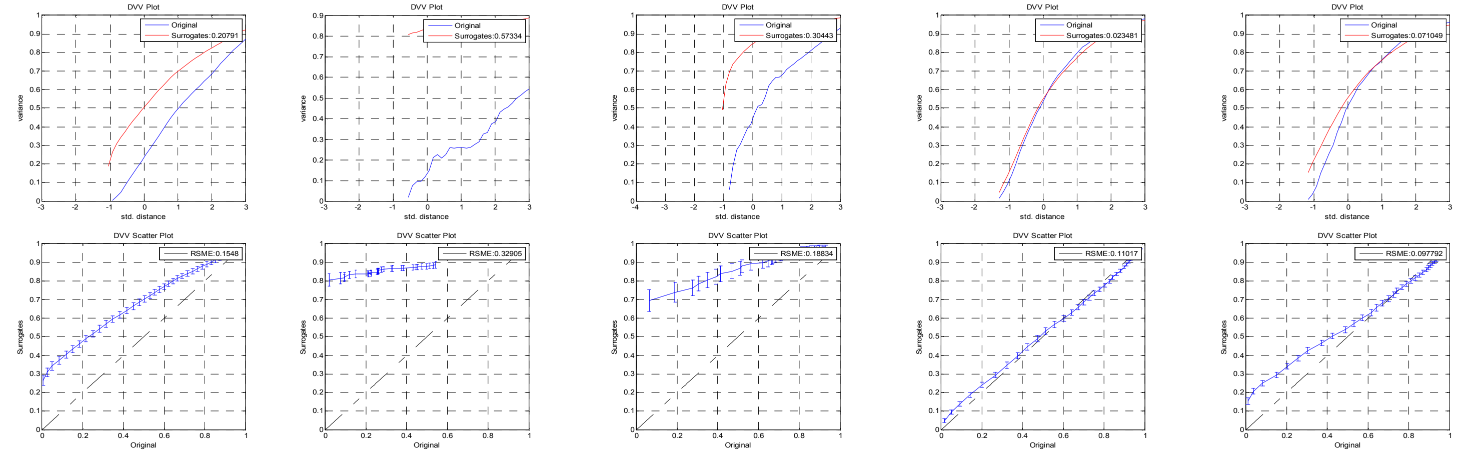

EXPERIMENT	SYSTEM CHARACTERISTCS	VARIABLES		METHOD 1				METHOD 2				METHOD 3			
				best **m**	best **τ**	rsme	RSME	calc **m**	**τ**	rsme	RSME	set **m**	set **τ**	rsme	RSME
12	SDOF CAR attached to fixed supports by 4 calibrated springs (2 on each side)	surface sand paper (rough) Middle spring taken out number of springs 2 x2; loading Sine Sweep	CH1	4	10	0.2691	0.1546	9	1	0.1486	0.1102	3	1	0.1325	0.1415
			CH2	10	3	0.5232	0.2775	15	1	0.5250	0.2838	3	1	0.4094	0.2149
			CH3	10	1	0.3269	0.1693	14	1	0.3390	0.1803	3	1	0.1057	0.1350
			LDVg	8	3	0.1022	0.1263	8	1	0.0302	0.1164	3	1	0.0276	0.1029
			LDV1	7	7	0.1367	0.1063	24	1	0.0805	0.1195	3	1	0.0141	0.0981

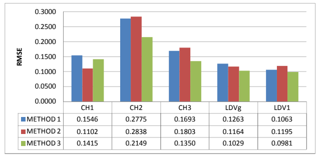

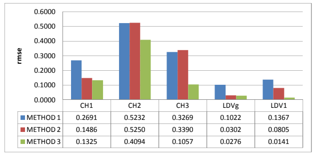

Data recorded 3D Accelerometer

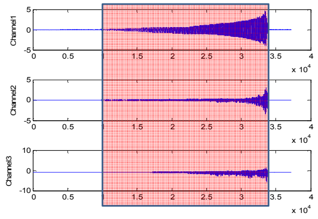

Data analysed 3D Accelerometer

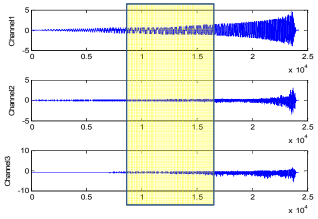

Data recorded LDV

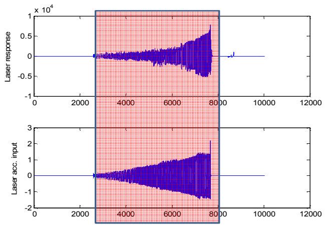

Data analysed LDV

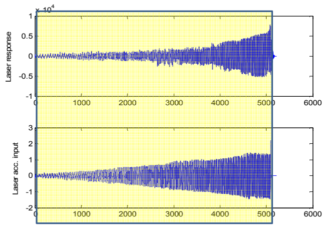

CH1 **CH2** **CH3** **LDVg** **LDV1**

METHOD 1

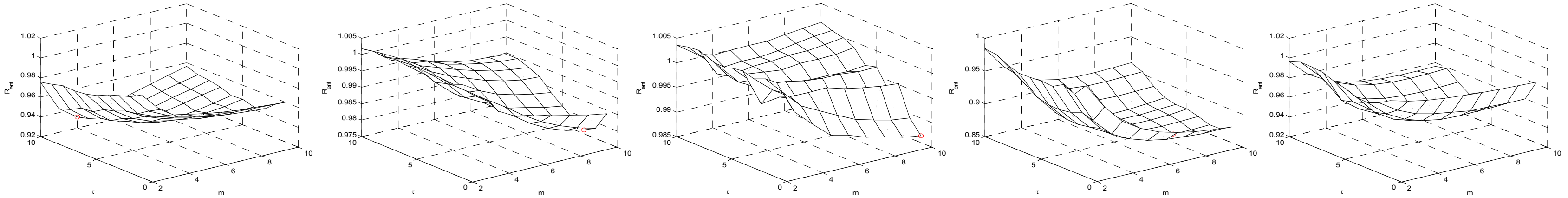

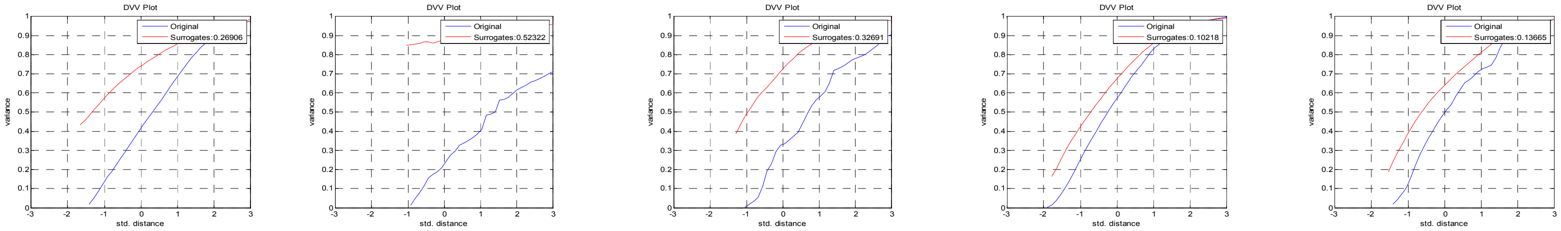

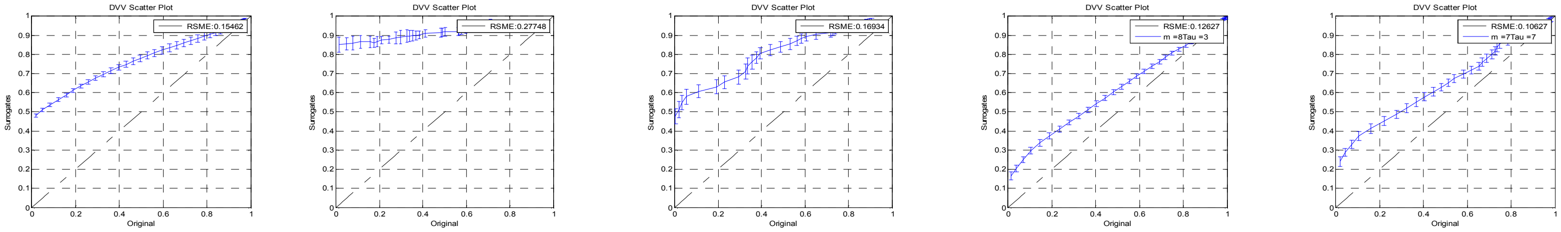

CH1 **CH2** **CH3** **LDVg** **LDV1**

EXP 12 METHOD 2

CH1 | CH2 | CH3 | LDVg | LDV1

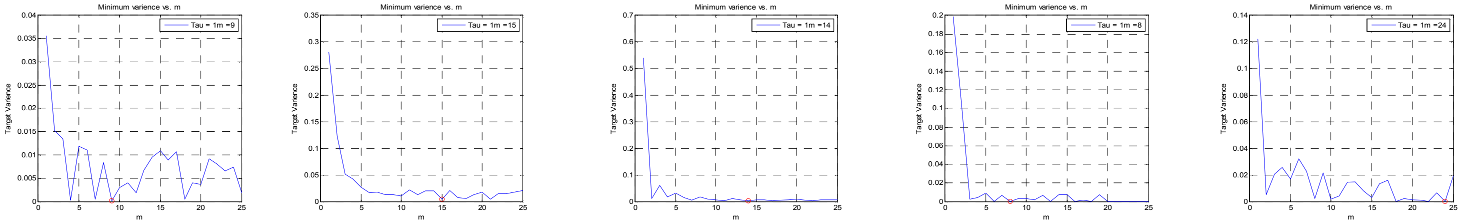

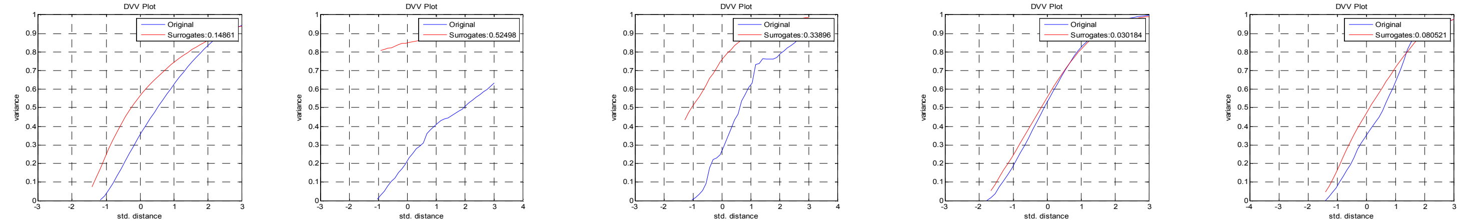

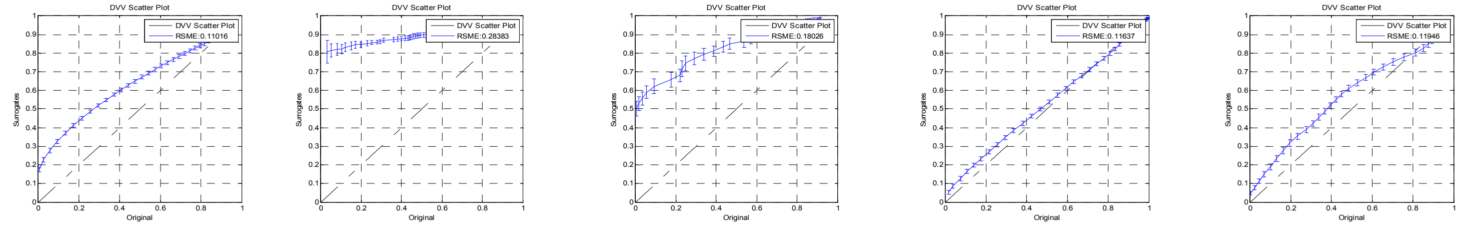

METHOD 3

CH1 | CH2 | CH3 | LDVg | LDV1

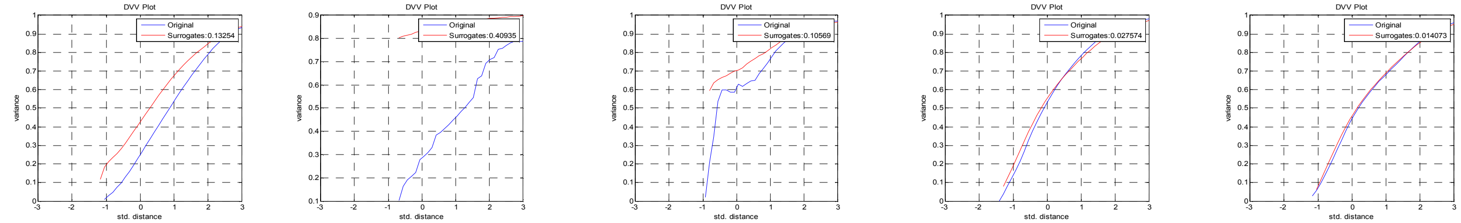

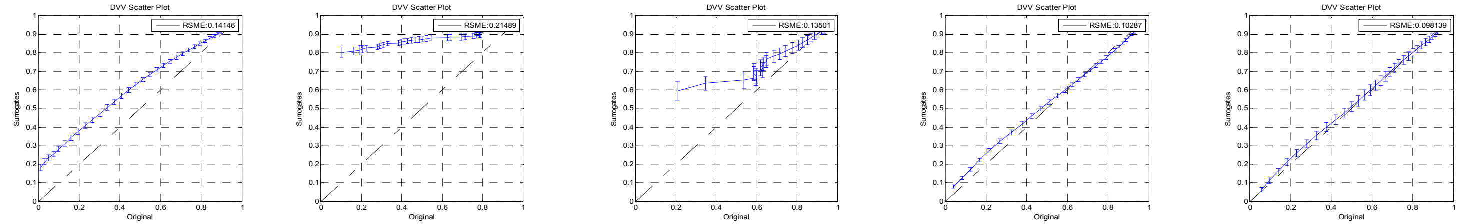

EXPERIMENT	SYSTEM CHARACTERISTCS	VARIABLES		METHOD 1				METHOD 2				METHOD 3			
				best **m**	best **τ**	rsme	RSME	calc **m**	**τ**	rsme	RSME	set **m**	set **τ**	rsme	RSME
13	SDOF CAR attached to fixed supports by 4 calibrated springs (2 on each side) REPEATED EXPERIMENT NO.12	surface sand paper (rough) Middle spring taken out number of springs 2 x2; loading Sine Sweep	CH1	4	10	0.2932	0.1736	19	1	0.1795	0.1250	3	1	0.1026	0.1393
			CH2	10	3	0.4319	0.3034	13	1	0.4338	0.2673	3	1	0.2973	0.2284
			CH3	9	1	0.2023	0.1111	15	1	0.2177	0.1216	3	1	0.1123	0.0981
			LDVg	8	3	0.1056	0.1260	20	1	0.0875	0.1072	3	1	0.0357	0.1047
			LDV1	7	5	0.1701	0.1632	12	1	0.2371	0.1559	3	1	0.1701	0.1410

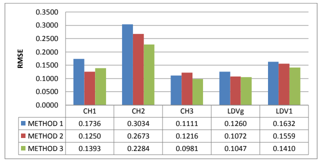

	CH1	CH2	CH3	LDVg	LDV1
METHOD 1	0.1736	0.3034	0.1111	0.1260	0.1632
METHOD 2	0.1250	0.2673	0.1216	0.1072	0.1559
METHOD 3	0.1393	0.2284	0.0981	0.1047	0.1410

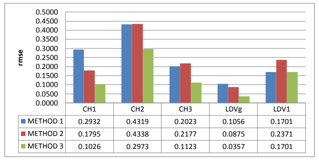

	CH1	CH2	CH3	LDVg	LDV1
METHOD 1	0.2932	0.4319	0.2023	0.1056	0.1701
METHOD 2	0.1795	0.4338	0.2177	0.0875	0.2371
METHOD 3	0.1026	0.2973	0.1123	0.0357	0.1701

Data recorded 3D Accelerometer

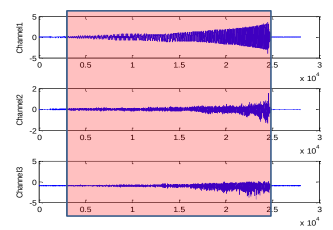

Data analysed 3D Accelerometer

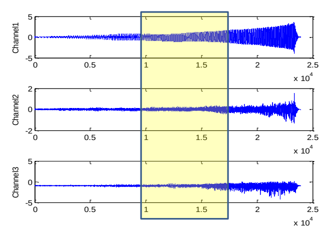

Data recorded LDV

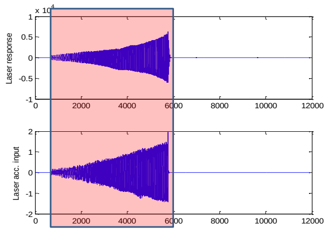

Data analysed LDV

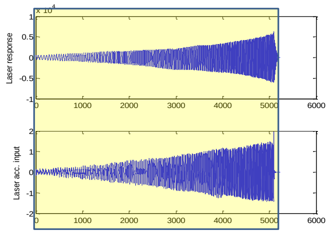

CH1 **CH2** **CH3** **LDVg** **LDV1**

METHOD 1

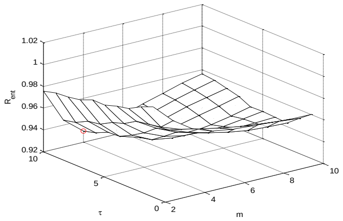

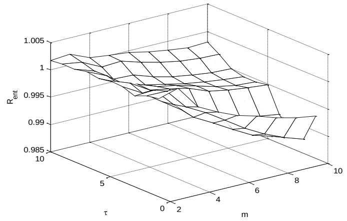

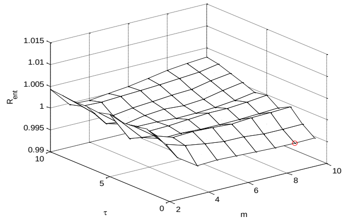

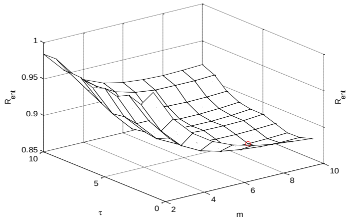

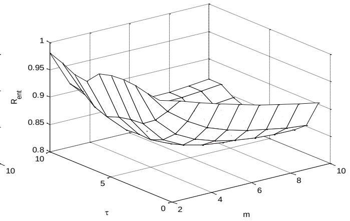

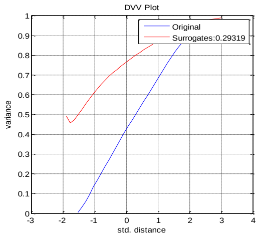

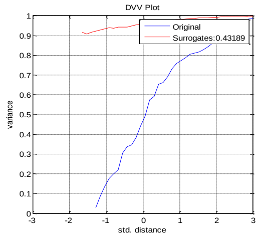

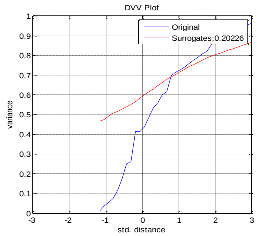

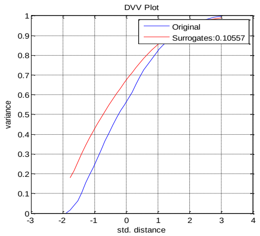

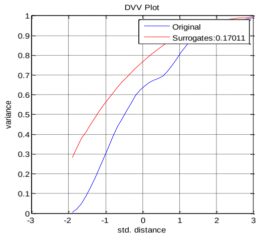

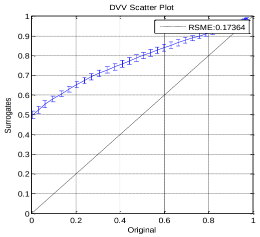

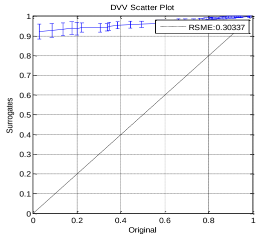

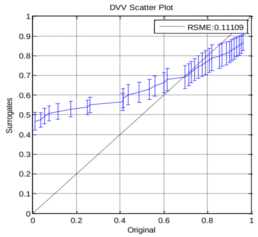

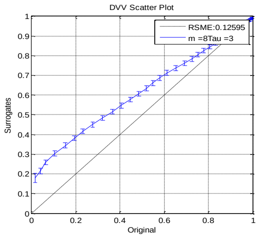

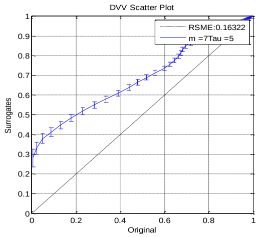

CH1 **CH2** **CH3** **LDVg** **LDV1**

CH1 CH2 CH3 LDVg LDV1

EXP 13 **METHOD 2**

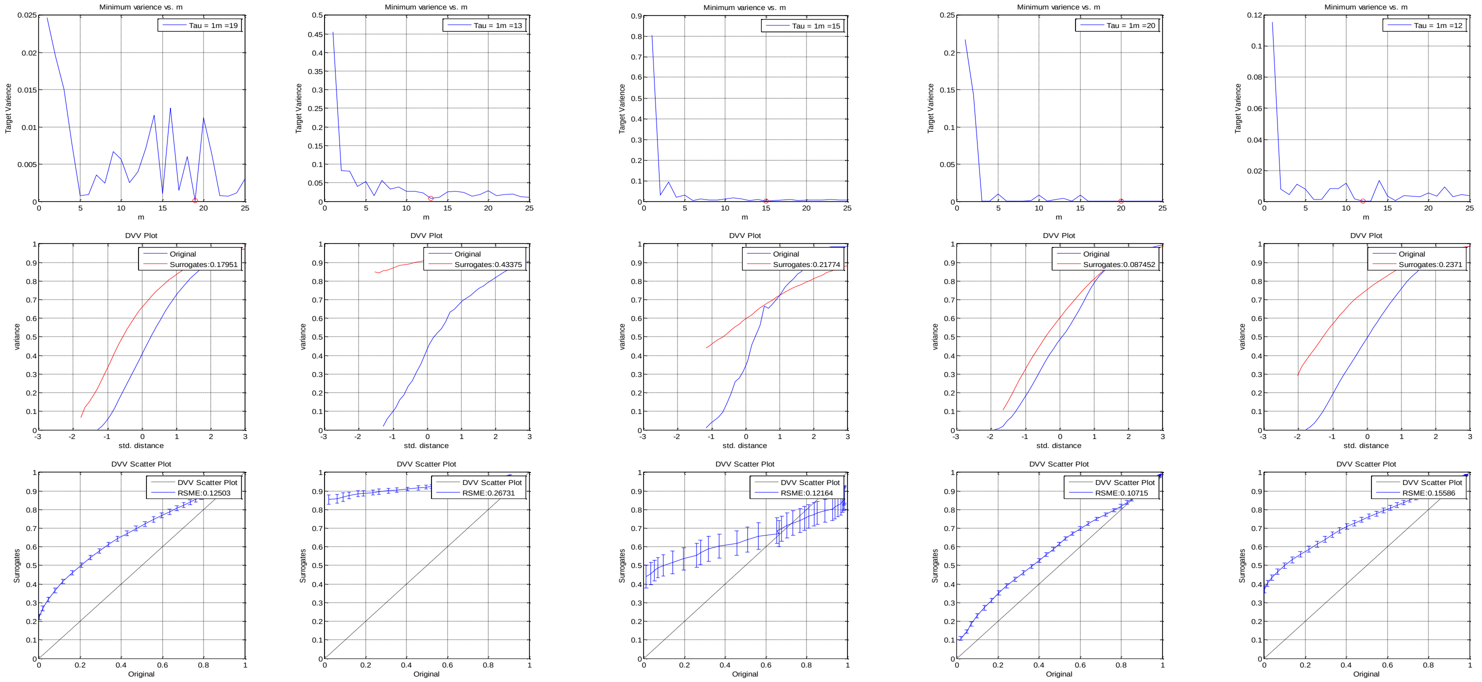

CH1 CH2 CH3 LDVg LDV1

METHOD 3

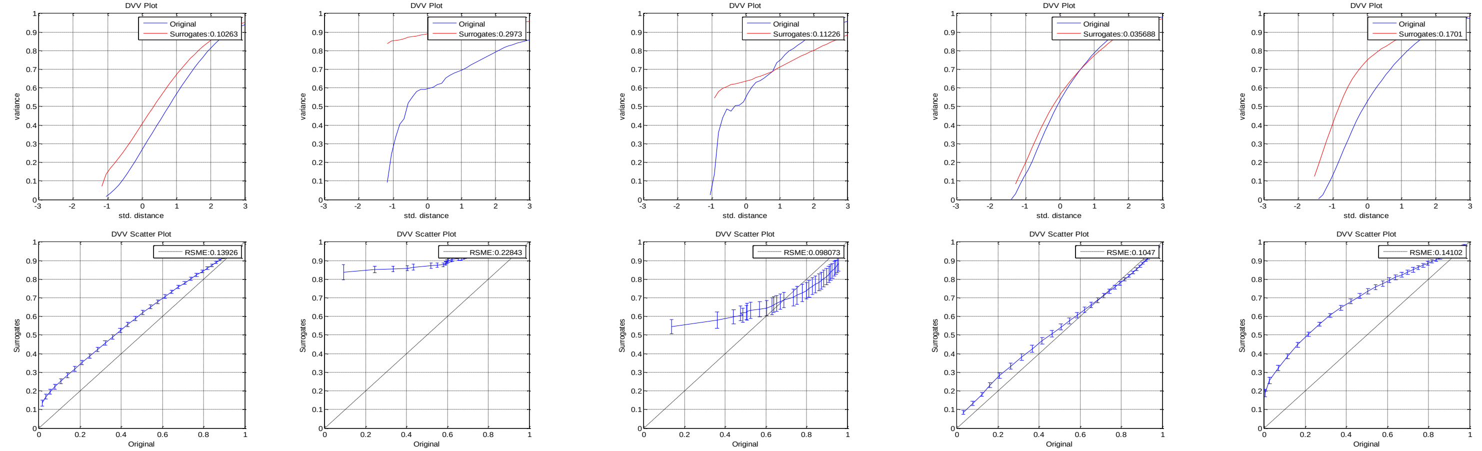

EXPERIMENT	SYSTEM CHARACTERISTCS	VARIABLES		METHOD 1				METHOD 2				METHOD 3			
				best **m**	best **τ**	rsme	RSME	calc **m**	**τ**	rsme	RSME	set **m**	set **τ**	rsme	RSME
14	SDOF CAR attached to fixed supports by 4 calibrated springs (2 on each side)	surface sand paper (rough) Middle spring taken out number of springs 2 x2; loading White Noise	CH1	6	1	0.1597	0.1052	21	1	0.1710	0.1335	3	1	0.1340	0.1064
			CH2	5	1	0.3213	0.3289	14	1	0.4115	0.3509	3	1	0.2359	0.3252
			CH3	3	6	0.0762	0.2905	25	1	0.1015	0.1225	3	1	0.0583	0.1759
			LDVg	6	1	0.0144	0.2840	4	1	0.1934	0.3564	3	1	0.2385	0.3479
			LDV1	4	1	0.2109	0.1760	8	1	0.2282	0.1448	3	1	0.1994	0.1780

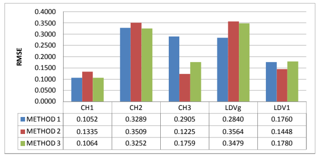

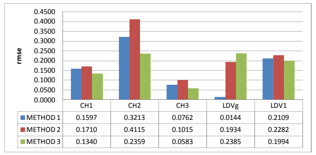

Data recorded 3D Accelerometer

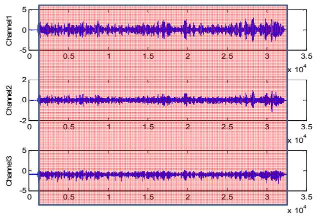

Data analysed 3D Accelerometer

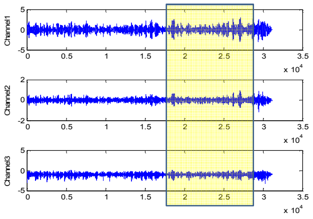

Data recorded LDV

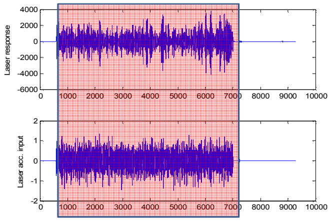

Data analysed LDV

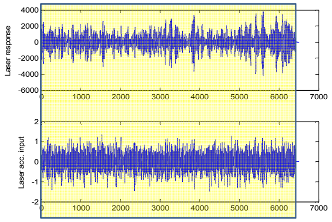

CH1 **CH2** **CH3** **LDVg** **LDV1**

METHOD 1

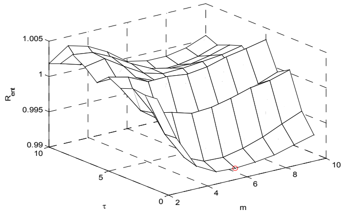
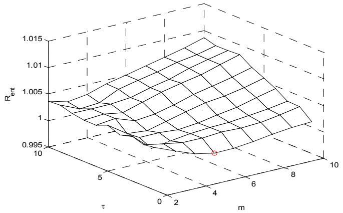
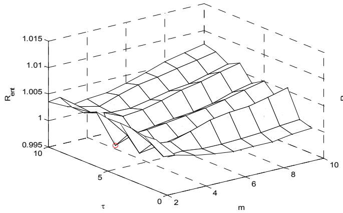
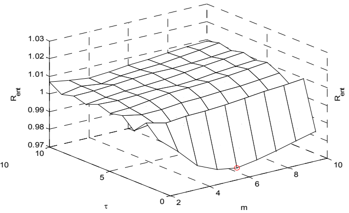
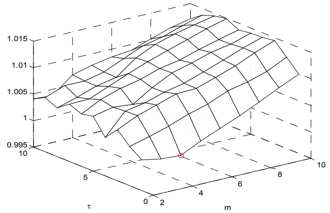

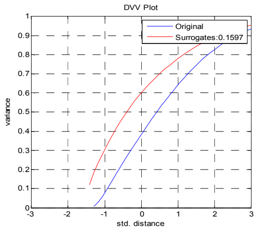

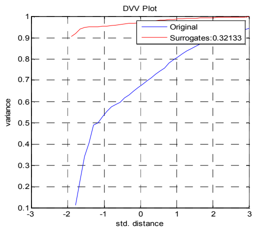

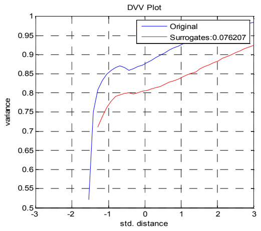

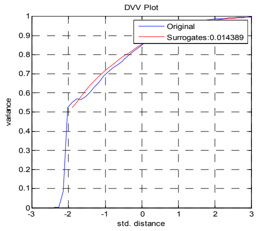

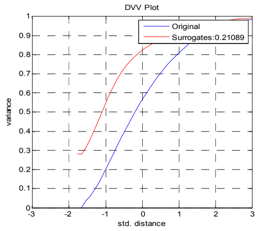

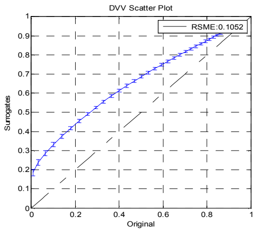

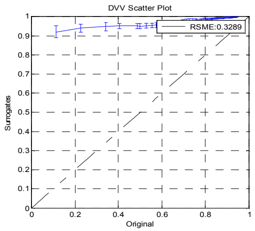

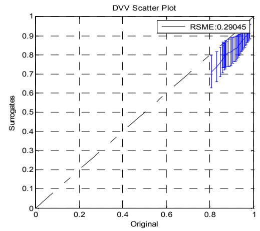

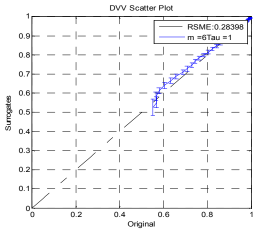

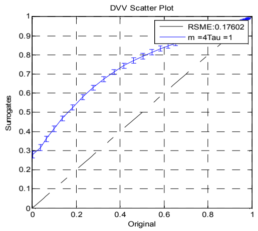

CH1 **CH2** **CH3** **LDVg** **LDV1**

CH1 CH2 CH3 LDVg LDV1

EXP 14 METHOD 2

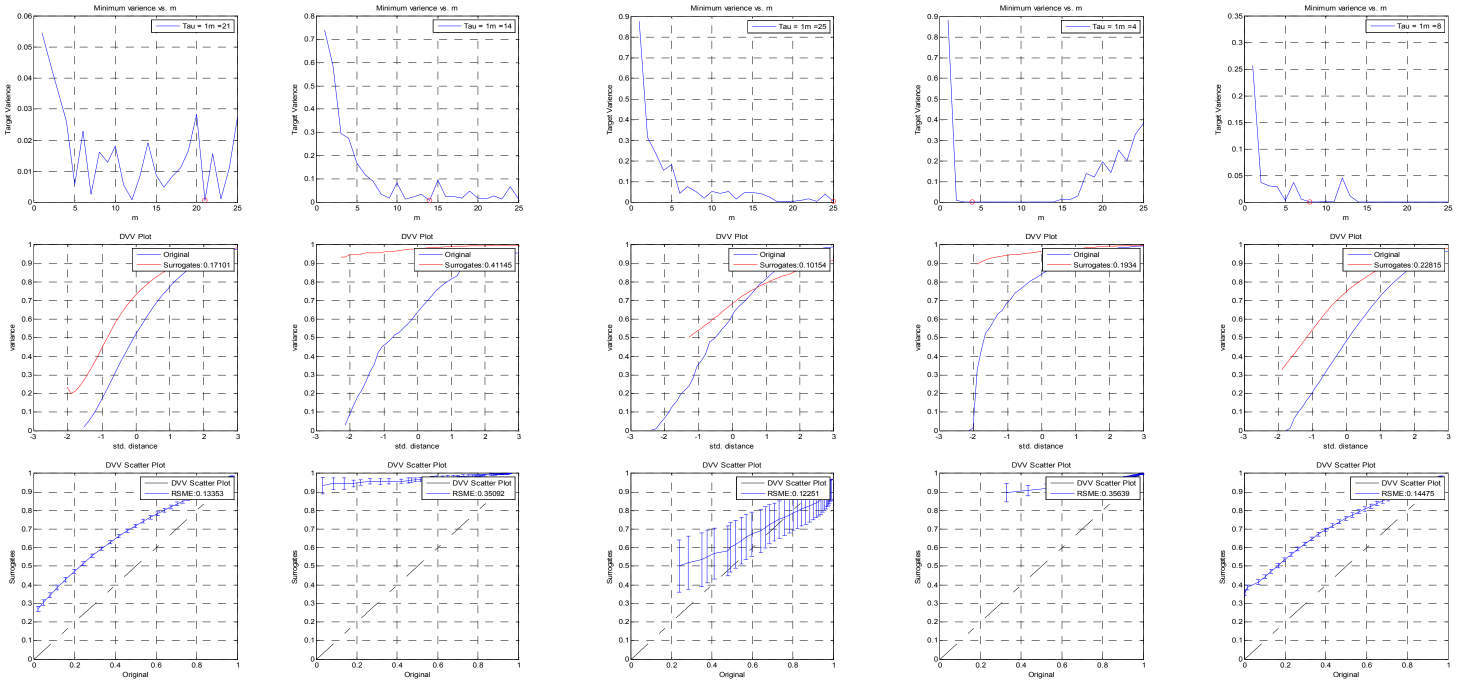

CH1 CH2 CH3 LDVg LDV1

METHOD 3

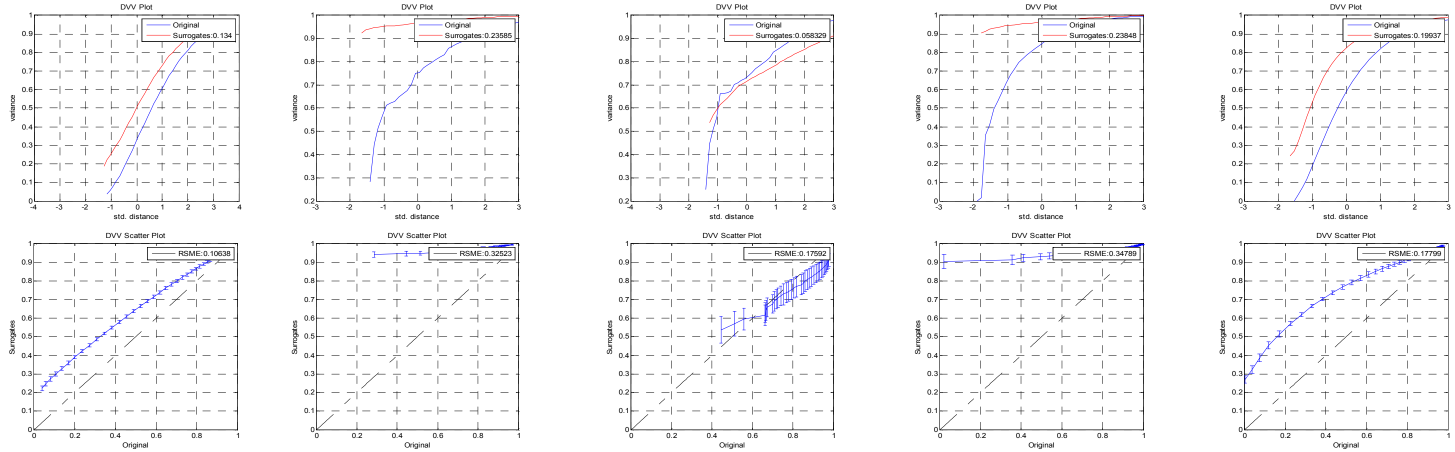

EXPERIMENT	SYSTEM CHARACTERISTCS	VARIABLES		METHOD 1				METHOD 2				METHOD 3			
				best **m**	best **τ**	rsme	RSME	calc **m**	**τ**	rsme	RSME	set **m**	set **τ**	rsme	RSME
15	SDOF CAR attached to fixed supports by 4 calibrated springs (2 on each side)	surface plastic (smooth) Middle spring taken out number of springs 2 x2; loading White Noise	CH1	4	1	0.1428	0.1078	7	1	0.1520	0.1053	3	1	0.1312	0.1118
			CH2	4	5	0.1913	0.3615	19	1	0.3416	0.2899	3	1	0.1730	0.2628
			CH3	8	1	0.2986	0.3386	10	1	0.2631	0.2176	3	1	0.1307	0.2297
			LDVg	6	1	0.0212	0.2731	9	1	0.1961	0.4149	3	1	0.2533	0.3451
			LDV1	2	1	0.1941	0.1550	11	1	0.2138	0.1470	3	1	0.1862	0.1727

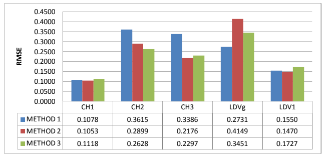

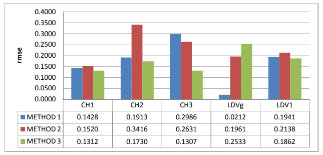

Data recorded 3D Accelerometer

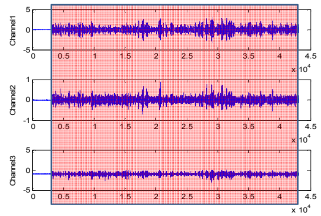

Data analysed 3D Accelerometer

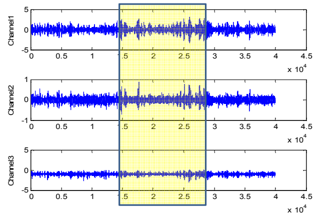

Data recorded LDV

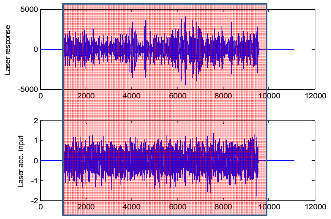

Data analysed LDV

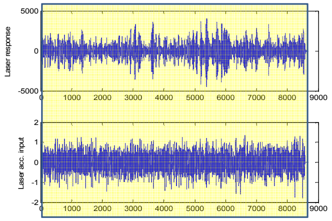

CH1 CH2 CH3 LDVg LDV1

METHOD 1

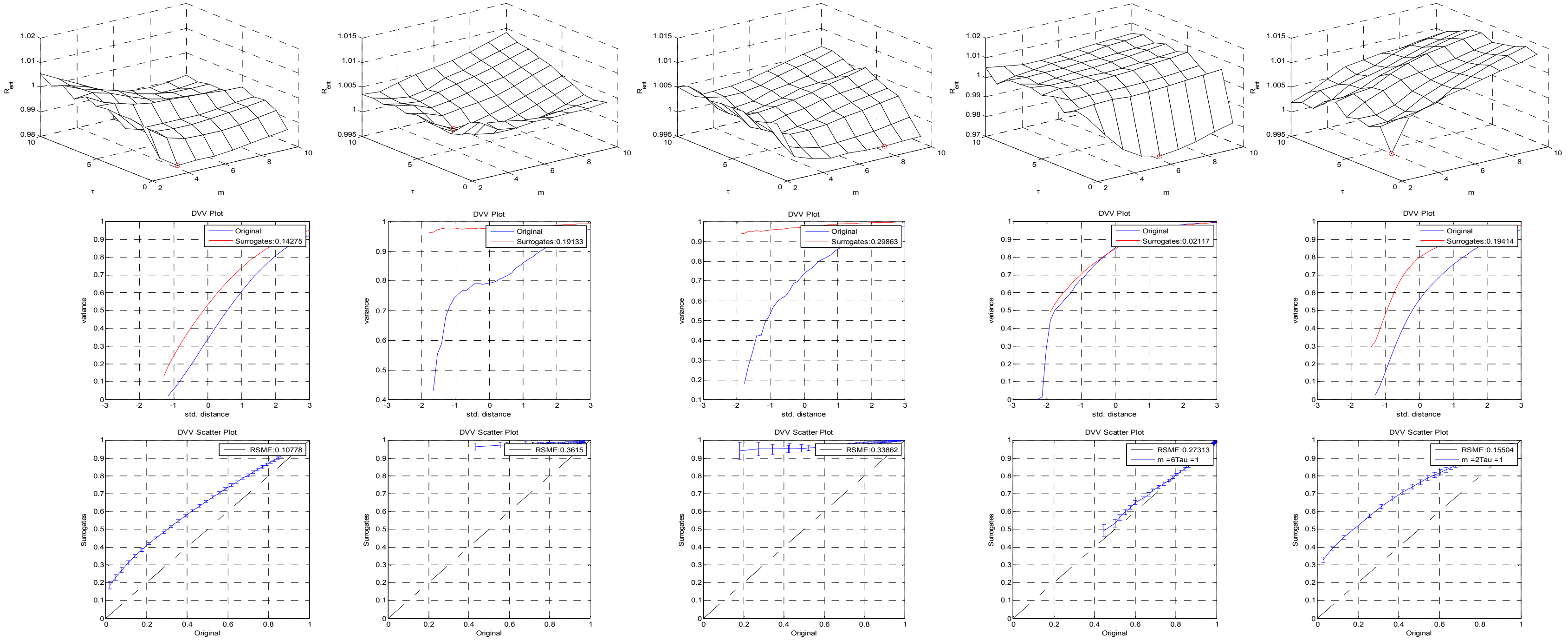

CH1 CH2 CH3 LDVg LDV1

EXP **15** **METHOD 2**

CH1 CH2 CH3 LDVg LDV1

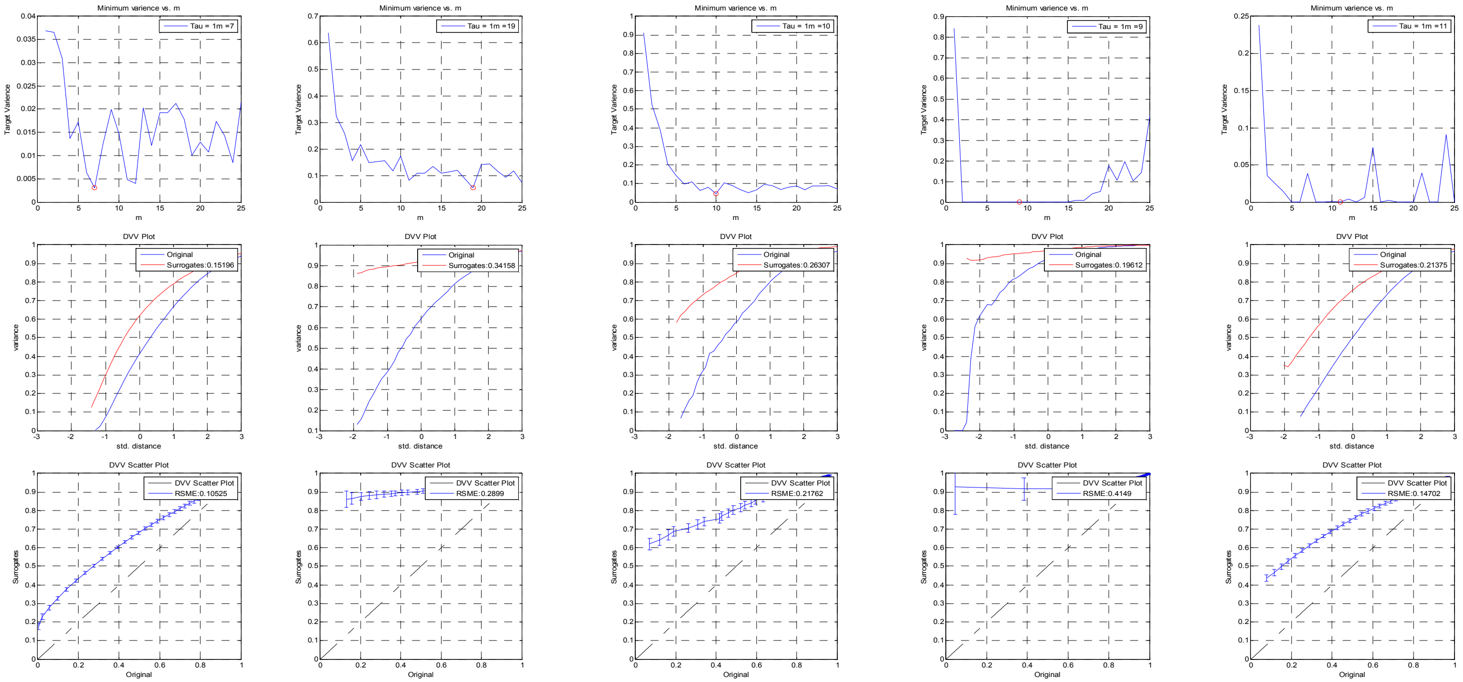

METHOD 3

CH1 CH2 CH3 LDVg LDV1

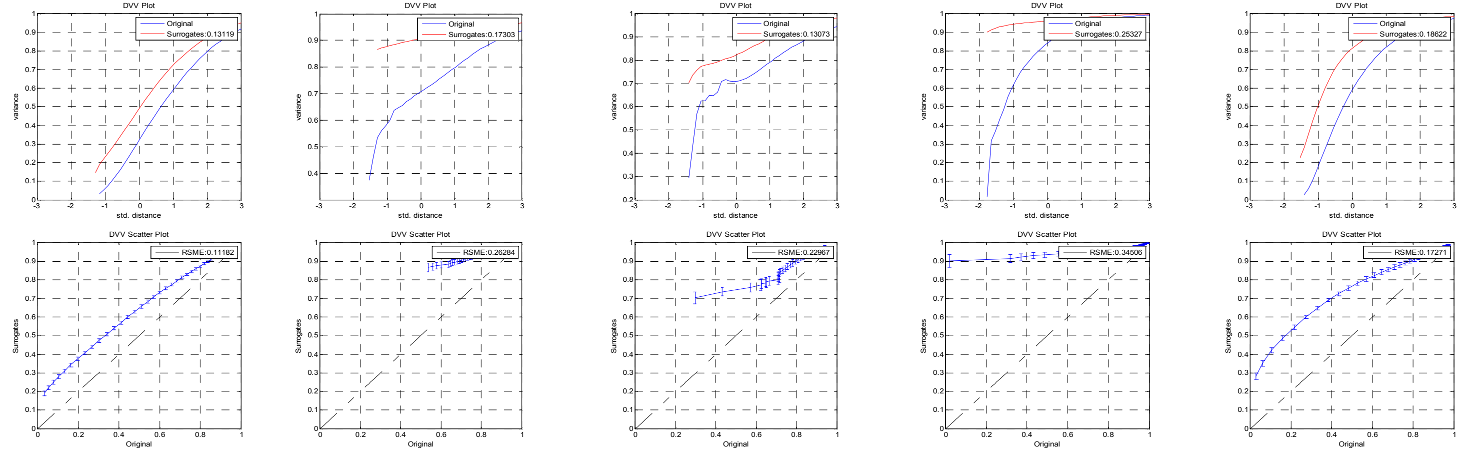

EXPERIMENT	SYSTEM CHARACTERISTCS	VARIABLES		METHOD 1				METHOD 2				METHOD 3			
				best **m**	best **τ**	rsme	RSME	calc **m**	**τ**	rsme	RSME	set **m**	set **τ**	rsme	RSME
16	SDOF CAR attached to fixed supports by 4 calibrated springs (2 on each side)	surface plastic (smooth) Middle spring taken out number of springs 2 x2; loading Sine Sweep	CH1	5	9	0.2767	0.1552	17	1	0.1652	0.1195	3	1	0.0990	0.1493
			CH2	10	2	0.3390	0.2481	13	1	0.3118	0.2162	3	1	0.2198	0.2020
			CH3	10	1	0.3098	0.1654	18	1	0.3506	0.1856	3	1	0.1513	0.1137
			LDVg	8	3	0.0936	0.1236	19	1	0.0688	0.1058	3	1	0.0306	0.1046
			LDV1	8	5	0.1726	0.1714	21	1	0.2837	0.1924	3	1	0.1688	0.1401

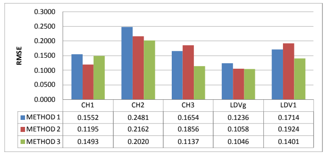

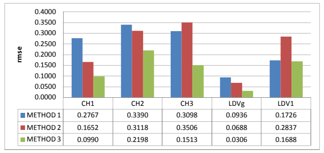

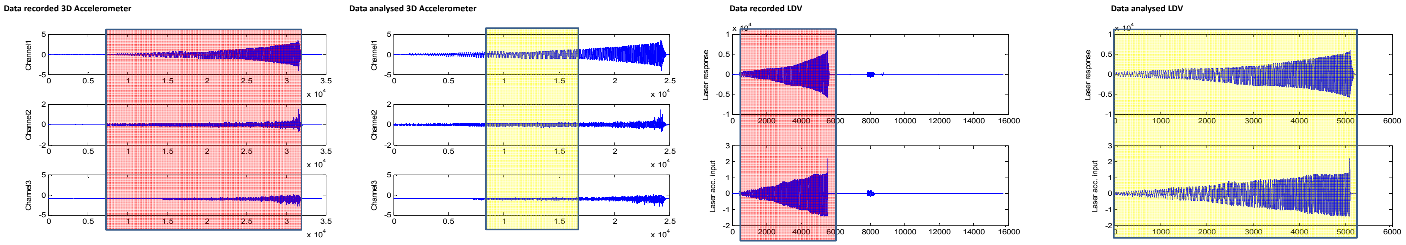

CH1 CH2 CH3 LDVg LDV1

METHOD 1

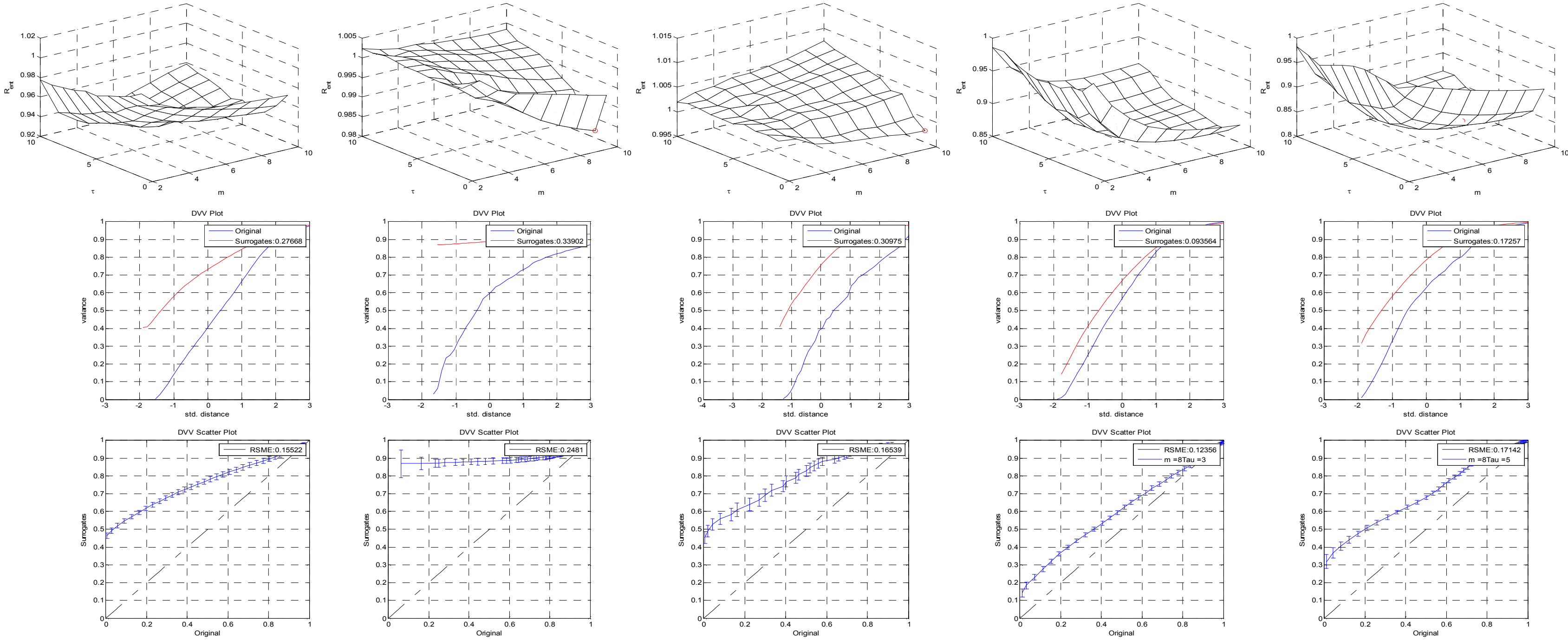

CH1 CH2 CH3 LDVg LDV1

CH1	CH2	CH3	LDVg	LDV1

EXP 16 METHOD 2

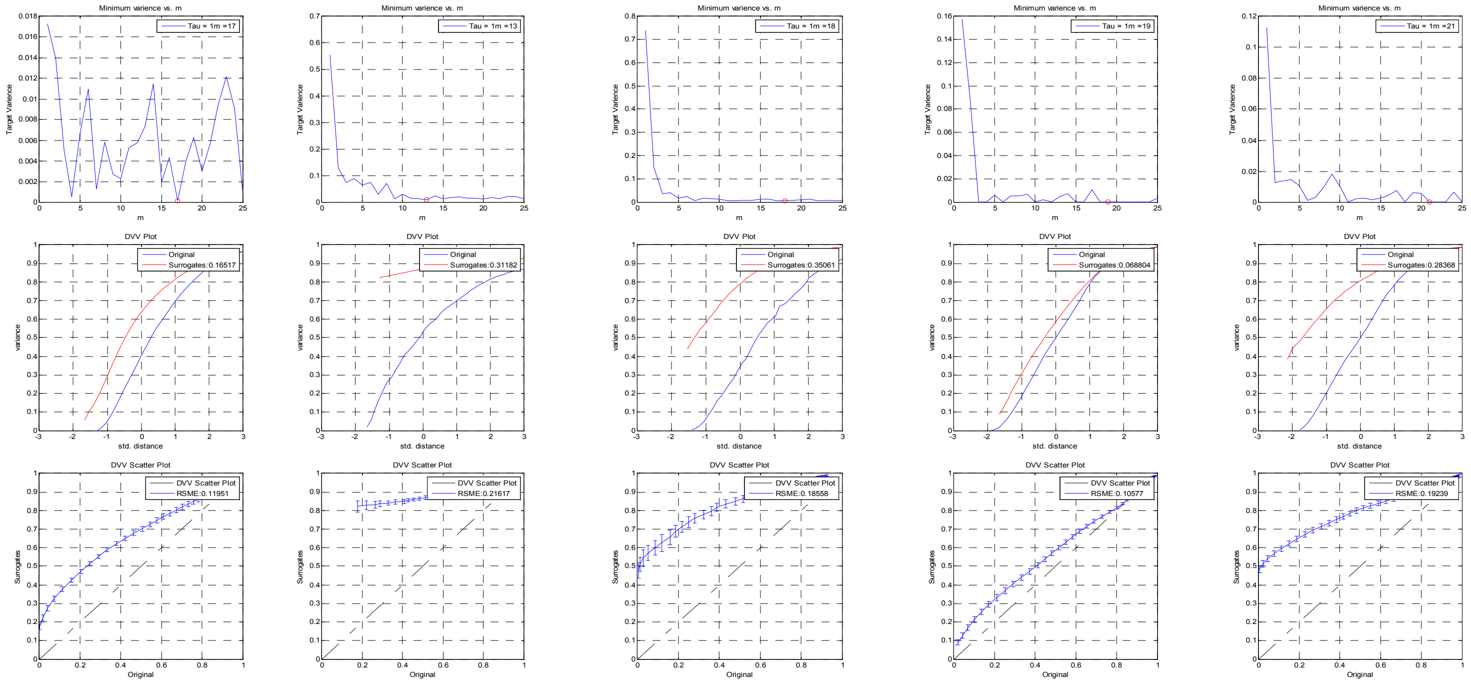

CH1	CH2	CH3	LDVg	LDV1

METHOD 3

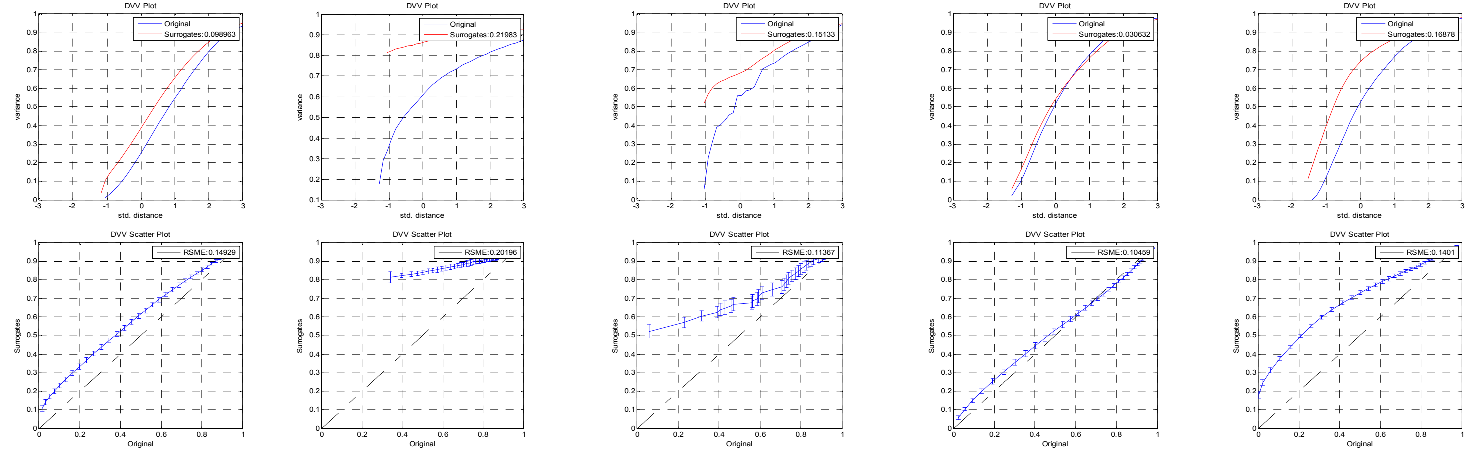

EXPERIMENT	SYSTEM CHARACTERISTCS	VARIABLES		METHOD 1				METHOD 2				METHOD 3			
				best **m**	best **τ**	rsme	RSME	calc **m**	**τ**	rsme	RSME	set **m**	set **τ**	rsme	RSME
17	SDOF CAR attached to fixed supports by 4 calibrated springs (2 on each side)	surface plastic (smooth) Middle spring taken out number of springs 2 x2; loading harmonic 2-4-6-8-10 Hz	CH1						1			3	1		
			CH2						1			3	1		
			CH3						1			3	1		
			LDVg	8	7	0.4169	0.2947	9	1	0.0295	0.1135	3	1	0.0213	0.1132
			LDV1	6	6	0.3819	0.2660	11	1	0.2797	0.1535	3	1	0.2075	0.1473

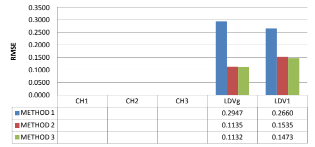

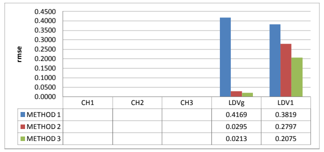

Data recorded 3D Accelerometer

Data analysed 3D Accelerometer

Data recorded LDV

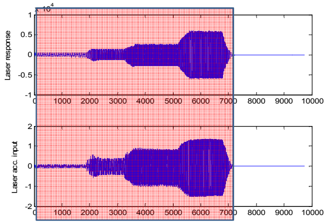

Data analysed LDV

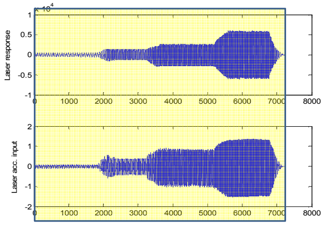

CH1 CH2 CH3 LDVg LDV1

METHOD 1

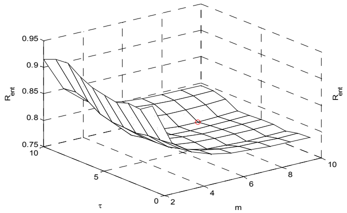

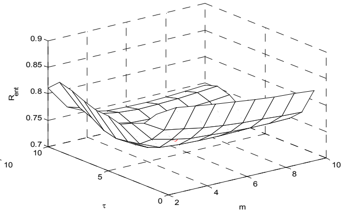

NO DATA

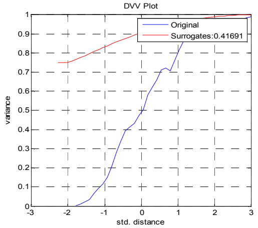

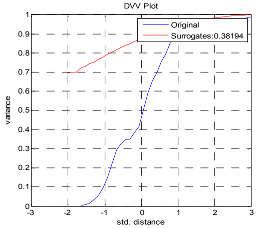

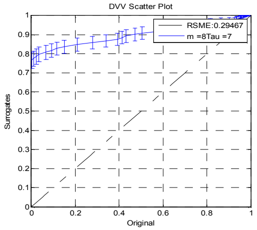

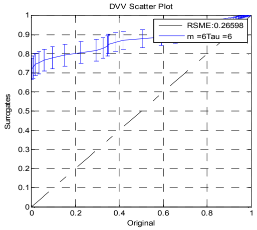

CH1 CH2 CH3 LDVg LDV1

CH1 CH2 CH3 LDVg LDV1

EXP 17 METHOD 2

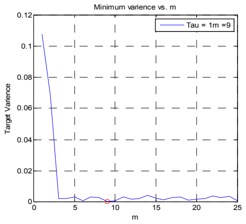

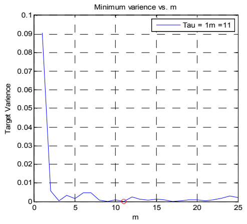

NO DATA

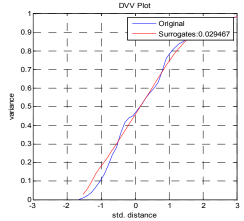

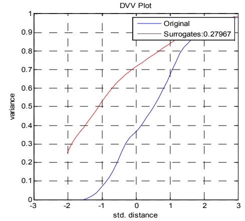

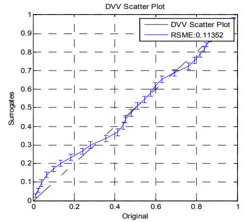

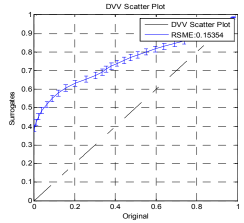

CH1 CH2 CH3 LDVg LDV1

METHOD 3

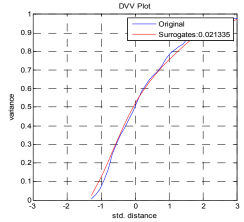

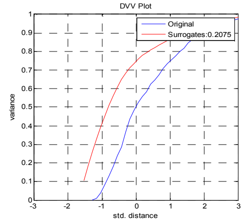

NO DATA

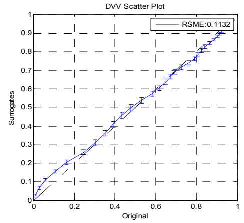

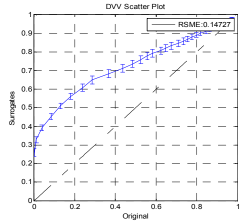

EXPERIMENT	SYSTEM CHARACTERISTCS	VARIABLES		METHOD 1				METHOD 2				METHOD 3			
				best **m**	best **τ**	rsme	RSME	calc **m**	**τ**	rsme	RSME	set **m**	set **τ**	rsme	RSME
18	SDOF CAR attached to fixed supports by 4 calibrated springs (2 on each side)	surface wood<br>Middle spring taken out<br>number of springs 2 x2;<br>loading harmonic 2-4-6-8-10 Hz	CH1	4	10	0.3734	0.1949	2	1	0.2415	0.1973	3	1	0.2609	0.2021
			CH2	9	1	0.6737	0.3737	25	1	0.7082	0.3989	3	1	0.6603	0.3757
			CH3	5	2	0.3453	0.1859	8	1	0.2877	0.1577	3	1	0.2672	0.1497
			LDVg	9	7	0.4325	0.3138	17	1	0.0782	0.1186	3	1	0.0362	0.1067
			LDV1	7	5	0.2536	0.1535	3	1	0.0788	0.0906	3	1	0.0824	0.0909

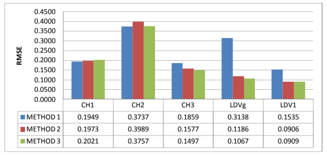

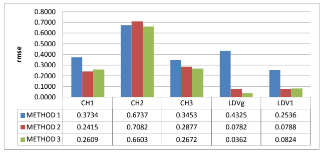

Data recorded 3D Accelerometer

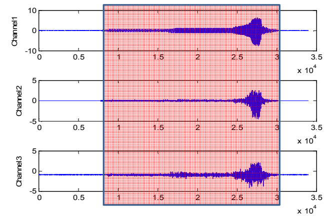

Data analysed 3D Accelerometer

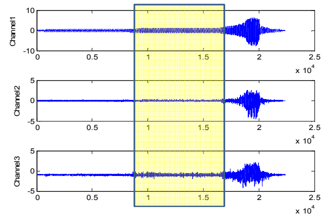

Data recorded LDV

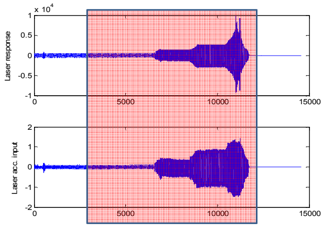

Data analysed LDV

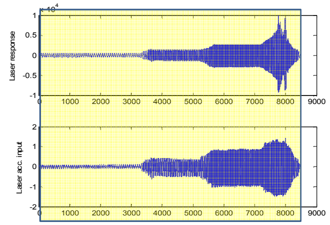

CH1 **CH2** **CH3** **LDVg** **LDV1**

METHOD 1

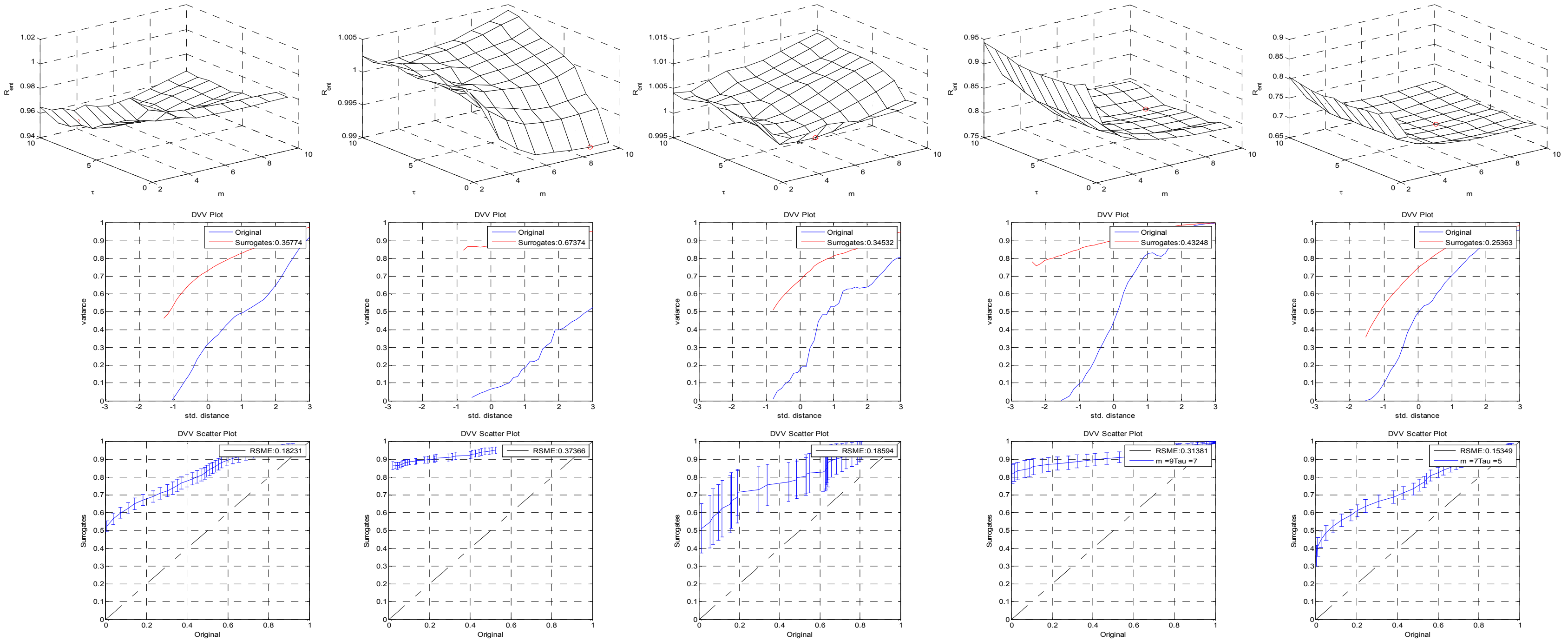

CH1 **CH2** **CH3** **LDVg** **LDV1**

CH1 CH2 CH3 LDVg LDV1

EXP 18 METHOD 2

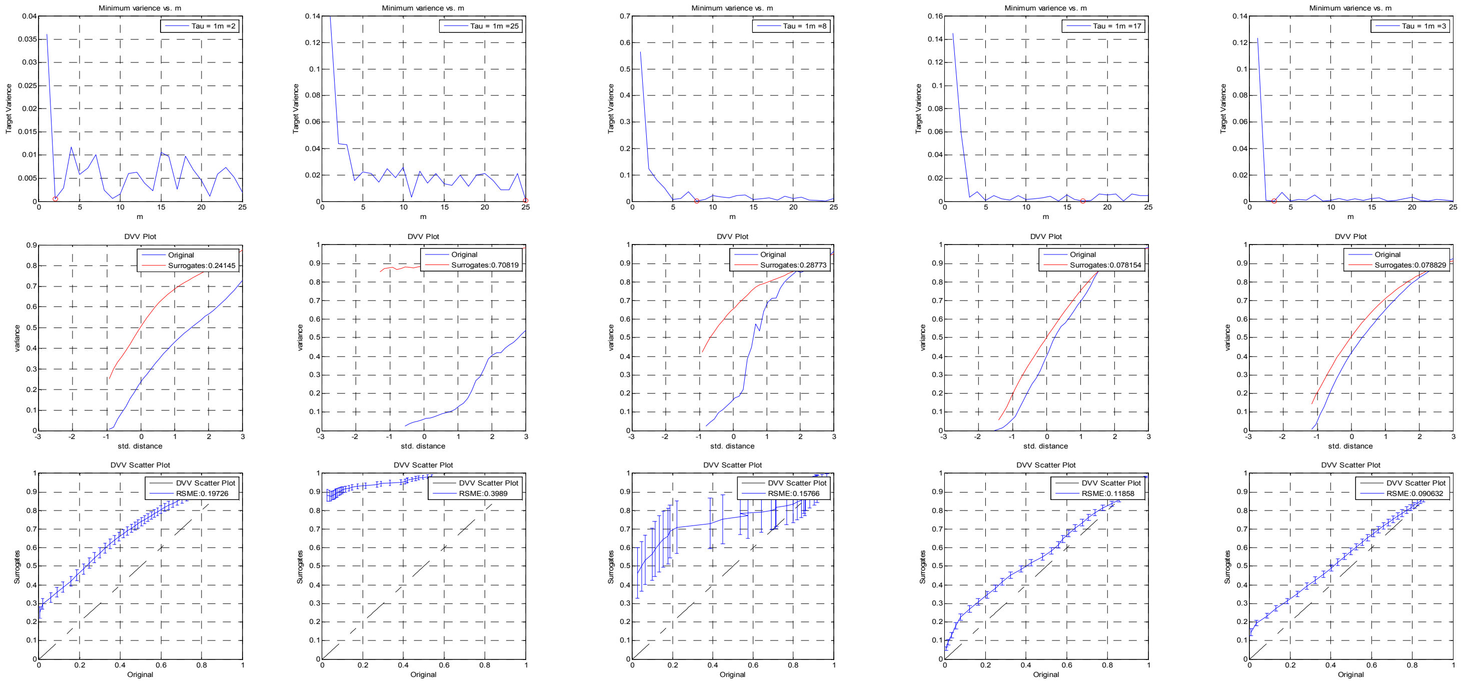

CH1 CH2 CH3 LDVg LDV1

METHOD 3

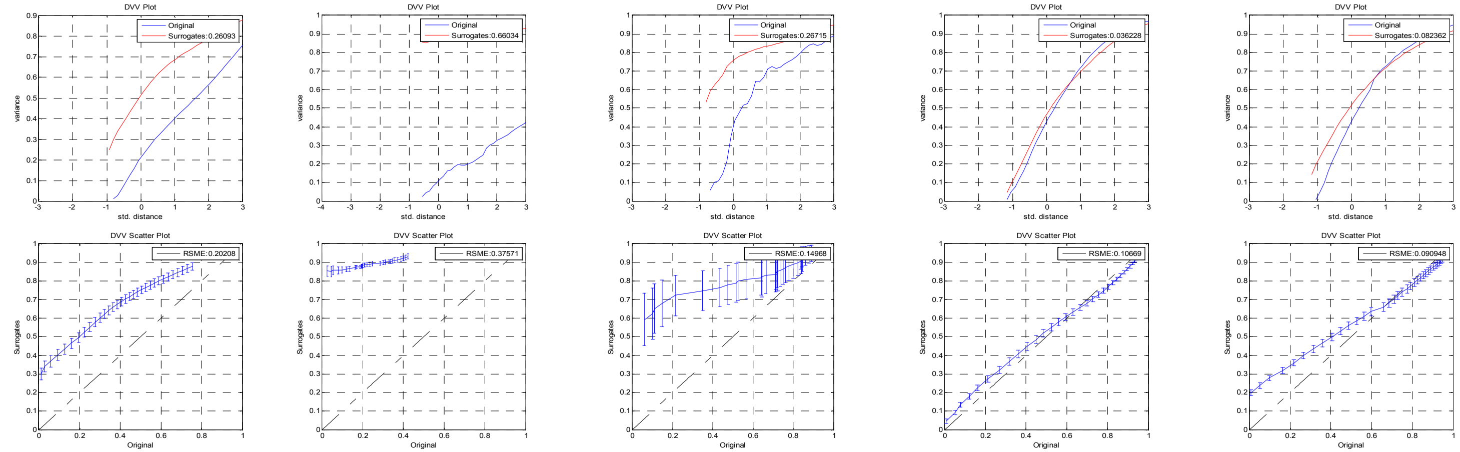

EXPERIMENT	SYSTEM CHARACTERISTCS	VARIABLES		METHOD 1				METHOD 2				METHOD 3			
				best **m**	best **τ**	rsme	RSME	calc **m**	**τ**	rsme	RSME	set **m**	set **τ**	rsme	RSME
19	SDOF CAR attached to fixed supports by 4 calibrated springs (2 on each side)	surface wood Middle spring taken out number of springs 2 x2; loading White Noise	CH1	6	2	0.1763	0.1301	10	1	0.1571	0.1116	3	1	0.1344	0.1141
			CH2	4	1	0.2821	0.3305	22	1	0.4097	0.3801	3	1	0.2458	0.3314
			CH3	4	1	0.2183	0.2895	24	1	0.4016	0.3327	3	1	0.1942	0.2893
			LDVg	6	1	0.2554	0.3742	3	1	0.2775	0.3400	3	1	0.2496	0.3423
			LDV1	2	4	0.2657	0.2551	9	1	0.2023	0.1411	3	1	0.1988	0.1703

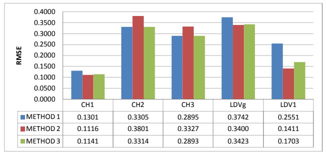

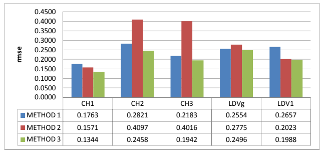

Data recorded 3D Accelerometer

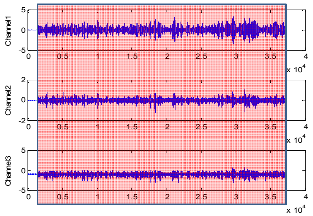

Data analysed 3D Accelerometer

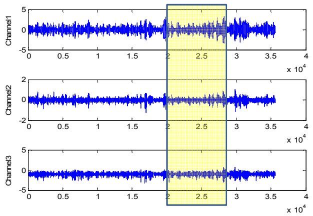

Data recorded LDV

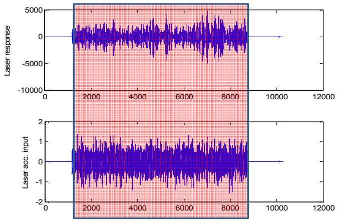

Data analysed LDV

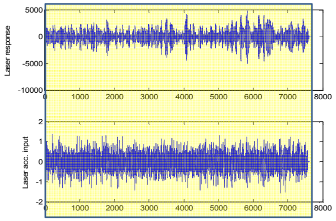

CH1 **CH2** **CH3** **LDVg** **LDV1**

METHOD 1

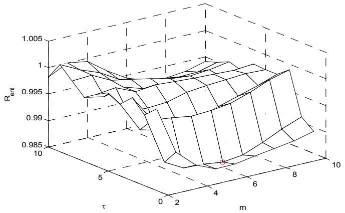

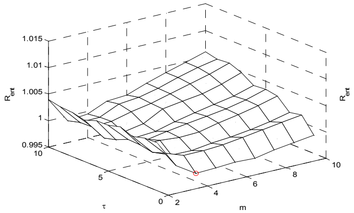

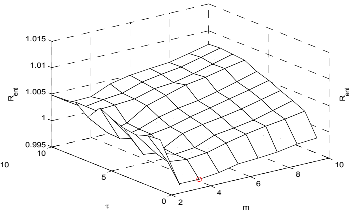

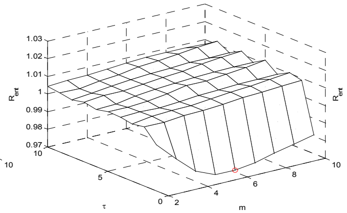

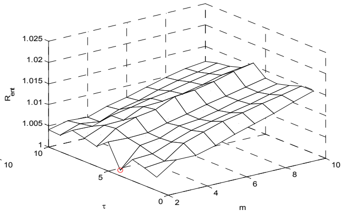

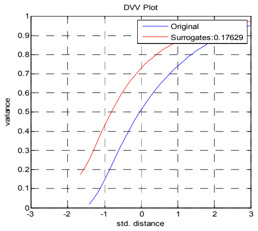

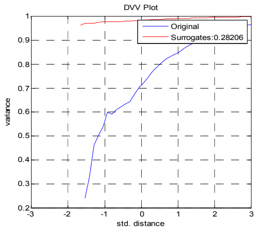

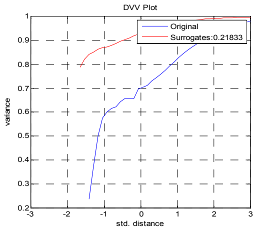

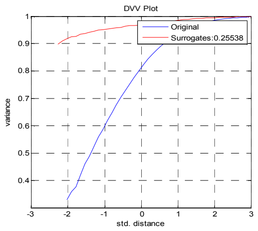

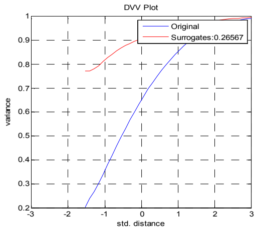

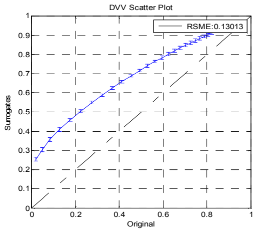

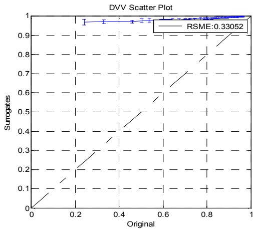

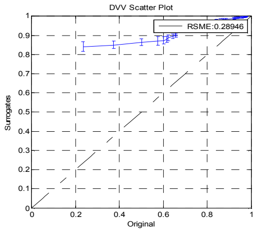

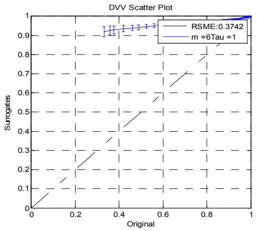

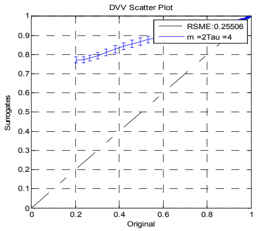

CH1 **CH2** **CH3** **LDVg** **LDV1**

EXP 19 METHOD 2

CH1

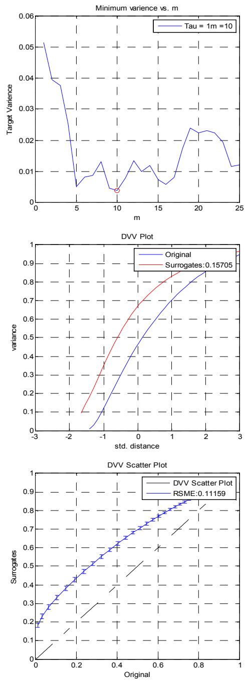

CH2

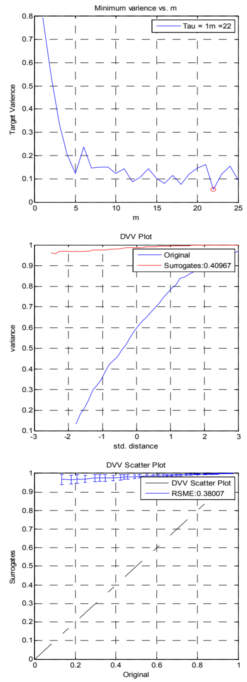

CH3

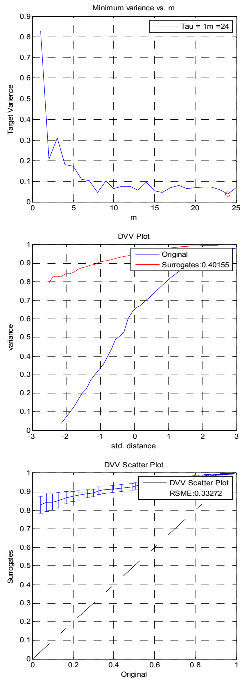

LDVg

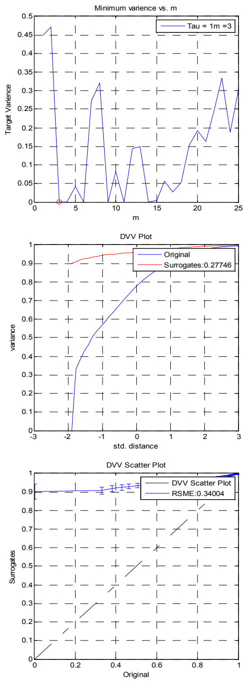

LDV1

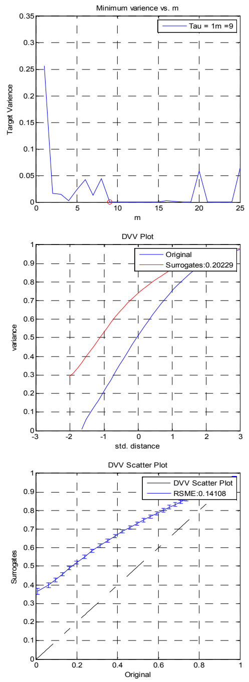

METHOD 3

CH1

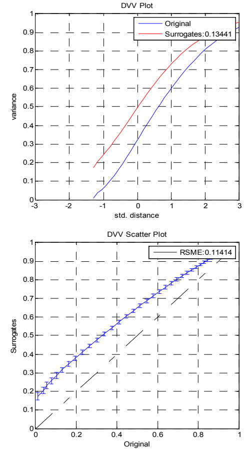

CH2

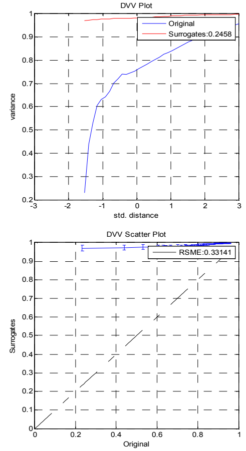

CH3

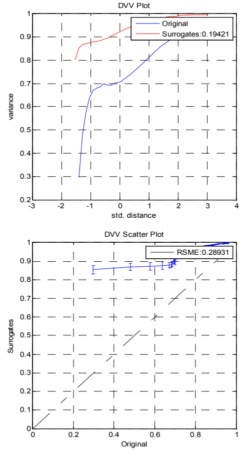

LDVg

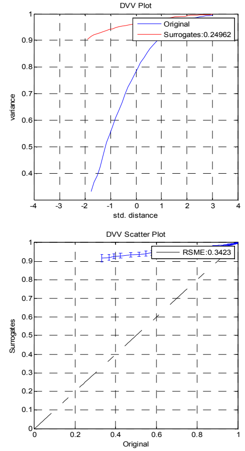

LDV1

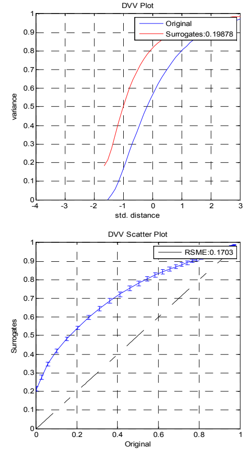

EXPERIMENT	SYSTEM CHARACTERISTCS	VARIABLES		METHOD 1				METHOD 2				METHOD 3			
				best **m**	best **τ**	rsme	RSME	calc **m**	**τ**	rsme	RSME	set **m**	set **τ**	rsme	RSME
20	SDOF CAR attached to fixed supports by 4 calibrated springs (2 on each side)	surface wood<br>Middle spring taken out<br>number of springs 2 x2;<br>loading Sine Sweep	CH1	5	10	0.2834	0.1563	10	1	0.1620	0.1213	3	1	0.1279	0.1515
			CH2	10	1	0.4544	0.3182	12	1	0.4903	0.3278	3	1	0.3329	0.2866
			CH3	4	1	0.3403	0.2314	25	1	0.5086	0.2976	3	1	0.2175	0.2418
			LDVg	9	3	0.1069	0.1251	17	1	0.0593	0.1081	3	1	0.0348	0.1059
			LDV1	8	5	0.1804	0.1634	14	1	0.2493	0.1649	3	1	0.1649	0.1388

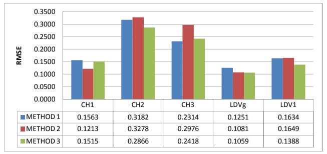

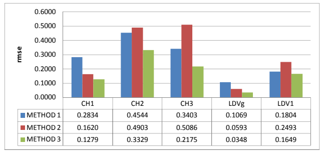

Data recorded 3D Accelerometer

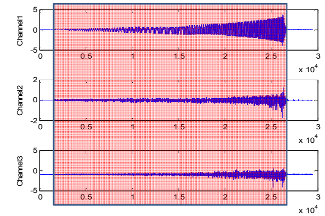

Data analysed 3D Accelerometer

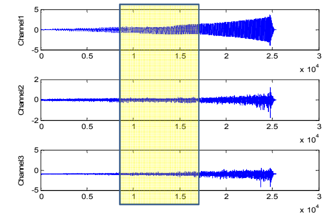

Data recorded LDV

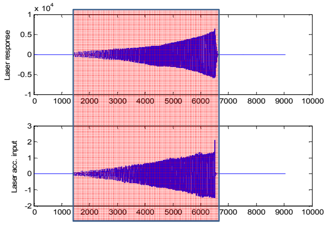

Data analysed LDV

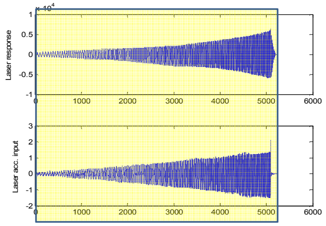

CH1 **CH2** **CH3** **LDVg** **LDV1**

METHOD 1

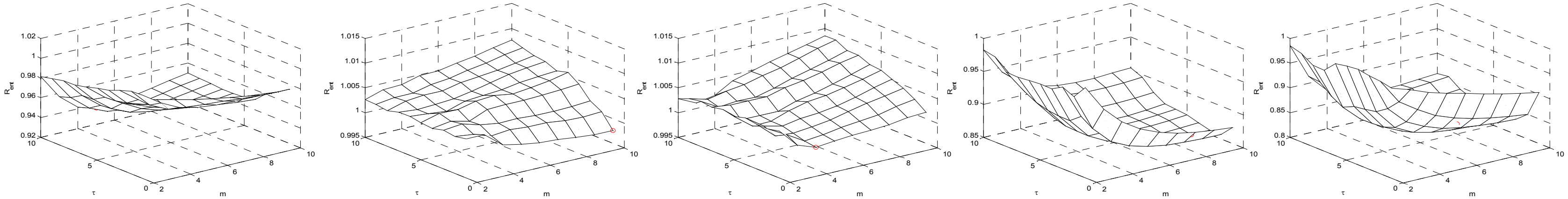

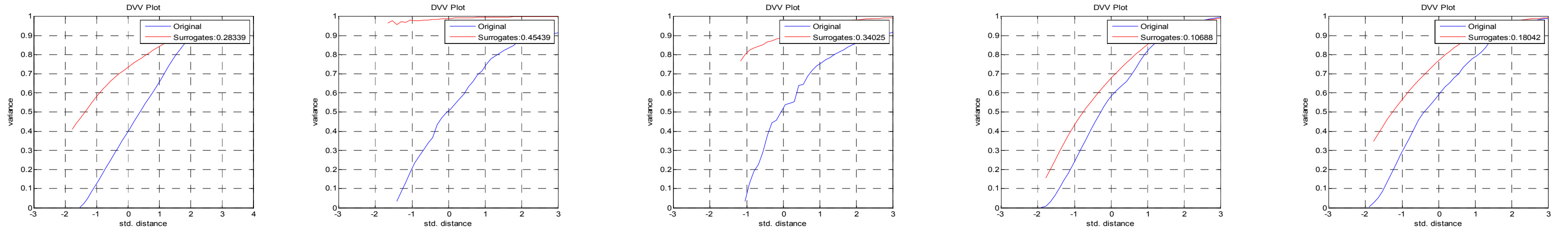

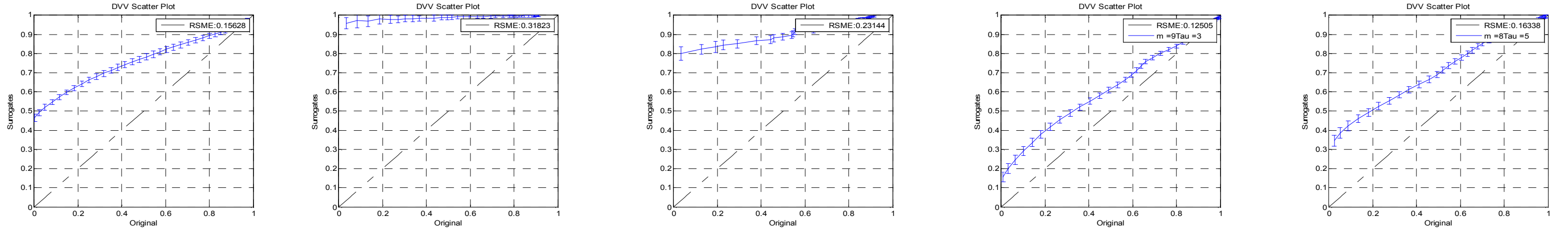

CH1 **CH2** **CH3** **LDVg** **LDV1**

CH1 | CH2 | CH3 | LDVg | LDV1

EXP 20 METHOD 2

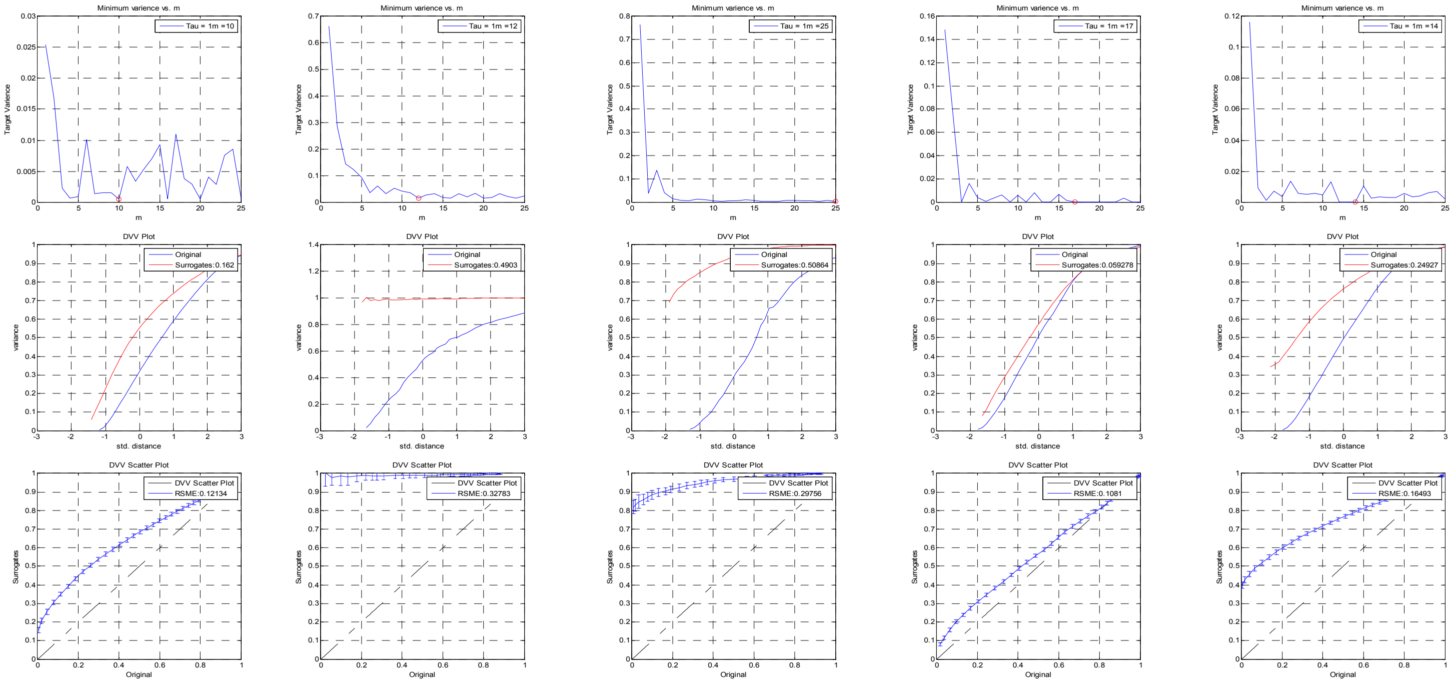

CH1 | CH2 | CH3 | LDVg | LDV1

METHOD 3

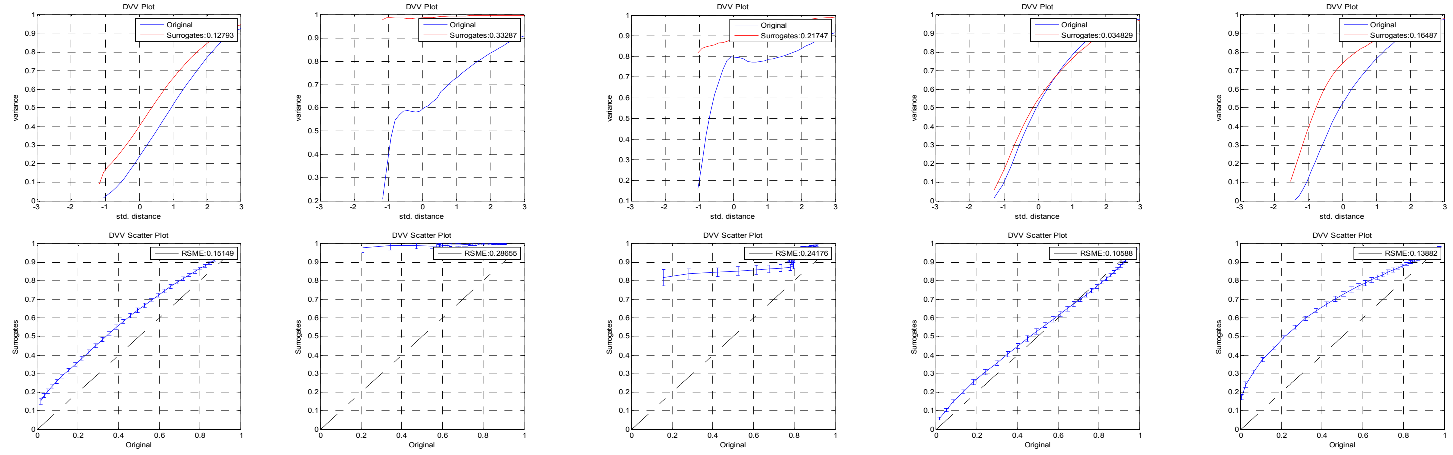

EXPERIMENT	SYSTEM CHARACTERISTCS	VARIABLES		METHOD 1				METHOD 2				METHOD 3			
				best m	best τ	rsme	RSME	calc m	τ	rsme	RSME	set m	set τ	rsme	RSME
21	SDOF CAR attached to fixed supports by 6 / 4 calibrated springs; Two middle springs are glued, detached at 13 sec / 38sec	surface wood Two middle springs are glued, detached at 13 sec / 38sec number of springs 2x3 / 2 x2; loading White Noise	CH1	6	1	0.1562	0.1031	6	1	0.1554	0.1043	3	1	0.1335	0.1079
			CH2	4	4	0.2074	0.3631	19	1	0.3613	0.3404	3	1	0.1947	0.3281
			CH3	3	1	0.1947	0.3281	14	1	0.3901	0.3646	3	1	0.2148	0.3271
			LDVg	6	1	0.1677	0.3935	10	1	0.0412	0.3492	3	1	0.0193	0.2759
			LDV1	2	8	0.0519	0.2460	20	1	0.0448	0.1007	3	1	0.0050	0.1372

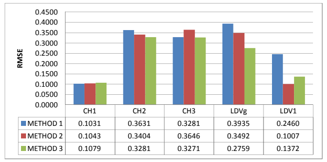

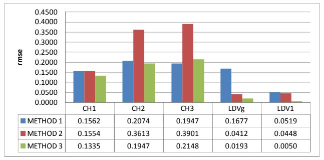

Data recorded 3D Accelerometer

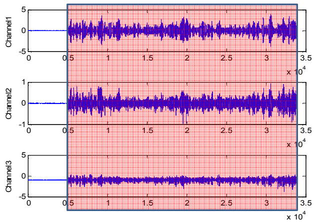

Data analysed 3D Accelerometer

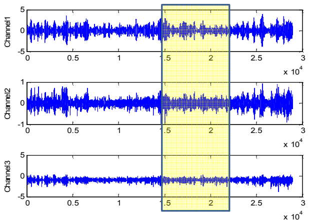

Data recorded LDV

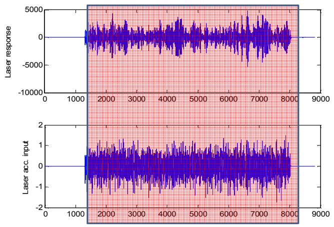

Data analysed LDV

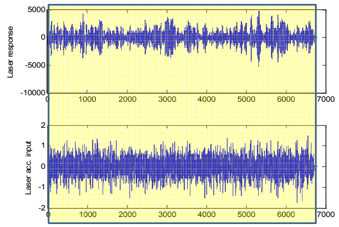

CH1 **CH2** **CH3** **LDVg** **LDV1**

METHOD 1

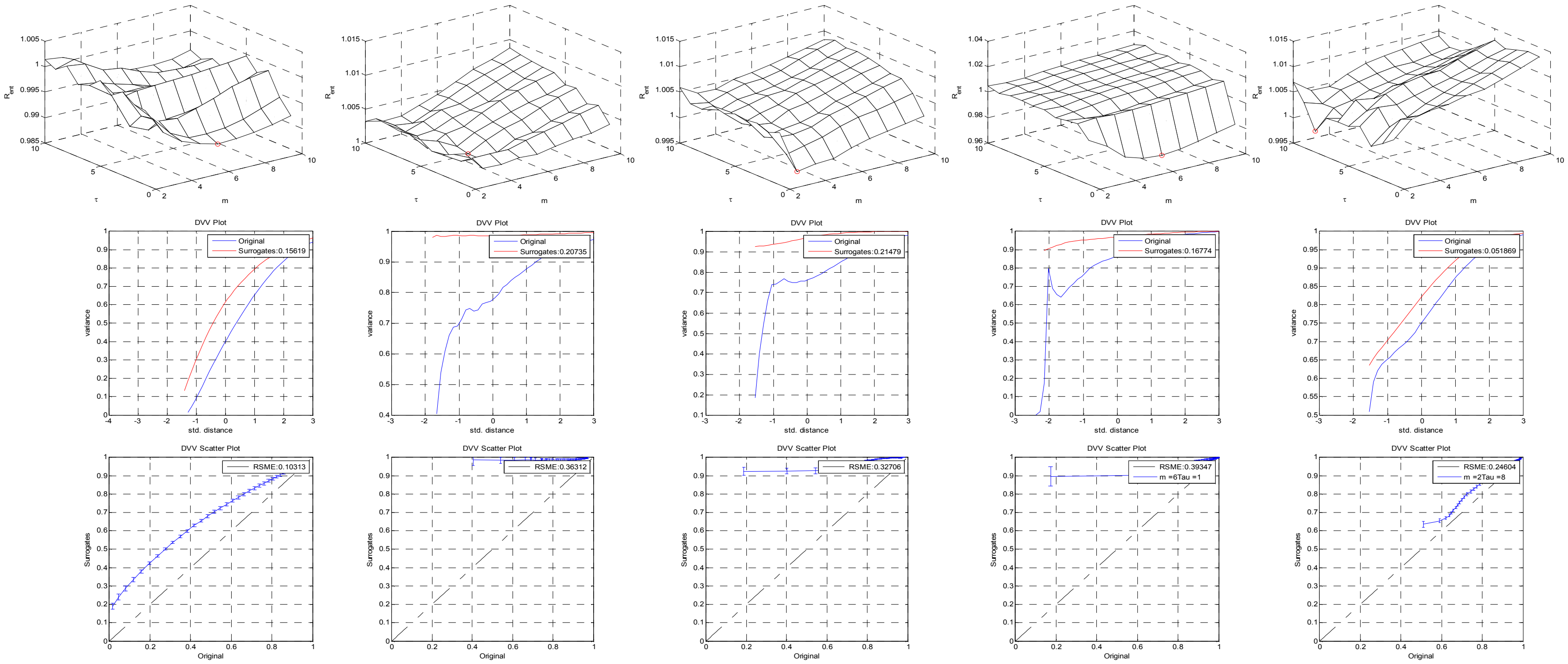

CH1 **CH2** **CH3** **LDVg** **LDV1**

CH1 **CH2** **CH3** **LDVg** **LDV1**

EXP 21 **METHOD 2**

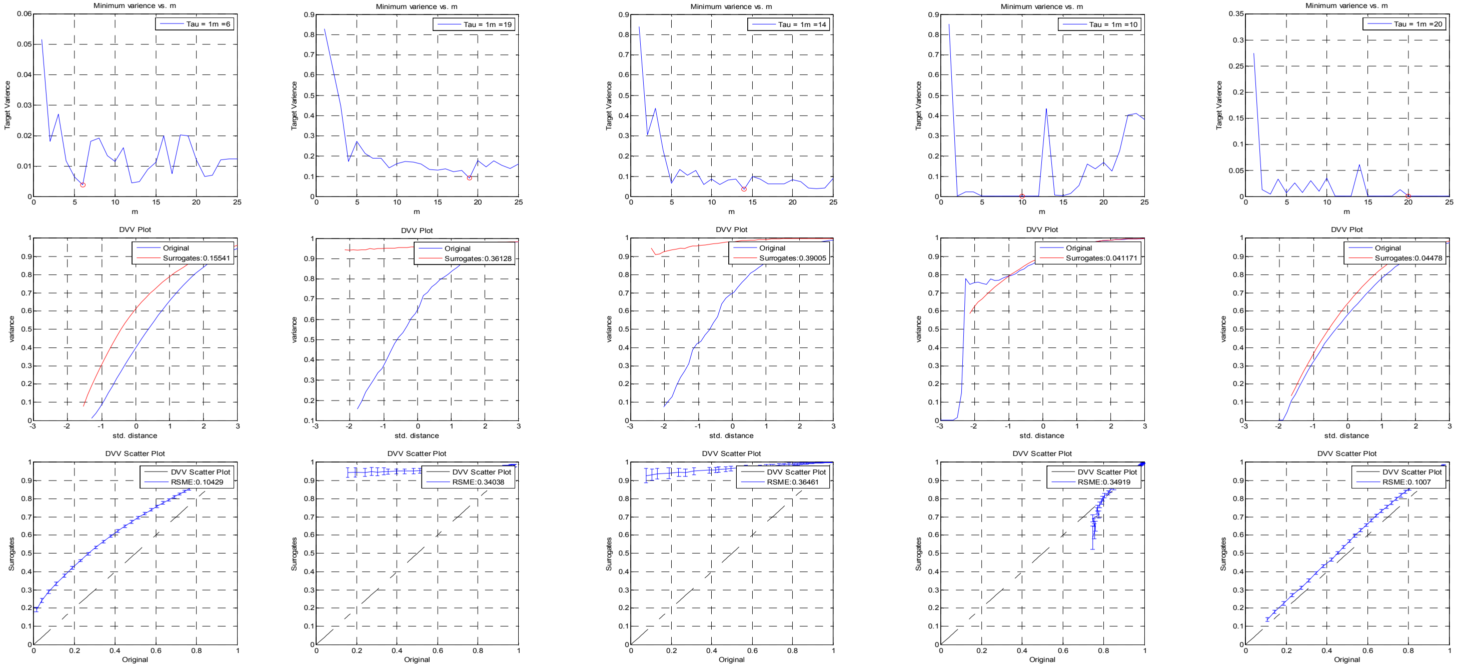

CH1 **METHOD 3** **CH2** **CH3** **LDVg** **LDV1**

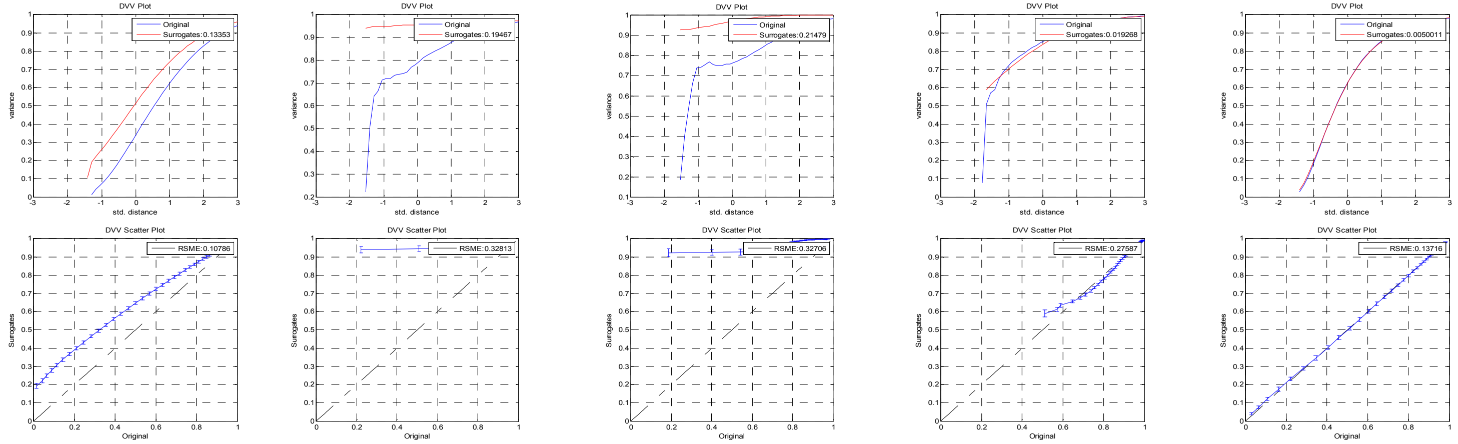

EXPERIMENT	SYSTEM CHARACTERISTCS	VARIABLES		METHOD 1				METHOD 2				METHOD 3			
				best m	best τ	rsme	RSME	calc m	τ	rsme	RSME	set m	set τ	rsme	RSME
HF	SDOF CAR attached to fixed supports by 4 calibrated springs	surface plastic (smooth) number of springs 2 x 3; loading High Frequency	CH1	2	8	0.1395	0.3746	24	1	0.3550	0.3235	3	1	0.2382	0.3390
			CH2	2	4	0.0564	0.4120	22	1	0.4344	0.3732	3	1	0.1624	0.3904
			CH3	2	2	0.0888	0.3616	20	1	0.4163	0.3340	3	1	0.1799	0.3568
			LDVg	5	1	0.3344	0.3091	22	1	0.4547	0.3248	3	1	0.2707	0.2848
			LDV1	2	2	0.0344	0.1209	13	1	0.2846	0.2393	3	1	0.2800	0.2289

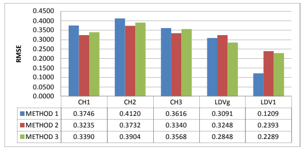

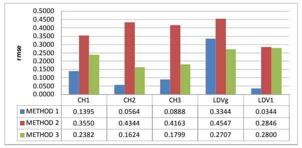

Data recorded 3D Accelerometer

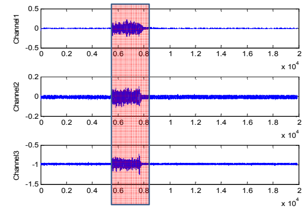

Data analysed 3D Accelerometer

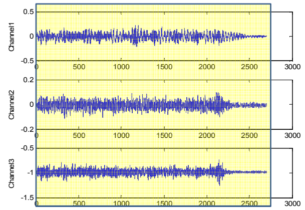

Data recorded LDV

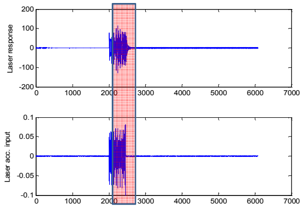

Data analysed LDV

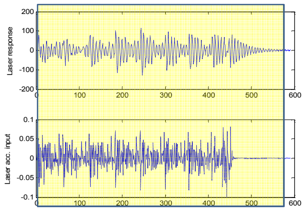

CH1 CH2 CH3 LDVg LDV1

METHOD 1

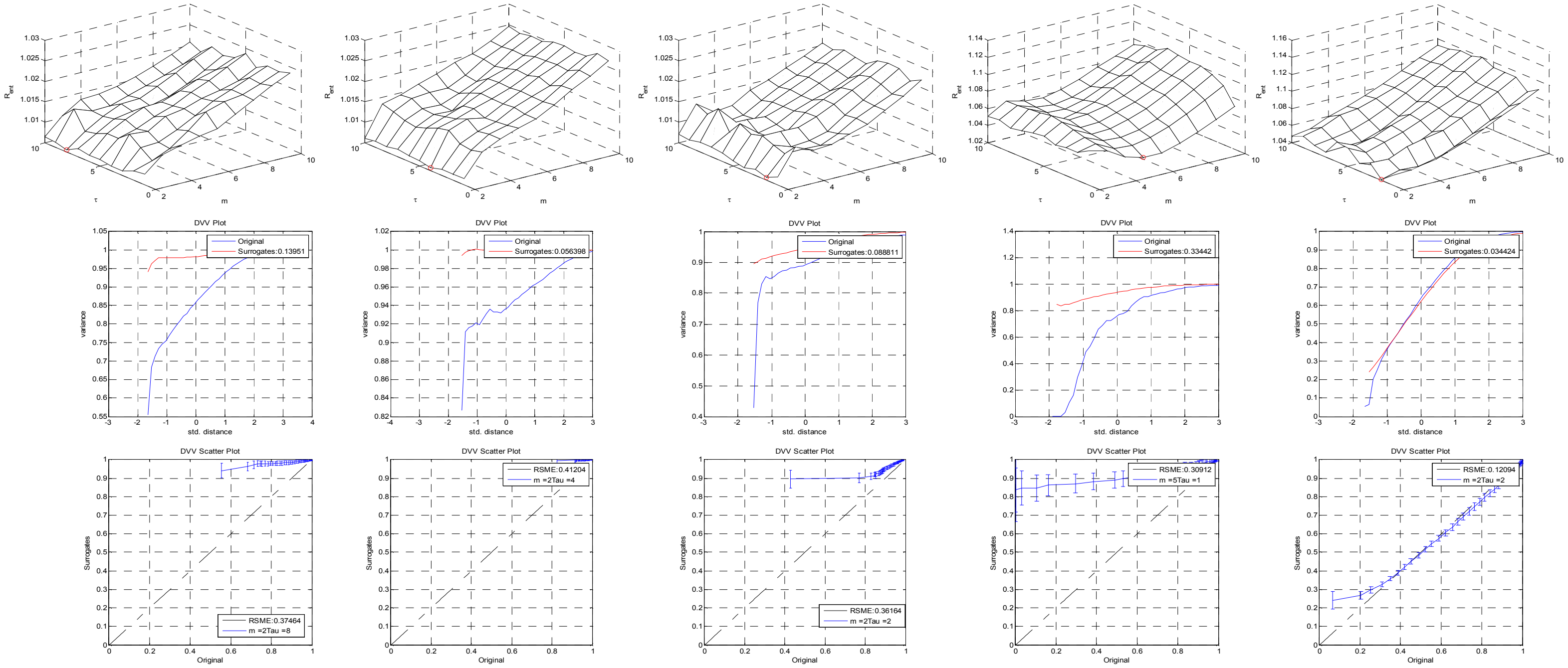

CH1 CH2 CH3 LDVg LDV1

CH1 CH2 CH3 LDVg LDV1

EXP HF METHOD 2

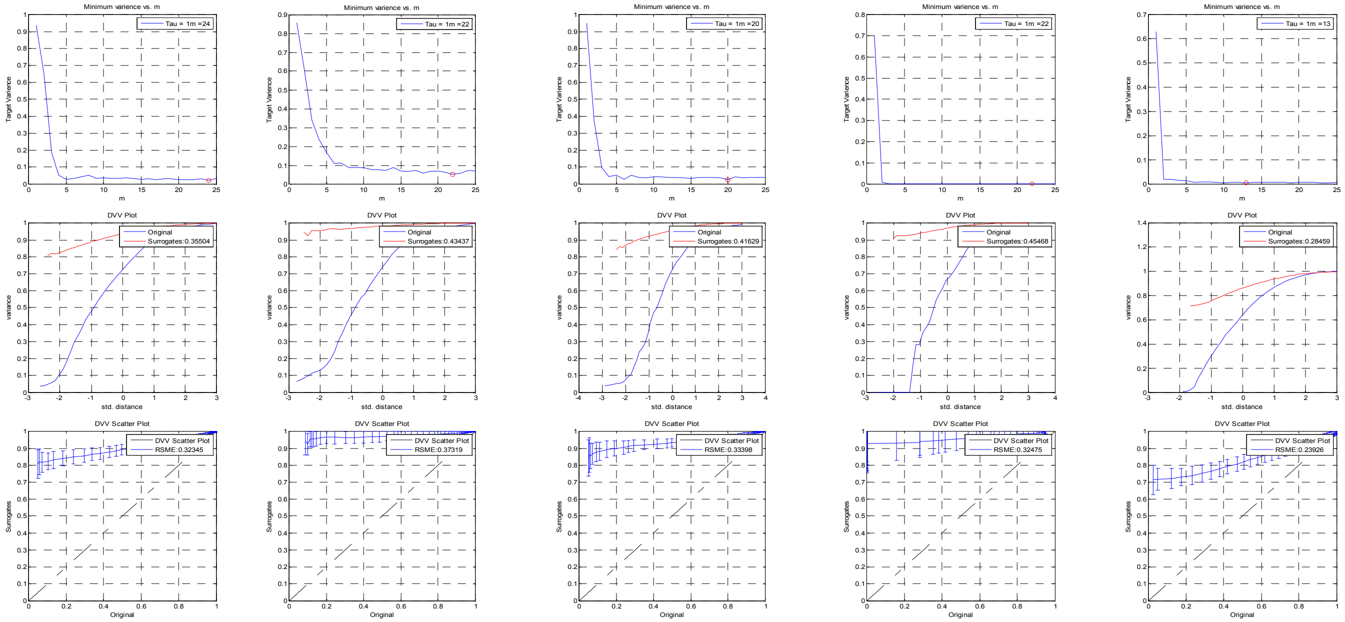

CH1 CH2 CH3 LDVg LDV1

METHOD 3

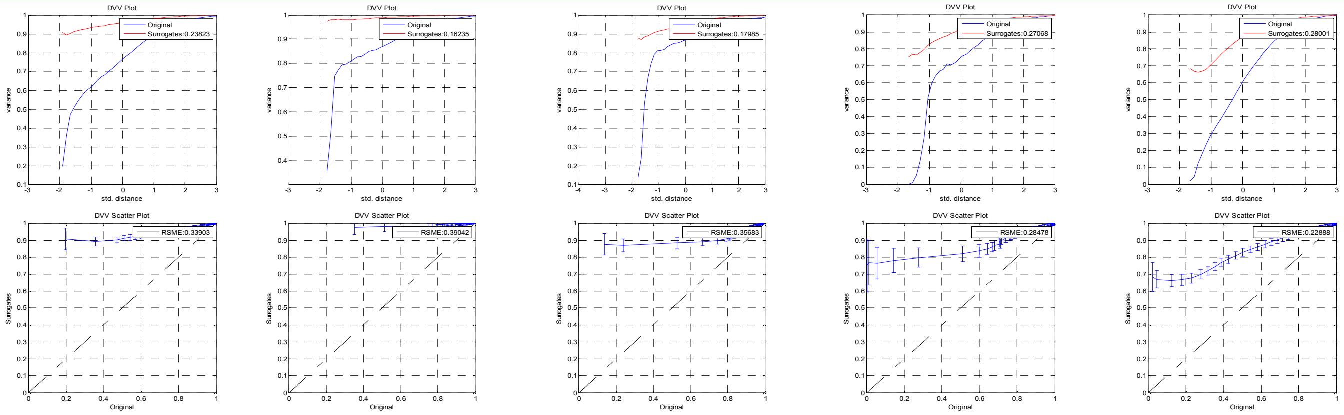

Supplement: APPENDIX 4 [file rsos150493supp4.pdf]
