# Supplementary material for: A comprehensive study of the delay vector variance method for quantification of nonlinearity in dynamical systems
Source: R Soc Open Sci. 2016 Jan 6;3(1):150493. doi: 10.1098/rsos.150493 (PMC4736930; doi:10.1098/rsos.150493)
Supplement: APPENDIX 5 [file rsos150493supp5.pdf]

APPENDIX 5

Delay Vector Variance Method WTB Experiment Results

	SYSTEM LOADING	VARIABLES	METHOD 1				METHOD 2				METHOD 3			
			best m	best τ	rsme	RSME	calc m	τ	rsme	RSME	calc m	τ	rsme	RSME
1	Initial exp<br>4.38 Hz<br>Angle of incidence 8 degree	CH1	6	1	0.7205	0.3879	22	1	0.4213	0.3237	3	1	0.4583	0.2916
		CH2	7	9	0.9878	0.5660	17	1	0.1372	0.6237	3	1	0.3264	0.5585
		CH3	6	1	0.6747	0.4278	23	1	0.6317	0.4002	3	1	0.5505	0.4688
		LDVg	5	5	0.0953	0.3211	5	1	0.5040	0.3054	3	1	0.5773	0.3781
		LDV1	5	1	0.0438	0.3418	24	1	0.0972	0.3084	3	1	0.0412	0.3481
		LDV2	4	2	0.3101	0.2569	6	1	0.3365	0.2308	3	1	0.3086	0.1947
		Strain 1	9	8	0.6131	0.3351	14	1	0.2633	0.2194	3	1	0.3456	0.1875
		Strain 2	8	8	0.5617	0.3094	16	1	0.1099	0.1336	3	1	0.1305	0.1011
		Strain 3	7	7	0.3638	0.2181	17	1	0.0544	0.0697	3	1	0.0233	0.0422
		Strain 4	6	7	0.3284	0.1853	19	1	0.0703	0.0856	3	1	0.0382	0.0543
2	Initial exp<br>Angle of incidence 8 degree	CH1	5	1	0.6093	0.3173	5	1	0.3588	0.2668	3	1	0.4816	0.3171
		CH2	5	8	0.9668	0.5607	23	1	0.3603	0.5924	3	1	0.2699	0.2972
		CH3	5	1	0.8097	0.4405	9	1	0.9294	0.5168	3	1	0.8021	0.4437
		LDVg	4	9	0.9328	0.5527	14	1	0.8386	0.5160	3	1	0.4085	0.3380
		LDV1	6	3	0.0671	0.1716	8	1	0.0213	0.1324	3	1	0.0119	0.1794
		LDV2	5	3	0.1485	0.1734	14	1	0.4531	0.2417	3	1	0.3783	0.1961
		Strain 1	9	8	0.6019	0.3269	19	1	0.2566	0.2387	3	1	0.2918	0.1683
		Strain 2	8	7	0.5645	0.3068	19	1	0.1864	0.1825	3	1	0.0941	0.1157
		Strain 3	8	7	0.4423	0.2436	17	1	0.1131	0.1043	3	1	0.0545	0.0430
		Strain 4	7	7	0.3938	0.2139	19	1	0.1028	0.1161	3	1	0.0666	0.0737
3	FOCUS AT THE ACCELEROMETER	CH1	5	1	0.4504	0.3275	22	1	0.5728	0.3890	3	1	0.3546	0.2426
		CH2	6	1	0.5751	0.4730	12	1	0.4747	0.3636	3	1	0.6560	0.4446
		CH3	5	1	0.5871	0.4145	17	1	0.8665	0.4817	3	1	0.8260	0.4483
		LDVg	4	4	0.3597	0.2869	9	1	0.2614	0.2851	3	1	0.1330	0.4179
		LDV1	6	1	0.1596	0.5475	18	1	0.1519	0.5148	3	1	0.0494	0.5983
		LDV2	5	3	0.4586	0.2360	15	1	0.4340	0.2332	3	1	0.3864	0.2038
		Strain 1	9	1	0.5881	0.3173	12	1	0.2616	0.2070	3	1	0.3687	0.2082
		Strain 2	9	3	0.6479	0.3525	14	1	0.1868	0.1676	3	1	0.1133	0.1077
		Strain 3	9	8	0.5000	0.3055	16	1	0.0878	0.0847	3	1	0.0595	0.0413
		Strain 5	7	10	0.3831	0.2087	23	1	0.1138	0.1097	3	1	0.0591	0.0695
4	with knocks, no movement, best result	CH1	6	2	0.0636	0.1588	24	1	0.0935	0.2328	3	1	0.0541	0.2235
		CH2	6	2	0.2161	0.3215	17	1	0.1887	0.5295	3	1	0.1357	0.4681
		CH3	9	10	0.5314	0.3025	12	1	0.2107	0.1925	3	1	0.3073	0.2552
		LDVg	4	9	0.8576	0.5388	24	1	0.7246	0.5184	3	1	0.1639	0.3749
		LDV1	6	6	0.1467	0.1748	4	1	0.0136	0.1512	3	1	0.0432	0.1841
		LDV2	6	10	0.4526	0.2310	4	1	0.2622	0.1386	3	1	0.2510	0.1401
		Strain 1	7	7	0.2510	0.1820	19	1	0.2288	0.1852	3	1	0.2072	0.1376
		Strain 2	7	7	0.1704	0.1744	22	1	0.1201	0.1642	3	1	0.0469	0.1272
		Strain 3	7	7	0.3900	0.1977	24	1	0.2027	0.1712	3	1	0.1166	0.1391
		Strain 4	6	7	0.3486	0.1778	24	1	0.1359	0.1640	3	1	0.0758	0.1462
5	Harmonic resonance<br>4.4Hz<br>Focus at accelerometer	CH1	6	10	0.0572	0.1473	13	1	0.0206	0.0943	3	1	0.0154	0.0872
		CH2	6	9	0.2603	0.2511	18	1	0.0178	0.1082	3	1	0.0139	0.1140
		CH3	3	9	0.0311	0.2153	7	1	0.0025	0.1630	3	1	0.0027	0.1741
		LDV	6	9	0.1335	0.2412	15	1	0.0863	0.1701	3	1	0.0595	0.1685
		LDV1	4	1	0.0070	0.1382	4	1	0.0090	0.1386	3	1	0.0031	0.1345
		LDV2	10	10	0.1712	0.1689	15	1	0.1024	0.1561	3	1	0.1424	0.1490
		Strain 1	10	9	0.1363	0.1717	4	1	0.1029	0.1583	3	1	0.1087	0.1381
		Strain 2	10	9	0.1358	0.1728	14	1	0.1973	0.2004	3	1	0.1003	0.1408
		Strain 3	10	9	0.1342	0.1726	4	1	0.1294	0.1608	3	1	0.1163	0.1417
		Strain 4	10	9	0.1340	0.1733	15	1	0.2504	0.2158	3	1	0.1123	0.1423
6	Harmonic resonance<br>2.0 Hz 2.5 Hz<br>3.0 Hz 3.5 Hz<br>4.0 Hz 4.2 Hz<br>4.3 Hz 4.4 Hz<br>4.5 Hz 4.6 Hz<br>5.0Hz 5.5 Hz<br>6.0 Hz 6.5Hz<br>7.0Hz<br>Focus at accelerometer	CH1	7	10	0.2275	0.1270	5	1	0.0798	0.1487	3	1	0.0232	0.1595
		CH2	6	10	0.0394	0.1630	25	1	0.0428	0.1091	3	1	0.0170	0.0370
		CH3	5	10	0.0115	0.1307	13	1	0.0120	0.1562	3	1	0.0093	0.1984
		LDVg	9	9	0.2784	0.2324	23	1	0.1238	0.0992	3	1	0.0457	0.0861
		LDV1	6	10	0.1399	0.1412	8	1	0.0647	0.1352	3	1	0.0227	0.1566
		LDV2	10	10	0.3261	0.1723	4	1	0.2445	0.1365	3	1	0.2419	0.1441
		Strain 1	10	9	0.4033	0.2160	8	1	0.2391	0.1495	3	1	0.2333	0.1283
		Strain 2	9	7	0.2962	0.1651	21	1	0.2939	0.1699	3	1	0.2085	0.1367
		Strain 3	8	7	0.3948	0.2094	20	1	0.4380	0.2272	3	1	0.2630	0.1443
		Strain 4	8	7	0.3774	0.1991	5	1	0.2741	0.1440	3	1	0.2032	0.1226

7	Loading Sine sweep<br>2.0 Hz 6.0 Hz<br>60 sec<br>Focus at accelerometer	CH1	4	10	0.0464	0.2276	11	1	0.0367	0.2039	3	1	0.0600	0.3182
		CH2	2	6	0.0578	0.2472	19	1	0.0725	0.1439	3	1	0.0688	0.1016
		CH3	4	9	0.0413	0.1323	25	1	0.0384	0.1946	3	1	0.0041	0.2724
		LDVg	10	2	0.3604	0.2572	12	1	0.1016	0.1091	3	1	0.0431	0.1088
		LDV1	6	6	0.3849	0.2246	10	1	0.0405	0.1303	3	1	0.0128	0.1705
		LDV2	8	10	0.0475	0.1453	12	1	0.3114	0.1656	3	1	0.2466	0.1656
		Strain 1	10	10	0.1344	0.1098	7	1	0.2157	0.1395	3	1	0.2384	0.1329
		Strain 2	10	9	0.1039	0.1309	18	1	0.2502	0.1598	3	1	0.1746	0.1363
		Strain 3	10	9	0.3815	0.2012	19	1	0.3918	0.2048	3	1	0.2432	0.1374
		Strain 4	10	9	0.3811	0.1996	23	1	0.3339	0.1717	3	1	0.2277	0.1420
8	Loading White Noise<br>Focus at accelerometer	CH1	4	1	0.0036	0.0732	10	1	0.0160	0.1327	3	1	0.0104	0.0686
		CH2	5	1	0.0435	0.1647	19	1	0.0804	0.1612	3	1	0.0347	0.1264
		CH3	3	1	0.0224	0.0949	16	1	0.0383	0.1911	3	1	0.0168	0.0917
		LDVg	4	1	0.2189	0.3606	19	1	0.0113	0.3882	3	1	0.0444	0.2542
		LDV1	6	10	0.1990	0.1854	12	1	0.0274	0.1337	3	1	0.0057	0.1268
		LDV2	9	10	0.2077	0.2278	14	1	0.3092	0.2399	3	1	0.2351	0.2062
		Strain 1	7	7	0.2163	0.3643	22	1	0.3935	0.3613	3	1	0.2497	0.3134
		Strain 2	7	7	0.1296	0.2625	24	1	0.2236	0.2427	3	1	0.0852	0.2064
		Strain 3	10	6	0.3315	0.2834	17	1	0.1921	0.1646	3	1	0.1455	0.1260
		Strain 4	10	10	0.3392	0.2944	16	1	0.0946	0.1124	3	1	0.0854	0.0854
9	Loading White noise<br>4.365Hz at the peak<br>Focus at top strain gauge	CH1	2	7	0.0203	0.1421	7	1	0.0114	0.1050	3	1	0.0121	0.0704
		CH2	5	1	0.0394	0.1675	18	1	0.0761	0.1552	3	1	0.0297	0.1257
		CH3	5	1	0.0123	0.1200	25	1	0.0501	0.2024	3	1	0.0222	0.0947
		LDVg	5	1	0.2075	0.3547	15	1	0.0690	0.3539	3	1	0.0522	0.2421
		LDV1	3	9	0.0339	0.1178	15	1	0.0119	0.1097	3	1	0.0071	0.0961
		LDV2	2	1	0.0059	0.1578	17	1	0.2185	0.2670	3	1	0.1820	0.2898
		Strain 1	3	3	0.0082	0.3065	6	1	0.2182	0.3492	3	1	0.2197	0.3364
		Strain 2	2	9	0.0052	0.3211	7	1	0.0842	0.2754	3	1	0.0730	0.2318
		Strain 3	10	6	0.2223	0.2959	23	1	0.1423	0.1939	3	1	0.1275	0.1328
		Strain 4	10	10	0.0073	0.1965	7	1	0.0813	0.1675	3	1	0.0804	0.0913
10	Loading Sine Sweep<br>2.0 Hz 6.0 Hz<br>60 sec<br>Focus at top strain gauge	CH1	7	10	0.0696	0.1634	19	1	0.0964	0.2472	3	1	0.0406	0.2608
		CH2	2	8	0.0451	0.2468	20	1	0.0536	0.1117	3	1	0.0443	0.0576
		CH3	5	10	0.0142	0.1677	3	1	0.0102	0.2814	3	1	0.0688	0.3002
		LDVg	10	2	0.3382	0.2526	4	1	0.0559	0.1080	3	1	0.0501	0.0948
		LDV1	6	9	0.3271	0.1985	22	1	0.0585	0.1792	3	1	0.0137	0.1854
		LDV2	8	10	0.1156	0.1703	15	1	0.3039	0.1775	3	1	0.2673	0.1696
		Strain 1	10	10	0.1380	0.1337	14	1	0.4121	0.2053	3	1	0.2496	0.1449
		Strain 2	10	9	0.3985	0.2059	10	1	0.2505	0.1497	3	1	0.2046	0.1533
		Strain 3	10	9	0.4153	0.2148	11	1	0.3410	0.1694	3	1	0.3243	0.1976
		Strain 4	10	9	0.3978	0.2049	12	1	0.3542	0.1764	3	1	0.2607	0.1687
11	Harmonic resonance<br>2.0 Hz 2.5 Hz<br>3.0 Hz 3.5 Hz<br>4.0 Hz 4.2 Hz<br>4.3 Hz 4.4 Hz<br>4.5 Hz 4.6 Hz<br>5.0Hz 5.5 Hz<br>6.0 Hz 6.5Hz<br>7.0Hz<br>Focus at top strain gauge	CH1	7	10	0.0832	0.1831	19	1	0.0857	0.2276	3	1	0.0090	0.2512
		CH2	6	10	0.0562	0.1197	22	1	0.0283	0.0817	3	1	0.0116	0.0566
		CH3	4	9	0.0117	0.1400	22	1	0.0071	0.1270	3	1	0.0033	0.1931
		LDVg	10	3	0.1712	0.1685	25	1	0.1249	0.1246	3	1	0.0476	0.1111
		LDV1	8	10	0.1882	0.1488	22	1	0.1733	0.1619	3	1	0.0513	0.1534
		LDV2	10	10	0.3138	0.1713	23	1	0.2482	0.1402	3	1	0.2315	0.1334
		Strain 1	10	10	0.2620	0.1491	17	1	0.4013	0.2202	3	1	0.2123	0.1152
		Strain 2	10	9	0.3953	0.2354	21	1	0.3584	0.1932	3	1	0.2224	0.1279
		Strain 3	10	9	0.3963	0.2366	5	1	0.2270	0.1398	3	1	0.2484	0.1399
		Strain 4	10	9	0.3737	0.2128	19	1	0.4437	0.2166	3	1	0.2365	0.1354
12	Harmonic resonance<br>2.0 Hz 2.5 Hz<br>3.0 Hz 3.5 Hz<br>4.0 Hz 4.2 Hz<br>4.3 Hz 4.4 Hz<br>4.5 Hz 4.6 Hz<br>5.0Hz 5.5 Hz<br>6.0 Hz 6.5Hz<br>7.0Hz<br>Focus at mid strain gauge	CH1	2	1	0.1527	0.1006	4	1	0.0109	0.0823	3	1	0.0125	0.0867
		CH2	2	8	0.0106	0.3796	22	1	0.0315	0.2460	3	1	0.0153	0.1746
		CH3	4	4	0.0039	0.1078	1	1	0.0044	0.1585	3	1	0.0020	0.1523
		LDVg	10	2	0.1702	0.1444	22	1	0.1419	0.1289	3	1	0.0433	0.1084
		LDV1	7	9	0.1465	0.1440	2	1	0.0104	0.1702	3	1	0.0289	0.1622
		LDV2	10	9	0.3163	0.1872	24	1	0.2205	0.1337	3	1	0.2131	0.1233
		Strain 1	10	10	0.2410	0.1465	5	1	0.2325	0.1457	3	1	0.2172	0.1187
		Strain 2	10	10	0.2919	0.1654	23	1	0.3287	0.1857	3	1	0.1936	0.1191
		Strain 3	10	9	0.3749	0.2301	18	1	0.4264	0.2194	3	1	0.2271	0.1299
		Strain 4	10	9	0.3499	0.2086	7	1	0.2936	0.1784	3	1	0.2519	0.1433

13	Loading Sine Sweep<br>2.0 Hz 6.0 Hz<br>60 sec<br>Focus at mid strain gauge	CH1	3	9	0.0425	0.2044	21	1	0.0639	0.3005	3	1	0.0241	0.2905
		CH2	8	9	0.1169	0.2192	17	1	0.0798	0.1423	3	1	0.0533	0.0738
		CH3	3	8	0.0259	0.1349	22	1	0.0196	0.1741	3	1	0.0227	0.2591
		LDVg	10	2	0.1912	0.1799	7	1	0.1105	0.1670	3	1	0.0619	0.1380
		LDV1	6	6	0.1866	0.2810	5	1	0.0367	0.3386	3	1	0.0668	0.3511
		LDV2	7	7	0.0617	0.2329	24	1	0.5283	0.3078	3	1	0.4848	0.3156
		Strain 1	10	10	0.0882	0.1039	22	1	0.4030	0.2106	3	1	0.2604	0.1358
		Strain 2	9	9	0.0766	0.1423	7	1	0.2112	0.1369	3	1	0.2040	0.1564
		Strain 3	10	9	0.3634	0.1895	5	1	0.2536	0.1320	3	1	0.2723	0.1620
		Strain 4	9	9	0.3700	0.1903	4	1	0.2454	0.1417	3	1	0.2522	0.1643
14	Loading White noise<br>4.336Hz at the peak<br>next 23Hz<br>Focus at mid strain gauge	CH1	5	1	0.0072	0.0756	24	1	0.0156	0.0853	3	1	0.0080	0.0732
		CH2	5	1	0.0372	0.1616	14	1	0.0489	0.1552	3	1	0.0277	0.1218
		CH3	4	1	0.0190	0.1089	21	1	0.0537	0.1977	3	1	0.0153	0.0961
		LDVg	8	1	0.1692	0.3932	25	1	0.0381	0.4272	3	1	0.0524	0.2549
		LDV1	2	2	0.0030	0.2366	3	1	0.0044	0.1145	3	1	0.0019	0.1134
		LDV2	2	4	0.1031	0.3799	4	1	0.2186	0.2779	3	1	0.2233	0.2656
		Strain 1	2	5	0.0023	0.3227	18	1	0.2176	0.4070	3	1	0.2059	0.3447
		Strain 2	3	5	0.0068	0.2992	22	1	0.0866	0.2729	3	1	0.0637	0.2257
		Strain 3	2	9	0.2249	0.3144	25	1	0.1299	0.1882	3	1	0.1149	0.1277
		Strain 4	7	2	0.2378	0.2272	18	1	0.0800	0.1192	3	1	0.0721	0.0910
15	Loading White noise<br>4.336Hz at the peak<br>next 23Hz<br>Focus at bottom strain gauge	CH1	4	1	0.0062	0.0692	5	1	0.0066	0.0772	3	1	0.0038	0.0668
		CH2	5	1	0.0321	0.1750	22	1	0.0695	0.1675	3	1	0.0206	0.1353
		CH3	4	1	0.0192	0.1124	17	1	0.0467	0.1979	3	1	0.0168	0.0963
		LDVg	8	1	0.0505	0.3124	9	1	0.0654	0.3185	3	1	0.0442	0.2607
		LDV1	2	10	0.0086	0.1173	19	1	0.0120	0.0846	3	1	0.0041	0.1057
		LDV2	6	1	0.3039	0.3063	18	1	0.3192	0.3524	3	1	0.2591	0.2782
		Strain 1	8	1	0.3199	0.3504	14	1	0.3787	0.3699	3	1	0.2443	0.3280
		Strain 2	7	7	0.1514	0.3172	19	1	0.1445	0.2367	3	1	0.2132	0.2352
		Strain 3	10	6	0.1905	0.2279	18	1	0.1627	0.1652	3	1	0.2765	0.1664
		Strain 4	10	10	0.1453	0.2298	19	1	0.0940	0.1269	3	1	0.2801	0.1815
16	Loading Sine Sweep<br>2.0 Hz 6.0 Hz<br>60 sec<br>Focus at bottom strain gauge	CH1	4	10	0.0716	0.1065	25	1	0.0620	0.1974	3	1	0.0354	0.2434
		CH2	2	5	0.0396	0.2383	7	1	0.0333	0.0989	3	1	0.0144	0.0673
		CH3	3	10	0.0141	0.1101	9	1	0.0089	0.1931	3	1	0.0087	0.2193
		LDVg	10	2	0.1700	0.1688	21	1	0.1494	0.1387	3	1	0.0528	0.1211
		LDV1	6	6	0.1758	0.1823	11	1	0.0872	0.1796	3	1	0.0314	0.2079
		LDV2	8	8	0.3659	0.2303	20	1	0.3000	0.1593	3	1	0.2509	0.1316
		Strain 1	10	10	0.1578	0.1270	9	1	0.2749	0.1510	3	1	0.2501	0.1336
		Strain 2	9	9	0.1196	0.1483	6	1	0.2365	0.1597	3	1	0.1830	0.1559
		Strain 3	10	10	0.4309	0.2256	10	1	0.3394	0.1691	3	1	0.2680	0.1610
		Strain 4	9	9	0.3920	0.2015	5	1	0.2818	0.1535	3	1	0.2522	0.1629
17	Harmonic resonance<br>2.0 Hz 2.5 Hz<br>3.0 Hz 3.5 Hz<br>4.0 Hz 4.2 Hz<br>4.3 Hz 4.4 Hz<br>4.5 Hz 4.6 Hz<br>5.0Hz 5.5 Hz<br>6.0 Hz 6.5Hz<br>7.0Hz<br>Focus at bottom strain gauge.	CH1	7	10	0.1119	0.1255	22	1	0.1015	0.1965	3	1	0.0530	0.2385
		CH2	6	10	0.0359	0.1084	17	1	0.0197	0.0814	3	1	0.0073	0.0491
		CH3	4	10	0.0191	0.1464	14	1	0.0181	0.1311	3	1	0.0152	0.1867
		LDVg	10	8	0.2920	0.2477	13	1	0.1010	0.0961	3	1	0.0445	0.1009
		LDV1	5	9	0.0212	0.1582	11	1	0.0163	0.1671	3	1	0.0111	0.1873
		LDV2	8	7	0.3506	0.2324	21	1	0.3050	0.1632	3	1	0.2263	0.1206
		Strain 1	10	7	0.3605	0.2185	15	1	0.3875	0.2077	3	1	0.2459	0.1285
		Strain 2	9	7	0.3372	0.1942	10	1	0.2350	0.1589	3	1	0.2011	0.1230
		Strain 3	10	7	0.4138	0.2289	10	1	0.2820	0.1681	3	1	0.2267	0.1304
		Strain 4	9	7	0.3715	0.2099	9	1	0.2784	0.1719	3	1	0.2293	0.1324

* accelerometer placed close to the top of WTB

CH1 - accelerometer channel 1 in the direction of applied vibrations

CH2 - accelerometer channel 2 perpendicular to the direction of applied vibrations

CH3 - accelerometer channel 3 perpendicular to the direction of applied vibrations

LDVg - laser dopler vibrometer (LDV) measurements of accelerometer input

LDV1 - LDV measurements (displacement)

LDV2 - LDV measurements (velocity)

Strain 1 - strain gauge 1 at 1/3 length of WTB from the top

Strain 2 - strain gauge 2 at 2/3 length of WTB from the top

Strain 3 - strain gauge 3 close to bottom of WTB

Strain 5 - strain gauge 5 opposite of 3

EXPERIMENT	VARIABLES		METHOD 1				METHOD 2				METHOD 3			
			best **m**	best **τ**	rsme	RSME	calc **m**	**τ**	rsme	RSME	set **m**	set **τ**	rsme	RSME
1	Initial exp<br>4.38 Hz<br>Angle of incidence 8 degree	CH1	6	1	0.7205	0.3879	22	1	0.4213	0.3237	3	1	0.4583	0.2916
		CH2	7	9	0.9878	0.5660	17	1	0.1372	0.6237	3	1	0.3264	0.5585
		CH3	6	1	0.6747	0.4278	23	1	0.6317	0.4002	3	1	0.5505	0.4688
		LDVg	5	5	0.0953	0.3211	5	1	0.5040	0.3054	3	1	0.5773	0.3781
		LDV1	5	1	0.0438	0.3418	24	1	0.0972	0.3084	3	1	0.0412	0.3481
		LDV2	4	2	0.3101	0.2569	6	1	0.3365	0.2308	3	1	0.3086	0.1947
		Strain 1	9	8	0.6131	0.3351	14	1	0.2633	0.2194	3	1	0.3456	0.1875
		Strain 2	8	8	0.5617	0.3094	16	1	0.1099	0.1336	3	1	0.1305	0.1011
		Strain 3	7	7	0.3638	0.2181	17	1	0.0544	0.0697	3	1	0.0233	0.0422
		Strain 4	6	7	0.3284	0.1853	19	1	0.0703	0.0856	3	1	0.0382	0.0543

Data recorded 3D Accelerometer

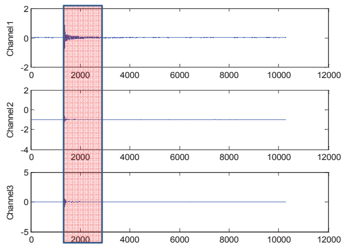

Data analysed 3D Accelerometer

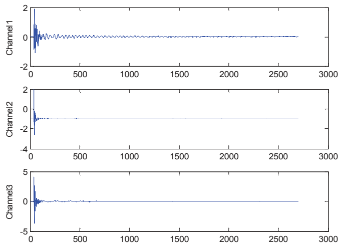

METHOD 1

CH1

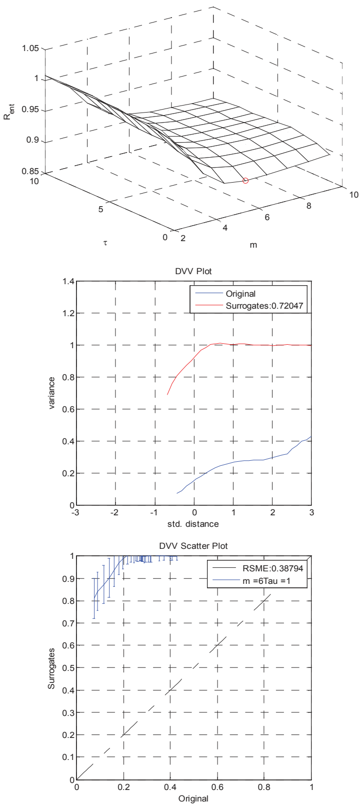

CH2

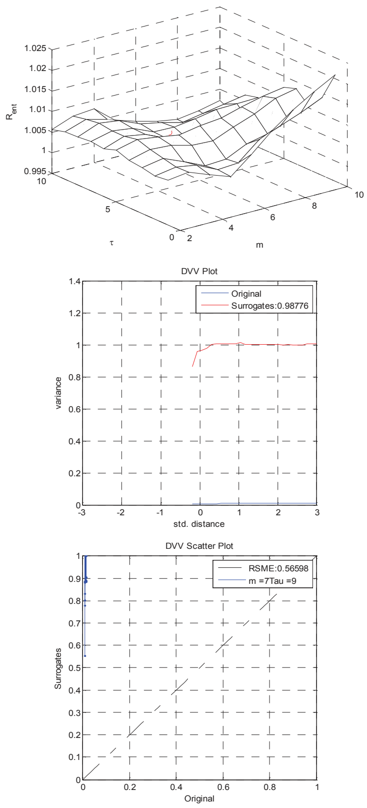

CH3

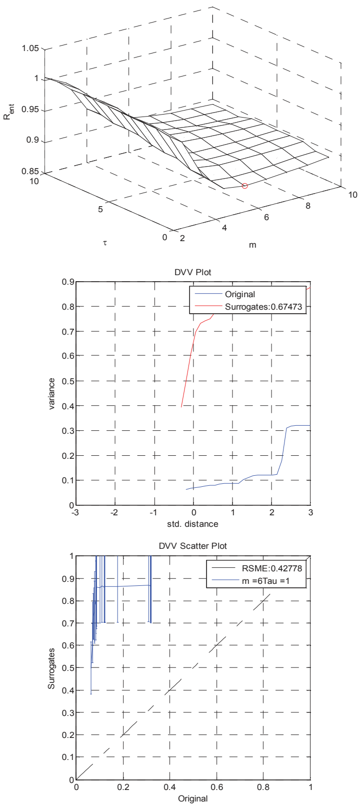

EXP 1 METHOD 2

CH1 **CH2** **CH3**

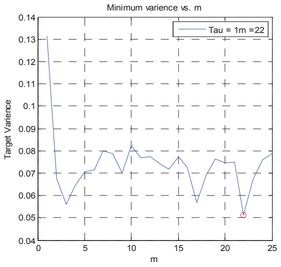

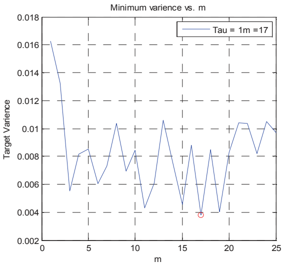

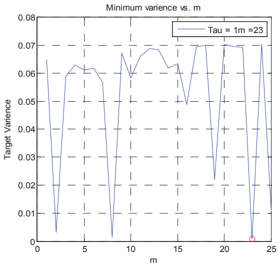

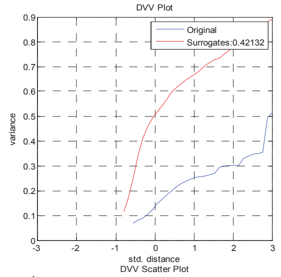

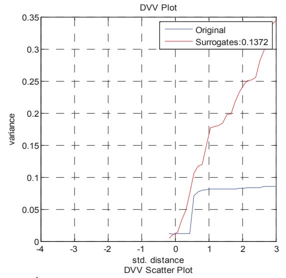

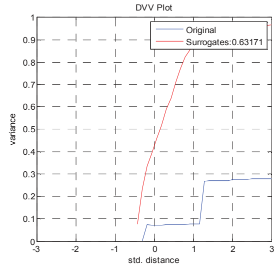

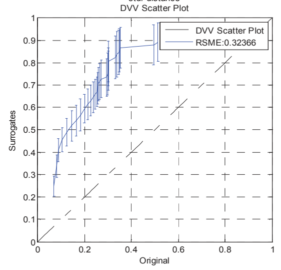

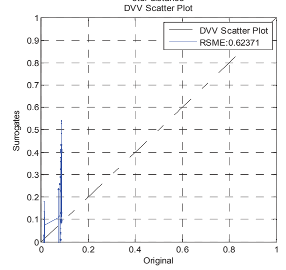

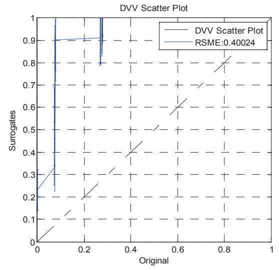

METHOD 3

CH1 **CH2** **CH3**

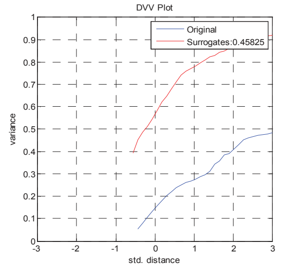

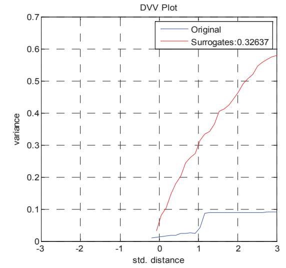

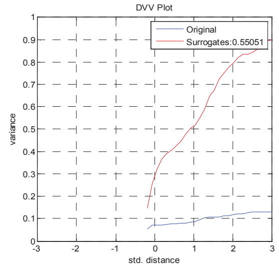

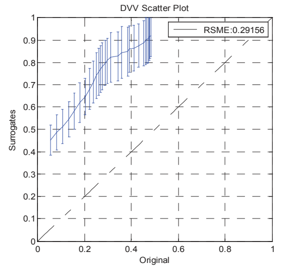

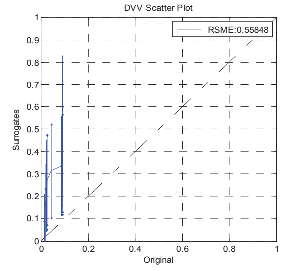

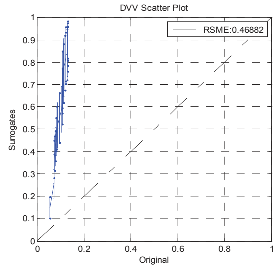

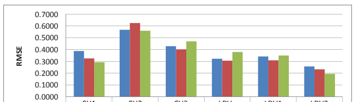

	CH1	CH2	CH3	LDVg	LDV1	LDV2
METHOD 1	0.3879	0.5660	0.4278	0.3211	0.3418	0.2569
METHOD 2	0.3237	0.6237	0.4002	0.3054	0.3084	0.2308
METHOD 3	0.2916	0.5585	0.4688	0.3781	0.3481	0.1947

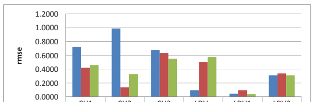

	CH1	CH2	CH3	LDVg	LDV1	LDV2
METHOD 1	0.7205	0.9878	0.6747	0.0953	0.0438	0.3101
METHOD 2	0.4213	0.1372	0.6317	0.5040	0.0972	0.3365
METHOD 3	0.4583	0.3264	0.5505	0.5773	0.0412	0.3086

Data recorded LDV

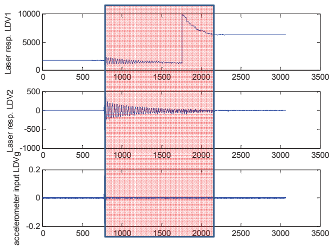

Data analysed LDV

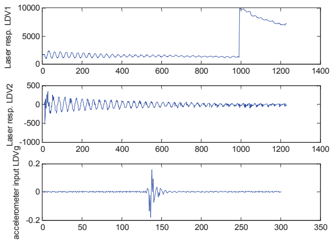

EXP 1

LDVg

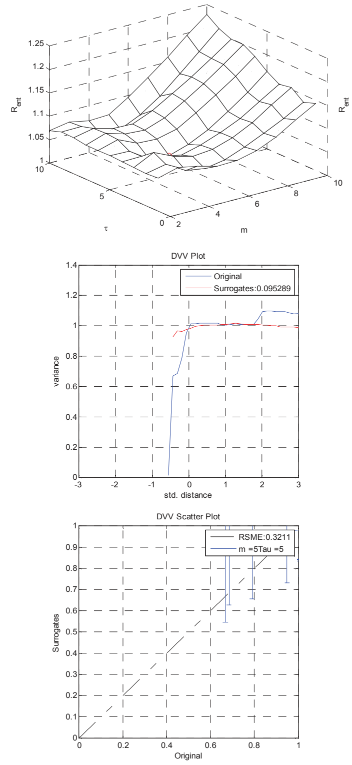

LDV1

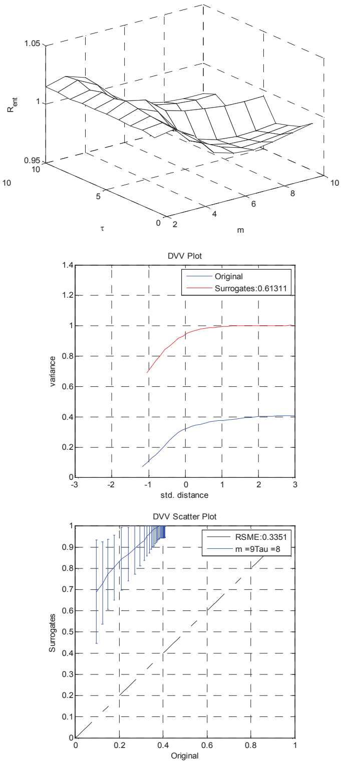

LDV2

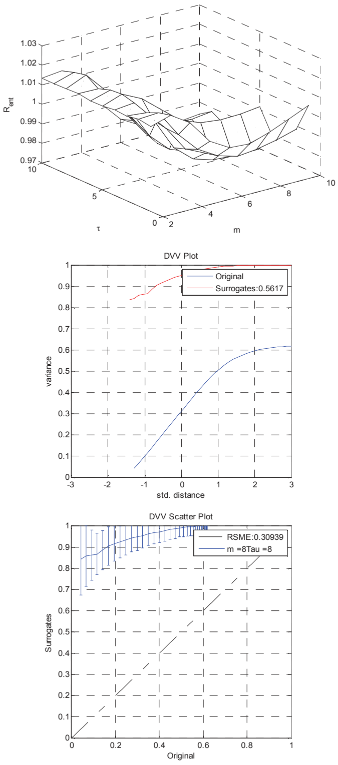

EXP 1

LDVg

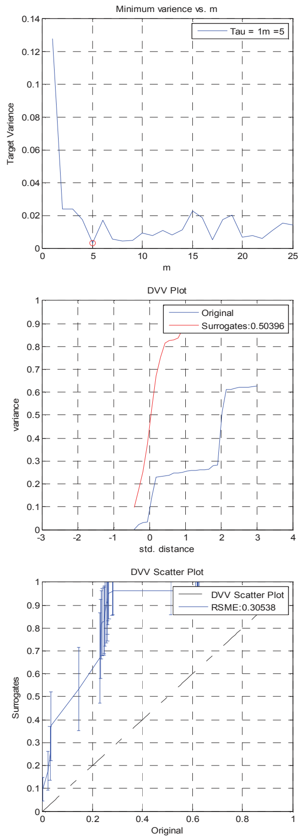

LDV1

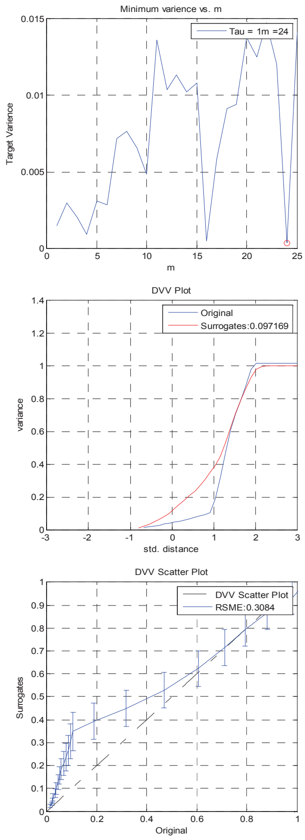

LDV2

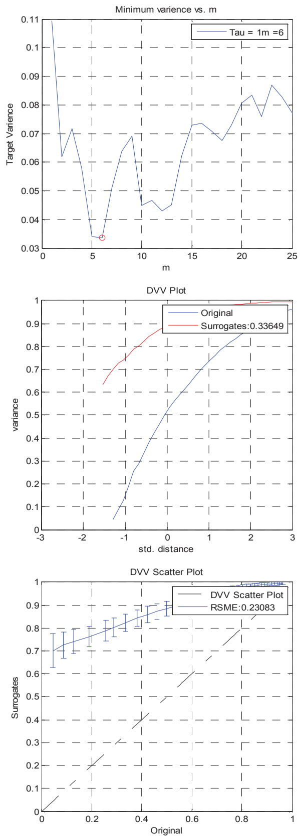

LDVg

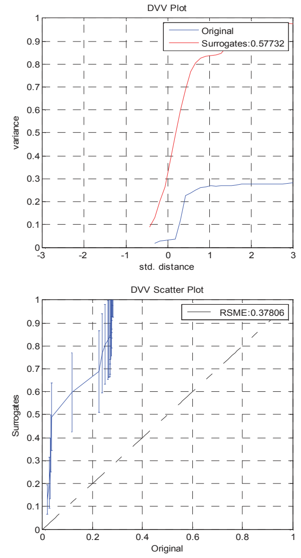

LDV1

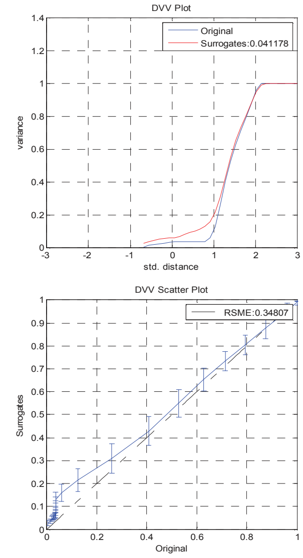

LDV2

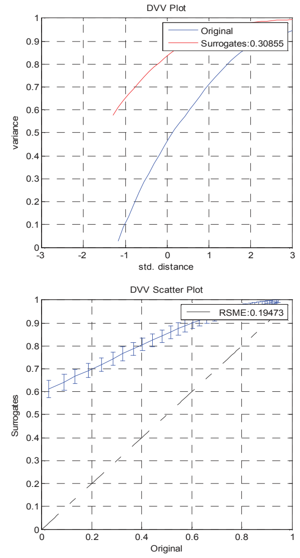

Data recorded Strain Gauge

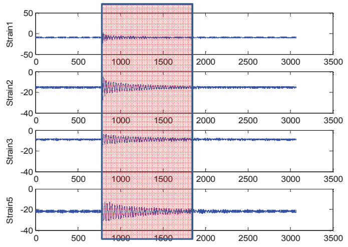

Data analysed Strain Gauge

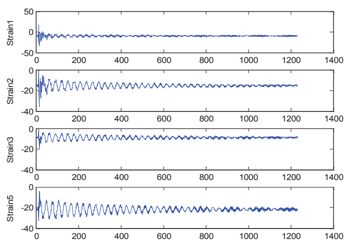

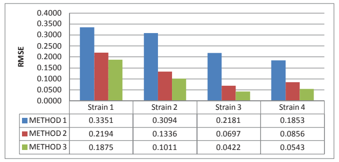

	Strain 1	Strain 2	Strain 3	Strain 4
METHOD 1	0.3351	0.3094	0.2181	0.1853
METHOD 2	0.2194	0.1336	0.0697	0.0856
METHOD 3	0.1875	0.1011	0.0422	0.0543

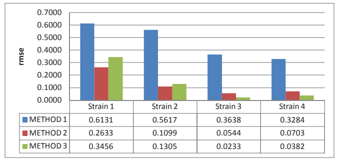

	Strain 1	Strain 2	Strain 3	Strain 4
METHOD 1	0.6131	0.5617	0.3638	0.3284
METHOD 2	0.2633	0.1099	0.0544	0.0703
METHOD 3	0.3456	0.1305	0.0233	0.0382

EXP 1

STRAIN 1

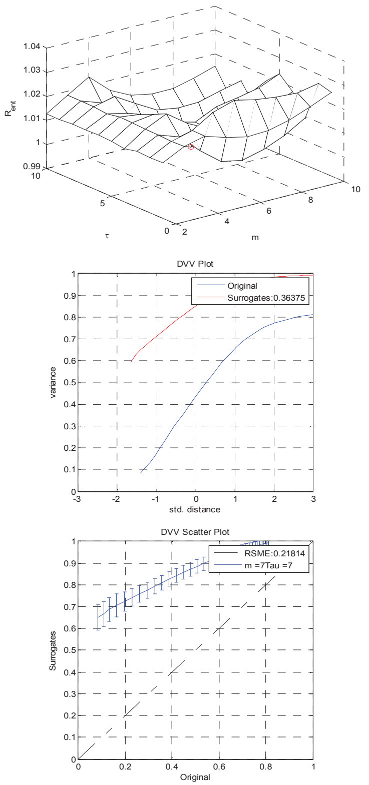

STRAIN 2

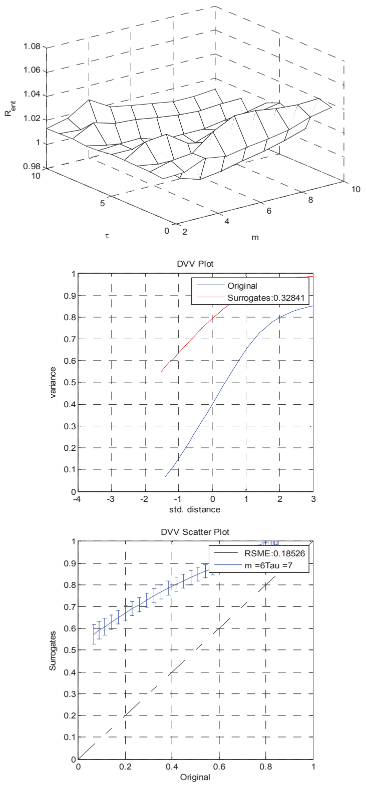

STRAIN 3

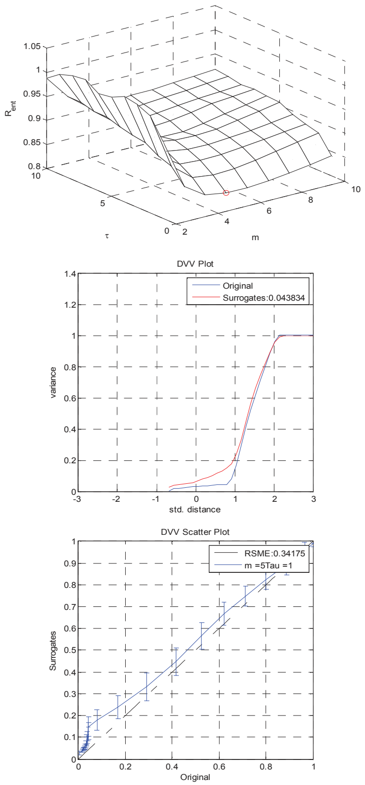

STRAIN 4

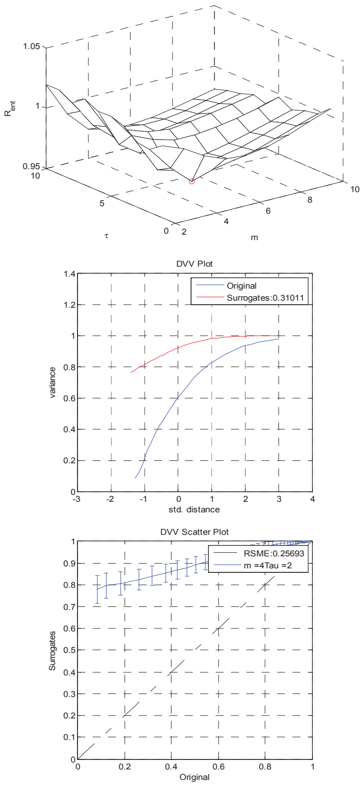

EXP 1

STRAIN 1 | STRAIN 2 | STRAIN 3 | STRAIN 4

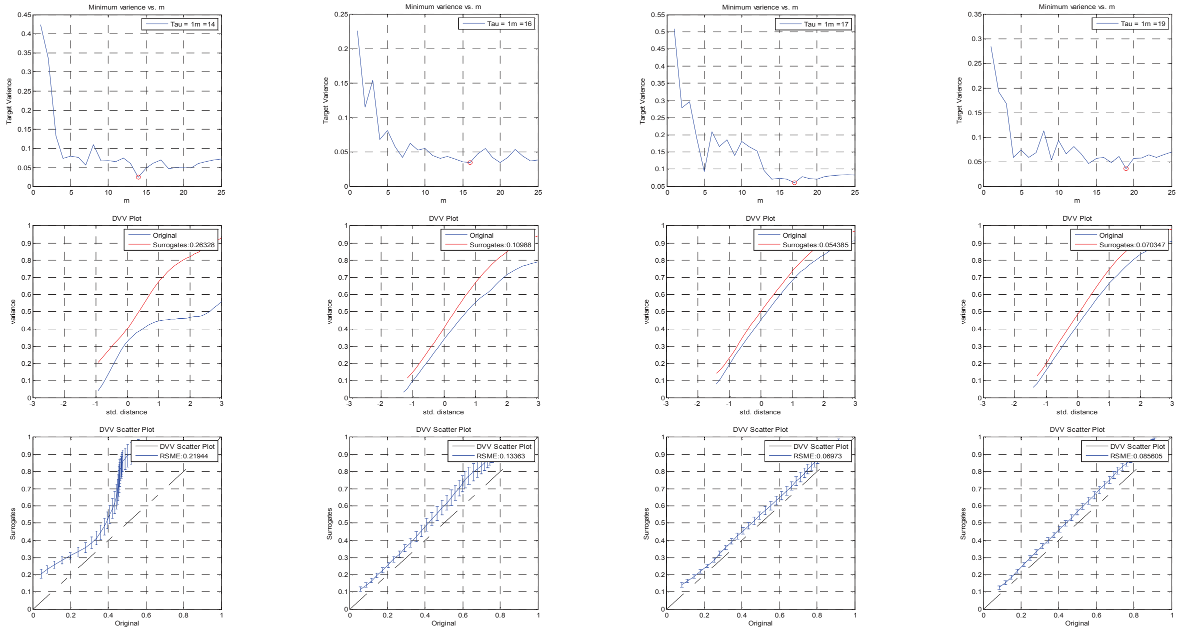

STRAIN 1 | STRAIN 2 | STRAIN 3 | STRAIN 4

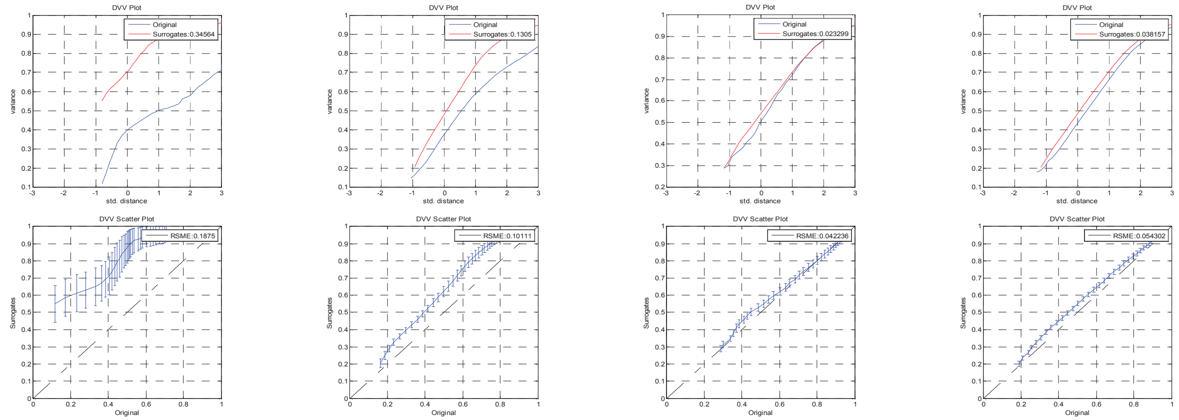

EXPERIMENT	VARIABLES		METHOD 1				METHOD 2				METHOD 3			
			best **m**	best **τ**	rsme	RSME	calc **m**	**τ**	rsme	RSME	set **m**	set **τ**	rsme	RSME
2	Initial exp<br>Angle of incidence 8 degree	CH1	5	1	0.6093	0.3173	5	1	0.3588	0.2668	3	1	0.4816	0.3171
		CH2	5	8	0.9668	0.5607	23	1	0.3603	0.5924	3	1	0.2699	0.2972
		CH3	5	1	0.8097	0.4405	9	1	0.9294	0.5168	3	1	0.8021	0.4437
		LDVg	4	9	0.9328	0.5527	14	1	0.8386	0.5160	3	1	0.4085	0.3380
		LDV1	6	3	0.0671	0.1716	8	1	0.0213	0.1324	3	1	0.0119	0.1794
		LDV2	5	3	0.1485	0.1734	14	1	0.4531	0.2417	3	1	0.3783	0.1961
		Strain 1	9	8	0.6019	0.3269	19	1	0.2566	0.2387	3	1	0.2918	0.1683
		Strain 2	8	7	0.5645	0.3068	19	1	0.1864	0.1825	3	1	0.0941	0.1157
		Strain 3	8	7	0.4423	0.2436	17	1	0.1131	0.1043	3	1	0.0545	0.0430
		Strain 4	7	7	0.3938	0.2139	19	1	0.1028	0.1161	3	1	0.0666	0.0737

Data recorded 3D Accelerometer

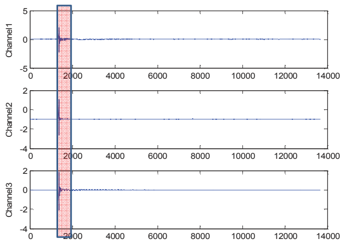

Data analysed 3D Accelerometer

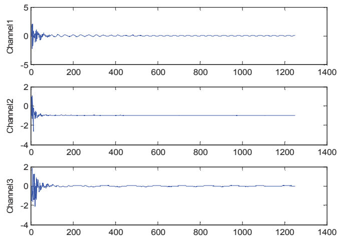

METHOD 1

CH1

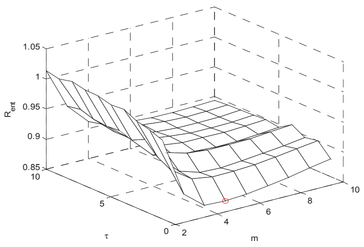

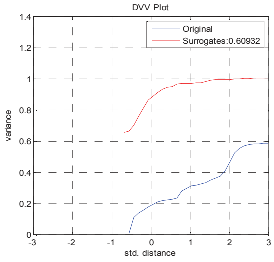

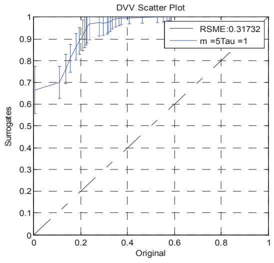

CH2

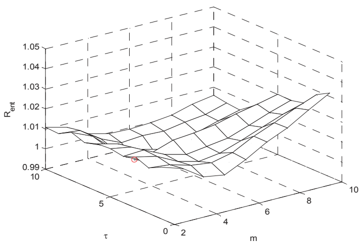

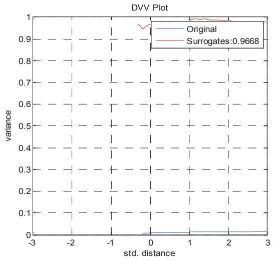

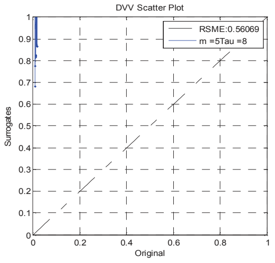

CH3

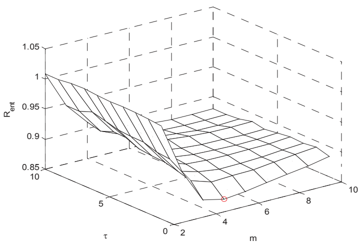

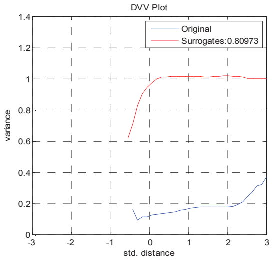

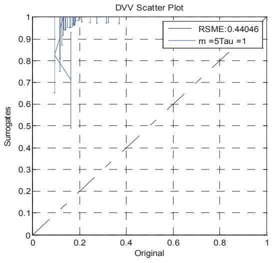

EXP 2 METHOD 2

CH1

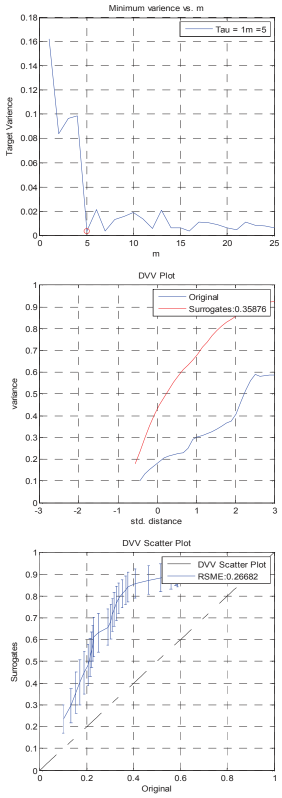

CH2

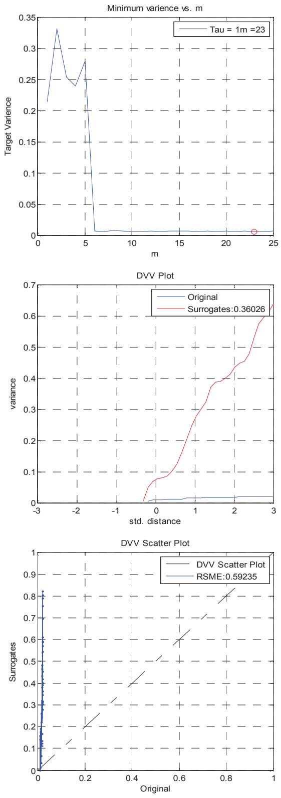

CH3

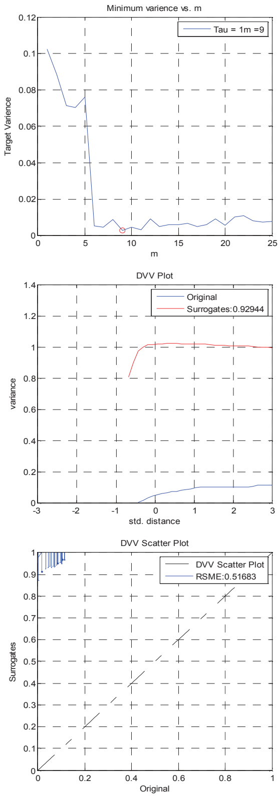

METHOD 3

CH1

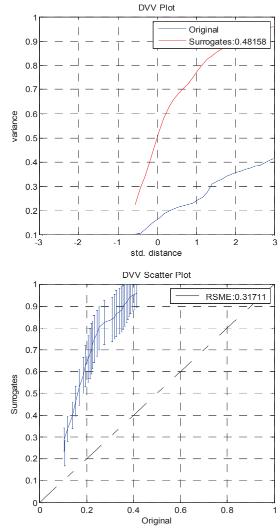

CH2

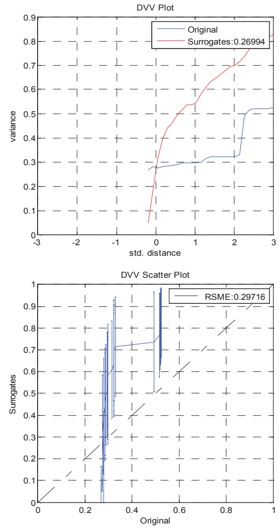

CH3

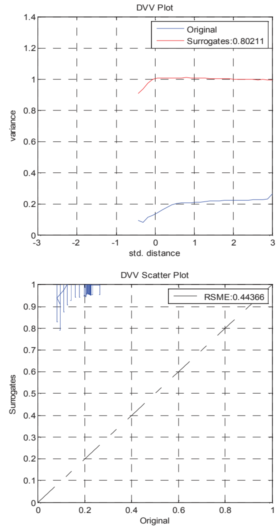

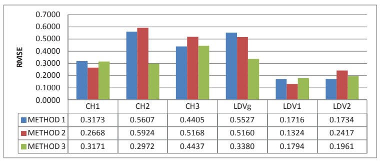

	CH1	CH2	CH3	LDVg	LDV1	LDV2
METHOD 1	0.3173	0.5607	0.4405	0.5527	0.1716	0.1734
METHOD 2	0.2668	0.5924	0.5168	0.5160	0.1324	0.2417
METHOD 3	0.3171	0.2972	0.4437	0.3380	0.1794	0.1961

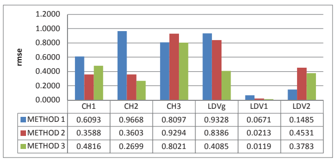

	CH1	CH2	CH3	LDVg	LDV1	LDV2
METHOD 1	0.6093	0.9668	0.8097	0.9328	0.0671	0.1485
METHOD 2	0.3588	0.3603	0.9294	0.8386	0.0213	0.4531
METHOD 3	0.4816	0.2699	0.8021	0.4085	0.0119	0.3783

Data recorded LDV

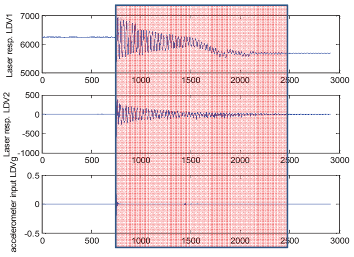

Data analysed LDV

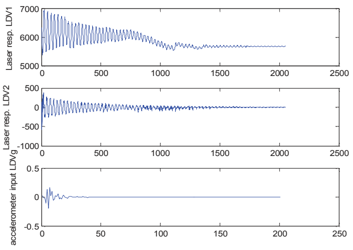

EXP 2

LDVg

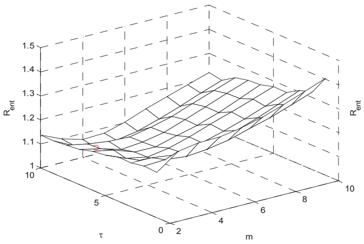

LDV1

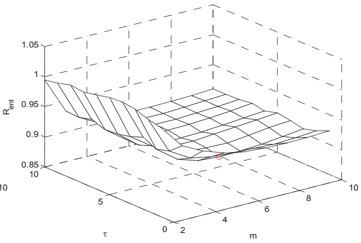

LDV2

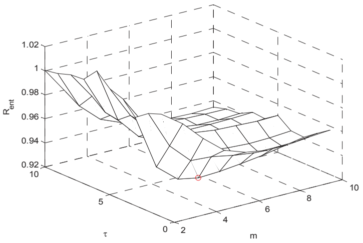

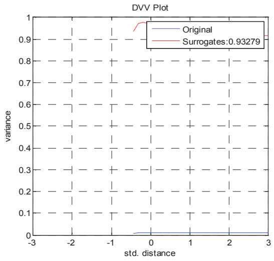

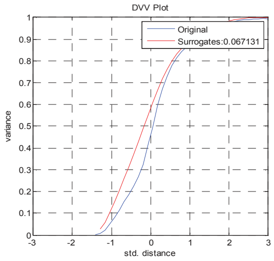

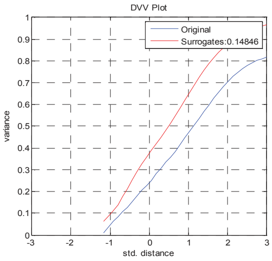

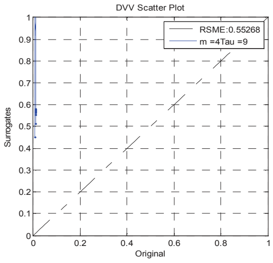

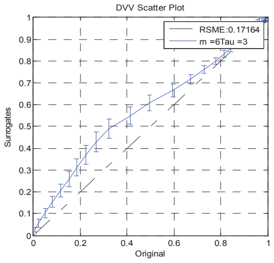

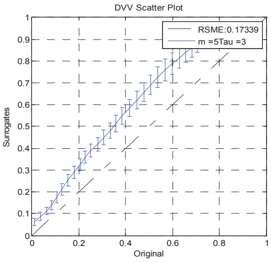

EXP 2

LDVg

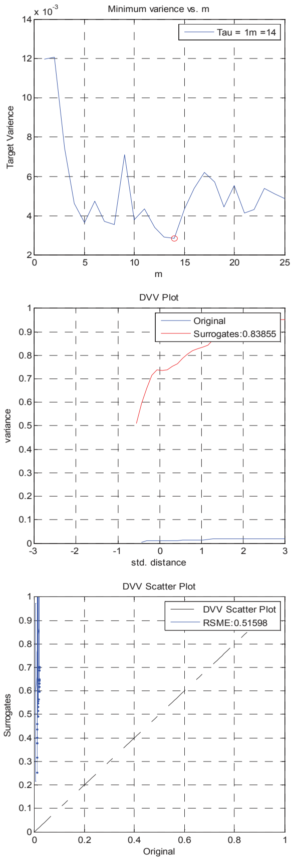

LDV1

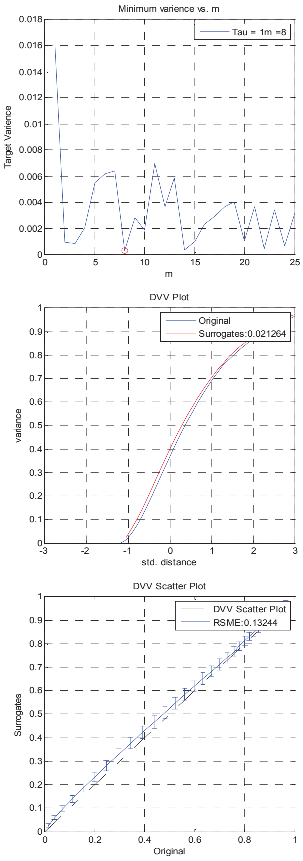

LDV2

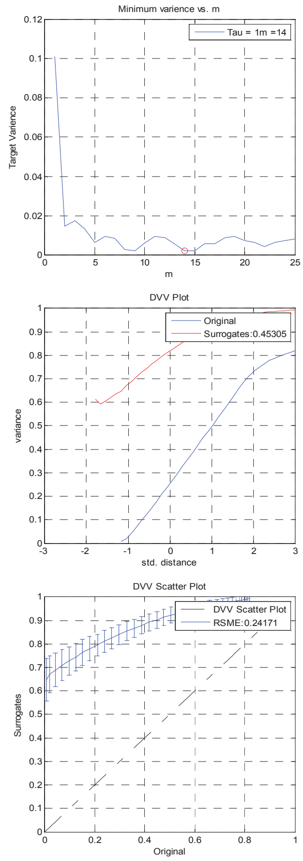

LDVg

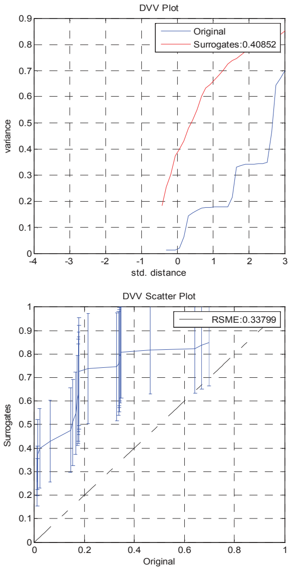

LDV1

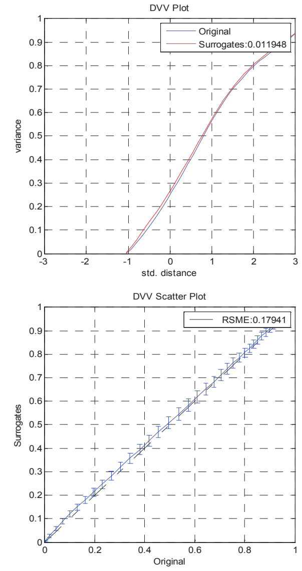

LDV2

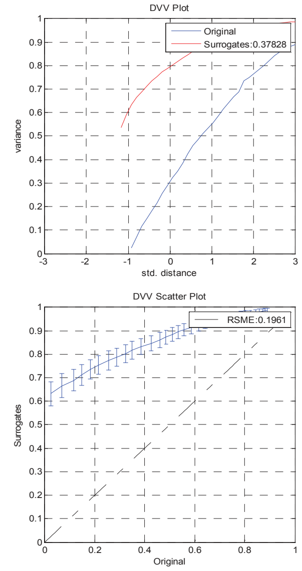

Data recorded Strain Gauge

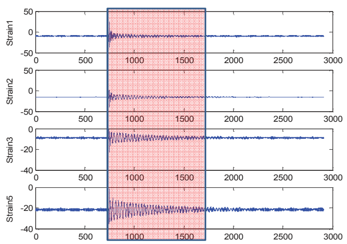

Data analysed Strain Gauge

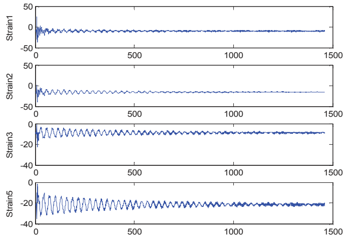

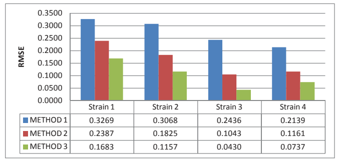

	Strain 1	Strain 2	Strain 3	Strain 4
METHOD 1	0.3269	0.3068	0.2436	0.2139
METHOD 2	0.2387	0.1825	0.1043	0.1161
METHOD 3	0.1683	0.1157	0.0430	0.0737

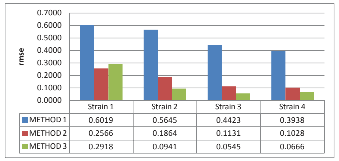

	Strain 1	Strain 2	Strain 3	Strain 4
METHOD 1	0.6019	0.5645	0.4423	0.3938
METHOD 2	0.2566	0.1864	0.1131	0.1028
METHOD 3	0.2918	0.0941	0.0545	0.0666

EXP 2

STRAIN 1

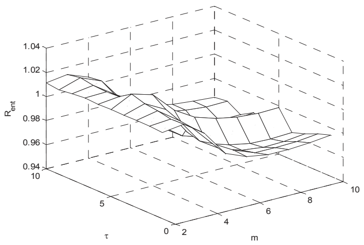

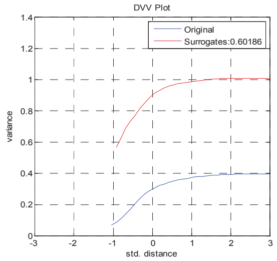

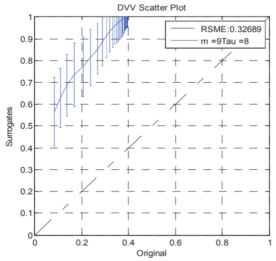

STRAIN 2

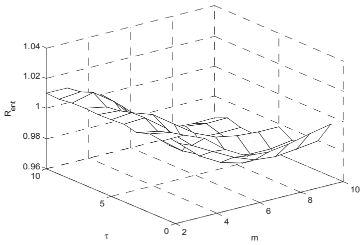

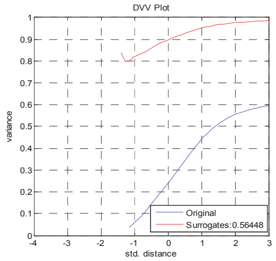

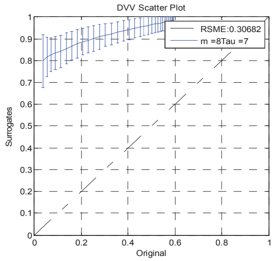

STRAIN 3

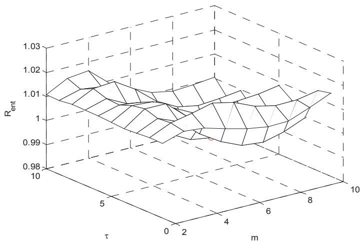

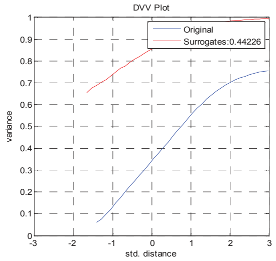

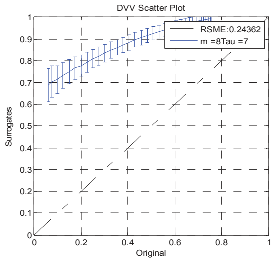

STRAIN 4

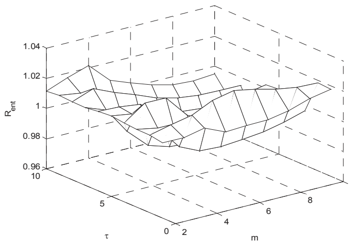

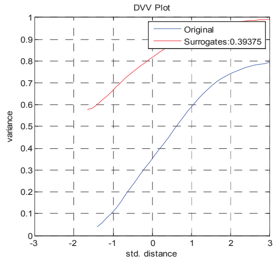

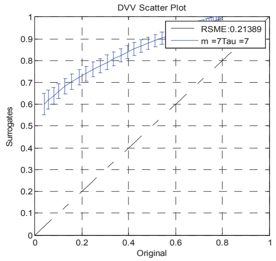

EXP 2

STRAIN 1 | STRAIN 2 | STRAIN 3 | STRAIN 4

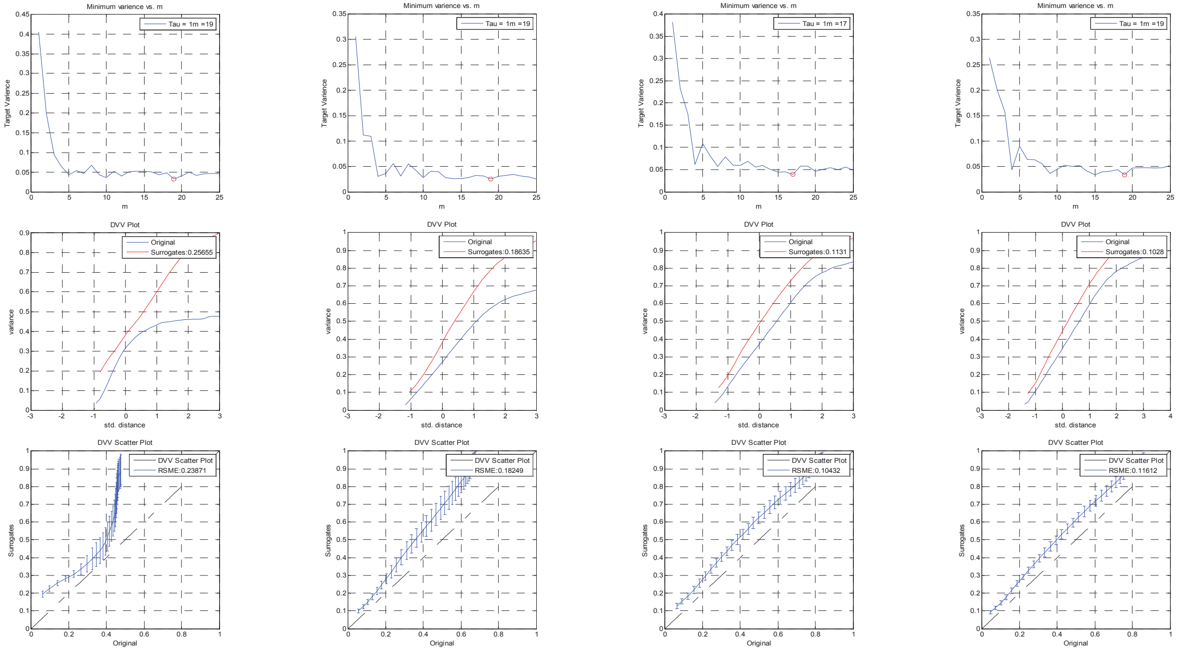

STRAIN 1 | STRAIN 2 | STRAIN 3 | STRAIN 4

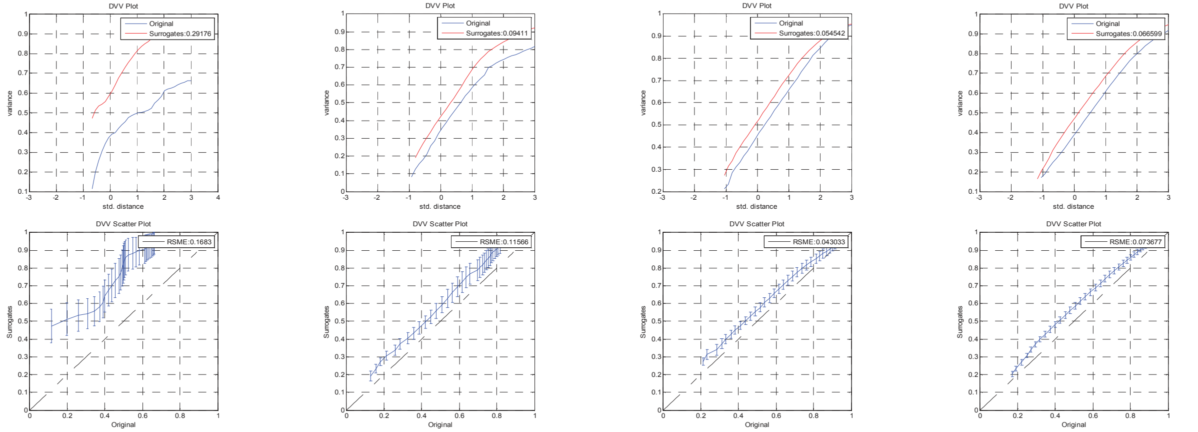

EXPERIMENT	VARIABLES		METHOD 1				METHOD 2				METHOD 3			
			best **m**	best **τ**	rsme	RSME	calc **m**	**τ**	rsme	RSME	set **m**	set **τ**	rsme	RSME
3	FOCUS AT THE ACCELEROMETER	CH1	5	1	0.4504	0.3275	22	1	0.5728	0.3890	3	1	0.3546	0.2426
		CH2	6	1	0.5751	0.4730	12	1	0.4747	0.3636	3	1	0.6560	0.4446
		CH3	5	1	0.5871	0.4145	17	1	0.8665	0.4817	3	1	0.8260	0.4483
		LDVg	4	4	0.3597	0.2869	9	1	0.2614	0.2851	3	1	0.1330	0.4179
		LDV1	6	1	0.1596	0.5475	18	1	0.1519	0.5148	3	1	0.0494	0.5983
		LDV2	5	3	0.4586	0.2360	15	1	0.4340	0.2332	3	1	0.3864	0.2038
		Strain 1	9	8	0.5881	0.3173	12	1	0.2616	0.2070	3	1	0.3687	0.2082
		Strain 2	9	10	0.6479	0.3525	14	1	0.1868	0.1676	3	1	0.1133	0.1077
		Strain 3	9	9	0.5000	0.3055	16	1	0.0878	0.0847	3	1	0.0595	0.0413
		Strain 5	7	7	0.3831	0.2087	23	1	0.1138	0.1097	3	1	0.0591	0.0695

Data recorded 3D Accelerometer

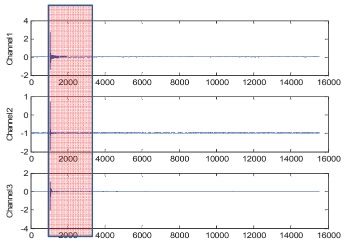

Data analysed 3D Accelerometer

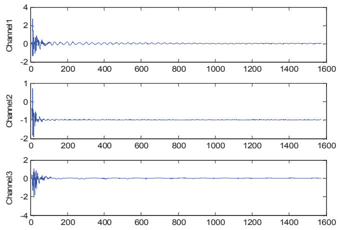

METHOD 1

CH1 **CH2** **CH3**

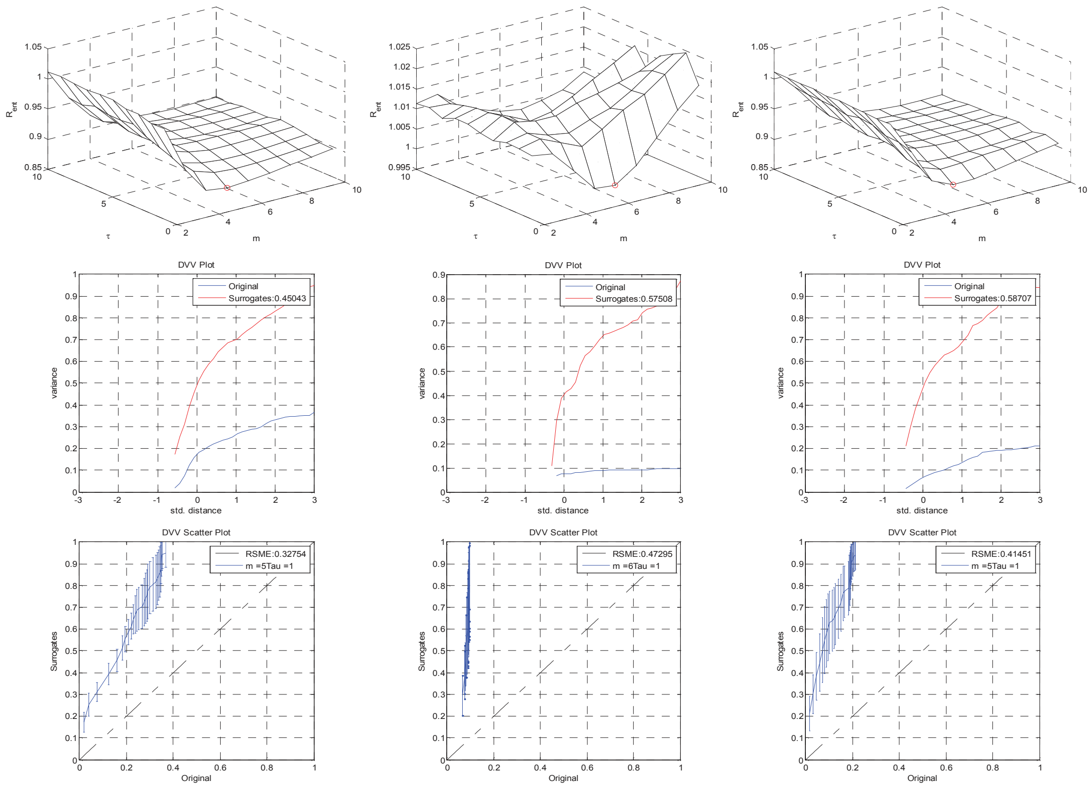

EXP 3 METHOD 2

CH1 | CH2 | CH3

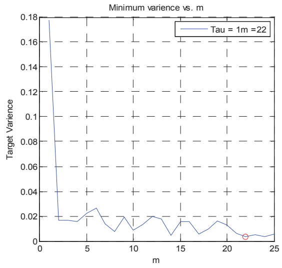

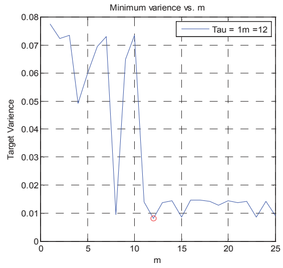

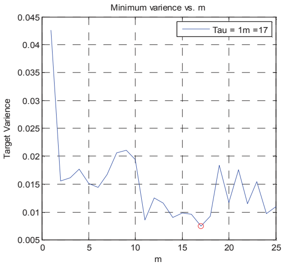

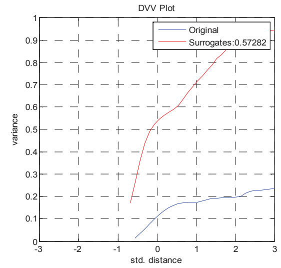

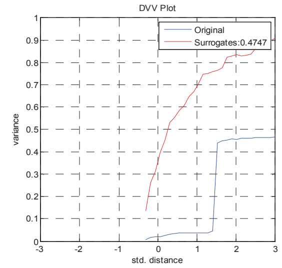

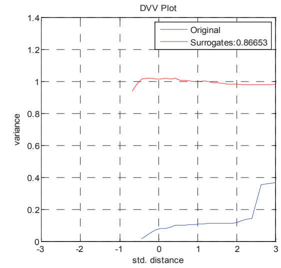

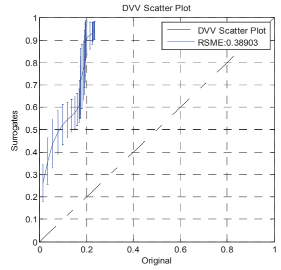

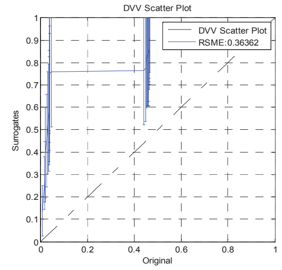

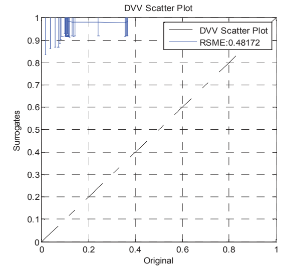

METHOD 3

CH1 | CH2 | CH3

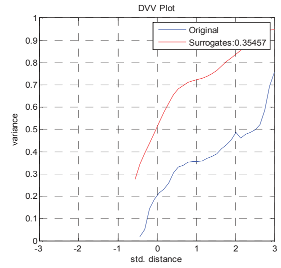

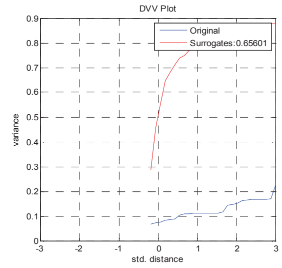

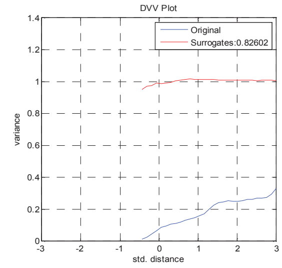

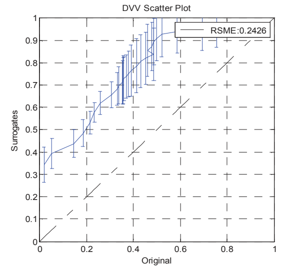

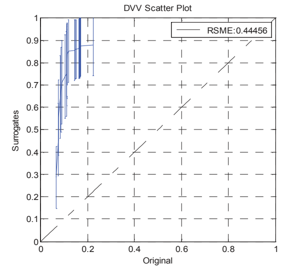

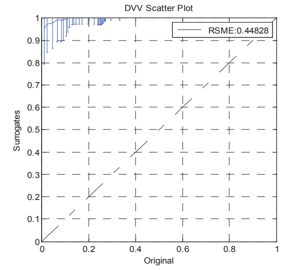

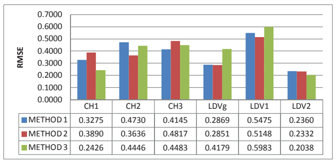

	CH1	CH2	CH3	LDVg	LDV1	LDV2
METHOD 1	0.3275	0.4730	0.4145	0.2869	0.5475	0.2360
METHOD 2	0.3890	0.3636	0.4817	0.2851	0.5148	0.2332
METHOD 3	0.2426	0.4446	0.4483	0.4179	0.5983	0.2038

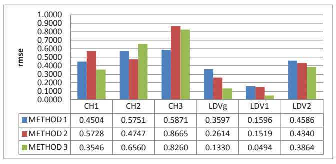

	CH1	CH2	CH3	LDVg	LDV1	LDV2
METHOD 1	0.4504	0.5751	0.5871	0.3597	0.1596	0.4586
METHOD 2	0.5728	0.4747	0.8665	0.2614	0.1519	0.4340
METHOD 3	0.3546	0.6560	0.8260	0.1330	0.0494	0.3864

Data recorded LDV

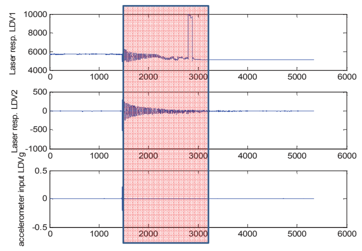

Data analysed LDV

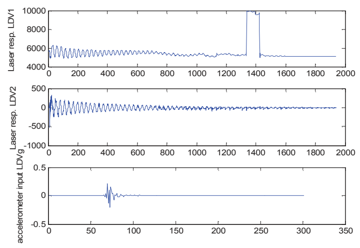

EXP 3

LDVg

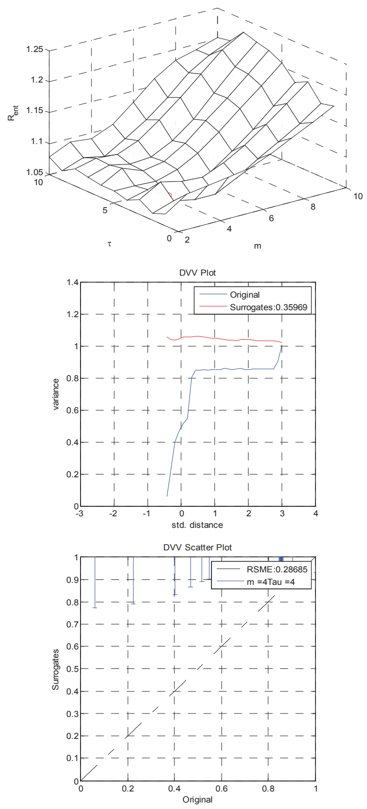

LDV1

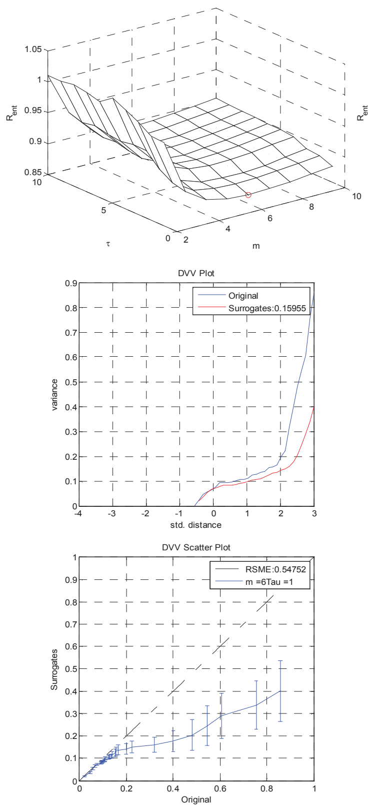

LDV2

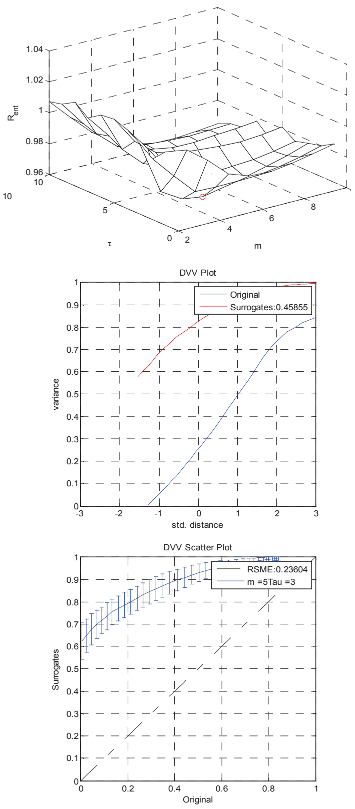

EXP 3

LDVg	LDV1	LDV2

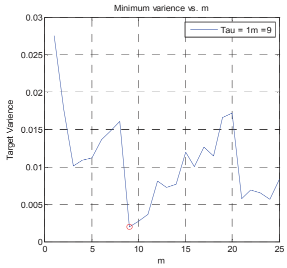

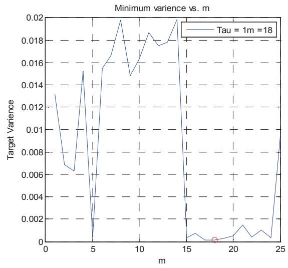

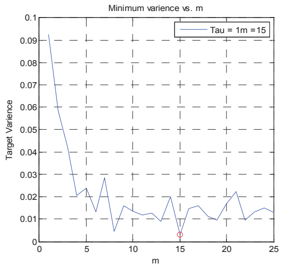

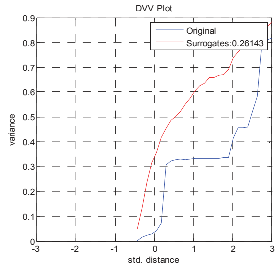

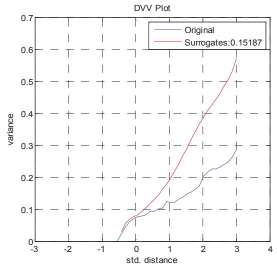

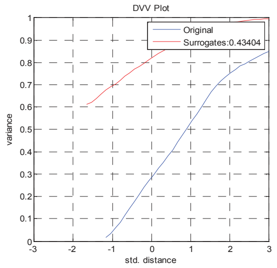

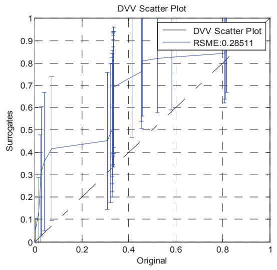

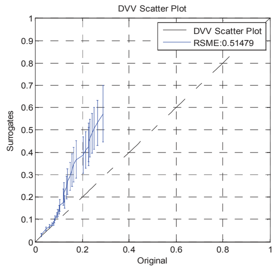

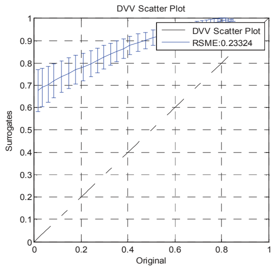

LDVg	LDV1	LDV2

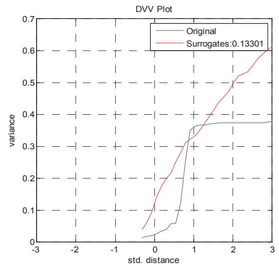

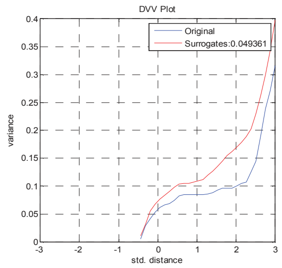

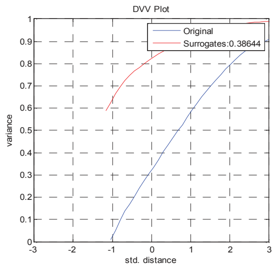

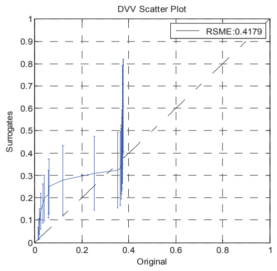

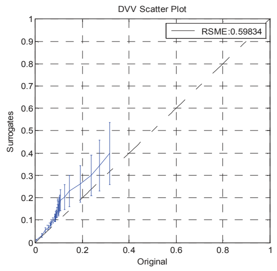

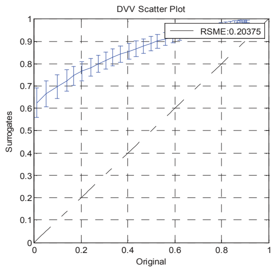

Data recorded Strain Gauge

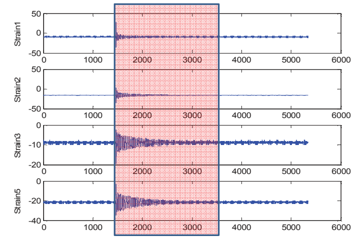

Data analysed Strain Gauge

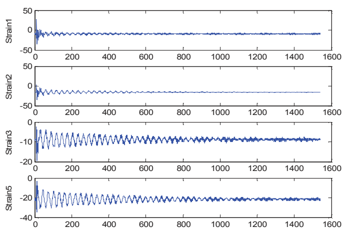

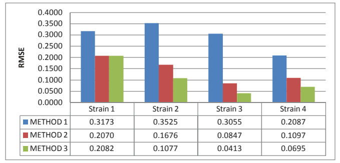

	Strain 1	Strain 2	Strain 3	Strain 4
METHOD 1	0.3173	0.3525	0.3055	0.2087
METHOD 2	0.2070	0.1676	0.0847	0.1097
METHOD 3	0.2082	0.1077	0.0413	0.0695

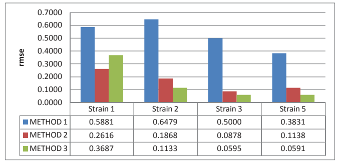

	Strain 1	Strain 2	Strain 3	Strain 5
METHOD 1	0.5881	0.6479	0.5000	0.3831
METHOD 2	0.2616	0.1868	0.0878	0.1138
METHOD 3	0.3687	0.1133	0.0595	0.0591

EXP 3

STRAIN 1

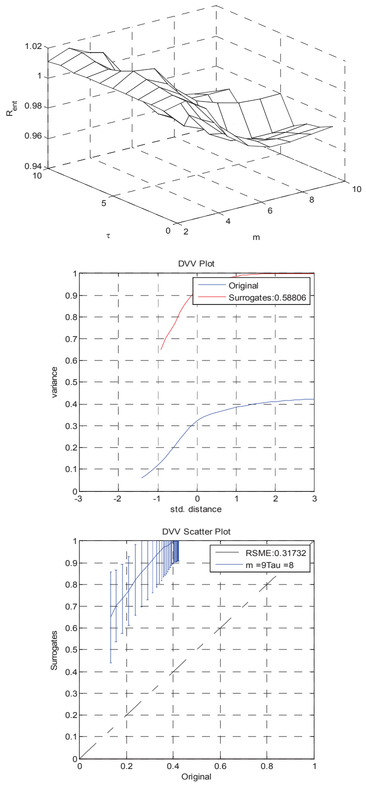

STRAIN 2

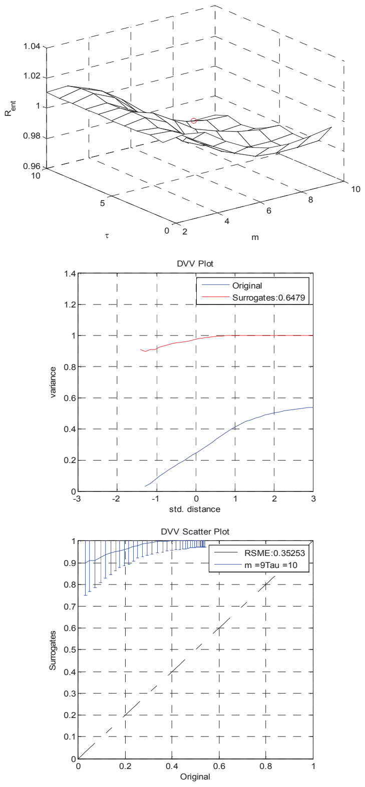

STRAIN 3

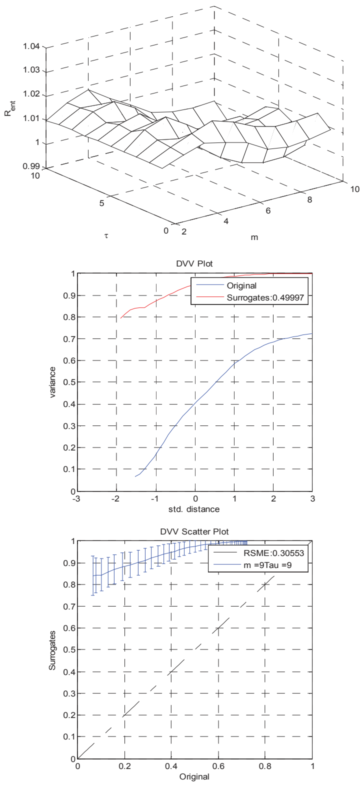

STRAIN 4

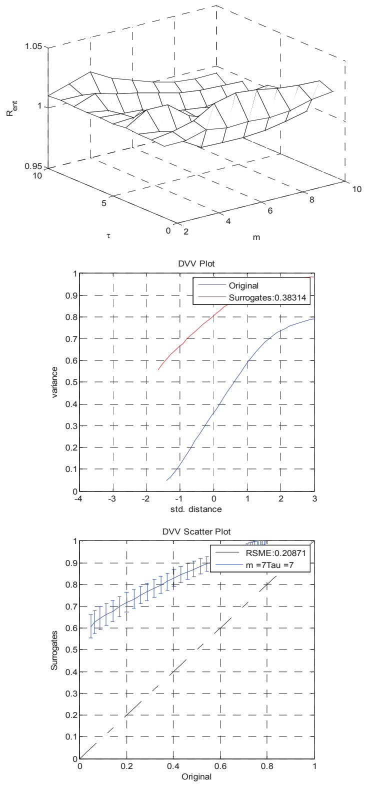

EXP 3

STRAIN 1

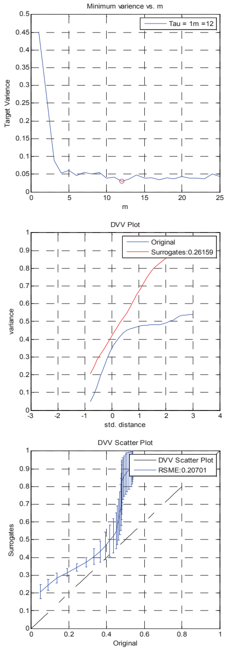

STRAIN 2

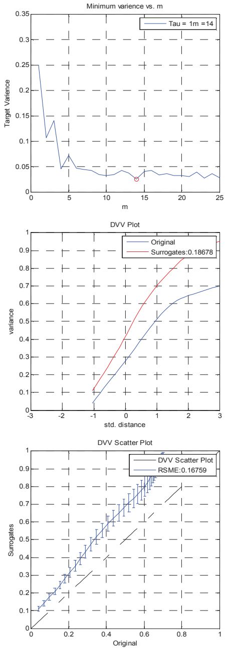

STRAIN 3

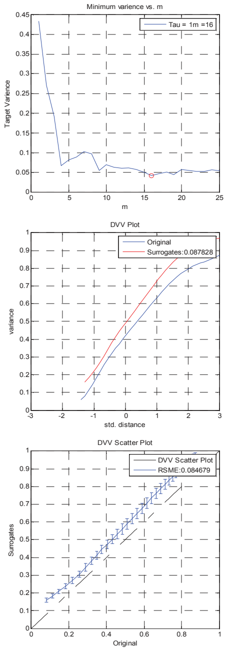

STRAIN 4

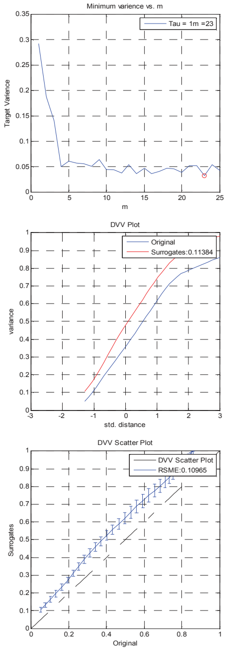

STRAIN 1

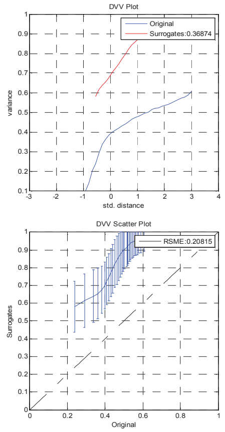

STRAIN 2

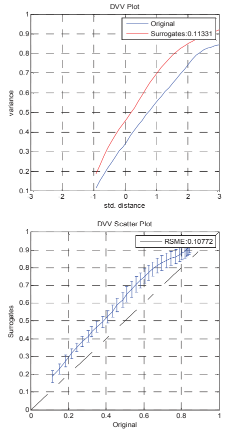

STRAIN 3

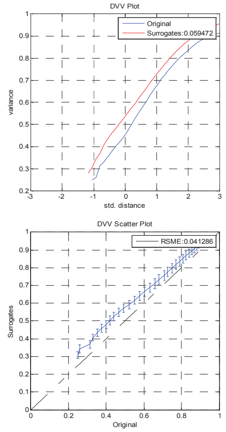

STRAIN 4

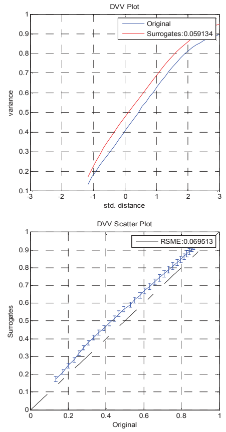

EXPERIMENT	VARIABLES		METHOD 1				METHOD 2				METHOD 3			
			best **m**	best **τ**	rsme	RSME	calc **m**	**τ**	rsme	RSME	set **m**	set **τ**	rsme	RSME
4	with knocks, no movement, best result	CH1	6	2	0.0636	0.1588	24	1	0.0935	0.2328	3	1	0.0541	0.2235
		CH2	6	2	0.2161	0.3215	17	1	0.1887	0.5295	3	1	0.1357	0.4681
		CH3	9	10	0.5314	0.3025	12	1	0.2107	0.1925	3	1	0.3073	0.2552
		LDVg	4	9	0.8576	0.5388	24	1	0.7246	0.5184	3	1	0.1639	0.3749
		LDV1	6	6	0.1467	0.1748	4	1	0.0136	0.1512	3	1	0.0432	0.1841
		LDV2	6	10	0.4526	0.2310	4	1	0.2622	0.1386	3	1	0.2510	0.1401
		Strain 1	7	7	0.2510	0.1820	19	1	0.2288	0.1852	3	1	0.2072	0.1376
		Strain 2	7	7	0.1704	0.1744	22	1	0.1201	0.1642	3	1	0.0469	0.1272
		Strain 3	7	7	0.3900	0.1977	24	1	0.2027	0.1712	3	1	0.1166	0.1391
		Strain 4	6	7	0.3486	0.1778	24	1	0.1359	0.1640	3	1	0.0758	0.1462

Data recorded 3D Accelerometer

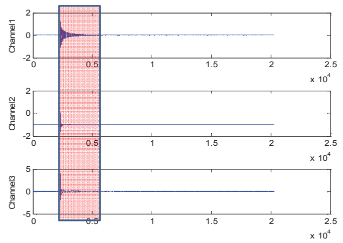

Data analysed 3D Accelerometer

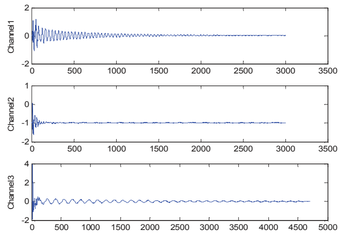

METHOD 1

CH1

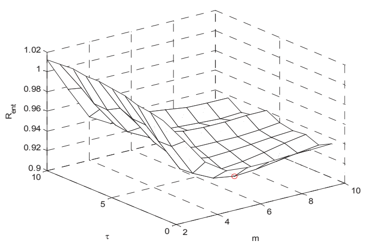

CH2

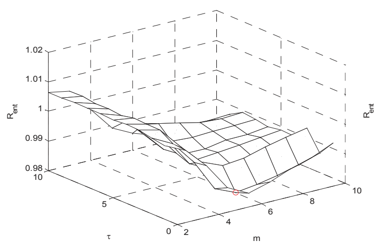

CH3

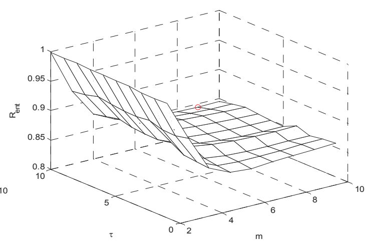

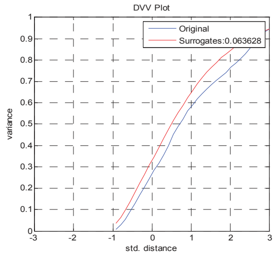

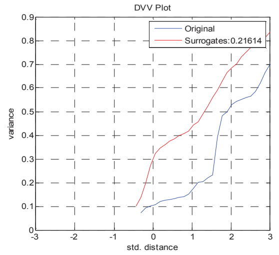

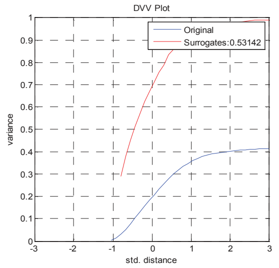

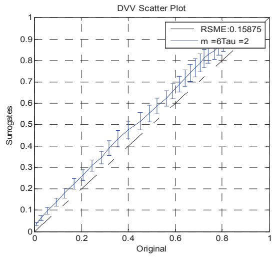

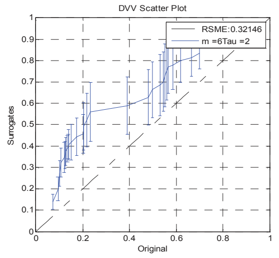

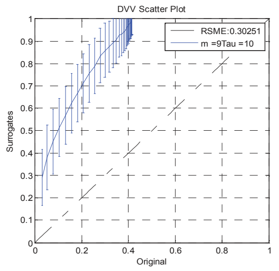

EXP 4 METHOD 2

CH1 CH2 CH3

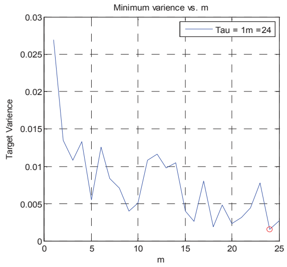

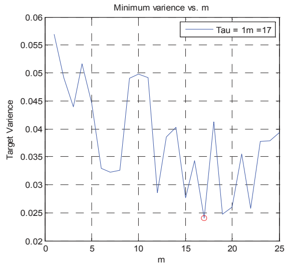

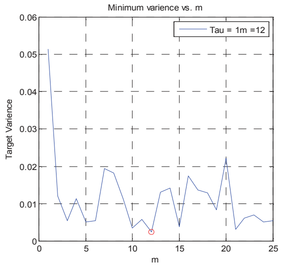

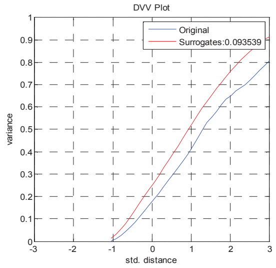

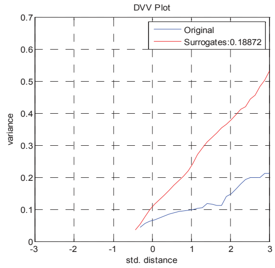

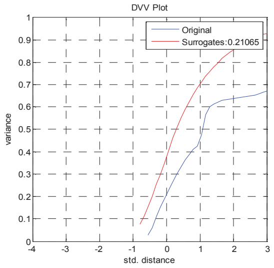

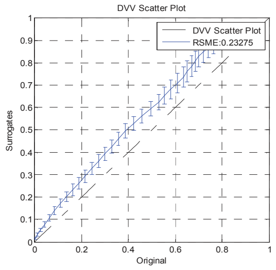

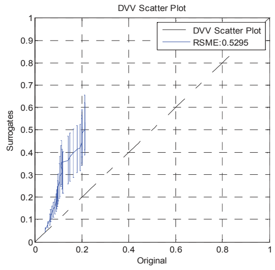

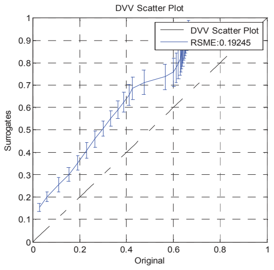

METHOD 3

CH1 CH2 CH3

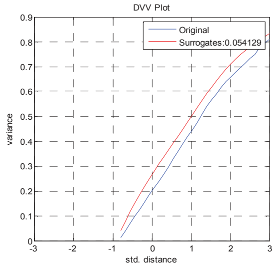

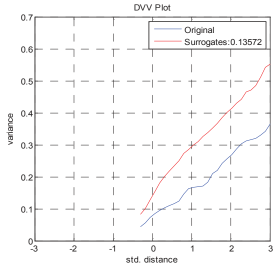

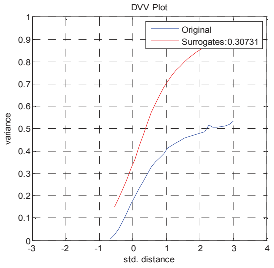

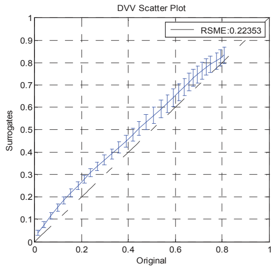

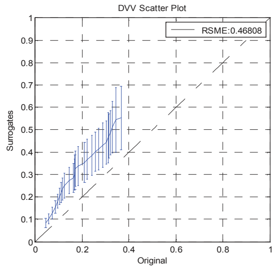

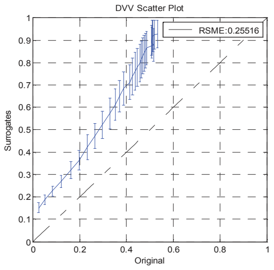

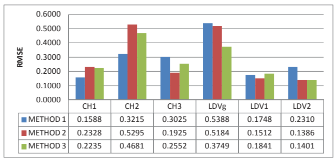

	CH1	CH2	CH3	LDVg	LDV1	LDV2
METHOD 1	0.1588	0.3215	0.3025	0.5388	0.1748	0.2310
METHOD 2	0.2328	0.5295	0.1925	0.5184	0.1512	0.1386
METHOD 3	0.2235	0.4681	0.2552	0.3749	0.1841	0.1401

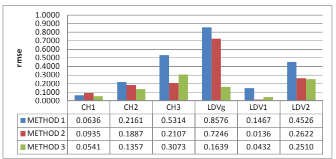

	CH1	CH2	CH3	LDVg	LDV1	LDV2
METHOD 1	0.0636	0.2161	0.5314	0.8576	0.1467	0.4526
METHOD 2	0.0935	0.1887	0.2107	0.7246	0.0136	0.2622
METHOD 3	0.0541	0.1357	0.3073	0.1639	0.0432	0.2510

Data recorded LDV

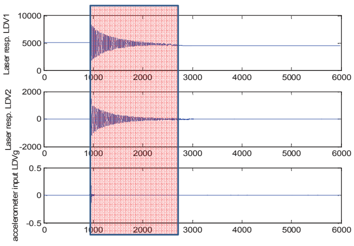

Data analysed LDV

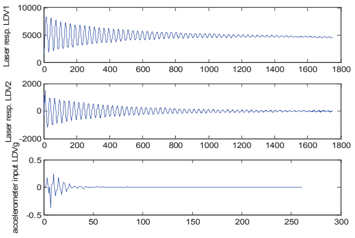

EXP 4

LDVg **LDV1** **LDV2**

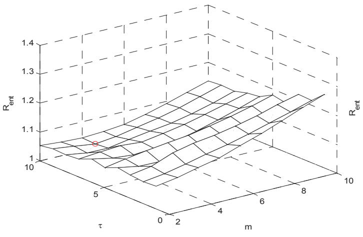

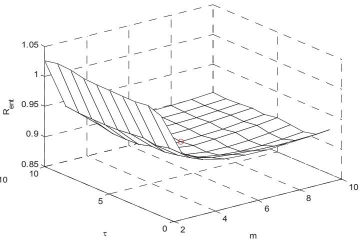

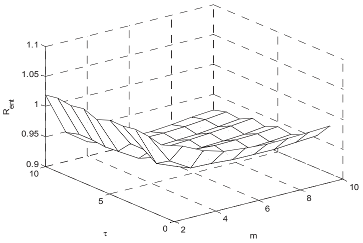

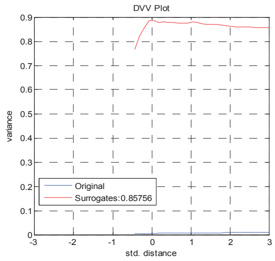

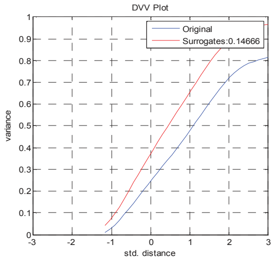

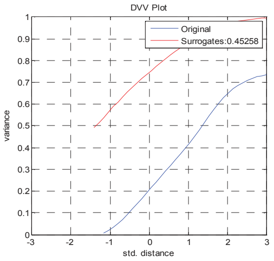

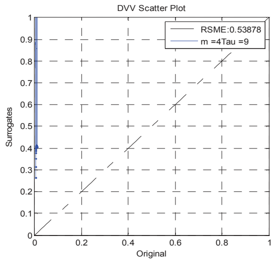

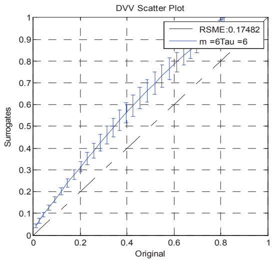

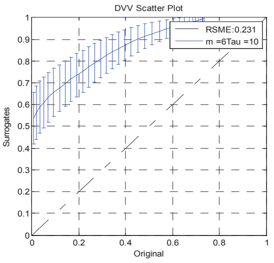

EXP 4

LDVg | LDV1 | LDV2

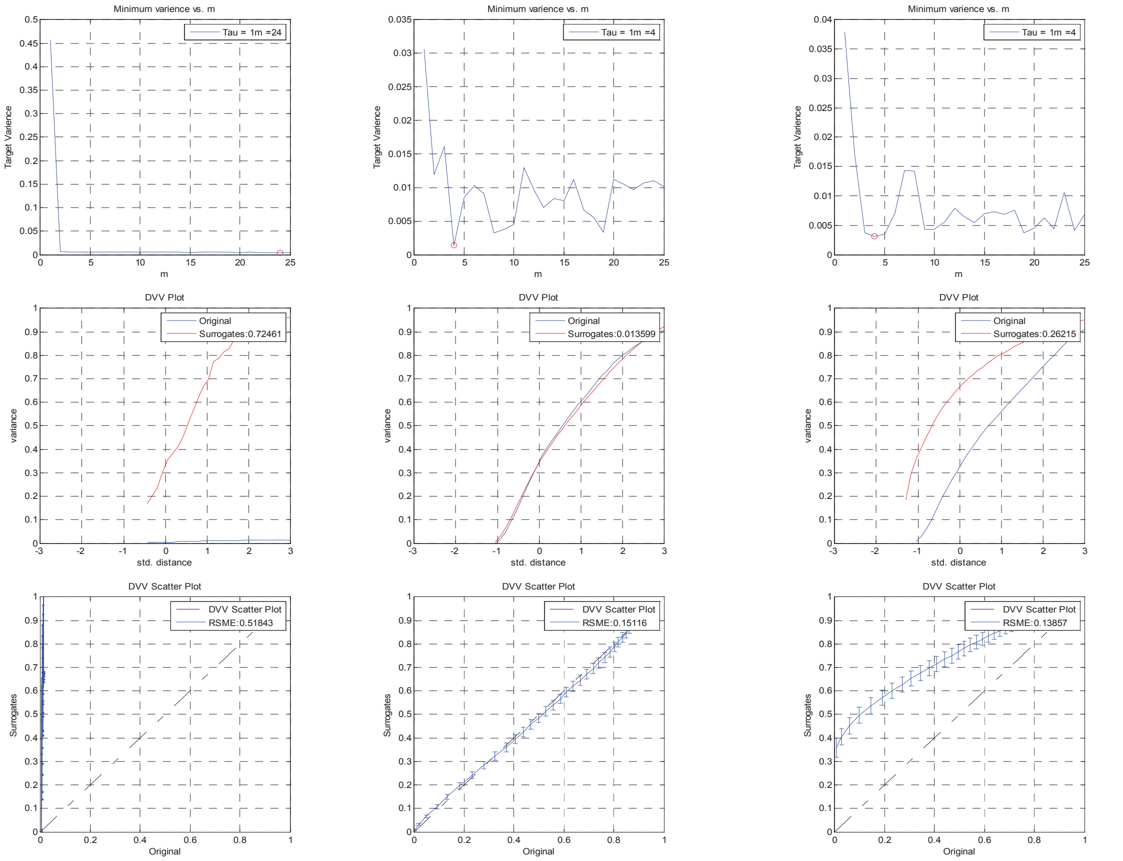

LDVg | LDV1 | LDV2

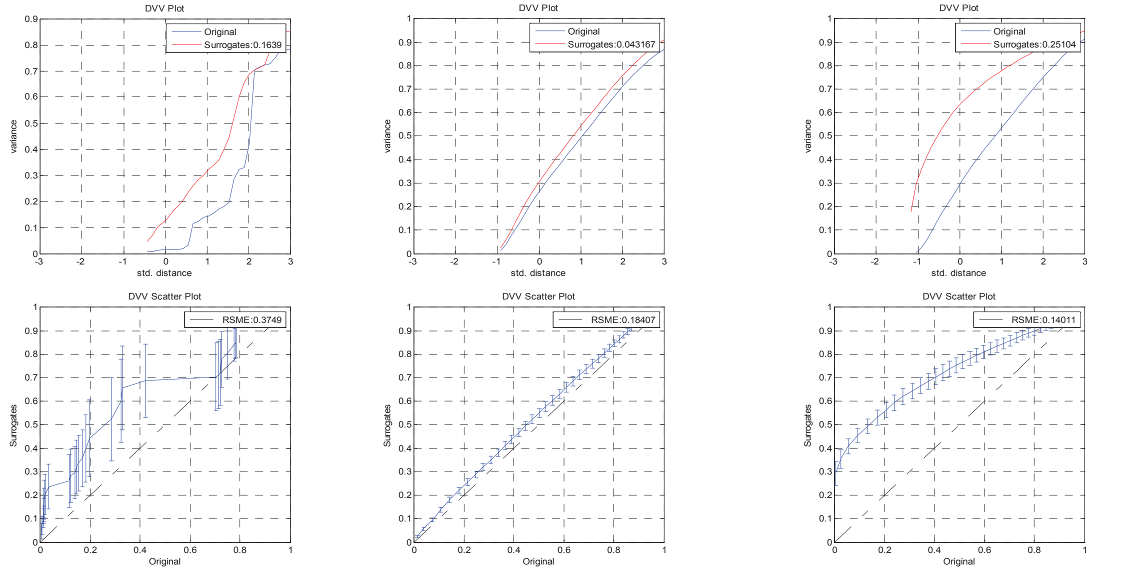

Data recorded Strain Gauge

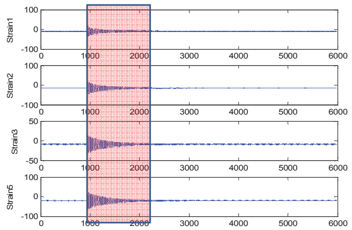

Data analysed Strain Gauge

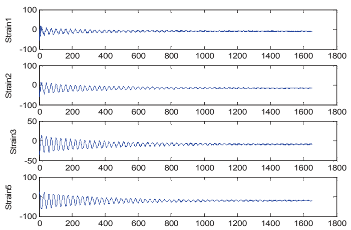

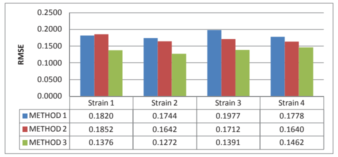

	Strain 1	Strain 2	Strain 3	Strain 4
METHOD 1	0.1820	0.1744	0.1977	0.1778
METHOD 2	0.1852	0.1642	0.1712	0.1640
METHOD 3	0.1376	0.1272	0.1391	0.1462

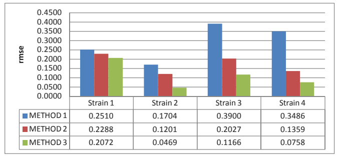

	Strain 1	Strain 2	Strain 3	Strain 4
METHOD 1	0.2510	0.1704	0.3900	0.3486
METHOD 2	0.2288	0.1201	0.2027	0.1359
METHOD 3	0.2072	0.0469	0.1166	0.0758

EXP 4

STRAIN 1

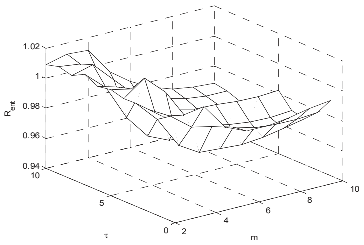

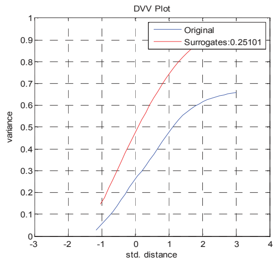

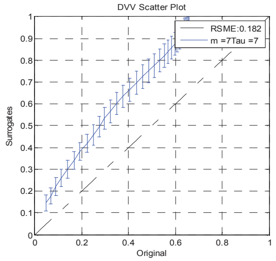

STRAIN 2

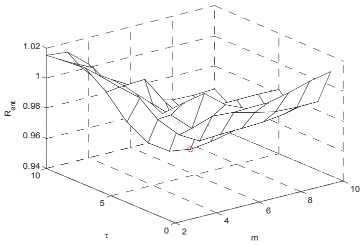

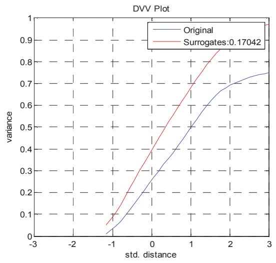

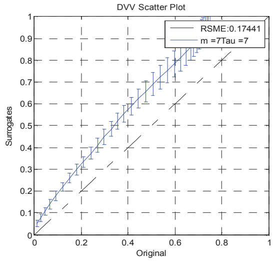

STRAIN 3

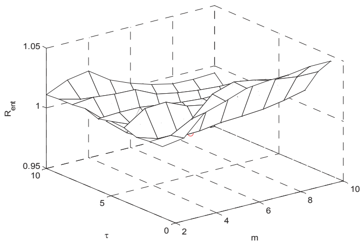

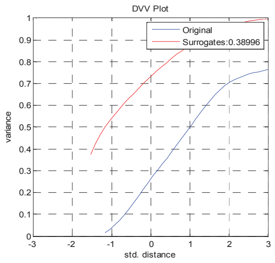

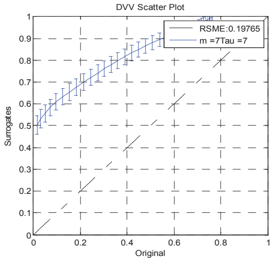

STRAIN 4

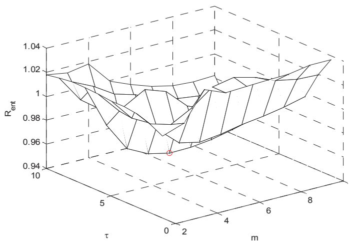

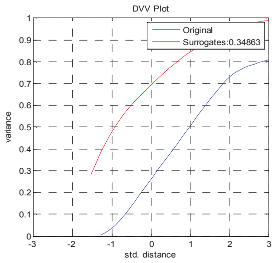

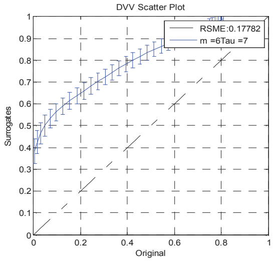

EXP 4

STRAIN 1

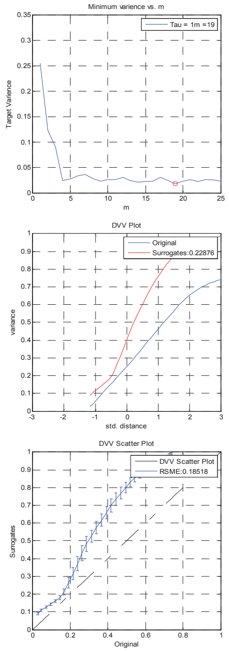

STRAIN 2

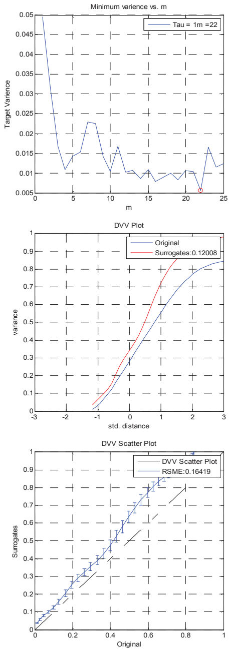

STRAIN 3

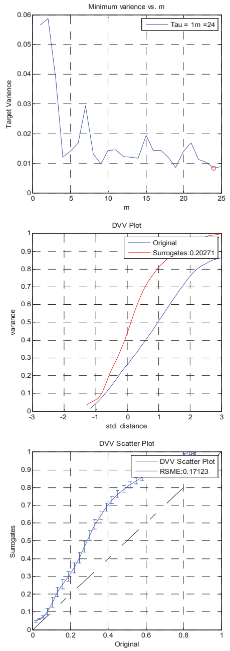

STRAIN 4

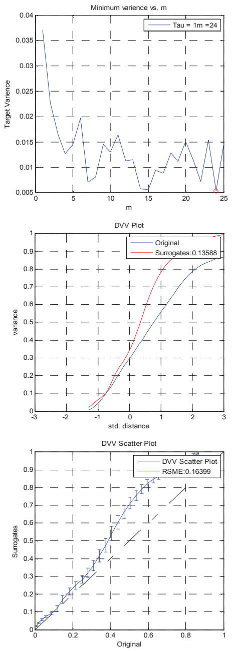

STRAIN 1

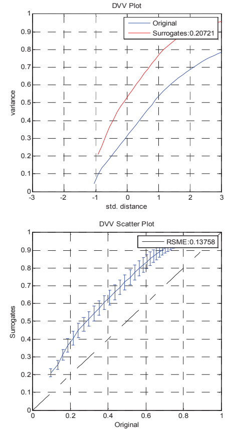

STRAIN 2

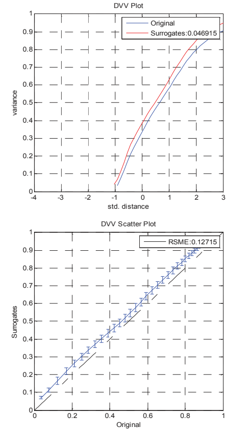

STRAIN 3

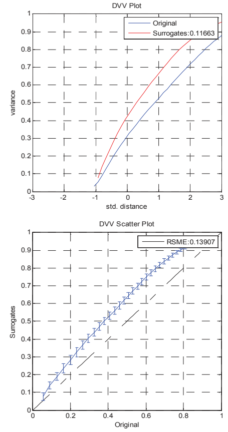

STRAIN 4

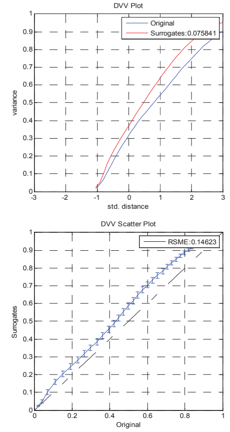

EXPERIMENT	VARIABLES		METHOD 1				METHOD 2				METHOD 3			
			best **m**	best **τ**	rsme	RSME	calc **m**	**τ**	rsme	RSME	set **m**	set **τ**	rsme	RSME
5	Harmonic resonance 4.4Hz Focus at accelerometer	CH1	6	10	0.0572	0.1473	13	1	0.0206	0.0943	3	1	0.0154	0.0872
		CH2	6	9	0.2603	0.2511	18	1	0.0178	0.1082	3	1	0.0139	0.1140
		CH3	3	9	0.0311	0.2153	7	1	0.0025	0.1630	3	1	0.0027	0.1741
		LDV	6	9	0.1335	0.2412	15	1	0.0863	0.1701	3	1	0.0595	0.1685
		LDV1	4	1	0.0070	0.1382	4	1	0.0090	0.1386	3	1	0.0031	0.1345
		LDV2	10	10	0.1712	0.1689	15	1	0.1024	0.1561	3	1	0.1424	0.1490
		Strain 1	10	9	0.1363	0.1717	4	1	0.1029	0.1583	3	1	0.1087	0.1381
		Strain 2	10	9	0.1358	0.1728	14	1	0.1973	0.2004	3	1	0.1003	0.1408
		Strain 3	10	9	0.1342	0.1726	4	1	0.1294	0.1608	3	1	0.1163	0.1417
		Strain 4	10	9	0.1340	0.1733	15	1	0.2504	0.2158	3	1	0.1123	0.1423

Data recorded 3D Accelerometer

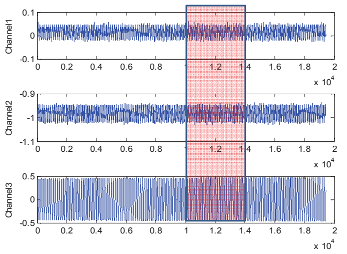

Data analysed 3D Accelerometer

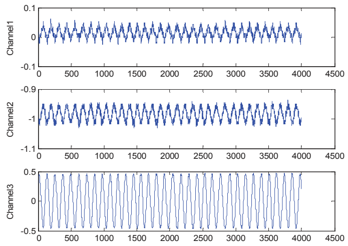

METHOD 1

CH1

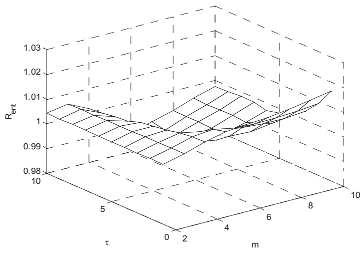

CH2

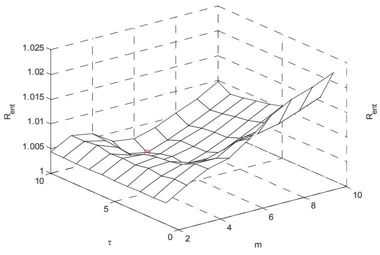

CH3

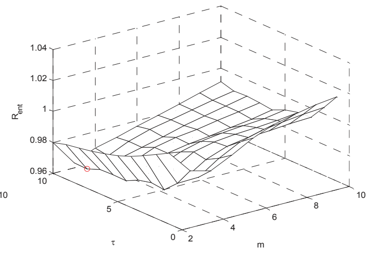

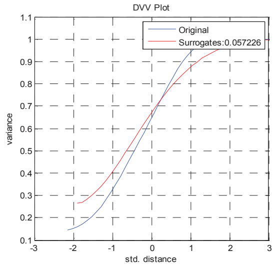

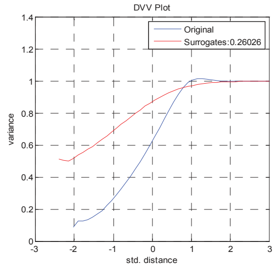

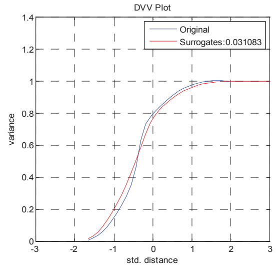

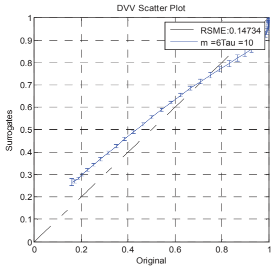

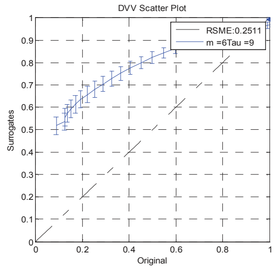

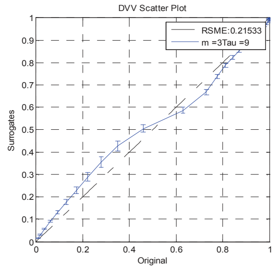

EXP 5 METHOD 2

CH1	CH2	CH3

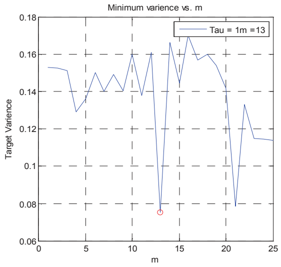

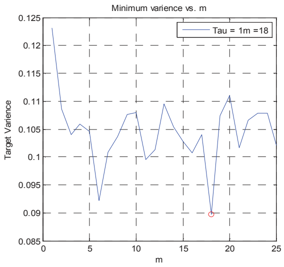

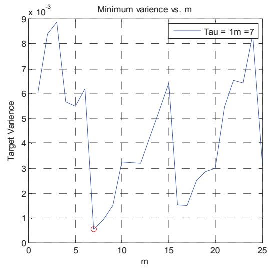

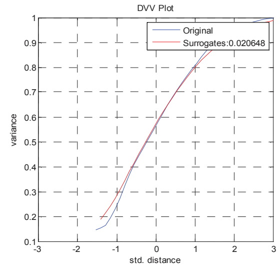

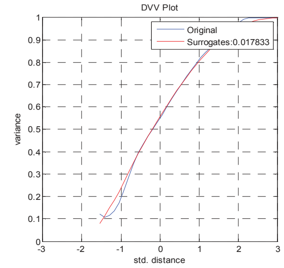

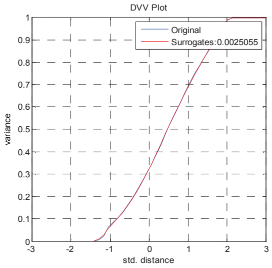

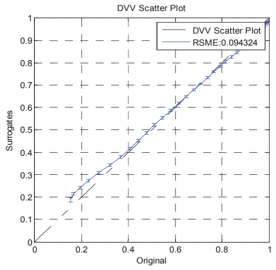

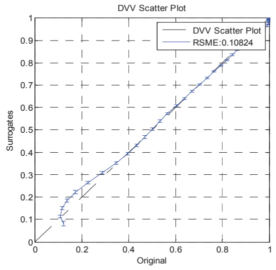

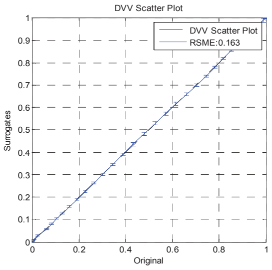

METHOD 3

CH1	CH2	CH3

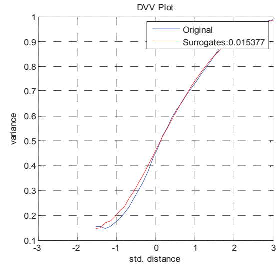

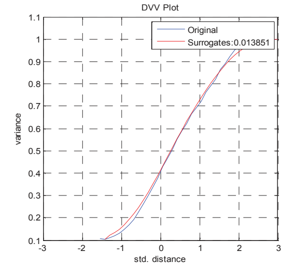

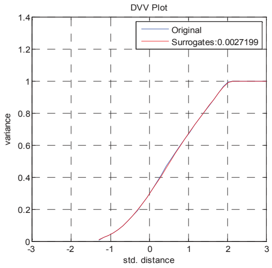

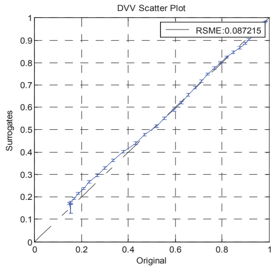

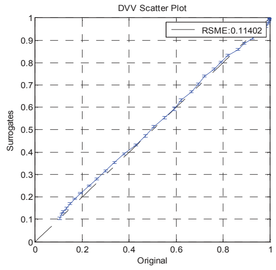

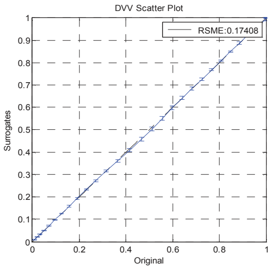

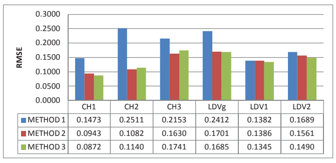

	CH1	CH2	CH3	LDVg	LDV1	LDV2
METHOD 1	0.1473	0.2511	0.2153	0.2412	0.1382	0.1689
METHOD 2	0.0943	0.1082	0.1630	0.1701	0.1386	0.1561
METHOD 3	0.0872	0.1140	0.1741	0.1685	0.1345	0.1490

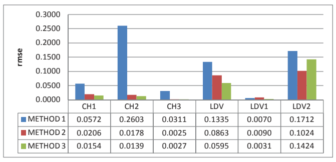

	CH1	CH2	CH3	LDV	LDV1	LDV2
METHOD 1	0.0572	0.2603	0.0311	0.1335	0.0070	0.1712
METHOD 2	0.0206	0.0178	0.0025	0.0863	0.0090	0.1024
METHOD 3	0.0154	0.0139	0.0027	0.0595	0.0031	0.1424

Data recorded LDV

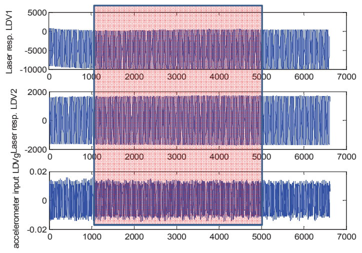

Data analysed LDV

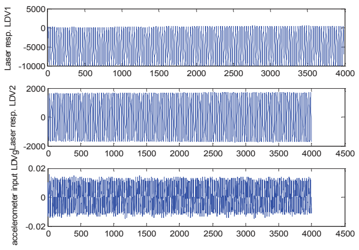

EXP 5

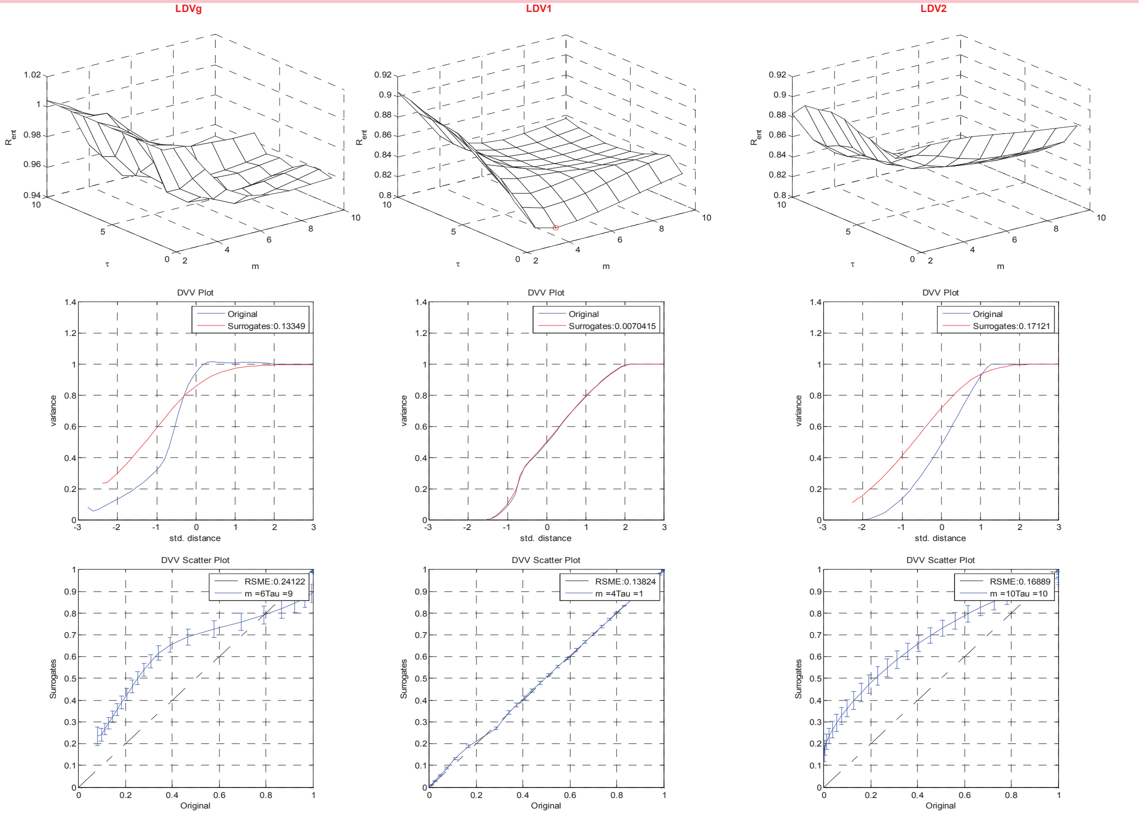

EXP 5

LDVg | LDV1 | LDV2

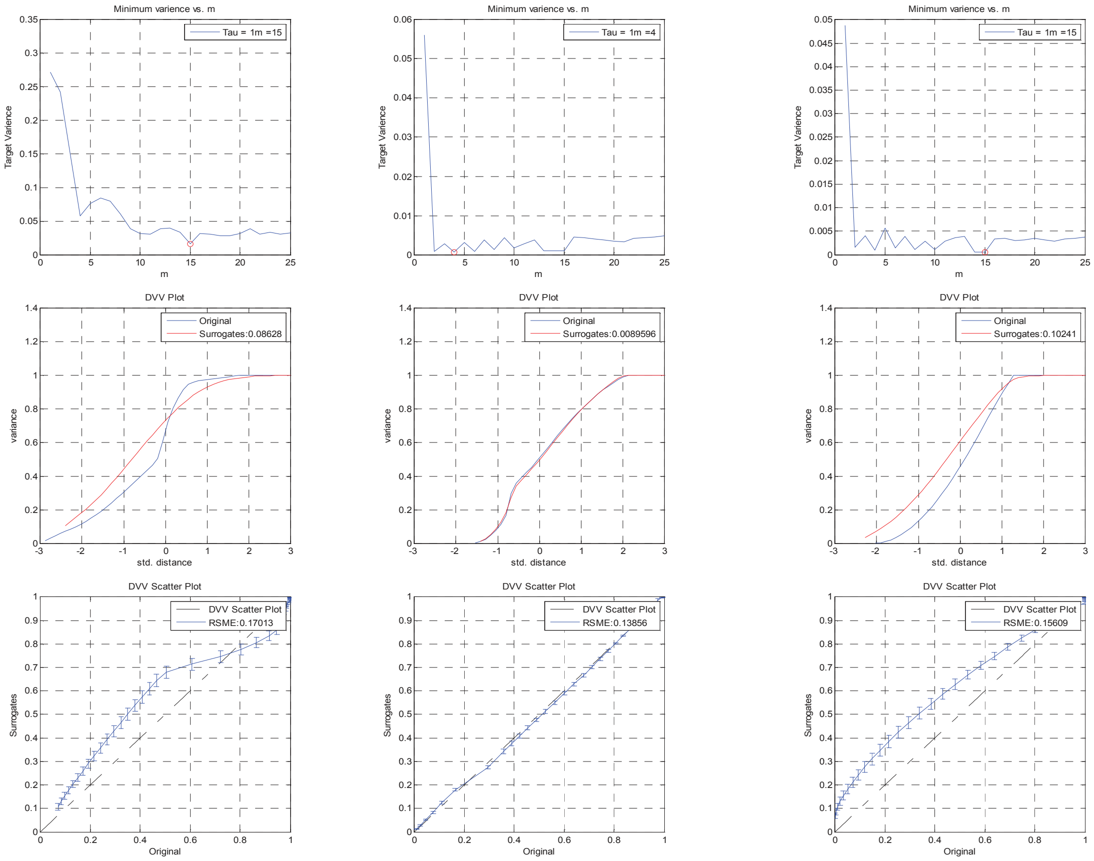

LDVg | LDV1 | LDV2

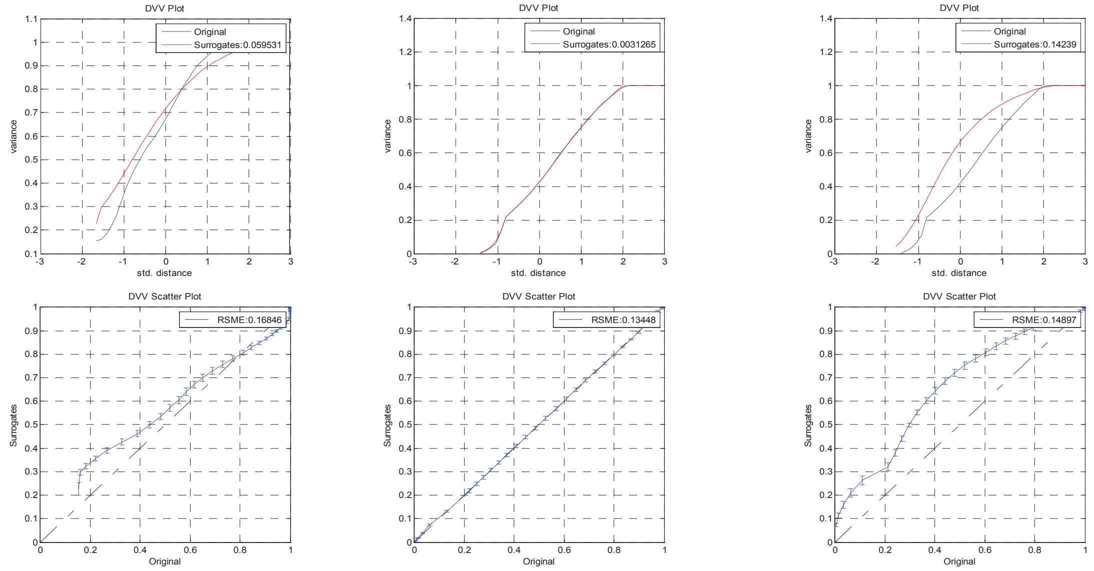

Data recorded Strain Gauge

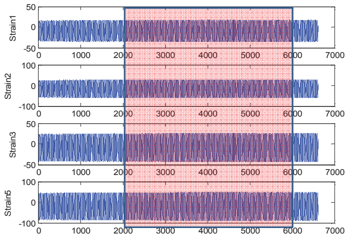

Data analysed Strain Gauge

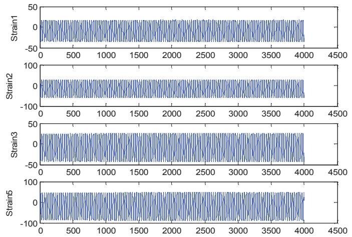

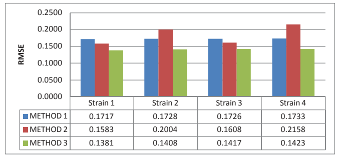

	Strain 1	Strain 2	Strain 3	Strain 4
METHOD 1	0.1717	0.1728	0.1726	0.1733
METHOD 2	0.1583	0.2004	0.1608	0.2158
METHOD 3	0.1381	0.1408	0.1417	0.1423

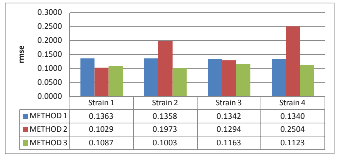

	Strain 1	Strain 2	Strain 3	Strain 4
METHOD 1	0.1363	0.1358	0.1342	0.1340
METHOD 2	0.1029	0.1973	0.1294	0.2504
METHOD 3	0.1087	0.1003	0.1163	0.1123

affected by change in ampliyude

EXP 5

STRAIN 1

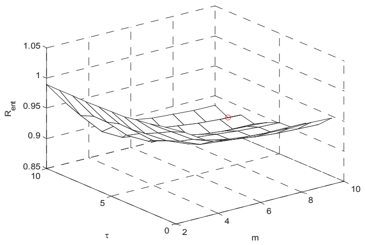

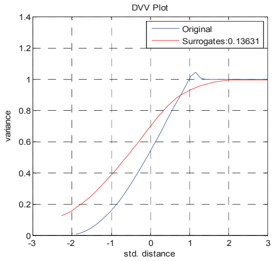

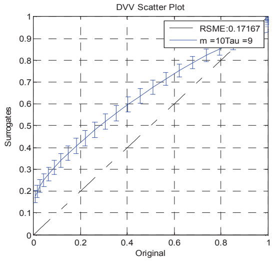

STRAIN 2

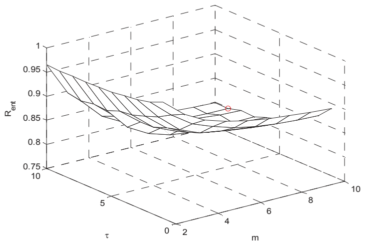

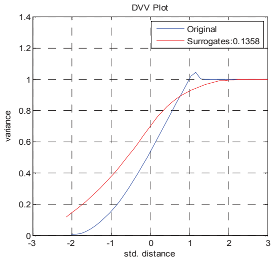

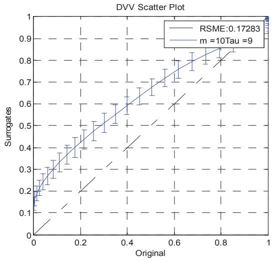

STRAIN 3

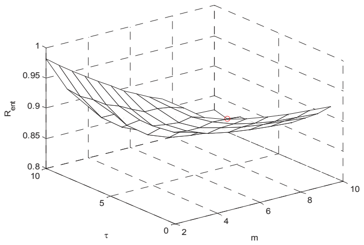

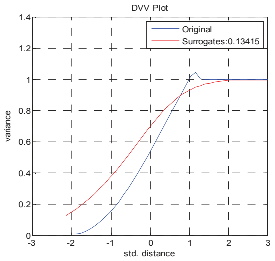

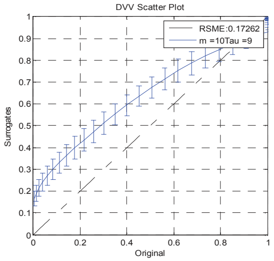

STRAIN 4

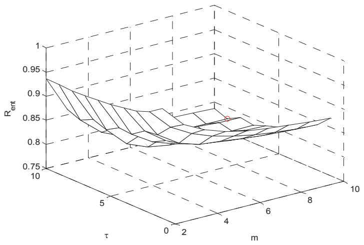

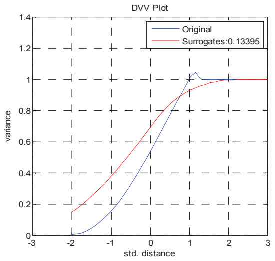

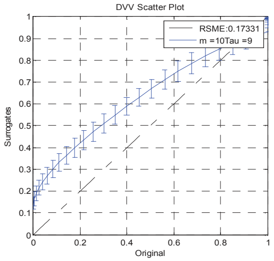

EXP 5

STRAIN 1

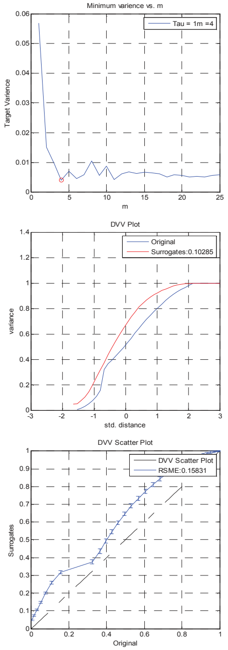

STRAIN 2

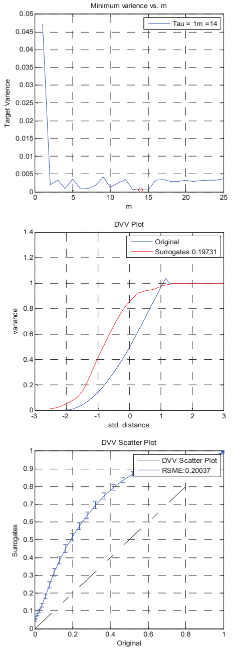

STRAIN 3

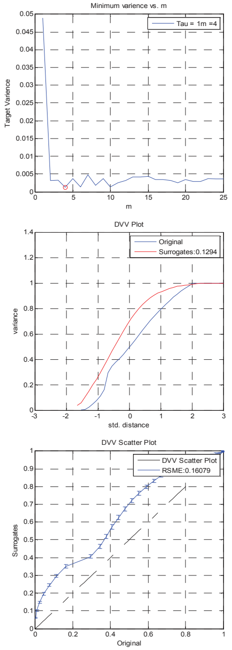

STRAIN 4

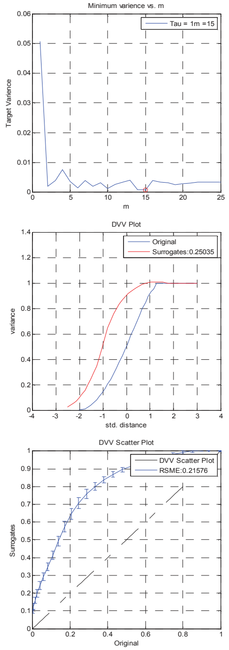

STRAIN 1

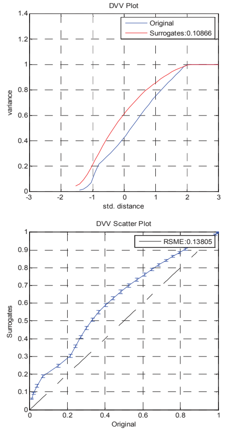

STRAIN 2

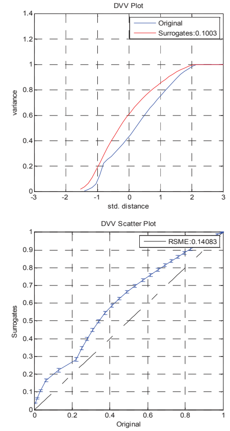

STRAIN 3

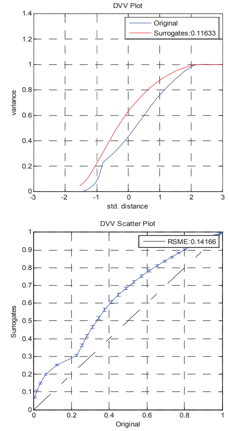

STRAIN 4

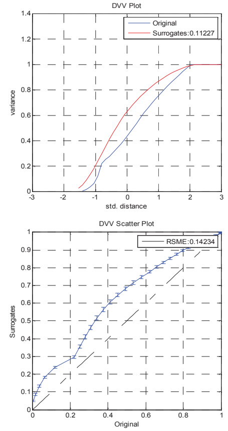

EXPERIMENT	VARIABLES		METHOD 1				METHOD 2				METHOD 3			
			best **m**	best **τ**	rsme	RSME	calc **m**	**τ**	rsme	RSME	set **m**	set **τ**	rsme	RSME
6	Harmonic resonance<br>2.0 Hz 2.5 Hz<br>3.0 Hz 3.5 Hz<br>4.0 Hz 4.2 Hz<br>4.3 Hz 4.4 Hz<br>4.5 Hz 4.6 Hz<br>5.0Hz 5.5 Hz<br>6.0 Hz 6.5Hz<br>7.0Hz<br>Focus at accelerometer	CH1	7	10	0.2275	0.1270	5	1	0.0798	0.1487	3	1	0.0232	0.1595
		CH2	6	10	0.0394	0.1630	25	1	0.0428	0.1091	3	1	0.0170	0.0370
		CH3	5	10	0.0115	0.1307	13	1	0.0120	0.1562	3	1	0.0093	0.1984
		LDVg	9	9	0.2784	0.2324	23	1	0.1238	0.0992	3	1	0.0457	0.0861
		LDV1	6	10	0.1399	0.1412	8	1	0.0647	0.1352	3	1	0.0227	0.1566
		LDV2	10	10	0.3261	0.1723	4	1	0.2445	0.1365	3	1	0.2419	0.1441
		Strain 1	10	9	0.4033	0.2160	8	1	0.2391	0.1495	3	1	0.2333	0.1283
		Strain 2	9	7	0.2962	0.1651	21	1	0.2939	0.1699	3	1	0.2085	0.1367
		Strain 3	8	7	0.3948	0.2094	20	1	0.4380	0.2272	3	1	0.2630	0.1443
		Strain 4	8	7	0.3774	0.1991	5	1	0.2741	0.1440	3	1	0.2032	0.1226

Data recorded 3D Accelerometer

*Optimum embeding parameter m and time lag tau are determined as per EXP6b interval

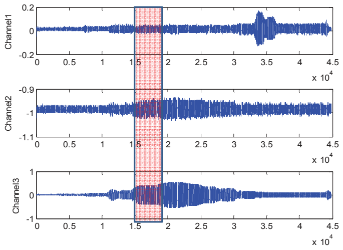

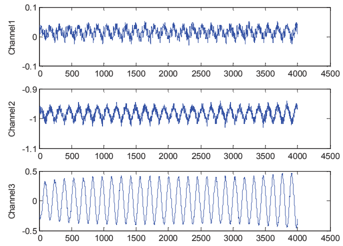

METHOD 1

CH1

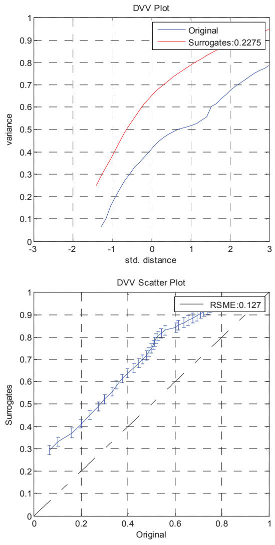

CH2

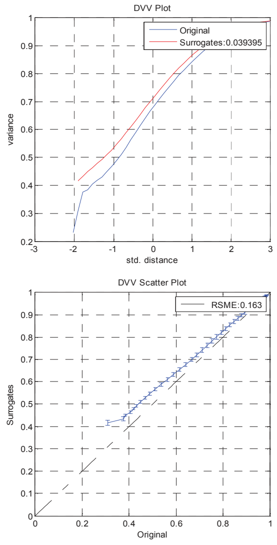

CH3

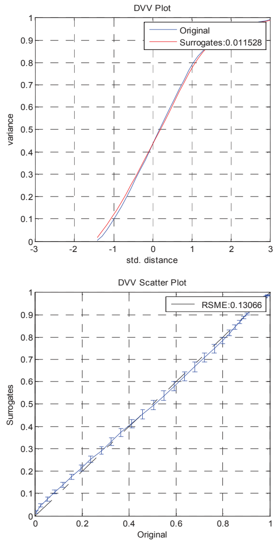

EXP 6 METHOD 2

CH1 CH2 CH3

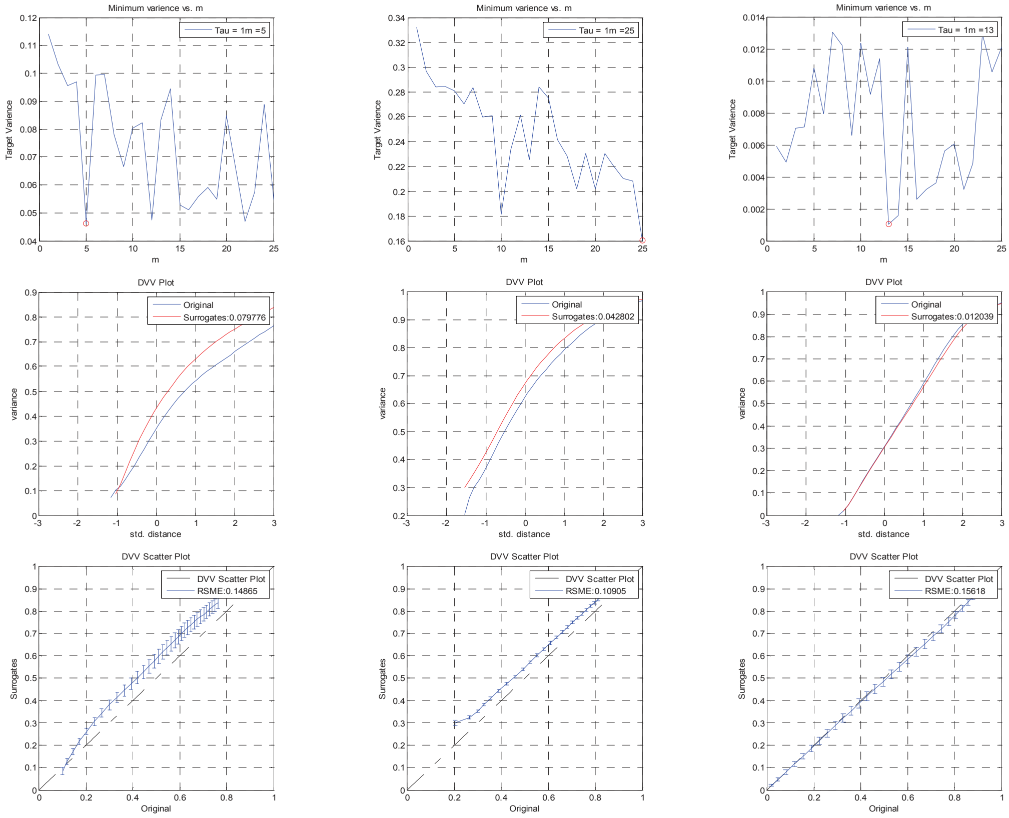

METHOD 3

CH1 CH2 CH3

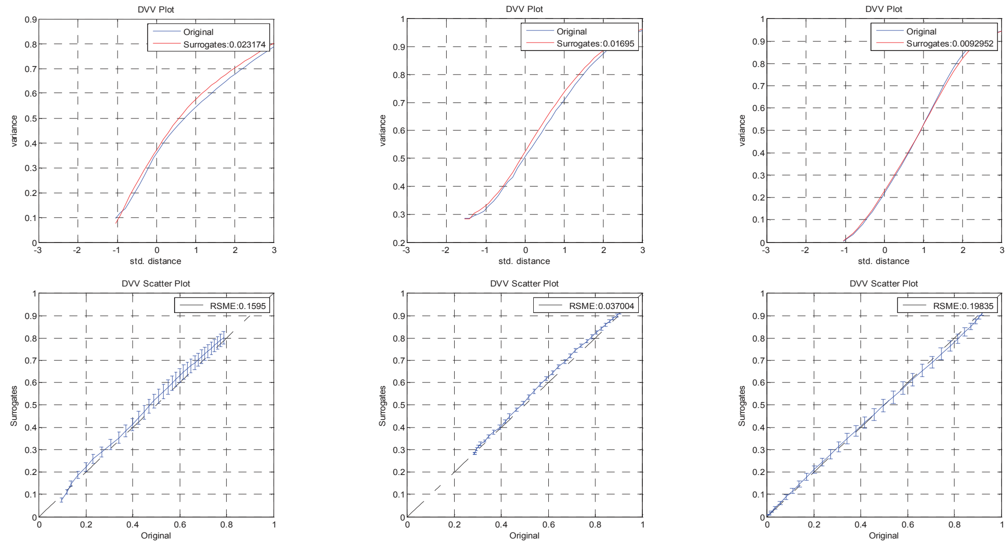

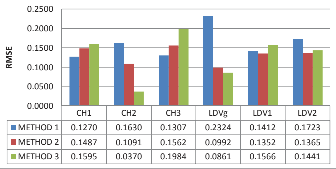

	CH1	CH2	CH3	LDVg	LDV1	LDV2
METHOD 1	0.1270	0.1630	0.1307	0.2324	0.1412	0.1723
METHOD 2	0.1487	0.1091	0.1562	0.0992	0.1352	0.1365
METHOD 3	0.1595	0.0370	0.1984	0.0861	0.1566	0.1441

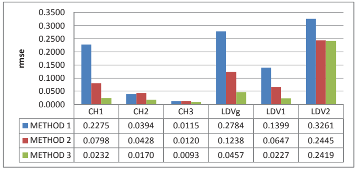

	CH1	CH2	CH3	LDVg	LDV1	LDV2
METHOD 1	0.2275	0.0394	0.0115	0.2784	0.1399	0.3261
METHOD 2	0.0798	0.0428	0.0120	0.1238	0.0647	0.2445
METHOD 3	0.0232	0.0170	0.0093	0.0457	0.0227	0.2419

Data recorded LDV

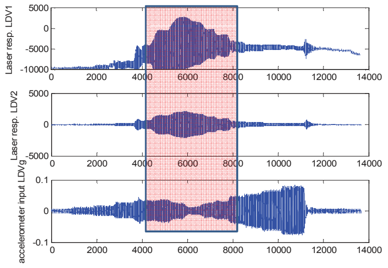

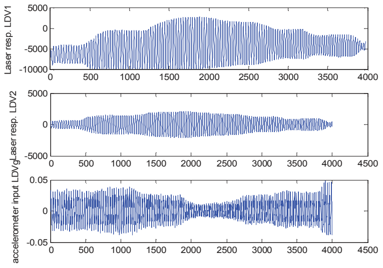

EXP 6

LDVg

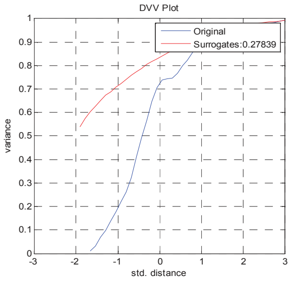

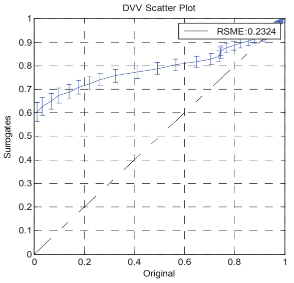

LDV1

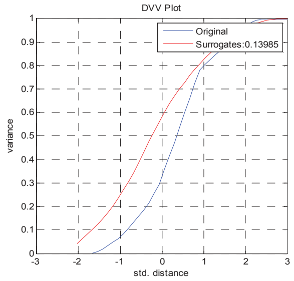

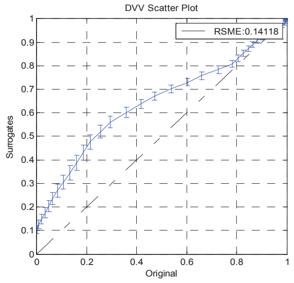

LDV2

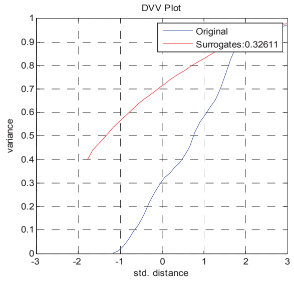

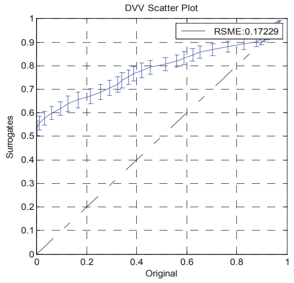

EXP 6

LDVg

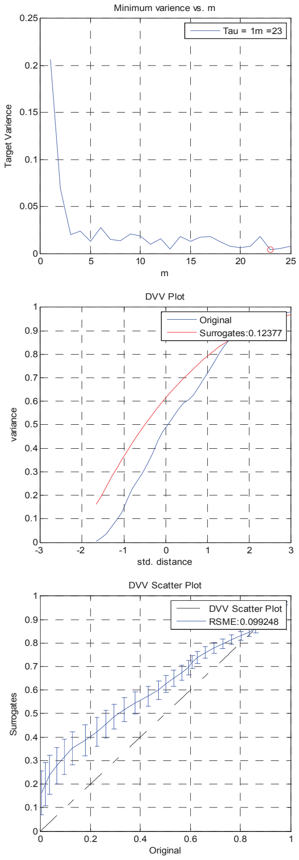

LDV1

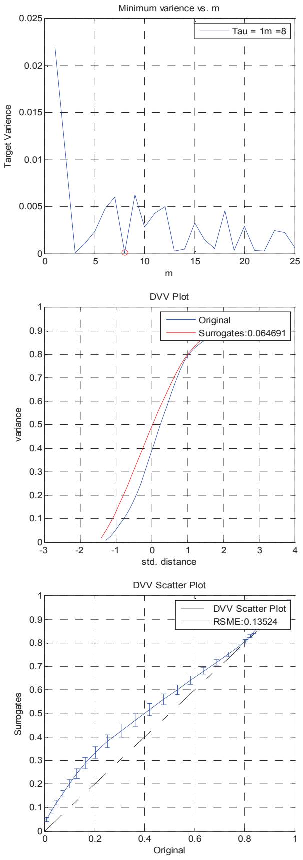

LDV2

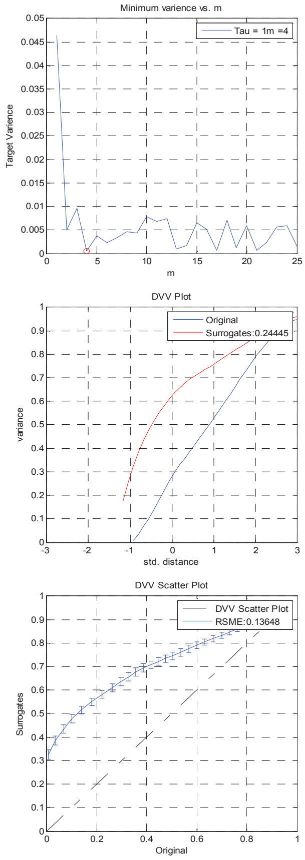

LDVg

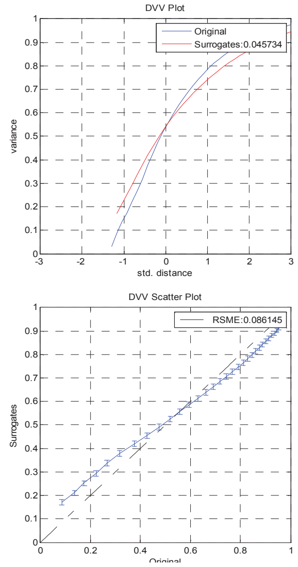

LDV1

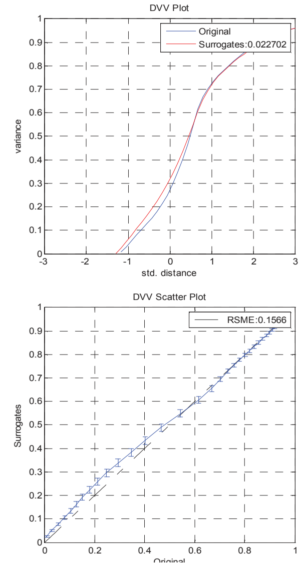

LDV2

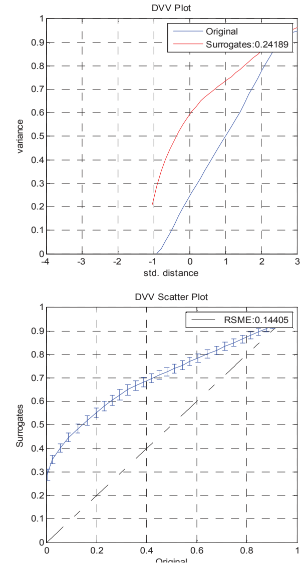

Data recorded Strain Gauge

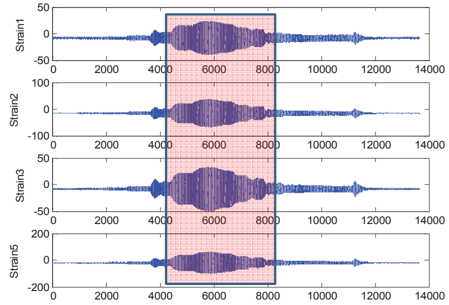

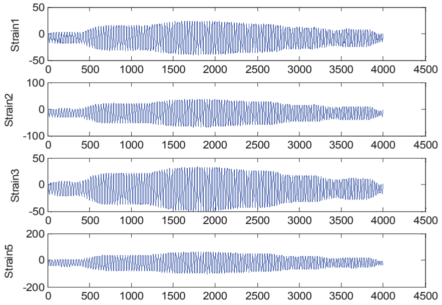

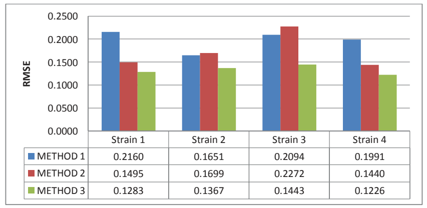

	Strain 1	Strain 2	Strain 3	Strain 4
METHOD 1	0.2160	0.1651	0.2094	0.1991
METHOD 2	0.1495	0.1699	0.2272	0.1440
METHOD 3	0.1283	0.1367	0.1443	0.1226

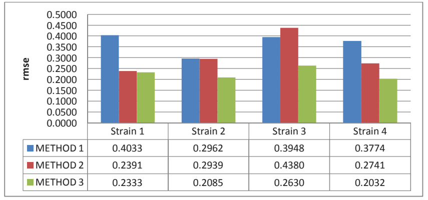

	Strain 1	Strain 2	Strain 3	Strain 4
METHOD 1	0.4033	0.2962	0.3948	0.3774
METHOD 2	0.2391	0.2939	0.4380	0.2741
METHOD 3	0.2333	0.2085	0.2630	0.2032

EXP 6

STRAIN 1

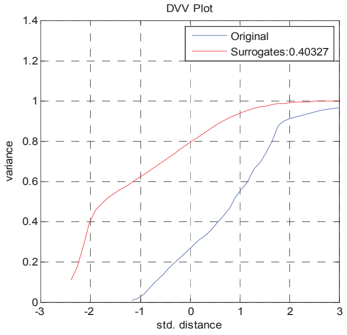

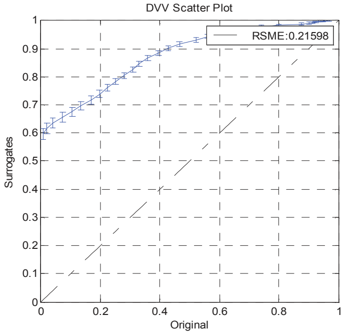

STRAIN 2

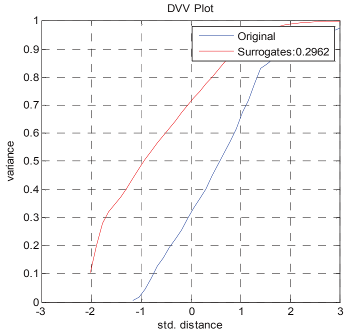

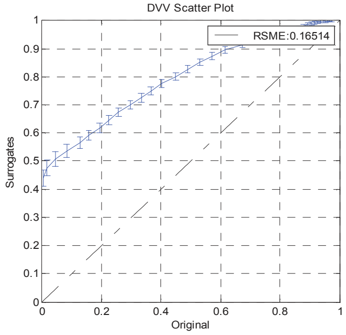

STRAIN 3

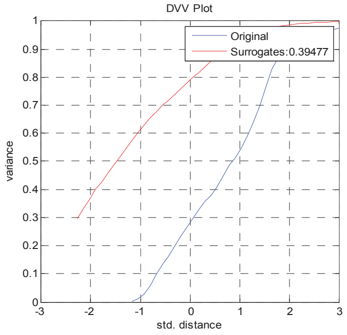

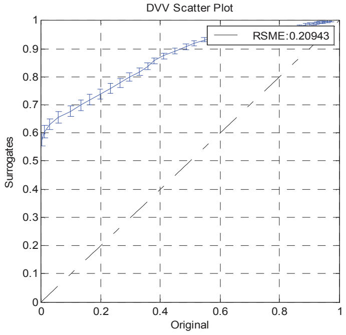

STRAIN 4

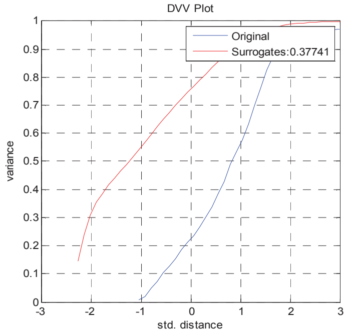

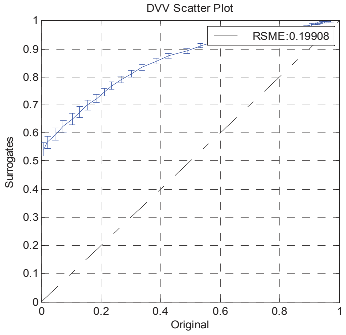

EXP 6

STRAIN 1

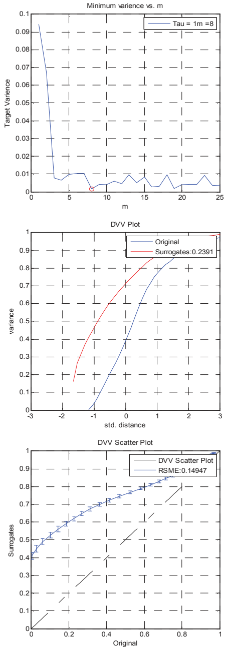

STRAIN 2

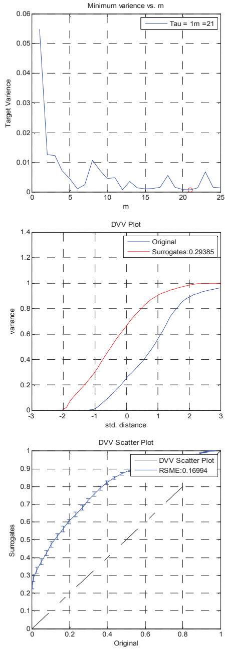

STRAIN 3

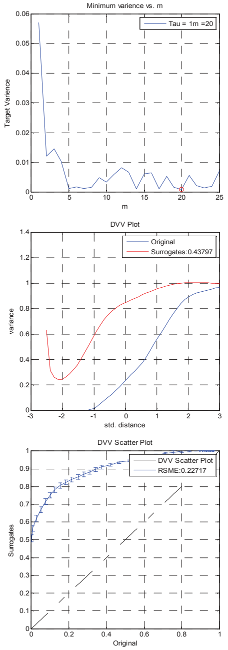

STRAIN 4

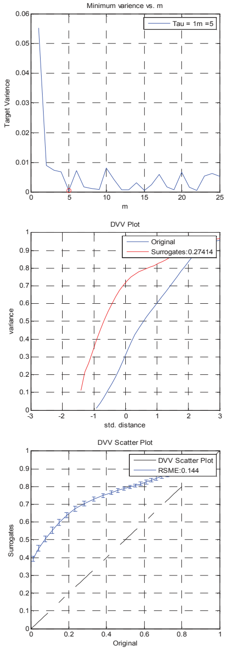

STRAIN 1

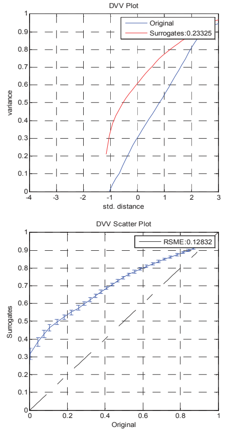

STRAIN 2

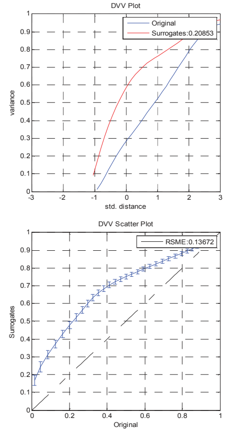

STRAIN 3

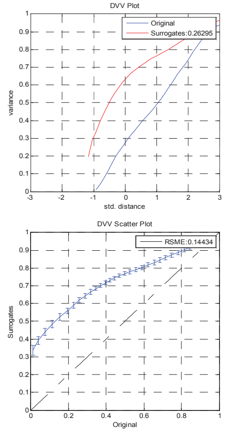

STRAIN 4

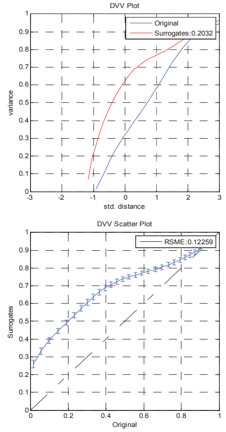

EXPERIMENT	VARIABLES		METHOD 1				METHOD 2				METHOD 3			
			best **m**	best **τ**	rsme	RSME	calc **m**	**τ**	rsme	RSME	set **m**	set **τ**	rsme	RSME
7	Loading Sine sweep<br>2.0 Hz 6.0 Hz<br>60 sec<br>Focus at accelerometer	CH1	4	10	0.0464	0.2276	11	1	0.0367	0.2039	3	1	0.0600	0.3182
		CH2	2	6	0.0578	0.2472	19	1	0.0725	0.1439	3	1	0.0688	0.1016
		CH3	4	9	0.0413	0.1323	25	1	0.0384	0.1946	3	1	0.0041	0.2724
		LDVg	10	2	0.3604	0.2572	12	1	0.1016	0.1091	3	1	0.0431	0.1088
		LDV1	6	6	0.3849	0.2246	10	1	0.0405	0.1303	3	1	0.0128	0.1705
		LDV2	8	10	0.0475	0.1453	12	1	0.3114	0.1656	3	1	0.2466	0.1656
		Strain 1	10	10	0.1344	0.1098	7	1	0.2157	0.1395	3	1	0.2384	0.1329
		Strain 2	10	9	0.1039	0.1309	18	1	0.2502	0.1598	3	1	0.1746	0.1363
		Strain 3	10	9	0.3815	0.2012	19	1	0.3918	0.2048	3	1	0.2432	0.1374
		Strain 4	10	9	0.3811	0.1996	23	1	0.3339	0.1717	3	1	0.2277	0.1420

METHOD 1 for data section			
best **m**	best **τ**	rsme	RSME
4	10	0.0063	0.1050
2	6	0.0197	0.1682
4	9	0.0260	0.1286

Data recorded 3D Accelerometer

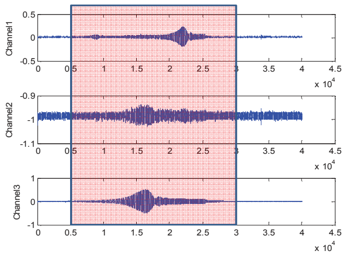

Data analysed 3D Accelerometer

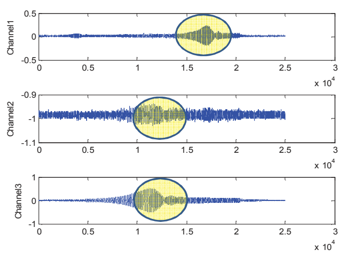

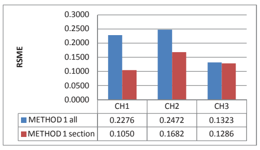

METHOD 1

CH1

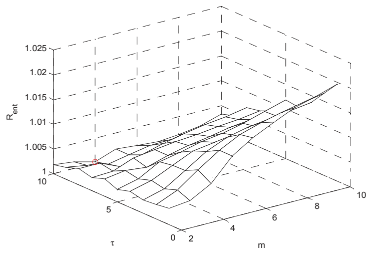

CH2

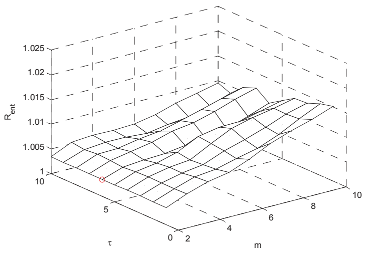

CH3

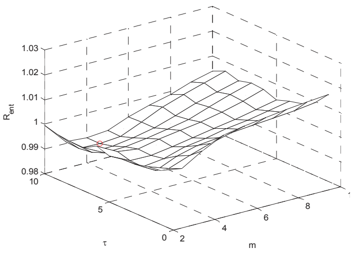

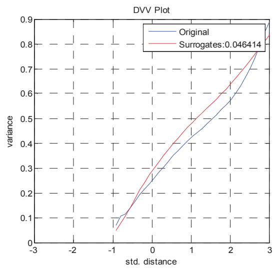

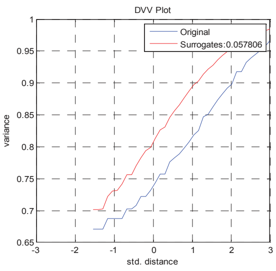

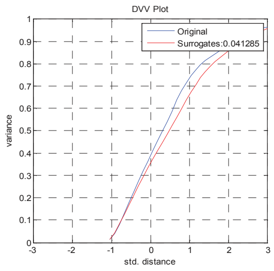

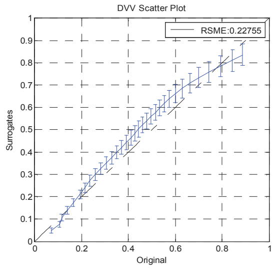

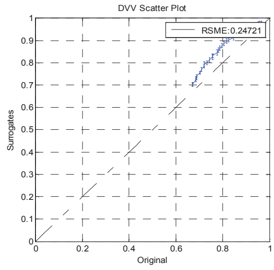

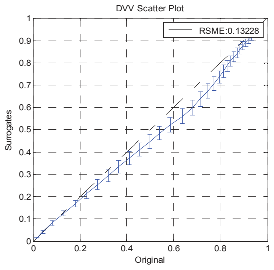

METHOD 2

CH1

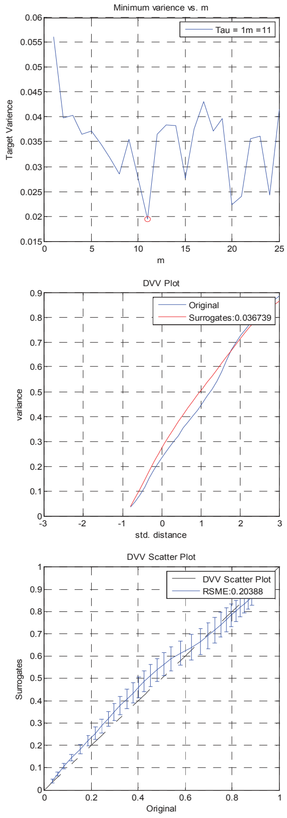

CH2

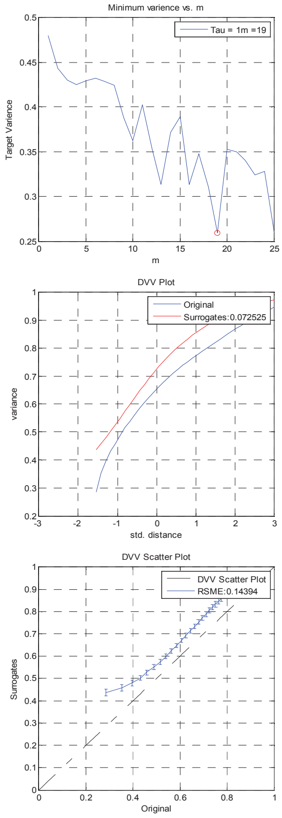

CH3

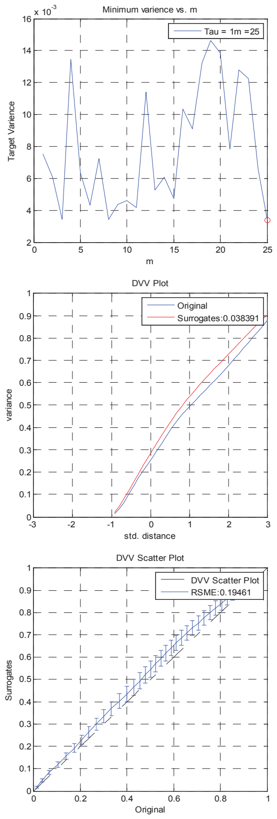

METHOD 3

CH1

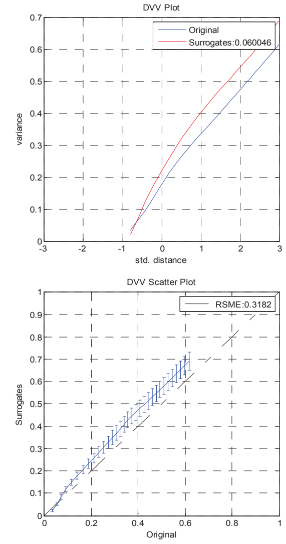

CH2

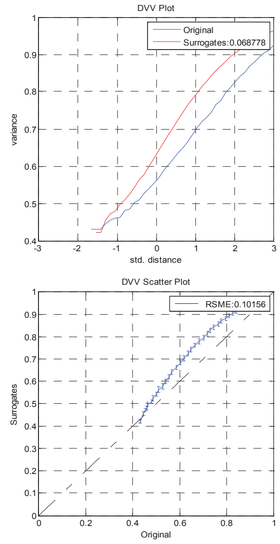

CH3

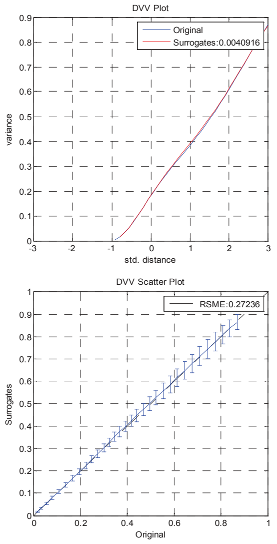

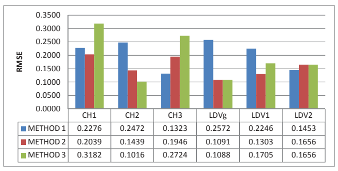

	CH1	CH2	CH3	LDVg	LDV1	LDV2
METHOD 1	0.2276	0.2472	0.1323	0.2572	0.2246	0.1453
METHOD 2	0.2039	0.1439	0.1946	0.1091	0.1303	0.1656
METHOD 3	0.3182	0.1016	0.2724	0.1088	0.1705	0.1656

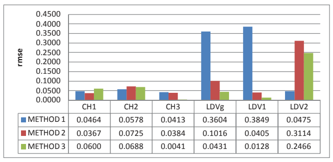

	CH1	CH2	CH3	LDVg	LDV1	LDV2
METHOD 1	0.0464	0.0578	0.0413	0.3604	0.3849	0.0475
METHOD 2	0.0367	0.0725	0.0384	0.1016	0.0405	0.3114
METHOD 3	0.0600	0.0688	0.0041	0.0431	0.0128	0.2466

Data recorded LDV

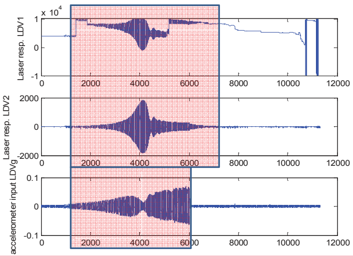

Data analysed LDV

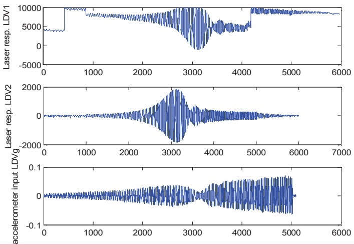

LDVg LDV1 LDV2

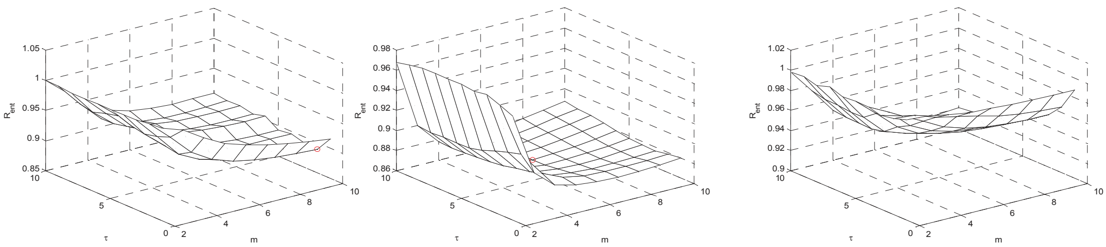

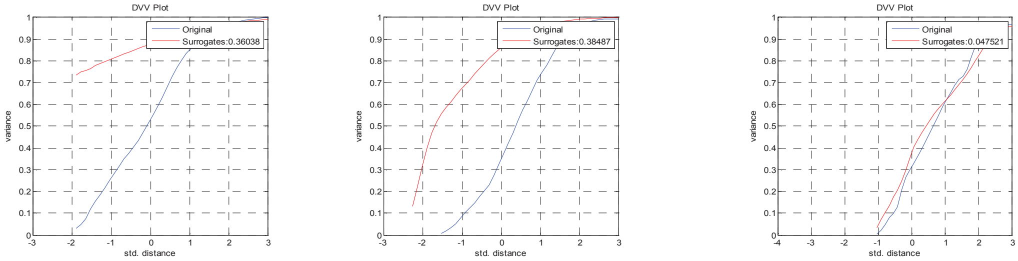

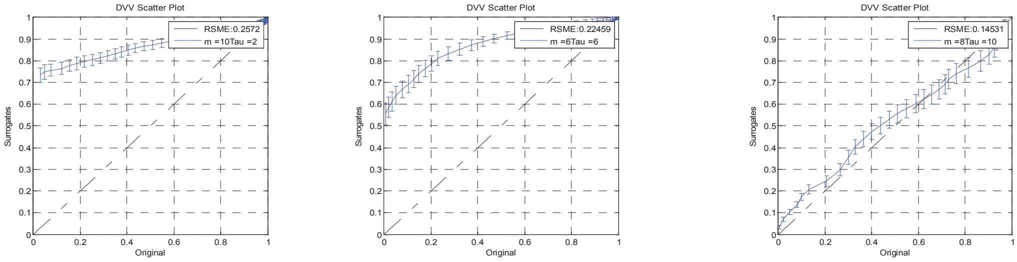

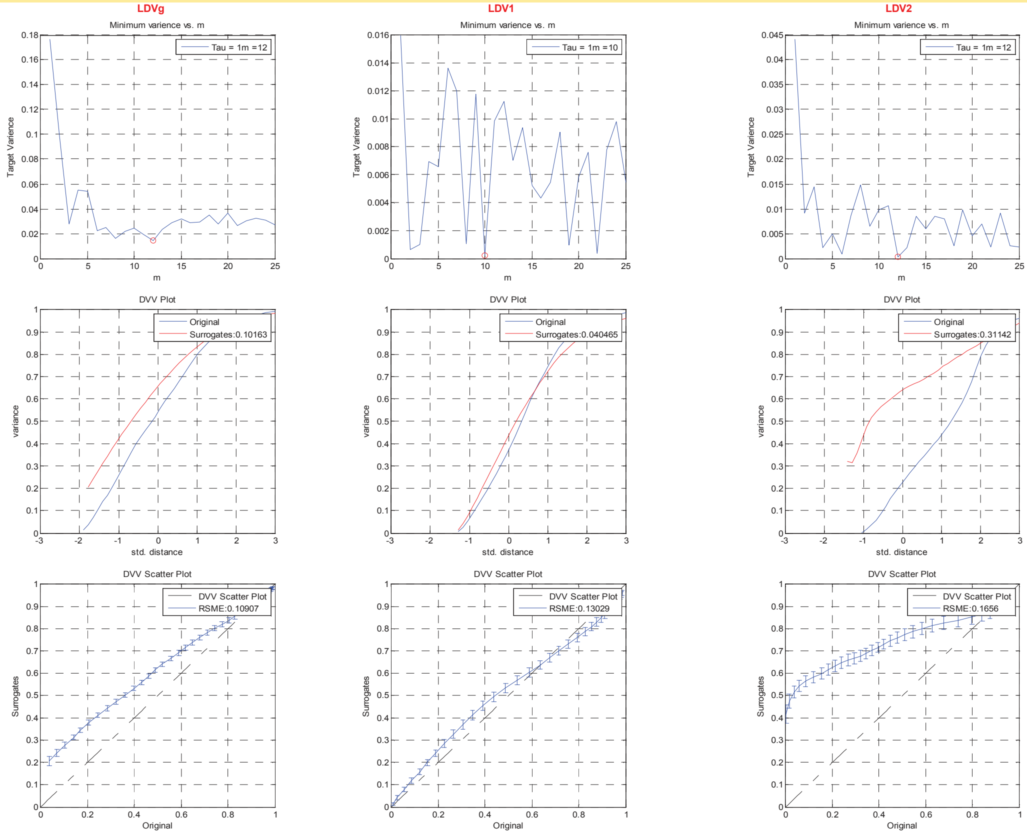
LDVg
LDV1
LDV2
Minimum varience vs. m
Tau = 1m =12
Tau = 1m =10
Tau = 1m =12
Target Varience
m
DVV Plot
Original
Surrogates:0.10163
Surrogates:0.040465
Surrogates:0.31142
variance
std. distance
DVV Scatter Plot
RSME:0.10907
RSME:0.13029
RSME:0.1656
Surrogates
Original

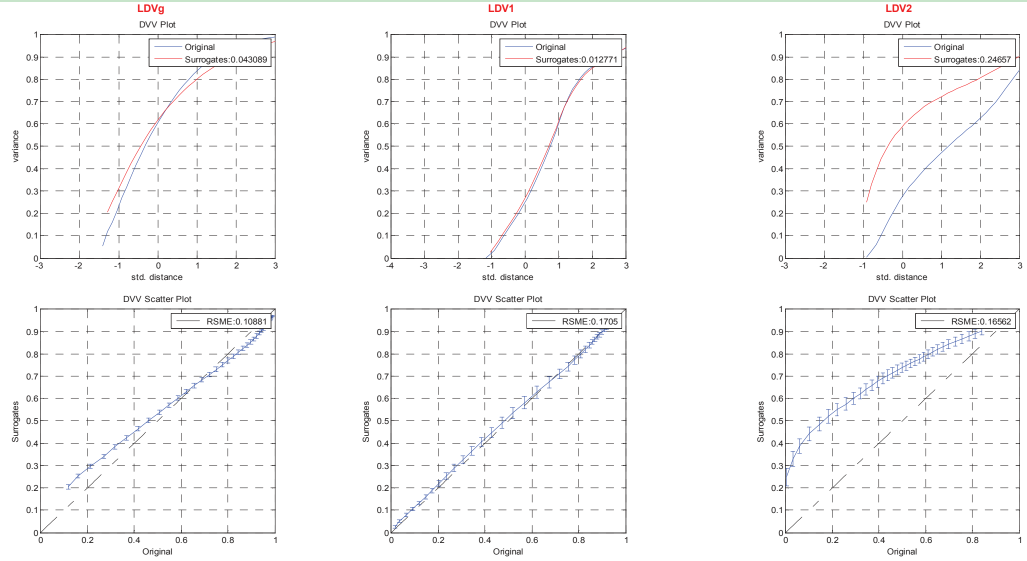
LDVg
LDV1
LDV2
DVV Plot
Original
Surrogates:0.043089
Surrogates:0.012771
Surrogates:0.24657
variance
std. distance
DVV Scatter Plot
RSME:0.10881
RSME:0.1705
RSME:0.16562
Surrogates
Original

Data recorded Strain Gauge

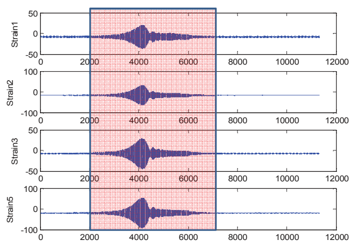

Data analysed Strain Gauge

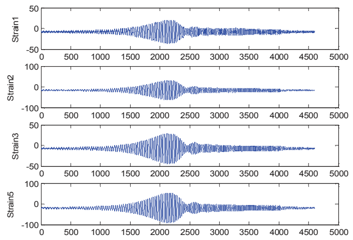

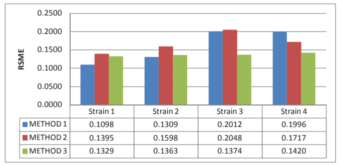

	Strain 1	Strain 2	Strain 3	Strain 4
METHOD 1	0.1098	0.1309	0.2012	0.1996
METHOD 2	0.1395	0.1598	0.2048	0.1717
METHOD 3	0.1329	0.1363	0.1374	0.1420

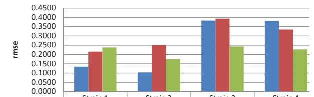

	Strain 1	Strain 2	Strain 3	Strain 4
METHOD 1	0.1344	0.1039	0.3815	0.3811
METHOD 2	0.2157	0.2502	0.3918	0.3339
METHOD 3	0.2384	0.1746	0.2432	0.2277

STRAIN 1

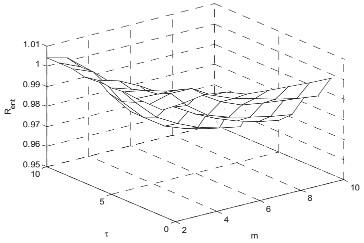

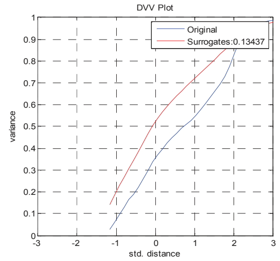

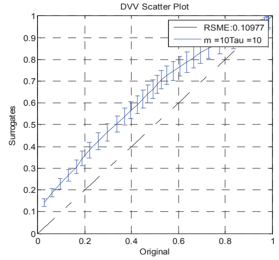

STRAIN 2

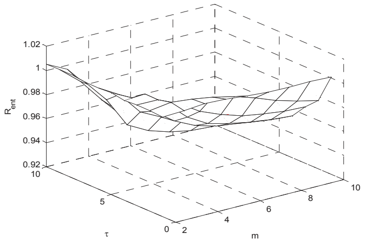

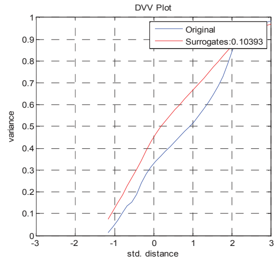

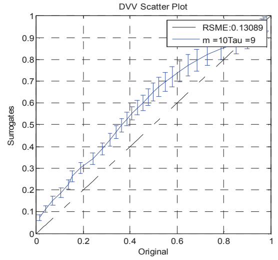

STRAIN 3

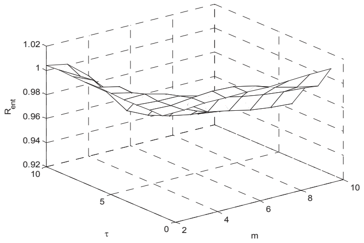

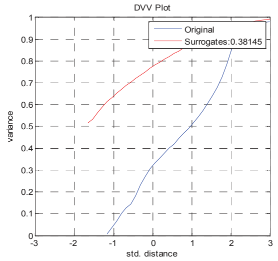

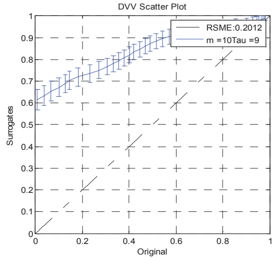

STRAIN 4

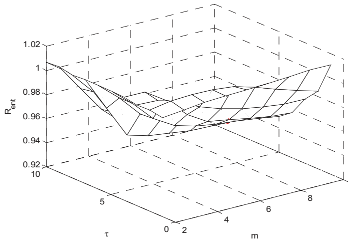

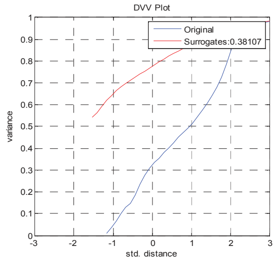

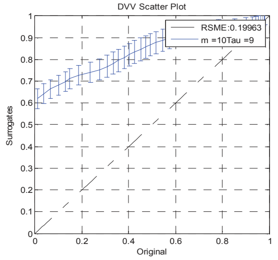

STRAIN 1

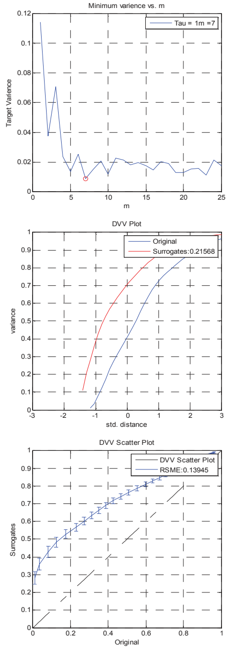

STRAIN 2

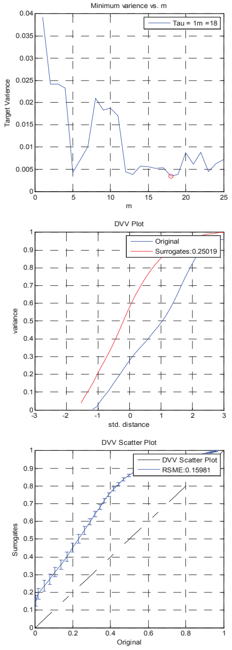

STRAIN 3

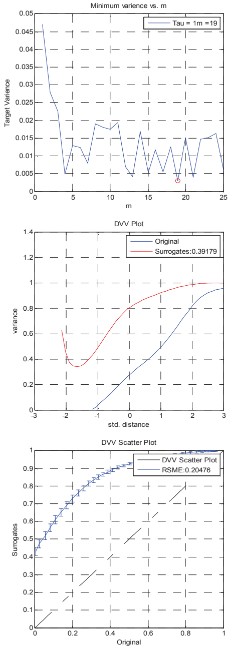

STRAIN 4

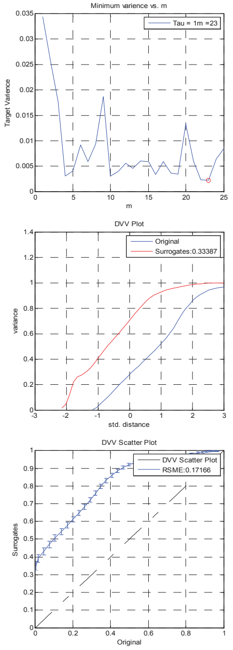

STRAIN 1

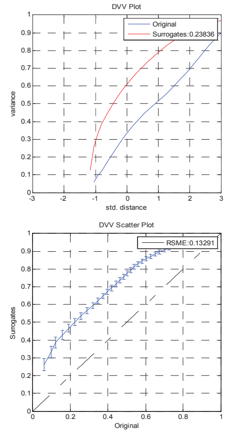

STRAIN 2

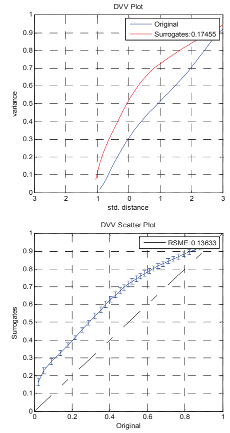

STRAIN 3

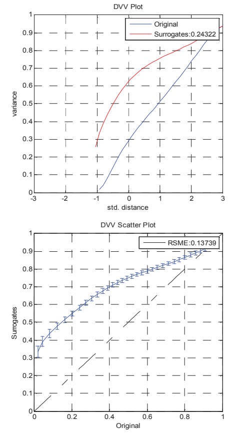

STRAIN 4

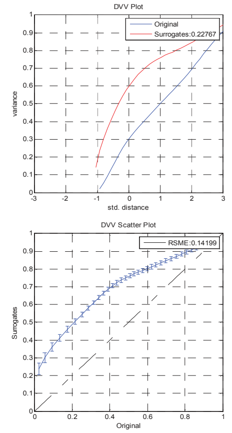

EXPERIMENT	VARIABLES		METHOD 1				METHOD 2				METHOD 3			
			best **m**	best **τ**	rsme	RSME	calc **m**	**τ**	rsme	RSME	set **m**	set **τ**	rsme	RSME
8	Loading White Noise Focus at accelerometer	CH1	4	1	0.0036	0.1327	10	1	0.0160	0.1327	3	1	0.0104	0.0686
		CH2	5	1	0.0435	0.1612	19	1	0.0804	0.1612	3	1	0.0347	0.1264
		CH3	3	1	0.0224	0.1911	16	1	0.0383	0.1911	3	1	0.0168	0.0917
		LDVg	4	1	0.2189	0.3882	19	1	0.0113	0.3882	3	1	0.0444	0.2542
		LDV1	6	10	0.1990	0.1337	12	1	0.0274	0.1337	3	1	0.0057	0.1268
		LDV2	9	10	0.2077	0.2399	14	1	0.3092	0.2399	3	1	0.2351	0.2062
		Strain 1	7	7	0.2163	0.3613	22	1	0.3935	0.3613	3	1	0.2497	0.3134
		Strain 2	7	7	0.1296	0.2427	24	1	0.2236	0.2427	3	1	0.0852	0.2064
		Strain 3	10	6	0.3315	0.1646	17	1	0.1921	0.1646	3	1	0.1455	0.1260
		Strain 4	10	10	0.3392	0.1124	16	1	0.0946	0.1124	3	1	0.0854	0.0854

METHOD 1 for data section			
best **m**	best **τ**	rsme	RSME
4	1	0.0054	0.0738
5	1	0.2186	0.2704
3	1	0.0138	0.0827

Data recorded 3D Accelerometer

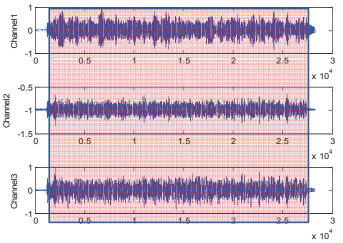

Data analysed 3D Accelerometer

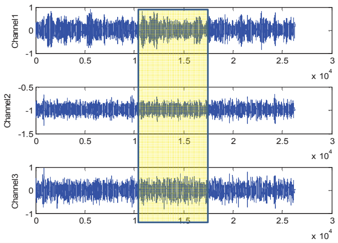

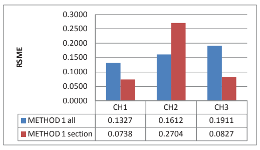

METHOD 1

CH1

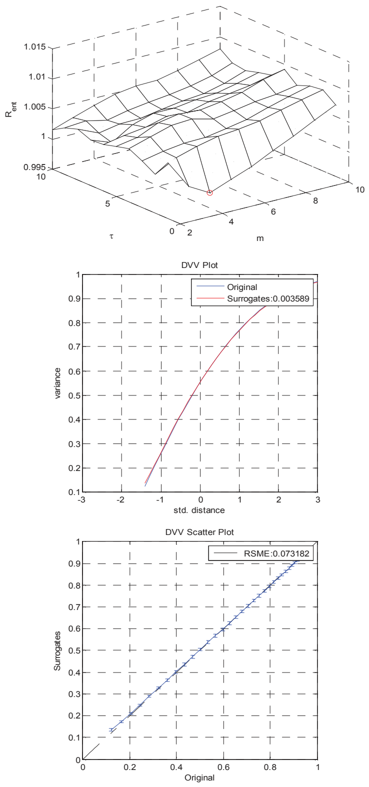

CH2

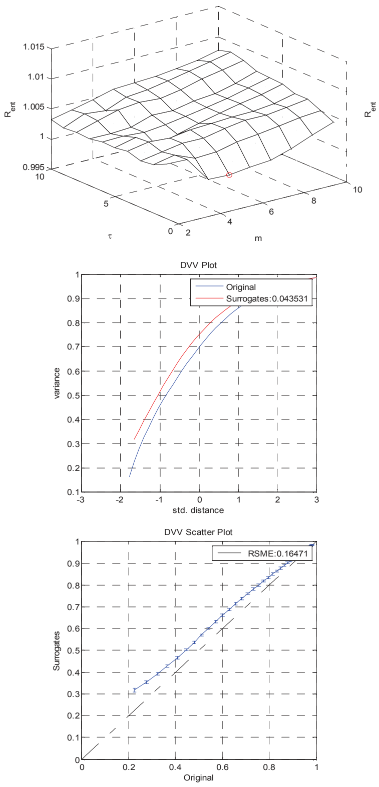

CH3

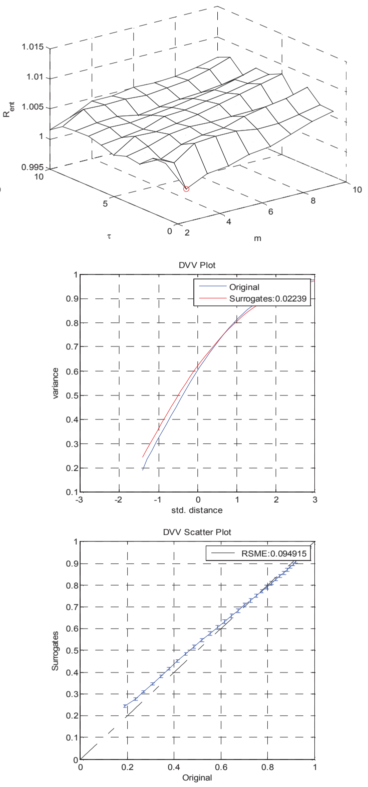

EXP 8 METHOD 2

CH1 **CH2** **CH3**

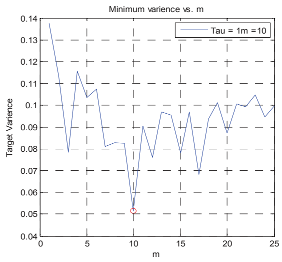

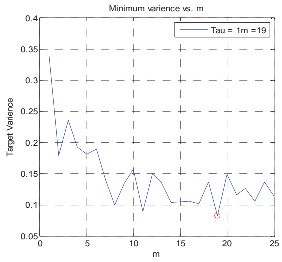

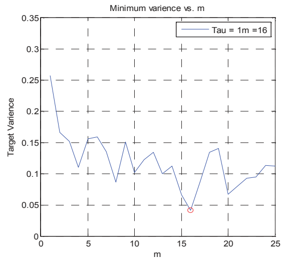

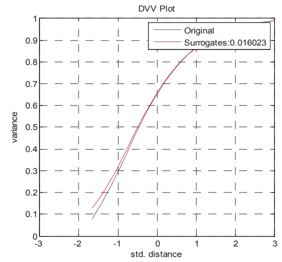

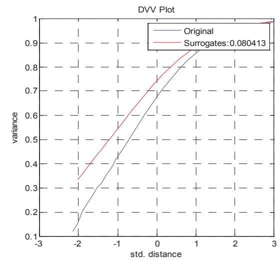

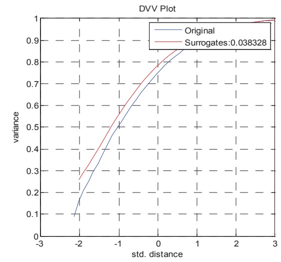

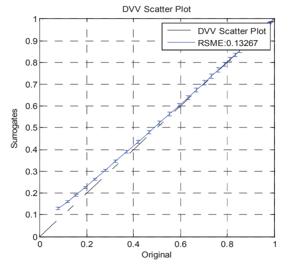

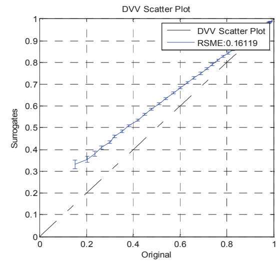

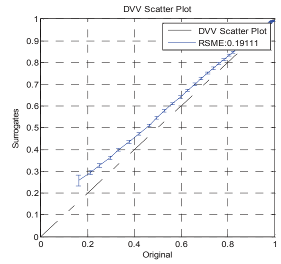

METHOD 3

CH1 **CH2** **CH3**

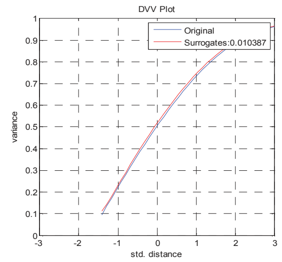

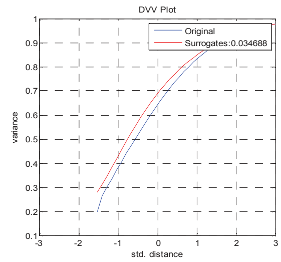

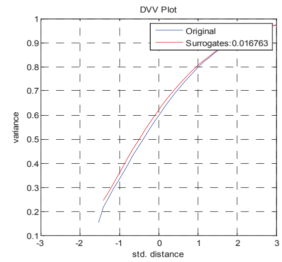

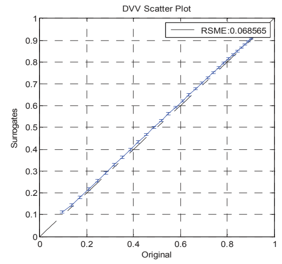

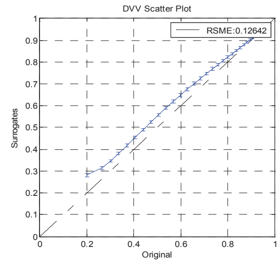

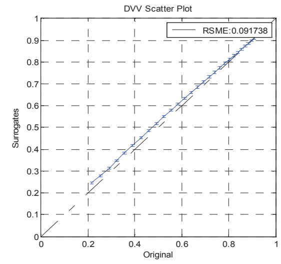

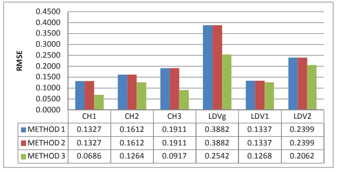

	CH1	CH2	CH3	LDVg	LDV1	LDV2
METHOD 1	0.1327	0.1612	0.1911	0.3882	0.1337	0.2399
METHOD 2	0.1327	0.1612	0.1911	0.3882	0.1337	0.2399
METHOD 3	0.0686	0.1264	0.0917	0.2542	0.1268	0.2062

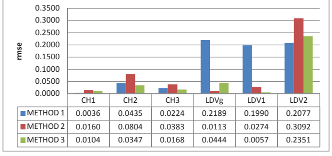

	CH1	CH2	CH3	LDVg	LDV1	LDV2
METHOD 1	0.0036	0.0435	0.0224	0.2189	0.1990	0.2077
METHOD 2	0.0160	0.0804	0.0383	0.0113	0.0274	0.3092
METHOD 3	0.0104	0.0347	0.0168	0.0444	0.0057	0.2351

Data recorded LDV

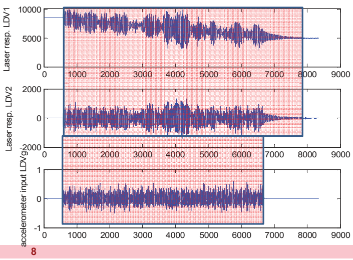

Data analysed LDV

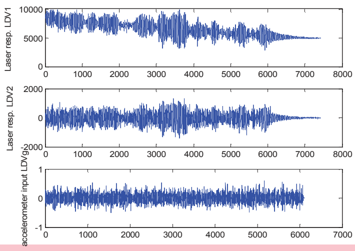

EXP 8

LDVg

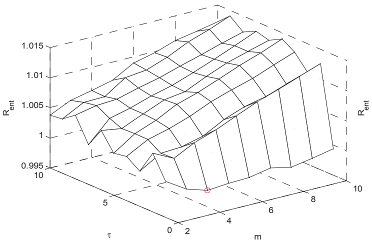

LDV1

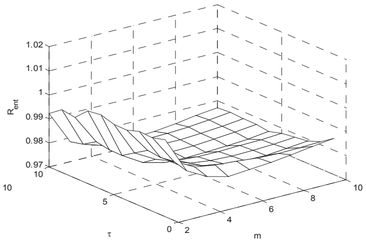

LDV2

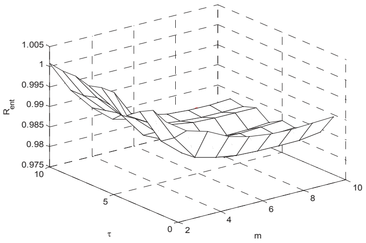

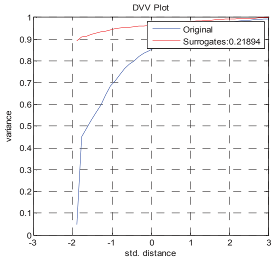

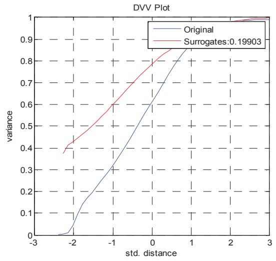

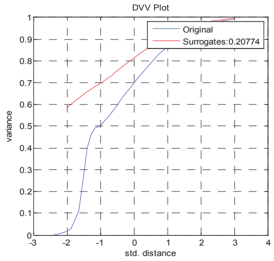

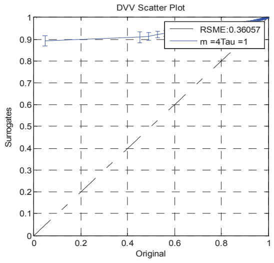

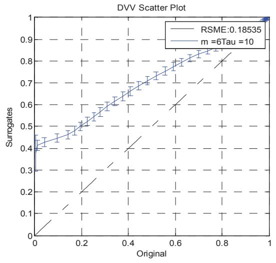

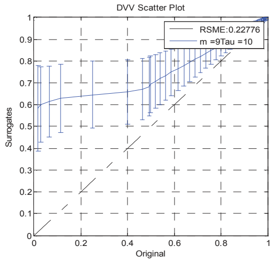

EXP 8

LDVg

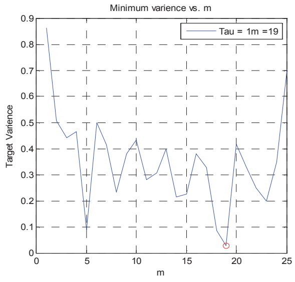

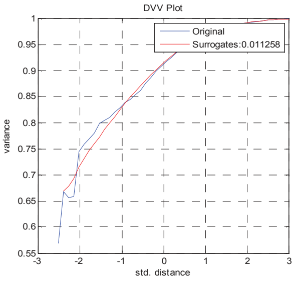

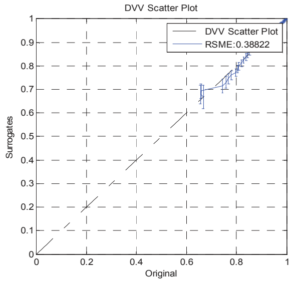

LDV1

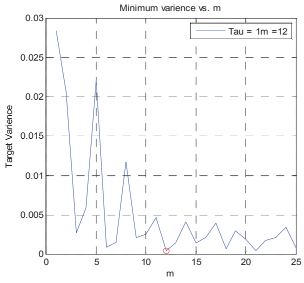

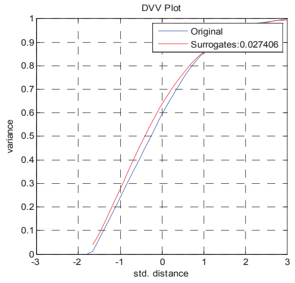

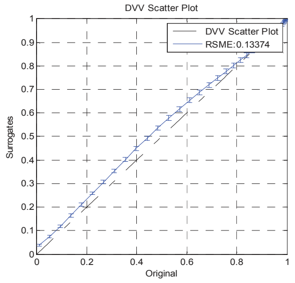

LDV2

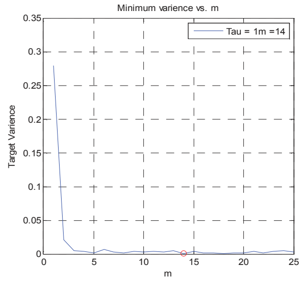

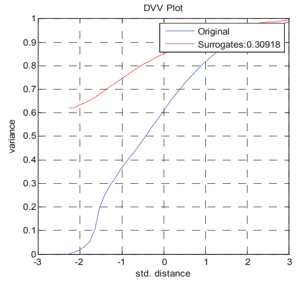

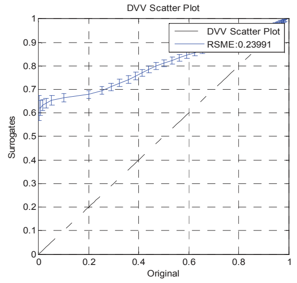

LDVg

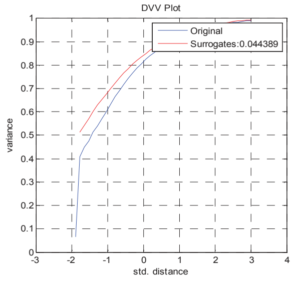

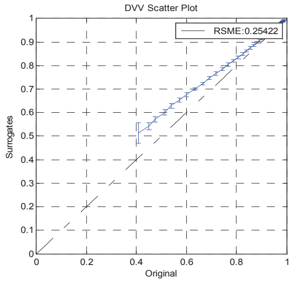

LDV1

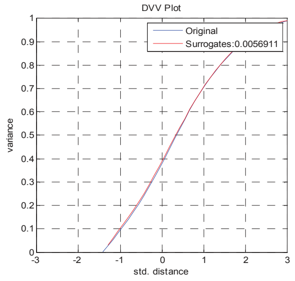

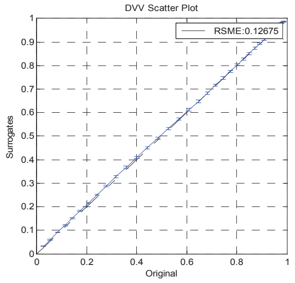

LDV2

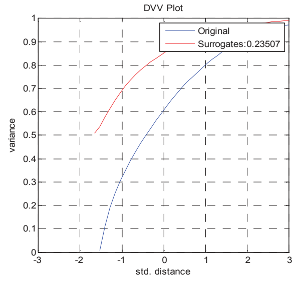

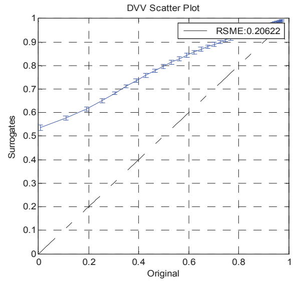

Data recorded Strain Gauge

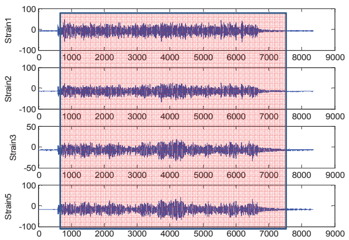

Data analysed Strain Gauge

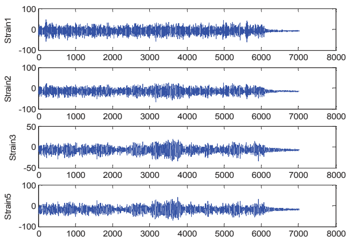

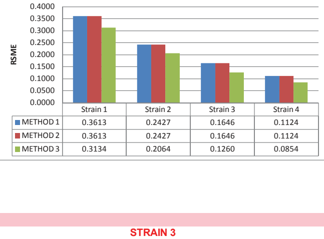

	Strain 1	Strain 2	Strain 3	Strain 4
METHOD 1	0.3613	0.2427	0.1646	0.1124
METHOD 2	0.3613	0.2427	0.1646	0.1124
METHOD 3	0.3134	0.2064	0.1260	0.0854

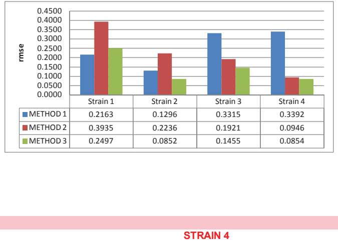

	Strain 1	Strain 2	Strain 3	Strain 4
METHOD 1	0.2163	0.1296	0.3315	0.3392
METHOD 2	0.3935	0.2236	0.1921	0.0946
METHOD 3	0.2497	0.0852	0.1455	0.0854

EXP 8

STRAIN 1

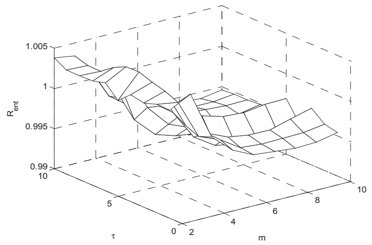

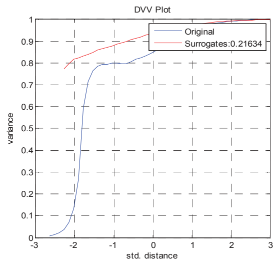

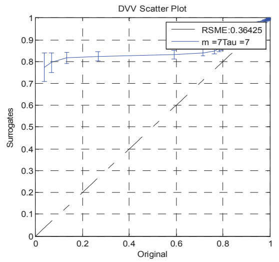

STRAIN 2

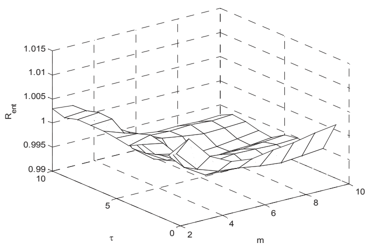

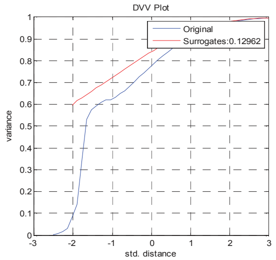

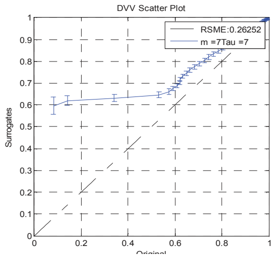

STRAIN 3

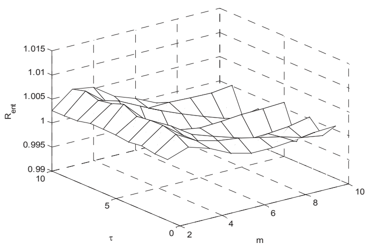

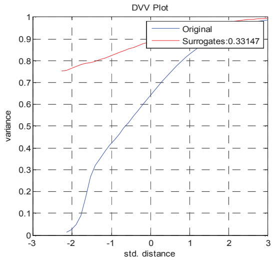

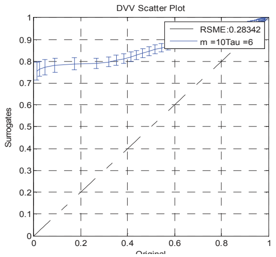

STRAIN 4

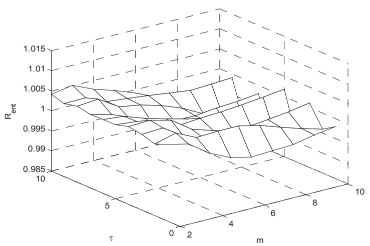

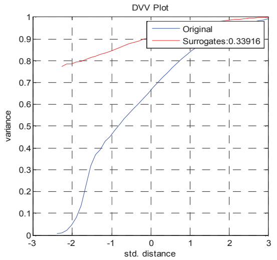

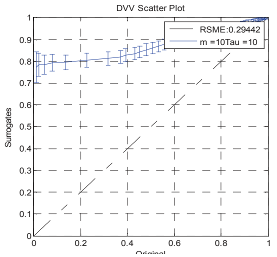

EXP 8

STRAIN 1 | STRAIN 2 | STRAIN 3 | STRAIN 4

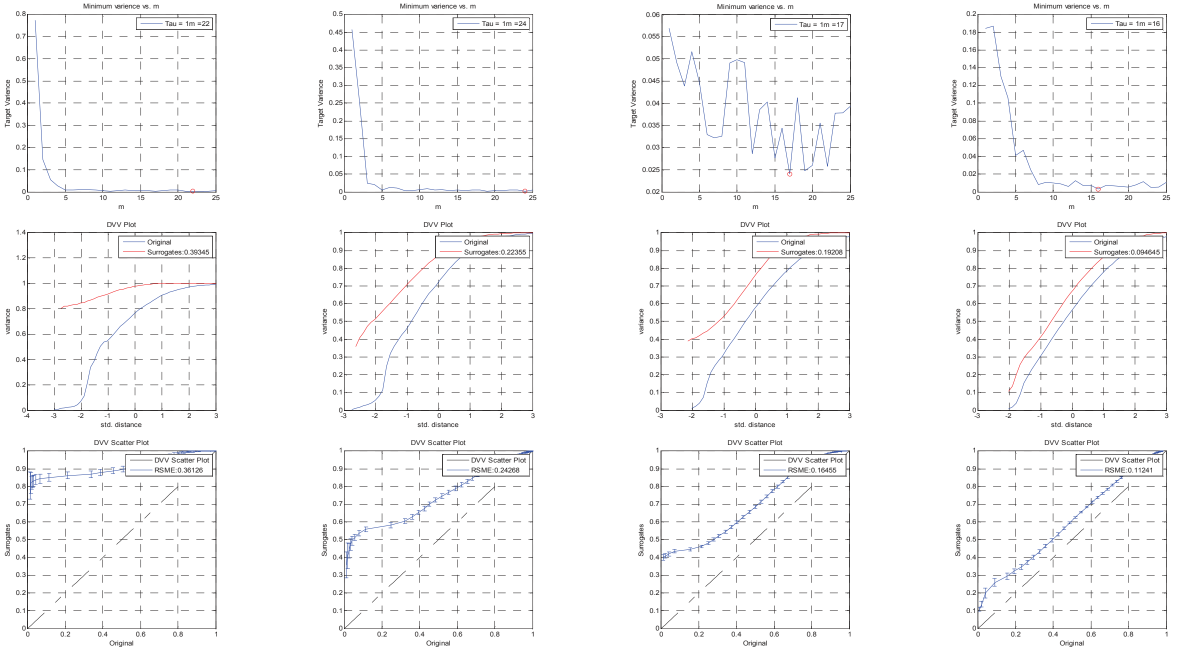

STRAIN 1 | STRAIN 2 | STRAIN 3 | STRAIN 4

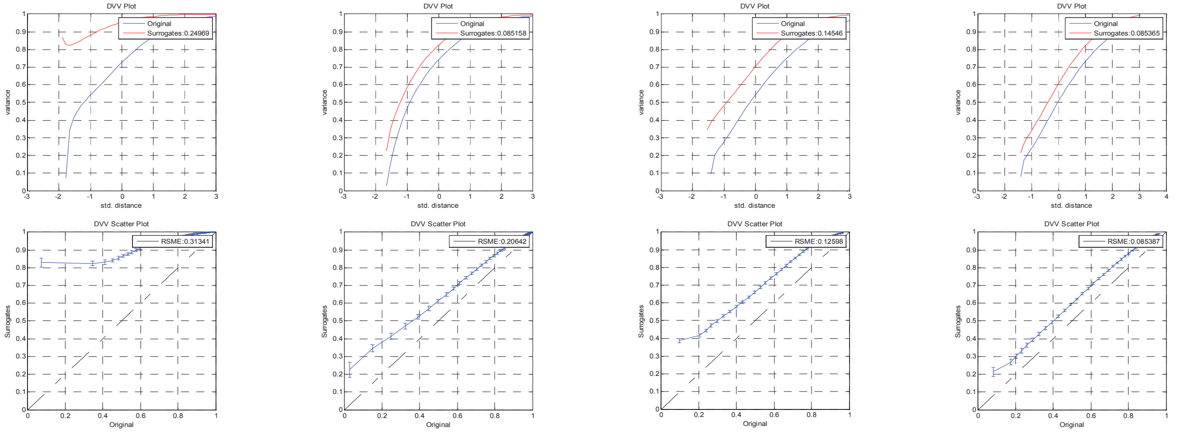

EXPERIMENT	VARIABLES		METHOD 1				METHOD 2				METHOD 3			
			best **m**	best **τ**	rsme	RSME	calc **m**	**τ**	rsme	RSME	set **m**	set **τ**	rsme	RSME
9	Loading White noise 4.365Hz at the peak Focus at top strain gauge	CH1	2	7	0.0203	0.1421	7	1	0.0114	0.1050	3	1	0.0121	0.0704
		CH2	5	1	0.0394	0.1675	18	1	0.0761	0.1552	3	1	0.0297	0.1257
		CH3	5	1	0.0123	0.1200	25	1	0.0501	0.2024	3	1	0.0222	0.0947
		LDVg	5	1	0.2075	0.3547	15	1	0.0690	0.3539	3	1	0.0522	0.2421
		LDV1	3	9	0.0339	0.1178	15	1	0.0119	0.1097	3	1	0.0071	0.0961
		LDV2	2	1	0.0059	0.1578	17	1	0.2185	0.2670	3	1	0.1820	0.2898
		Strain 1	3	3	0.0082	0.3065	6	1	0.2182	0.3492	3	1	0.2197	0.3364
		Strain 2	2	9	0.0052	0.3211	7	1	0.0842	0.2754	3	1	0.0730	0.2318
		Strain 3	10	6	0.2223	0.2959	23	1	0.1423	0.1939	3	1	0.1275	0.1328
		Strain 4	10	10	0.0073	0.1965	7	1	0.0813	0.1675	3	1	0.0804	0.0913

METHOD 1 for data section			
best **m**	best **τ**	rsme	RSME
2	7	0.0090	0.1557
5	1	0.2138	0.2550
5	1	0.0167	0.1036

Data recorded 3D Accelerometer

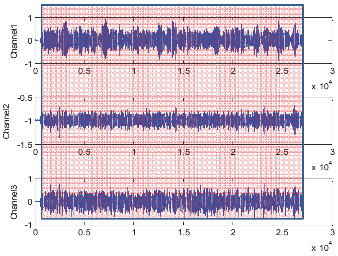

Data analysed 3D Accelerometer

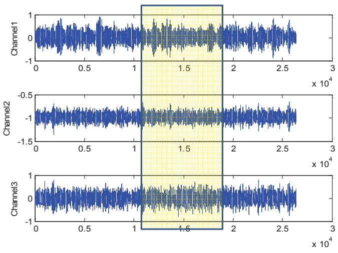

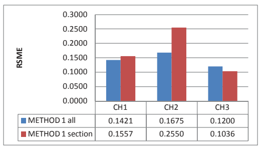

METHOD 1

CH1

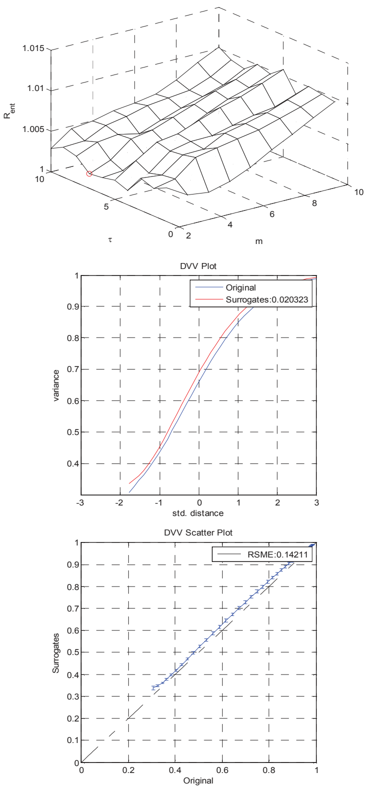

CH2

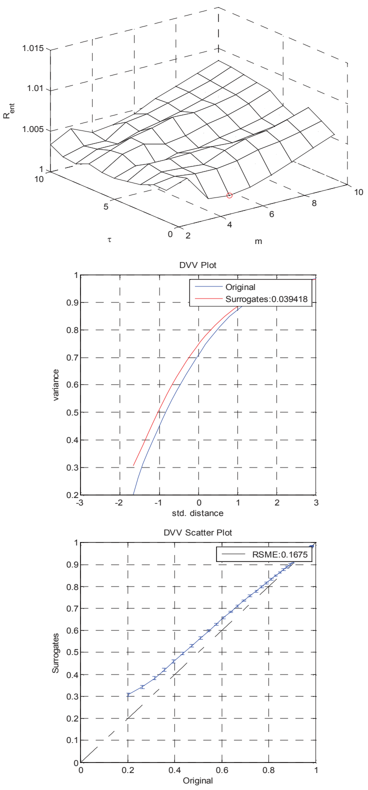

CH3

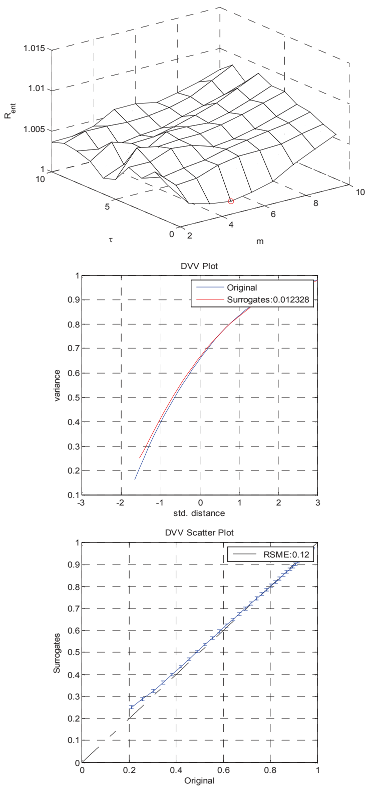

METHOD 2

CH1 **CH2** **CH3**

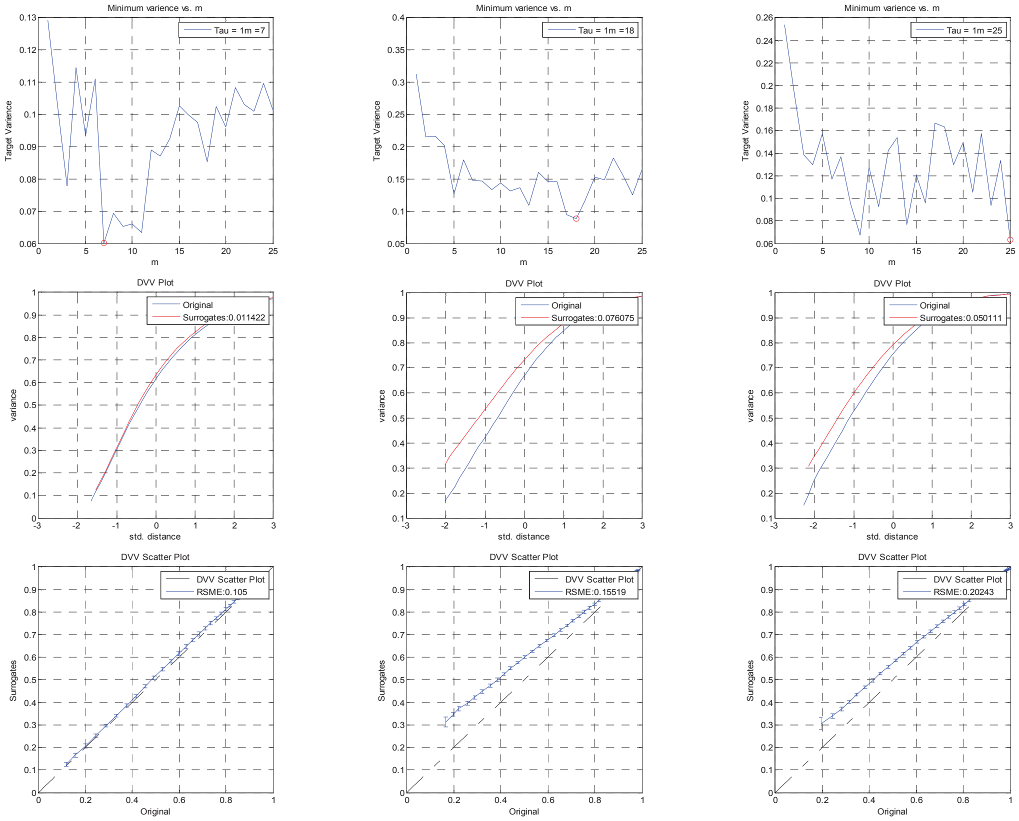

METHOD 3

CH1 **CH2** **CH3**

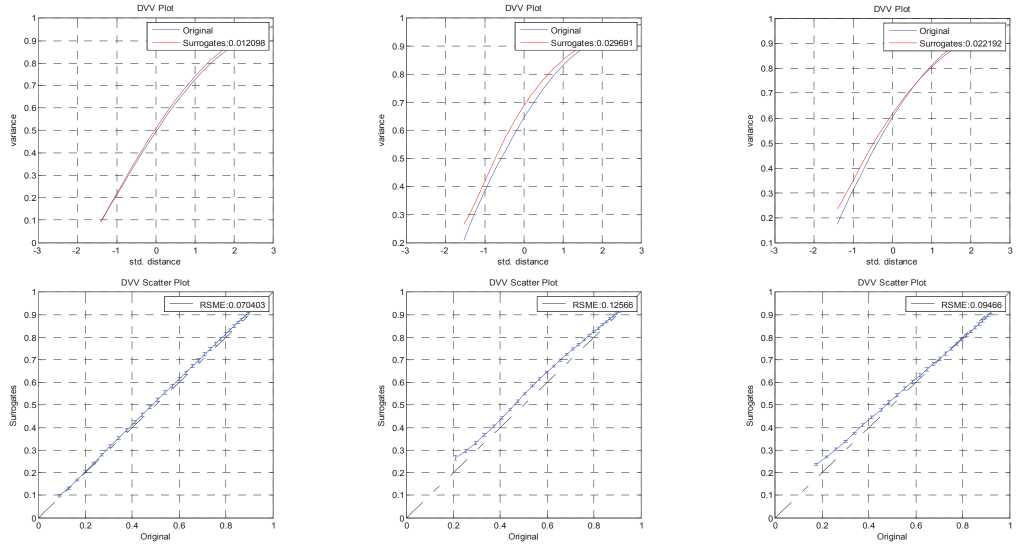

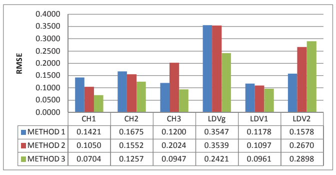

	CH1	CH2	CH3	LDVg	LDV1	LDV2
METHOD 1	0.1421	0.1675	0.1200	0.3547	0.1178	0.1578
METHOD 2	0.1050	0.1552	0.2024	0.3539	0.1097	0.2670
METHOD 3	0.0704	0.1257	0.0947	0.2421	0.0961	0.2898

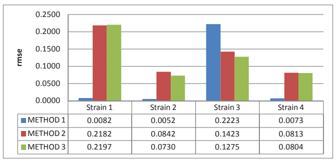

	Strain 1	Strain 2	Strain 3	Strain 4
METHOD 1	0.0082	0.0052	0.2223	0.0073
METHOD 2	0.2182	0.0842	0.1423	0.0813
METHOD 3	0.2197	0.0730	0.1275	0.0804

Data recorded LDV

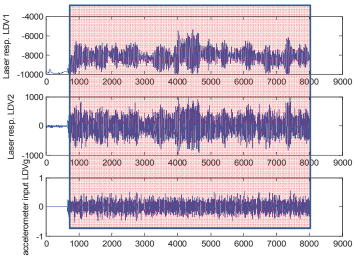

Data analysed LDV

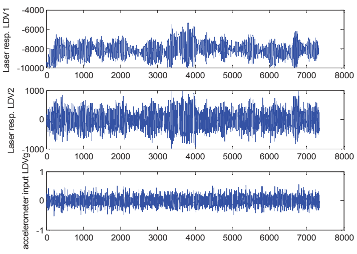

LDVg

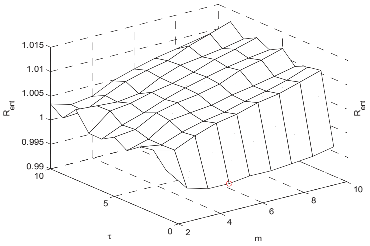

LDV1

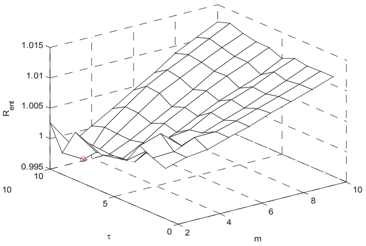

LDV2

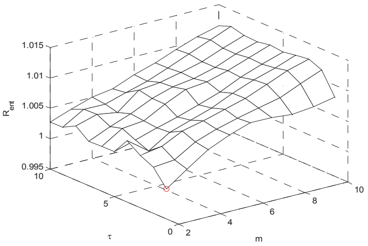

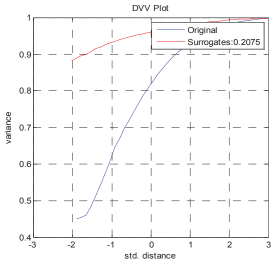

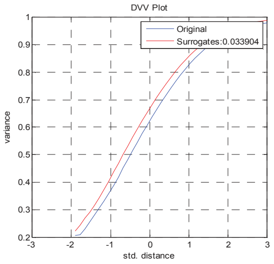

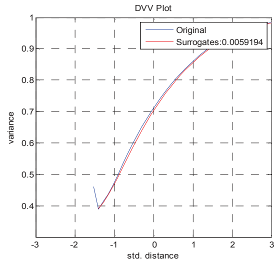

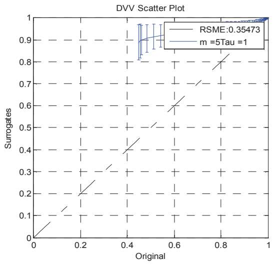

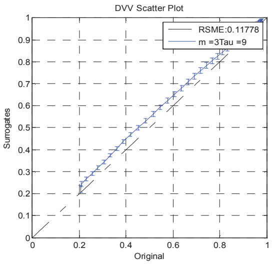

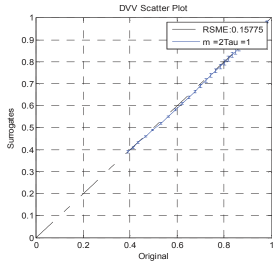

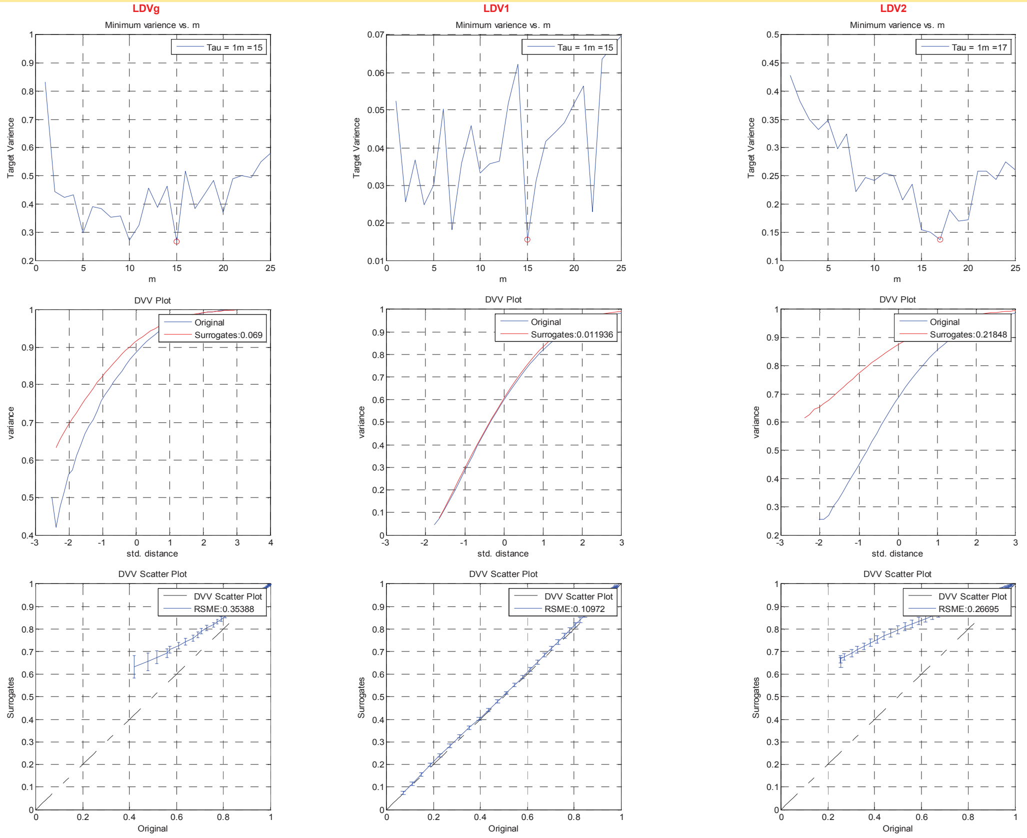
LDVg
LDV1
LDV2
Minimum varience vs. m
Tau = 1m =15
Tau = 1m =15
Tau = 1m =17
Target Varience
m
DVV Plot
Original
Surrogates:0.069
Surrogates:0.011936
Surrogates:0.21848
variance
std. distance
DVV Scatter Plot
RSME:0.35388
RSME:0.10972
RSME:0.26695
Surrogates
Original

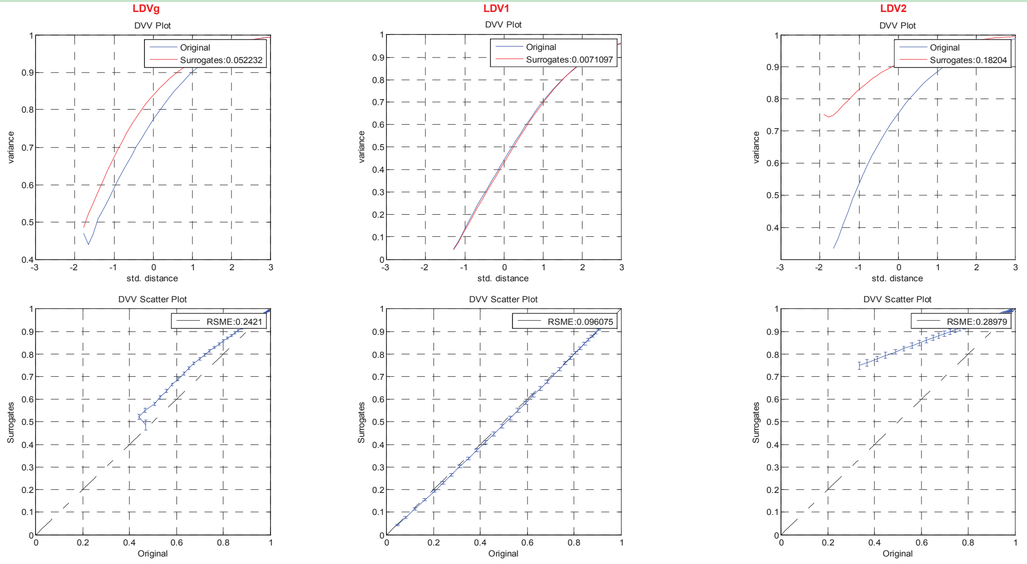
LDVg
LDV1
LDV2
DVV Plot
Original
Surrogates:0.052232
Surrogates:0.0071097
Surrogates:0.18204
variance
std. distance
DVV Scatter Plot
RSME:0.2421
RSME:0.096075
RSME:0.28979
Surrogates
Original

Data recorded Strain Gauge

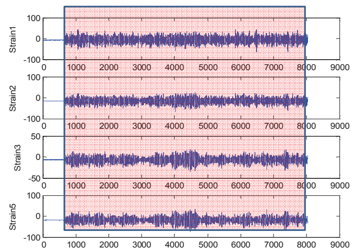

Data analysed Strain Gauge

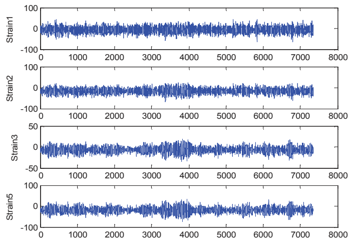

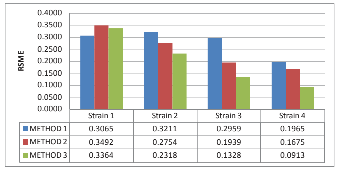

	Strain 1	Strain 2	Strain 3	Strain 4
METHOD 1	0.3065	0.3211	0.2959	0.1965
METHOD 2	0.3492	0.2754	0.1939	0.1675
METHOD 3	0.3364	0.2318	0.1328	0.0913

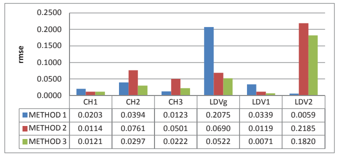

	CH1	CH2	CH3	LDVg	LDV1	LDV2
METHOD 1	0.0203	0.0394	0.0123	0.2075	0.0339	0.0059
METHOD 2	0.0114	0.0761	0.0501	0.0690	0.0119	0.2185
METHOD 3	0.0121	0.0297	0.0222	0.0522	0.0071	0.1820

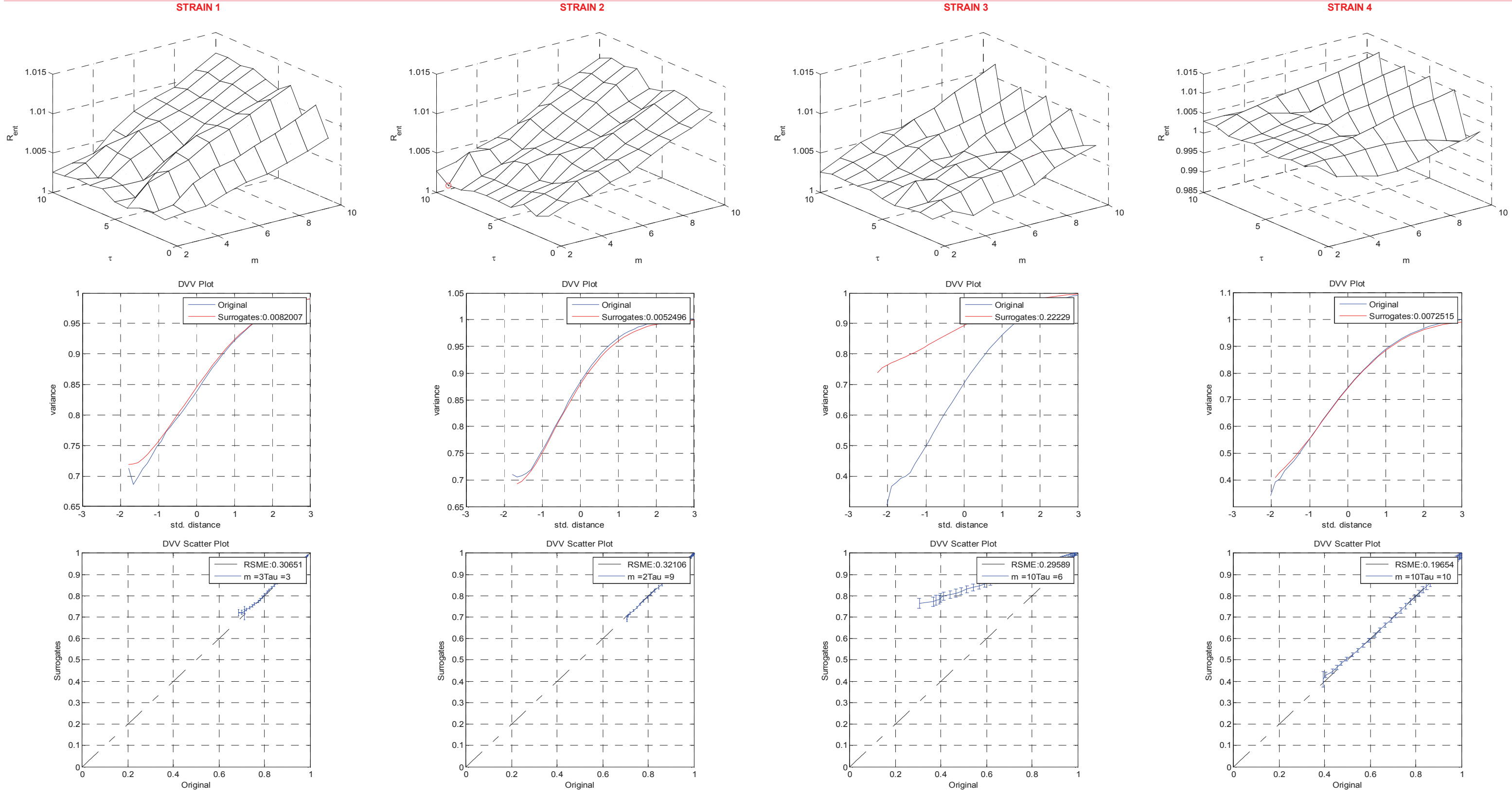

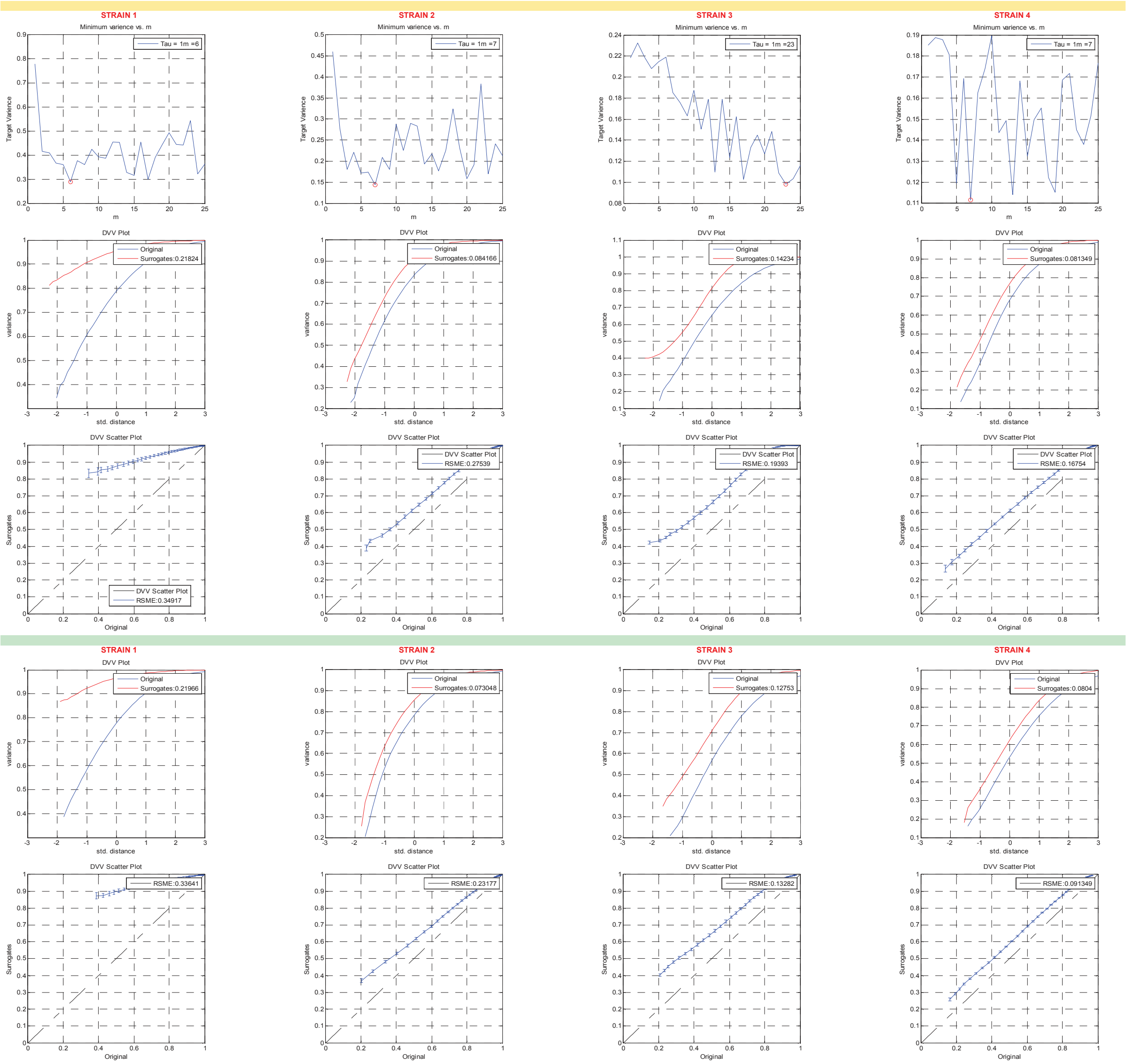

STRAIN 1
STRAIN 2
STRAIN 3
STRAIN 4
Minimum varience vs. m
Tau = 1m =6
Tau = 1m =7
Tau = 1m =23
Tau = 1m =7
Target Varience
m
DVV Plot
Original
Surrogates:0.21824
Surrogates:0.084166
Surrogates:0.14234
Surrogates:0.081349
variance
std. distance
DVV Scatter Plot
DVV Scatter Plot
RSME:0.34917
RSME:0.27539
RSME:0.19393
RSME:0.16754
Surrogates
Original
STRAIN 1
STRAIN 2
STRAIN 3
STRAIN 4
DVV Plot
Original
Surrogates:0.21966
Surrogates:0.073048
Surrogates:0.12753
Surrogates:0.0804
variance
std. distance
DVV Scatter Plot
RSME:0.33641
RSME:0.23177
RSME:0.13282
RSME:0.091349
Surrogates
Original

EXPERIMENT	VARIABLES		METHOD 1				METHOD 2				METHOD 3			
			best **m**	best **τ**	rsme	RSME	calc **m**	**τ**	rsme	RSME	set **m**	set **τ**	rsme	RSME
10	Loading Sine Sweep<br>2.0 Hz 6.0 Hz<br>60 sec<br>Focus at top strain gauge	CH1	7	10	0.0696	0.1634	19	1	0.0964	0.2472	3	1	0.0406	0.2608
		CH2	2	8	0.0451	0.2468	20	1	0.0536	0.1117	3	1	0.0443	0.0576
		CH3	5	10	0.0142	0.1677	3	1	0.0102	0.2814	3	1	0.0688	0.3002
		LDVg	10	2	0.3382	0.2526	4	1	0.0559	0.1080	3	1	0.0501	0.0948
		LDV1	6	9	0.3271	0.1985	22	1	0.0585	0.1792	3	1	0.0137	0.1854
		LDV2	8	10	0.1156	0.1703	15	1	0.3039	0.1775	3	1	0.2673	0.1696
		Strain 1	10	10	0.1380	0.1337	14	1	0.4121	0.2053	3	1	0.2496	0.1449
		Strain 2	10	9	0.3985	0.2059	10	1	0.2505	0.1497	3	1	0.2046	0.1533
		Strain 3	10	9	0.4153	0.2148	11	1	0.3410	0.1694	3	1	0.3243	0.1976
		Strain 4	10	9	0.3978	0.2049	12	1	0.3542	0.1764	3	1	0.2607	0.1687

METHOD 1 for data section			
best **m**	best **τ**	rsme	RSME
7	10	0.072	0.283
2	8	0.151	0.217
5	10	0.210	0.164

Data recorded 3D Accelerometer

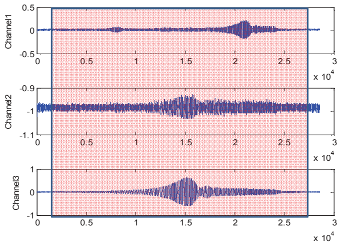

Data analysed 3D Accelerometer

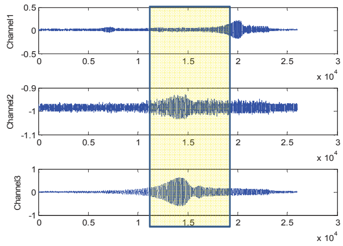

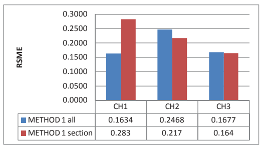

METHOD 1

CH1

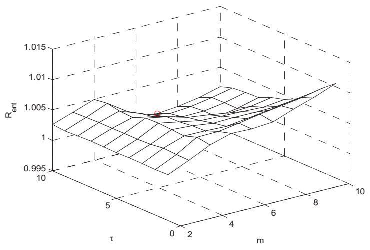

CH2

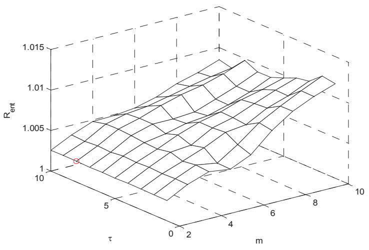

CH3

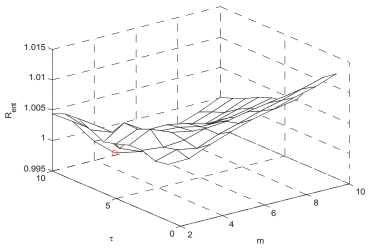

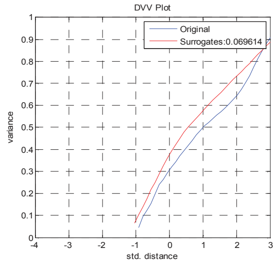

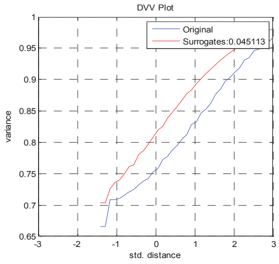

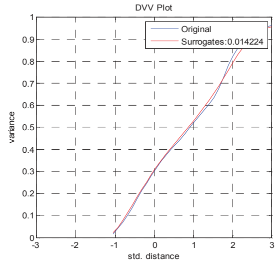

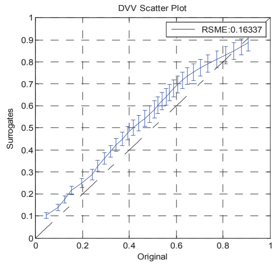

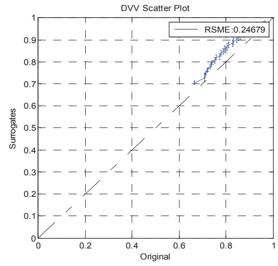

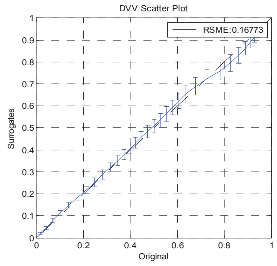

METHOD 2

CH1 CH2 CH3

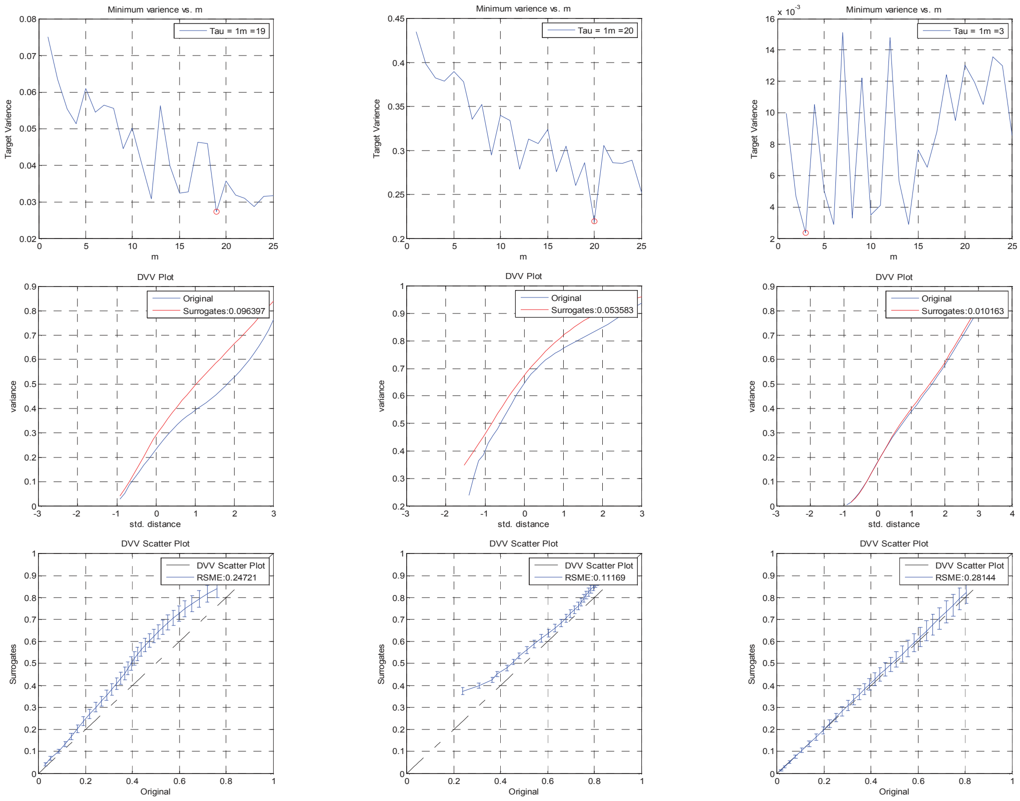

METHOD 3

CH1 CH2 CH3

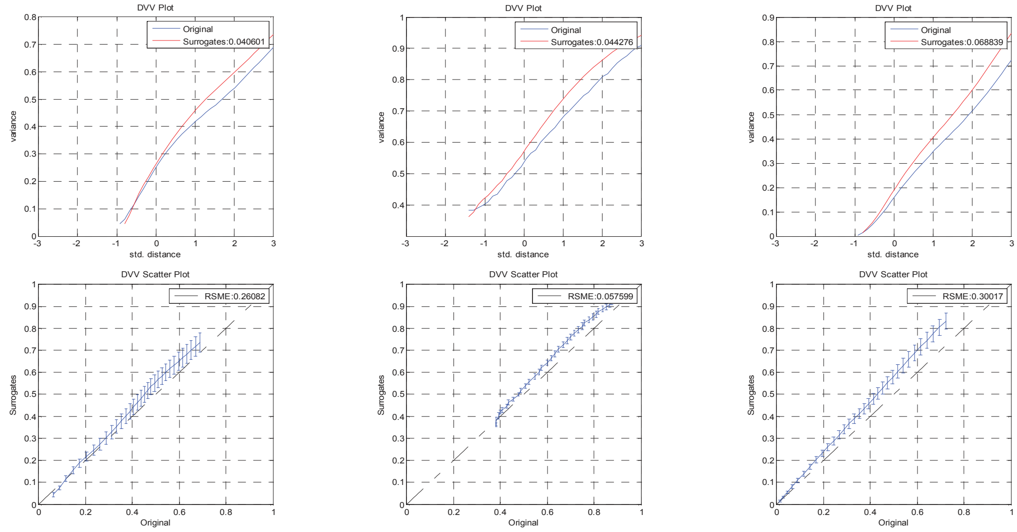

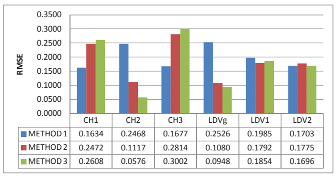

	CH1	CH2	CH3	LDVg	LDV1	LDV2
METHOD 1	0.1634	0.2468	0.1677	0.2526	0.1985	0.1703
METHOD 2	0.2472	0.1117	0.2814	0.1080	0.1792	0.1775
METHOD 3	0.2608	0.0576	0.3002	0.0948	0.1854	0.1696

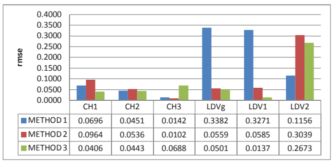

	CH1	CH2	CH3	LDVg	LDV1	LDV2
METHOD 1	0.0696	0.0451	0.0142	0.3382	0.3271	0.1156
METHOD 2	0.0964	0.0536	0.0102	0.0559	0.0585	0.3039
METHOD 3	0.0406	0.0443	0.0688	0.0501	0.0137	0.2673

Data recorded LDV

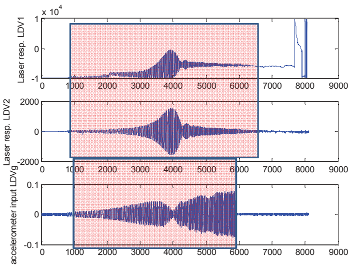

Data analysed LDV

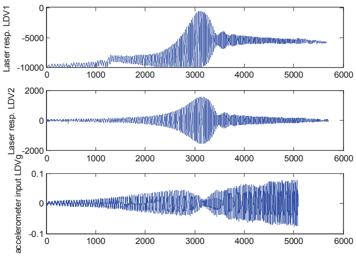

LDVg

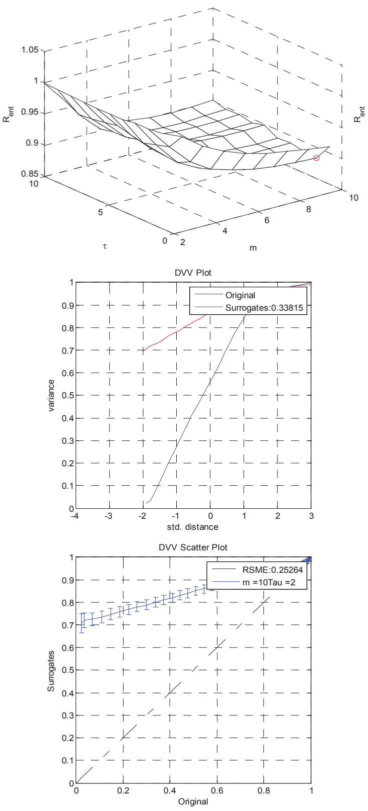

LDV1

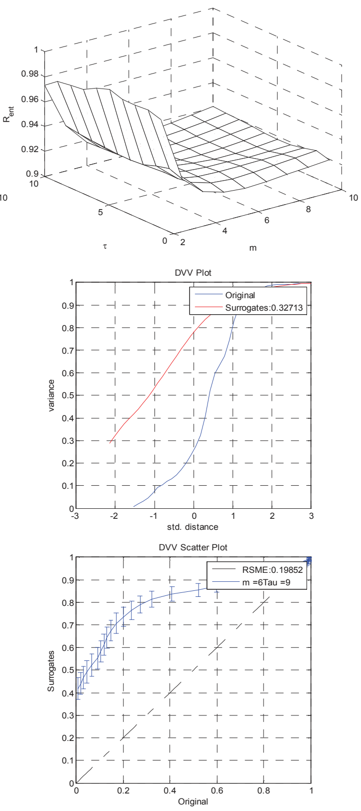

LDV2

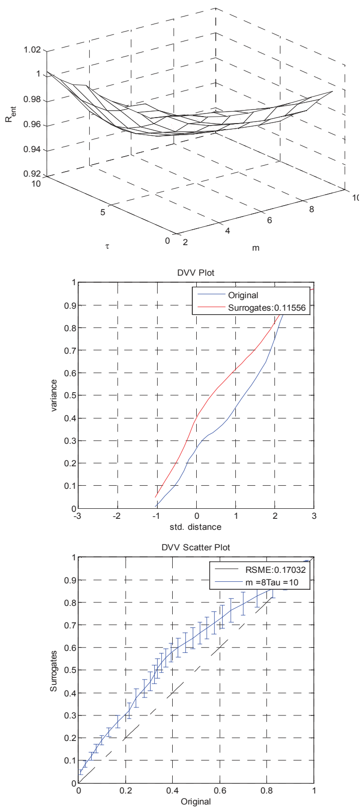

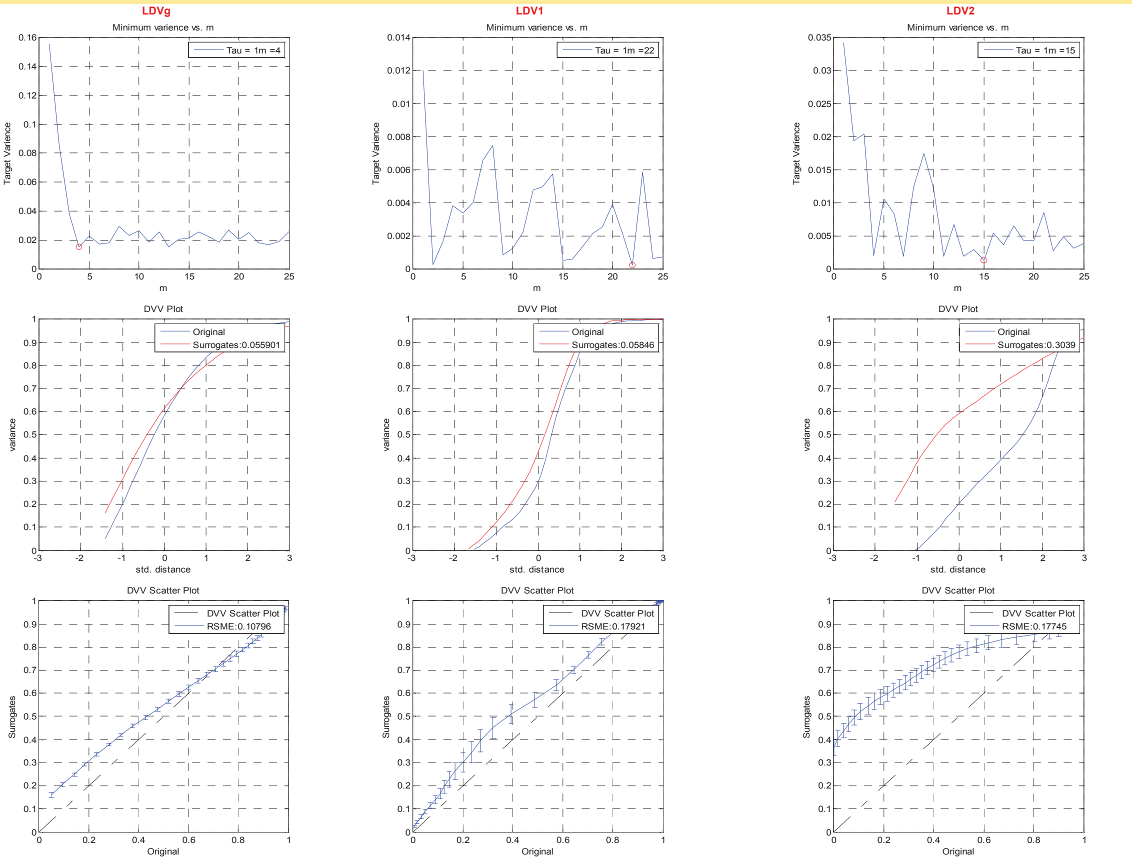
LDVg
LDV1
LDV2
Minimum varience vs. m
Tau = 1m =4
Tau = 1m =22
Tau = 1m =15
Target Varience
m
DVV Plot
Original
Surrogates:0.055901
Surrogates:0.05846
Surrogates:0.3039
variance
std. distance
DVV Scatter Plot
RSME:0.10796
RSME:0.17921
RSME:0.17745
Surrogates
Original

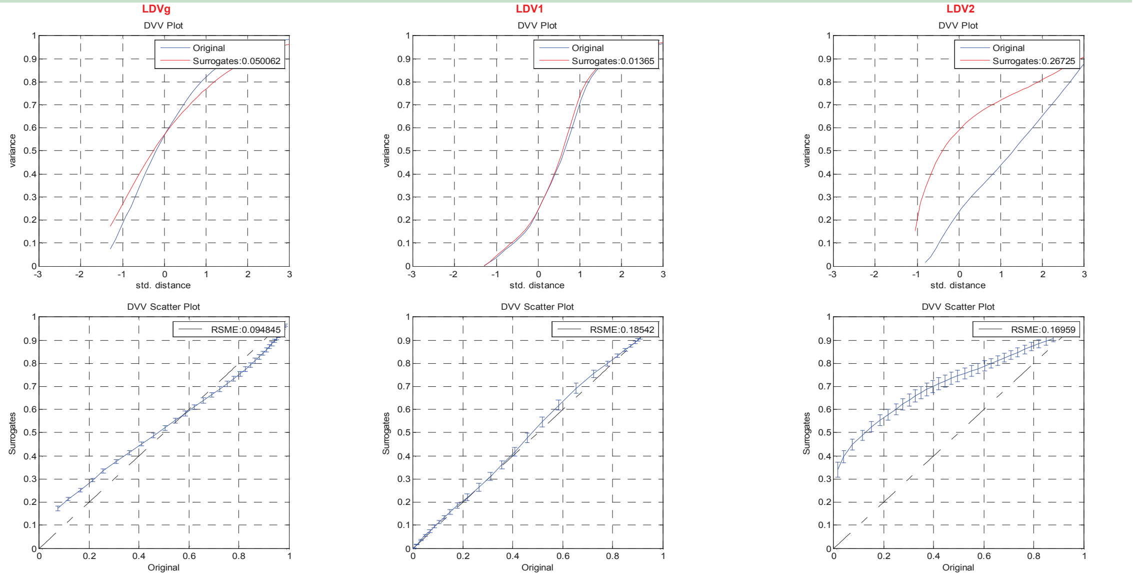
LDVg
LDV1
LDV2
DVV Plot
Original
Surrogates:0.050062
Surrogates:0.01365
Surrogates:0.26725
variance
std. distance
DVV Scatter Plot
RSME:0.094845
RSME:0.18542
RSME:0.16959
Surrogates
Original

Data recorded Strain Gauge

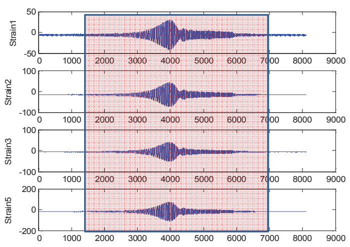

Data analysed Strain Gauge

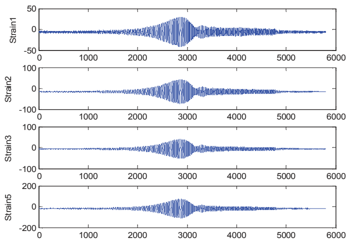

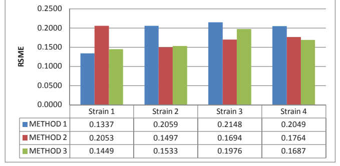

	Strain 1	Strain 2	Strain 3	Strain 4
METHOD 1	0.1337	0.2059	0.2148	0.2049
METHOD 2	0.2053	0.1497	0.1694	0.1764
METHOD 3	0.1449	0.1533	0.1976	0.1687

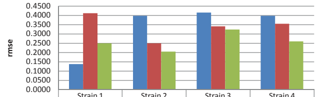

	Strain 1	Strain 2	Strain 3	Strain 4
METHOD 1	0.1380	0.3985	0.4153	0.3978
METHOD 2	0.4121	0.2505	0.3410	0.3542
METHOD 3	0.2496	0.2046	0.3243	0.2607

STRAIN 1

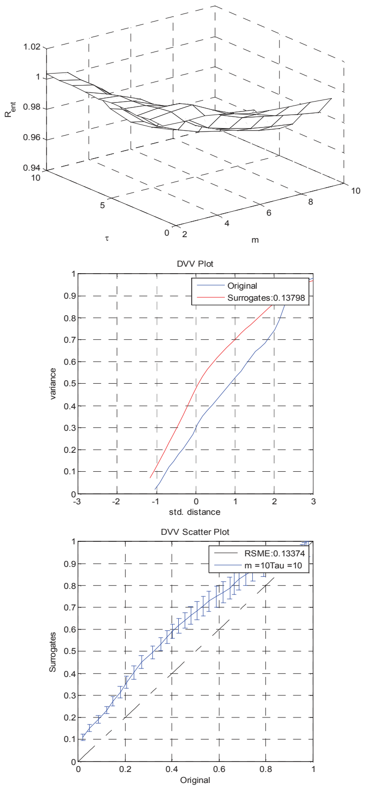

STRAIN 2

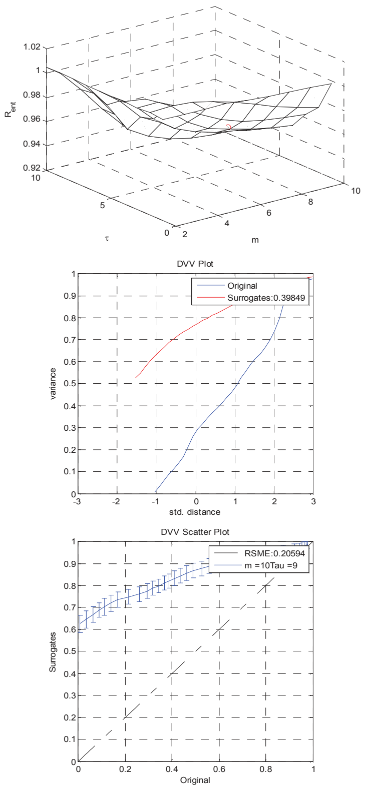

STRAIN 3

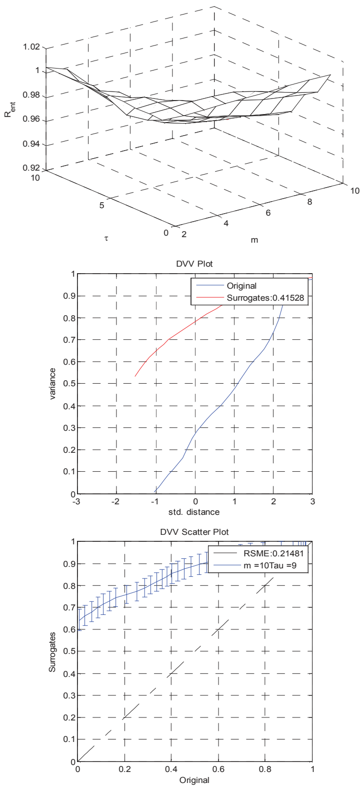

STRAIN 4

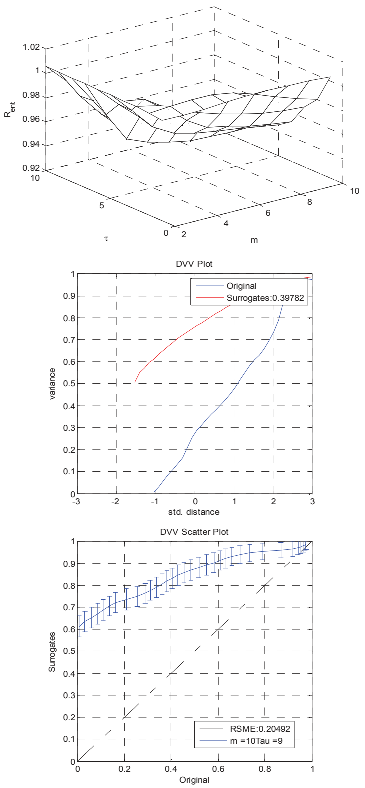

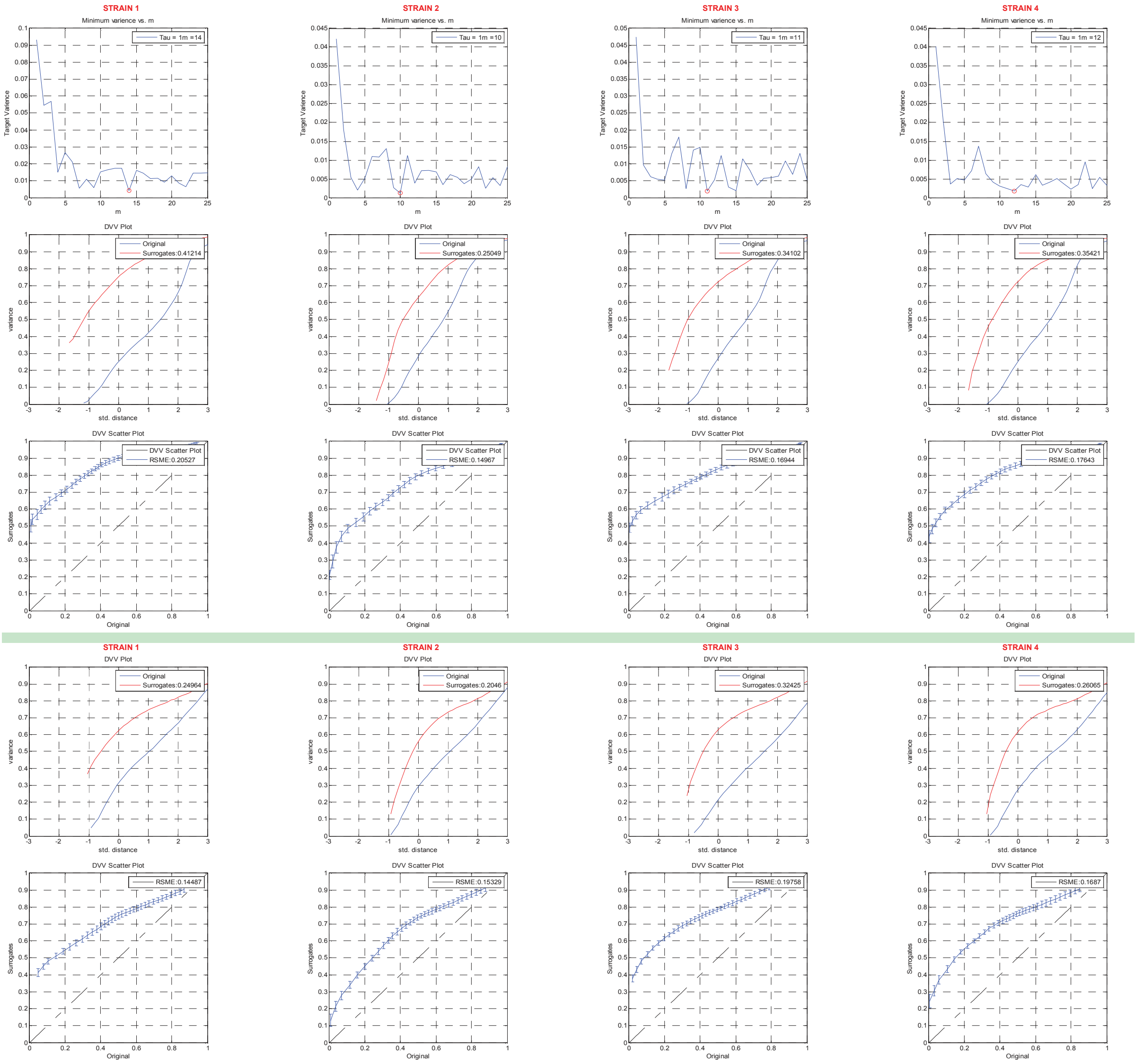
STRAIN 1
STRAIN 2
STRAIN 3
STRAIN 4
Minimum varience vs. m
Target Varience
m
Tau = 1m =14
Tau = 1m =10
Tau = 1m =11
Tau = 1m =12
DVV Plot
variance
std. distance
Original
Surrogates:0.41214
Surrogates:0.25049
Surrogates:0.34102
Surrogates:0.35421
DVV Scatter Plot
Surrogates
Original
DVV Scatter Plot
RSME:0.20527
RSME:0.14967
RSME:0.16944
RSME:0.17643
STRAIN 1
STRAIN 2
STRAIN 3
STRAIN 4
Surrogates:0.24964
Surrogates:0.2046
Surrogates:0.32425
Surrogates:0.26065
RSME:0.14487
RSME:0.15329
RSME:0.19758
RSME:0.1687

EXPERIMENT	VARIABLES		METHOD 1				METHOD 2				METHOD 3			
			best **m**	best **τ**	rsme	RSME	calc **m**	**τ**	rsme	RSME	set **m**	set **τ**	rsme	RSME
11	Harmonic resonance<br>2.0 Hz 2.5 Hz<br>3.0 Hz 3.5 Hz<br>4.0 Hz 4.2 Hz<br>4.3 Hz 4.4 Hz<br>4.5 Hz 4.6 Hz<br>5.0Hz 5.5 Hz<br>6.0 Hz 6.5Hz<br>7.0Hz<br>Focus at top strain gauge	CH1	7	10	0.0832	0.1831	19	1	0.0857	0.2276	3	1	0.0090	0.2512
		CH2	6	10	0.0562	0.1197	22	1	0.0283	0.0817	3	1	0.0116	0.0566
		CH3	4	9	0.0117	0.1400	22	1	0.0071	0.1270	3	1	0.0033	0.1931
		LDVg	10	3	0.1712	0.1685	25	1	0.1249	0.1246	3	1	0.0476	0.1111
		LDV1	8	10	0.1882	0.1488	22	1	0.1733	0.1619	3	1	0.0513	0.1534
		LDV2	10	10	0.3138	0.1713	23	1	0.2482	0.1402	3	1	0.2315	0.1334
		Strain 1	10	10	0.2620	0.1491	17	1	0.4013	0.2202	3	1	0.2123	0.1152
		Strain 2	10	9	0.3953	0.2354	21	1	0.3584	0.1932	3	1	0.2224	0.1279
		Strain 3	10	9	0.3963	0.2366	5	1	0.2270	0.1398	3	1	0.2484	0.1399
		Strain 4	10	9	0.3737	0.2128	19	1	0.4437	0.2166	3	1	0.2365	0.1354

Data recorded 3D Accelerometer

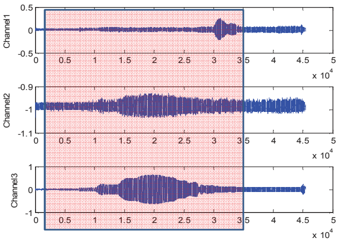

Data analysed 3D Accelerometer

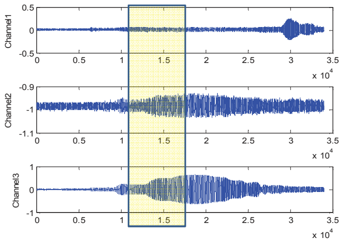

METHOD 1

CH1

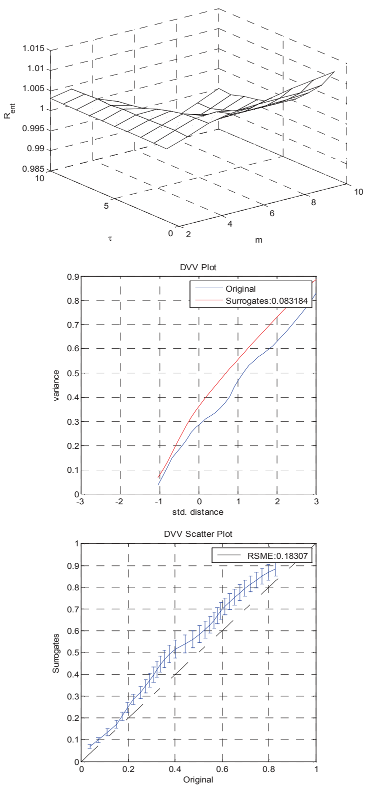

CH2

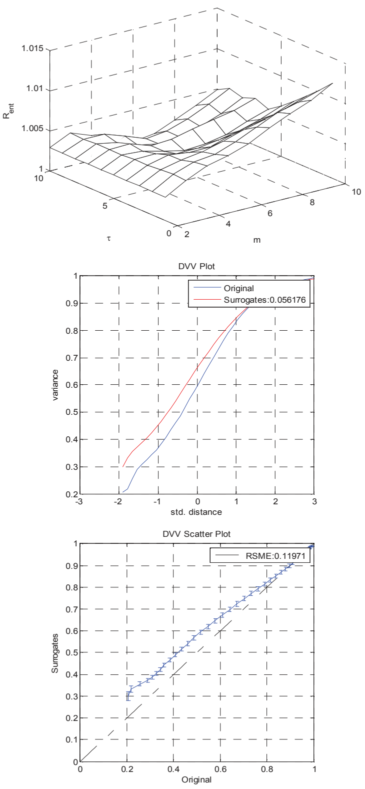

CH3

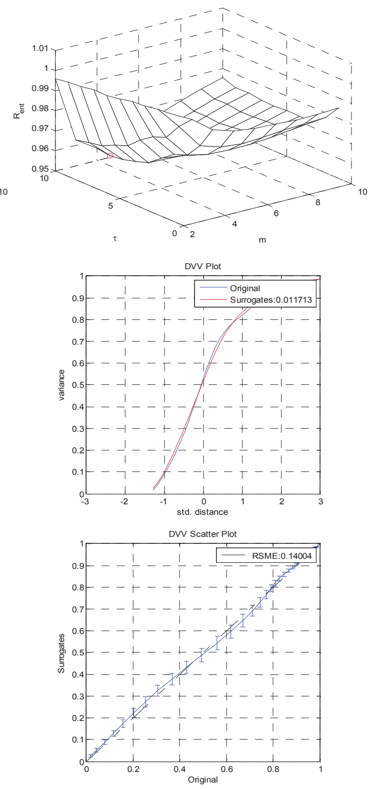

METHOD 2

CH1 | CH2 | CH3

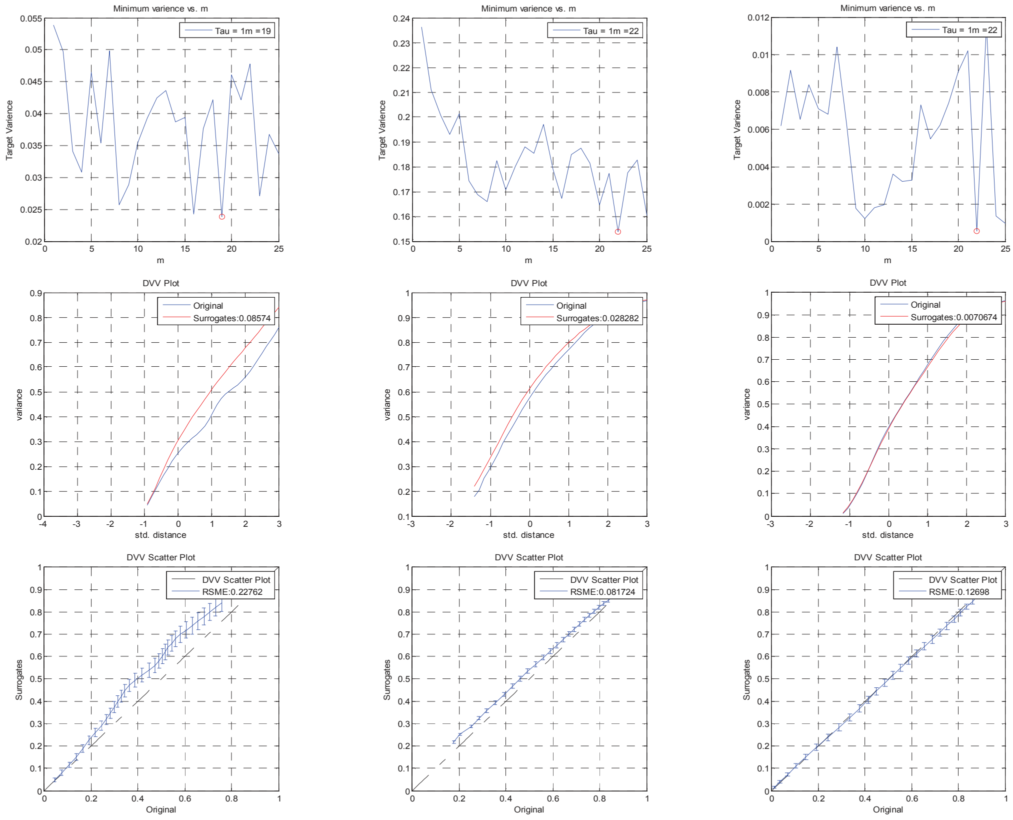

METHOD 3

CH1 | CH2 | CH3

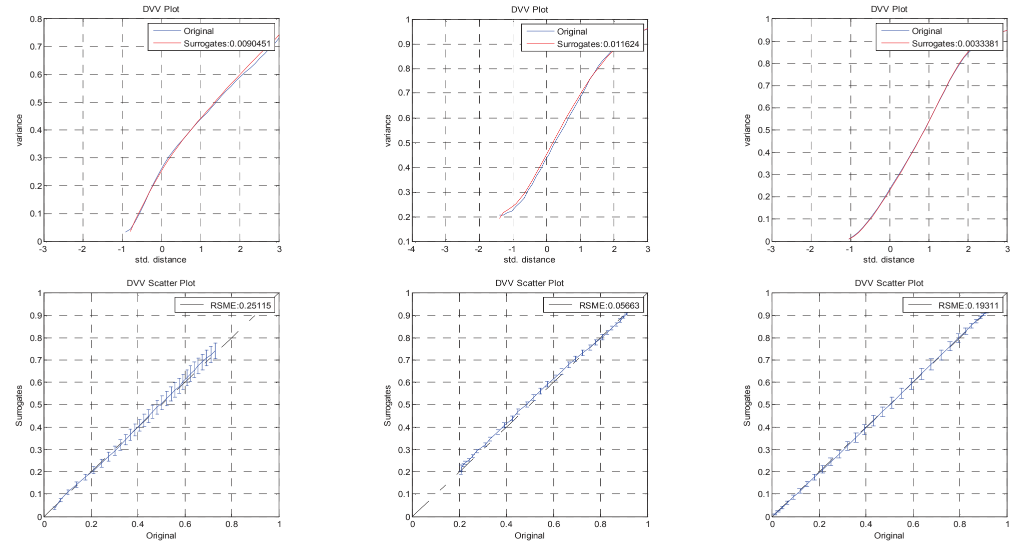

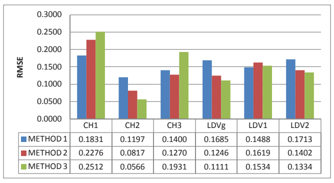

	CH1	CH2	CH3	LDVg	LDV1	LDV2
METHOD 1	0.1831	0.1197	0.1400	0.1685	0.1488	0.1713
METHOD 2	0.2276	0.0817	0.1270	0.1246	0.1619	0.1402
METHOD 3	0.2512	0.0566	0.1931	0.1111	0.1534	0.1334

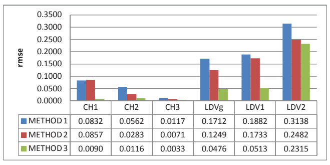

	CH1	CH2	CH3	LDVg	LDV1	LDV2
METHOD 1	0.0832	0.0562	0.0117	0.1712	0.1882	0.3138
METHOD 2	0.0857	0.0283	0.0071	0.1249	0.1733	0.2482
METHOD 3	0.0090	0.0116	0.0033	0.0476	0.0513	0.2315

Data recorded LDV

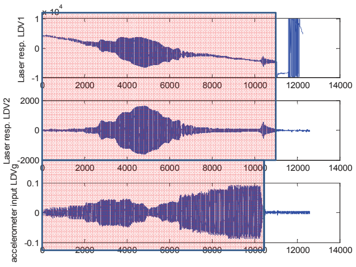

Data analysed LDV

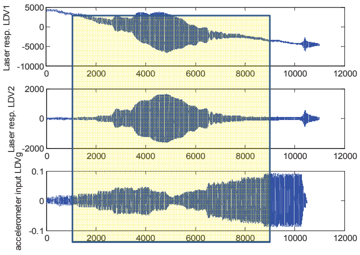

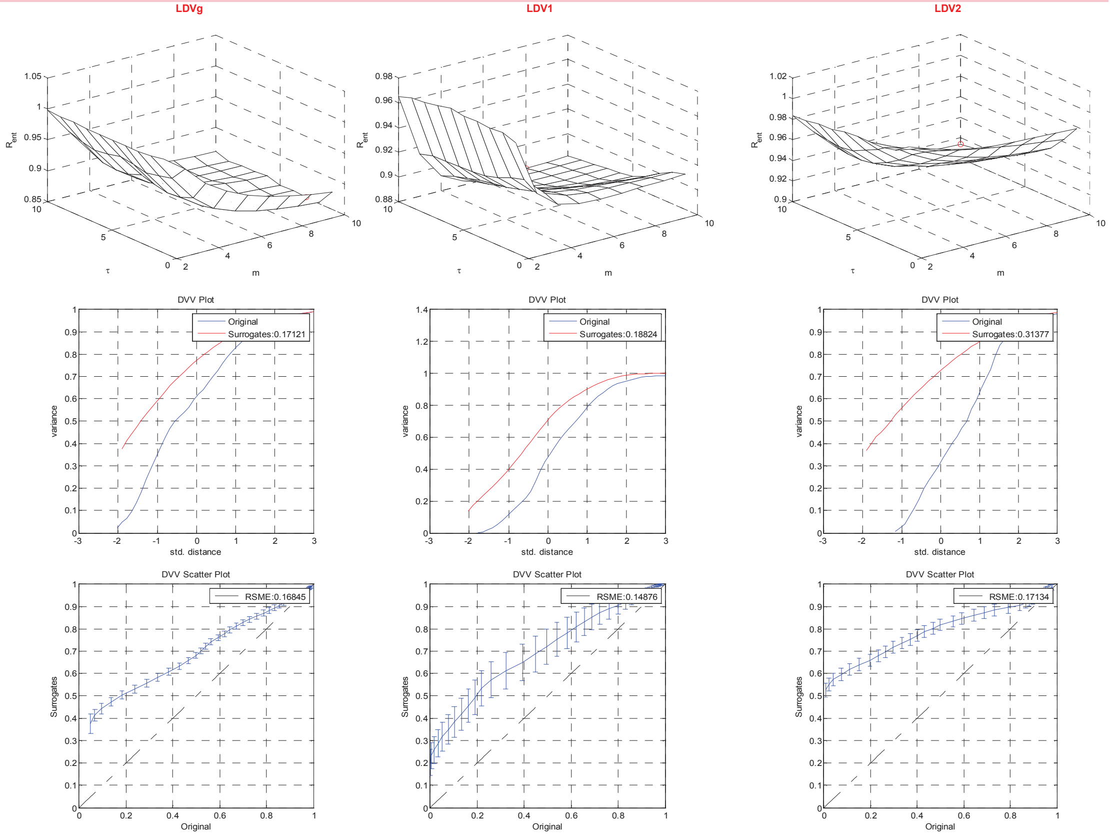

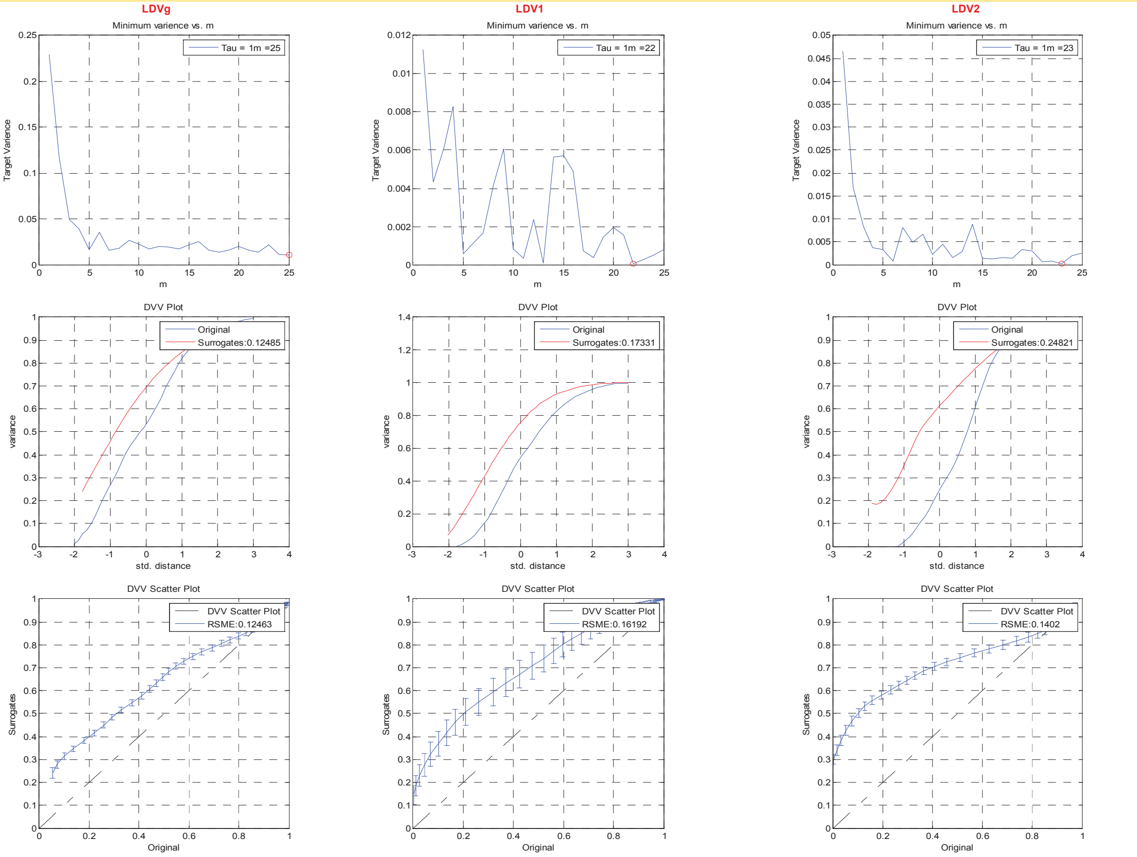
LDVg
LDV1
LDV2
Minimum varience vs. m
Target Varience
Tau = 1m =25
Tau = 1m =22
Tau = 1m =23
m
DVV Plot
variance
std. distance
Original
Surrogates:0.12485
Surrogates:0.17331
Surrogates:0.24821
DVV Scatter Plot
Surrogates
Original
RSME:0.12463
RSME:0.16192
RSME:0.1402

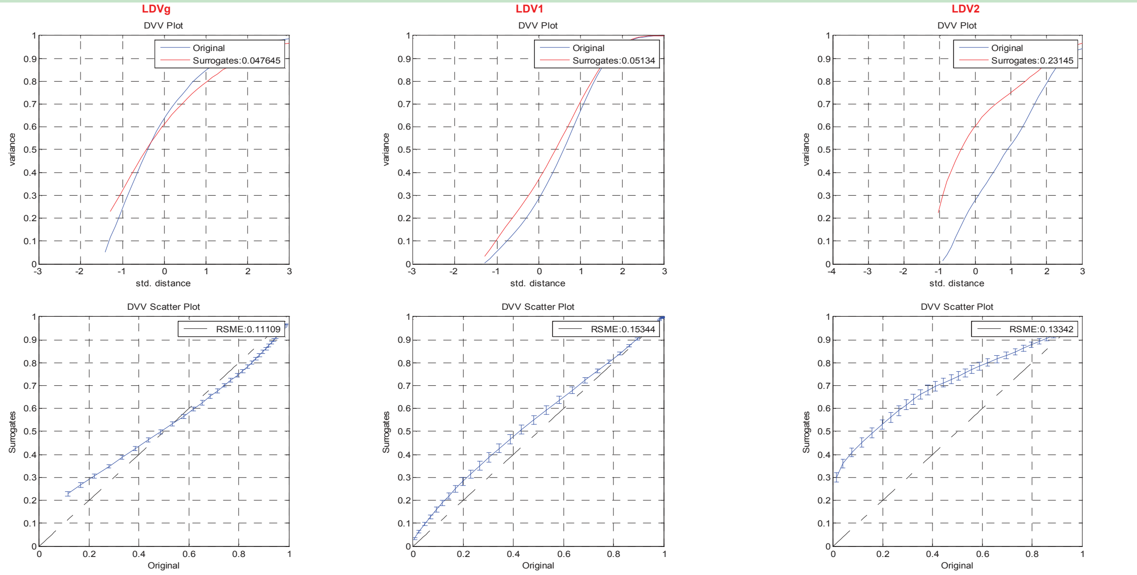
LDVg
LDV1
LDV2
DVV Plot
variance
std. distance
Original
Surrogates:0.047645
Surrogates:0.05134
Surrogates:0.23145
DVV Scatter Plot
Surrogates
Original
RSME:0.11109
RSME:0.15344
RSME:0.13342

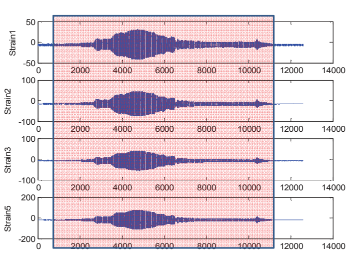

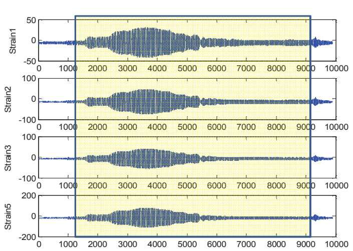

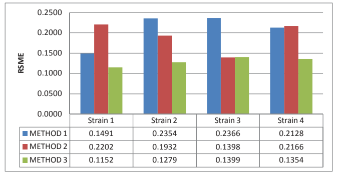

	Strain 1	Strain 2	Strain 3	Strain 4
METHOD 1	0.1491	0.2354	0.2366	0.2128
METHOD 2	0.2202	0.1932	0.1398	0.2166
METHOD 3	0.1152	0.1279	0.1399	0.1354

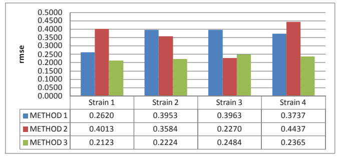

	Strain 1	Strain 2	Strain 3	Strain 4
METHOD 1	0.2620	0.3953	0.3963	0.3737
METHOD 2	0.4013	0.3584	0.2270	0.4437
METHOD 3	0.2123	0.2224	0.2484	0.2365

STRAIN 1

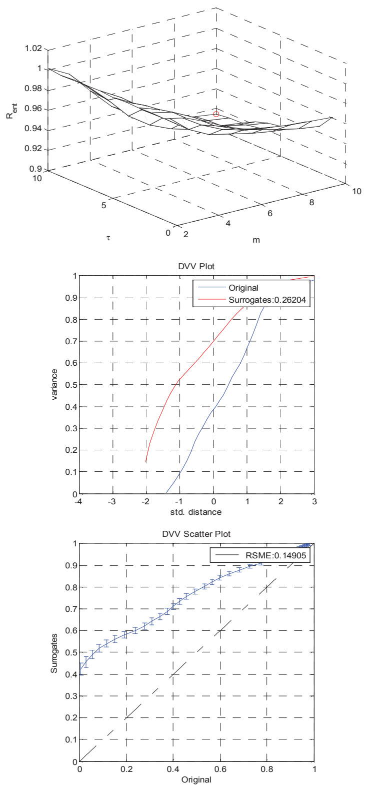

STRAIN 2

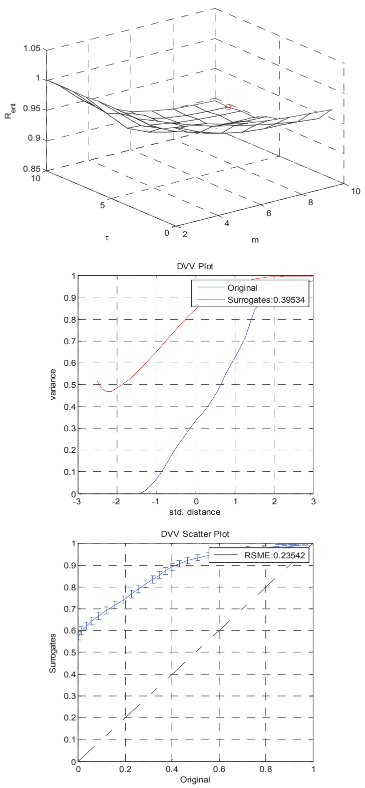

STRAIN 3

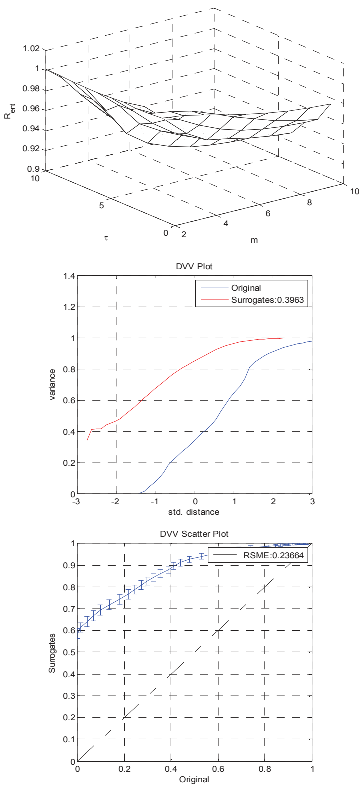

STRAIN 4

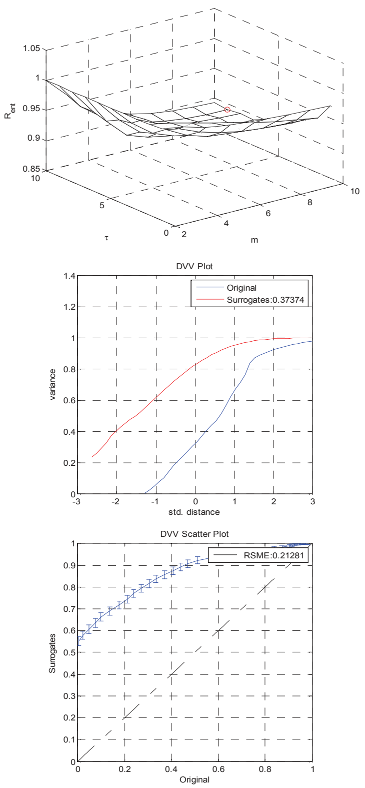

EXPERIMENT	VARIABLES		METHOD 1				METHOD 2				METHOD 3			
			best m	best τ	rsme	RSME	calc m	τ	rsme	RSME	set m	set τ	rsme	RSME
11	Harmonic resonance<br>2.0 Hz 2.5 Hz<br>3.0 Hz 3.5 Hz<br>4.0 Hz 4.2 Hz<br>4.3 Hz 4.4 Hz<br>4.5 Hz 4.6 Hz<br>5.0Hz 5.5 Hz<br>6.0 Hz 6.5Hz<br>7.0Hz<br>Focus at top strain gauge	CH1	7	10	0.0832	0.1831	19	1	0.0857	0.2276	3	1	0.0090	0.2512
		CH2	6	10	0.0562	0.1197	22	1	0.0283	0.0817	3	1	0.0116	0.0566
		CH3	4	9	0.0117	0.1400	22	1	0.0071	0.1270	3	1	0.0033	0.1931
		LDVg	10	3	0.1712	0.1685	25	1	0.1249	0.1246	3	1	0.0476	0.1111
		LDV1	8	10	0.1882	0.1488	22	1	0.1733	0.1619	3	1	0.0513	0.1534
		LDV2	10	10	0.3138	0.1713	23	1	0.2482	0.1402	3	1	0.2315	0.1334
		Strain 1	10	10	0.2620	0.1491	17	1	0.4013	0.2202	3	1	0.2123	0.1152
		Strain 2	10	9	0.3953	0.2354	21	1	0.3584	0.1932	3	1	0.2224	0.1279
		Strain 3	10	9	0.3963	0.2366	5	1	0.2270	0.1398	3	1	0.2484	0.1399
		Strain 4	10	9	0.3737	0.2128	19	1	0.4437	0.2166	3	1	0.2365	0.1354

METHOD 1 for data section

Optimum embeding parameter m and time lag tau are determined using data sqered yellow. Using these parameters RMSE and rmse were calculated for all valid data squered in pink

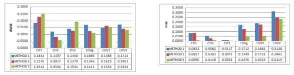

Data recorded 3D Accelerometer **Data analysed 3D Accelerometer**

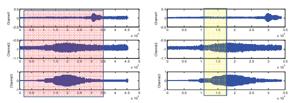

Data recorded LDV **Data analysed LDV**

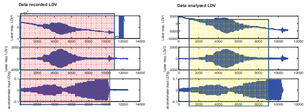

Data recorded Strain Gauge **Data analysed Strain Gauge**

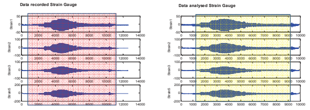

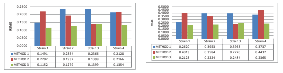

METHOD 1

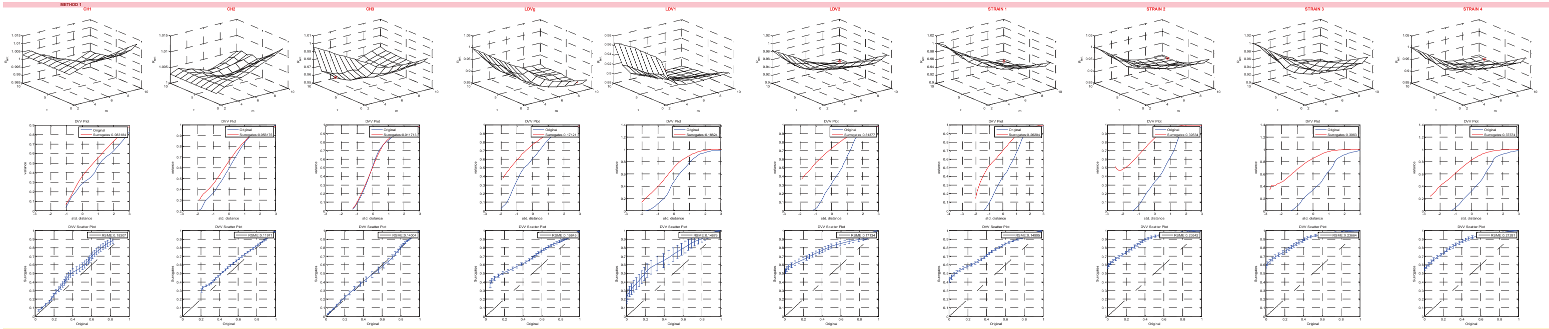

METHOD 2

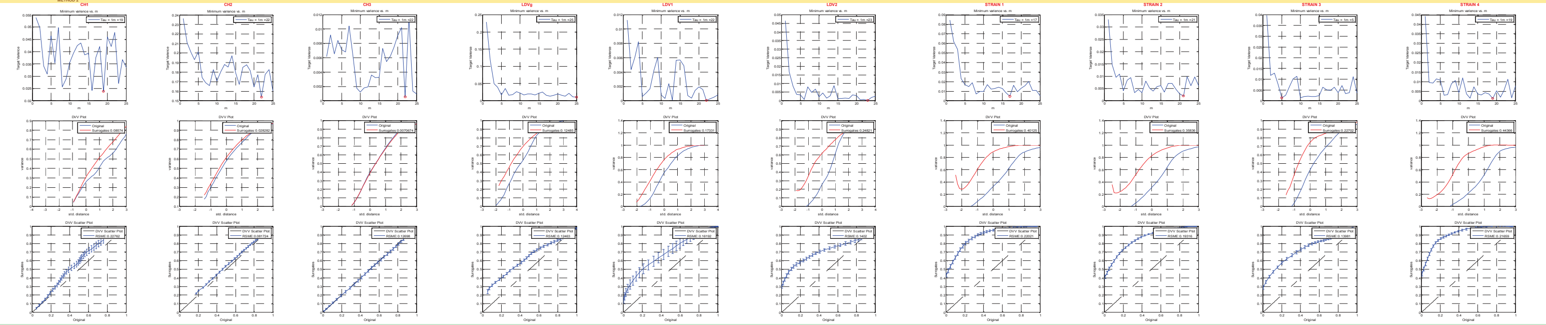

METHOD 3

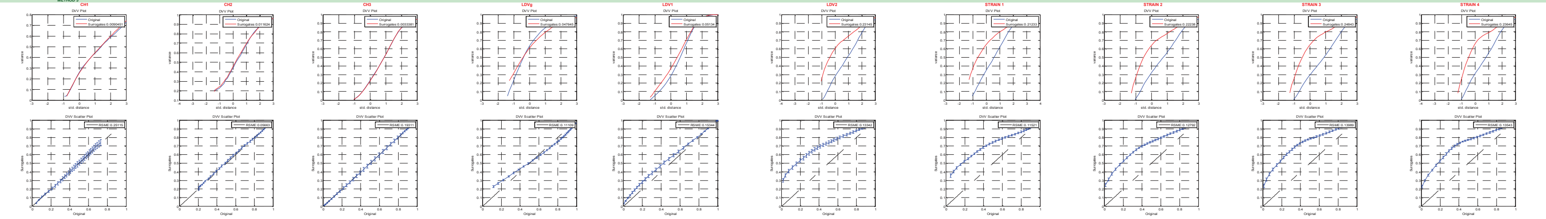

EXPERIMENT	VARIABLES		METHOD 1				METHOD 2				METHOD 3			
			best **m**	best **τ**	rsme	RSME	calc **m**	**τ**	rsme	RSME	set **m**	set **τ**	rsme	RSME
12	Harmonic resonance<br>2.0 Hz 2.5 Hz<br>3.0 Hz 3.5 Hz<br>4.0 Hz 4.2 Hz<br>4.3 Hz 4.4 Hz<br>4.5 Hz 4.6 Hz<br>5.0Hz 5.5 Hz<br>6.0 Hz 6.5Hz<br>7.0Hz<br>Focus at mid strain gauge	CH1	2	1	0.1527	0.1006	4	1	0.0109	0.0823	3	1	0.0125	0.0867
		CH2	2	8	0.0106	0.3796	22	1	0.0315	0.2460	3	1	0.0153	0.1746
		CH3	4	4	0.0039	0.1078	1	1	0.0044	0.1585	3	1	0.0020	0.1523
		LDVg	10	2	0.1702	0.1444	22	1	0.1419	0.1289	3	1	0.0433	0.1084
		LDV1	7	9	0.1465	0.1440	2	1	0.0104	0.1702	3	1	0.0289	0.1622
		LDV2	10	9	0.3163	0.1872	24	1	0.2205	0.1337	3	1	0.2131	0.1233
		Strain 1	10	10	0.2410	0.1465	5	1	0.2325	0.1457	3	1	0.2172	0.1187
		Strain 2	10	10	0.2919	0.1654	23	1	0.3287	0.1857	3	1	0.1936	0.1191
		Strain 3	10	9	0.3749	0.2301	18	1	0.4264	0.2194	3	1	0.2271	0.1299
		Strain 4	10	9	0.3499	0.2086	7	1	0.2936	0.1784	3	1	0.2519	0.1433

Data recorded 3D Accelerometer

Data analysed 3D Accelerometer

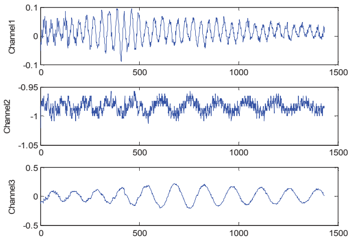

METHOD 1

CH1

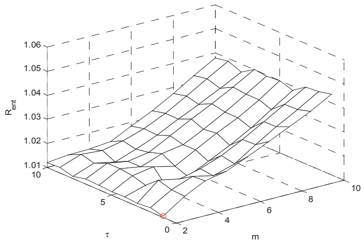

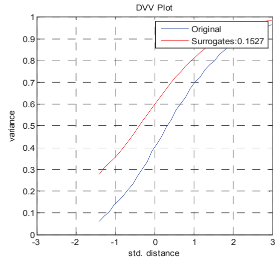

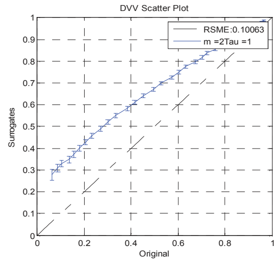

CH2

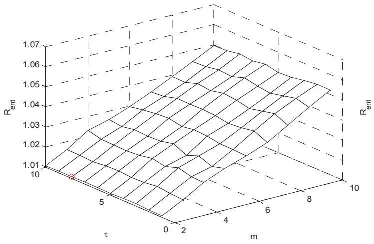

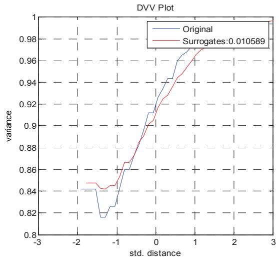

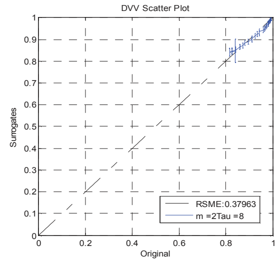

CH3

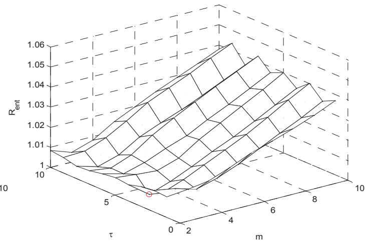

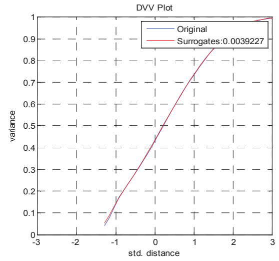

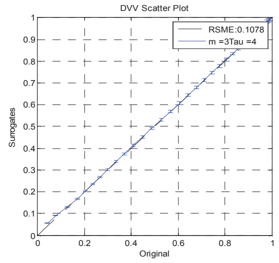

METHOD 2

CH1	CH2	CH3

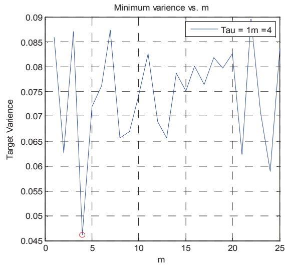

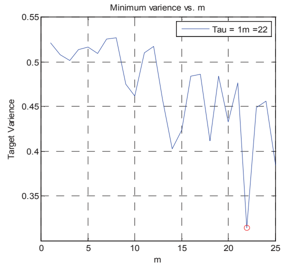

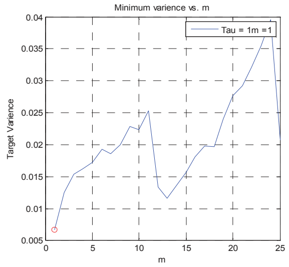

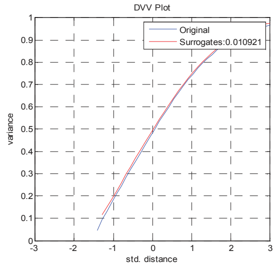

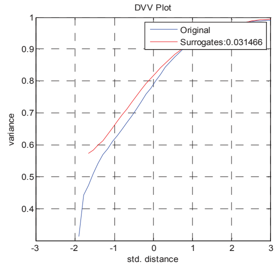

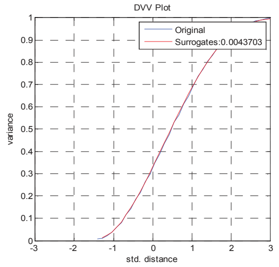

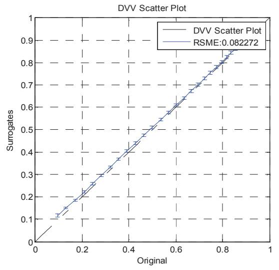

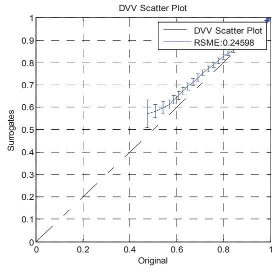

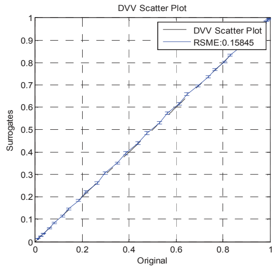

METHOD 3

CH1	CH2	CH3

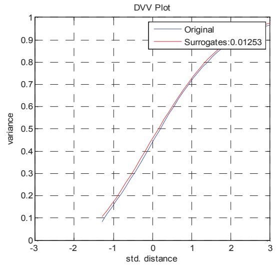

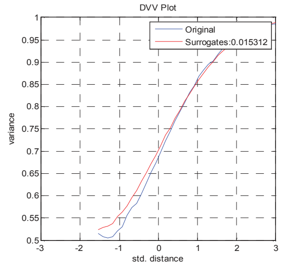

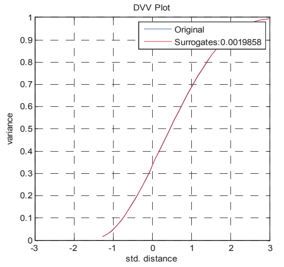

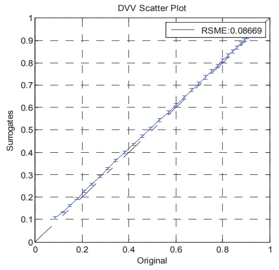

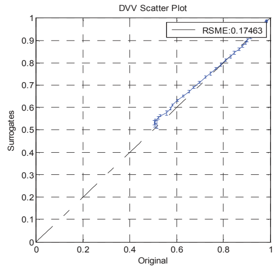

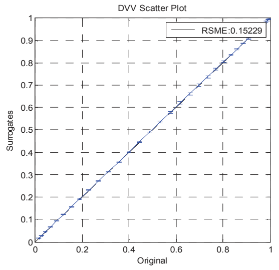

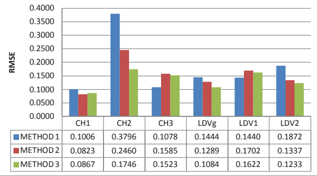

	CH1	CH2	CH3	LDVg	LDV1	LDV2
METHOD 1	0.1006	0.3796	0.1078	0.1444	0.1440	0.1872
METHOD 2	0.0823	0.2460	0.1585	0.1289	0.1702	0.1337
METHOD 3	0.0867	0.1746	0.1523	0.1084	0.1622	0.1233

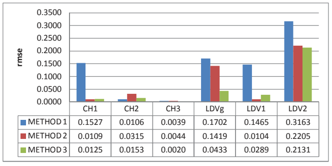

	CH1	CH2	CH3	LDVg	LDV1	LDV2
METHOD 1	0.1527	0.0106	0.0039	0.1702	0.1465	0.3163
METHOD 2	0.0109	0.0315	0.0044	0.1419	0.0104	0.2205
METHOD 3	0.0125	0.0153	0.0020	0.0433	0.0289	0.2131

Data recorded LDV

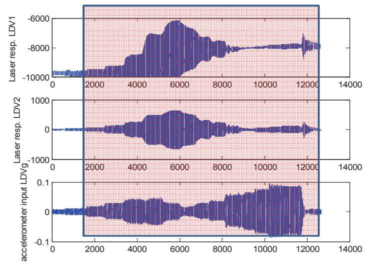

Data analysed LDV

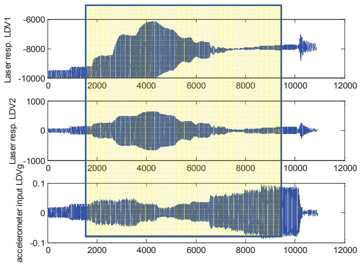

LDVg

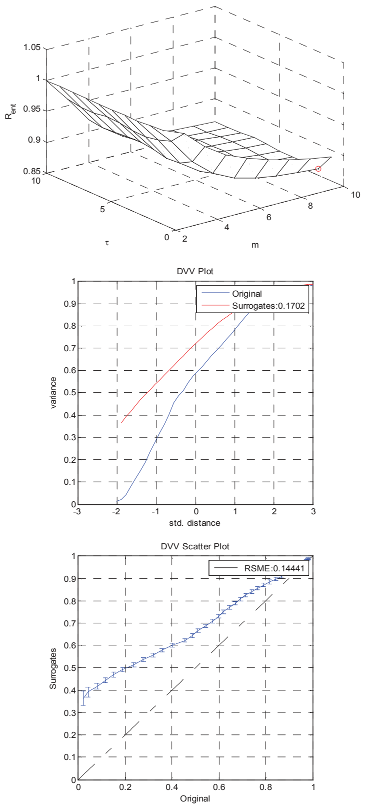

LDV1

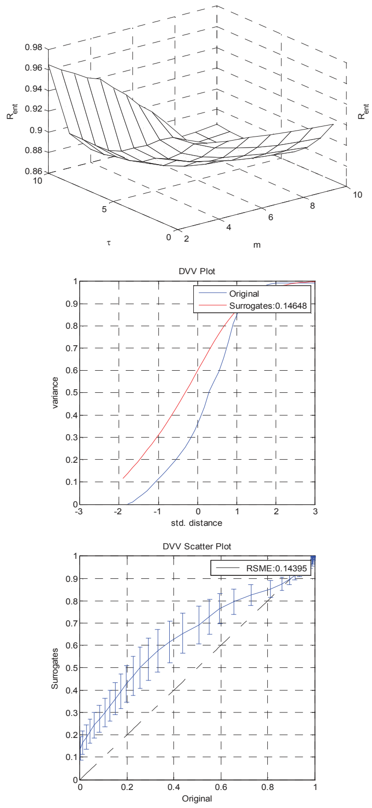

LDV2

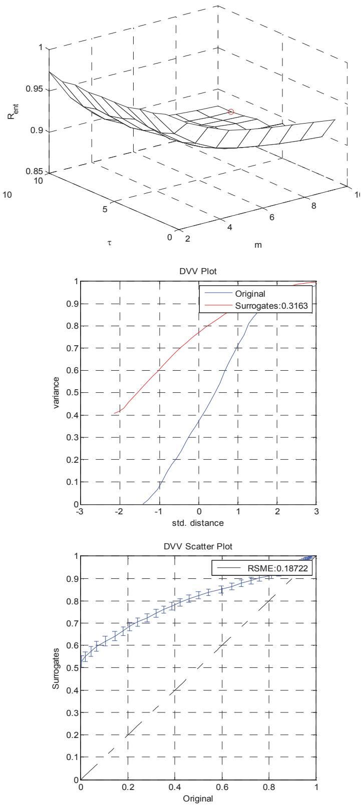

LDVg | **LDV1** | **LDV2**

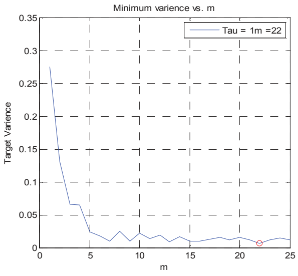

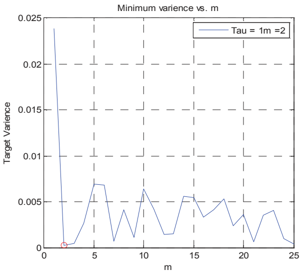

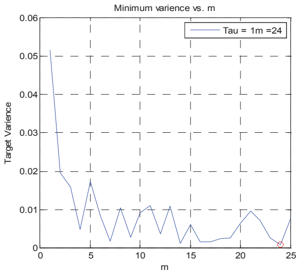

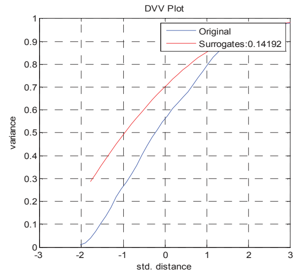

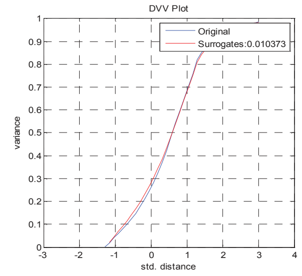

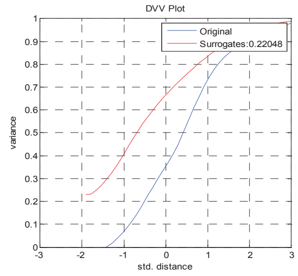

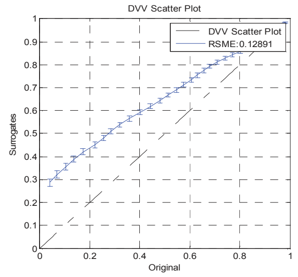

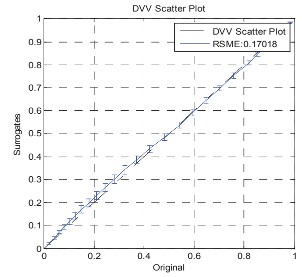

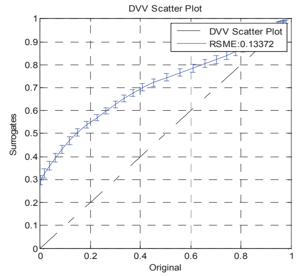

LDVg | **LDV1** | **LDV2**

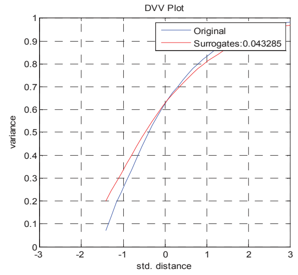

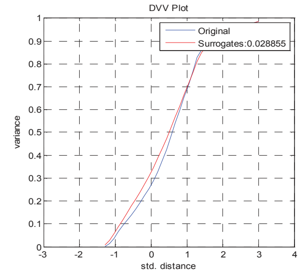

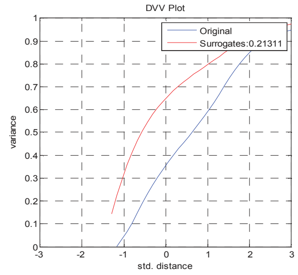

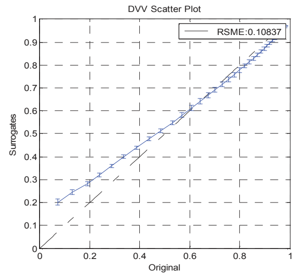

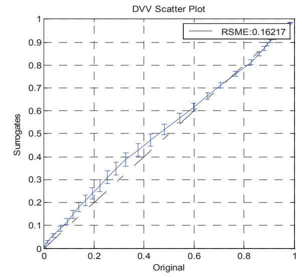

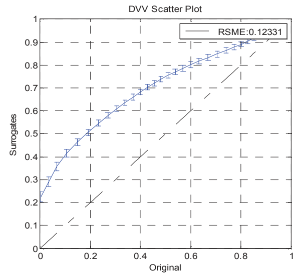

Data recorded Strain Gauge

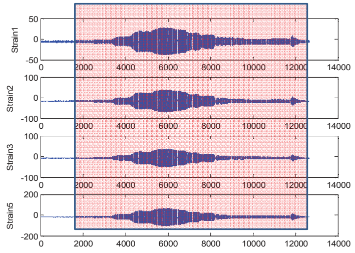

Data analysed Strain Gauge

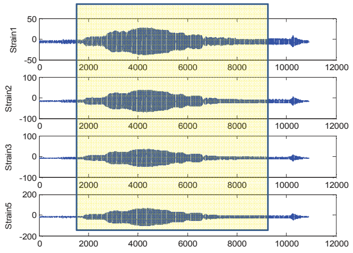

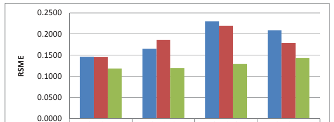

	Strain 1	Strain 2	Strain 3	Strain 4
METHOD 1	0.1465	0.1654	0.2301	0.2086
METHOD 2	0.1457	0.1857	0.2194	0.1784
METHOD 3	0.1187	0.1191	0.1299	0.1433

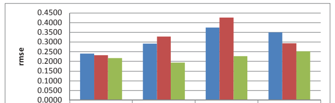

	Strain 1	Strain 2	Strain 3	Strain 4
METHOD 1	0.2410	0.2919	0.3749	0.3499
METHOD 2	0.2325	0.3287	0.4264	0.2936
METHOD 3	0.2172	0.1936	0.2271	0.2519

STRAIN 1 **STRAIN 2** **STRAIN 3** **STRAIN 4**

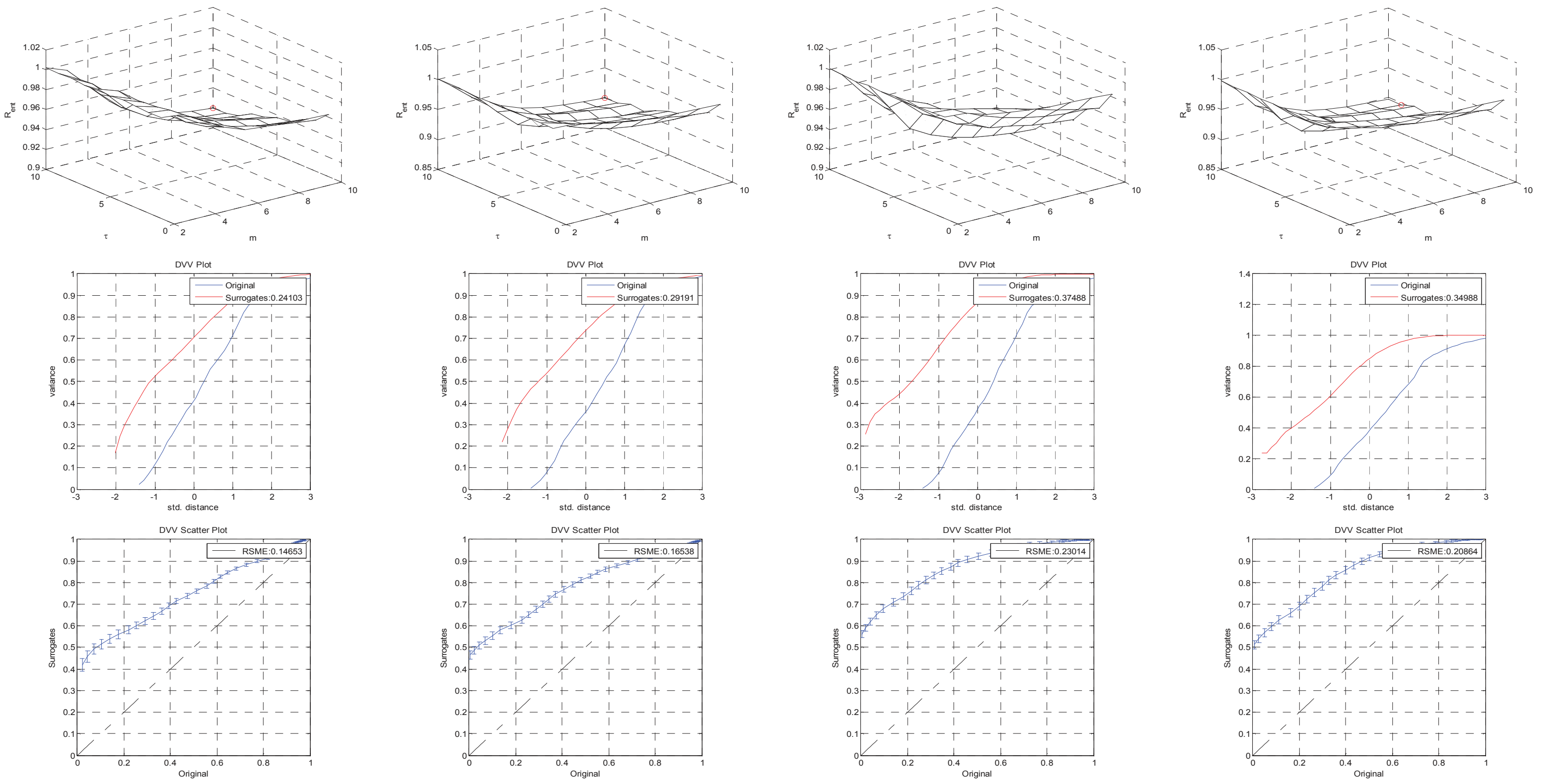

STRAIN 1

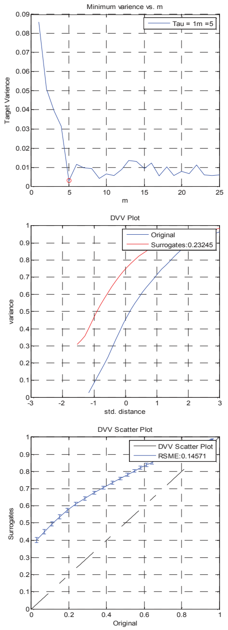

STRAIN 2

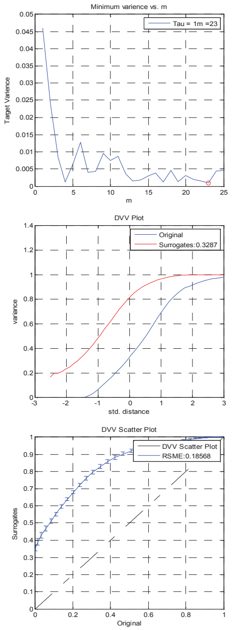

STRAIN 3

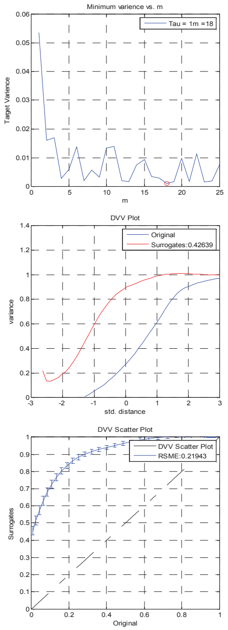

STRAIN 4

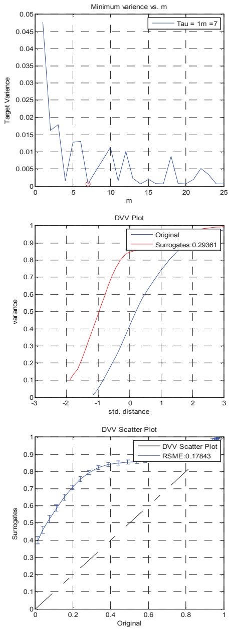

STRAIN 1

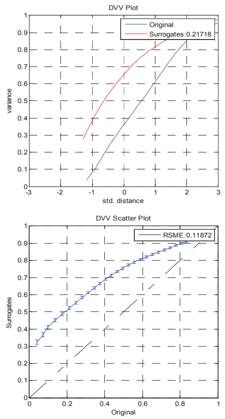

STRAIN 2

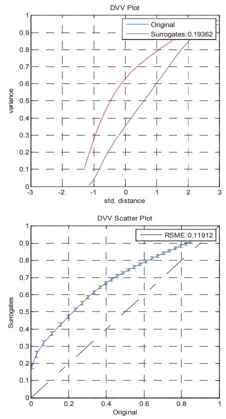

STRAIN 3

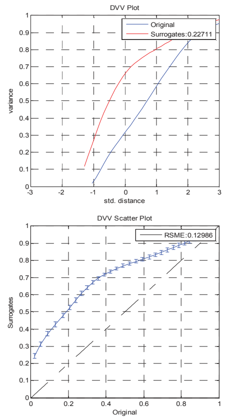

STRAIN 4

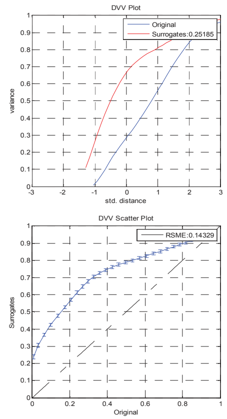

EXPERIMENT	VARIABLES		METHOD 1				METHOD 2				METHOD 3			
			best **m**	best **τ**	rsme	RSME	calc **m**	**τ**	rsme	RSME	set **m**	set **τ**	rsme	RSME
13	Loading Sine Sweep<br>2.0 Hz 6.0 Hz<br>60 sec<br>Focus at mid strain gauge	CH1	3	9	0.0425	0.2044	21	1	0.0639	0.3005	3	1	0.0241	0.2905
		CH2	8	9	0.1169	0.2192	17	1	0.0798	0.1423	3	1	0.0533	0.0738
		CH3	3	8	0.0259	0.1349	22	1	0.0196	0.1741	3	1	0.0227	0.2591
		LDVg	10	2	0.1912	0.1799	7	1	0.1105	0.1670	3	1	0.0619	0.1380
		LDV1	6	6	0.1866	0.2810	5	1	0.0367	0.3386	3	1	0.0668	0.3511
		LDV2	7	7	0.0617	0.2329	24	1	0.5283	0.3078	3	1	0.4848	0.3156
		Strain 1	10	10	0.0882	0.1039	22	1	0.4030	0.2106	3	1	0.2604	0.1358
		Strain 2	9	9	0.0766	0.1423	7	1	0.2112	0.1369	3	1	0.2040	0.1564
		Strain 3	10	9	0.3634	0.1895	5	1	0.2536	0.1320	3	1	0.2723	0.1620
		Strain 4	9	9	0.3700	0.1903	4	1	0.2454	0.1417	3	1	0.2522	0.1643

Data recorded 3D Accelerometer

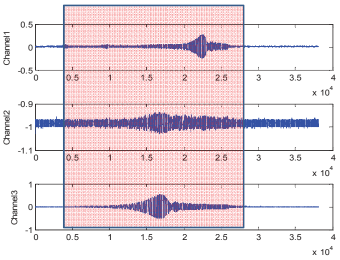

Data analysed 3D Accelerometer

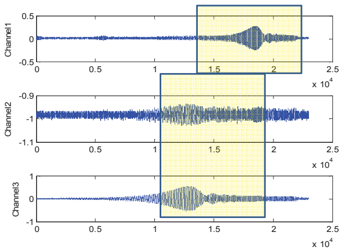

METHOD 1

CH1

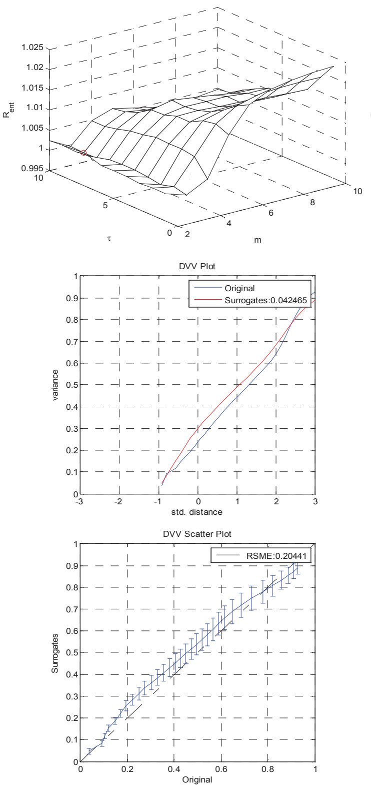

CH2

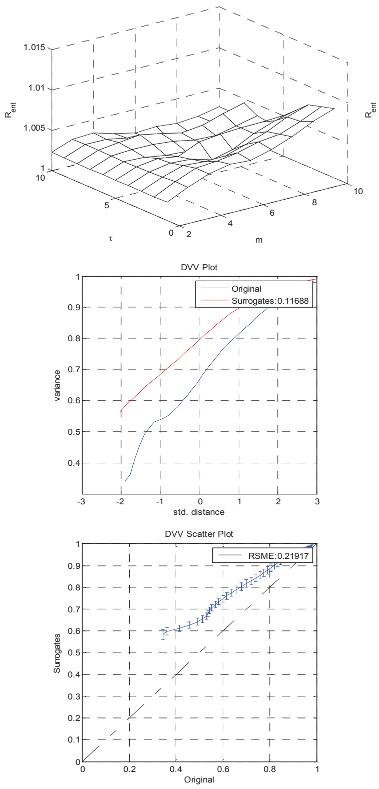

CH3

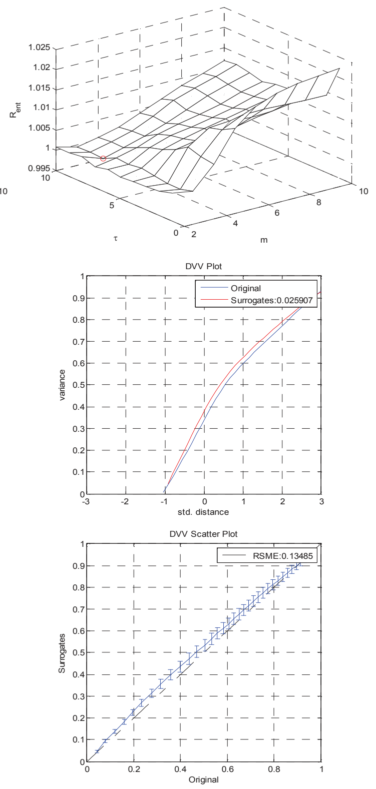

METHOD 2

CH1

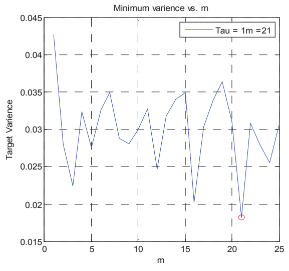

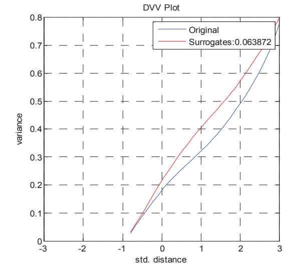

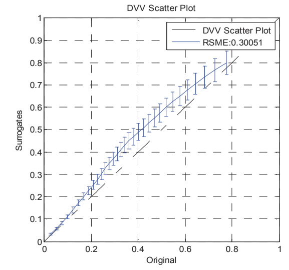

CH2

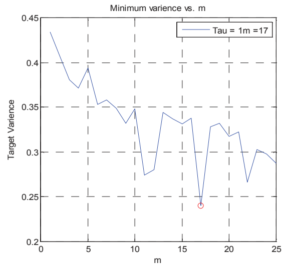

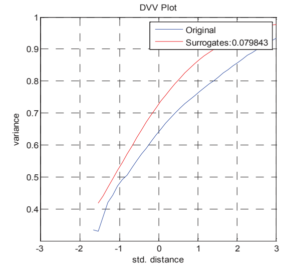

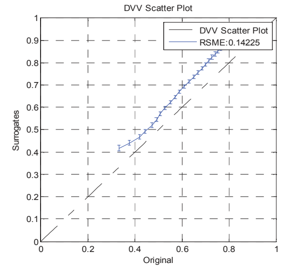

CH3

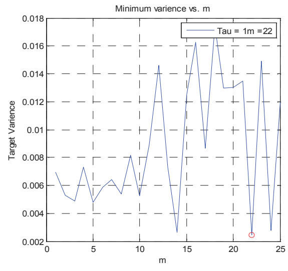

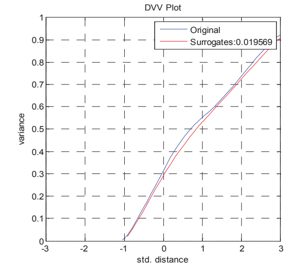

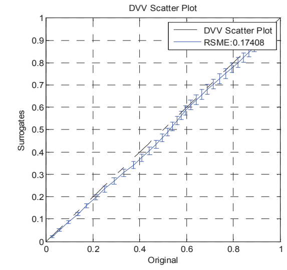

METHOD 3

CH1

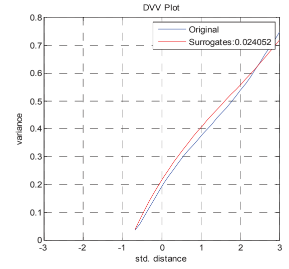

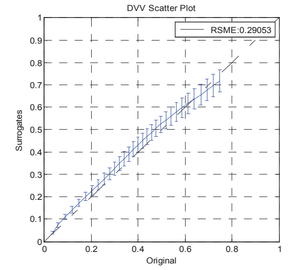

CH2

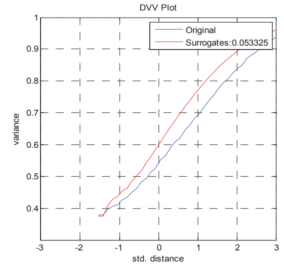

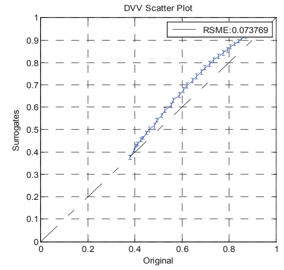

CH3

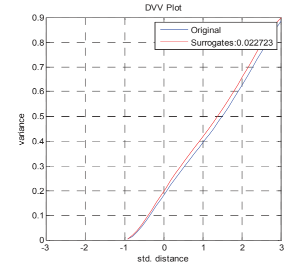

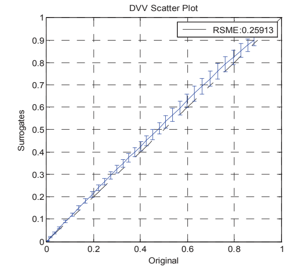

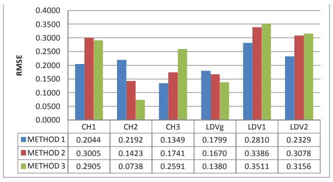

	CH1	CH2	CH3	LDVg	LDV1	LDV2
METHOD 1	0.2044	0.2192	0.1349	0.1799	0.2810	0.2329
METHOD 2	0.3005	0.1423	0.1741	0.1670	0.3386	0.3078
METHOD 3	0.2905	0.0738	0.2591	0.1380	0.3511	0.3156

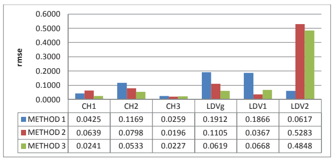

	CH1	CH2	CH3	LDVg	LDV1	LDV2
METHOD 1	0.0425	0.1169	0.0259	0.1912	0.1866	0.0617
METHOD 2	0.0639	0.0798	0.0196	0.1105	0.0367	0.5283
METHOD 3	0.0241	0.0533	0.0227	0.0619	0.0668	0.4848

Data recorded LDV ?

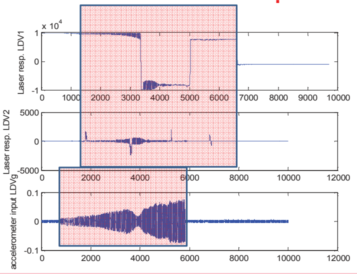

Data analysed LDV

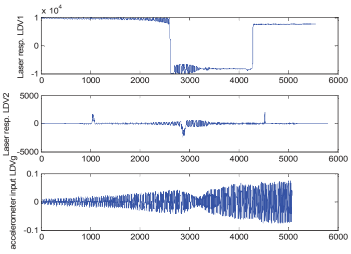

LDVg **LDV1** **LDV2**

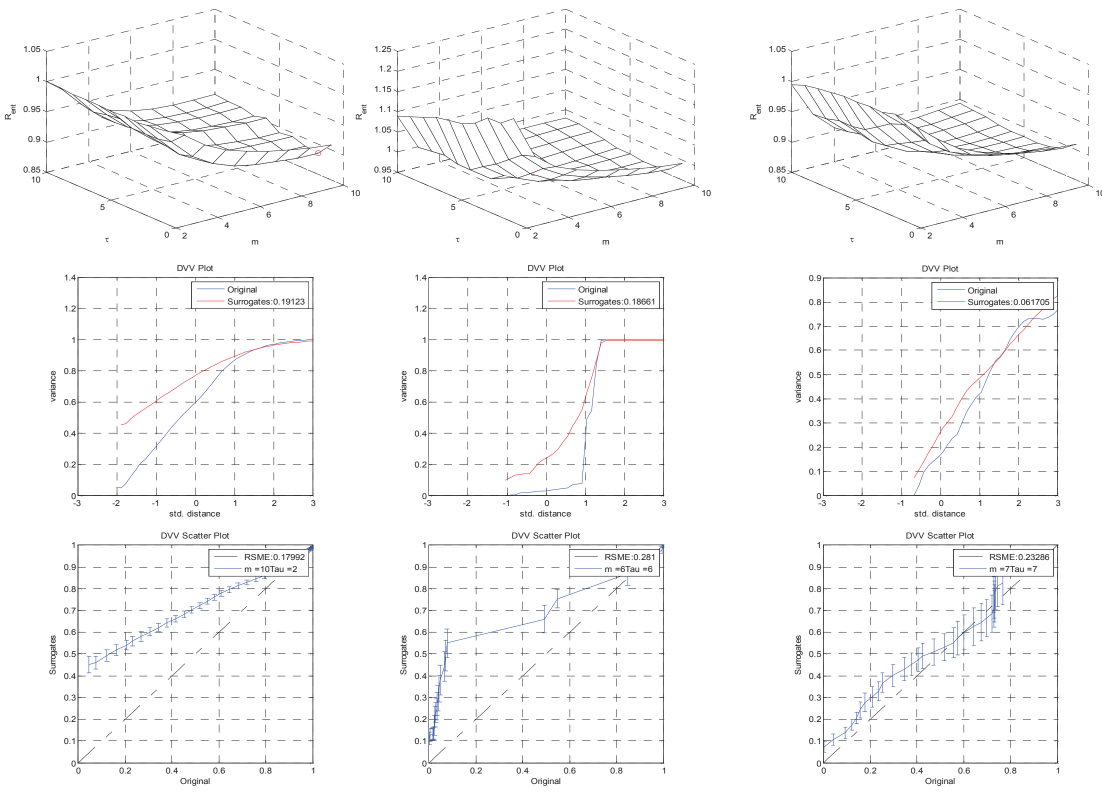

LDVg LDV1 LDV2

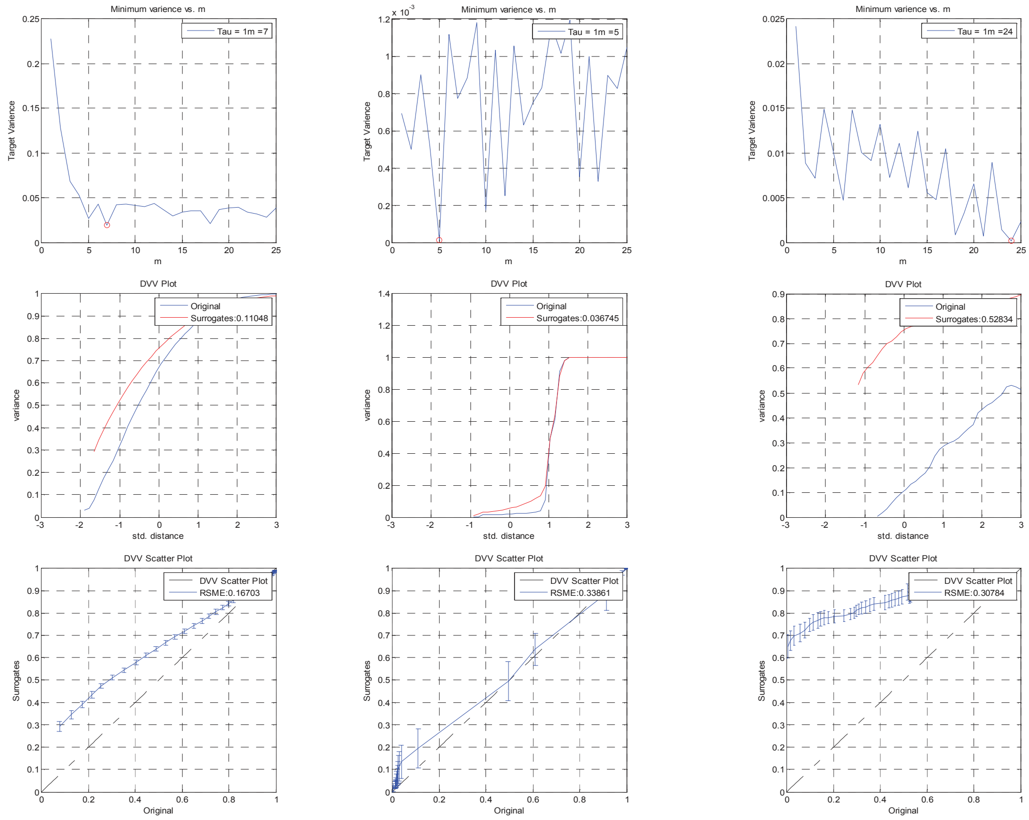

LDVg LDV1 LDV2

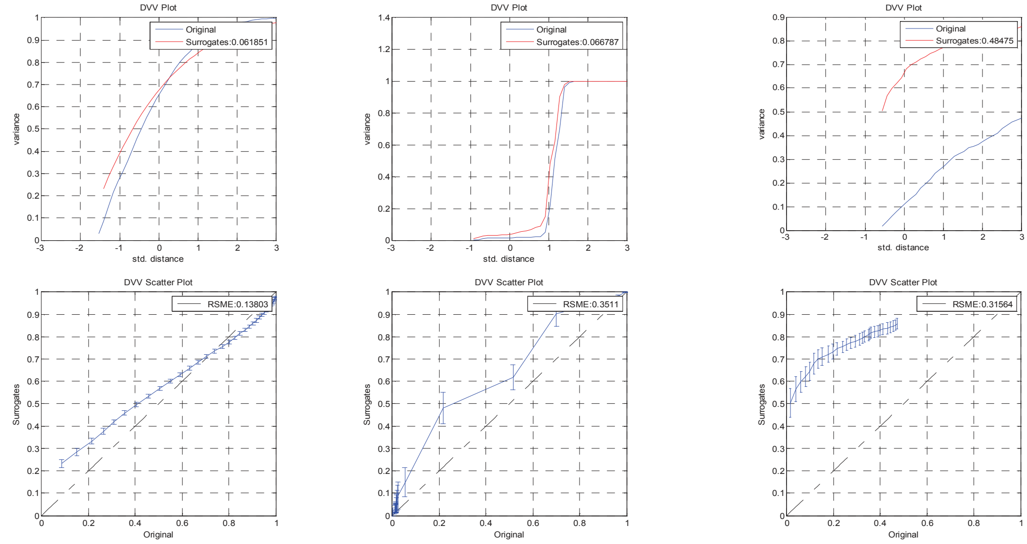

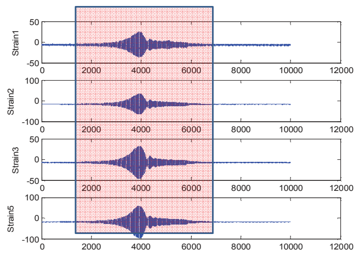

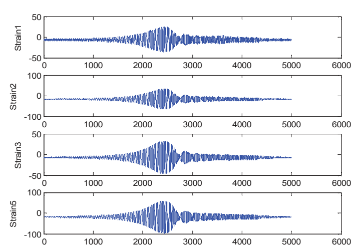

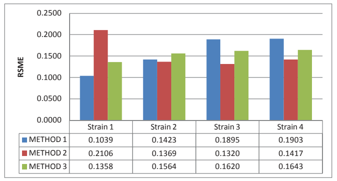

	Strain 1	Strain 2	Strain 3	Strain 4
METHOD 1	0.1039	0.1423	0.1895	0.1903
METHOD 2	0.2106	0.1369	0.1320	0.1417
METHOD 3	0.1358	0.1564	0.1620	0.1643

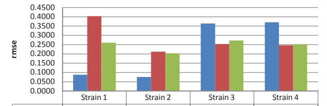

	Strain 1	Strain 2	Strain 3	Strain 4
METHOD 1	0.0882	0.0766	0.3634	0.3700
METHOD 2	0.4030	0.2112	0.2536	0.2454
METHOD 3	0.2604	0.2040	0.2723	0.2522

STRAIN 1

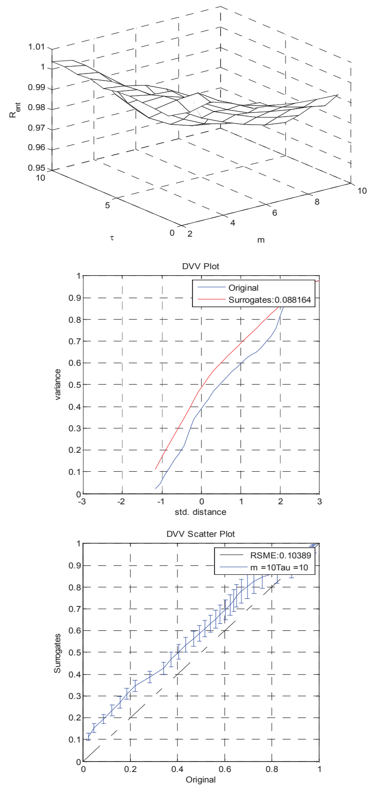

STRAIN 2

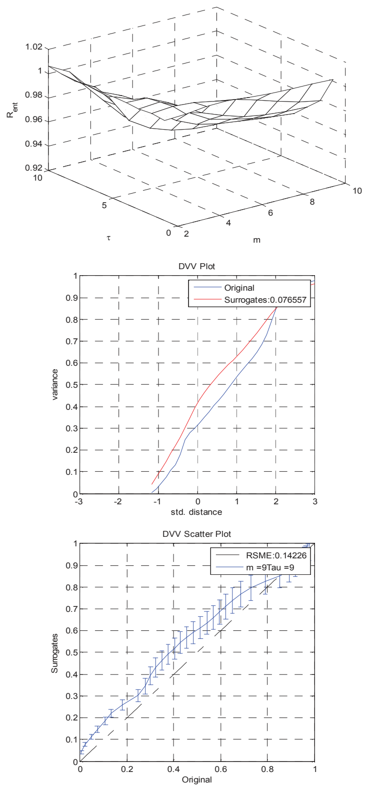

STRAIN 3

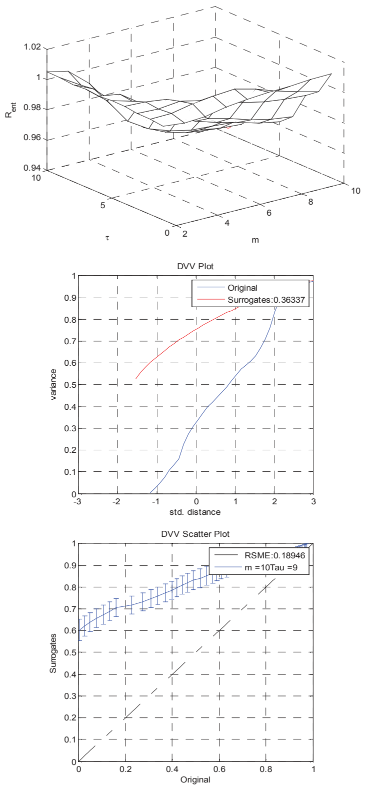

STRAIN 4

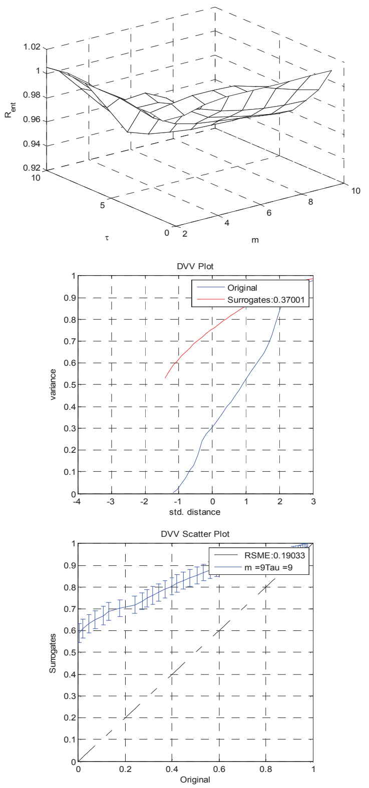

STRAIN 1

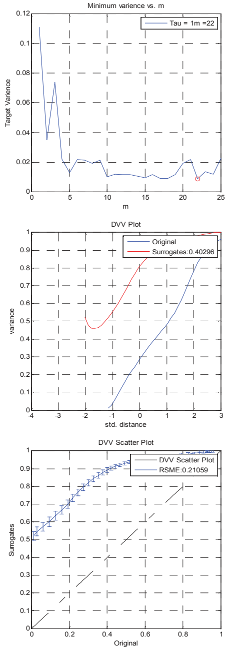

STRAIN 2

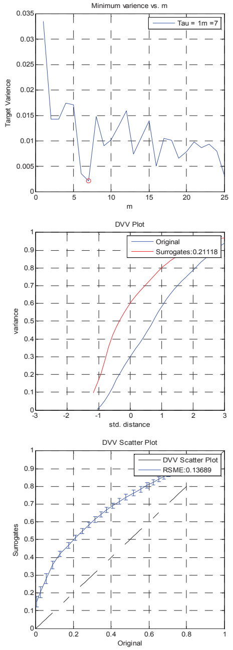

STRAIN 3

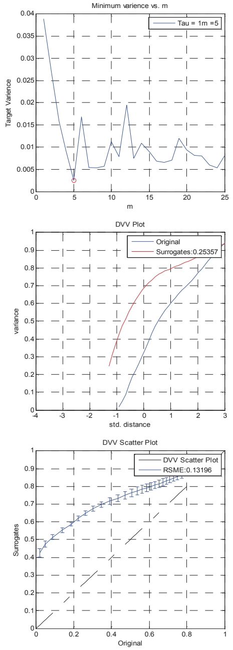

STRAIN 4

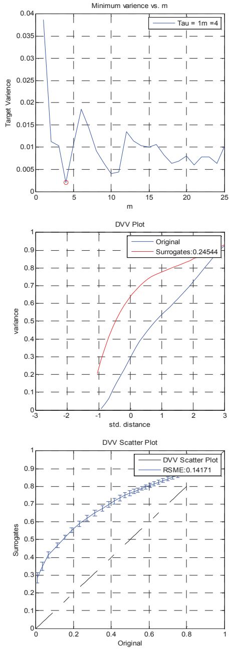

STRAIN 1

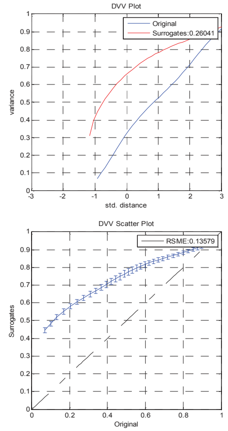

STRAIN 2

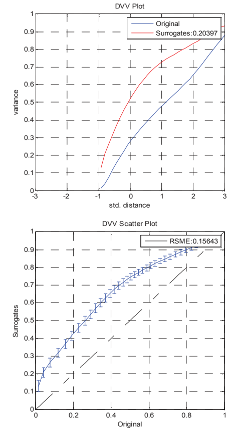

STRAIN 3

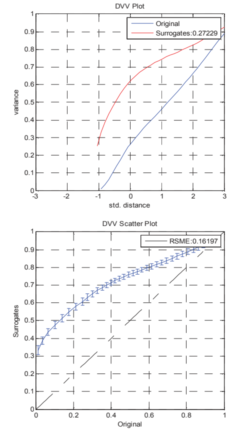

STRAIN 4

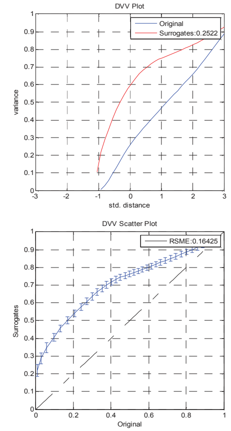

EXPERIMENT	VARIABLES		METHOD 1				METHOD 2				METHOD 3			
			best **m**	best **τ**	rsme	RSME	calc **m**	**τ**	rsme	RSME	set **m**	set **τ**	rsme	RSME
14	Loading White noise<br>4.336Hz at the peak<br>next 23Hz<br>Focus at mid strain gauge	CH1	5	1	0.0072	0.0756	24	1	0.0156	0.0853	3	1	0.0080	0.0732
		CH2	5	1	0.0372	0.1616	14	1	0.0489	0.1552	3	1	0.0277	0.1218
		CH3	4	1	0.0190	0.1089	21	1	0.0537	0.1977	3	1	0.0153	0.0961
		LDVg	8	1	0.1692	0.3932	25	1	0.0381	0.4272	3	1	0.0524	0.2549
		LDV1	2	2	0.0030	0.2366	3	1	0.0044	0.1145	3	1	0.0019	0.1134
		LDV2	2	4	0.1031	0.3799	4	1	0.2186	0.2779	3	1	0.2233	0.2656
		Strain 1	2	5	0.0023	0.3227	18	1	0.2176	0.4070	3	1	0.2059	0.3447
		Strain 2	3	5	0.0068	0.2992	22	1	0.0866	0.2729	3	1	0.0637	0.2257
		Strain 3	2	9	0.2249	0.3144	25	1	0.1299	0.1882	3	1	0.1149	0.1277
		Strain 4	7	2	0.2378	0.2272	18	1	0.0800	0.1192	3	1	0.0721	0.0910

Data recorded 3D Accelerometer

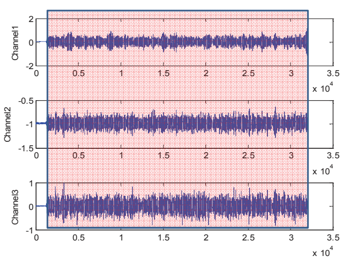

Data analysed 3D Accelerometer

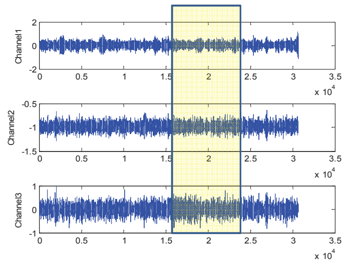

METHOD 1

CH1

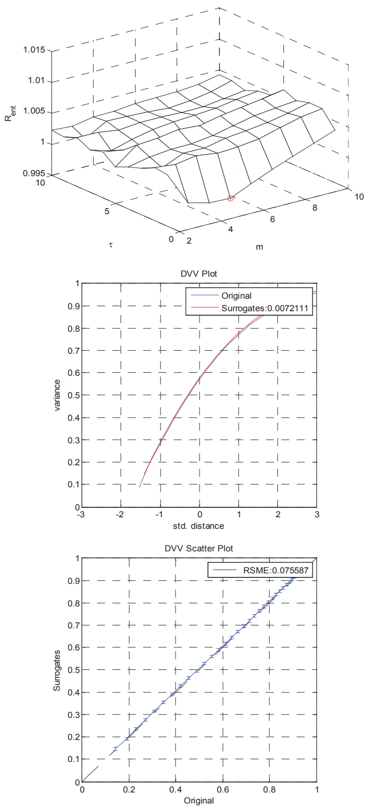

CH2

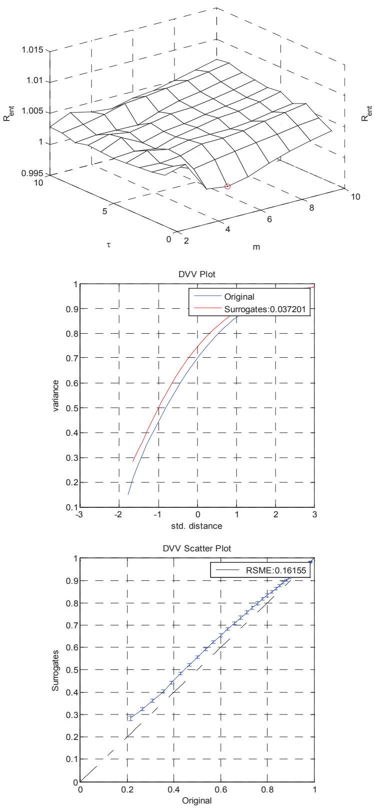

CH3

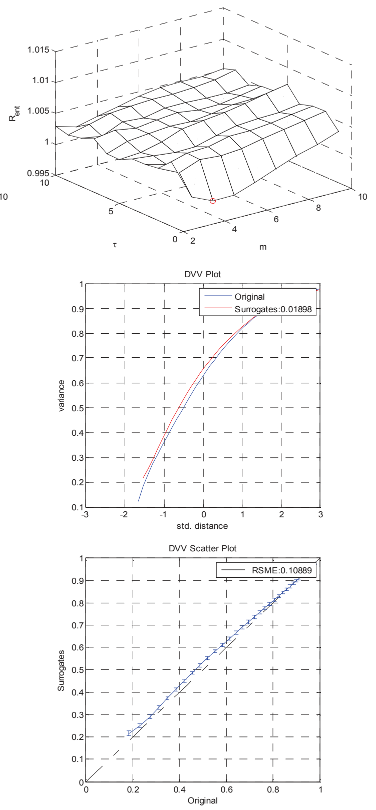

METHOD 2

CH1 **CH2** **CH3**

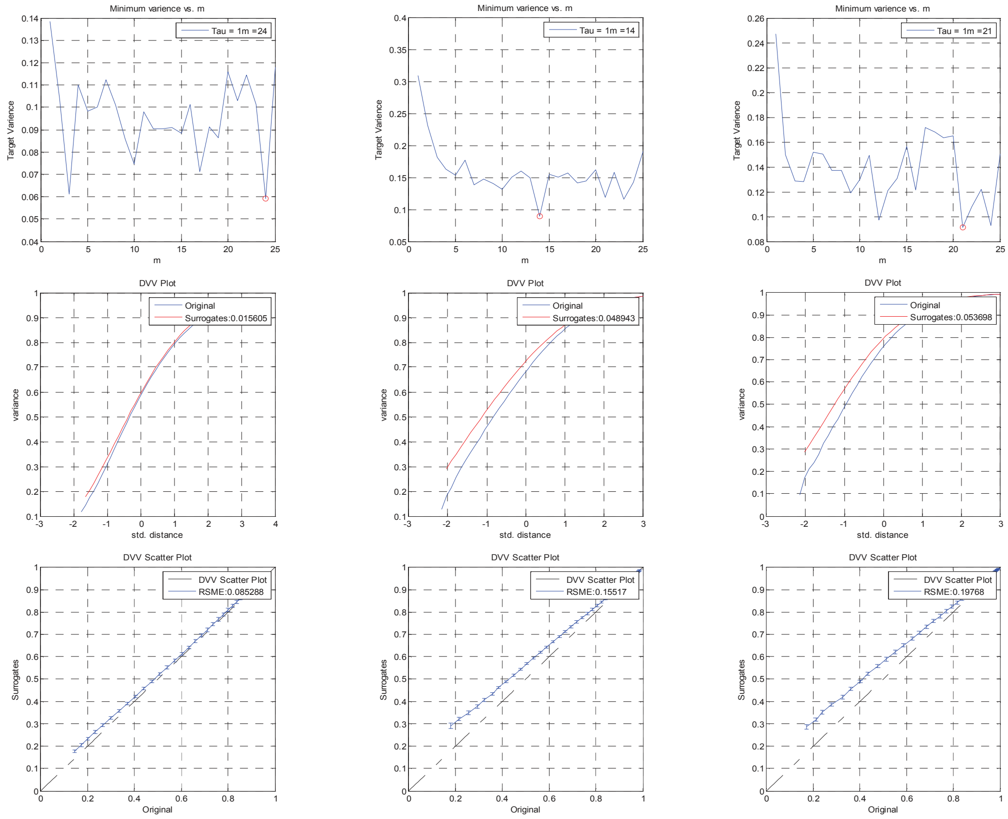

METHOD 3

CH1 **CH2** **CH3**

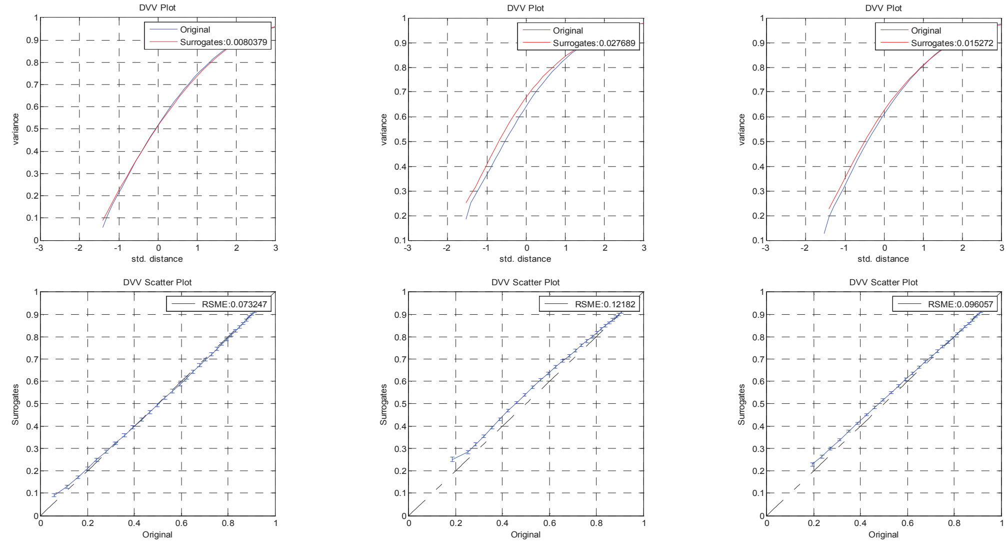

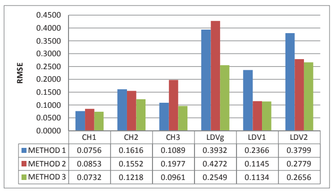

	CH1	CH2	CH3	LDVg	LDV1	LDV2
METHOD 1	0.0756	0.1616	0.1089	0.3932	0.2366	0.3799
METHOD 2	0.0853	0.1552	0.1977	0.4272	0.1145	0.2779
METHOD 3	0.0732	0.1218	0.0961	0.2549	0.1134	0.2656

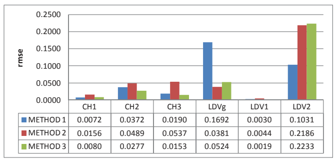

	CH1	CH2	CH3	LDVg	LDV1	LDV2
METHOD 1	0.0072	0.0372	0.0190	0.1692	0.0030	0.1031
METHOD 2	0.0156	0.0489	0.0537	0.0381	0.0044	0.2186
METHOD 3	0.0080	0.0277	0.0153	0.0524	0.0019	0.2233

Data recorded LDV

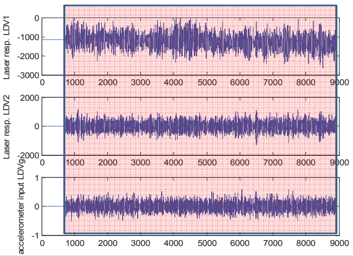

Data analysed LDV

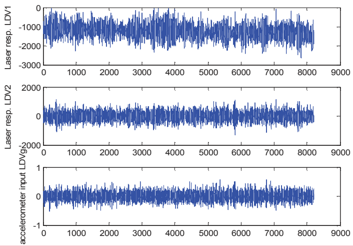

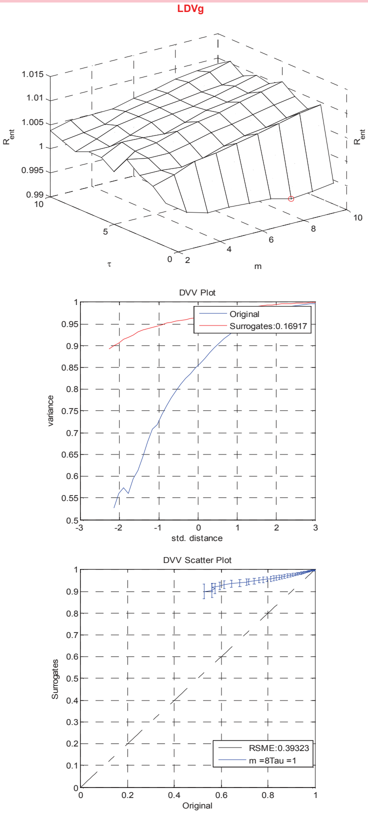

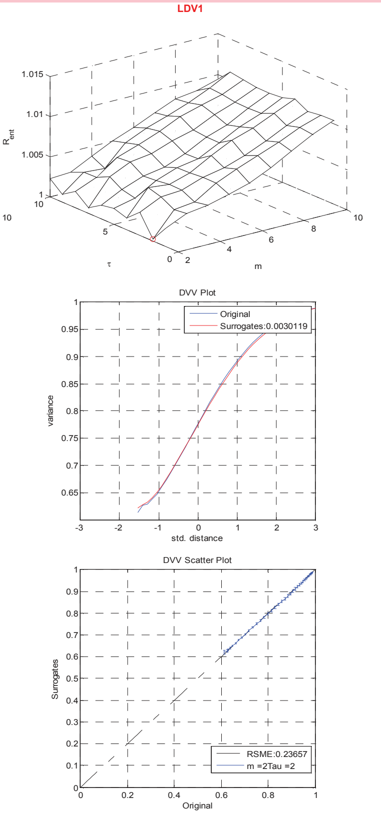

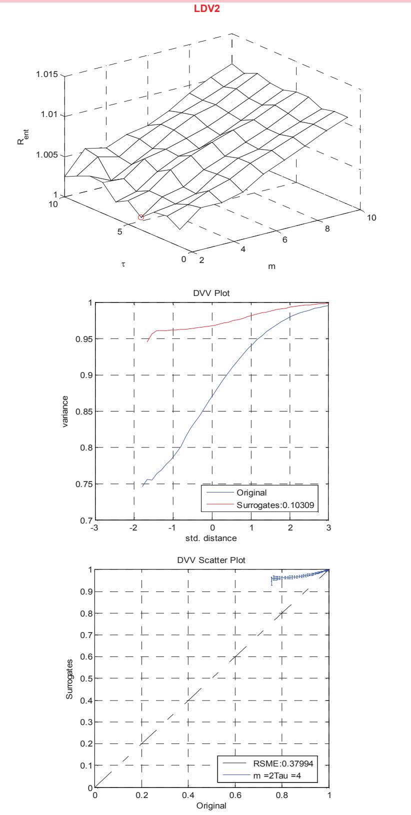

LDVg **LDV1** **LDV2**

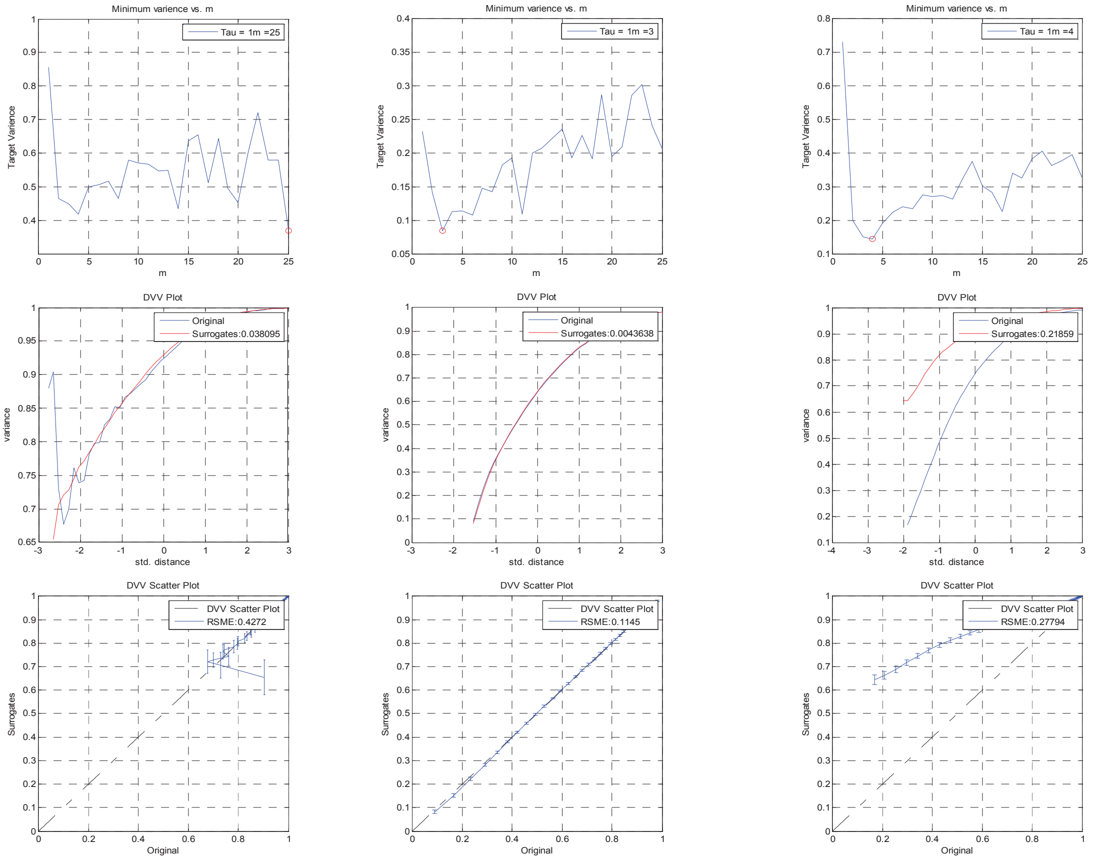

LDVg **LDV1** **LDV2**

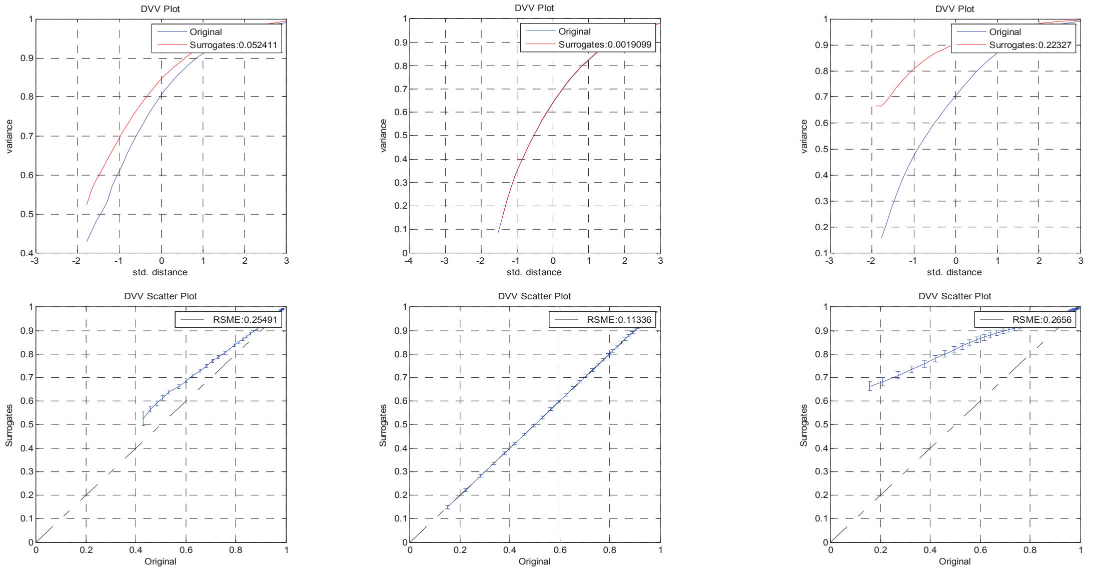

Data recorded Strain Gauge

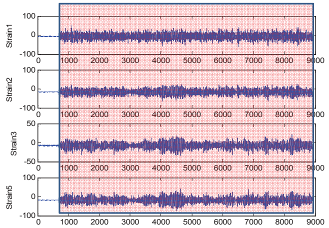

Data analysed Strain Gauge

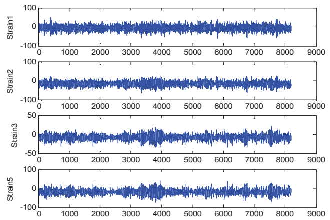

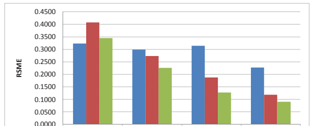

	Strain 1	Strain 2	Strain 3	Strain 4
METHOD 1	0.3227	0.2992	0.3144	0.2272
METHOD 2	0.4070	0.2729	0.1882	0.1192
METHOD 3	0.3447	0.2257	0.1277	0.0910

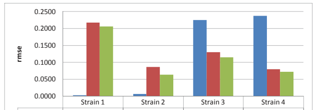

	Strain 1	Strain 2	Strain 3	Strain 4
METHOD 1	0.0023	0.0068	0.2249	0.2378
METHOD 2	0.2176	0.0866	0.1299	0.0800
METHOD 3	0.2059	0.0637	0.1149	0.0721

STRAIN 1

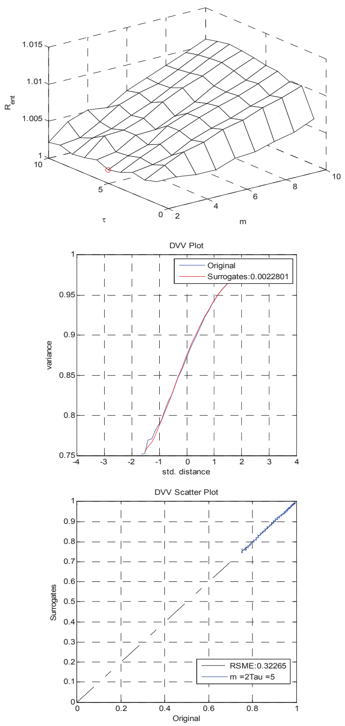

STRAIN 2

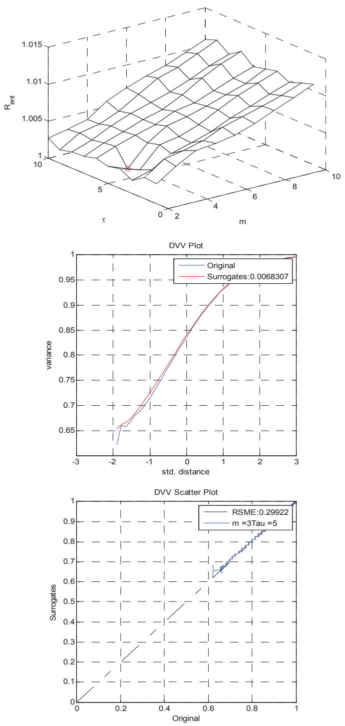

STRAIN 3

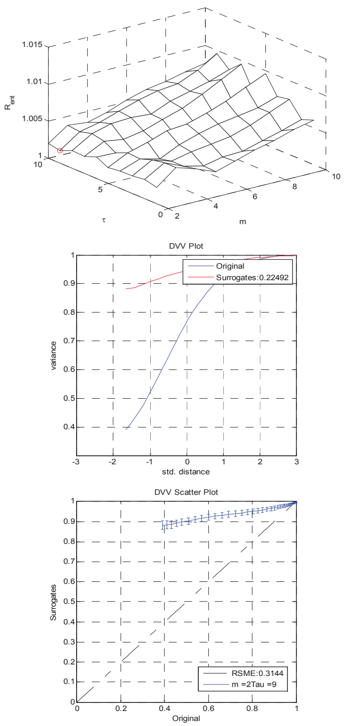

STRAIN 4

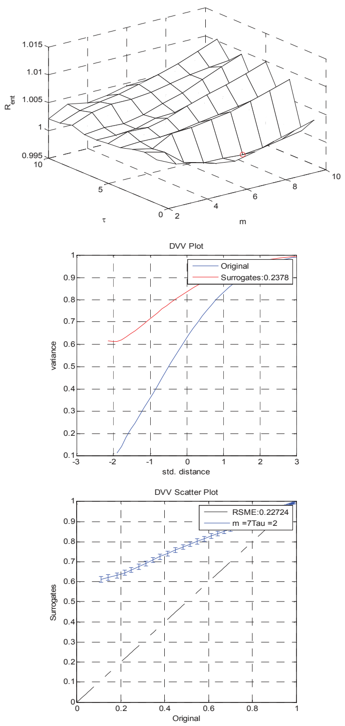

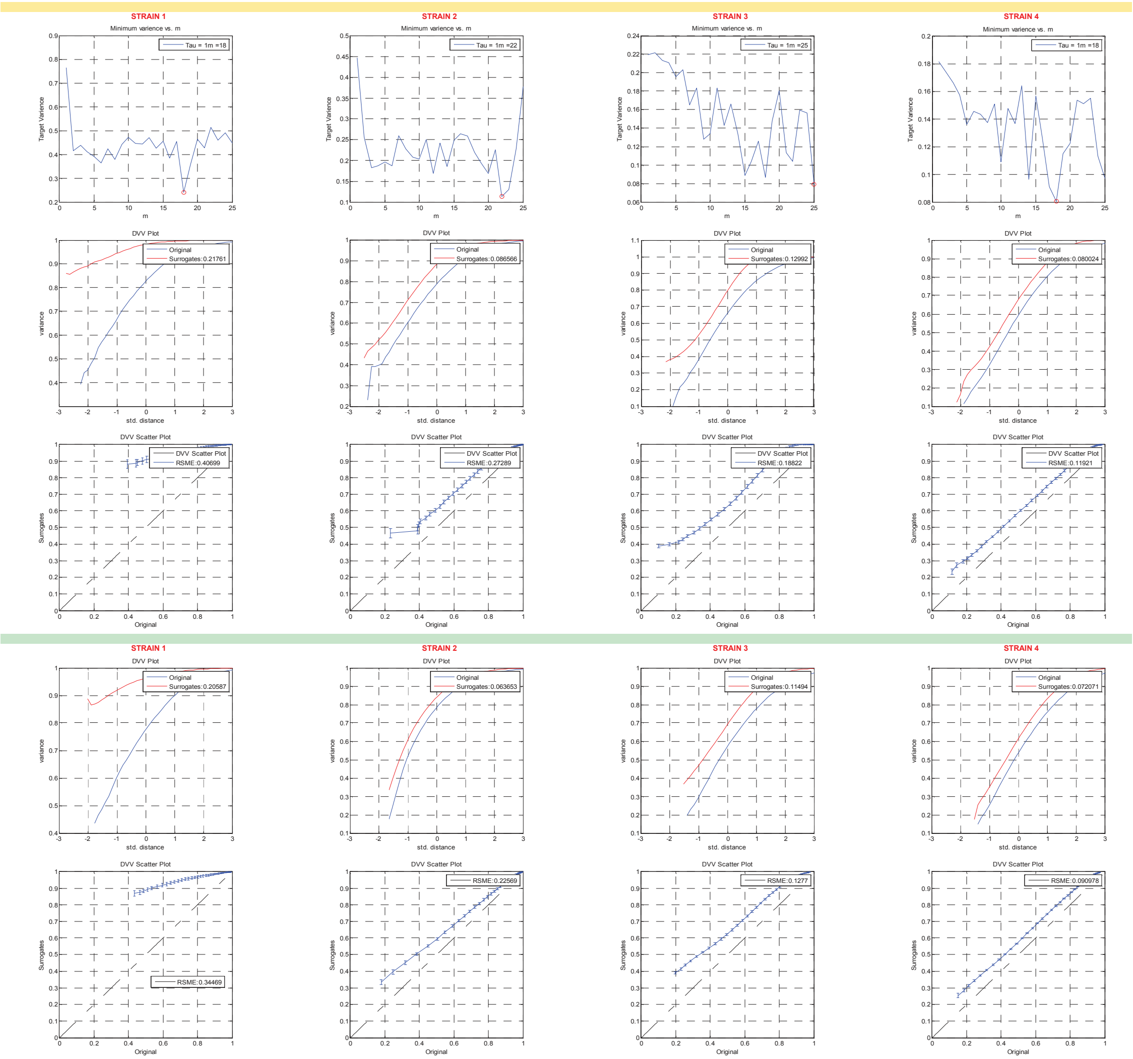
STRAIN 1
Minimum varience vs. m
Tau = 1m =18
Target Varience
m
DVV Plot
Original
Surrogates:0.21761
variance
std. distance
DVV Scatter Plot
RSME:0.40699
Surrogates
Original
STRAIN 2
Minimum varience vs. m
Tau = 1m =22
Surrogates:0.086566
RSME:0.27289
STRAIN 3
Tau = 1m =25
Surrogates:0.12992
RSME:0.18822
STRAIN 4
Tau = 1m =18
Surrogates:0.080024
RSME:0.11921
STRAIN 1
Surrogates:0.20587
RSME:0.34469
STRAIN 2
Surrogates:0.063653
RSME:0.22569
STRAIN 3
Surrogates:0.11494
RSME:0.1277
STRAIN 4
Surrogates:0.072071
RSME:0.090978

EXPERIMENT	VARIABLES		METHOD 1				METHOD 2				METHOD 3			
			best **m**	best τ	rsme	RSME	calc **m**	τ	rsme	RSME	set **m**	set τ	rsme	RSME
15	Loading White noise 4.336Hz at the peak next 23Hz Focus at bottom strain gauge	CH1	4	1	0.0062	0.0692	5	1	0.0066	0.0772	3	1	0.0038	0.0668
		CH2	5	1	0.0321	0.1750	22	1	0.0695	0.1675	3	1	0.0206	0.1353
		CH3	4	1	0.0192	0.1124	17	1	0.0467	0.1979	3	1	0.0168	0.0963
		LDVg	8	1	0.0505	0.3124	9	1	0.0654	0.3185	3	1	0.0442	0.2607
		LDV1	2	10	0.0086	0.1173	19	1	0.0120	0.0846	3	1	0.0041	0.1057
		LDV2	6	1	0.3039	0.3063	18	1	0.3192	0.3524	3	1	0.2591	0.2782
		Strain 1	8	1	0.3199	0.3504	14	1	0.3787	0.3699	3	1	0.2443	0.3280
		Strain 2	7	7	0.1514	0.3172	19	1	0.1445	0.2367	3	1	0.2132	0.2352
		Strain 3	10	6	0.1905	0.2279	18	1	0.1627	0.1652	3	1	0.2765	0.1664
		Strain 4	10	10	0.1453	0.2298	19	1	0.0940	0.1269	3	1	0.2801	0.1815

Data recorded 3D Accelerometer

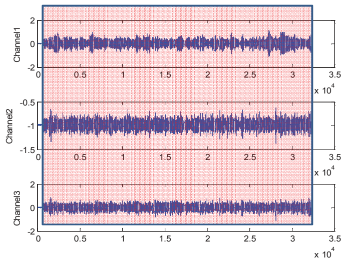

Data analysed 3D Accelerometer

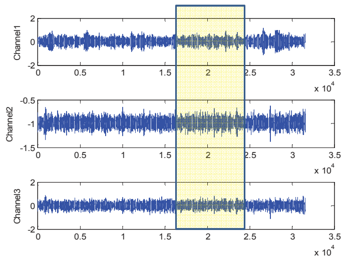

METHOD 1

CH1

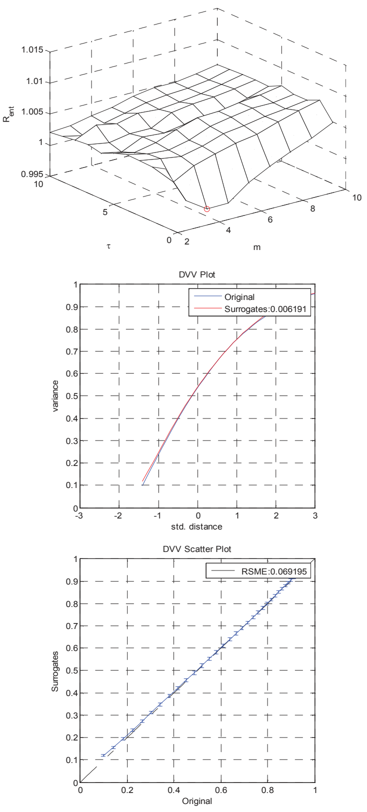

CH2

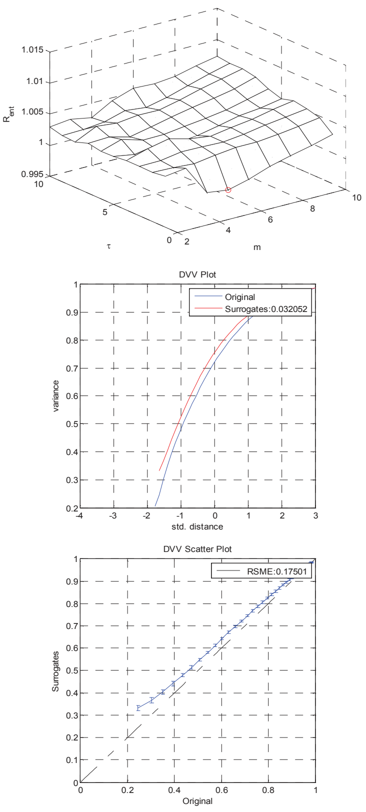

CH3

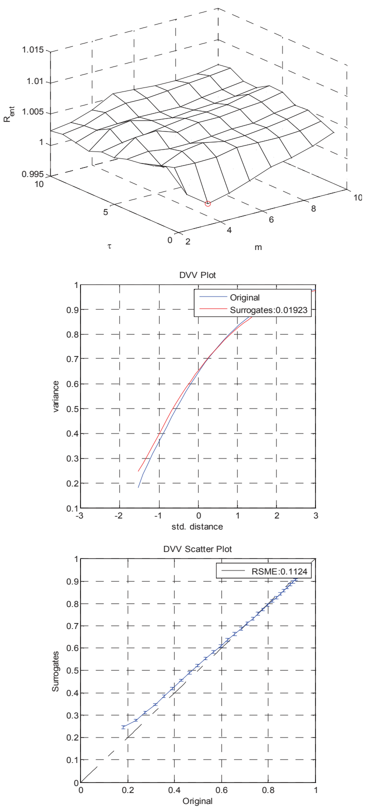

METHOD 2

CH1

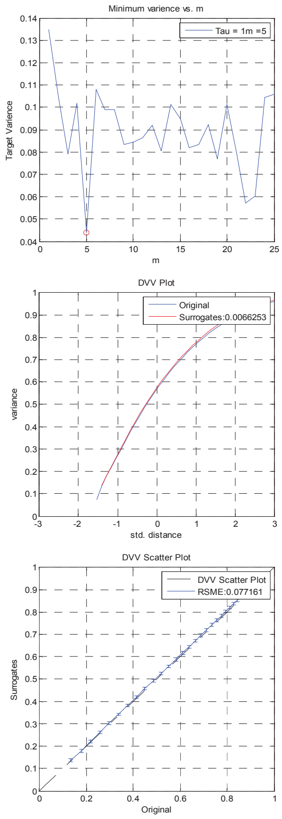

CH2

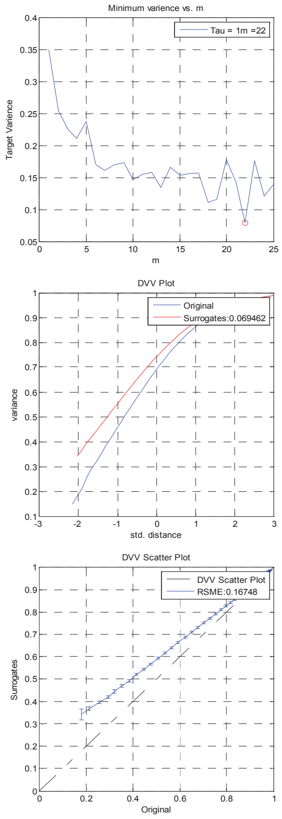

CH3

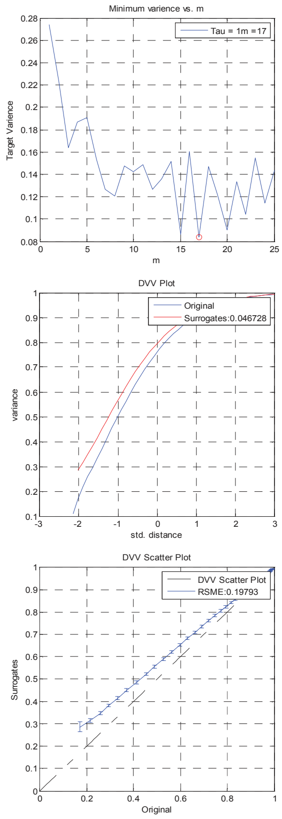

METHOD 3

CH1

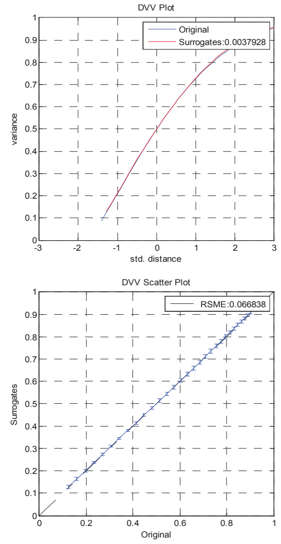

CH2

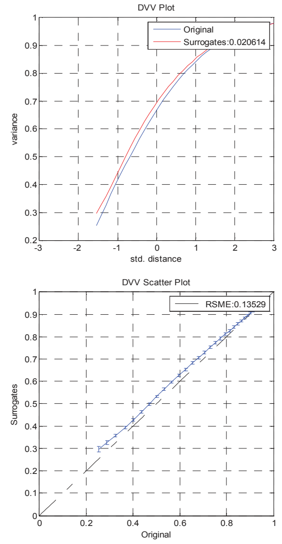

CH3

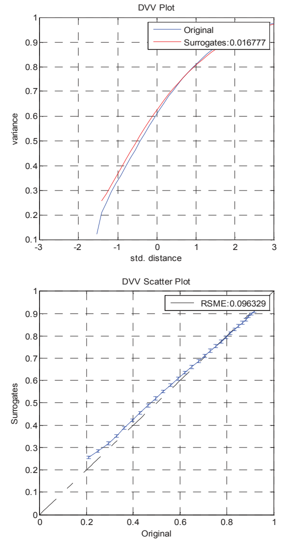

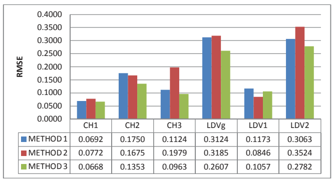

	CH1	CH2	CH3	LDVg	LDV1	LDV2
METHOD 1	0.0692	0.1750	0.1124	0.3124	0.1173	0.3063
METHOD 2	0.0772	0.1675	0.1979	0.3185	0.0846	0.3524
METHOD 3	0.0668	0.1353	0.0963	0.2607	0.1057	0.2782

Data recorded LDV

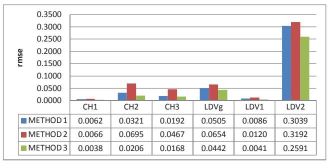

	CH1	CH2	CH3	LDVg	LDV1	LDV2
METHOD 1	0.0062	0.0321	0.0192	0.0505	0.0086	0.3039
METHOD 2	0.0066	0.0695	0.0467	0.0654	0.0120	0.3192
METHOD 3	0.0038	0.0206	0.0168	0.0442	0.0041	0.2591

Data analysed LDV

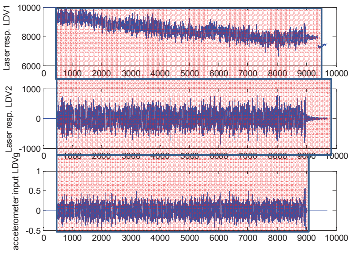

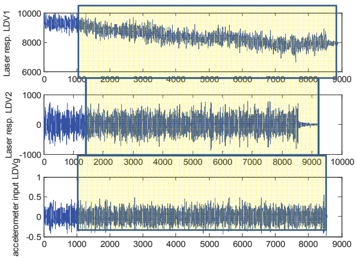

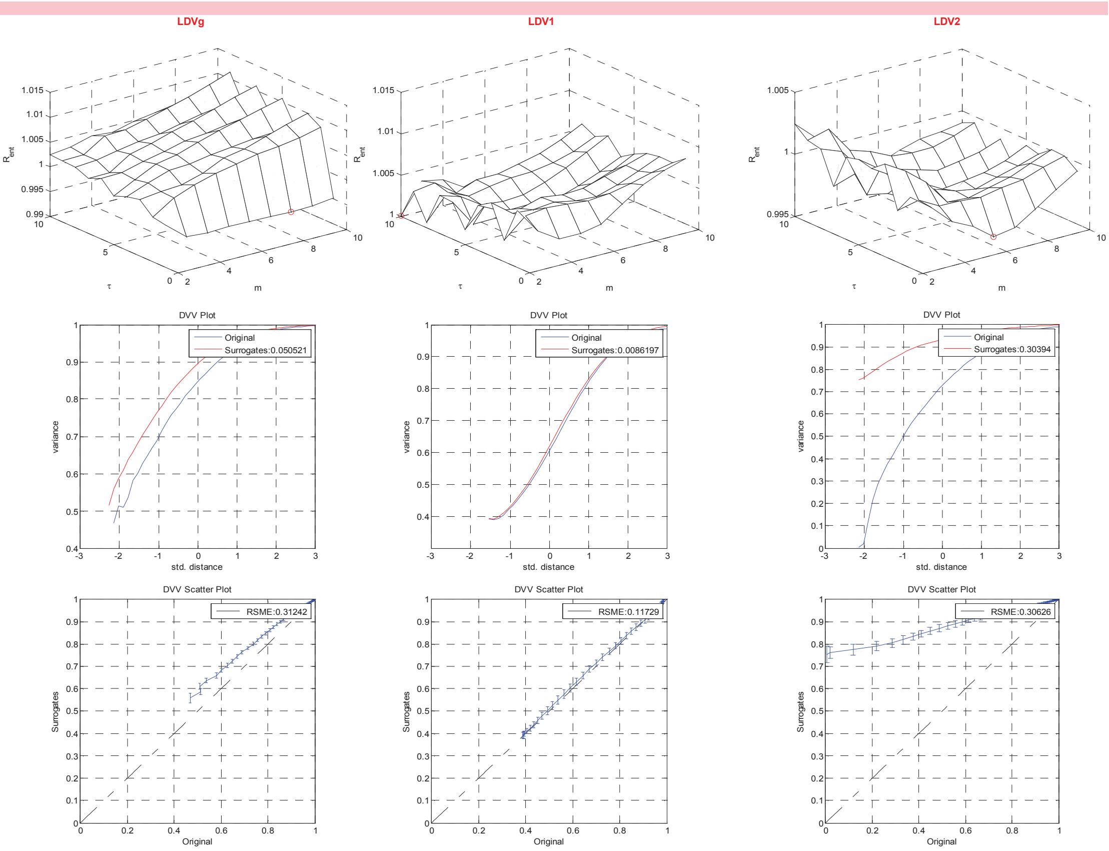

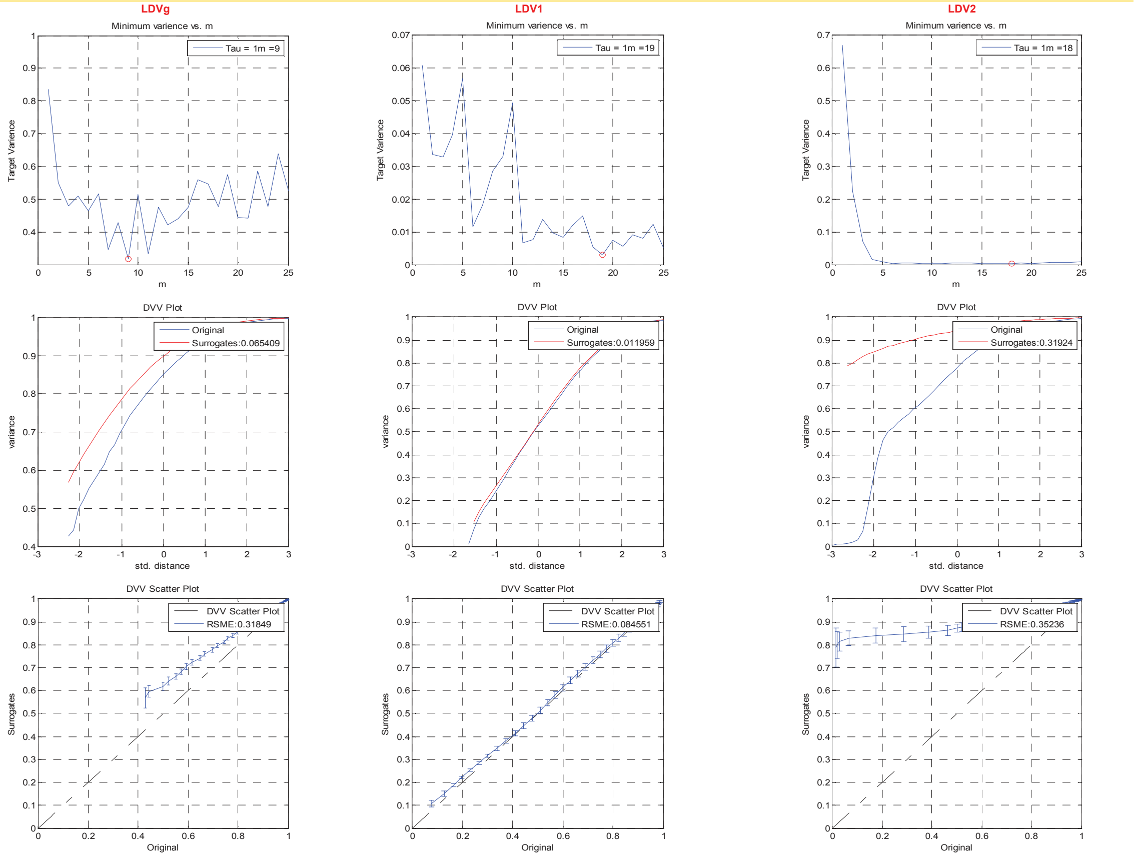
LDVg
LDV1
LDV2
Minimum varience vs. m
Tau = 1m =9
Tau = 1m =19
Tau = 1m =18
Target Varience
m
DVV Plot
Original
Surrogates:0.065409
Surrogates:0.011959
Surrogates:0.31924
variance
std. distance
DVV Scatter Plot
RSME:0.31849
RSME:0.084551
RSME:0.35236
Surrogates
Original

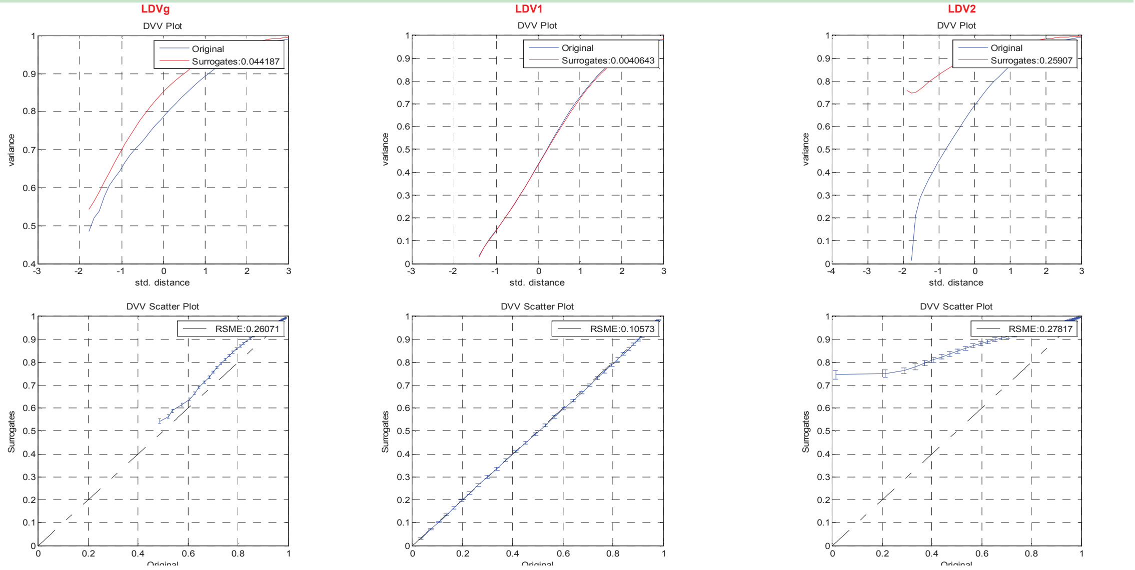
LDVg
LDV1
LDV2
DVV Plot
Original
Surrogates:0.044187
Surrogates:0.0040643
Surrogates:0.25907
variance
std. distance
DVV Scatter Plot
RSME:0.26071
RSME:0.10573
RSME:0.27817
Surrogates

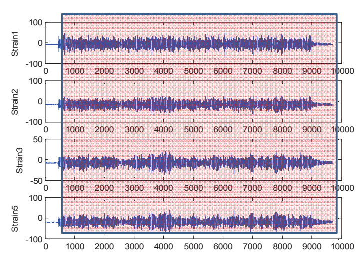

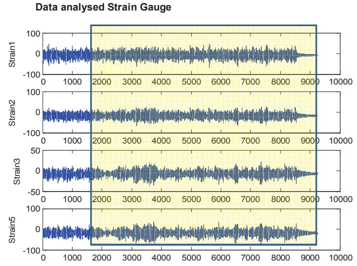

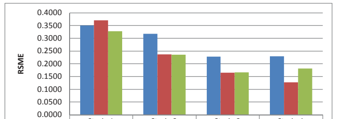

	Strain 1	Strain 2	Strain 3	Strain 4
METHOD 1	0.3504	0.3172	0.2279	0.2298
METHOD 2	0.3699	0.2367	0.1652	0.1269
METHOD 3	0.3280	0.2352	0.1664	0.1815

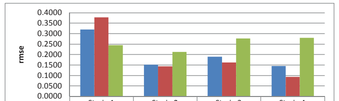

	Strain 1	Strain 2	Strain 3	Strain 4
METHOD 1	0.3199	0.1514	0.1905	0.1453
METHOD 2	0.3787	0.1445	0.1627	0.0940
METHOD 3	0.2443	0.2132	0.2765	0.2801

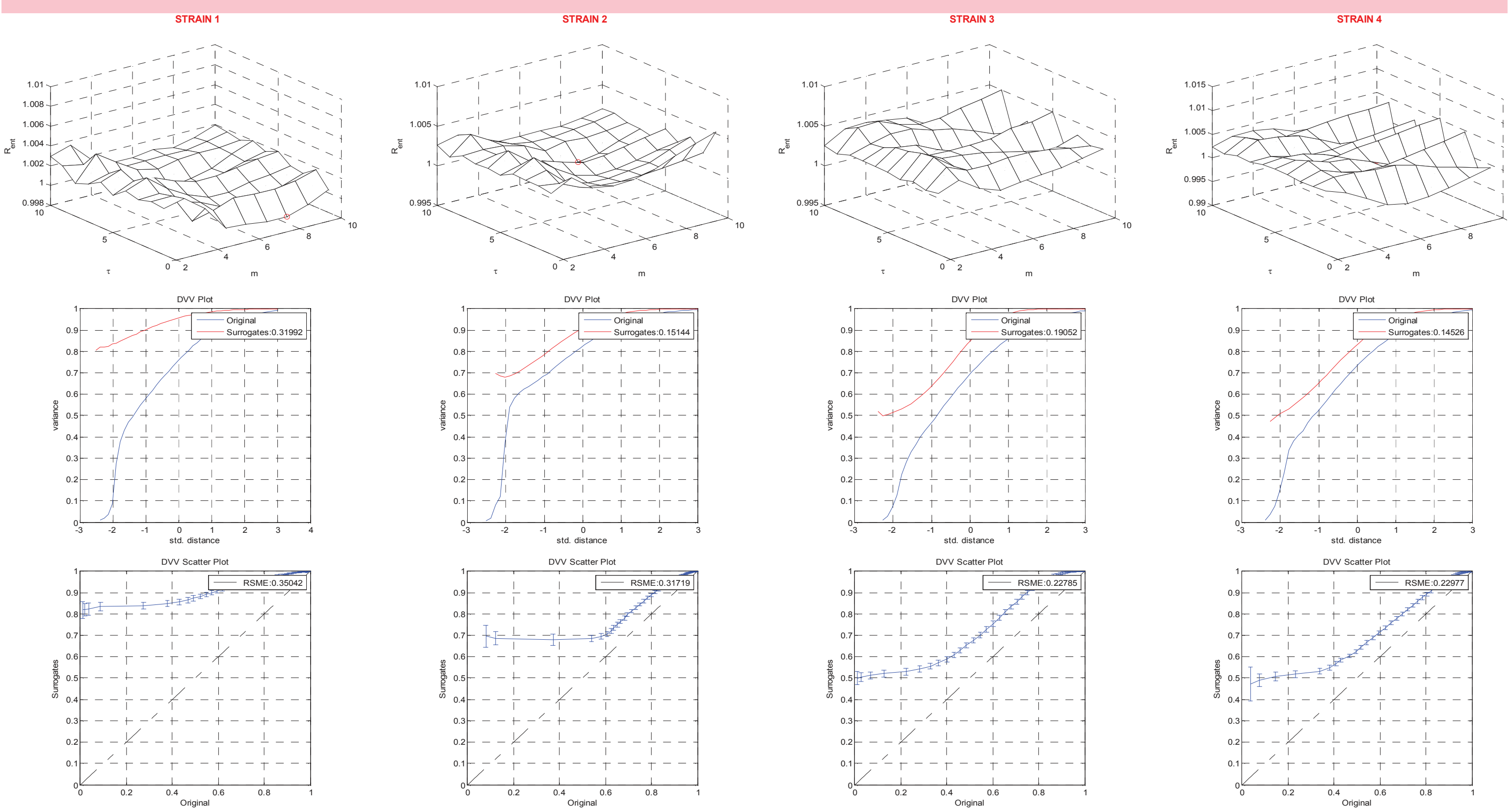

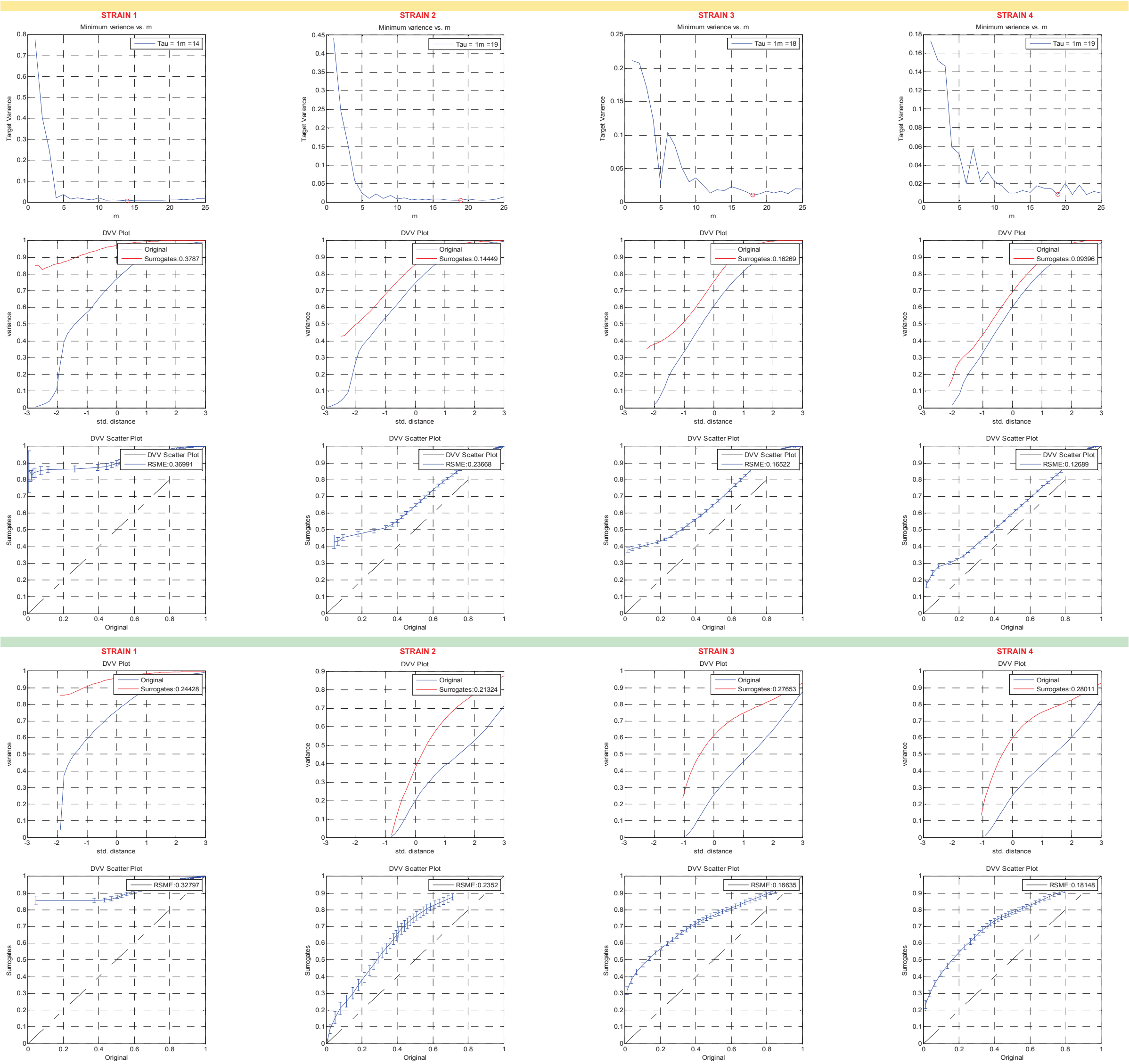
STRAIN 1
STRAIN 2
STRAIN 3
STRAIN 4
Minimum varience vs. m
Target Varience
m
Tau = 1m =14
Tau = 1m =19
Tau = 1m =18
Tau = 1m =19
DVV Plot
variance
std. distance
Original
Surrogates:0.3787
Surrogates:0.14449
Surrogates:0.16269
Surrogates:0.09396
DVV Scatter Plot
Surrogates
Original
RSME:0.36991
RSME:0.23668
RSME:0.16522
RSME:0.12689
STRAIN 1
STRAIN 2
STRAIN 3
STRAIN 4
Surrogates:0.24428
Surrogates:0.21324
Surrogates:0.27653
Surrogates:0.28011
RSME:0.32797
RSME:0.2352
RSME:0.16635
RSME:0.18148

EXPERIMENT	VARIABLES		METHOD 1				METHOD 2				METHOD 3			
			best **m**	best **τ**	rsme	RSME	calc **m**	**τ**	rsme	RSME	set **m**	set **τ**	rsme	RSME
16	Loading Sine Sweep<br>2.0 Hz 6.0 Hz<br>60 sec<br>Focus at bottom strain gauge	CH1	4	10	0.0716	0.1065	25	1	0.0620	0.1974	3	1	0.0354	0.2434
		CH2	2	5	0.0396	0.2383	7	1	0.0333	0.0989	3	1	0.0144	0.0673
		CH3	3	10	0.0141	0.1101	9	1	0.0089	0.1931	3	1	0.0087	0.2193
		LDVg	10	2	0.1700	0.1688	21	1	0.1494	0.1387	3	1	0.0528	0.1211
		LDV1	6	6	0.1758	0.1823	11	1	0.0872	0.1796	3	1	0.0314	0.2079
		LDV2	8	8	0.3659	0.2303	20	1	0.3000	0.1593	3	1	0.2509	0.1316
		Strain 1	10	10	0.1578	0.1270	9	1	0.2749	0.1510	3	1	0.2501	0.1336
		Strain 2	9	9	0.1196	0.1483	6	1	0.2365	0.1597	3	1	0.1830	0.1559
		Strain 3	10	10	0.4309	0.2256	10	1	0.3394	0.1691	3	1	0.2680	0.1610
		Strain 4	9	9	0.3920	0.2015	5	1	0.2818	0.1535	3	1	0.2522	0.1629

Data recorded 3D Accelerometer

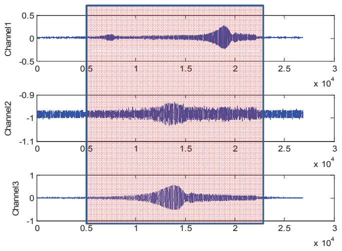

Data analysed 3D Accelerometer

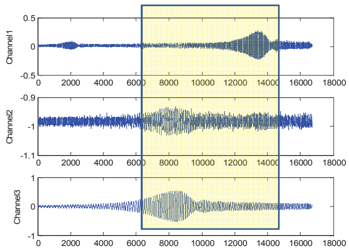

METHOD 1

CH1

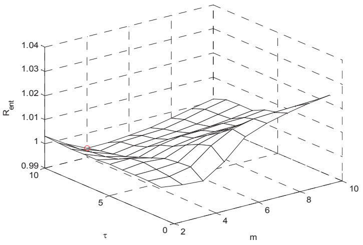

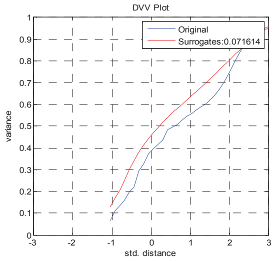

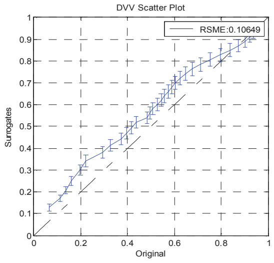

CH2

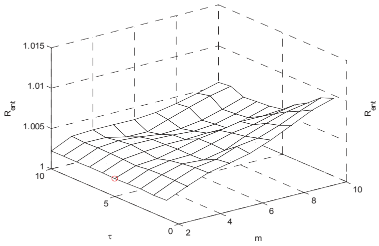

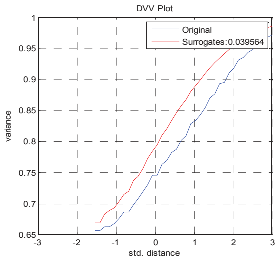

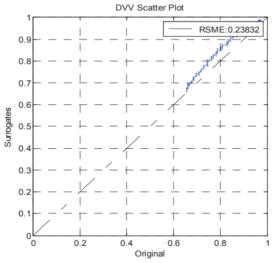

CH3

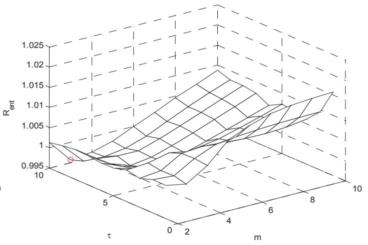

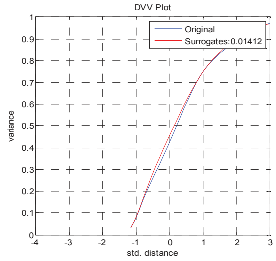

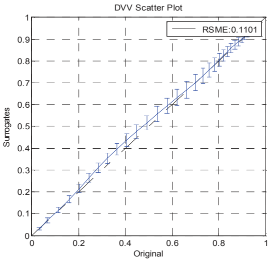

METHOD 2

CH1

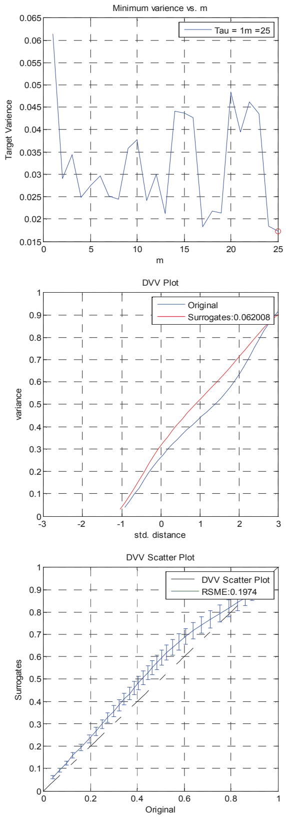

CH2

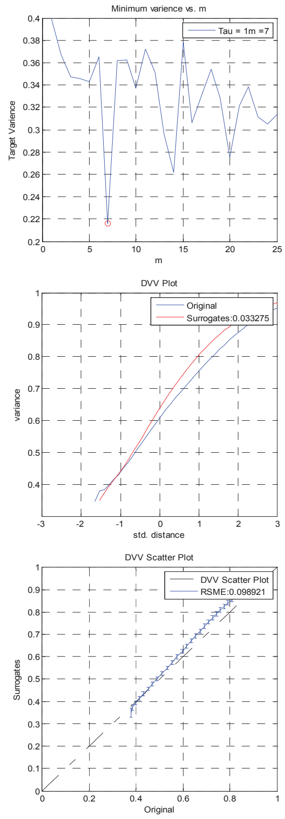

CH3

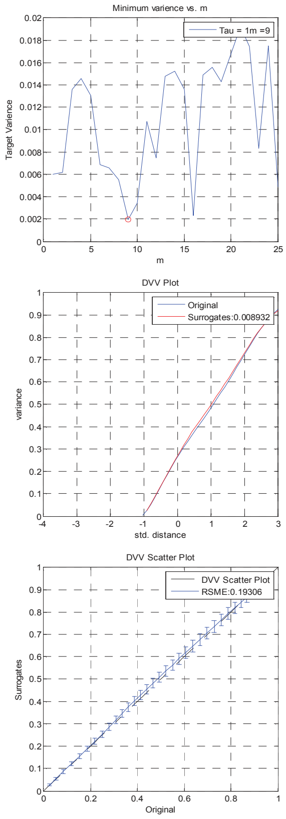

METHOD 3

CH1

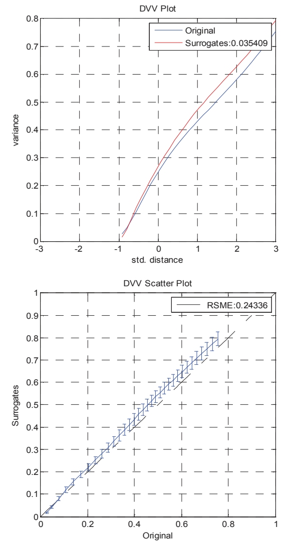

CH2

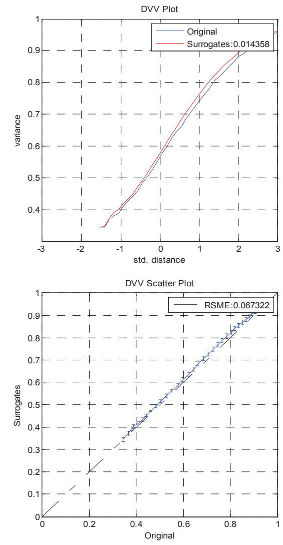

CH3

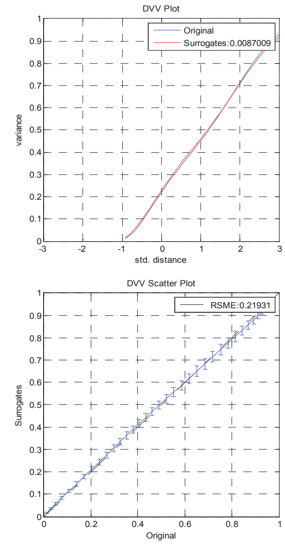

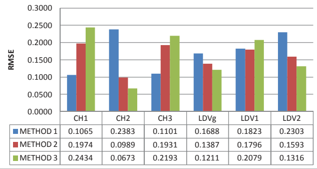

	CH1	CH2	CH3	LDVg	LDV1	LDV2
METHOD 1	0.1065	0.2383	0.1101	0.1688	0.1823	0.2303
METHOD 2	0.1974	0.0989	0.1931	0.1387	0.1796	0.1593
METHOD 3	0.2434	0.0673	0.2193	0.1211	0.2079	0.1316

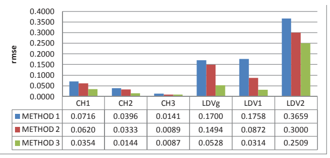

	CH1	CH2	CH3	LDVg	LDV1	LDV2
METHOD 1	0.0716	0.0396	0.0141	0.1700	0.1758	0.3659
METHOD 2	0.0620	0.0333	0.0089	0.1494	0.0872	0.3000
METHOD 3	0.0354	0.0144	0.0087	0.0528	0.0314	0.2509

Data recorded LDV

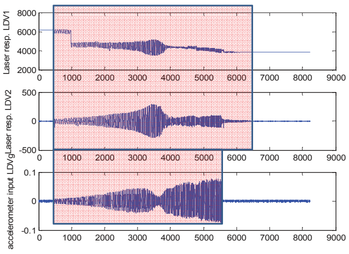

Data analysed LDV

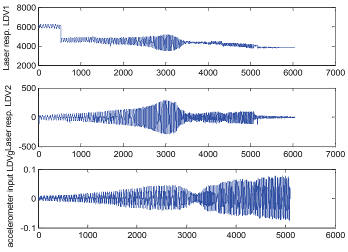

LDVg **LDV1** **LDV2**

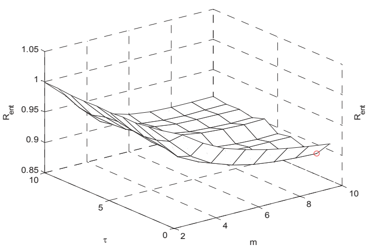

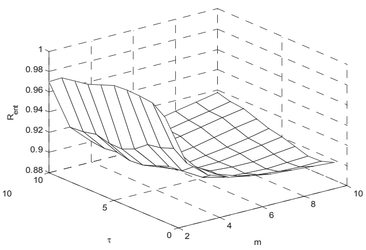

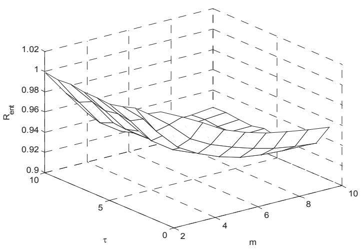

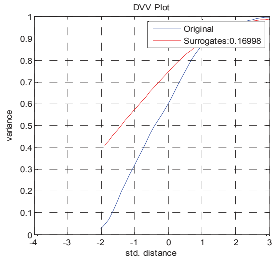

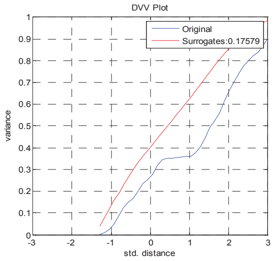

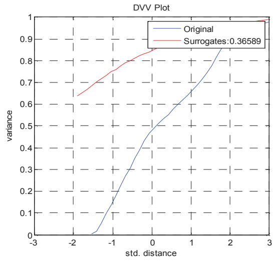

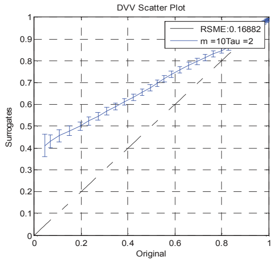

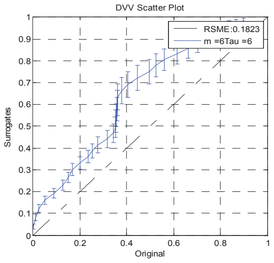

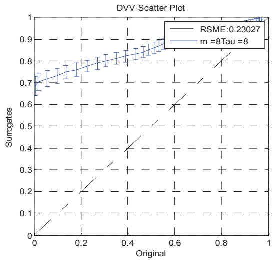

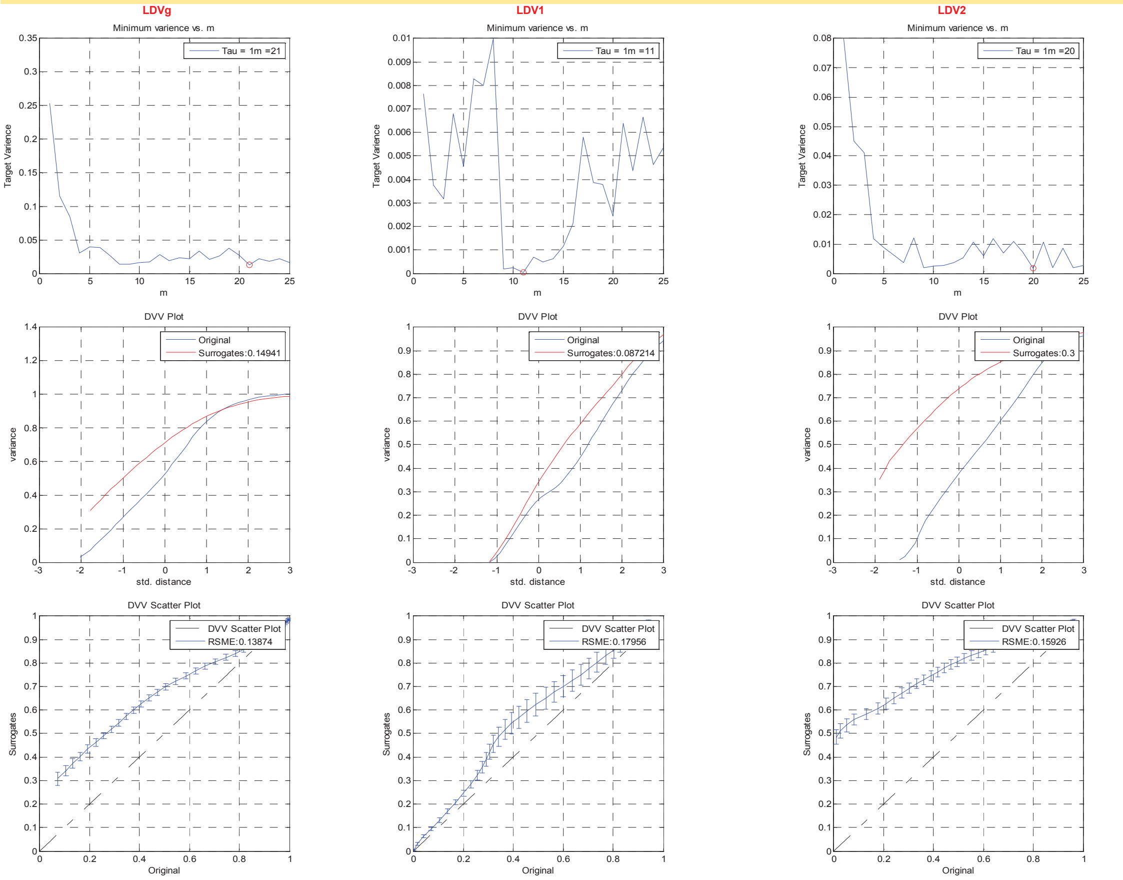
LDVg
LDV1
LDV2
Minimum varience vs. m
Tau = 1m =21
Tau = 1m =11
Tau = 1m =20
Target Varience
m
DVV Plot
Original
Surrogates:0.14941
Surrogates:0.087214
Surrogates:0.3
variance
std. distance
DVV Scatter Plot
RSME:0.13874
RSME:0.17956
RSME:0.15926
Surrogates
Original

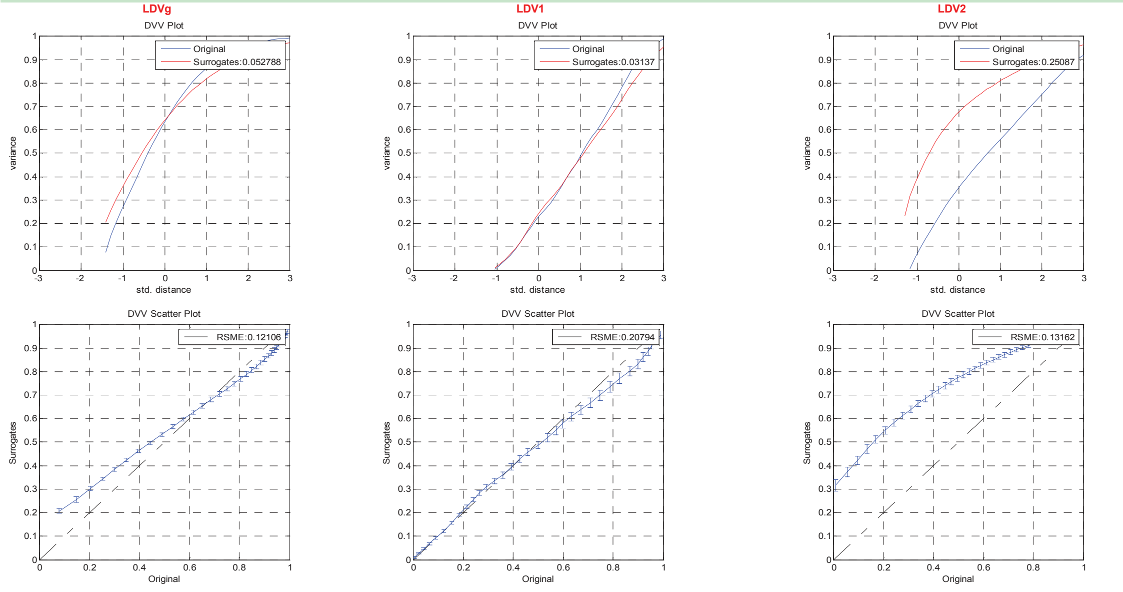
LDVg
LDV1
LDV2
DVV Plot
Original
Surrogates:0.052788
Surrogates:0.03137
Surrogates:0.25087
variance
std. distance
DVV Scatter Plot
RSME:0.12106
RSME:0.20794
RSME:0.13162
Surrogates
Original

Data recorded Strain Gauge

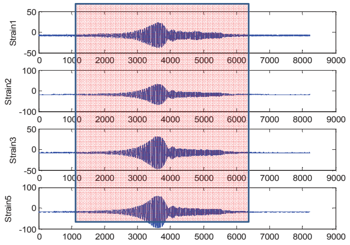

Data analysed Strain Gauge

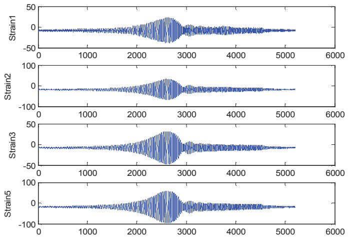

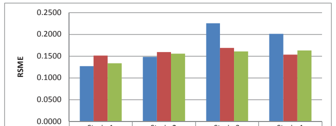

	Strain 1	Strain 2	Strain 3	Strain 4
METHOD 1	0.1270	0.1483	0.2256	0.2015
METHOD 2	0.1510	0.1597	0.1691	0.1535
METHOD 3	0.1336	0.1559	0.1610	0.1629

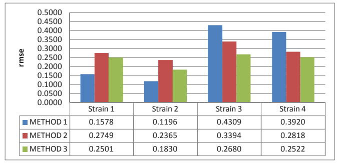

	Strain 1	Strain 2	Strain 3	Strain 4
METHOD 1	0.1578	0.1196	0.4309	0.3920
METHOD 2	0.2749	0.2365	0.3394	0.2818
METHOD 3	0.2501	0.1830	0.2680	0.2522

STRAIN 1

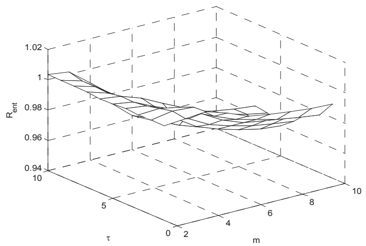

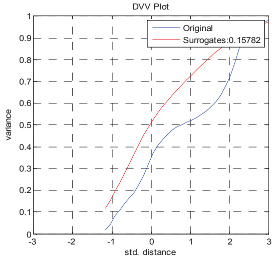

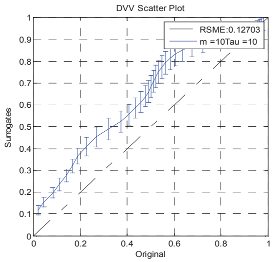

STRAIN 2

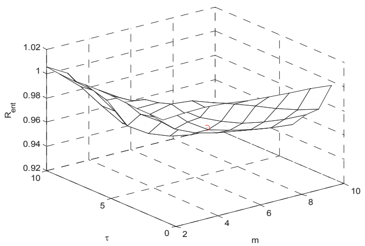

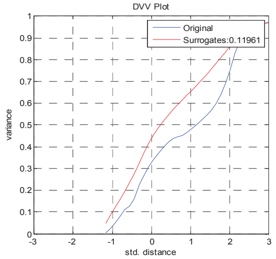

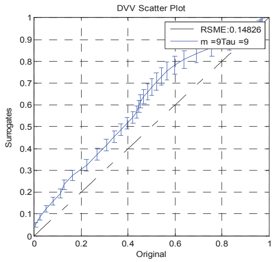

STRAIN 3

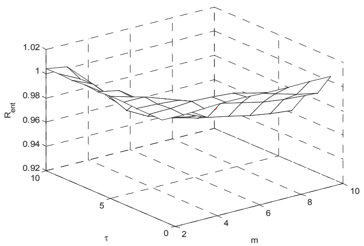

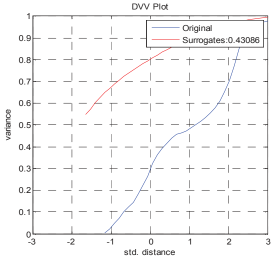

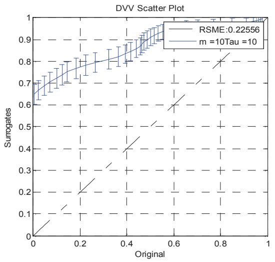

STRAIN 4

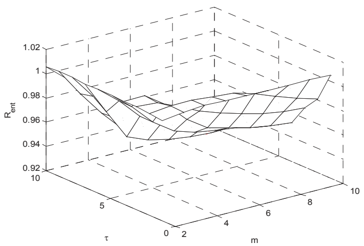

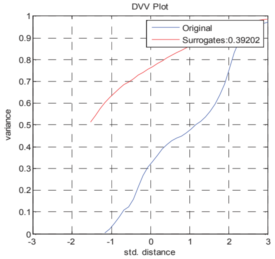

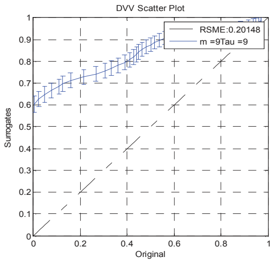

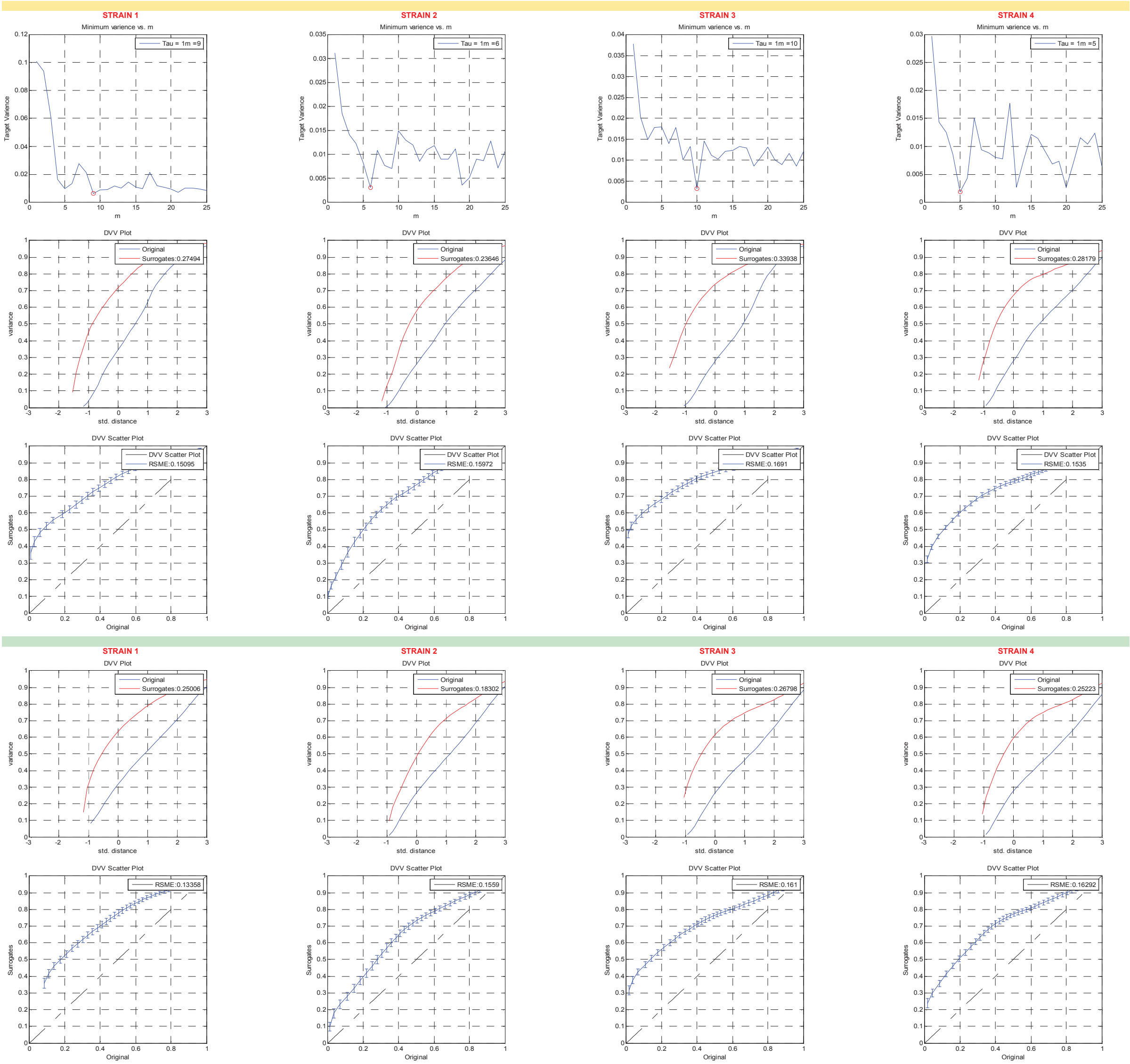

STRAIN 1
STRAIN 2
STRAIN 3
STRAIN 4
Minimum varience vs. m
Target Variance
m
Tau = 1m =9
Tau = 1m =6
Tau = 1m =10
Tau = 1m =5
DVV Plot
variance
std. distance
Original
Surrogates:0.27494
Surrogates:0.23646
Surrogates:0.33938
Surrogates:0.28179
DVV Scatter Plot
Surrogates
Original
RSME:0.15095
RSME:0.15972
RSME:0.1691
RSME:0.1535
STRAIN 1
STRAIN 2
STRAIN 3
STRAIN 4
Surrogates:0.25006
Surrogates:0.18302
Surrogates:0.26798
Surrogates:0.25223
RSME:0.13358
RSME:0.1559
RSME:0.161
RSME:0.16292

EXPERIMENT	VARIABLES		METHOD 1				METHOD 2				METHOD 3			
			best **m**	best **τ**	rsme	RSME	calc **m**	**τ**	rsme	RSME	set **m**	set **τ**	rsme	RSME
17	Harmonic resonance<br>2.0 Hz 2.5 Hz<br>3.0 Hz 3.5 Hz<br>4.0 Hz 4.2 Hz<br>4.3 Hz 4.4 Hz<br>4.5 Hz 4.6 Hz<br>5.0Hz 5.5 Hz<br>6.0 Hz 6.5Hz<br>7.0Hz<br>Focus at bottom strain gauge.	CH1	7	10	0.1119	0.1255	22	1	0.1015	0.1965	3	1	0.0530	0.2385
		CH2	6	10	0.0359	0.1084	17	1	0.0197	0.0814	3	1	0.0073	0.0491
		CH3	4	10	0.0191	0.1464	14	1	0.0181	0.1311	3	1	0.0152	0.1867
		LDVg	10	8	0.2920	0.2477	13	1	0.1010	0.0961	3	1	0.0445	0.1009
		LDV1	5	9	0.0212	0.1582	11	1	0.0163	0.1671	3	1	0.0111	0.1873
		LDV2	8	7	0.3506	0.2324	21	1	0.3050	0.1632	3	1	0.2263	0.1206
		Strain 1	10	7	0.3605	0.2185	15	1	0.3875	0.2077	3	1	0.2459	0.1285
		Strain 2	9	7	0.3372	0.1942	10	1	0.2350	0.1589	3	1	0.2011	0.1230
		Strain 3	10	7	0.4138	0.2289	10	1	0.2820	0.1681	3	1	0.2267	0.1304
		Strain 4	9	7	0.3715	0.2099	9	1	0.2784	0.1719	3	1	0.2293	0.1324

Data recorded 3D Accelerometer

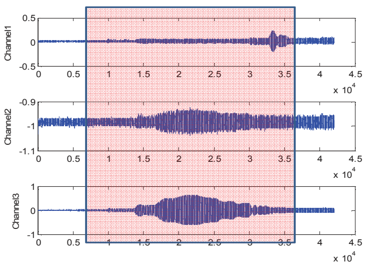

Data analysed 3D Accelerometer

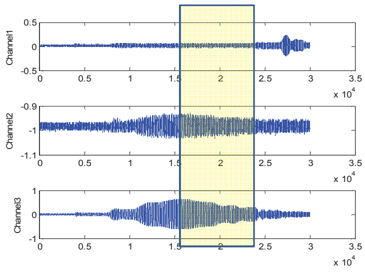

METHOD 1

CH1 **CH2** **CH3**

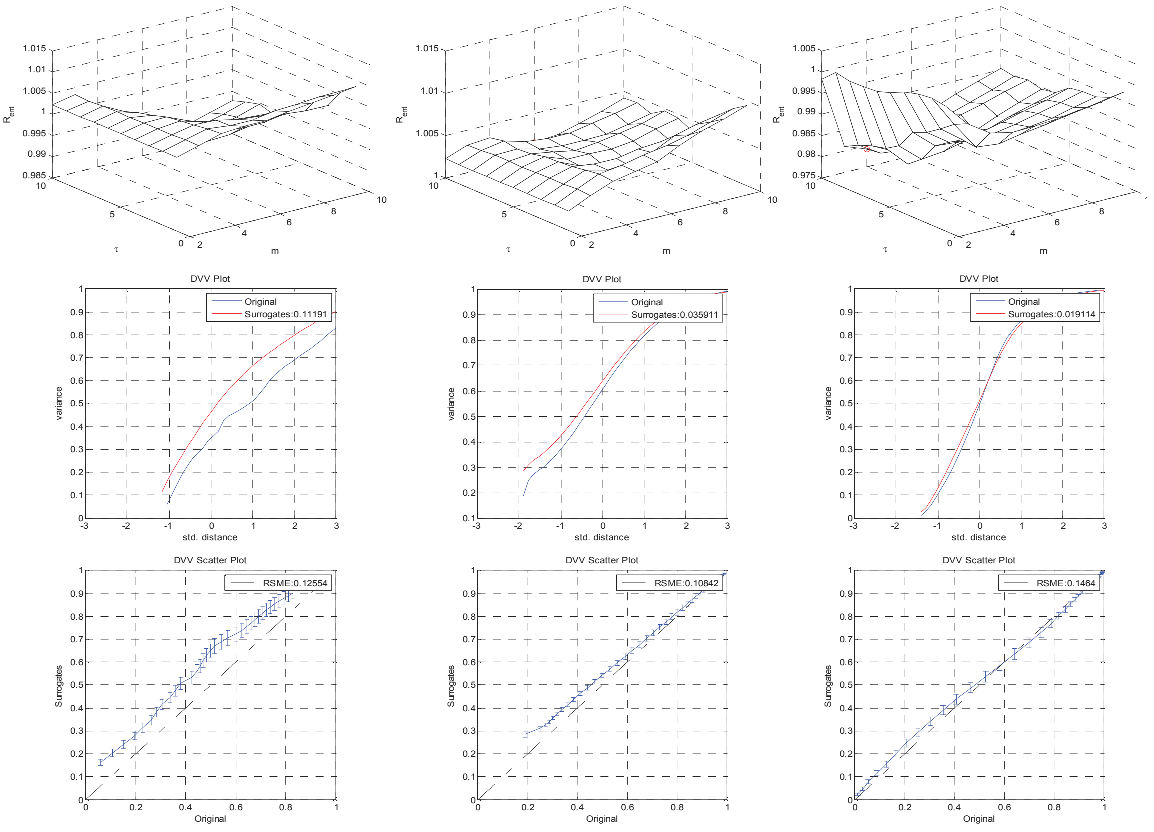

METHOD 2

CH1 **CH2** **CH3**

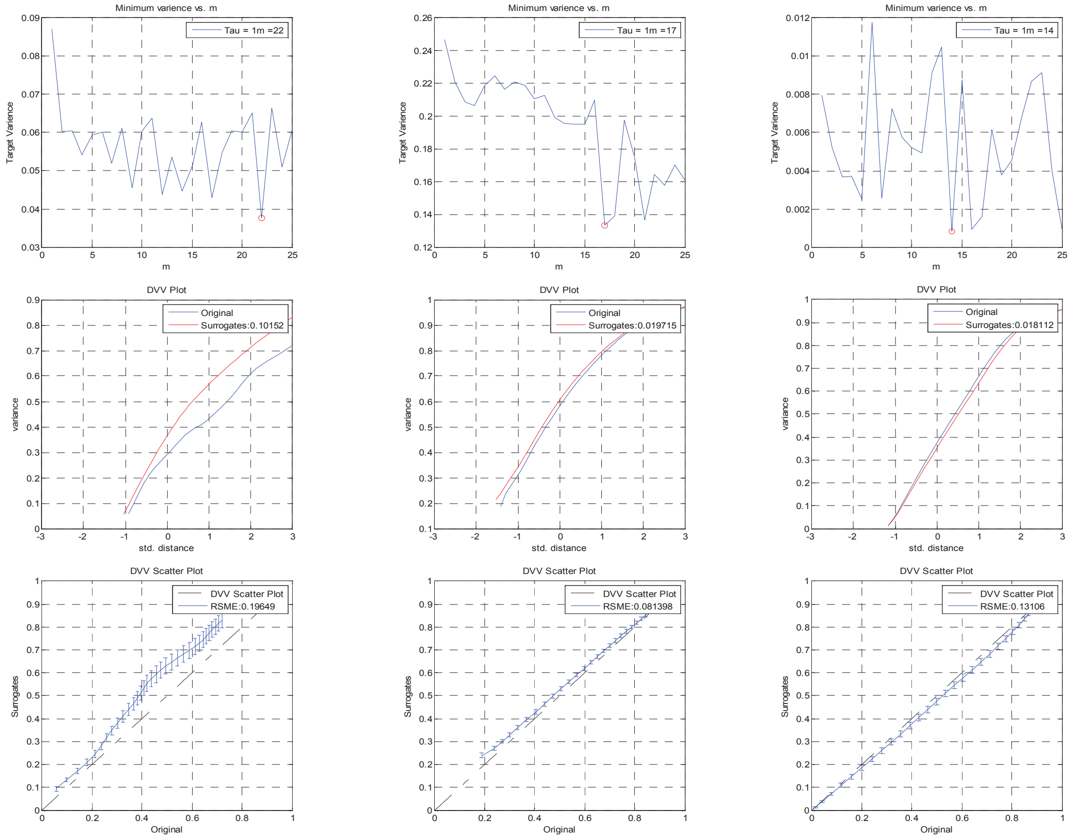

METHOD 3

CH1 **CH2** **CH3**

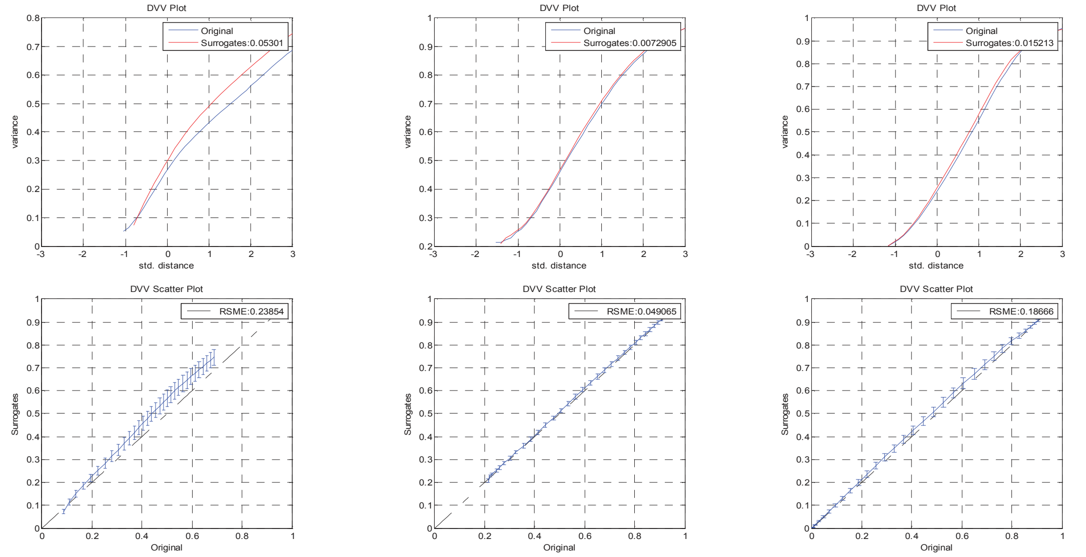

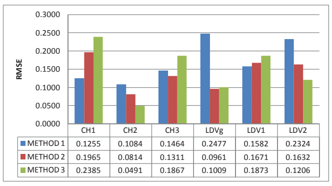

	CH1	CH2	CH3	LDVg	LDV1	LDV2
METHOD 1	0.1255	0.1084	0.1464	0.2477	0.1582	0.2324
METHOD 2	0.1965	0.0814	0.1311	0.0961	0.1671	0.1632
METHOD 3	0.2385	0.0491	0.1867	0.1009	0.1873	0.1206

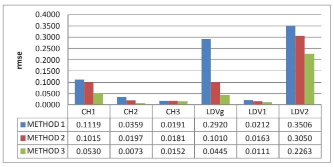

	CH1	CH2	CH3	LDVg	LDV1	LDV2
METHOD 1	0.1119	0.0359	0.0191	0.2920	0.0212	0.3506
METHOD 2	0.1015	0.0197	0.0181	0.1010	0.0163	0.3050
METHOD 3	0.0530	0.0073	0.0152	0.0445	0.0111	0.2263

Data recorded LDV

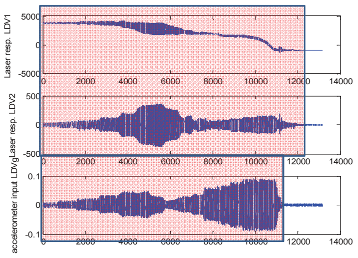

Data analysed LDV

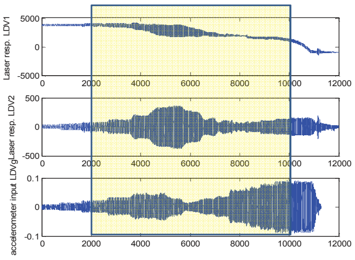

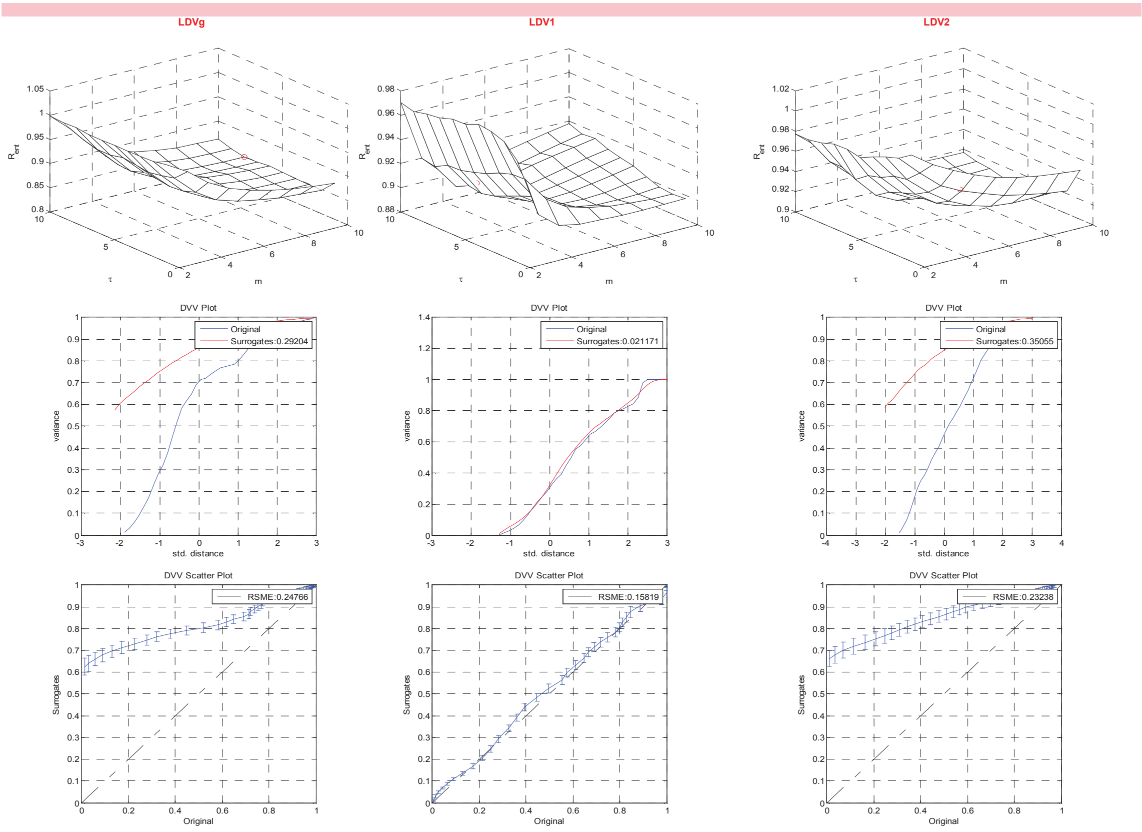

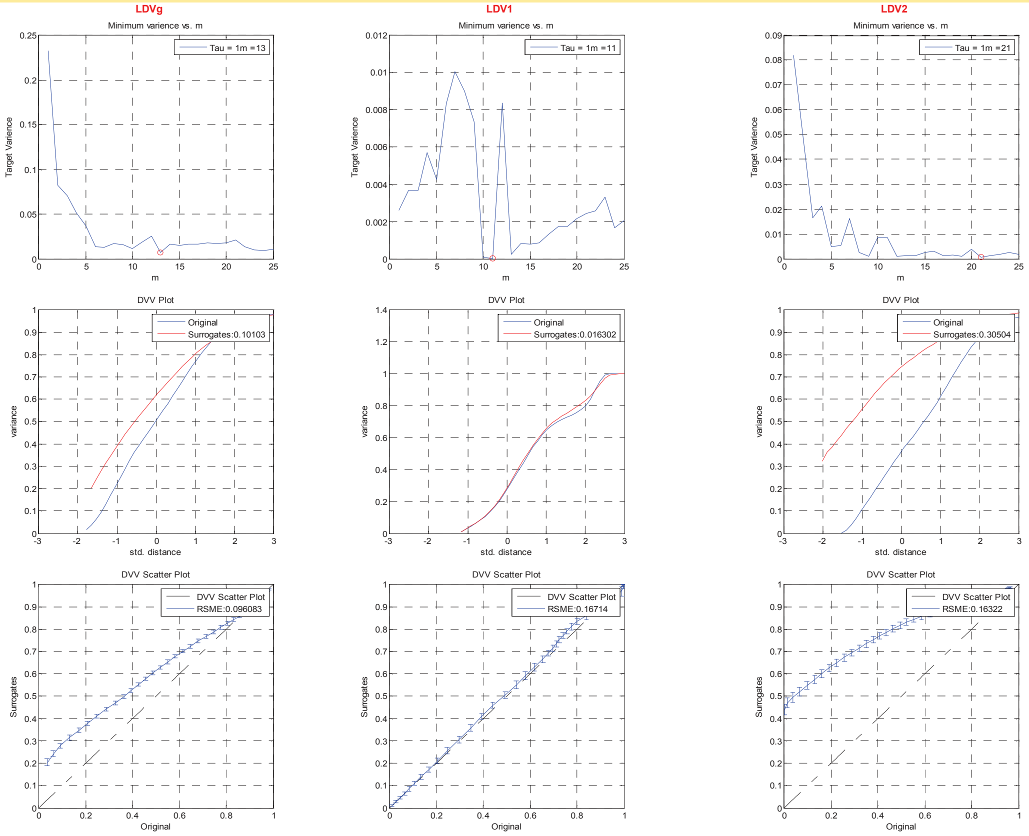
LDVg
LDV1
LDV2
Minimum varience vs. m
Tau = 1m =13
Tau = 1m =11
Tau = 1m =21
Target Varience
m
DVV Plot
Original
Surrogates:0.10103
Surrogates:0.016302
Surrogates:0.30504
variance
std. distance
DVV Scatter Plot
RSME:0.096083
RSME:0.16714
RSME:0.16322
Surrogates
Original

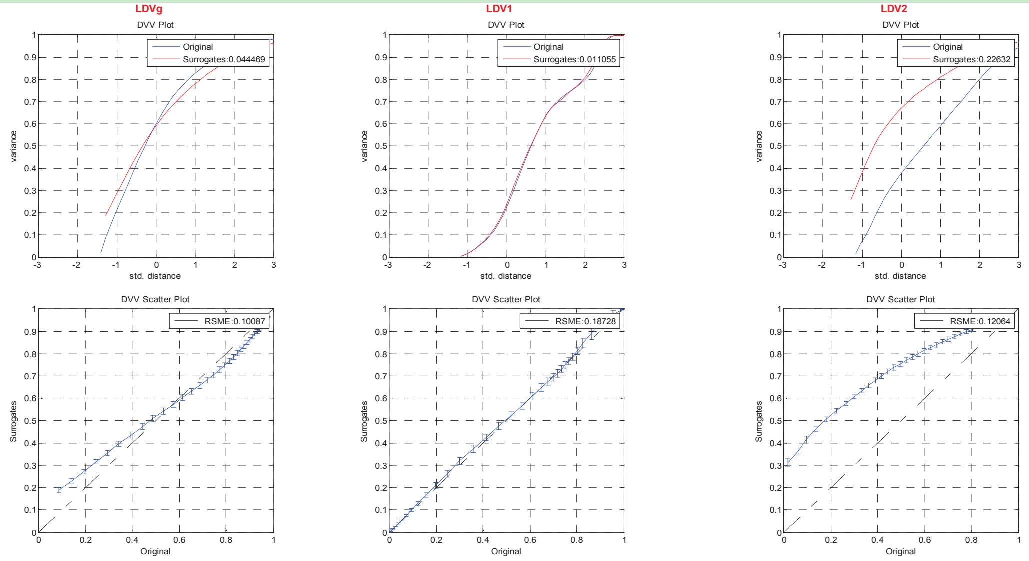
LDVg
LDV1
LDV2
DVV Plot
Original
Surrogates:0.044469
Surrogates:0.011055
Surrogates:0.22632
variance
std. distance
DVV Scatter Plot
RSME:0.10087
RSME:0.18728
RSME:0.12064
Surrogates
Original

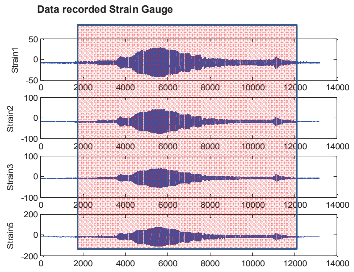

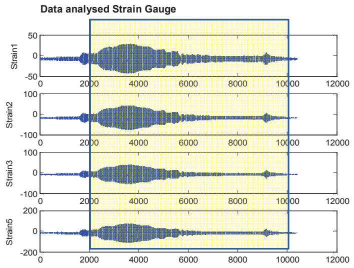

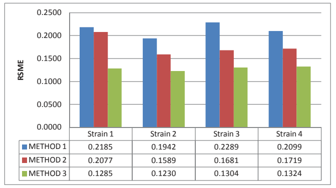

	Strain 1	Strain 2	Strain 3	Strain 4
METHOD 1	0.2185	0.1942	0.2289	0.2099
METHOD 2	0.2077	0.1589	0.1681	0.1719
METHOD 3	0.1285	0.1230	0.1304	0.1324

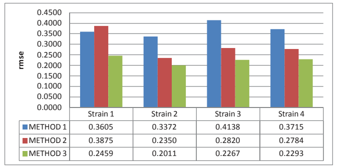

	Strain 1	Strain 2	Strain 3	Strain 4
METHOD 1	0.3605	0.3372	0.4138	0.3715
METHOD 2	0.3875	0.2350	0.2820	0.2784
METHOD 3	0.2459	0.2011	0.2267	0.2293

STRAIN 1

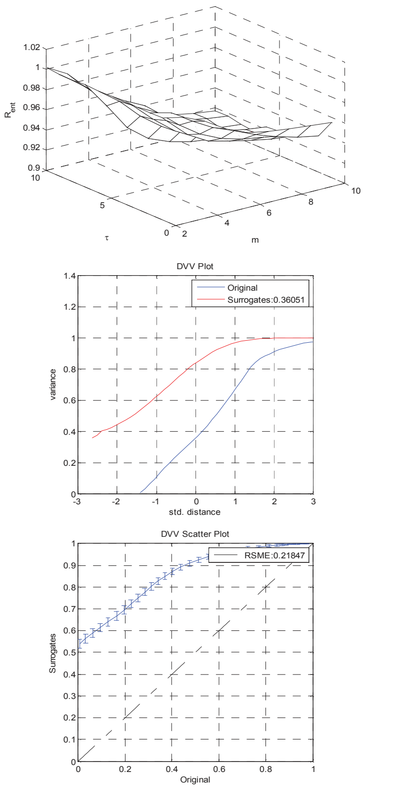

STRAIN 2

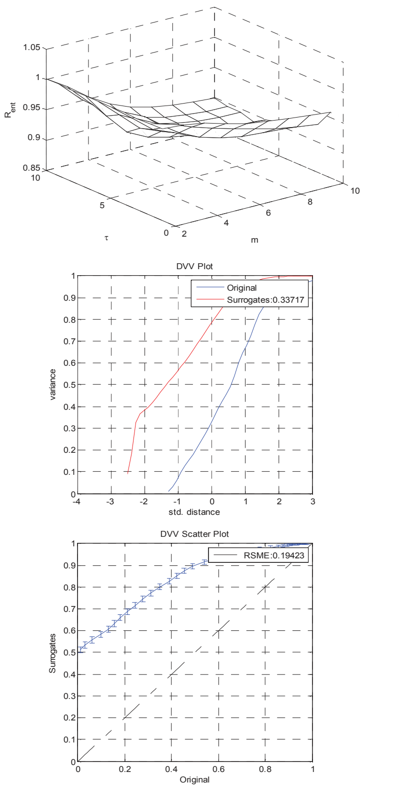

STRAIN 3

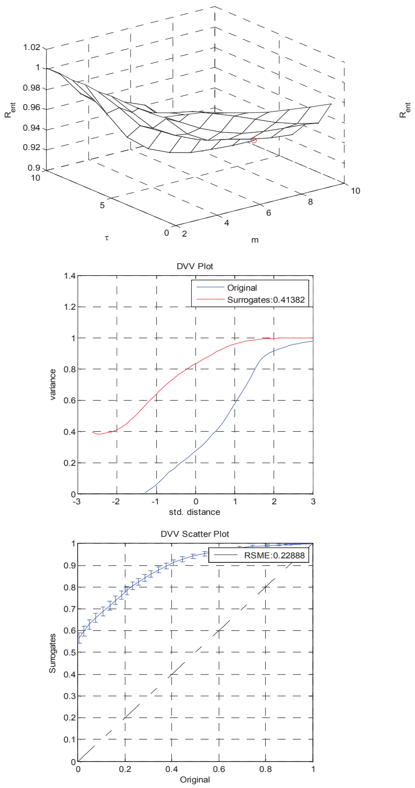

STRAIN 4

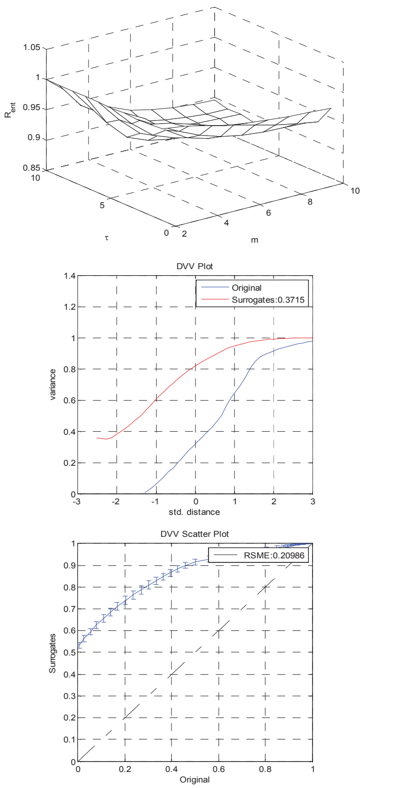

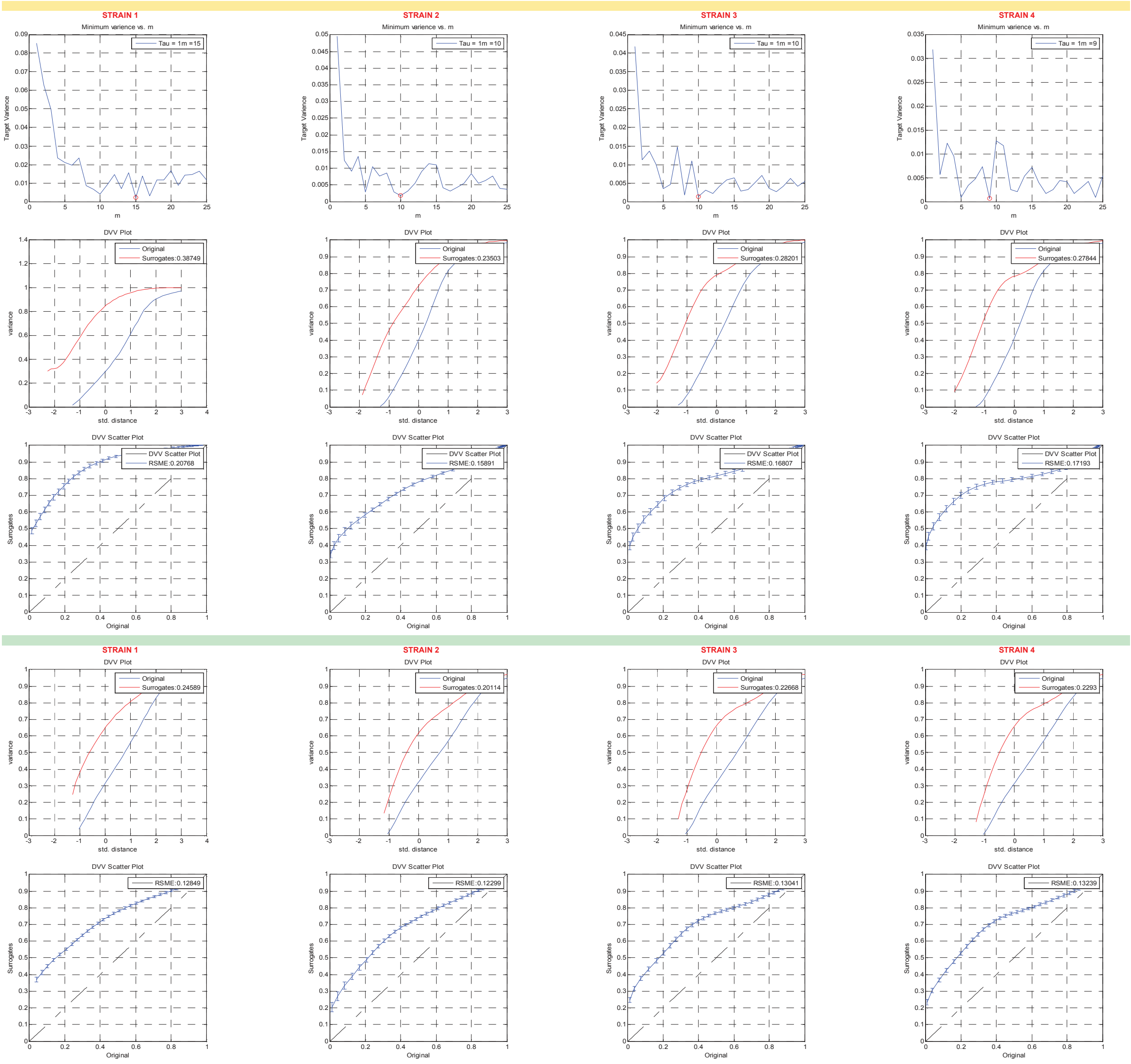

STRAIN 1
STRAIN 2
STRAIN 3
STRAIN 4
Minimum varience vs. m
Tau = 1m =15
Tau = 1m =10
Tau = 1m =10
Tau = 1m =9
Target Varience
m
DVV Plot
Original
Surrogates:0.38749
Surrogates:0.23503
Surrogates:0.28201
Surrogates:0.27844
variance
std. distance
DVV Scatter Plot
RSME:0.20768
RSME:0.15891
RSME:0.16807
RSME:0.17193
Surrogates
Original
STRAIN 1
STRAIN 2
STRAIN 3
STRAIN 4
Surrogates:0.24589
Surrogates:0.20114
Surrogates:0.22668
Surrogates:0.2293
RSME:0.12849
RSME:0.12299
RSME:0.13041
RSME:0.13239
